# Phylogenomic Characterization of a Novel *Corynebacterium* Species Associated with Fatal Diphtheritic Stomatitis in Endangered Yellow-Eyed Penguins

**DOI:** 10.1128/mSystems.00320-21

**Published:** 2021-06-08

**Authors:** Sarah C. Saunderson, Imen Nouioui, Anne C. Midwinter, David A. Wilkinson, Melanie J. Young, Kate M. McInnes, Jim Watts, Vartul Sangal

**Affiliations:** a Department of Microbiology and Immunology, School of Biomedical Sciences, University of Otago, Dunedin, New Zealand; b Leibniz-Institut DSMZ–Deutsche Sammlung von Mikroorganismen und Zellkulturen GmbH, Braunschweig, Germany; c School of Natural and Environmental Sciences, Faculty of Science, Agriculture, and Engineering, Newcastle University, Newcastle upon Tyne, United Kingdom; d Molecular Epidemiology and Veterinary Public Health Laboratory (mEpiLab), Infectious Disease Research Centre, School of Veterinary Science, Massey University, Palmerston North, New Zealand; e Science and Capability, Department of Conservation, Wellington, New Zealand; f Department of Zoology, University of Otago, Dunedin, New Zealand; g Coastal Otago District Office, Department of Conservation, Dunedin, New Zealand; h Faculty of Health and Life Sciences, Northumbria University, Newcastle upon Tyne, United Kingdom; University of Trento

**Keywords:** *Corynebacterium*, virulence genes, yellow-eyed penguin, diphtheritic stomatitis, *Megadyptes antipodes*, core genome, novel species

## Abstract

Yellow-eyed penguins, Megadyptes antipodes, are an endangered species that are endemic to New Zealand. Outbreaks of diphtheritic stomatitis have caused significant mortality for this species, especially among young chicks. In this study, we isolated 16 *Corynebacterium* sp. isolates from the oral cavities of 2- to 14-day-old chicks at a range of infection stages and sequenced the genomes to understand their virulence mechanisms. Phylogenomic and matrix-assisted laser desorption ionization–time of flight (MALDI-TOF) characterization indicate that these strains belong to a novel *Corynebacterium* species. A simple multiplex PCR-based diagnostic assay has been developed to identify these strains rapidly and reliably. Similar to other corynebacteria, genomic islands and prophages introduced significant diversity among these strains that has potentially led to minor functional variations between the two lineages. Despite the presence of multiple corynebacterial virulence genes and a *spaDEF*-type pilus gene cluster among these strains, the survival rate was much higher in Galleria mellonella larvae than in those inoculated with Corynebacterium ulcerans NZRM 818 and Corynebacterium pseudotuberculosis NZRM 3004. Therefore, these strains are opportunistic pathogens causing high mortality among young penguin chicks due to a less-developed immune system.

**IMPORTANCE** Yellow-eyed penguins, Megadyptes antipodes, are endangered species with a sharp decline in the numbers of breeding pairs over the last 2 decades. Diphtheritic stomatitis, characterized by a thick fibrinopurulent exudate in the oral cavities and symptoms, including inanition and significant weight loss, is responsible for significant mortality among the young chicks. These chicks are treated with antibiotics, amoxicillin-clavulanic acid or enrofloxacin, but do not always recover from the infection. The pathogen causing these infections and the mechanism of pathogenesis are unclear. This study has identified a novel *Corynebacterium* species to be associated with diphtheritic stomatitis in yellow-eyed penguins with potential virulence genes that are likely involved in pathogenesis. Importantly, a gene encoding an exotoxin, phospholipase D, is present among these strains. The inactivated form of this enzyme could potentially be used as an effective vaccine to protect these penguins from infection.

## INTRODUCTION

Yellow-eyed penguins (Megadyptes antipodes), or hoiho (te reo Māori), are one of the world’s rarest penguin species and are endemic to New Zealand and its outlying islands (1). Over the last 2 decades, the numbers of breeding pairs have significantly declined due to a range of factors, including climate change (2), direct and indirect conflicts with commercial fisheries (3, 4), starvation events, and unexplained adult mortalities (5). Underpinning this decline in adult and juvenile numbers are mass mortality events affecting chick production (5–7), including starvation and oral infections known as diphtheritic stomatitis (5). Diphtheritic stomatitis is responsible for high mortality rates among 1- to 28-day-old chicks, which is characterized by a thick cream-colored fibrinopurulent exudate in the oral cavities and symptoms, including inanition and significant weight loss leading to death (7, 8). Corynebacterial strains, identified as Corynebacterium amycolatum, were previously isolated from the oral lesions of infected chicks (8). However, similar strains were also isolated from the oral cavity of healthy adult penguins (8). While the presence of avipoxvirus and an unidentified virus-like agent have been detected among the infected chicks (8), their roles with regard to infection and mortality remain unclear.

First described by Lehmann and Neumann in 1896, the genus *Corynebacterium* comprises >130 species and 11 subspecies (9, 10). *Corynebacterium* strains are Gram-stain-positive rod- or club-shaped bacteria characterized by genomic G+C content between 46 and 74 mol% (9). Several corynebacterial species are of biotechnological, medical, or veterinary importance (9, 10). In this study, we report the findings on novel *Corynebacterium* strains isolated from lesions derived from the oral cavities of 2- to 14-day-old yellow-eyed penguin chicks. The phylogenomic characterization identified two distinct lineages with potential virulence genes, which may be associated with diphtheritic stomatitis.

## RESULTS

### Clinical information related to bacterial isolates.

**FIG 1:**
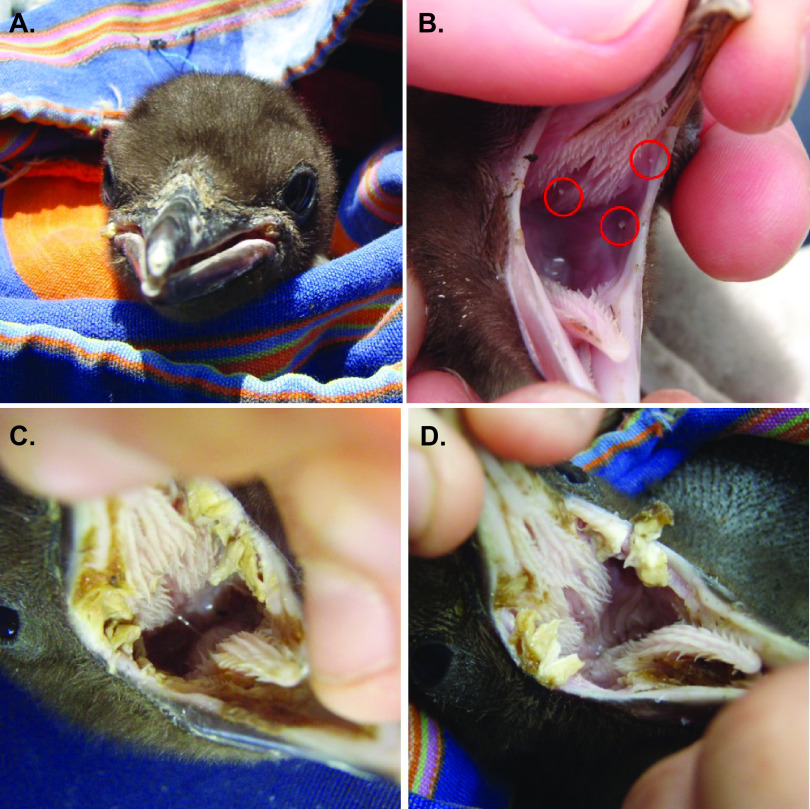
Fibrinopurulent diphtheria lesions in yellow-eyed penguin chicks. (A) Lesions at the commissures of the beak, which prevent closure of the oral cavity. (B) Red circles denote minor “pinpoint” lesions in the gape and choanal slit that are firmly adhered to the mucosa before overgrowth of fibrinopurulent lesion exudate and mucosa ulceration. (C and D) Extensive fibrinopurulent diphtheria lesions in the oral cavity of infected yellow-eyed penguin chicks, including both sides of the gape and under the tongue. Photos: M. J. Young.

Yellow-eyed penguin chicks (2 to 14 days old) at a range of diphtheritic stomatitis infection stages were identified within breeding regions of the Otago Peninsula (Table 1; Fig. 1; Fig. S1). Lesion exudate was swabbed in 10 chicks presenting with oral lesions consistent with the disease syndrome. The oral cavity of five chicks with no visible lesions were also swabbed from nests that were classed as infected due to a previous or an ongoing infection. In particular, two chicks, YEP-20 and YEP-50, previously possessed lesions that were debrided less than a week before the sampling. One chick, YEP-52, shared a nest with a twin chick that had a previous lesion debridement recorded. All three chicks developed lesions within 1-week postsampling. No previous lesions were observed in the twin chicks (YEP-5 and YEP-6) from the same nest; however, the former was found deceased of unknown causes 3 days postsampling, while the latter chick developed lesions 22 days later. The lesion status and nest details for one sample (YEP-12) are not recorded.

**TABLE 1:** Details of genome assemblies of *Corynebacterium* sp. isolates from penguins

Strain^*a*^	Breeding area	Chick ID	Swab site	Lineage	Approx coverage (×)	*N*_50_ (bp)	No. of contigs	Total size (Mb)	GC content (%)	No. of CDS^*b*^	No. of tRNAs	No. of tmRNAs
3B	Highcliff	YEP-3	Lesion	1	64	241,725	16	2.45	62.9	2,138	52	1
5A^†^	Highcliff	YEP-5	Oral cavity	1	82	217,541	15	2.40	62.9	2,069	52	1
6A^†^	Highcliff	YEP-6	Oral cavity	1	75	241,723	16	2.45	62.9	2,132	52	1
19B	Midsection	YEP-19	Lesion	1	91	294,621	14	2.45	62.9	2,131	52	1
48B^¶^	Okia	YEP-48	Lesion	1	54	174,460	19	2.40	62.9	2,071	52	1
49B^¶^	Okia	YEP-49	Lesion	1	68	199,773	17	2.40	62.9	2,071	52	1
50A*	Okia	YEP-50	Oral cavity	1	88	241,730	16	2.40	62.9	2,070	52	1
51B*	Okia	YEP-51	Lesion	1	72	280,374	12	2.40	62.9	2,070	52	1
52A	Okia	YEP-52	Oral cavity	1	82	241,719	18	2.40	62.9	2,067	52	1
71B	Highcliff	YEP-71	Lesion	1	68	252,028	15	2.45	62.9	2,130	52	1
73A	Highcliff	YEP-73	Lesion	1	82	240,796	15	2.40	62.9	2,069	52	1
7B	Highcliff	YEP-7	Lesion	2	59	215,111	17	2.46	62.7	2,106	51	1
12B	Unknown	YEP-12	Unknown	2	89	289,014	16	2.46	62.7	2,107	51	1
20A	A1 section	YEP-20	Oral cavity	2	84	254,507	17	2.50	62.7	2,072	51	1
74A	A1 section	YEP-74	Lesion	2	99	437,349	11	2.46	62.7	2,105	51	1
11A	Highcliff	YEP-11	Lesion	2	63	135,658	24	2.40	62.5	2,075	52	1

a Strains isolated from the same nests are labeled with the same symbols.

b CDS, coding DNA sequence.

10.1128/mSystems.00320-21.1 FIG S1 A map of the Otago Peninsula showing the sampling sites. Download FIG S1, PDF file, 1.9 MB. Copyright © 2021 Saunderson et al. 2021 Saunderson et al. https://creativecommons.org/licenses/by/4.0/ This content is distributed under the terms of the Creative Commons Attribution 4.0 International license.

Small circular bacterial white-cream-color colonies appeared on the blood agar plates after 16 h of incubation at 37°C (Fig. S2). These isolates were Gram-positive coccobacilli, and a BLAST search of the nucleotide sequence of the 16S rRNA gene extracted from these genomes in GenBank (https://blast.ncbi.nlm.nih.gov/) revealed 98.7% similarity with a partial sequence (92% coverage) of 16S rRNA gene of Corynebacterium ciconiae strain BS13^T^. However, these strains could not be assigned to any known species by matrix-assisted laser desorption ionization–time of flight mass spectrometry (MALDI-TOF MS) analysis, indicating that they are potentially novel *Corynebacterium* species.

10.1128/mSystems.00320-21.2 FIG S2 Bacterial growth from a swab on a blood agar plate. Download FIG S2, PDF file, 2.3 MB. Copyright © 2021 Saunderson et al. 2021 Saunderson et al. https://creativecommons.org/licenses/by/4.0/ This content is distributed under the terms of the Creative Commons Attribution 4.0 International license.

### Phylogenomic diversity between *Corynebacterium* sp. isolates.

The size of genome assemblies of all 16 isolates from yellow-eyed penguins varied from 2.4 to 2.5 Mb with a GC content of 62.5 to 62.9 mol% and 54- to 99-fold average coverage (Table 1). The draft assemblies were annotated with 2,067 to 2,138 genes, 51 to 52 tRNAs, and 1 transfer-messenger RNA (tmRNA) gene (Table 1). Whole-genome sequences of these strains have been submitted to GenBank (accession numbers PQMG00000000 to PQMV00000000; Table S1).

10.1128/mSystems.00320-21.5 TABLE S1 Details of the *Corynebacterium* strains obtained from GenBank and sequenced in this study. Download Table S1, PDF file, 0.1 MB. Copyright © 2021 Saunderson et al. 2021 Saunderson et al. https://creativecommons.org/licenses/by/4.0/ This content is distributed under the terms of the Creative Commons Attribution 4.0 International license.

**FIG 2:**
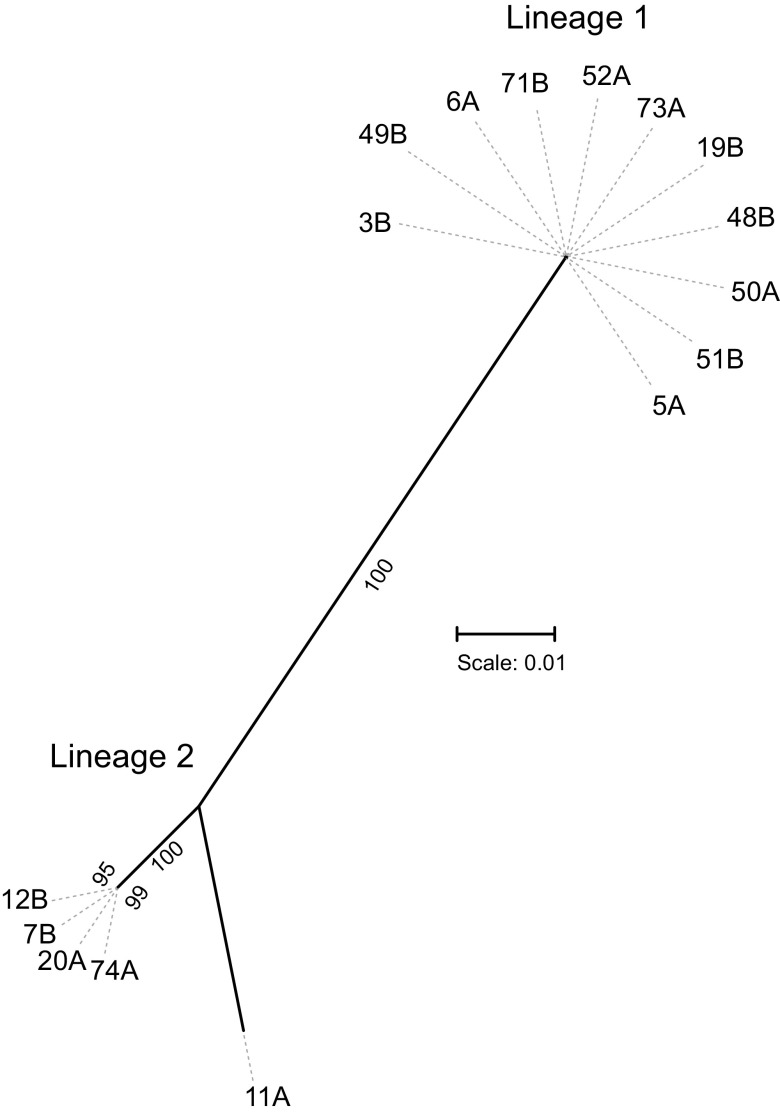
A maximum-likelihood tree from the core genome of *Corynebacterium* isolates from yellow-eyed penguins. The scale bar represents nucleotide substitutions per nucleotide site.

The pan-genome contains 2,272 genes, with 1,962 shared by all of the strains, and 310 genes belonged to the accessory genome. The phylogenetic tree from the core genome separated the strains into two distinct lineages. These isolates were collected from yellow-eyed penguin chicks from four nest sites (Table 1; Fig. S1). Lineage 1 encompassed 11 of the 16 isolates from three nest sites, whereas the remaining five isolates from two sites grouped in lineage 2 strains (Fig. 2; Table 1). Interestingly, all the isolates collected from yellow-eyed penguins at the Victory Beach (Okia Reserve) and most of the isolates from Highcliff clustered in lineage 1, whereas both the isolates from the A1 section were restricted to lineage 2. This indicates a potential association between the lineages and the sampling sites; however, more samples need to be analyzed to establish such an association.

Lineage 1 is a relatively homogenous in comparison to lineage 2, where one isolate, 11A, is clearly distinct from the other isolates (Fig. 2). A total of 51 genes were specific to lineage 1, 28 genes with predicted functions and 23 encoding hypothetical proteins (Table S2). Similarly, 61 genes were unique to lineage 2 with 32 encoding hypothetical proteins (Table S2). Some lineage-specific genes with known functions encode cellular enzymes; e.g., the gene encoding β-glucosidase A is only present in lineage 1 isolates (Table S2). This gene is involved in carbohydrate metabolism, particularly hydrolyzing short-chain oligosaccharides and cellobiose (11). Similarly, a gene encoding acetaldehyde dehydrogenase is specific to lineage 2 which is absent among lineage 1 strains (Table S2). Acetaldehyde dehydrogenase protects cells from cytotoxicity by oxidizing acetaldehyde to acetyl-CoA (12). Such variations potentially indicate minor variations in metabolic activities between the two lineages.

10.1128/mSystems.00320-21.6 TABLE S2 Pan-genome of *Corynebacterium* isolates from yellow-eyed penguins with predicted genomic islands and prophages. Prediction inconsistencies, where genes were predicted to be GI or bacteriophages in some strains but not in others, are highlighted in yellow and orange, respectively. Table S2, PDF file, 1.1 MB Copyright © 2021 Saunderson et al. 2021 Saunderson et al. https://creativecommons.org/licenses/by/4.0/ This content is distributed under the terms of the Creative Commons Attribution 4.0 International license.

### Genome plasticity.

Genomic islands (GIs) and prophages are often the sources of genome plasticity among pathogenic *Corynebacterium* species (13–16). A total of 15 GIs were identified among 16 *Corynebacterium* isolates from yellow-eyed penguins (Table S2). Five of the GIs (GI-1, GI-3, GI-6, GI-7, and GI-10) were conserved among all the strains. GI-1 encompasses genes involved in iron transport, including an anguibactin system regulator that plays an important role in virulence in vibrios (17, 18). The siderophore anguibactin system is located on a virulence plasmid in the fish pathogen Vibrio anguillarum (18). It is possible that these *Corynebacterium* strains have horizontally acquired this system from other marine pathogens. GI-1 also contains genes encoding polyketide synthase, PksR, and phthiocerol synthesis polyketide synthase type I PpsE (Table S2) that are involved in diverse metabolic activities, including biosynthesis of mycolic acids (19). Similarly, genes on GI-3 encoded proteins that are involved in various cellular activities, including transcriptional regulator ClgR, membrane chaperone PspA/IM30 family protein, energy-coupling factor transporter transmembrane protein EcfT, biotin transport ATP-binding protein BioM, biotin transporter BioY, a hypothetical protein, and recombinase A (Table S2). GI-6 included genes encoding enzymes for carbohydrate metabolism, whereas GI-7 and GI-10 carried Cas (CRISPR-associated) genes (Table S2).

Most of the genes on lineage 1-specific GI-2 and GI-8 encoded hypothetical proteins, whereas GI-4 carries the genes involved in DNA metabolism, including a type-III restriction modification system (Table S2). This locus is present between the genes encoding bifunctional protein PutA and a hypothetical protein (locus tags in strain 3B, hoi102_00282 and hoi102_00288, respectively) that are also conserved among lineage 2 isolates, except for the latter gene, which is missing in strain 11A. A small genomic island with four genes, GI-5, followed the gene encoding hypothetical protein in four lineage 2 strains, with two genes encoding ascorbate-specific phosphotransferase enzyme IIA component and ascorbate-specific permease IIC component UlaA, respectively, and the remaining two encoding hypothetical proteins (Table S2). Similarly, GI-13 carried some Cas genes that were specific to lineage 2 strains except for strain 11A.

A majority of genes on lineage 2-specific GI-11 and GI-14 encoded hypothetical proteins, whereas GI-12 contained several genes involved in oxidation of aromatic compounds, including the subunits of 3-phenylpropionate/cinnamic acid dioxygenase, ferredoxin-NAD(+) reductase subunit of benzene 1,2-dioxygenase, biphenyl dioxygenase subunit beta, cis-2,3-dihydrobiphenyl-2,3-diol dehydrogenase, and a long-chain fatty acid-CoA ligase, FadD13 (Table S2). GI-11 has been identified as a prophage (partial; Φ3) that has integrated between *attL* and *attR* sites in the genome.

GI-15 was only present in strain 11A, which included two genes encoding adenylate and guanylate cyclase catalytic domain proteins and two hypothetical proteins. GI-9, a large genomic island carrying a number of genes coding for phage-related proteins or hypothetical proteins was present in four lineage 1 isolates (3B, 6A, 19B, and 71B) and strain 11A of lineage 2. Indeed, a large part of this island has also been identified as an incomplete prophage, Φ2 (Table S2).

In addition, a 6.2- to 6.3-Kb conserved region was predicted as a potential incomplete prophage that carried the genes encoding sporulation initiation inhibitor protein (ParA family), chromosome-partitioning protein ParB, a hypothetical protein, *N*-acetylmuramoyl-l-alanine amidase LytC precursor, thioredoxin, thioredoxin reductase, and extracytoplasmic function (ECF) RNA polymerase sigma factor SigM (Φ1; Table S2). ParA and ParB proteins are involved in DNA separation and partitioning and are commonly present on bacterial chromosomes, plasmids, and bacteriophages (20–22). Thioredoxin and thioredoxin reductase are involved in a number of cellular activities, including response to oxidative stress (23). In bacteriophages, thioredoxin is important for the phage assembly (24). LytC is an autolysin responsible for hydrolysis of *N*-acetylmuramoyl-l-alanine linkage in peptidoglycan associated with virulence in S. pneumoniae (25). Overall, recombination has played a key role in introducing diversity among these corynebacterial strains, and the proteins encoded on these GIs are likely responsible for variations in cellular and metabolic activities both between and within each lineage.

### Virulence characteristics.

Surface pili are important for adhesion and invasion of the host cells (26–28). These pili are encoded by *spa* (sortase-mediated pilus assembly) gene clusters, and the number of pilus gene clusters varies between one and three among pathogenic corynebacteria (13–15, 28, 29). One pilus gene cluster has been observed among the genomes of *Corynebacterium* isolates from yellow-eyed penguins (*hoi102_00558* to *hoi102_00561* in strain 3B). The genes *hoi102_00558* and *hoi102_00560* encode 443-amino acid (aa)-long and 510-aa-long fimbrial subunits, respectively, and the *hoi102_00561* gene encodes a cell wall anchor domain-containing protein (891 aa). *hoi102_00559* is a class C sortase that is responsible for assembling the pilus. An additional class E sortase is present elsewhere in the genome (*hoi102_01169*), which is also conserved among all isolates.

The gene encoding diphtheria-like toxin is absent among these isolates; however, two genes encode phospholipase YtpA, and one gene encoding phospholipase D precursor may be responsible for virulence characteristics of these strains (30). Phospholipase D is a key virulence factor in Corynebacterium pseudotuberculosis and Corynebacterium ulcerans (29, 31, 32). However, the predicted phospholipase D precursor among penguin isolates (*hoi102_01010* in strain 3B) is relatively larger in size (577 aa) than those present in C. pseudotuberculosis and C. ulcerans (307 aa). A search in the Conserved Domain Database (33) confirmed that *hoi102_01010* belongs to the PhoD super family (activity, phosphodiesterase/alkaline phosphatase D; accession number cl26056). A BLAST search of the gene in the UniProt proteome database (https://www.uniprot.org/) showed 54% similarities with the phospholipase D genes of Corynebacterium geronticis, Corynebacterium pelargi, and Corynebacterium pseudopelargi. Phospholipase D is a member of the PhoD family, a diverse superfamily of enzymes that cleaves the phosphodiester bond linking the head group and diacyl glycerol moieties in phospholipids and is also involved in wide range of cellular processes (34–37).

A protein BLAST search of the virulence-associated genes from pathogenic corynebacteria identified two copies of cell wall-associated hydrolase, a gene encoding peptidoglycan endopeptidase RipA protein (*rpfI*), and genes encoding resuscitation-promoting factors, RpfA and RpfB, among all the isolates from yellow-eyed penguins (Table 2). These proteins are important for cell division and cell surface organization and likely play an important role in adhesion to the host cells and internalization of the pathogen (29, 38–42). In addition, genes encoding corynebacterial/nonribosomal peptide synthetase (linear gramicidin synthase subunit D) and subunits of acyl/propionyl-CoA carboxylase (DtsR1, DtsR2, and AccD3) are also identified in all yellow-eyed penguin isolates (Table 2). These genes were found to be upregulated during the macrophage infection by C. pseudotuberculosis (43). Moreover, *dtsR1*, *dtsR2*, and *accD3* genes are involved in fatty acid and mycolic acid biosynthesis, which contribute to the virulence characteristics (44, 45). The virulence genes also include subtilisin, a protease potentially involved in hypoxic response during the colonization of the respiratory tract (46).

**TABLE 2:** Corynebacterial virulence-associated genes identified among penguin isolates

Gene	Function	Loci in strain 3B^*a*^
*cwlH*	Cell wall-associated hydrolase	*hoi102_01530*; *hoi102_01529*
*rpfI*	Peptidoglycan endopeptidase (RipA)	*hoi102_00347*
*rpfA*	Resuscitation-promoting factor A	*hoi102_02179*
*rpfB*	Resuscitation-promoting factor B	*hoi102_01747*
*nrpS2*	Nonribosomal peptide synthetase	*hoi102_00519*
*dtsR1*	Acyl/propionyl-CoA carboxylase b-subunit	*hoi102_01834*
*dtsR2*	Acyl/propionyl-CoA carboxylase b-subunit	*hoi102_01833*
*accD3*	Acyl/propionyl-CoA carboxylase b-subunit	*hoi102_01113*
	Secreted protein/subtilisin E precursor	*hoi102_00965*

a The locus tags of only one penguin isolate are mentioned, but these genes are present among all 16 isolates across both lineages.

The virulence potential of isolates from yellow-eyed penguins was tested in a Galleria mellonella model using C. ulcerans NZRM 818 and C. pseudotuberculosis NZRM 3004 as positive controls and nonpathogenic *Corynebacterium* sp. strain NZRM 2522 and phosphate-buffered saline (PBS) as negative controls. All the larvae injected with C. ulcerans and C. pseudotuberculosis died within 48 h (>90% within 24 h; Table S3). In contrast, only one of the 30 caterpillars inoculated with strain NZRM 2522, test strains 3B and 6A, and two larvae inoculated with PBS died during the experiment. Similarly, no mortality was recorded for other isolates from penguins except for strains 11A and 71B, where 1/10 larvae and 3/10 larvae died, respectively (Table S3). However, only Bacillus thuringensis strains were isolated from these dead caterpillars. Therefore, *Corynebacterium* isolates from yellow-eyed penguins appears to be nonpathogenic in the caterpillar challenge model.

10.1128/mSystems.00320-21.7 TABLE S3 Results of the Galleria mellonella infection assay. Download Table S3, PDF file, 0.01 MB. Copyright © 2021 Saunderson et al. 2021 Saunderson et al. https://creativecommons.org/licenses/by/4.0/ This content is distributed under the terms of the Creative Commons Attribution 4.0 International license.

### Taxogenomic characteristics.

**FIG 3:**
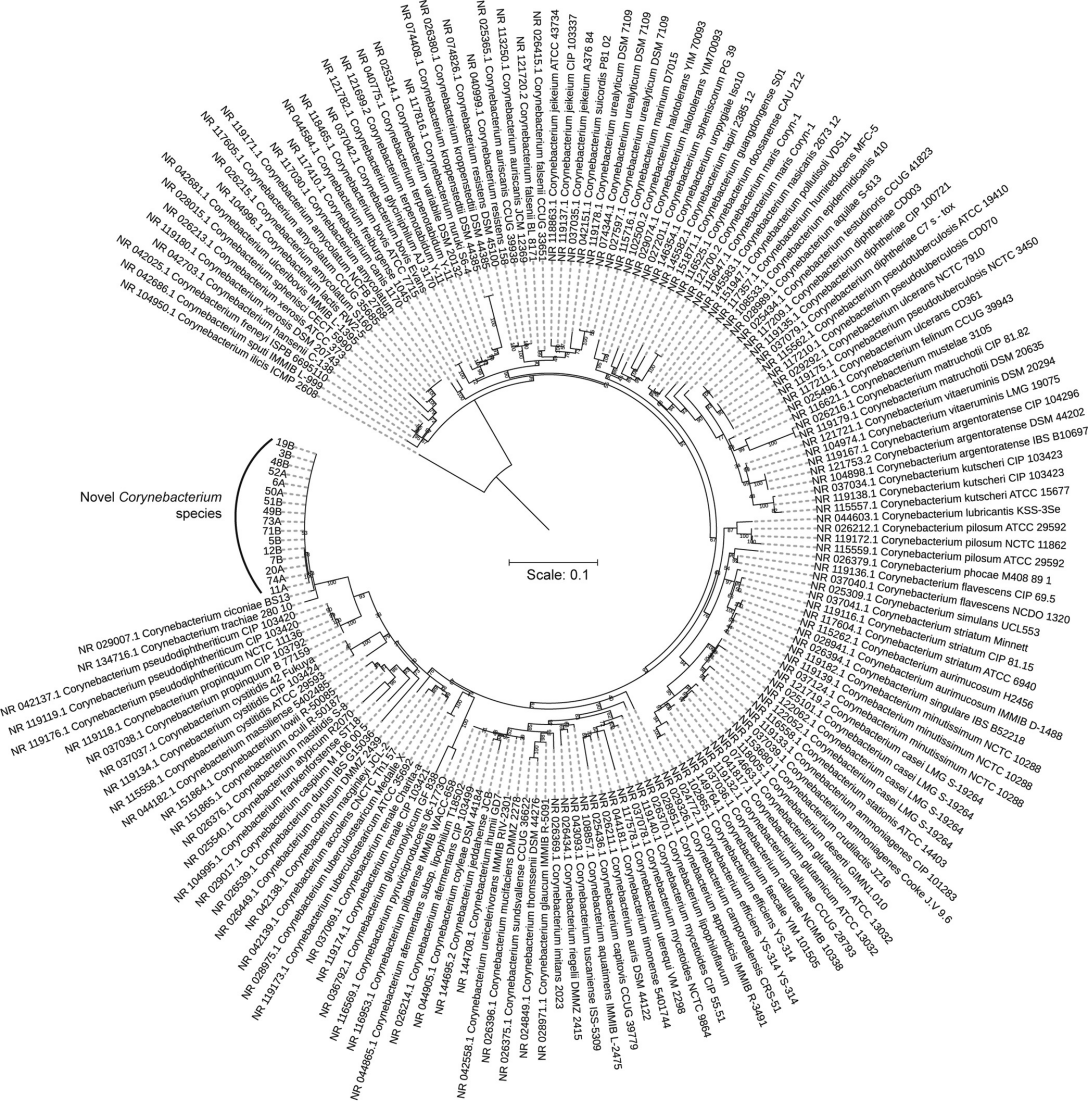
A maximum-likelihood tree from the nucleotide sequences of 16S rRNA genes of *Corynebacterium* strains obtained from GenBank. The scale bar represents nucleotide substitutions per nucleotide site.

**FIG 4:**
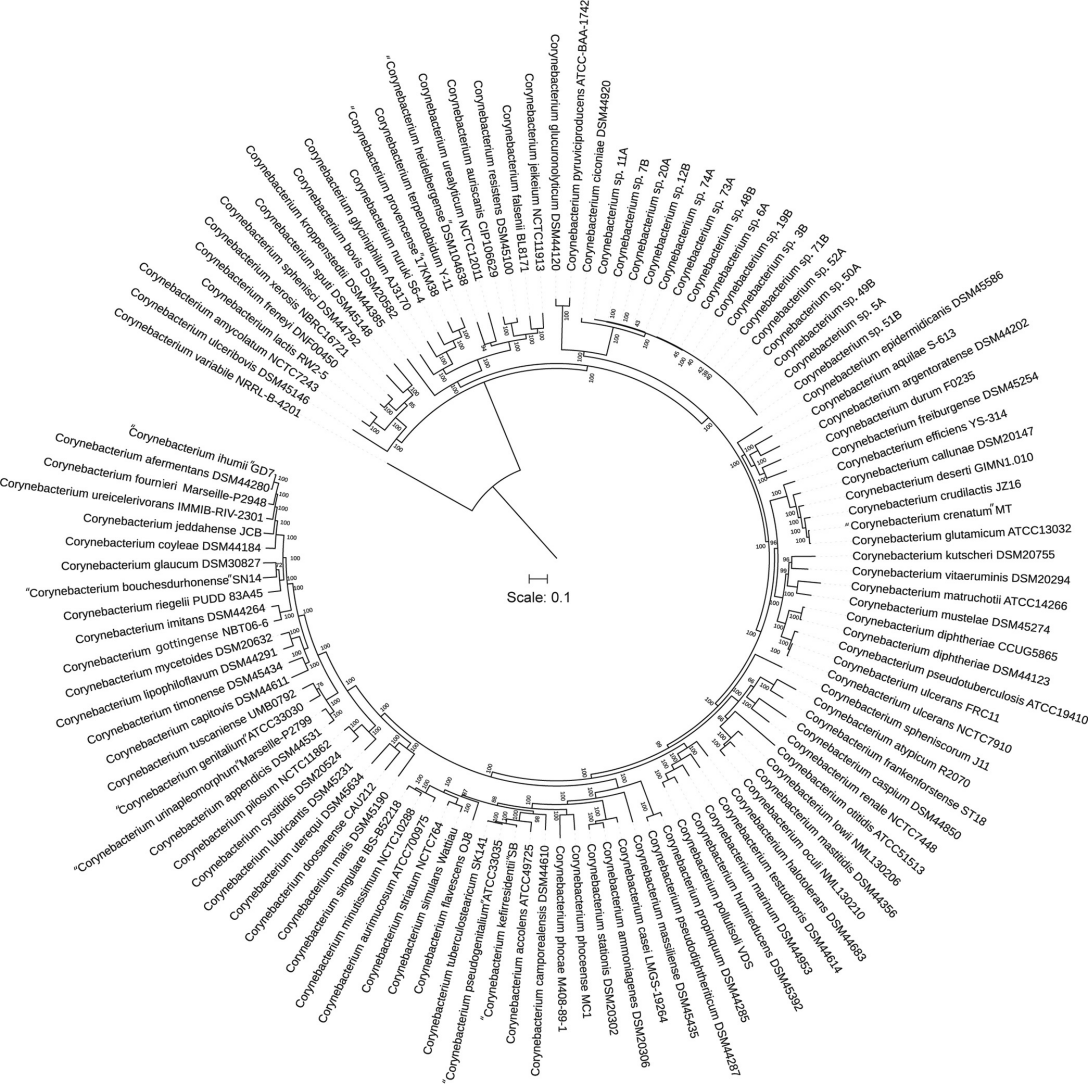
A maximum-likelihood tree from the alignment of 184 core proteins of 117 corynebacterial genomes (Table S1). The scale bar represents amino-acid substitutions per site. Some species designations in the genomic data obtained from GenBank are pending validation. These species designations are mentioned in quotation marks.

**FIG 5:**
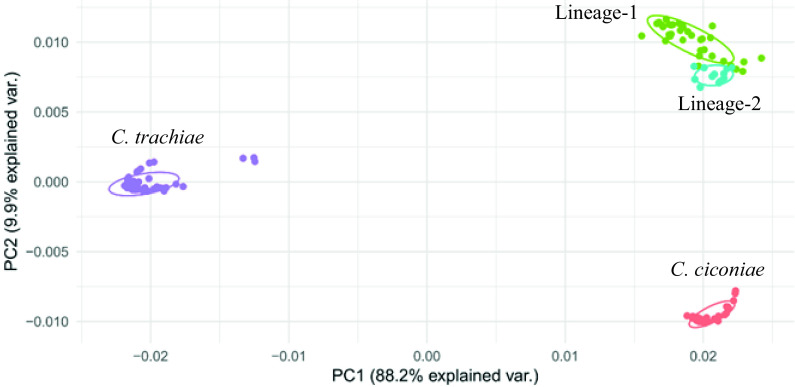
Principal-component analysis of MALDI-TOF peak intensity profiles.

A maximum-likelihood (ML) tree constructed from corynebacterial 16S rRNA sequences obtained from GenBank distinctly separated isolates from penguins that were closely related to *C. ciconiae* strain BS13^T^ (DSM 44920^T^; Fig. 3). Similarly, these novel isolates formed a distinct clade close to *C. ciconiae* DSM 44920^T^ in the ML tree constructed from 184 core genes, including representative *Corynebacterium* strains from GenBank (Fig. 4; Table S1). Principal-component analysis of MALDI-TOF spectra clustered these isolates distinctly from *C. ciconiae* and Corynebacterium trachiae (Fig. 5). Representative averaged MALDI-TOF spectra showed highly discriminant peaks between *C. ciconiae*, *C. trachiae*, and the penguin isolates (Fig. S3). Peaks at *m/z* values of 617, 6,484, and 11,224 showed positive associations with lineage 1 and lineage 2 strains with only subtle differences that could reliably distinguish them. A phenotypic characterization of representative strains from both the lineages, 3B and 7B, also revealed similar biochemical properties except for minor variations (Table 3). Strain 7B was able to produce *N*-acetyl-β-glucosaminidase and was able to utilize d-mannose, d-maltose, and malic acid that were not detected in strain 3B (Table 3). Strain 7B was also weakly positive for adipic acid fermentation.

**TABLE 3:** Biochemical properties of representative strains using API ZYM and API 20NE systems^*a*^

Biochemical test	Isolate 3B	Isolate 7B
API ZYM		
Esterase (C4)	−	−
Esterase lipase (C8)	−	−
Lipase (C14)	+	+
Leucine aminopeptidase	+	+
Valine aminopeptidase	−	−
Cystine aminopeptidase	−	−
Trypsin	−	−
Α-chymotrypsin	−	−
Acid phosphatase	−	−
Naphthol-AS-BI-phosphohydrolase	−	−
α-Galactosidase	+	+
β-Galactosidase	−	−
β-Glucuronidase	−	−
α-Glucosidase	−	−
β-Glucosidase	−	−
*N*-acetyl-β-glucosaminidase	−	+
α-Mannosidase	−	−
α-fucosidase		−
API 20NE		
Potassium nitrate	−	−
l-Tryptophane	−	−
d-Glucose	−	−
l-Arginine	−	−
Urea	−	−
Esculin ferric citrate	−	−
Gelatin (bovine origin)	−	−
4-Nitrophenyl-β-d-galactopyranoside	−	−
d-Glucose	+	+
l-Arabinose	−	−
d-Mannose	−	+
d-Mannitol	−	−
*N*-acetyl-glucosamine	−	−
d-Maltose	−	+
Potassium gluconate	−	−
Capric acid	−	−
Adipic acid	−	W
Malic acid	−	+
Trisodium citrate	−	−
Phenylacetic acid	−	−

a +, positive reaction; −, negative reaction; W, weak positive.

10.1128/mSystems.00320-21.3 FIG S3 Average MALDI-TOF spectra for members of *Corynebacterium* spp. Highlighted peaks are those among the top 20 most discriminant peaks that showed positive associations with the indicated species. Download FIG S3, PDF file, 0.7 MB. Copyright © 2021 Saunderson et al. 2021 Saunderson et al. https://creativecommons.org/licenses/by/4.0/ This content is distributed under the terms of the Creative Commons Attribution 4.0 International license.

Mean fragmented BLAST scores (FBS values) between the penguin isolates and *C. ciconiae* were 30.5 ± 0.9, which is significantly higher than the values between the penguin isolates and other corynebacterial genomes (1.9 ± 0.7; Data Set S1A). Average BLAST-based nucleotide identities (ANIb values) between the penguin isolates and *C. ciconiae* strains were 82.6 ± 0.1, again higher than those observed between the novel isolates and other corynebacterial strains (69.8 ± 1.1; Data Set S1B). Digital DNA-DNA hybridization (dDDH) values between the genomic sequences also suggest isolates from yellow-eyed penguins to be a novel species (Data Set S1C).

10.1128/mSystems.00320-21.9 DATA SET S1 **(**A) Pairwise fragmented BLAST scores between the *Corynebacterium* genomes. (B) Pairwise ANIb values between the *Corynebacterium* genomes. (C) Digital DDH values between *Corynebacterium* strain 3B and other *Corynebacterium* genomes following the recommended Formula-2. (D) Pairwise digital-DDH between novel *Corynebacterium* strains. (E) Pairwise AAI between novel *Corynebacterium* strains. Download Data Set S1, XLSX file, 0.3 MB. Copyright © 2021 Saunderson et al. 2021 Saunderson et al. https://creativecommons.org/licenses/by/4.0/ This content is distributed under the terms of the Creative Commons Attribution 4.0 International license.

**FIG 6:**
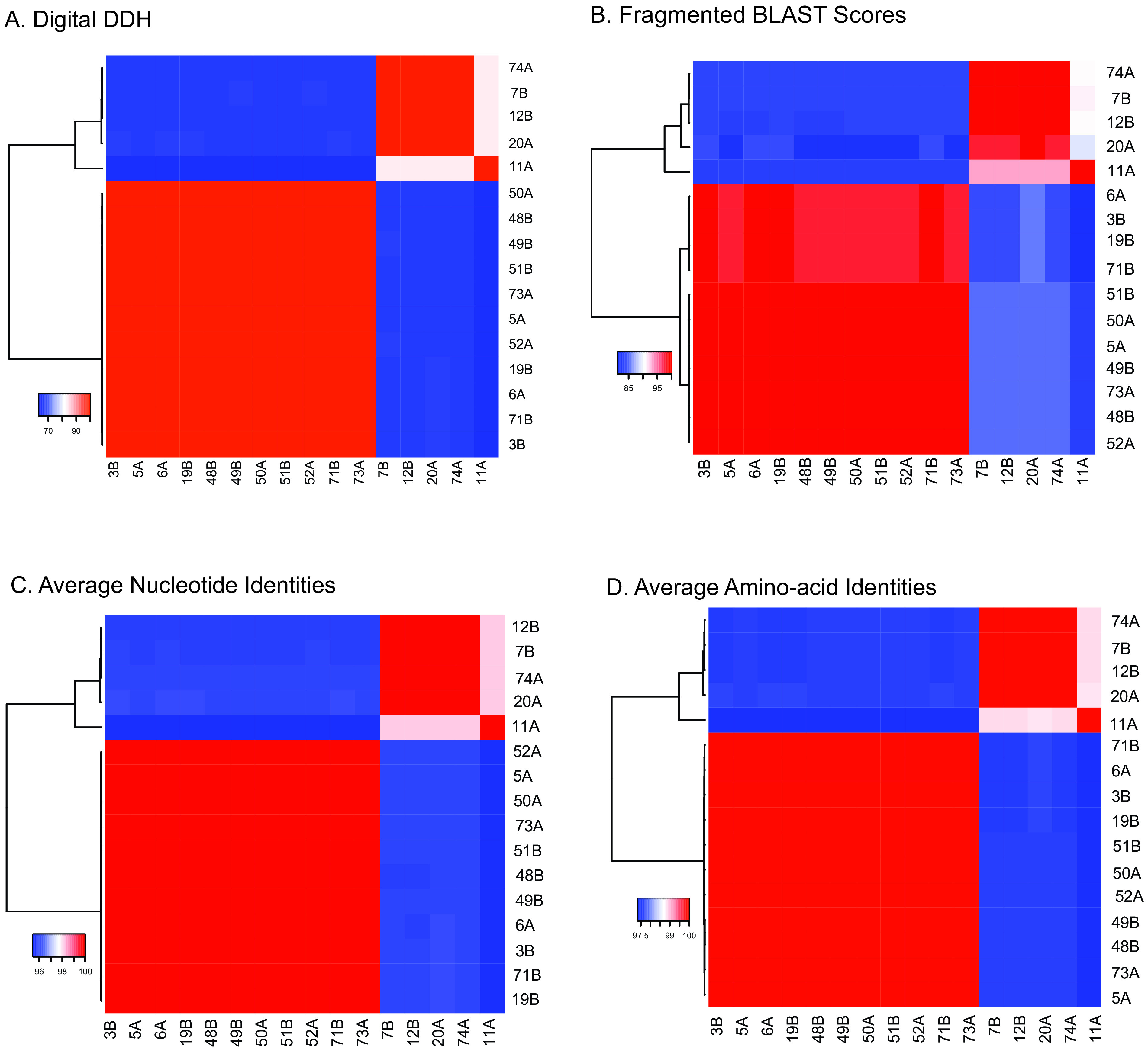
Heat maps from the pairwise genomic matrices for *Corynebacterium* isolates from yellow-eyed penguins. (A) fragmented BLAST scores; (B) digital DNA-DNA hybridization values; (C) genomic average nucleotide identities; (D) average amino-acid identities.

The dDDH values between strains within lineage 1 and lineage 2 were >99.7% and 83.3%, respectively. However, the pairwise dDDH values between individuals of the two lineages were slightly lower than the suggested cutoff value of 70% and varied between 63.2 and 66.7% (Fig. 6A; Data Set S1D), indicating that these lineages may represent two different species. However, average FBS and ANIb values between the two lineages are 83.6 ± 1.2 (81.2 to 85.5%; Fig. 6B; Data Set S1A) and 95.8 ± 0.2 (95.5 to 96.0%; Fig. 6C; Data Set S1B), respectively. Average amino acid identities (AAI) between the two lineages were also above the species threshold and varied between 97.4 and 97.7% (97.5 ± 0.8; Fig. 6D; Data Set S1E). These FBS, ANIb, and AAI values are consistent with lineage 1 and lineage 2 strains belonging to the same species (47, 48).

### PCR-based diagnostic assay.

Two genes specific to lineage 1 encoding a hypothetical protein and α/β hydrolase family protein, respectively, and two genes specific to lineage 2, both encoding hypothetical proteins (Table S4), did not show significant similarities with other *Corynebacterium* genomes and were selected for the diagnostics assay. These loci were amplified in a multiplex PCR in all isolates from yellow-eyed penguins, including two strains of each from C. diphtheriae and C. ulcerans. All 11 lineage 1 strains produced two amplicons of 219 bp and 274 bp, while all lineage 2 strains produced amplicons of 425 bp and 486 bp (Fig. S4). None of the four loci were amplified among C. diphtheriae or C. ulcerans strains.

10.1128/mSystems.00320-21.4 FIG S4 Diagnostic multiplex PCR for novel *Corynebacterium* strains from yellow-eyed penguins. (A) Two loci specific to lineage 1 strains; (B) two loci specific to lineage 2 strains along with two C. diphtheriae strains (NCTC 05011 and CCUG 5865), two C. ulcerans strains (2590 and BRAD 6249), and a no-template control (NTC). L represents a 50-bp hyperladder (Bioline). Download FIG S4, PDF file, 0.8 MB. Copyright © 2021 Saunderson et al. 2021 Saunderson et al. https://creativecommons.org/licenses/by/4.0/ This content is distributed under the terms of the Creative Commons Attribution 4.0 International license.

10.1128/mSystems.00320-21.8 TABLE S4 Primer details used for amplifying lineage-specific loci. Download Table S4, PDF file, 0.1 MB. Copyright © 2021 Saunderson et al. 2021 Saunderson et al. https://creativecommons.org/licenses/by/4.0/ This content is distributed under the terms of the Creative Commons Attribution 4.0 International license.

## DISCUSSION

### *Corynebacterium* sp. isolates from yellow-eyed penguins belong to a novel species.

*Corynebacterium* is a diverse genus that encompasses species of industrial, veterinary, or medical importance (9). In this study, we identified a novel *Corynebacterium* species isolated from yellow-eyed penguins in New Zealand. Phylogenetic analyses of rRNA gene sequences and the core genome revealed these strains to be quite distinct from other corynebacteria, with *C. ciconiae* being the closest relative (Fig. 3 and 4). MALDI-TOF spectral analyses distinctly separated these strains from the type strains of *C. ciconiae* and *C. trachiae* (Fig. 5 and Fig. S3), and genome-based taxonomic analyses, FBS (48), and ANIb values (47–50), also indicated these *Corynebacterium* strains from yellow-eyed penguins to be a novel species (Data Set S1A-C).

Phylogenomic analyses of these strains revealed two lineages within this species (Fig. 2), which is consistent with the ANIb and AAI values between individuals of both the lineages (species threshold, ≥95%; Fig. 6C and D; Data Set S1B and S1E). However, dDDH values were slightly below the threshold of ≥70% (Fig. 5A; Data Set S1D) (51, 52). The taxogenomic thresholds should be considered with flexibility in combination with other phylogenetic and biological properties to define species (53). The gene content is highly conserved between the two lineages (Table S2) with only minor differences in the biochemical properties (Table 3) and in MALDI-TOF spectra (Fig. 5; Fig. S3). The strains of both the lineages are associated with diphtheritic stomatitis with similar clinical manifestations and possess the same set of virulence-associated genes (Table 3). Therefore, these lineages could potentially be assigned to a single species. A manuscript is currently being prepared with a detailed description of this novel *Corynebacterium* species, including a proposal to name lineage 1 and lineage 2 as subspecies. Two type strains, 3B^T^ and 7B^T^, representatives of lineages 1 and 2, have been deposited at the DSMZ German collection of microorganisms and cell cultures (strains DSM 111184 and DSM 111183) and at the New Zealand Reference Culture Collection (strains NZRM 4755 and NZRM 4756), respectively.

Both the lineages can easily be identified in a simple multiplex PCR (Fig. S4). A BLAST search of the entire NCBI nucleotide database did not return any significant similarities of these loci with any other genomes, confirming their suitability for diagnostics of these lineages. This will also eliminate the need to run time- and labor-intensive biochemical tests for identifying these strains in the laboratory, which may expedite the treatment of the affected yellow-eyed penguin chicks.

### Opportunistic pathogens to young penguin chicks.

Lineage 1 is more prevalent among the nests than lineage 2, the latter being more common to nests at the site A1 section (Table 1; Fig. S1). Also, the pairs of twin chicks (YEP-5 and YEP-6; YEP-48 and YEP-49; YEP-50 and YEP-51) harbored identical lineages, which suggests each nest is infected by a single prevalent lineage. Both the lineages are associated with diphtheritic stomatitis, which has a high infection and mortality rate among mainland New Zealand populations of neonate yellow-eyed penguin chicks (7, 8). Adhesion and invasion of the host cells are important for infection and are facilitated by surface pili (54). These pili are generally composed of major subunit (shaft pilin), minor subunit (base pilin), and tip pilin that are assembled by sortases (54, 55). Hoi102_00559 showed 49% sequence identity (89% query coverage) with the *srtC* gene, and Hoi102_00560 showed 31% identity (81% query coverage) with SpaD, a major pilin subunit, of C. diphtheriae. Hoi102_00558 is similar to the minor pilin subunit, SpaI (27% query coverage and 31% identity). The Hoi102_00561 protein is an LPXTG cell wall anchor domain-containing protein also identified as von Willebrand factor type A domain protein. The von Willebrand factor type A domain is also present in the tip protein of SpaDEF pili, SpaF; therefore, isolates from yellow-eyed penguins possess a *spaDEF*-type pilus gene cluster which is reported to interact with laryngeal and lung epithelial cells (54, 55).

Several corynebacterial virulence genes are present among these strains (Table 2), including two genes encoding phospholipase YtpA, which is reported to be involved in the biosynthesis of bacilysocin, which competitively inhibits growth of other microorganisms (56). The gene (*hoi102_01010*) encoding phospholipase D may be the main virulence factor among these strains (Table S2), which is an exotoxin involved in the persistence and spread of the pathogen within the host cells through hydrolysis of sphingomyelin in endothelial membranes, which increases the vascular permeability (29, 57). However, mortality in Galleria mellonella larvae on inoculation of penguin strains was similar to that caused by nonpathogenic corynebacteria or PBS (Table S3). A protein BLAST search revealed that all corynebacterial virulence-associated genes reported among the penguin isolates are also present in the *C. ciconiae* genome (data not shown). *C. ciconiae* was isolated from the trachea of wild black storks, Ciconia nigra (58), and is not reported to be causing any fatal infections. Therefore, the novel strains from yellow-eyed penguins are likely opportunistic pathogens causing high mortality among young chicks, potentially due to weak immune systems.

### Diphtheritic stomatitis in chicks and a potential prevention strategy.

Yellow-eyed penguins are an endangered species that are threatened with extinction if current population trends persist (2). Annual outbreaks of diphtheritic stomatitis in chicks results in widespread reproductive failure throughout their mainland distribution. An intensive treatment with a combination of amoxicillin-clavulanic acid or enrofloxacin does not always result in full recovery of the chicks, a problem compounded with the potential physical stress during multiple handling events and crushing injuries from adult penguins. An effective vaccine to protect yellow-eyed penguins from diphtheritic stomatitis that will also limit the direct handling of young chicks might help mitigate this population decline.

A phospholipase D vaccine is an option for disease prevention. Vaccines targeting phospholipase have been tested against Acinetobacter baumannii, Mycobacterium abscessus, and C. pseudotuberculosis (59–61). Survival rates were improved in mice, with reduced pulmonary bacterial load and cytokine levels in the broncho-alveolar lavage fluid and the serum when they were immunized with histidine fusion proteins, small protein A (His-SmpA), and phospolipase D (His-PLD) following the challenge with A. baumannii (61). Similarly, immunization with plasmid DNA encoding the M. abscessus phospholipase C (MA-PLC) or with purified MA-PLC protein rapidly cleared the infection in the mouse model (60). Formalin-inactivated phospolipase D-rich culture supernatants offered 95% protection among sheep challenged with C. pseudotuberculosis (59). Therefore, inactivated phospholipase D enzyme from these novel strains could potentially be used to immunize yellow-eyed penguins. Due to the early age at which yellow-eyed penguin chicks are affected by diphtheritic stomatitis, a passive immunization strategy would be pertinent, that is the immunization of female adults for maternal transfer of protective IgY into chicks via their egg yolk. This strategy has shown promise, with maternal IgY providing protective effects to young broiler chickens against Clostridium perfringens*-*induced necrotic enteritis (62).

In summary, *Corynebacterium* isolates associated with significant mortality among young chicks of yellow-eyed penguins belong to a novel species. All these strains are equipped with genes encoding the subunits and assembly of SpaDEF type surface pili that are important for adhesion and invasion of the host cells and produce phospholipase D, which is a well-characterized virulence factor. These strains are nonpathogenic to wax moth caterpillars and appear to be opportunistic pathogens for young penguin chicks, with high mortality rates.

## MATERIALS AND METHODS

### Bacterial isolates and microbiological characterization.

In November 2014, swabs were collected from the oral cavities of 2- to 14-day-old yellow-eyed penguin chicks from nests from four breeding areas on the Otago Peninsula (Table 1; Fig. 1; Fig. S1). Swabs were transported using Transystem Amies transport medium with charcoal (Copan, Brescia, Italy) and were stored at 6 to 15°C for up to 24 h after field collection. They were cultured on Columbia horse blood agar plates (Fort Richard, Auckland, New Zealand) at 37°C for 16 to 20 h. Isolates were characterized using Gram staining and MALDI-TOF mass spectrometry as previously described (63). Phenotypic characteristics of two representative isolates, 3B and 7B, were determined using API ZYM and API 20 NE kits following the manufacturer’s instructions (bioMérieux, France).

### Genome sequencing and analyses.

First, 5 ml brain heart infusion broth (Oxoid, UK) was inoculated with a single colony and was incubated overnight at 37°C for 16 h in a shaking incubator. Genomic DNA was extracted from 2 ml of this culture using the UltraClean microbial DNA isolation kit (MoBio, USA). The genomes were sequenced on a MiSeq instrument (Illumina, Inc., USA) and the paired-end reads were assembled using SPAdes 3.9.0 (64). Genome sequences were annotated using Prokka 1.11 (65) and were compared using Roary with an identity threshold of 70% (66, 67). A maximum-likelihood (ML) tree was constructed from the core genomic sequence alignment using IQ-TREE (68) with the best-fit substitution model GTR+I, 100,000 ultrafast bootstraps, and 100,000 approximate likelihood ratio test and Shimodaira-Hasegawa (SH-aLRT) tests. IslandViewer 4 was used to predict genomic islands (GIs) in the genomes using Corynebacterium epidermidicanis strain DSM 45586 as the reference (69). Any prediction inconsistencies, i.e., regions or parts of any region predicted as a GI in some strains but not in others, were not considered unless they were predicted in ≥50% strains. In the latter case, they were recorded as GIs. Potential prophage sequences were identified using PHASTER (70, 71). Any inconsistencies in the predicted prophage-associated regions were also resolved using the approach used for GIs.

The protein sequences of known virulence genes from pathogenic Corynebacterium diphtheriae, Corynebacterium pseudotuberculosis, and Corynebacterium ulcerans (13, 14, 29, 42) were searched within the data set using BLAST (72). BLAST hits with ≥40% query coverage and sequence identities with an E value of <10^−5^ were considered positive.

### Characterization of virulence properties in Galleria mellonella.

Thirty Galleria mellonella larvae (Biosuppliers, Auckland, New Zealand) were each inoculated with two randomly selected penguin isolates (3B and 6A), C. ulcerans NZRM 818 (positive control), PBS (negative control), and nonpathogenic *Corynebacterium* sp. strain NZRM 2522 (negative control) as previously described (73). A smaller number, 10 larvae each, were inoculated for 11 additional strains from penguins and C. pseudotuberculosis NZRM 3004 to test the robustness of the results. Larvae were incubated at 37°C and assessed daily for up to 5 days. Hemolymph of dead larvae was cultured on Columbia horse blood agar.

### Taxogenomic analyses.

16S rRNA gene sequences were extracted from assembled genomes using the RNAmmer 1.2 server (74). Nucleotide sequences of 16S rRNA genes of all known *Corynebacterium* strains were obtained from GenBank (accessed on 10 May 2018). The sequences were aligned using MUSCLE (75), and the sites with the missing data were removed using MEGA X (76). A maximum-likelihood (ML) tree was calculated from the resulting alignment using a TPM3u+I+G4 substitution model and 100,000 ultrafast bootstraps and SH-aLRT tests using IQ-TREE (68).

Whole-genome sequences of *Corynebacterium* strains representing 99 species were obtained from GenBank (Table S1; accessed on 10 May 2018). All corynebacterial genome sequences were annotated using Prokka 1.11 (65) and were compared using BGPA 1.3 with the default settings (77). An ML tree was constructed from the concatenated sequence alignment of 184 core proteins after stripping the sites with missing data following the LG+F+I+G4 substitution model and 100,000 ultrafast bootstraps and SH-aLRT tests using IQ-TREE (68). The phylogenetic trees were visualized using iTOL (78) and were rerooted on the longest branch separating Corynebacterium ilicis ICMP 2608 for the 16S rRNA gene phylogeny and Corynebacterium variabile NRRL-B 4201 for the core genome phylogeny.

Pairwise FBS values were calculated using Gegenees (79) with a fragment size of 500 bp, and pairwise ANIb was calculated using JSpecies (80). dDDH values were calculated between one representative strain, 3B, and other *Corynebacterium* genomes using GGDC 2.1 (51, 52). Pairwise dDDH values were also calculated among the genomes of penguin isolates using the Genome-to-Genome Distance Calculator (GGDC) 2.1, and pairwise average amino acid identity (AAI) was calculated using the genome-based distance matrix calculator (81). Heat maps from the pairwise genomic matrices were created using Heatmapper (82).

### MALDI-TOF spectral analysis.

MALDI-TOF protein spectra were obtained in triplicate for all isolates using a previously described protein purification method (83) on a MALDI Biotyper Microflex LT/SH device using the MALDI Biotyper 8468 library (Bruker, USA). Principal-component analyses and the identification of discriminant peaks between species were performed using the MALDIquant (84) and sda (85) R packages. Control strains of *C. trachiae* and *C. ciconiae*, species that showed the closest genetic relationship to penguin isolates, were used for reference spectra.

### Primer designing and PCR amplification.

The pan-genomic nucleotide sequences were BLAST-searched (72) in the entire *Corynebacterium* genomic data set to identify genes that are unique to the strains isolated from yellow-eyed penguins. Primers were designed using the Web-based version of Primer3 (86) for two genes specific to each lineage (Table S3). The genes were amplified in a 25 μl multiplex PCR containing 200 μM deoxynucleoside triphosphate (dNTP), 5 μl of 5× Q5 reaction buffer, 1 unit of Q5 high-fidelity *Taq* polymerase (New England Biolabs, USA), 10 pmol of each primer, and ∼50 ng of the template DNA. Thermal cycling conditions include an initial hold at 94°C for 5 min, 30 cycles of 94°C for 30 s, 55°C for 45 s, and 72°C 1 min, followed by final extension at 72°C for 10 min. The amplicons were separated by electrophoresis on 1.5% agarose gel.

### Data availability.

The whole-genome shotgun project has been deposited at DDBJ/ENA/GenBank under the accession numbers PQMG00000000 to PQMV00000000 (BioProject PRJNA393261).
