# Supplementary figures and images for: Phylogenomic Characterization of a Novel Corynebacterium Species Associated with Fatal Diphtheritic Stomatitis in Endangered Yellow-Eyed Penguins
Source: mSystems. 2021 Jun 8;6(3):e00320-21. doi: 10.1128/mSystems.00320-21 (PMC8269222; doi:10.1128/mSystems.00320-21)

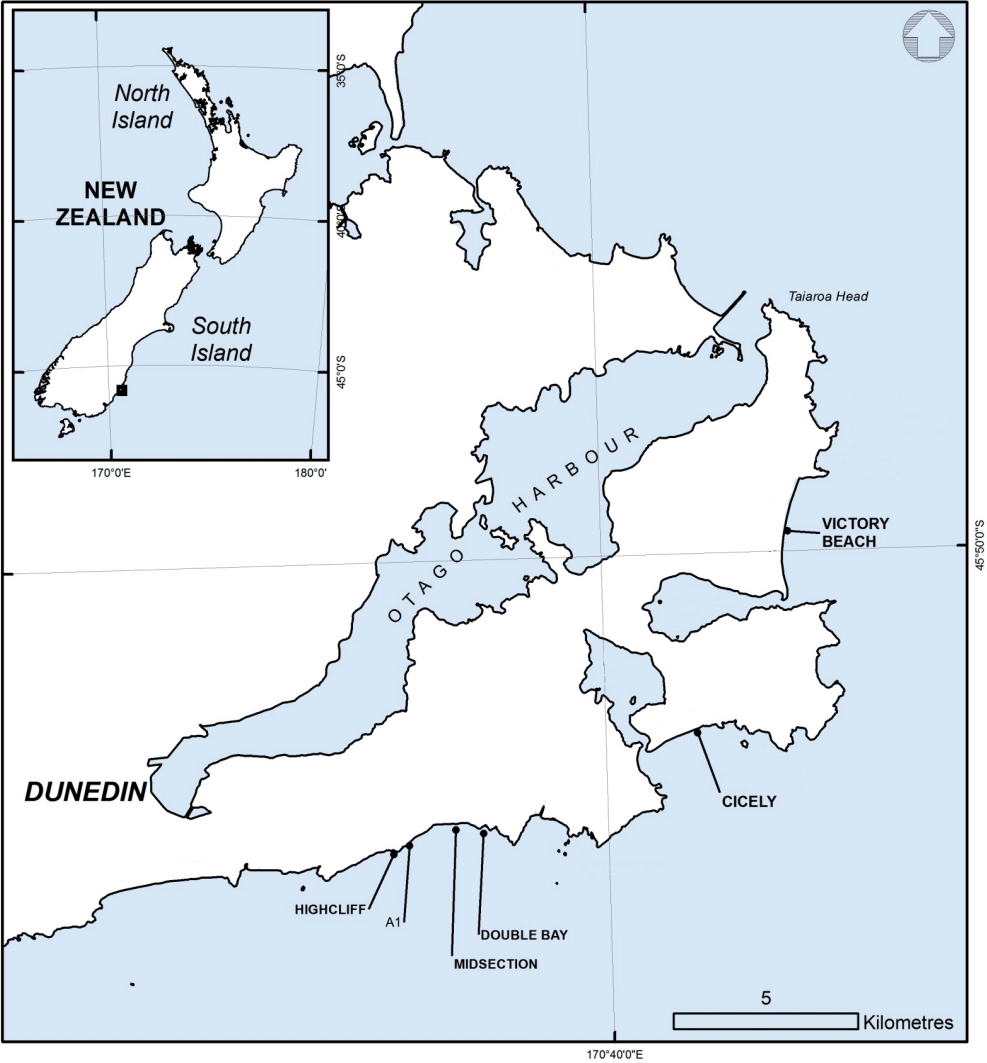

Supplement: FIG S1 [file msystems.00320-21-sf001.pdf]

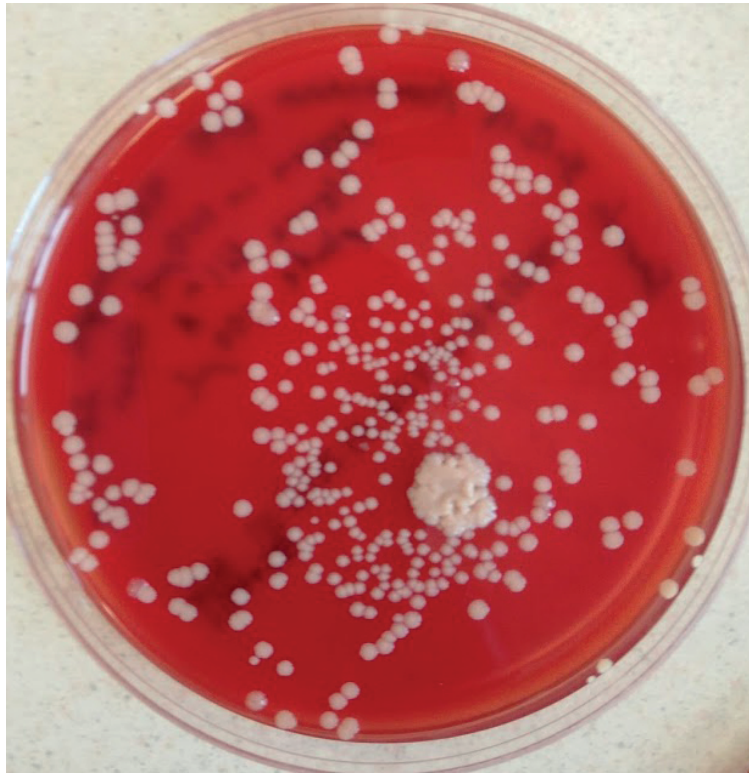

Supplement: FIG S2 [file msystems.00320-21-sf002.pdf]

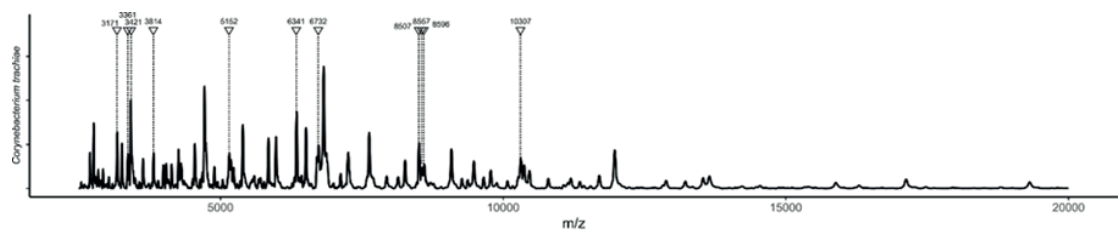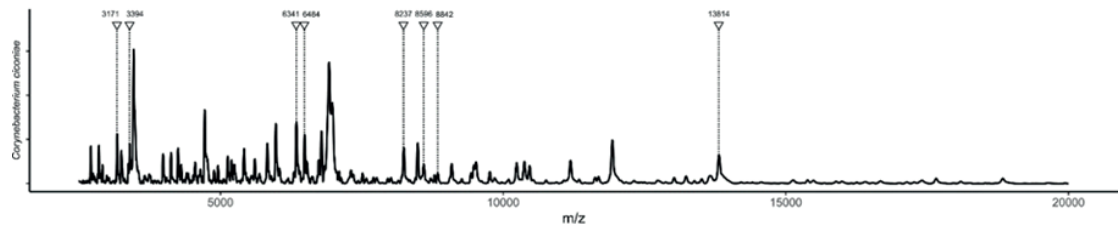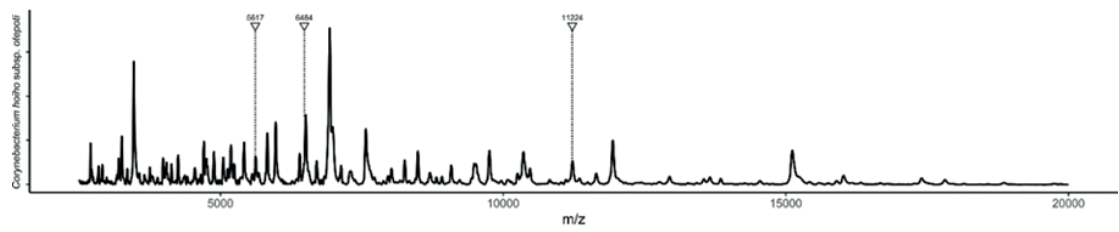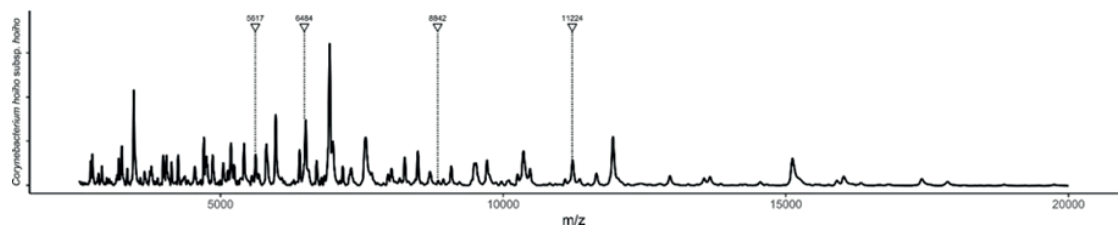

Supplement: FIG S3 [file msystems.00320-21-sf003.pdf]

A.

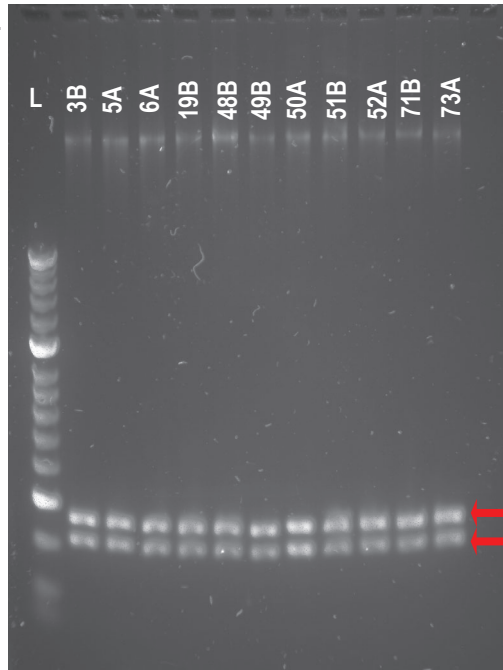

B.

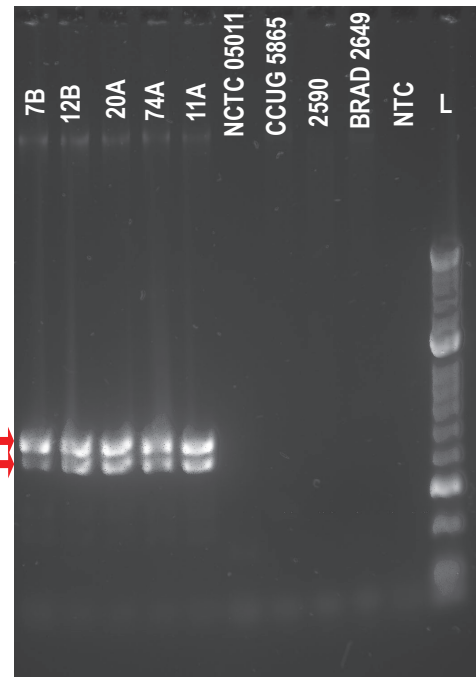

Supplement: FIG S4 [file msystems.00320-21-sf004.pdf]
