# Supplementary material for: Phylogenomic Characterization of a Novel Corynebacterium Species Associated with Fatal Diphtheritic Stomatitis in Endangered Yellow-Eyed Penguins
Source: mSystems. 2021 Jun 8;6(3):e00320-21. doi: 10.1128/mSystems.00320-21 (PMC8269222; doi:10.1128/mSystems.00320-21)
Supplement: TABLE S1 [file msystems.00320-21-st001.pdf]

S No	#Organism Name	Strain	Type	Size(Mb)	GC%	#Contigs	Accession
1	Corynebacterium accolens	ATCC 49725	y	2.44	59.5	38	ACGD01
2	Corynebacterium afermentans	DSM 44280	y	2.33	64.9	45	FTMH01
3	Corynebacterium ammoniagenes	DSM 20306	y	2.79	55.5	1	NZ_CP009244.1
4	Corynebacterium amycolatum	NCTC7243	n	2.64	58.8	6	UFXE01
5	Corynebacterium appendicis	DSM 44531	y	2.25	64.3	32	FTOF01
6	Corynebacterium aquilae	S-613	y	2.93	60.9	1	NZ_CP009245.1
7	Corynebacterium argentoratense	DSM 44202	y	2.03	58.9	1	NC_022198.1
8	Corynebacterium atypicum	R2070	y	2.36	65.4	2	NZ_CP008944.1
9	Corynebacterium aurimucosum	DSM 44827	n	2.82	60.5	2	NC_012590.1
10	Corynebacterium auriscanis	CIP 106629	y	2.57	58.5	33	JRVJ01
11	"Corynebacterium bouchesdurhonense"	SN14	c	2.26	68.0	18	FJVG01
12	Corynebacterium bovis	DSM 20582	y	2.52	72.6	503	AENJ01
13	Corynebacterium callunae	DSM 20147	y	2.93	52.5	3	NC_020506.1
14	Corynebacterium camporealensis	DSM 44610	y	2.45	59.4	1	NZ_CP011311.1
15	Corynebacterium capitovis	DSM 44611	y	1.96	64.5	5	AQUV01
16	Corynebacterium casei	LMG S-19264	y	3.13	55.7	3	NZ_CP004350.1
17	Corynebacterium caspium	DSM 44850	y	1.84	49.7	8	ARBM01
18	Corynebacterium ciconiae	DSM 44920	y	2.55	62.1	22	AQUW01
19	Corynebacterium coyleae	DSM 44184	y	2.57	61.3	2	FNRU01
20	"Corynebacterium crenatum"	MT	c	3.35	53.9	48	AQPS01
21	Corynebacterium crudilactis	JZ16	y	3.22	51.8	3	NZ_CP015622.1
22	Corynebacterium cystitidis	DSM 20524	y	2.95	57.0	40	FOGQ01
23	Corynebacterium deserti	GIMN1.010	y	3.03	55.3	3	NZ_CP009220.1
24	Corynebacterium diphtheriae	DSM 44123	y	2.37	53.5	28	LJXR01
25	Corynebacterium diphtheriae	CCUG 5865	y	2.60	53.6	132	LTAR01
26	Corynebacterium doosanense	CAU 212	y	2.70	66.7	2	NZ_CP006764.1
27	Corynebacterium durum	F0235	n	2.81	56.8	20	AMEM01
28	Corynebacterium efficiens	YS-314	y	3.22	62.9	3	NC_004369.1
29	Corynebacterium epidermidicanis	DSM 45586	y	2.69	58.1	1	NZ_CP011541.1
30	Corynebacterium falsenii	BL 8171	y	2.72	63.2	2	NZ_CP007156.1
31	Corynebacterium flavescens	OJ8	y	2.76	59.9	1	NZ_CP009246.1
32	Corynebacterium fournieri	Marseille-P2948	c	2.38	64.9	24	FWYQ01
33	Corynebacterium frankenforstense	ST18	y	2.60	71.5	1	NZ_CP009247.1
34	Corynebacterium freiburgense	DSM 45254	y	2.91	49.8	19	AUAQ01
35	Corynebacterium freneyi	DNF00450	n	3.04	68.8	90	JRNE01
36	"Corynebacterium genitalium"	ATCC 33030	c	2.35	62.7	1	NZ_CM000961.1ACLJ02
37	Corynebacterium glaucum	DSM 30827	n	2.52	63.0	1	NZ_CP019688.1
38	Corynebacterium glucuronolyticum	DSM 44120	y	2.82	59.1	41	FWWS01
39	Corynebacterium glutamicum	ATCC 13032	y	3.31	53.8	1	NC_003450.3
40	Corynebacterium glyciniphilum	AJ 3170	y	3.57	64.7	2	NZ_CP006842.1
41	Corynebacterium gottingense	NBT06-6	y	2.69	65.1	76	NQMQ01
42	Corynebacterium halotolerans	DSM 44683	y	3.22	68.3	2	NC_020302.1
43	"Corynebacterium heidelbergense"	DSM 104638	c	2.29	65.0	185	PHQP01
44	Corynebacterium humireducens	DSM 45392	y	2.68	68.6	1	NZ_CP005286.1
45	"Corynebacterium ihumii"	GD7	c	2.25	64.9	5	CAVS02
46	Corynebacterium imitans	DSM 44264	y	2.57	64.3	1	NZ_CP009211.1
47	Corynebacterium jeddahense	JCB	y	2.47	67.2	244	CBYN01
48	Corynebacterium jeikeium	NCTC11913	y	2.53	61.4	2	UFXO01
49	"Corynebacterium kefirresidentii"	SB	c	2.63	57.5	472	NGUZ01
50	Corynebacterium kroppenstedtii	DSM 44385	y	2.45	57.5	1	NC_012704.1
51	Corynebacterium kutscheri	DSM 20755	y	2.35	46.5	1	NZ_CP011312.1
52	Corynebacterium lactis	RW2-5	y	2.77	60.5	1	NZ_CP006841.1
53	Corynebacterium lipophiloflavum	DSM 44291	y	2.39	64.2	77	ACHJ01
54	Corynebacterium lowii	NML 130206	y	2.35	62.9	11	LKEV01
55	Corynebacterium lubricantis	DSM 45231	y	2.94	58.6	58	ARAV01
56	Corynebacterium marinum	DSM 44953	y	2.73	67.8	3	NZ_CP007790.1
57	Corynebacterium maris	DSM 45190	y	2.83	66.6	2	NC_021915.1
58	Corynebacterium massiliense	DSM 45435	y	2.18	65.0	26	ATVG01

59	Corynebacterium mastitidis	DSM 44356	y	2.37	69.0	39	AQXB01
60	Corynebacterium matruchotii	ATCC 14266	y	2.86	57.1	8	ACSH02
61	Corynebacterium minutissimum	NCTC10288	y	2.70	59.9	1	NZ_LS483460.1
62	Corynebacterium mustelae	DSM 45274	y	3.47	52.5	3	NZ_CP011542.1
63	Corynebacterium mycetoides	DSM 20632	y	2.27	66.6	1	NZ_LT629700.1
64	Corynebacterium nuruki	S6-4	y	3.11	69.5	68	AFIZ01
65	Corynebacterium oculi	NML 130210	y	2.41	64.8	4	LKST01
66	Corynebacterium otitidis	ATCC 51513	c	2.12	71.0	6	AHAE01
67	Corynebacterium phocae	M408/89/1	y	2.79	58.8	2	NZ_CP009249.1
68	Corynebacterium phoceense	MC1	c	2.79	63.1	14	FLTI01
69	Corynebacterium pilosum	NCTC11862	y	2.59	60.7	2	UFXQ01
70	Corynebacterium pollutisoli	VDS	n	2.54	68.5	15	FXAR01
71	Corynebacterium propinquum	DSM 44285	y	2.46	56.4	22	AQXC01
72	"Corynebacterium provencense"	17KM38	c	3.11	66.9	1	NZ_CP024988.1
73	Corynebacterium pseudodiphtheriticum	DSM 44287	y	2.26	55.3	13	JIAH01
74	"Corynebacterium pseudogenitalium"	ATCC 33035	c	2.60	59.5	9	ABYQ02
75	Corynebacterium pseudotuberculosis	ATCC 19410	y	2.34	52.2	1	NZ_CP021251.1
76	Corynebacterium pyruviciproducens	ATCC BAA-1742	y	2.72	61.1	3	ATBY01
77	Corynebacterium renale	NCTC7448	y	2.34	59.1	1	NZ_LS483464.1
78	Corynebacterium resistens	DSM 45100	y	2.60	57.1	1	NC_015673.1
79	Corynebacterium riegelii	PUDD_83A45	n	2.56	60.5	1	NZ_CP012342.1
80	Corynebacterium simulans	Wattiau	n	2.60	59.3	1	NZ_CP014635.1
81	Corynebacterium singulare	IBS B52218	y	2.83	60.1	1	NZ_CP010827.1
82	Corynebacterium sphenisci	DSM 44792	t	2.59	74.7	1	NZ_CP009248.1
83	Corynebacterium spheniscorum	J11, PG 39	y	2.46	57.5	27	FOPJ01
84	Corynebacterium sputi	DSM 45148	y	2.92	61.5	39	ATYV01
85	Corynebacterium stationis	DSM 20302	y	2.86	54.9	2	NZ_CP009251.1
86	Corynebacterium striatum	NCTC764	y	2.92	59.1	3	UFXV01
87	Corynebacterium terpenotabidum	Y-11	y	2.75	67.0	1	NC_021663.1
88	Corynebacterium testudinoris	DSM 44614	y	2.72	63.1	1	NZ_CP011545.1
89	Corynebacterium timonense	DSM 45434	y	2.63	66.6	1	NZ_LT629765.1
90	Corynebacterium tuberculostearicum	SK141	n	2.37	60.0	37	ACVP01
91	Corynebacterium tuscaniense	UMB0792	n	2.25	59.5	44	PNHG01
92	Corynebacterium ulcerans	NCTC7910	y	2.45	53.3	1	NZ_LT906443.1
93	Corynebacterium ulcerans	FRC11	c2	2.44	53.3	1	NZ_CP009622.1
94	Corynebacterium ulceribovis	DSM 45146	y	2.30	59.2	8	AQUY01
95	Corynebacterium urealyticum	NCTC12011	y	2.38	64.2	1	NZ_LT906481.1
96	Corynebacterium ureicelerivorans	IMMIB RIV-2301	y	2.33	65.0	2	NZ_CP009215.1
97	"Corynebacterium urinapleomorphum"	Marseille-P2799	c	2.26	63.4	13	FTLL01
98	Corynebacterium uterequi	DSM 45634	y	2.42	65.5	1	NZ_CP011546.1
99	Corynebacterium variabile	NRRL B-4201	y	4.71	71.7	29	JOIN01
100	Corynebacterium vitaeruminis	DSM 20294	y	2.93	65.5	1	NZ_CP004353.1
101	Corynebacterium xerosis	NBRC 16721	y	2.69	69.7	14	BCRD01
102	Corynebacterium sp.	3B		2.45	62.9	16	PQMV01
103	Corynebacterium sp.	5A		2.40	62.9	15	PQMU01
104	Corynebacterium sp.	6A		2.45	62.9	16	PQMT01
105	Corynebacterium sp.	7B		2.46	62.7	17	PQMS01
106	Corynebacterium sp.	11A		2.40	62.5	24	PQMR01
107	Corynebacterium sp.	12B		2.46	62.7	16	PQMQ01
108	Corynebacterium sp.	19B		2.45	62.9	14	PQMP01
109	Corynebacterium sp.	20A		2.51	62.7	17	PQMO01
110	Corynebacterium sp.	48B		2.40	62.9	19	PQMN01
111	Corynebacterium sp.	49B		2.40	62.9	17	PQMM01
112	Corynebacterium sp.	50A		2.40	62.9	16	PQML01
113	Corynebacterium sp.	51B		2.40	62.9	12	PQMK01
114	Corynebacterium sp.	52A		2.40	62.9	18	PQMJ01
115	Corynebacterium sp.	71B		2.45	62.9	15	PQMI01
116	Corynebacterium sp.	73A		2.40	62.9	15	PQMH01
117	Corynebacterium sp.	74A		2.46	62.7	11	PQMG01

Note: Some species designations in the genomic data obtained from the GenBank are pending validation. These species designations are mentioned in inverted commas.
