# Supplementary material for: Phylogenomic Characterization of a Novel Corynebacterium Species Associated with Fatal Diphtheritic Stomatitis in Endangered Yellow-Eyed Penguins
Source: mSystems. 2021 Jun 8;6(3):e00320-21. doi: 10.1128/mSystems.00320-21 (PMC8269222; doi:10.1128/mSystems.00320-21)
Supplement: TABLE S2 [file msystems.00320-21-st002.pdf]

S. No	Function	# isolates	Type	Island	Phage	Strain 3B	Strain 5A	Strain 6A	Strain 19B	Strain 48B	Strain 49B	Strain 50A	Strain 51B	Strain 52A	Strain 71B	Strain 73A	Strain 7B	Strain 12B	Strain 20A	Strain 74A	Strain 11A
1	HNH endonuclease	16	Core			hoi102_00001	hoi103_00460	hoi104_00375	hoi108_01662	hoi110_00001	hoi111_00954	hoi112_00001	hoi113_00461	hoi114_00001	hoi115_00001	hoi116_00388	hoi105_00141	hoi107_00141	hoi109_00450	hoi117_00449	hoi106_00140
2	hypothetical protein	16	Core			hoi102_00002	hoi103_00459	hoi104_00374	hoi108_01661	hoi110_00002	hoi111_00953	hoi112_00002	hoi113_00460	hoi114_00002	hoi115_00002	hoi116_00387	hoi105_00142	hoi107_00142	hoi109_00449	hoi117_00448	hoi106_00141
3	Transcription elongation factor GreA	16	Core			hoi102_00003	hoi103_00458	hoi104_00373	hoi108_01660	hoi110_00003	hoi111_00952	hoi112_00003	hoi113_00459	hoi114_00003	hoi115_00003	hoi116_00386	hoi105_00143	hoi107_00143	hoi109_00448	hoi117_00447	hoi106_00142
4	hypothetical protein	16	Core			hoi102_00004	hoi103_00457	hoi104_00372	hoi108_01659	hoi110_00004	hoi111_00951	hoi112_00004	hoi113_00458	hoi114_00004	hoi115_00004	hoi116_00385	hoi105_00144	hoi107_00144	hoi109_00447	hoi117_00446	hoi106_00143
5	Mycothiol S-conjugate amidase	16	Core			hoi102_00005	hoi103_00456	hoi104_00371	hoi108_01658	hoi110_00005	hoi111_00950	hoi112_00005	hoi113_00457	hoi114_00005	hoi115_00005	hoi116_00384	hoi105_00145	hoi107_00145	hoi109_00446	hoi117_00445	hoi106_00144
6	hypothetical protein	16	Core			hoi102_00006	hoi103_00455	hoi104_00370	hoi108_01657	hoi110_00006	hoi111_00949	hoi112_00006	hoi113_00456	hoi114_00006	hoi115_00006	hoi116_00383	hoi105_00146	hoi107_00146	hoi109_00445	hoi117_00444	hoi106_00145
7	(2Z,6E)-farnesyl diphosphate synthase	16	Core			hoi102_00007	hoi103_00454	hoi104_00369	hoi108_01656	hoi110_00007	hoi111_00948	hoi112_00007	hoi113_00455	hoi114_00007	hoi115_00007	hoi116_00382	hoi105_00147	hoi107_00147	hoi109_00444	hoi117_00443	hoi106_00146
8	Flavodoxin domain protein	16	Core			hoi102_00008	hoi103_00453	hoi104_00368	hoi108_01655	hoi110_00008	hoi111_00947	hoi112_00008	hoi113_00454	hoi114_00008	hoi115_00008	hoi116_00381	hoi105_00148	hoi107_00148	hoi109_00443	hoi117_00442	hoi106_00147
9	Pantothenate kinase	16	Core			hoi102_00009	hoi103_00452	hoi104_00367	hoi108_01654	hoi110_00009	hoi111_00946	hoi112_00009	hoi113_00453	hoi114_00009	hoi115_00009	hoi116_00380	hoi105_00149	hoi107_00149	hoi109_00442	hoi117_00441	hoi106_00148
10	Serine hydroxymethyltransferase 1	16	Core			hoi102_00010	hoi103_00451	hoi104_00366	hoi108_01653	hoi110_00010	hoi111_00945	hoi112_00010	hoi113_00452	hoi114_00010	hoi115_00010	hoi116_00379	hoi105_00150	hoi107_00150	hoi109_00441	hoi117_00440	hoi106_00149
11	hypothetical protein	16	Core			hoi102_00011	hoi103_00450	hoi104_00365	hoi108_01652	hoi110_00011	hoi111_00944	hoi112_00011	hoi113_00451	hoi114_00011	hoi115_00011	hoi116_00378	hoi105_00151	hoi107_00151	hoi109_00440	hoi117_00439	hoi106_00150
12	hypothetical protein	16	Core			hoi102_00012	hoi103_00449	hoi104_00364	hoi108_01651	hoi110_00012	hoi111_00943	hoi112_00012	hoi113_00450	hoi114_00012	hoi115_00012	hoi116_00377	hoi105_00152	hoi107_00152	hoi109_00439	hoi117_00438	hoi106_00151
13	Putative phosphinothricin acetyltransferase Ywn	16	Core			hoi102_00013	hoi103_00448	hoi104_00363	hoi108_01650	hoi110_00013	hoi111_00942	hoi112_00013	hoi113_00449	hoi114_00013	hoi115_00013	hoi116_00376	hoi105_00153	hoi107_00153	hoi109_00438	hoi117_00437	hoi106_00152
14	Multidrug resistance protein 3	16	Core			hoi102_00014	hoi103_00447	hoi104_00362	hoi108_01649	hoi110_00014	hoi111_00941	hoi112_00014	hoi113_00448	hoi114_00014	hoi115_00014	hoi116_00375	hoi105_00154	hoi107_00154	hoi109_00437	hoi117_00436	hoi106_00153
15	Bacterial regulatory proteins, tetR family	16	Core			hoi102_00015	hoi103_00446	hoi104_00361	hoi108_01648	hoi110_00015	hoi111_00940	hoi112_00015	hoi113_00447	hoi114_00015	hoi115_00015	hoi116_00374	hoi105_00155	hoi107_00155	hoi109_00436	hoi117_00435	hoi106_00154
16	Fumarate hydratase class II	16	Core			hoi102_00016	hoi103_00445	hoi104_00360	hoi108_01647	hoi110_00016	hoi111_00939	hoi112_00016	hoi113_00446	hoi114_00016	hoi115_00016	hoi116_00373	hoi105_00156	hoi107_00156	hoi109_00435	hoi117_00434	hoi106_00155
17	putative inner membrane protein	16	Core			hoi102_00017	hoi103_00444	hoi104_00359	hoi108_01646	hoi110_00017	hoi111_00938	hoi112_00017	hoi113_00445	hoi114_00017	hoi115_00017	hoi116_00372	hoi105_00157	hoi107_00157	hoi109_00434	hoi117_00433	hoi106_00156
18	NADH dehydrogenase	16	Core			hoi102_00018	hoi103_00443	hoi104_00358	hoi108_01645	hoi110_00018	hoi111_00937	hoi112_00018	hoi113_00444	hoi114_00018	hoi115_00018	hoi116_00371	hoi105_00158	hoi107_00158	hoi109_00433	hoi117_00432	hoi106_00157
19	Anguibactin system regulator	16	Core	GI1		hoi102_00019	hoi103_00442	hoi104_00357	hoi108_01644	hoi110_00019	hoi111_00936	hoi112_00019	hoi113_00443	hoi114_00019	hoi115_00019	hoi116_00370	hoi105_00159	hoi107_00159	hoi109_00432	hoi117_00431	hoi106_00158
20	Iron import ATP-binding/permease protein IrtA	16	Core	GI1		hoi102_00020	hoi103_00441	hoi104_00356	hoi108_01643	hoi110_00020	hoi111_00935	hoi112_00020	hoi113_00442	hoi114_00020	hoi115_00020	hoi116_00369	hoi105_00160	hoi107_00160	hoi109_00431	hoi117_00430	hoi106_00159
21	Heterocyst differentiation ATP-binding protein H	16	Core	GI1		hoi102_00021	hoi103_00440	hoi104_00355	hoi108_01642	hoi110_00021	hoi111_00934	hoi112_00021	hoi113_00441	hoi114_00021	hoi115_00021	hoi116_00368	hoi105_00161	hoi107_00161	hoi109_00430	hoi117_00429	hoi106_00160
22	Polyketide synthase PksR	16	Core	GI1		hoi102_00022	hoi103_00439	hoi104_00354	hoi108_01641	hoi110_00022	hoi111_00933	hoi112_00022	hoi113_00440	hoi114_00022	hoi115_00022	hoi116_00367	hoi105_00162	hoi107_00162	hoi109_00429	hoi117_00428	hoi106_00161
23	Phthiocerol synthesis polyketide synthase type I	16	Core	GI1		hoi102_00023	hoi103_00438	hoi104_00353	hoi108_01640	hoi110_00023	hoi111_00932	hoi112_00023	hoi113_00439	hoi114_00023	hoi115_00023	hoi116_00366	hoi105_00163	hoi107_00163	hoi109_00428	hoi117_00427	hoi106_00162
24	Periplasmic oligopeptide-binding protein precurs	16	Core			hoi102_00024	hoi103_00437	hoi104_00352	hoi108_01639	hoi110_00024	hoi111_00931	hoi112_00024	hoi113_00438	hoi114_00024	hoi115_00024	hoi116_00365	hoi105_00164	hoi107_00164	hoi109_00427	hoi117_00426	hoi106_00163
25	Dipeptide transport system permease protein Dp	16	Core			hoi102_00025	hoi103_00436	hoi104_00351	hoi108_01638	hoi110_00025	hoi111_00930	hoi112_00025	hoi113_00437	hoi114_00025	hoi115_00025	hoi116_00364	hoi105_00165	hoi107_00165	hoi109_00426	hoi117_00425	hoi106_00164
26	Methionine import ATP-binding protein MetN	16	Core			hoi102_00026	hoi103_00435	hoi104_00350	hoi108_01637	hoi110_00026	hoi111_00929	hoi112_00026	hoi113_00436	hoi114_00026	hoi115_00026	hoi116_00363	hoi105_00166	hoi107_00166	hoi109_00425	hoi117_00424	hoi106_00165
27	Oxygen-dependent choline dehydrogenase	16	Core			hoi102_00027	hoi103_00434	hoi104_00349	hoi108_01636	hoi110_00027	hoi111_00928	hoi112_00027	hoi113_00435	hoi114_00027	hoi115_00027	hoi116_00362	hoi105_00167	hoi107_00167	hoi109_00424	hoi117_00423	hoi106_00166
28	High-affinity choline transport protein	16	Core			hoi102_00028	hoi103_00433	hoi104_00348	hoi108_01635	hoi110_00028	hoi111_00927	hoi112_00028	hoi113_00434	hoi114_00028	hoi115_00028	hoi116_00361	hoi105_00168	hoi107_00168	hoi109_00423	hoi117_00422	hoi106_00167
29	Betaine aldehyde dehydrogenase	16	Core			hoi102_00029	hoi103_00432	hoi104_00347	hoi108_01634	hoi110_00029	hoi111_00926	hoi112_00029	hoi113_00433	hoi114_00029	hoi115_00029	hoi116_00360	hoi105_00169	hoi107_00169	hoi109_00422	hoi117_00421	hoi106_00168
30	Fructose-1,6-bisphosphatase class 2	16	Core			hoi102_00030	hoi103_00431	hoi104_00346	hoi108_01633	hoi110_00030	hoi111_00925	hoi112_00030	hoi113_00432	hoi114_00030	hoi115_00030	hoi116_00359	hoi105_00170	hoi107_00170	hoi109_00421	hoi117_00420	hoi106_00169
31	hypothetical protein	16	Core			hoi102_00031	hoi103_00430	hoi104_00345	hoi108_01632	hoi110_00031	hoi111_00924	hoi112_00031	hoi113_00431	hoi114_00031	hoi115_00031	hoi116_00358	hoi105_00171	hoi107_00171	hoi109_00420	hoi117_00419	hoi106_00170
32	exodeoxyribonuclease VII small subunit	16	Core			hoi102_00032	hoi103_00429	hoi104_00344	hoi108_01631	hoi110_00032	hoi111_00923	hoi112_00032	hoi113_00430	hoi114_00032	hoi115_00032	hoi116_00357	hoi105_00172	hoi107_00172	hoi109_00419	hoi117_00418	hoi106_00171
33	Exodeoxyribonuclease 7 large subunit	16	Core			hoi102_00033	hoi103_00428	hoi104_00343	hoi108_01630	hoi110_00033	hoi111_00922	hoi112_00033	hoi113_00429	hoi114_00033	hoi115_00033	hoi116_00356	hoi105_00173	hoi107_00173	hoi109_00418	hoi117_00417	hoi106_00172
34	4-hydroxy-3-methylbut-2-enyl diphosphate redu	16	Core			hoi102_00034	hoi103_00427	hoi104_00342	hoi108_01629	hoi110_00034	hoi111_00921	hoi112_00034	hoi113_00428	hoi114_00034	hoi115_00034	hoi116_00355	hoi105_00174	hoi107_00174	hoi109_00417	hoi117_00416	hoi106_00173
35	hypothetical protein	16	Core			hoi102_00035	hoi103_00426	hoi104_00341	hoi108_01628	hoi110_00035	hoi111_00920	hoi112_00035	hoi113_00427	hoi114_00035	hoi115_00035	hoi116_00354	hoi105_00175	hoi107_00175	hoi109_00416	hoi117_00415	hoi106_00174
36	DNA recombination protein RmuC	16	Core			hoi102_00036	hoi103_00425	hoi104_00340	hoi108_01627	hoi110_00036	hoi111_00919	hoi112_00036	hoi113_00426	hoi114_00036	hoi115_00036	hoi116_00353	hoi105_00176	hoi107_00176	hoi109_00415	hoi117_00414	hoi106_00175
37	pheromone autoinducer 2 transporter	16	Core			hoi102_00037	hoi103_00424	hoi104_00339	hoi108_01626	hoi110_00037	hoi111_00918	hoi112_00037	hoi113_00425	hoi114_00037	hoi115_00037	hoi116_00352	hoi105_00177	hoi107_00177	hoi109_00414	hoi117_00413	hoi106_00176
38	Ribosome-binding ATPase YchF	16	Core			hoi102_00038	hoi103_00423	hoi104_00338	hoi108_01625	hoi110_00038	hoi111_00917	hoi112_00038	hoi113_00424	hoi114_00038	hoi115_00038	hoi116_00351	hoi105_00178	hoi107_00178	hoi109_00413	hoi117_00412	hoi106_00177
39	hypothetical protein	4	Accessory														hoi105_00179	hoi107_00179	hoi109_00412	hoi117_00411	
40	hypothetical protein	11	Accessory	GI2		hoi102_00039	hoi103_00422	hoi104_00337	hoi108_01624	hoi110_00039	hoi111_00916	hoi112_00039	hoi113_00423	hoi114_00039	hoi115_00039	hoi116_00350					
41	Protein of unknown function (Hypoth_ymh)	11	Accessory	GI2		hoi102_00040	hoi103_00421	hoi104_00336	hoi108_01623	hoi110_00040	hoi111_00915	hoi112_00040	hoi113_00422	hoi114_00040	hoi115_00040	hoi116_00349					
42	N-6 DNA Methylase	11	Accessory	GI2		hoi102_00041	hoi103_00420	hoi104_00335	hoi108_01622	hoi110_00041	hoi111_00914	hoi112_00041	hoi113_00421	hoi114_00041	hoi115_00041	hoi116_00348					
43	hypothetical protein	11	Accessory	GI2		hoi102_00042	hoi103_00419	hoi104_00334	hoi108_01621	hoi110_00042	hoi111_00913	hoi112_00042	hoi113_00420	hoi114_00042	hoi115_00042	hoi116_00347					
44	hypothetical protein	11	Accessory	GI2		hoi102_00043	hoi103_00418	hoi104_00333	hoi108_01620	hoi110_00043	hoi111_00912	hoi112_00043	hoi113_00419	hoi114_00043	hoi115_00043	hoi116_00346					
45	hypothetical protein	11	Accessory	GI2		hoi102_00044	hoi103_00417	hoi104_00332	hoi108_01619	hoi110_00044	hoi111_00911	hoi112_00044	hoi113_00418	hoi114_00044	hoi115_00044	hoi116_00345					
46	Low molecular weight protein-tyrosine-phosphat	15	Accessory			hoi102_00045	hoi103_00416	hoi104_00331	hoi108_01618	hoi110_00045	hoi111_00910	hoi112_00045	hoi113_00417	hoi114_00045	hoi115_00045	hoi116_00344	hoi105_00180	hoi107_00180	hoi109_00411	hoi117_00410	
47	hypothetical protein	15	Accessory			hoi102_00046	hoi103_00415	hoi104_00330	hoi108_01617	hoi110_00046	hoi111_00909	hoi112_00046	hoi113_00416	hoi114_00046	hoi115_00046	hoi116_00343	hoi105_00181	hoi107_00181	hoi109_00410	hoi117_00409	
48	coenzyme F420-reducing hydrogenase subunit b	15	Accessory			hoi102_00047	hoi103_00414	hoi104_00329	hoi108_01616	hoi110_00047	hoi111_00908	hoi112_00047	hoi113_00415	hoi114_00047	hoi115_00047	hoi116_00342	hoi105_00182	hoi107_00182	hoi109_00409	hoi117_00408	
49	Putative N-acetylmannosaminyltransferase	15	Accessory			hoi102_00048	hoi103_00413	hoi104_00328	hoi108_01615	hoi110_00048	hoi111_00907	hoi112_00048	hoi113_00414	hoi114_00048	hoi115_00048	hoi116_00341	hoi105_00183	hoi107_00183	hoi109_00408	hoi117_00407	
50	hypothetical protein	15	Accessory			hoi102_00049	hoi103_00412	hoi104_00327	hoi108_01614	hoi110_00049	hoi111_00906	hoi112_00049	hoi113_00413	hoi114_00049	hoi115_00049	hoi116_00340	hoi105_00184	hoi107_00184	hoi109_00407	hoi117_00406	
51	Polysaccharide pyruvyl transferase	15	Accessory			hoi102_00050	hoi103_00411	hoi104_00326	hoi108_01613	hoi110_00050	hoi111_00905	hoi112_00050	hoi113_00412	hoi114_00050	hoi115_00050	hoi116_00339	hoi105_00185	hoi107_00185	hoi109_00406	hoi117_00405	
52	Tyrosine-protein kinase YwqD	15	Accessory			hoi102_00051	hoi103_00410	hoi104_00325	hoi108_01612	hoi110_00051	hoi111_00904	hoi112_00051	hoi113_00411	hoi114_00051	hoi115_00051	hoi116_00338	hoi105_00186	hoi107_00186	hoi109_00405	hoi117_00404	
53	UDP-glucose:undecaprenyl-phosphate glucose-1	15	Accessory			hoi102_00052	hoi103_00409	hoi104_00324	hoi108_01611	hoi110_00052	hoi111_00903	hoi112_00052	hoi113_00410	hoi114_00052	hoi115_00052	hoi116_00337	hoi105_00187	hoi107_00187	hoi109_00404	hoi117_00403	
54	Biofilm regulatory protein A precursor	16	Core			hoi102_00053	hoi103_00408	hoi104_00323	hoi108_01610	hoi110_00053	hoi111_00902	hoi112_00053	hoi113_00409	hoi114_00053	hoi115_00053	hoi116_00336	hoi105_00188	hoi107_00188	hoi109_00403	hoi117_00402	hoi106_00182
55	hypothetical protein	16	Core			hoi102_00054	hoi103_00407	hoi104_00322	hoi108_01609	hoi110_00054	hoi111_00901	hoi112_00054	hoi113_00408	hoi114_00054	hoi115_00054	hoi116_00335	hoi105_00189	hoi107_00189	hoi109_00402	hoi117_00401	hoi106_00183
56	hypothetical protein	16	Core			hoi102_00055	hoi103_00406	hoi104_00321	hoi108_01608	hoi110_00055	hoi111_00900	hoi112_00055	hoi113_00407	hoi114_00055	hoi115_00055	hoi116_00334	hoi105_00190	hoi107_00190	hoi109_00401	hoi117_00400	hoi106_00184
57	hypothetical protein	16	Core			hoi102_00056	hoi103_00405	hoi104_00320	hoi108_01607	hoi110_00056	hoi111_00899	hoi112_00056	hoi113_00406	hoi114_00056	hoi115_00056	hoi116_00333	hoi105_00191	hoi107_00191	hoi109_00400	hoi117_00399	hoi106_00185
58	Glycerate kinase	16	Core			hoi102_00057	hoi103_00404	hoi104_00319	hoi108_01606	hoi110_00057	hoi111_00898	hoi112_00057	hoi113_00405	hoi114_00057	hoi115_00057	hoi116_00332	hoi105_00192	hoi107_00192	hoi109_00399	hoi117_00398	hoi106_00186
59	Alpha/beta hydrolase family protein	16	Core			hoi102_00058	hoi103_00403	hoi104_00318	hoi108_01605	hoi110_00058	hoi111_00897	hoi112_00058	hoi113_00404	hoi114_00058	hoi115_00058	hoi116_00331	hoi105_00193	hoi107_00193	hoi109_00398	hoi117_00397	hoi106_00187
60	NADP-specific glutamate dehydrogenase	16	Core			hoi102_00059	hoi103_00402	hoi104_00317	hoi108_01604	hoi110_00059	hoi111_00896	hoi112_00059	hoi113_00403	hoi114_00059	hoi115_00059	hoi116_00330	hoi105_00194	hoi107_00194	hoi109_00397	hoi117_00396	hoi106_00188
61	V-type ATP synthase subunit E	16	Core			hoi102_00060	hoi103_00401	hoi104_00316	hoi108_01603	hoi110_00060	hoi111_00895	hoi112_00060	hoi113_00402	hoi114_00060	hoi115_00060	hoi116_00329	hoi105_00195	hoi107_00195	hoi109_00396	hoi117_00395	hoi106_00189
62	hypothetical protein	16	Core			hoi102_00061	hoi103_00400	hoi104_00315	hoi108_01602	hoi110_00061	hoi111_00894	hoi112_00061	hoi113_00401	hoi114_00061	hoi115_00061	hoi116_00328	hoi105_00196	hoi107_00196	hoi109_00395	hoi117_00394	hoi106_00190
63	Ribonuclease 3	16	Core			hoi102_00062	hoi103_00399	hoi104_00314	hoi108_01601	hoi110_00062	hoi111_00893	hoi112_00062	hoi113_00400	hoi114_00062	hoi115_00062	hoi116_00327	hoi105_00197	hoi107_00197	hoi109_00394	hoi117_00393	hoi106_00191
64	Formamidopyrimidine-DNA glycosylase 1	16	Core			hoi102_00063	hoi103_00398	hoi104_00313	hoi108_01600	hoi110_00063	hoi111_00892	hoi112_00063	hoi113_00399	hoi114_00063	hoi115_00063	hoi116_00326	hoi105_00198	hoi107_00198	hoi109_00393	hoi117_00392	hoi106_00192
65	Amino-acid carrier protein AlsT	16	Core			hoi102_00064	hoi103_00397	hoi104_00312	hoi108_01599	hoi110_00064	hoi111_00891	hoi112_00064	hoi113_00398	hoi114_00064	hoi115_00064	hoi116_00325	hoi105_00199	hoi107_00199	hoi109_00392	hoi117_00391	hoi106_00193
66	Acylphosphatase	16	Core			hoi102_00065	hoi103_00396	hoi104_00311	hoi108_01598	hoi110_00065	hoi111_00890	hoi112_00065	hoi113_00397	hoi114_00065	hoi115_00065	hoi116_00324	hoi105_00200	hoi107_00200	hoi109_00391	hoi117_00390	hoi106_00194
67	Chromosome partition protein Smc	16	Core			hoi102_00066	hoi103_00395	hoi104_00310	hoi108_01597	hoi110_00066	hoi111_00889	hoi112_00066	hoi113_00396	hoi114_00066	hoi115_00066	hoi116_00323	hoi105_00201	hoi107_00201	hoi109_00390	hoi117_00389	hoi106_00195
68	hypothetical protein	16	Core			hoi102_00067	hoi103_00394	hoi104_00309	hoi108_01596	hoi110_00067	hoi111_00888	hoi112_00067	hoi113_00395	hoi114_00067	hoi115_00067	hoi116_00322	hoi105_00202	hoi107_00202	hoi109_00389	hoi117_00388	hoi106_00196
69	Signal recognition particle receptor FtsY	16	Core			hoi102_00068	hoi103_00393	hoi104_00308	hoi108_01595	hoi110_00068	hoi111_00887	hoi112_00068	hoi113_00394	hoi114_00068	hoi115_00068	hoi116_00321	hoi105_00203	hoi107_00203	hoi109_00388	hoi117_00387	hoi106_00197
70	Ammonia channel precursor	16	Core			hoi102_00069	hoi103_00392	hoi104_00307	hoi108_01594	hoi110_00069	hoi111_00886	hoi112_00069	hoi113_00393	hoi114_00069	hoi115_00069	hoi116_00320	hoi105_00204	hoi107_00204	hoi109_00387	hoi117_00386	hoi106_00198
71	Nitrogen regulatory protein P-II	16	Core			hoi102_00070	hoi103_00391	hoi104_00306	hoi108_01593	hoi110_00070	hoi111_00885	hoi112_00070	hoi113_00392	hoi114_00070	hoi115_00070	hoi116_00319	hoi105_00205	hoi107_00205	hoi109_00386	hoi117_00385	hoi106_00199
72	Bifunctional uridylyltransferase/uridylyl-removin	16	Core			hoi102_00071	hoi103_00390	hoi104_00305	hoi108_01592	hoi110_00071	hoi111_00884	hoi112_00071	hoi113_00391	hoi114_00071	hoi115_00071	hoi116_00318	hoi105_00206	hoi107_00206	hoi109_00385	hoi117_00384	hoi106_00200
73	Signal recognition particle protein	16	Core			hoi102_00072	hoi103_00389	hoi104_00304	hoi108_01591	hoi110_00072	hoi111_00883	hoi112_00072	hoi113_00390	hoi114_00072	hoi115_00072	hoi116_00317	hoi105_00207	hoi107_00207	hoi109_00384	hoi117_00383	hoi106_00201
74	O-acetyltransferase OatA	16	Core			hoi102_00073	hoi103_00388	hoi104_00303	hoi108_01590	hoi110_00073	hoi111_00882	hoi112_00073	hoi113_00389	hoi114_00073	hoi115_00073	hoi116_00316	hoi105_00208	hoi107_00208	hoi109_00383	hoi117_00382	hoi106_00202
75	30S ribosomal protein S16	16	Core			hoi102_00074	hoi103_00387	hoi104_00302	hoi108_01589	hoi110_00074	hoi111_00881	hoi112_00074	hoi113_00388	hoi114_00074	hoi115_00074	hoi116_00315	hoi105_00209	hoi107_00209	hoi109_00382	hoi117_00381	hoi106_00203
76	Ribosome maturation factor RimM	16	Core			hoi102_00075	hoi103_00386	hoi104_00301	hoi108_01588	hoi110_00075	hoi111_00880	hoi112_00075	hoi113_00387	hoi114_00075	hoi115_00075	hoi116_00314	hoi105_00210	hoi107_00210	hoi109_00381	hoi117_00380	hoi106_00204
77	tRNA (guanine-N(1)-)-methyltransferase	16	Core			hoi102_00076	hoi103_00385	hoi104_00300	hoi108_01587	hoi110_00076	hoi111_00879	hoi112_00076	hoi113_00386	hoi114_00076	hoi115_00076	hoi116_00313	hoi105_00211	hoi107_00211	hoi109_00380	hoi117_00379	hoi106_00205

78	High-affinity choline transport protein	16	Core		hoi102_00077	hoi103_00384	hoi104_00298	hoi108_01586	hoi110_00078	hoi111_00877	hoi112_00078	hoi113_00384	hoi114_00077	hoi115_00078	hoi116_00312	hoi105_00212	hoi107_00212	hoi109_00379	hoi117_00378	hoi106_00206
79	hypothetical protein	16	Core		hoi102_00078	hoi103_00383	hoi104_00297	hoi108_01585	hoi110_00079	hoi111_00876	hoi112_00079	hoi113_00383	hoi114_00078	hoi115_00079	hoi116_00311	hoi105_00213	hoi107_00213	hoi109_00378	hoi117_00377	hoi106_00207
80	hypothetical protein	16	Core		hoi102_00079	hoi103_00382	hoi104_00296	hoi108_01584	hoi110_00080	hoi111_00875	hoi112_00080	hoi113_00382	hoi114_00079	hoi115_00080	hoi116_00310	hoi105_00214	hoi107_00214	hoi109_00377	hoi117_00376	hoi106_00208
81	Thiazole synthase	16	Core		hoi102_00080	hoi103_00381	hoi104_00295	hoi108_01583	hoi110_00081	hoi111_00874	hoi112_00081	hoi113_00381	hoi114_00080	hoi115_00081	hoi116_00309	hoi105_00215	hoi107_00215	hoi109_00376	hoi117_00375	hoi106_00209
82	sulfur carrier protein ThiS	16	Core		hoi102_00081	hoi103_00380	hoi104_00294	hoi108_01582	hoi110_00082	hoi111_00873	hoi112_00082	hoi113_00380	hoi114_00081	hoi115_00082	hoi116_00308	hoi105_00216	hoi107_00216	hoi109_00375	hoi117_00374	hoi106_00210
83	Hydrogen cyanide synthase subunit HcnC precur	16	Core		hoi102_00082	hoi103_00379	hoi104_00293	hoi108_01581	hoi110_00083	hoi111_00872	hoi112_00083	hoi113_00379	hoi114_00082	hoi115_00083	hoi116_00307	hoi105_00217	hoi107_00217	hoi109_00374	hoi117_00373	hoi106_00211
84	Thiamine-phosphate synthase	16	Core		hoi102_00083	hoi103_00378	hoi104_00292	hoi108_01580	hoi110_00084	hoi111_00871	hoi112_00084	hoi113_00378	hoi114_00083	hoi115_00084	hoi116_00306	hoi105_00218	hoi107_00218	hoi109_00373	hoi117_00372	hoi106_00212
85	hypothetical protein	16	Core		hoi102_00084	hoi103_00377	hoi104_00291	hoi108_01579	hoi110_00085	hoi111_00870	hoi112_00085	hoi113_00377	hoi114_00084	hoi115_00085	hoi116_00305	hoi105_00219	hoi107_00219	hoi109_00372	hoi117_00371	hoi106_00213
86	50S ribosomal protein L19	16	Core		hoi102_00085	hoi103_00376	hoi104_00290	hoi108_01578	hoi110_00086	hoi111_00869	hoi112_00086	hoi113_00376	hoi114_00085	hoi115_00086	hoi116_00304	hoi105_00220	hoi107_00220	hoi109_00371	hoi117_00370	hoi106_00214
87	Signal peptidase I	16	Core		hoi102_00086	hoi103_00375	hoi104_00289	hoi108_01577	hoi110_00087	hoi111_00868	hoi112_00087	hoi113_00375	hoi114_00086	hoi115_00087	hoi116_00303	hoi105_00221	hoi107_00221	hoi109_00370	hoi117_00369	hoi106_00215
88	Ribonuclease HII	16	Core		hoi102_00087	hoi103_00374	hoi104_00288	hoi108_01576	hoi110_00088	hoi111_00867	hoi112_00088	hoi113_00374	hoi114_00087	hoi115_00088	hoi116_00302	hoi105_00222	hoi107_00222	hoi109_00369	hoi117_00368	hoi106_00216
89	hypothetical protein	16	Core		hoi102_00088	hoi103_00373	hoi104_00287	hoi108_01575	hoi110_00089	hoi111_00866	hoi112_00089	hoi113_00373	hoi114_00088	hoi115_00089	hoi116_00301	hoi105_00223	hoi107_00223	hoi109_00368	hoi117_00367	hoi106_00217
90	hypothetical protein	16	Core		hoi102_00089	hoi103_00372	hoi104_00286	hoi108_01574	hoi110_00090	hoi111_00865	hoi112_00090	hoi113_00372	hoi114_00089	hoi115_00090	hoi116_00300	hoi105_00224	hoi107_00224	hoi109_00367	hoi117_00366	hoi106_00218
91	Competence protein ComM	16	Core		hoi102_00090	hoi103_00371	hoi104_00285	hoi108_01573	hoi110_00091	hoi111_00864	hoi112_00091	hoi113_00371	hoi114_00090	hoi115_00091	hoi116_00299	hoi105_00225	hoi107_00225	hoi109_00366	hoi117_00365	hoi106_00219
92	hypothetical protein	16	Core		hoi102_00091	hoi103_00370	hoi104_00284	hoi108_01572	hoi110_00092	hoi111_00863	hoi112_00092	hoi113_00370	hoi114_00091	hoi115_00092	hoi116_00298	hoi105_00226	hoi107_00226	hoi109_00365	hoi117_00364	hoi106_00220
93	Tyrosine recombinase XerC	16	Core		hoi102_00092	hoi103_00369	hoi104_00283	hoi108_01571	hoi110_00093	hoi111_00862	hoi112_00093	hoi113_00369	hoi114_00092	hoi115_00093	hoi116_00297	hoi105_00227	hoi107_00227	hoi109_00364	hoi117_00363	hoi106_00221
94	Peptidase family M23	16	Core		hoi102_00093	hoi103_00368	hoi104_00282	hoi108_01570	hoi110_00094	hoi111_00861	hoi112_00094	hoi113_00368	hoi114_00093	hoi115_00094	hoi116_00296	hoi105_00228	hoi107_00228	hoi109_00363	hoi117_00362	hoi106_00222
95	30S ribosomal protein S2	16	Core		hoi102_00094	hoi103_00367	hoi104_00281	hoi108_01569	hoi110_00095	hoi111_00860	hoi112_00095	hoi113_00367	hoi114_00094	hoi115_00095	hoi116_00295	hoi105_00229	hoi107_00229	hoi109_00362	hoi117_00361	hoi106_00223
96	Elongation factor Ts	16	Core		hoi102_00095	hoi103_00366	hoi104_00280	hoi108_01568	hoi110_00096	hoi111_00859	hoi112_00096	hoi113_00366	hoi114_00095	hoi115_00096	hoi116_00294	hoi105_00230	hoi107_00230	hoi109_00361	hoi117_00360	hoi106_00224
97	Uridylate kinase	16	Core		hoi102_00096	hoi103_00365	hoi104_00279	hoi108_01567	hoi110_00097	hoi111_00858	hoi112_00097	hoi113_00365	hoi114_00096	hoi115_00097	hoi116_00293	hoi105_00231	hoi107_00231	hoi109_00360	hoi117_00359	hoi106_00225
98	Ribosome-recycling factor	16	Core		hoi102_00097	hoi103_00364	hoi104_00278	hoi108_01566	hoi110_00098	hoi111_00857	hoi112_00098	hoi113_00364	hoi114_00097	hoi115_00098	hoi116_00292	hoi105_00232	hoi107_00232	hoi109_00359	hoi117_00358	hoi106_00226
99	Phosphatidate cytidylyltransferase	16	Core		hoi102_00098	hoi103_00363	hoi104_00277	hoi108_01565	hoi110_00099	hoi111_00856	hoi112_00099	hoi113_00363	hoi114_00098	hoi115_00099	hoi116_00291	hoi105_00233	hoi107_00233	hoi109_00358	hoi117_00357	hoi106_00227
100	Antiseptic resistance protein	16	Core		hoi102_00099	hoi103_00362	hoi104_00276	hoi108_01564	hoi110_00100	hoi111_00855	hoi112_00100	hoi113_00362	hoi114_00099	hoi115_00100	hoi116_00290	hoi105_00234	hoi107_00234	hoi109_00357	hoi117_00356	hoi106_00228
101	hypothetical protein	16	Core		hoi102_00100	hoi103_00361	hoi104_00275	hoi108_01563	hoi110_00101	hoi111_00854	hoi112_00101	hoi113_00361	hoi114_00100	hoi115_00101	hoi116_00289	hoi105_00235	hoi107_00235	hoi109_00356	hoi117_00355	hoi106_00229
102	putative dual-specificity RNA methyltransferase I	16	Core		hoi102_00101	hoi103_00360	hoi104_00274	hoi108_01562	hoi110_00102	hoi111_00853	hoi112_00102	hoi113_00360	hoi114_00101	hoi115_00102	hoi116_00288	hoi105_00236	hoi107_00236	hoi109_00355	hoi117_00354	hoi106_00230
103	hypothetical protein	16	Core		hoi102_00102	hoi103_00359	hoi104_00273	hoi108_01561	hoi110_00103	hoi111_00852	hoi112_00103	hoi113_00359	hoi114_00102	hoi115_00103	hoi116_00287	hoi105_00237	hoi107_00237	hoi109_00354	hoi117_00353	hoi106_00231
104	1-deoxy-D-xylulose 5-phosphate reductoisomera	16	Core		hoi102_00103	hoi103_00358	hoi104_00272	hoi108_01560	hoi110_00104	hoi111_00851	hoi112_00104	hoi113_00358	hoi114_00103	hoi115_00104	hoi116_00286	hoi105_00238	hoi107_00238	hoi109_00353	hoi117_00352	hoi106_00232
105	Zinc metalloprotease Rip1	16	Core		hoi102_00104	hoi103_00357	hoi104_00271	hoi108_01559	hoi110_00105	hoi111_00850	hoi112_00105	hoi113_00357	hoi114_00104	hoi115_00105	hoi116_00285	hoi105_00239	hoi107_00239	hoi109_00352	hoi117_00351	hoi106_00233
106	4-hydroxy-3-methylbut-2-en-1-yl diphosphate sy	16	Core		hoi102_00105	hoi103_00356	hoi104_00270	hoi108_01558	hoi110_00106	hoi111_00849	hoi112_00106	hoi113_00356	hoi114_00105	hoi115_00106	hoi116_00284	hoi105_00240	hoi107_00240	hoi109_00351	hoi117_00350	hoi106_00234
107	Methionine aminopeptidase 2	16	Core		hoi102_00106	hoi103_00355	hoi104_00269	hoi108_01557	hoi110_00107	hoi111_00848	hoi112_00107	hoi113_00355	hoi114_00106	hoi115_00107	hoi116_00283	hoi105_00241	hoi107_00241	hoi109_00350	hoi117_00349	hoi106_00235
108	Cobyric acid synthase	16	Core		hoi102_00107	hoi103_00354	hoi104_00268	hoi108_01556	hoi110_00108	hoi111_00847	hoi112_00108	hoi113_00354	hoi114_00107	hoi115_00108	hoi116_00282	hoi105_00242	hoi107_00242	hoi109_00349	hoi117_00348	hoi106_00236
109	hypothetical protein	16	Core		hoi102_00108	hoi103_00353	hoi104_00267	hoi108_01555	hoi110_00109	hoi111_00846	hoi112_00109	hoi113_00353	hoi114_00108	hoi115_00109	hoi116_00281	hoi105_00243	hoi107_00243	hoi109_00348	hoi117_00347	hoi106_00237
110	Mycothione reductase	16	Core		hoi102_00109	hoi103_00352	hoi104_00266	hoi108_01554	hoi110_00110	hoi111_00845	hoi112_00110	hoi113_00352	hoi114_00109	hoi115_00110	hoi116_00280	hoi105_00244	hoi107_00244	hoi109_00347	hoi117_00346	hoi106_00238
111	Alpha/beta hydrolase family protein	16	Core		hoi102_00110	hoi103_00351	hoi104_00265	hoi108_01553	hoi110_00111	hoi111_00844	hoi112_00111	hoi113_00351	hoi114_00110	hoi115_00111	hoi116_00279	hoi105_00245	hoi107_00245	hoi109_00346	hoi117_00345	hoi106_00239
112	Malate:quinone oxidoreductase	16	Core		hoi102_00111	hoi103_00350	hoi104_00264	hoi108_01552	hoi110_00112	hoi111_00843	hoi112_00112	hoi113_00350	hoi114_00111	hoi115_00112	hoi116_00278	hoi105_00246	hoi107_00246	hoi109_00345	hoi117_00344	hoi106_00240
113	Magnesium-chelatase 38 kDa subunit	16	Core		hoi102_00112	hoi103_00349	hoi104_00263	hoi108_01551	hoi110_00113	hoi111_00842	hoi112_00113	hoi113_00349	hoi114_00112	hoi115_00113	hoi116_00277	hoi105_00247	hoi107_00247	hoi109_00344	hoi117_00343	hoi106_00241
114	Magnesium-chelatase 60 kDa subunit	16	Core		hoi102_00113	hoi103_00348	hoi104_00262	hoi108_01550	hoi110_00114	hoi111_00841	hoi112_00114	hoi113_00348	hoi114_00113	hoi115_00114	hoi116_00276	hoi105_00248	hoi107_00248	hoi109_00343	hoi117_00342	hoi106_00242
115	Cob(I)yrinic acid a,c-diamide adenosyltransferase	16	Core		hoi102_00114	hoi103_00347	hoi104_00261	hoi108_01549	hoi110_00115	hoi111_00840	hoi112_00115	hoi113_00347	hoi114_00114	hoi115_00115	hoi116_00275	hoi105_00249	hoi107_00249	hoi109_00342	hoi117_00341	hoi106_00243
116	Cobyrinic acid A,C-diamide synthase	16	Core		hoi102_00115	hoi103_00346	hoi104_00260	hoi108_01548	hoi110_00116	hoi111_00839	hoi112_00116	hoi113_00346	hoi114_00115	hoi115_00116	hoi116_00274	hoi105_00250	hoi107_00250	hoi109_00341	hoi117_00340	hoi106_00244
117	Uroporphyrinogen-III C-methyltransferase	16	Core		hoi102_00116	hoi103_00345	hoi104_00259	hoi108_01547	hoi110_00117	hoi111_00838	hoi112_00117	hoi113_00345	hoi114_00116	hoi115_00117	hoi116_00273	hoi105_00251	hoi107_00251	hoi109_00340	hoi117_00339	hoi106_00245
118	hypothetical protein	16	Core		hoi102_00117	hoi103_00344	hoi104_00258	hoi108_01546	hoi110_00118	hoi111_00837	hoi112_00118	hoi113_00344	hoi114_00117	hoi115_00118	hoi116_00272	hoi105_00252	hoi107_00252	hoi109_00339	hoi117_00338	hoi106_00246
119	Proline--tRNA ligase	16	Core		hoi102_00118	hoi103_00343	hoi104_00257	hoi108_01545	hoi110_00119	hoi111_00836	hoi112_00119	hoi113_00343	hoi114_00118	hoi115_00119	hoi116_00271	hoi105_00253	hoi107_00253	hoi109_00338	hoi117_00337	hoi106_00247
120	Glucosamine-6-phosphate deaminase 1	16	Core		hoi102_00119	hoi103_00342	hoi104_00256	hoi108_01544	hoi110_00120	hoi111_00835	hoi112_00120	hoi113_00342	hoi114_00119	hoi115_00120	hoi116_00270	hoi105_00254	hoi107_00254	hoi109_00337	hoi117_00336	hoi106_00248
121	hypothetical protein	16	Core		hoi102_00120	hoi103_00341	hoi104_00255	hoi108_01543	hoi110_00121	hoi111_00834	hoi112_00121	hoi113_00341	hoi114_00120	hoi115_00121	hoi116_00269	hoi105_00255	hoi107_00255	hoi109_00336	hoi117_00335	hoi106_00249
122	hypothetical protein	16	Core		hoi102_00121	hoi103_00340	hoi104_00254	hoi108_01542	hoi110_00122	hoi111_00833	hoi112_00122	hoi113_00340	hoi114_00121	hoi115_00122	hoi116_00268	hoi105_00256	hoi107_00256	hoi109_00335	hoi117_00334	hoi106_00250
123	hypothetical protein	16	Core		hoi102_00122	hoi103_00339	hoi104_00253	hoi108_01541	hoi110_00123	hoi111_00832	hoi112_00123	hoi113_00339	hoi114_00122	hoi115_00123	hoi116_00267	hoi105_00257	hoi107_00257	hoi109_00334	hoi117_00333	hoi106_00251
124	ribosome maturation protein RimP	16	Core		hoi102_00123	hoi103_00338	hoi104_00252	hoi108_01540	hoi110_00124	hoi111_00831	hoi112_00124	hoi113_00338	hoi114_00123	hoi115_00124	hoi116_00266	hoi105_00258	hoi107_00258	hoi109_00333	hoi117_00332	hoi106_00252
125	hypothetical protein	16	Core		hoi102_00124	hoi103_00337	hoi104_00251	hoi108_01539	hoi110_00125	hoi111_00830	hoi112_00125	hoi113_00337	hoi114_00124	hoi115_00125	hoi116_00265	hoi105_00259	hoi107_00259	hoi109_00332	hoi117_00331	hoi106_00253
126	Translation initiation factor IF-2	16	Core		hoi102_00125	hoi103_00336	hoi104_00250	hoi108_01538	hoi110_00126	hoi111_00829	hoi112_00126	hoi113_00336	hoi114_00125	hoi115_00126	hoi116_00264	hoi105_00260	hoi107_00260	hoi109_00330	hoi117_00329	hoi106_00255
127	Ribosome-binding factor A	16	Core		hoi102_00126	hoi103_00335	hoi104_00249	hoi108_01537	hoi110_00127	hoi111_00828	hoi112_00127	hoi113_00335	hoi114_00126	hoi115_00127	hoi116_00263	hoi105_00261	hoi107_00261	hoi109_00329	hoi117_00328	hoi106_00256
128	Bifunctional oligoribonuclease and PAP phospha	16	Core		hoi102_00127	hoi103_00334	hoi104_00248	hoi108_01536	hoi110_00128	hoi111_00827	hoi112_00128	hoi113_00334	hoi114_00127	hoi115_00128	hoi116_00262	hoi105_00262	hoi107_00262	hoi109_00328	hoi117_00327	hoi106_00257
129	DNA-damage-inducible protein F	16	Core		hoi102_00128	hoi103_00333	hoi104_00247	hoi108_01535	hoi110_00129	hoi111_00826	hoi112_00129	hoi113_00333	hoi114_00128	hoi115_00129	hoi116_00261	hoi105_00263	hoi107_00263	hoi109_00327	hoi117_00326	hoi106_00258
130	Calcineurin-like phosphoesterase	16	Core		hoi102_00129	hoi103_00332	hoi104_00246	hoi108_01534	hoi110_00130	hoi111_00825	hoi112_00130	hoi113_00332	hoi114_00129	hoi115_00130	hoi116_00260	hoi105_00264	hoi107_00264	hoi109_00326	hoi117_00325	hoi106_00259
131	4'-phosphopantetheinyl transferase Npt	16	Core		hoi102_00130	hoi103_00331	hoi104_00245	hoi108_01533	hoi110_00131	hoi111_00824	hoi112_00131	hoi113_00331	hoi114_00130	hoi115_00131	hoi116_00259	hoi105_00265	hoi107_00265	hoi109_00325	hoi117_00324	hoi106_00260
132	tRNA pseudouridine synthase B	16	Core		hoi102_00131	hoi103_00330	hoi104_00244	hoi108_01532	hoi110_00132	hoi111_00823	hoi112_00132	hoi113_00330	hoi114_00131	hoi115_00132	hoi116_00258	hoi105_00266	hoi107_00266	hoi109_00324	hoi117_00323	hoi106_00261
133	Riboflavin biosynthesis protein RibF	16	Core		hoi102_00132	hoi103_00329	hoi104_00243	hoi108_01531	hoi110_00133	hoi111_00822	hoi112_00133	hoi113_00329	hoi114_00132	hoi115_00133	hoi116_00257	hoi105_00267	hoi107_00267	hoi109_00323	hoi117_00322	hoi106_00262
134	30S ribosomal protein S15	16	Core		hoi102_00133	hoi103_00328	hoi104_00242	hoi108_01530	hoi110_00134	hoi111_00821	hoi112_00134	hoi113_00328	hoi114_00133	hoi115_00134	hoi116_00256	hoi105_00268	hoi107_00268	hoi109_00322	hoi117_00321	hoi106_00263
135	Polyribonucleotide nucleotidyltransferase	16	Core		hoi102_00134	hoi103_00327	hoi104_00241	hoi108_01529	hoi110_00135	hoi111_00820	hoi112_00135	hoi113_00327	hoi114_00134	hoi115_00135	hoi116_00255	hoi105_00269	hoi107_00269	hoi109_00321	hoi117_00320	hoi106_00264
136	Aldose 1-epimerase	16	Core		hoi102_00135	hoi103_00326	hoi104_00240	hoi108_01528	hoi110_00136	hoi111_00819	hoi112_00136	hoi113_00326	hoi114_00135	hoi115_00136	hoi116_00254	hoi105_00270	hoi107_00270	hoi109_00320	hoi117_00319	hoi106_00265
137	Sodium/glucose cotransporter	16	Core		hoi102_00136	hoi103_00325	hoi104_00239	hoi108_01527	hoi110_00137	hoi111_00818	hoi112_00137	hoi113_00325	hoi114_00136	hoi115_00137	hoi116_00253	hoi105_00271	hoi107_00271	hoi109_00319	hoi117_00318	hoi106_00266
138	hypothetical protein	16	Core		hoi102_00137	hoi103_00324	hoi104_00238	hoi108_01526	hoi110_00138	hoi111_00817	hoi112_00138	hoi113_00324	hoi114_00137	hoi115_00138	hoi116_00252	hoi105_00272	hoi107_00272	hoi109_00318	hoi117_00317	hoi106_00267
139	Galactose-1-phosphate uridylyltransferase	16	Core		hoi102_00138	hoi103_00323	hoi104_00237	hoi108_01525	hoi110_00139	hoi111_00816	hoi112_00139	hoi113_00323	hoi114_00138	hoi115_00139	hoi116_00251	hoi105_00273	hoi107_00273	hoi109_00317	hoi117_00316	hoi106_00268
140	Galactokinase	16	Core		hoi102_00139	hoi103_00322	hoi104_00236	hoi108_01524	hoi110_00140	hoi111_00815	hoi112_00140	hoi113_00322	hoi114_00139	hoi115_00140	hoi116_00250	hoi105_00274	hoi107_00274	hoi109_00316	hoi117_00315	hoi106_00269
141	4-hydroxy-tetrahydrodipicolinate reductase	16	Core		hoi102_00140	hoi103_00321	hoi104_00235	hoi108_01523	hoi110_00141	hoi111_00814	hoi112_00141	hoi113_00321	hoi114_00140	hoi115_00141	hoi116_00249	hoi105_00275	hoi107_00275	hoi109_00315	hoi117_00314	hoi106_00270
142	Thymidylate synthase ThyX	16	Core		hoi102_00141	hoi103_00320	hoi104_00234	hoi108_01522	hoi110_00142	hoi111_00813	hoi112_00142	hoi113_00320	hoi114_00141	hoi115_00142	hoi116_00248	hoi105_00276	hoi107_00276	hoi109_00314	hoi117_00313	hoi106_00271
143	4-hydroxy-tetrahydrodipicolinate synthase	16	Core		hoi102_00142	hoi103_00319	hoi104_00233	hoi108_01521	hoi110_00143	hoi111_00812	hoi112_00143	hoi113_00319	hoi114_00142	hoi115_00143	hoi116_00247	hoi105_00277	hoi107_00277	hoi109_00313	hoi117_00312	hoi106_00272
144	Putative ribonuclease J	16	Core		hoi102_00143	hoi103_00318	hoi104_00232	hoi108_01520	hoi110_00144	hoi111_00811	hoi112_00144	hoi113_00318	hoi114_00143	hoi115_00144	hoi116_00246	hoi105_00278	hoi107_00278	hoi109_00312	hoi117_00311	hoi106_00273
145	hypothetical protein	16	Core		hoi102_00144	hoi103_00317	hoi104_00231	hoi108_01519	hoi110_00145	hoi111_00810	hoi112_00145	hoi113_00317	hoi114_00144	hoi115_00145	hoi116_00245	hoi105_00279	hoi107_00279	hoi109_00311	hoi117_00310	hoi106_00274
146	DNA translocase SpoIIIE	16	Core		hoi102_00145	hoi103_00316	hoi104_00230	hoi108_01518	hoi110_00146	hoi111_00809	hoi112_00146	hoi113_00316	hoi114_00145	hoi115_00146	hoi116_00244	hoi105_00280	hoi107_00280	hoi109_00310	hoi117_00309	hoi106_00275
147	Inner membrane protein alx	16	Core		hoi102_00146	hoi103_00315	hoi104_00229	hoi108_01517	hoi110_00147	hoi111_00808	hoi112_00147	hoi113_00315	hoi114_00146	hoi115_00147	hoi116_00243	hoi105_00281	hoi107_00281	hoi109_00309	hoi117_00308	hoi106_00276
148	YciI-like protein	16	Core		hoi102_00147	hoi103_00314	hoi104_00228	hoi108_01516	hoi110_00148	hoi111_00807	hoi112_00148	hoi113_00314	hoi114_00147	hoi115_00148	hoi116_00242	hoi105_00282	hoi107_00282	hoi109_00308	hoi117_00307	hoi106_00277
149	Putative CDP-diacylglycerol--glycerol-3-phosphat	16	Core		hoi102_00148	hoi103_00313	hoi104_00227	hoi108_01515	hoi110_00149	hoi111_00806	hoi112_00149	hoi113_00313	hoi114_00148	hoi115_00149	hoi116_00241	hoi105_00283	hoi107_00283	hoi109_00307	hoi117_00306	hoi106_00278
150	Putative competence-damage inducible protein	16	Core	GI3	hoi102_00149	hoi103_00312	hoi104_00226	hoi108_01514	hoi110_00150	hoi111_00805	hoi112_00150	hoi113_00312	hoi114_00149	hoi115_00150	hoi116_00240	hoi105_00284	hoi107_00284	hoi109_00306	hoi117_00305	hoi106_00279
151	Transcriptional regulator ClgR	16	Core	GI3	hoi102_00150	hoi103_00311	hoi104_00225	hoi108_01513	hoi110_00151	hoi111_00804	hoi112_00151	hoi113_00311	hoi114_00150	hoi115_00151	hoi116_00239	hoi105_00285	hoi107_00285	hoi109_00305	hoi117_00304	hoi106_00280
152	PspA/IM30 family protein	16	Core	GI3	hoi102_00151	hoi103_00310	hoi104_00224	hoi108_01512	hoi110_00152	hoi111_00803	hoi112_00152	hoi113_00310	hoi114_00151	hoi115_00152	hoi116_00238	hoi105_00286	hoi107_00286	hoi109_00304	hoi117_00303	hoi106_00281
153	Energy-coupling factor transporter transmembra	16	Core	GI3	hoi102_00152	hoi103_00309	hoi104_00223	hoi108_01511	hoi110_00153	hoi111_00802	hoi112_00153	hoi113_00309	hoi114_00152	hoi115_00153	hoi116_00237	hoi105_00287	hoi107_00287	hoi109_00303	hoi117_00302	hoi106_00282
154	Biotin transport ATP-binding protein BioM	16	Core	GI3	hoi102_00153	hoi103_00308	hoi104_00222	hoi108_01510	hoi110_00154	hoi111_00801	hoi112_00154	hoi113_00308	hoi114_00153	hoi115_00154	hoi116_00236	hoi105_00288	hoi107_00288	hoi109_00302	hoi117_00301	hoi106_00283
155	Biotin transporter BioY	16	Core	GI3	hoi102_00154	hoi103_00307	hoi104_00221	hoi108_01509	hoi110_00155	hoi111_00800	hoi112_00155	hoi113_00307	hoi114_00154	hoi115_00155	hoi116_00235	hoi105_00289	hoi107_00289	hoi109_00301	hoi117_00300	hoi106_00284
156	hypothetical protein	16	Core	GI3	hoi102_00155	hoi103_00306	hoi104_00220	hoi108_01508	hoi110_00156	hoi111_00799	hoi112_00156	hoi113_00306	hoi114_00155	hoi115_00156	hoi116_00234	hoi105_00290	hoi107_00290	hoi109_00300	hoi117_00299	hoi106_00285
157	recombinase A	16	Core	GI3	hoi102_00156	hoi103_00305	hoi104_00219	hoi108_01507	hoi110_00157	hoi111_00798	hoi112_00157	hoi113_00305	hoi114_00156	hoi115_00157	hoi116_00233	hoi105_00291	hoi107_00291	hoi109_00299	hoi117_00298	hoi106_00286

158	Regulatory protein RecX	16	Core	hoi102_00157	hoi103_00304	hoi104_00218	hoi108_01506	hoi110_00158	hoi111_00797	hoi112_00158	hoi113_00304	hoi114_00157	hoi115_00158	hoi116_00232	hoi105_00292	hoi107_00292	hoi109_00298	hoi117_00297	hoi106_00287
159	(Dimethylallyl)adenosine tRNA methylthiotransfe	16	Core	hoi102_00158	hoi103_00303	hoi104_00217	hoi108_01505	hoi110_00159	hoi111_00796	hoi112_00159	hoi113_00303	hoi114_00158	hoi115_00159	hoi116_00231	hoi105_00293	hoi107_00293	hoi109_00297	hoi117_00296	hoi106_00288
160	hypothetical protein	16	Core	hoi102_00159	hoi103_00302	hoi104_00216	hoi108_01504	hoi110_00160	hoi111_00795	hoi112_00160	hoi113_00302	hoi114_00159	hoi115_00160	hoi116_00230	hoi105_00294	hoi107_00294	hoi109_00296	hoi117_00295	hoi106_00289
161	hypothetical protein	16	Core	hoi102_00160	hoi103_00301	hoi104_00215	hoi108_01503	hoi110_00161	hoi111_00794	hoi112_00161	hoi113_00301	hoi114_00160	hoi115_00161	hoi116_00229	hoi105_00295	hoi107_00295	hoi109_00295	hoi117_00294	hoi106_00290
162	hypothetical protein	16	Core	hoi102_00161	hoi103_00300	hoi104_00214	hoi108_01502	hoi110_00162	hoi111_00793	hoi112_00162	hoi113_00300	hoi114_00161	hoi115_00162	hoi116_00228	hoi105_00296	hoi107_00296	hoi109_00294	hoi117_00293	hoi106_00291
163	hypothetical protein	16	Core	hoi102_00162	hoi103_00299	hoi104_00213	hoi108_00779	hoi110_00163	hoi111_00792	hoi112_00163	hoi113_00299	hoi114_00162	hoi115_00163	hoi116_00227	hoi105_00297	hoi107_00297	hoi109_00293	hoi117_00292	hoi106_00292
164	tRNA dimethylallyltransferase	16	Core	hoi102_00163	hoi103_00298	hoi104_00212	hoi108_00780	hoi110_00164	hoi111_00791	hoi112_00164	hoi113_00298	hoi114_00163	hoi115_00164	hoi116_00226	hoi105_00298	hoi107_00298	hoi109_00292	hoi117_00291	hoi106_00293
165	hypothetical protein	16	Core	hoi102_00164	hoi103_00297	hoi104_00211	hoi108_00781	hoi110_00165	hoi111_00790	hoi112_00165	hoi113_00297	hoi114_00164	hoi115_00165	hoi116_00225	hoi105_00299	hoi107_00299	hoi109_00291	hoi117_00290	hoi106_00294
166	hypothetical protein	16	Core	hoi102_00165	hoi103_00296	hoi104_00210	hoi108_00782	hoi110_00166	hoi111_00789	hoi112_00166	hoi113_00296	hoi114_00165	hoi115_00166	hoi116_00224	hoi105_00300	hoi107_00300	hoi109_00290	hoi117_00289	hoi106_00295
167	FeoA domain protein	16	Core	hoi102_00166	hoi103_00295	hoi104_00209	hoi108_00783	hoi110_00167	hoi111_00788	hoi112_00167	hoi113_00295	hoi114_00166	hoi115_00167	hoi116_00223	hoi105_00301	hoi107_00301	hoi109_00289	hoi117_00288	hoi106_00296
168	Ferrous iron transport protein B	16	Core	hoi102_00167	hoi103_00294	hoi104_00208	hoi108_00784	hoi110_00168	hoi111_00787	hoi112_00168	hoi113_00294	hoi114_00167	hoi115_00168	hoi116_00222	hoi105_00302	hoi107_00302	hoi109_00288	hoi117_00287	hoi106_00297
169	hypothetical protein	16	Core	hoi102_00168	hoi103_00293	hoi104_00207	hoi108_00785	hoi110_00169	hoi111_00786	hoi112_00169	hoi113_00293	hoi114_00168	hoi115_00169	hoi116_00221	hoi105_00303	hoi107_00303	hoi109_00287	hoi117_00286	hoi106_00298
170	GTPase HflX	16	Core	hoi102_00169	hoi103_00292	hoi104_00206	hoi108_00786	hoi110_00170	hoi111_00785	hoi112_00170	hoi113_00292	hoi114_00169	hoi115_00170	hoi116_00220	hoi105_00304	hoi107_00304	hoi109_00286	hoi117_00285	hoi106_00299
171	Putative pyrimidine permease RutG	16	Core	hoi102_00170	hoi103_00291	hoi104_00205	hoi108_00787	hoi110_00171	hoi111_00784	hoi112_00171	hoi113_00291	hoi114_00170	hoi115_00171	hoi116_00219	hoi105_00305	hoi107_00305	hoi109_00285	hoi117_00284	hoi106_00300
172	Phosphocarrier protein HPr	16	Core	hoi102_00171	hoi103_00290	hoi104_00204	hoi108_00788	hoi110_00172	hoi111_00783	hoi112_00172	hoi113_00290	hoi114_00171	hoi115_00172	hoi116_00218	hoi105_00306	hoi107_00306	hoi109_00284	hoi117_00283	hoi106_00301
173	PTS system fructose-specific EIIABC component	16	Core	hoi102_00172	hoi103_00289	hoi104_00203	hoi108_00789	hoi110_00173	hoi111_00782	hoi112_00173	hoi113_00289	hoi114_00172	hoi115_00173	hoi116_00217	hoi105_00307	hoi107_00307	hoi109_00283	hoi117_00282	hoi106_00302
174	Putative phosphofructokinase PfkB	16	Core	hoi102_00173	hoi103_00288	hoi104_00202	hoi108_00790	hoi110_00174	hoi111_00781	hoi112_00174	hoi113_00288	hoi114_00173	hoi115_00174	hoi116_00216	hoi105_00308	hoi107_00308	hoi109_00282	hoi117_00281	hoi106_00303
175	Glycerol-3-phosphate regulon repressor	16	Core	hoi102_00174	hoi103_00287	hoi104_00201	hoi108_00791	hoi110_00175	hoi111_00780	hoi112_00175	hoi113_00287	hoi114_00174	hoi115_00175	hoi116_00215	hoi105_00309	hoi107_00309	hoi109_00281	hoi117_00280	hoi106_00304
176	Phosphoenolpyruvate-protein phosphotransfera	16	Core	hoi102_00175	hoi103_00286	hoi104_00200	hoi108_00792	hoi110_00176	hoi111_00779	hoi112_00176	hoi113_00286	hoi114_00175	hoi115_00176	hoi116_00214	hoi105_00310	hoi107_00310	hoi109_00280	hoi117_00279	hoi106_00305
177	1-phosphofructokinase	16	Core	hoi102_00176	hoi103_00285	hoi104_00199	hoi108_00793	hoi110_00177	hoi111_00778	hoi112_00177	hoi113_00285	hoi114_00176	hoi115_00177	hoi116_00213	hoi105_00311	hoi107_00311	hoi109_00279	hoi117_00278	hoi106_00306
178	HTH-type transcriptional repressor GlcR	16	Core	hoi102_00177	hoi103_00284	hoi104_00198	hoi108_00794	hoi110_00178	hoi111_00777	hoi112_00178	hoi113_00284	hoi114_00177	hoi115_00178	hoi116_00212	hoi105_00312	hoi107_00312	hoi109_00278	hoi117_00277	hoi106_00307
179	LexA repressor	16	Core	hoi102_00178	hoi103_00283	hoi104_00197	hoi108_00795	hoi110_00179	hoi111_00776	hoi112_00179	hoi113_00283	hoi114_00178	hoi115_00179	hoi116_00211	hoi105_00313	hoi107_00313	hoi109_00277	hoi117_00276	hoi106_00308
180	hypothetical protein	8	Accessory	hoi102_00179	hoi103_00282			hoi110_00180	hoi111_00775	hoi112_00180	hoi113_00282	hoi114_00179		hoi116_00210					
181	Transcriptional repressor NrdR	16	Core	hoi102_00180	hoi103_00281	hoi104_00196	hoi108_00796	hoi110_00181	hoi111_00774	hoi112_00181	hoi113_00281	hoi114_00180	hoi115_00180	hoi116_00209	hoi105_00314	hoi107_00314	hoi109_00276	hoi117_00275	hoi106_00309
182	ATP-dependent RNA helicase HrpB	16	Core	hoi102_00181	hoi103_00280	hoi104_00195	hoi108_00797	hoi110_00182	hoi111_00773	hoi112_00182	hoi113_00280	hoi114_00181	hoi115_00181	hoi116_00208	hoi105_00315	hoi107_00315	hoi109_00275	hoi117_00274	hoi106_00310
183	Hydrogen peroxide-inducible genes activator	16	Core	hoi102_00182	hoi103_00279	hoi104_00194	hoi108_00798	hoi110_00183	hoi111_00772	hoi112_00183	hoi113_00279	hoi114_00182	hoi115_00182	hoi116_00207	hoi105_00316	hoi107_00316	hoi109_00274	hoi117_00273	hoi106_00311
184	Alkyl hydroperoxide reductase subunit C	16	Core	hoi102_00183	hoi103_00278	hoi104_00193	hoi108_00799	hoi110_00184	hoi111_00771	hoi112_00184	hoi113_00278	hoi114_00183	hoi115_00183	hoi116_00206	hoi105_00317	hoi107_00317	hoi109_00273	hoi117_00272	hoi106_00312
185	Alkyl hydroperoxide reductase AhpD	16	Core	hoi102_00184	hoi103_00277	hoi104_00192	hoi108_00800	hoi110_00185	hoi111_00770	hoi112_00185	hoi113_00277	hoi114_00184	hoi115_00184	hoi116_00205	hoi105_00318	hoi107_00318	hoi109_00272	hoi117_00271	hoi106_00313
186	ski2-like helicase	16	Core	hoi102_00185	hoi103_00276	hoi104_00191	hoi108_00801	hoi110_00186	hoi111_00769	hoi112_00186	hoi113_00276	hoi114_00185	hoi115_00185	hoi116_00204	hoi105_00319	hoi107_00319	hoi109_00271	hoi117_00270	hoi106_00314
187	PAC2 family protein	16	Core	hoi102_00186	hoi103_00275	hoi104_00190	hoi108_00802	hoi110_00187	hoi111_00768	hoi112_00187	hoi113_00275	hoi114_00186	hoi115_00186	hoi116_00203	hoi105_00320	hoi107_00320	hoi109_00270	hoi117_00269	hoi106_00315
188	hypothetical protein	16	Core	hoi102_00187	hoi103_00274	hoi104_00189	hoi108_00803	hoi110_00188	hoi111_00767	hoi112_00188	hoi113_00274	hoi114_00187	hoi115_00187	hoi116_00202	hoi105_00321	hoi107_00321	hoi109_00269	hoi117_00268	hoi106_00316
189	UDP-glucose 4-epimerase	16	Core	hoi102_00188	hoi103_00273	hoi104_00188	hoi108_00804	hoi110_00189	hoi111_00766	hoi112_00189	hoi113_00273	hoi114_00188	hoi115_00188	hoi116_00201	hoi105_00322	hoi107_00322	hoi109_00268	hoi117_00267	hoi106_00317
190	Diphtheria toxin repressor	16	Core	hoi102_00189	hoi103_00272	hoi104_00187	hoi108_00805	hoi110_00190	hoi111_00765	hoi112_00190	hoi113_00272	hoi114_00189	hoi115_00189	hoi116_00200	hoi105_00323	hoi107_00323	hoi109_00267	hoi117_00266	hoi106_00318
191	RNA polymerase sigma factor SigB	16	Core	hoi102_00190	hoi103_00271	hoi104_00186	hoi108_00806	hoi110_00191	hoi111_00764	hoi112_00191	hoi113_00271	hoi114_00190	hoi115_00190	hoi116_00199	hoi105_00324	hoi107_00324	hoi109_00266	hoi117_00265	hoi106_00319
192	D-tyrosyl-tRNA(Tyr) deacylase	16	Core	hoi102_00191	hoi103_00270	hoi104_00185	hoi108_00807	hoi110_00192	hoi111_00763	hoi112_00192	hoi113_00270	hoi114_00191	hoi115_00191	hoi116_00198	hoi105_00325	hoi107_00325	hoi109_00265	hoi117_00264	hoi106_00320
193	Ribosomal RNA small subunit methyltransferase	16	Core	hoi102_00192	hoi103_00269	hoi104_00184	hoi108_00808	hoi110_00193	hoi111_00762	hoi112_00193	hoi113_00269	hoi114_00192	hoi115_00192	hoi116_00197	hoi105_00326	hoi107_00326	hoi109_00264	hoi117_00263	hoi106_00321
194	hypothetical protein	16	Core	hoi102_00193	hoi103_00268	hoi104_00183	hoi108_00809	hoi110_00194	hoi111_00761	hoi112_00194	hoi113_00268	hoi114_00193	hoi115_00193	hoi116_00196	hoi105_00327	hoi107_00327	hoi109_00263	hoi117_00262	hoi106_00322
195	hypothetical protein	16	Core	hoi102_00194	hoi103_00267	hoi104_00182	hoi108_00810	hoi110_00195	hoi111_00760	hoi112_00195	hoi113_00267	hoi114_00194	hoi115_00194	hoi116_00195	hoi105_00328	hoi107_00328	hoi109_00262	hoi117_00261	hoi106_00323
196	Type III restriction enzyme, res subunit	16	Core	hoi102_00195	hoi103_00266	hoi104_00181	hoi108_00811	hoi110_00196	hoi111_00759	hoi112_00196	hoi113_00266	hoi114_00195	hoi115_00195	hoi116_00194	hoi105_00329	hoi107_00329	hoi109_00261	hoi117_00260	hoi106_00324
197	hypothetical protein	16	Core	hoi102_00196	hoi103_00265	hoi104_00180	hoi108_00812	hoi110_00197	hoi111_00758	hoi112_00197	hoi113_00265	hoi114_00196	hoi115_00196	hoi116_00193	hoi105_00330	hoi107_00330	hoi109_00260	hoi117_00259	hoi106_00325
198	hypothetical protein	16	Core	hoi102_00197	hoi103_00264	hoi104_00179	hoi108_00813	hoi110_00198	hoi111_00757	hoi112_00198	hoi113_00264	hoi114_00197	hoi115_00197	hoi116_00192	hoi105_00331	hoi107_00331	hoi109_00259	hoi117_00258	hoi106_00326
199	RNA polymerase sigma factor SigA	16	Core	hoi102_00198	hoi103_00263	hoi104_00178	hoi108_00814	hoi110_00199	hoi111_00756	hoi112_00199	hoi113_00263	hoi114_00199	hoi115_00198	hoi116_00191	hoi105_00332	hoi107_00332	hoi109_00258	hoi117_00257	hoi106_00327
200	Polyphosphate glucokinase	16	Core	hoi102_00199	hoi103_00262	hoi104_00177	hoi108_00815	hoi110_00200	hoi111_00755	hoi112_00200	hoi113_00262	hoi114_00200	hoi115_00199	hoi116_00190	hoi105_00333	hoi107_00333	hoi109_00257	hoi117_00256	hoi106_00328
201	Inositol-1-monophosphatase SuhB	16	Core	hoi102_00200	hoi103_00261	hoi104_00176	hoi108_00816	hoi110_00201	hoi111_00754	hoi112_00201	hoi113_00261	hoi114_00201	hoi115_00200	hoi116_00189	hoi105_00334	hoi107_00334	hoi109_00256	hoi117_00255	hoi106_00329
202	hypothetical protein	16	Core	hoi102_00201	hoi103_00260	hoi104_00175	hoi108_00817	hoi110_00202	hoi111_00753	hoi112_00202	hoi113_00260	hoi114_00202	hoi115_00201	hoi116_00188	hoi105_00335	hoi107_00335	hoi109_00255	hoi117_00254	hoi106_00330
203	hypothetical protein	16	Core	hoi102_00202	hoi103_00259	hoi104_00174	hoi108_00818	hoi110_00203	hoi111_00752	hoi112_00203	hoi113_00259	hoi114_00203	hoi115_00202	hoi116_00187	hoi105_00336	hoi107_00336	hoi109_00254	hoi117_00253	hoi106_00331
204	Deoxyuridine 5'-triphosphate nucleotidohydrolas	16	Core	hoi102_00203	hoi103_00258	hoi104_00173	hoi108_00819	hoi110_00204	hoi111_00751	hoi112_00204	hoi113_00258	hoi114_00204	hoi115_00203	hoi116_00186	hoi105_00337	hoi107_00337	hoi109_00253	hoi117_00252	hoi106_00332
205	hypothetical protein	16	Core	hoi102_00204	hoi103_00257	hoi104_00172	hoi108_00820	hoi110_00205	hoi111_00750	hoi112_00205	hoi113_00257	hoi114_00205	hoi115_00204	hoi116_00185	hoi105_00338	hoi107_00338	hoi109_00252	hoi117_00251	hoi106_00333
206	putative RNA methyltransferase/cg2084	16	Core	hoi102_00205	hoi103_00256	hoi104_00171	hoi108_00821	hoi110_00206	hoi111_00749	hoi112_00206	hoi113_00256	hoi114_00206	hoi115_00205	hoi116_00184	hoi105_00339	hoi107_00339	hoi109_00251	hoi117_00250	hoi106_00334
207	1-deoxy-D-xylulose-5-phosphate synthase	16	Core	hoi102_00206	hoi103_00255	hoi104_00170	hoi108_00822	hoi110_00207	hoi111_00748	hoi112_00207	hoi113_00255	hoi114_00207	hoi115_00206	hoi116_00183	hoi105_00340	hoi107_00340	hoi109_00250	hoi117_00249	hoi106_00335
208	Formate/nitrite transporter	16	Core	hoi102_00207	hoi103_00254	hoi104_00169	hoi108_00823	hoi110_00208	hoi111_00747	hoi112_00208	hoi113_00254	hoi114_00208	hoi115_00207	hoi116_00182	hoi105_00341	hoi107_00341	hoi109_00249	hoi117_00248	hoi106_00336
209	Ribonuclease D	16	Core	hoi102_00208	hoi103_00253	hoi104_00168	hoi108_00824	hoi110_00209	hoi111_00746	hoi112_00209	hoi113_00253	hoi114_00209	hoi115_00208	hoi116_00181	hoi105_00342	hoi107_00342	hoi109_00248	hoi117_00247	hoi106_00337
210	hypothetical protein	16	Core	hoi102_00209	hoi103_00252	hoi104_00167	hoi108_00825	hoi110_00210	hoi111_00745	hoi112_00210	hoi113_00252	hoi114_00210	hoi115_00209	hoi116_00180	hoi105_00343	hoi107_00343	hoi109_00247	hoi117_00246	hoi106_00338
211	putative heme peroxidase	16	Core	hoi102_00210	hoi103_00251	hoi104_00166	hoi108_00826	hoi110_00211	hoi111_00744	hoi112_00211	hoi113_00251	hoi114_00211	hoi115_00210	hoi116_00179	hoi105_00344	hoi107_00344	hoi109_00246	hoi117_00245	hoi106_00339
212	Peptide methionine sulfoxide reductase MsrB	16	Core	hoi102_00211	hoi103_00250	hoi104_00165	hoi108_00827	hoi110_00212	hoi111_00743	hoi112_00212	hoi113_00250	hoi114_00212	hoi115_00211	hoi116_00178	hoi105_00345	hoi107_00345	hoi109_00245	hoi117_00244	hoi106_00340
213	Alpha-(1->3)-arabinofuranosyltransferase	16	Core	hoi102_00212	hoi103_00249	hoi104_00164	hoi108_00828	hoi110_00213	hoi111_00742	hoi112_00213	hoi113_00249	hoi114_00213	hoi115_00212	hoi116_00177	hoi105_00346	hoi107_00346	hoi109_00244	hoi117_00243	hoi106_00341
214	5-amino-6-(5-phosphoribosylamino)uracil reduct	12	Accessory	hoi102_00213	hoi103_00248	hoi104_00163	hoi108_00829	hoi110_00214	hoi111_00741	hoi112_00214	hoi113_00248	hoi114_00214	hoi115_00213	hoi116_00176					hoi106_00342
215	hypothetical protein	16	Core	hoi102_00221	hoi103_00240	hoi104_00155	hoi108_00837	hoi110_00222	hoi111_00733	hoi112_00222	hoi113_00240	hoi114_00222	hoi115_00221	hoi116_00168	hoi105_00354	hoi107_00354	hoi109_00236	hoi117_00235	hoi106_00350
216	hypothetical protein	16	Core	hoi102_00222	hoi103_00239	hoi104_00154	hoi108_00838	hoi110_00223	hoi111_00732	hoi112_00223	hoi113_00239	hoi114_00223	hoi115_00222	hoi116_00167	hoi105_00355	hoi107_00355	hoi109_00235	hoi117_00234	hoi106_00351
217	putative deferrochelatase/peroxidase EfeN precu	16	Core	hoi102_00223	hoi103_00238	hoi104_00153	hoi108_00839	hoi110_00224	hoi111_00731	hoi112_00224	hoi113_00238	hoi114_00224	hoi115_00223	hoi116_00166	hoi105_00356	hoi107_00356	hoi109_00234	hoi117_00233	hoi106_00352
218	Threonine--tRNA ligase	16	Core	hoi102_00224	hoi103_00237	hoi104_00152	hoi108_00840	hoi110_00225	hoi111_00730	hoi112_00225	hoi113_00237	hoi114_00225	hoi115_00224	hoi116_00165	hoi105_00357	hoi107_00357	hoi109_00233	hoi117_00232	hoi106_00353
219	AP-4-A phosphorylase	16	Core	hoi102_00225	hoi103_00236	hoi104_00151	hoi108_00841	hoi110_00226	hoi111_00729	hoi112_00226	hoi113_00236	hoi114_00226	hoi115_00225	hoi116_00164	hoi105_00358	hoi107_00358	hoi109_00232	hoi117_00231	hoi106_00354
220	CDP-diacylglycerol--inositol 3-phosphatidyltransf	16	Core	hoi102_00226	hoi103_00235	hoi104_00150	hoi108_00842	hoi110_00227	hoi111_00728	hoi112_00227	hoi113_00235	hoi114_00227	hoi115_00226	hoi116_00163	hoi105_00359	hoi107_00359	hoi109_00231	hoi117_00230	hoi106_00355
221	Phosphatidylinositol mannoside acyltransferase	16	Core	hoi102_00227	hoi103_00234	hoi104_00149	hoi108_00843	hoi110_00228	hoi111_00727	hoi112_00228	hoi113_00234	hoi114_00228	hoi115_00227	hoi116_00162	hoi105_00360	hoi107_00360	hoi109_00230	hoi117_00229	hoi106_00356
222	GDP-mannose-dependent alpha-(1-2)-phosphati	16	Core	hoi102_00228	hoi103_00233	hoi104_00148	hoi108_00844	hoi110_00229	hoi111_00726	hoi112_00229	hoi113_00233	hoi114_00229	hoi115_00228	hoi116_00161	hoi105_00361	hoi107_00361	hoi109_00229	hoi117_00228	hoi106_00357
223	hypothetical protein	16	Core	hoi102_00229	hoi103_00232	hoi104_00147	hoi108_00845	hoi110_00230	hoi111_00725	hoi112_00230	hoi113_00232	hoi114_00230	hoi115_00229	hoi116_00160	hoi105_00362	hoi107_00362	hoi109_00228	hoi117_00227	hoi106_00358
224	Pyridoxal biosynthesis lyase PdxS	16	Core	hoi102_00230	hoi103_00231	hoi104_00146	hoi108_00846	hoi110_00231	hoi111_00724	hoi112_00231	hoi113_00231	hoi114_00231	hoi115_00230	hoi116_00159	hoi105_00363	hoi107_00363	hoi109_00227	hoi117_00226	hoi106_00359
225	Acyl-CoA thioesterase 2	16	Core	hoi102_00231	hoi103_00230	hoi104_00145	hoi108_00847	hoi110_00232	hoi111_00723	hoi112_00232	hoi113_00230	hoi114_00232	hoi115_00231	hoi116_00158	hoi105_00364	hoi107_00364	hoi109_00226	hoi117_00225	hoi106_00360
226	putative transcriptional regulatory protein/MSM	16	Core	hoi102_00232	hoi103_00229	hoi104_00144	hoi108_00848	hoi110_00233	hoi111_00722	hoi112_00233	hoi113_00229	hoi114_00233	hoi115_00232	hoi116_00157	hoi105_00365	hoi107_00365	hoi109_00225	hoi117_00224	hoi106_00361
227	Crossover junction endodeoxyribonuclease RuvC	16	Core	hoi102_00233	hoi103_00228	hoi104_00143	hoi108_00849	hoi110_00234	hoi111_00721	hoi112_00234	hoi113_00228	hoi114_00234	hoi115_00233	hoi116_00156	hoi105_00366	hoi107_00366	hoi109_00224	hoi117_00223	hoi106_00362
228	Holliday junction ATP-dependent DNA helicase R	16	Core	hoi102_00234	hoi103_00227	hoi104_00142	hoi108_00850	hoi110_00235	hoi111_00720	hoi112_00235	hoi113_00227	hoi114_00235	hoi115_00234	hoi116_00155	hoi105_00367	hoi107_00367	hoi109_00223	hoi117_00222	hoi106_00363
229	Holliday junction ATP-dependent DNA helicase R	16	Core	hoi102_00235	hoi103_00226	hoi104_00141	hoi108_00851	hoi110_00236	hoi111_00719	hoi112_00236	hoi113_00226	hoi114_00236	hoi115_00235	hoi116_00154	hoi105_00368	hoi107_00368	hoi109_00222	hoi117_00221	hoi106_00364
230	preprotein translocase subunit YajC	16	Core	hoi102_00236	hoi103_00225	hoi104_00140	hoi108_00852	hoi110_00237	hoi111_00718	hoi112_00237	hoi113_00225	hoi114_00237	hoi115_00236	hoi116_00153	hoi105_00369	hoi107_00369	hoi109_00221	hoi117_00220	hoi106_00365
231	preprotein translocase subunit SecD	16	Core	hoi102_00237	hoi103_00224	hoi104_00139	hoi108_00853	hoi110_00238	hoi111_00717	hoi112_00238	hoi113_00224	hoi114_00238	hoi115_00237	hoi116_00152	hoi105_00370	hoi107_00370	hoi109_00220	hoi117_00219	hoi106_00366
232	preprotein translocase subunit SecF	16	Core	hoi102_00238	hoi103_00223	hoi104_00138	hoi108_00854	hoi110_00239	hoi111_00716	hoi112_00239	hoi113_00223	hoi114_00239	hoi115_00238	hoi116_00151	hoi105_00371	hoi107_00371	hoi109_00219	hoi117_00218	hoi106_00367
233	Bacterial extracellular solute-binding proteins, fa	16	Core	hoi102_00239	hoi103_00222	hoi104_00137	hoi108_00855	hoi110_00240	hoi111_00715	hoi112_00240	hoi113_00222	hoi114_00240	hoi115_00239	hoi116_00150	hoi105_00372	hoi107_00372	hoi109_00218	hoi117_00217	hoi106_00368
234	Adenine phosphoribosyltransferase	16	Core	hoi102_00240	hoi103_00221	hoi104_00136	hoi108_00856	hoi110_00241	hoi111_00714	hoi112_00241	hoi113_00221	hoi114_00241	hoi115_00240	hoi116_00149	hoi105_00373	hoi107_00373	hoi109_00217	hoi117_00216	hoi106_00369
235	Bifunctional (p)ppGpp synthase/hydrolase relA	16	Core	hoi102_00241	hoi103_00220	hoi104_00135	hoi108_00857	hoi110_00242	hoi111_00713	hoi112_00242	hoi113_00220	hoi114_00242	hoi115_00241	hoi116_00148	hoi105_00374	hoi107_00374	hoi109_00216	hoi117_00215	hoi106_00370
236	Peptidyl-prolyl cis-trans isomerase B	16	Core	hoi102_00242	hoi103_00219	hoi104_00134	hoi108_00858	hoi110_00243	hoi111_00712	hoi112_00243	hoi113_00219	hoi114_00243	hoi115_00242	hoi116_00147	hoi105_00375	hoi107_00375	hoi109_00215	hoi117_00214	hoi106_00371
237	putative metallo-hydrolase	16	Core	hoi102_00243	hoi103_00218	hoi104_00133	hoi108_00859	hoi110_00244	hoi111_00711	hoi112_00244	hoi113_00218	hoi114_00244	hoi115_00243	hoi116_00146	hoi105_00376	hoi107_00376	hoi109_00214	hoi117_00213	hoi106_00372

238	Histidine--tRNA ligase	16	Core		hoi102_00244	hoi103_00217	hoi104_00132	hoi108_00860	hoi110_00245	hoi111_00710	hoi112_00245	hoi113_00217	hoi114_00245	hoi115_00244	hoi116_00145	hoi105_00377	hoi107_00377	hoi109_00213	hoi117_00212	hoi106_00373
239	L-serine dehydratase 1	16	Core		hoi102_00245	hoi103_00216	hoi104_00131	hoi108_00861	hoi110_00246	hoi111_00709	hoi112_00246	hoi113_00216	hoi114_00246	hoi115_00245	hoi116_00144	hoi105_00378	hoi107_00378	hoi109_00212	hoi117_00211	hoi106_00374
240	FMN reductase (NADPH)	16	Core		hoi102_00246	hoi103_00215	hoi104_00130	hoi108_00862	hoi110_00247	hoi111_00708	hoi112_00247	hoi113_00215	hoi114_00247	hoi115_00246	hoi116_00143	hoi105_00379	hoi107_00379	hoi109_00211	hoi117_00210	hoi106_00375
241	hypothetical protein	16	Core		hoi102_00247	hoi103_00214	hoi104_00129	hoi108_00863	hoi110_00248	hoi111_00707	hoi112_00248	hoi113_00214	hoi114_00248	hoi115_00247	hoi116_00142	hoi105_00380	hoi107_00380	hoi109_00210	hoi117_00209	hoi106_00376
242	Putative neutral zinc metallopeptidase	16	Core		hoi102_00248	hoi103_00213	hoi104_00128	hoi108_00864	hoi110_00249	hoi111_00706	hoi112_00249	hoi113_00213	hoi114_00249	hoi115_00248	hoi116_00141	hoi105_00381	hoi107_00381	hoi109_00209	hoi117_00208	hoi106_00377
243	Aspartate--tRNA ligase	16	Core		hoi102_00249	hoi103_00212	hoi104_00127	hoi108_00865	hoi110_00250	hoi111_00705	hoi112_00250	hoi113_00212	hoi114_00250	hoi115_00249	hoi116_00140	hoi105_00382	hoi107_00382	hoi109_00208	hoi117_00207	hoi106_00378
244	Phosphotransferase enzyme family protein	16	Core		hoi102_00250	hoi103_00211	hoi104_00126	hoi108_00866	hoi110_00251	hoi111_00704	hoi112_00251	hoi113_00211	hoi114_00251	hoi115_00250	hoi116_00139	hoi105_00383	hoi107_00383	hoi109_00207	hoi117_00206	hoi106_00379
245	Replication-associated recombination protein A	16	Core		hoi102_00251	hoi103_00210	hoi104_00125	hoi108_00867	hoi110_00252	hoi111_00703	hoi112_00252	hoi113_00210	hoi114_00252	hoi115_00251	hoi116_00138	hoi105_00384	hoi107_00384	hoi109_00206	hoi117_00205	hoi106_00380
246	Alanine--tRNA ligase	16	Core		hoi102_00252	hoi103_00209	hoi104_00124	hoi108_00868	hoi110_00253	hoi111_00702	hoi112_00253	hoi113_00209	hoi114_00253	hoi115_00252	hoi116_00137	hoi105_00385	hoi107_00385	hoi109_00205	hoi117_00204	hoi106_00381
247	Putative Holliday junction resolvase	16	Core		hoi102_00253	hoi103_00208	hoi104_00123	hoi108_00869	hoi110_00254	hoi111_00701	hoi112_00254	hoi113_00208	hoi114_00254	hoi115_00253	hoi116_00136	hoi105_00386	hoi107_00386	hoi109_00204	hoi117_00203	hoi106_00382
248	putative aminodeoxychorismate lyase	15	Accessory		hoi102_00254	hoi103_00207	hoi104_00122	hoi108_00870	hoi110_00255	hoi111_00700	hoi112_00255	hoi113_00207	hoi114_00255	hoi115_00254	hoi116_00135	hoi105_00387	hoi107_00387	hoi109_00203	hoi117_00202	
249	Shikimate dehydrogenase	16	Core		hoi102_00255	hoi103_00206	hoi104_00121	hoi108_00871	hoi110_00256	hoi111_00699	hoi112_00256	hoi113_00206	hoi114_00256	hoi115_00255	hoi116_00134	hoi105_00388	hoi107_00388	hoi109_00202	hoi117_00201	hoi106_00384
250	hypothetical protein	16	Core		hoi102_00256	hoi103_00205	hoi104_00120	hoi108_00872	hoi110_00257	hoi111_00698	hoi112_00257	hoi113_00205	hoi114_00257	hoi115_00256	hoi116_00133	hoi105_00389	hoi107_00389	hoi109_00201	hoi117_00200	hoi106_00385
251	Chorismate synthase	16	Core		hoi102_00257	hoi103_00204	hoi104_00119	hoi108_00873	hoi110_00258	hoi111_00697	hoi112_00258	hoi113_00204	hoi114_00258	hoi115_00257	hoi116_00132	hoi105_00390	hoi107_00390	hoi109_00200	hoi117_00199	hoi106_00386
252	Shikimate kinase	16	Core		hoi102_00258	hoi103_00203	hoi104_00118	hoi108_00874	hoi110_00259	hoi111_00696	hoi112_00259	hoi113_00203	hoi114_00259	hoi115_00258	hoi116_00131	hoi105_00391	hoi107_00391	hoi109_00199	hoi117_00198	hoi106_00387
253	3-dehydroquinate synthase	16	Core		hoi102_00259	hoi103_00202	hoi104_00117	hoi108_00875	hoi110_00260	hoi111_00695	hoi112_00260	hoi113_00202	hoi114_00260	hoi115_00259	hoi116_00130	hoi105_00392	hoi107_00392	hoi109_00198	hoi117_00197	hoi106_00388
254	3-dehydroquinate dehydratase	16	Core		hoi102_00260	hoi103_00201	hoi104_00116	hoi108_00876	hoi110_00261	hoi111_00694	hoi112_00261	hoi113_00201	hoi114_00261	hoi115_00260	hoi116_00129	hoi105_00393	hoi107_00393	hoi109_00197	hoi117_00196	hoi106_00389
255	Xaa-Pro dipeptidase	16	Core		hoi102_00261	hoi103_00200	hoi104_00115	hoi108_00877	hoi110_00262	hoi111_00693	hoi112_00262	hoi113_00200	hoi114_00262	hoi115_00261	hoi116_00128	hoi105_00394	hoi107_00394	hoi109_00196	hoi117_00195	hoi106_00390
256	Elongation factor P	16	Core		hoi102_00262	hoi103_00199	hoi104_00114	hoi108_00878	hoi110_00263	hoi111_00692	hoi112_00263	hoi113_00199	hoi114_00263	hoi115_00262	hoi116_00127	hoi105_00395	hoi107_00395	hoi109_00195	hoi117_00194	hoi106_00391
257	hypothetical protein	16	Core		hoi102_00263	hoi103_00198	hoi104_00113	hoi108_00879	hoi110_00264	hoi111_00691	hoi112_00264	hoi113_00198	hoi114_00264	hoi115_00263	hoi116_00126	hoi105_00396	hoi107_00396	hoi109_00194	hoi117_00193	hoi106_00392
258	hypothetical protein	15	Accessory		hoi102_00264	hoi103_00197	hoi104_00112	hoi108_00880	hoi110_00265	hoi111_00690	hoi112_00265	hoi113_00197	hoi114_00265	hoi115_00264	hoi116_00125		hoi107_00397	hoi109_00193	hoi117_00192	hoi106_00393
259	hypothetical protein	16	Core		hoi102_00265	hoi103_00196	hoi104_00111	hoi108_00881	hoi110_00266	hoi111_00689	hoi112_00266	hoi113_00196	hoi114_00266	hoi115_00265	hoi116_00124	hoi105_00875	hoi107_00398	hoi109_00192	hoi117_00191	hoi106_00394
260	hypothetical protein	16	Core		hoi102_00266	hoi103_00195	hoi104_00110	hoi108_00882	hoi110_00267	hoi111_00688	hoi112_00267	hoi113_00195	hoi114_00267	hoi115_00266	hoi116_00123	hoi105_00876	hoi107_00399	hoi109_00191	hoi117_00190	hoi106_00395
261	hypothetical protein	16	Core		hoi102_00267	hoi103_00194	hoi104_00109	hoi108_00883	hoi110_00268	hoi111_00687	hoi112_00268	hoi113_00194	hoi114_00268	hoi115_00267	hoi116_00122	hoi105_00877	hoi107_00400	hoi109_00190	hoi117_00189	hoi106_00396
262	Epimerase family protein	16	Core		hoi102_00268	hoi103_00193	hoi104_00108	hoi108_00884	hoi110_00269	hoi111_00686	hoi112_00269	hoi113_00193	hoi114_00269	hoi115_00268	hoi116_00121	hoi105_00878	hoi107_00401	hoi109_00189	hoi117_00188	hoi106_00397
263	Bifunctional protein PyrR	16	Core		hoi102_00269	hoi103_00192	hoi104_00107	hoi108_00885	hoi110_00270	hoi111_00685	hoi112_00270	hoi113_00192	hoi114_00270	hoi115_00269	hoi116_00120	hoi105_00879	hoi107_00402	hoi109_00188	hoi117_00187	hoi106_00398
264	Aspartate carbamoyltransferase	16	Core		hoi102_00270	hoi103_00191	hoi104_00106	hoi108_00886	hoi110_00271	hoi111_00684	hoi112_00271	hoi113_00191	hoi114_00271	hoi115_00270	hoi116_00119	hoi105_00880	hoi107_00403	hoi109_00187	hoi117_00186	hoi106_00399
265	Dihydroorotase	16	Core		hoi102_00271	hoi103_00190	hoi104_00105	hoi108_00887	hoi110_00272	hoi111_00683	hoi112_00272	hoi113_00190	hoi114_00272	hoi115_00271	hoi116_00118	hoi105_00881	hoi107_00404	hoi109_00186	hoi117_00185	hoi106_00400
266	Carbamoyl-phosphate synthase small chain	16	Core		hoi102_00272	hoi103_00189	hoi104_00104	hoi108_00888	hoi110_00273	hoi111_00682	hoi112_00273	hoi113_00189	hoi114_00273	hoi115_00272	hoi116_00117	hoi105_00882	hoi107_00405	hoi109_00185	hoi117_00184	hoi106_00401
267	Carbamoyl-phosphate synthase large chain	16	Core		hoi102_00273	hoi103_00188	hoi104_00103	hoi108_00889	hoi110_00274	hoi111_00681	hoi112_00274	hoi113_00188	hoi114_00274	hoi115_00273	hoi116_00116	hoi105_00883	hoi107_00406	hoi109_00184	hoi117_00183	hoi106_00402
268	orotidine 5'-phosphate decarboxylase	16	Core		hoi102_00274	hoi103_00187	hoi104_00102	hoi108_00890	hoi110_00275	hoi111_00680	hoi112_00275	hoi113_00187	hoi114_00275	hoi115_00274	hoi116_00115	hoi105_00884	hoi107_00407	hoi109_00183	hoi117_00182	hoi106_00403
269	hypothetical protein	16	Core		hoi102_00275	hoi103_00186	hoi104_00101	hoi108_00891	hoi110_00276	hoi111_00679	hoi112_00276	hoi113_00186	hoi114_00276	hoi115_00275	hoi116_00114	hoi105_00885	hoi107_00408	hoi109_00182	hoi117_00181	hoi106_00404
270	Guanylate kinase	16	Core		hoi102_00276	hoi103_00185	hoi104_00100	hoi108_00892	hoi110_00277	hoi111_00678	hoi112_00277	hoi113_00185	hoi114_00277	hoi115_00276	hoi116_00113	hoi105_00886	hoi107_00409	hoi109_00181	hoi117_00180	hoi106_00405
271	DNA-directed RNA polymerase subunit omega	16	Core		hoi102_00277	hoi103_00184	hoi104_00099	hoi108_00893	hoi110_00278	hoi111_00677	hoi112_00278	hoi113_00184	hoi114_00278	hoi115_00277	hoi116_00112	hoi105_00887	hoi107_00410	hoi109_00180	hoi117_00179	hoi106_00406
272	Coenzyme A biosynthesis bifunctional protein Cc	16	Core		hoi102_00278	hoi103_00183	hoi104_00098	hoi108_00894	hoi110_00279	hoi111_00676	hoi112_00279	hoi113_00183	hoi114_00279	hoi115_00278	hoi116_00111	hoi105_00888	hoi107_00411	hoi109_00179	hoi117_00178	hoi106_00407
273	S-adenosylmethionine synthase	16	Core		hoi102_00279	hoi103_00182	hoi104_00097	hoi108_00895	hoi110_00280	hoi111_00675	hoi112_00280	hoi113_00182	hoi114_00280	hoi115_00279	hoi116_00110	hoi105_00889	hoi107_00412	hoi109_00178	hoi117_00177	hoi106_00408
274	Primosomal protein N'	16	Core		hoi102_00280	hoi103_00181	hoi104_00096	hoi108_00896	hoi110_00281	hoi111_00674	hoi112_00281	hoi113_00181	hoi114_00281	hoi115_00280	hoi116_00109	hoi105_00890	hoi107_00413	hoi109_00177	hoi117_00176	hoi106_00409
275	Bifunctional protein PutA	16	Core		hoi102_00281	hoi103_00180	hoi104_00095	hoi108_00897	hoi110_00282	hoi111_00673	hoi112_00282	hoi113_00180	hoi114_00282	hoi115_00281	hoi116_00108	hoi105_00891	hoi107_00414	hoi109_00176	hoi117_00175	hoi106_00410
276	hypothetical protein	3	Accessory													hoi105_00892	hoi107_00415	hoi109_00175		
277	hypothetical protein	11	Accessory	GI4	hoi102_00282	hoi103_00179	hoi104_00094	hoi108_00898	hoi110_00283	hoi111_00672	hoi112_00283	hoi113_00179	hoi114_00283	hoi115_00282	hoi116_00107					
278	Helix-turn-helix	11	Accessory	GI4	hoi102_00283	hoi103_00178	hoi104_00093	hoi108_00899	hoi110_00284	hoi111_00671	hoi112_00284	hoi113_00178	hoi114_00284	hoi115_00283	hoi116_00106					
279	putative methyltransferase	11	Accessory	GI4	hoi102_00284	hoi103_00177	hoi104_00092	hoi108_00900	hoi110_00285	hoi111_00670	hoi112_00285	hoi113_00177	hoi114_00285	hoi115_00284	hoi116_00105					
280	Type III restriction enzyme, res subunit	11	Accessory	GI4	hoi102_00285	hoi103_00176	hoi104_00091	hoi108_00901	hoi110_00286	hoi111_00669	hoi112_00286	hoi113_00176	hoi114_00286	hoi115_00285	hoi116_00104					
281	recombination protein F	11	Accessory	GI4	hoi102_00286	hoi103_00175	hoi104_00090	hoi108_00902	hoi110_00287	hoi111_00668	hoi112_00287	hoi113_00175	hoi114_00287	hoi115_00286	hoi116_00103					
282	ATP-dependent helicase/nuclease subunit A	11	Accessory	GI4	hoi102_00287	hoi103_00174	hoi104_00089	hoi108_00903	hoi110_00288	hoi111_00667	hoi112_00288	hoi113_00174	hoi114_00288	hoi115_00287	hoi116_00102					
283	hypothetical protein	15	Accessory		hoi102_00288	hoi103_00173	hoi104_00088	hoi108_00904	hoi110_00289	hoi111_00666	hoi112_00289	hoi113_00173	hoi114_00289	hoi115_00288	hoi116_00101	hoi105_00893	hoi107_00416	hoi109_00174	hoi117_00174	
284	type I restriction enzyme EcoKI subunit R	12	Accessory		hoi102_00289	hoi103_00172	hoi104_00087	hoi108_00905	hoi110_00290	hoi111_00665	hoi112_00290	hoi113_00172	hoi114_00290	hoi115_00289	hoi116_00100					hoi106_00412
285	Ascorbate-specific phosphotransferase enzyme I	15	Accessory		hoi102_00290	hoi103_00171	hoi104_00086	hoi108_00906	hoi110_00291	hoi111_00664	hoi112_00291	hoi113_00171	hoi114_00291	hoi115_00290	hoi116_00099	hoi105_00894	hoi107_00417	hoi109_00173	hoi117_00173	
286	Ascorbate-specific permease IIC component Ula/	15	Accessory		hoi102_00291	hoi103_00170	hoi104_00085	hoi108_00907	hoi110_00292	hoi111_00663	hoi112_00292	hoi113_00170	hoi114_00292	hoi115_00291	hoi116_00098	hoi105_00895	hoi107_00418	hoi109_00172	hoi117_00172	
287	hypothetical protein	4	Accessory	GI5												hoi105_00896	hoi107_00419	hoi109_00171	hoi117_00171	
288	hypothetical protein	4	Accessory	GI5												hoi105_00897	hoi107_00420	hoi109_00170	hoi117_00170	
289	DNA polymerase III subunit epsilon	15	Accessory		hoi102_00292	hoi103_00169	hoi104_00084	hoi108_00908	hoi110_00293	hoi111_00662	hoi112_00293	hoi113_00169	hoi114_00293	hoi115_00292	hoi116_00097	hoi105_00898	hoi107_00421	hoi109_00169	hoi117_00169	
290	ASCH domain protein	15	Accessory		hoi102_00293	hoi103_00168	hoi104_00083	hoi108_00909	hoi110_00294	hoi111_00661	hoi112_00294	hoi113_00168	hoi114_00294	hoi115_00293	hoi116_00096	hoi105_00899	hoi107_00422	hoi109_00168	hoi117_00168	
291	hypothetical protein	16	Core		hoi102_00294	hoi103_00167	hoi104_00082	hoi108_00910	hoi110_00295	hoi111_00660	hoi112_00295	hoi113_00167	hoi114_00295	hoi115_00294	hoi116_00095	hoi105_00900	hoi107_00423	hoi109_00167	hoi117_00167	hoi106_00415
292	hypothetical protein	16	Core		hoi102_00295	hoi103_00166	hoi104_00081	hoi108_00911	hoi110_00296	hoi111_00659	hoi112_00296	hoi113_00166	hoi114_00296	hoi115_00295	hoi116_00094	hoi105_00901	hoi107_00424	hoi109_00166	hoi117_00166	hoi106_00416
293	Carboxylate-amine ligase YbdK	16	Core		hoi102_00296	hoi103_00165	hoi104_00080	hoi108_00912	hoi110_00297	hoi111_00658	hoi112_00297	hoi113_00165	hoi114_00297	hoi115_00296	hoi116_00093	hoi105_00902	hoi107_00425	hoi109_00165	hoi117_00165	hoi106_00417
294	ABC-2 family transporter protein	16	Core		hoi102_00297	hoi103_00164	hoi104_00079	hoi108_00913	hoi110_00298	hoi111_00657	hoi112_00298	hoi113_00164	hoi114_00298	hoi115_00297	hoi116_00092	hoi105_00903	hoi107_00426	hoi109_00164	hoi117_00164	hoi106_00418
295	Diacylglycerol kinase	16	Core		hoi102_00298	hoi103_00163	hoi104_00078	hoi108_00914	hoi110_00299	hoi111_00656	hoi112_00299	hoi113_00163	hoi114_00299	hoi115_00298	hoi116_00091	hoi105_00904	hoi107_00427	hoi109_00163	hoi117_00163	hoi106_00419
296	putative FAD-linked oxidoreductase	16	Core		hoi102_00299	hoi103_00162	hoi104_00077	hoi108_00915	hoi110_00300	hoi111_00655	hoi112_00300	hoi113_00162	hoi114_00300	hoi115_00299	hoi116_00090	hoi105_00905	hoi107_00428	hoi109_00162	hoi117_00162	hoi106_00420
297	Bacterial regulatory proteins, tetR family	16	Core		hoi102_00300	hoi103_00161	hoi104_00076	hoi108_00916	hoi110_00301	hoi111_00654	hoi112_00301	hoi113_00161	hoi114_00301	hoi115_00300	hoi116_00089	hoi105_00906	hoi107_00429	hoi109_00161	hoi117_00161	hoi106_00421
298	Aerobic glycerol-3-phosphate dehydrogenase	16	Core		hoi102_00301	hoi103_00160	hoi104_00075	hoi108_00917	hoi110_00302	hoi111_00653	hoi112_00302	hoi113_00160	hoi114_00302	hoi115_00301	hoi116_00088	hoi105_00907	hoi107_00430	hoi109_00160	hoi117_00160	hoi106_00422
299	hypothetical protein	16	Core		hoi102_00302	hoi103_00159	hoi104_00074	hoi108_00918	hoi110_00303	hoi111_00652	hoi112_00303	hoi113_00159	hoi114_00303	hoi115_00302	hoi116_00087	hoi105_00908	hoi107_00431	hoi109_00159	hoi117_00159	hoi106_00423
300	Peptide deformylase	16	Core		hoi102_00303	hoi103_00158	hoi104_00073	hoi108_00919	hoi110_00304	hoi111_00651	hoi112_00304	hoi113_00158	hoi114_00304	hoi115_00303	hoi116_00086	hoi105_00909	hoi107_00432	hoi109_00158	hoi117_00158	hoi106_00424
301	Methionyl-tRNA formyltransferase	16	Core		hoi102_00304	hoi103_00157	hoi104_00072	hoi108_00920	hoi110_00305	hoi111_00650	hoi112_00305	hoi113_00157	hoi114_00305	hoi115_00304	hoi116_00085	hoi105_00910	hoi107_00433	hoi109_00157	hoi117_00157	hoi106_00425
302	Ribosomal RNA small subunit methyltransferase	16	Core		hoi102_00305	hoi103_00156	hoi104_00071	hoi108_00921	hoi110_00306	hoi111_00649	hoi112_00306	hoi113_00156	hoi114_00306	hoi115_00305	hoi116_00084	hoi105_00911	hoi107_00434	hoi109_00156	hoi117_00156	hoi106_00426
303	Ribulose-phosphate 3-epimerase	16	Core		hoi102_00306	hoi103_00155	hoi104_00070	hoi108_00922	hoi110_00307	hoi111_00648	hoi112_00307	hoi113_00155	hoi114_00307	hoi115_00306	hoi116_00083	hoi105_00912	hoi107_00435	hoi109_00155	hoi117_00155	hoi106_00427
304	Riboflavin biosynthesis protein RibD	16	Core		hoi102_00307	hoi103_00154	hoi104_00069	hoi108_00923	hoi110_00308	hoi111_00647	hoi112_00308	hoi113_00154	hoi114_00308	hoi115_00307	hoi116_00082	hoi105_00913	hoi107_00436	hoi109_00154	hoi117_00154	hoi106_00428
305	Riboflavin synthase	16	Core		hoi102_00308	hoi103_00153	hoi104_00068	hoi108_00924	hoi110_00309	hoi111_00646	hoi112_00309	hoi113_00153	hoi114_00309	hoi115_00308	hoi116_00081	hoi105_00914	hoi107_00437	hoi109_00153	hoi117_00153	hoi106_00429
306	Riboflavin biosynthesis protein RibBA	16	Core		hoi102_00309	hoi103_00152	hoi104_00067	hoi108_00925	hoi110_00310	hoi111_00645	hoi112_00310	hoi113_00152	hoi114_00310	hoi115_00309	hoi116_00080	hoi105_00915	hoi107_00438	hoi109_00152	hoi117_00152	hoi106_00430
307	6,7-dimethyl-8-ribityllumazine synthase	16	Core		hoi102_00310	hoi103_00151	hoi104_00066	hoi108_00926	hoi110_00311	hoi111_00644	hoi112_00311	hoi113_00151	hoi114_00311	hoi115_00310	hoi116_00079	hoi105_00916	hoi107_00439	hoi109_00151	hoi117_00151	hoi106_00431
308	hypothetical protein	16	Core		hoi102_00311	hoi103_00150	hoi104_00065	hoi108_00927	hoi110_00312	hoi111_00643	hoi112_00312	hoi113_00150	hoi114_00312	hoi115_00311	hoi116_00078	hoi105_00917	hoi107_00440	hoi109_00150	hoi117_00150	hoi106_00432
309	UvrABC system protein C	16	Core		hoi102_00312	hoi103_00149	hoi104_00064	hoi108_00928	hoi110_00313	hoi111_00642	hoi112_00313	hoi113_00149	hoi114_00313	hoi115_00312	hoi116_00077	hoi105_00918	hoi107_00441	hoi109_00149	hoi117_00149	hoi106_00433
310	glmZ(sRNA)-inactivating NTPase	16	Core		hoi102_00313	hoi103_00148	hoi104_00063	hoi108_00929	hoi110_00314	hoi111_00641	hoi112_00314	hoi113_00148	hoi114_00314	hoi115_00313	hoi116_00076	hoi105_00919	hoi107_00442	hoi109_00148	hoi117_00148	hoi106_00434
311	Putative gluconeogenesis factor	16	Core		hoi102_00314	hoi103_00147	hoi104_00062	hoi108_00930	hoi110_00315	hoi111_00640	hoi112_00315	hoi113_00147	hoi114_00315	hoi115_00314	hoi116_00075	hoi105_00920	hoi107_00443	hoi109_00147	hoi117_00147	hoi106_00435
312	Putative sporulation transcription regulator Whi/	16	Core		hoi102_00315	hoi103_00146	hoi104_00061	hoi108_00931	hoi110_00316	hoi111_00639	hoi112_00316	hoi113_00146	hoi114_00316	hoi115_00315	hoi116_00074	hoi105_00921	hoi107_00444	hoi109_00146	hoi117_00146	hoi106_00436
313	Glyceraldehyde-3-phosphate dehydrogenase	16	Core		hoi102_00316	hoi103_00145	hoi104_00060	hoi108_00932	hoi110_00317	hoi111_00638	hoi112_00317	hoi113_00145	hoi114_00317	hoi115_00316	hoi116_00073	hoi105_00922	hoi107_00445	hoi109_00145	hoi117_00145	hoi106_00437
314	Phosphoglycerate kinase	16	Core		hoi102_00317	hoi103_00144	hoi104_00059	hoi108_00933	hoi110_00318	hoi111_00637	hoi112_00318	hoi113_00144	hoi114_00318	hoi115_00317	hoi116_00072	hoi105_00923	hoi107_00446	hoi109_00144	hoi117_00144	hoi106_00438
315	Triosephosphate isomerase	16	Core		hoi102_00318	hoi103_00143	hoi104_00058	hoi108_00934	hoi110_00319	hoi111_00636	hoi112_00319	hoi113_00143	hoi114_00319	hoi115_00318	hoi116_00071	hoi105_00924	hoi107_00447	hoi109_00143	hoi117_00143	hoi106_00439
316	preprotein translocase subunit SecG	16	Core		hoi102_00319	hoi103_00142	hoi104_00057	hoi108_00935	hoi110_00320	hoi111_00635	hoi112_00320	hoi113_00142	hoi114_00320	hoi115_00319	hoi116_00070	hoi105_00925	hoi107_00448	hoi109_00142	hoi117_00142	hoi106_00440
317	6-phosphogluconolactonase	16	Core		hoi102_00320	hoi103_00141	hoi104_00056	hoi108_00936	hoi110_00321	hoi111_00634	hoi112_00321	hoi113_00141	hoi114_00321	hoi115_00320	hoi116_00069	hoi105_00926	hoi107_00449	hoi109_00141	hoi117_00141	hoi106_00441

318	Glucose-6-phosphate dehydrogenase subunit	16	Core	hoi102_00321	hoi103_00140	hoi104_00055	hoi108_00937	hoi110_00322	hoi111_00633	hoi112_00322	hoi113_00140	hoi114_00322	hoi115_00321	hoi116_00068	hoi105_00927	hoi107_00450	hoi109_00140	hoi117_00140	hoi106_00442
319	Glucose-6-phosphate 1-dehydrogenase	16	Core	hoi102_00322	hoi103_00139	hoi104_00054	hoi108_00938	hoi110_00323	hoi111_00632	hoi112_00323	hoi113_00139	hoi114_00323	hoi115_00322	hoi116_00067	hoi105_00928	hoi107_00451	hoi109_00139	hoi117_00139	hoi106_00443
320	Transaldolase	16	Core	hoi102_00323	hoi103_00138	hoi104_00053	hoi108_00939	hoi110_00324	hoi111_00631	hoi112_00324	hoi113_00138	hoi114_00324	hoi115_00323	hoi116_00066	hoi105_00929	hoi107_00452	hoi109_00138	hoi117_00138	hoi106_00444
321	Transketolase	16	Core	hoi102_00324	hoi103_00137	hoi104_00052	hoi108_00940	hoi110_00325	hoi111_00630	hoi112_00325	hoi113_00137	hoi114_00325	hoi115_00324	hoi116_00065	hoi105_00930	hoi107_00453	hoi109_00137	hoi117_00137	hoi106_00445
322	Protoheme IX farnesyltransferase	16	Core	hoi102_00325	hoi103_00136	hoi104_00051	hoi108_00941	hoi110_00326	hoi111_00629	hoi112_00326	hoi113_00136	hoi114_00326	hoi115_00325	hoi116_00064	hoi105_00931	hoi107_00454	hoi109_00136	hoi117_00136	hoi106_00446
323	Quinone oxidoreductase 1	16	Core	hoi102_00326	hoi103_00135	hoi104_00050	hoi108_00942	hoi110_00327	hoi111_00628	hoi112_00327	hoi113_00135	hoi114_00327	hoi115_00326	hoi116_00063	hoi105_00932	hoi107_00455	hoi109_00135	hoi117_00135	hoi106_00447
324	Heme A synthase	16	Core	hoi102_00327	hoi103_00134	hoi104_00049	hoi108_00943	hoi110_00328	hoi111_00627	hoi112_00328	hoi113_00134	hoi114_00328	hoi115_00327	hoi116_00062	hoi105_00933	hoi107_00456	hoi109_00134	hoi117_00134	hoi106_00448
325	ABC-2 family transporter protein	16	Core	hoi102_00328	hoi103_00133	hoi104_00048	hoi108_00944	hoi110_00329	hoi111_00626	hoi112_00329	hoi113_00133	hoi114_00329	hoi115_00328	hoi116_00061	hoi105_00934	hoi107_00457	hoi109_00133	hoi117_00133	hoi106_00449
326	Daunorubicin/doxorubicin resistance ATP-bindin	16	Core	hoi102_00329	hoi103_00132	hoi104_00047	hoi108_00945	hoi110_00330	hoi111_00625	hoi112_00330	hoi113_00132	hoi114_00330	hoi115_00329	hoi116_00060	hoi105_00935	hoi107_00458	hoi109_00132	hoi117_00132	hoi106_00450
327	hypothetical protein	16	Core	hoi102_00330	hoi103_00131	hoi104_00046	hoi108_00946	hoi110_00331	hoi111_00624	hoi112_00331	hoi113_00131	hoi114_00331	hoi115_00330	hoi116_00059	hoi105_00936	hoi107_00459	hoi109_00131	hoi117_00131	hoi106_00451
328	Bacterial regulatory protein, arsR family	16	Core	hoi102_00331	hoi103_00130	hoi104_00045	hoi108_00947	hoi110_00332	hoi111_00623	hoi112_00332	hoi113_00130	hoi114_00332	hoi115_00331	hoi116_00058	hoi105_00937	hoi107_00460	hoi109_00130	hoi117_00130	hoi106_00452
329	FeS cluster assembly protein SufB	16	Core	hoi102_00332	hoi103_00129	hoi104_00044	hoi108_00948	hoi110_00333	hoi111_00622	hoi112_00333	hoi113_00129	hoi114_00333	hoi115_00332	hoi116_00057	hoi105_00938	hoi107_00461	hoi109_00129	hoi117_00129	hoi106_00453
330	FeS cluster assembly protein SufD	16	Core	hoi102_00333	hoi103_00128	hoi104_00043	hoi108_00949	hoi110_00334	hoi111_00621	hoi112_00334	hoi113_00128	hoi114_00334	hoi115_00333	hoi116_00056	hoi105_00939	hoi107_00462	hoi109_00128	hoi117_00128	hoi106_00454
331	Vegetative protein 296	16	Core	hoi102_00334	hoi103_00127	hoi104_00042	hoi108_00950	hoi110_00335	hoi111_00620	hoi112_00335	hoi113_00127	hoi114_00335	hoi115_00334	hoi116_00055	hoi105_00940	hoi107_00463	hoi109_00127	hoi117_00127	hoi106_00455
332	putative cysteine desulfurase	16	Core	hoi102_00335	hoi103_00126	hoi104_00041	hoi108_00951	hoi110_00336	hoi111_00619	hoi112_00336	hoi113_00126	hoi114_00336	hoi115_00335	hoi116_00054	hoi105_00941	hoi107_00464	hoi109_00126	hoi117_00126	hoi106_00456
333	NifU-like protein	16	Core	hoi102_00336	hoi103_00125	hoi104_00040	hoi108_00952	hoi110_00337	hoi111_00618	hoi112_00337	hoi113_00125	hoi114_00337	hoi115_00336	hoi116_00053	hoi105_00942	hoi107_00465	hoi109_00125	hoi117_00125	hoi106_00457
334	hypothetical protein	16	Core	hoi102_00337	hoi103_00124	hoi104_00039	hoi108_00953	hoi110_00338	hoi111_00617	hoi112_00338	hoi113_00124	hoi114_00338	hoi115_00337	hoi116_00052	hoi105_00943	hoi107_00466	hoi109_00124	hoi117_00124	hoi106_00458
335	Lycopene beta cyclase	16	Core	hoi102_00338	hoi103_00123	hoi104_00038	hoi108_00954	hoi110_00339	hoi111_00616	hoi112_00339	hoi113_00123	hoi114_00339	hoi115_00338	hoi116_00051	hoi105_00944	hoi107_00467	hoi109_00123	hoi117_00123	hoi106_00459
336	putative ABC transporter ATP-binding protein Yh	16	Core	hoi102_00339	hoi103_00122	hoi104_00037	hoi108_00955	hoi110_00340	hoi111_00615	hoi112_00340	hoi113_00122	hoi114_00340	hoi115_00339	hoi116_00050	hoi105_00945	hoi107_00468	hoi109_00122	hoi117_00122	hoi106_00460
337	hypothetical protein	16	Core	hoi102_00340	hoi103_00121	hoi104_00036	hoi108_00956	hoi110_00341	hoi111_00614	hoi112_00341	hoi113_00121	hoi114_00341	hoi115_00340	hoi116_00049	hoi105_00946	hoi107_00469	hoi109_00121	hoi117_00121	hoi106_00461
338	hypothetical protein	16	Core	hoi102_00341	hoi103_00120	hoi104_00035	hoi108_00957	hoi110_00342	hoi111_00613	hoi112_00342	hoi113_00120	hoi114_00342	hoi115_00341	hoi116_00048	hoi105_00947	hoi107_00470	hoi109_00120	hoi117_00120	hoi106_00462
339	GMP synthase [glutamine-hydrolyzing]	16	Core	hoi102_00342	hoi103_00119	hoi104_00034	hoi108_00958	hoi110_00343	hoi111_00612	hoi112_00343	hoi113_00119	hoi114_00343	hoi115_00342	hoi116_00047	hoi105_00948	hoi107_00471	hoi109_00119	hoi117_00119	hoi106_00463
340	Formate--tetrahydrofolate ligase	16	Core	hoi102_00343	hoi103_00118	hoi104_00033	hoi108_00959	hoi110_00344	hoi111_00611	hoi112_00344	hoi113_00118	hoi114_00344	hoi115_00343	hoi116_00046	hoi105_00949	hoi107_00472	hoi109_00118	hoi117_00118	hoi106_00464
341	HTH-type transcriptional repressor AcnR	16	Core	hoi102_00344	hoi103_00117	hoi104_00032	hoi108_00960	hoi110_00345	hoi111_00610	hoi112_00345	hoi113_00117	hoi114_00345	hoi115_00344	hoi116_00045	hoi105_00950	hoi107_00473	hoi109_00117	hoi117_00117	hoi106_00465
342	Aconitate hydratase	16	Core	hoi102_00345	hoi103_00116	hoi104_00031	hoi108_00961	hoi110_00346	hoi111_00609	hoi112_00346	hoi113_00116	hoi114_00346	hoi115_00345	hoi116_00044	hoi105_00951	hoi107_00474	hoi109_00116	hoi117_00116	hoi106_00466
343	hypothetical protein	16	Core	hoi102_00346	hoi103_00115	hoi104_00030	hoi108_00962	hoi110_00347	hoi111_00608	hoi112_00347	hoi113_00115	hoi114_00347	hoi115_00346	hoi116_00043	hoi105_00952	hoi107_00475	hoi109_00115	hoi117_00115	hoi106_00467
344	Peptidoglycan endopeptidase RipA precursor	16	Core	hoi102_00347	hoi103_00114	hoi104_00029	hoi108_00963	hoi110_00348	hoi111_00607	hoi112_00348	hoi113_00114	hoi114_00348	hoi115_00347	hoi116_00042	hoi105_00953	hoi107_00476	hoi109_00114	hoi117_00114	hoi106_00468
345	Ferrochelatase	16	Core	hoi102_00348	hoi103_00113	hoi104_00028	hoi108_00964	hoi110_00349	hoi111_00606	hoi112_00349	hoi113_00113	hoi114_00349	hoi115_00348	hoi116_00041	hoi105_00954	hoi107_00477	hoi109_00113	hoi117_00113	hoi106_00469
346	hypothetical protein	16	Core	hoi102_00349	hoi103_00112	hoi104_00027	hoi108_00965	hoi110_00350	hoi111_00605	hoi112_00350	hoi113_00112	hoi114_00350	hoi115_00349	hoi116_00040	hoi105_00955	hoi107_00478	hoi109_00112	hoi117_00112	hoi106_00470
347	hypothetical protein	16	Core	hoi102_00350	hoi103_00111	hoi104_00026	hoi108_00966	hoi110_00351	hoi111_00604	hoi112_00351	hoi113_00111	hoi114_00351	hoi115_00350	hoi116_00039	hoi105_00956	hoi107_00479	hoi109_00111	hoi117_00111	hoi106_00471
348	Modulator of FtsH protease HflK	16	Core	hoi102_00351	hoi103_00110	hoi104_00025	hoi108_00967	hoi110_00352	hoi111_00603	hoi112_00352	hoi113_00110	hoi114_00352	hoi115_00351	hoi116_00038	hoi105_00957	hoi107_00480	hoi109_00110	hoi117_00110	hoi106_00472
349	hypothetical protein	16	Core	hoi102_00352	hoi103_00109	hoi104_00024	hoi108_00968	hoi110_00353	hoi111_00602	hoi112_00353	hoi113_00109	hoi114_00353	hoi115_00352	hoi116_00037	hoi105_00958	hoi107_00481	hoi109_00109	hoi117_00109	hoi106_00473
350	TVP38/TMEM64 family inner membrane protein	16	Core	hoi102_00353	hoi103_00108	hoi104_00023	hoi108_00969	hoi110_00354	hoi111_00601	hoi112_00354	hoi113_00108	hoi114_00354	hoi115_00353	hoi116_00036	hoi105_00959	hoi107_00482	hoi109_00108	hoi117_00108	hoi106_00474
351	Methylmalonyl-CoA mutase small subunit	16	Core	hoi102_00354	hoi103_00107	hoi104_00022	hoi108_00970	hoi110_00355	hoi111_00600	hoi112_00355	hoi113_00107	hoi114_00355	hoi115_00354	hoi116_00035	hoi105_00960	hoi107_00483	hoi109_00107	hoi117_00107	hoi106_00475
352	Methylmalonyl-CoA mutase large subunit	16	Core	hoi102_00355	hoi103_00106	hoi104_00021	hoi108_00971	hoi110_00356	hoi111_00599	hoi112_00356	hoi113_00106	hoi114_00356	hoi115_00355	hoi116_00034	hoi105_00961	hoi107_00484	hoi109_00106	hoi117_00106	hoi106_00476
353	putative GTPase/MT1543	16	Core	hoi102_00356	hoi103_00105	hoi104_00020	hoi108_00972	hoi110_00357	hoi111_00598	hoi112_00357	hoi113_00105	hoi114_00357	hoi115_00356	hoi116_00033	hoi105_00962	hoi107_00485	hoi109_00105	hoi117_00105	hoi106_00477
354	hypothetical protein	16	Core	hoi102_00357	hoi103_00104	hoi104_00019	hoi108_00973	hoi110_00358	hoi111_00597	hoi112_00358	hoi113_00104	hoi114_00358	hoi115_00357	hoi116_00032	hoi105_00963	hoi107_00486	hoi109_00104	hoi117_00104	hoi106_00478
355	HTH-type transcriptional regulator GlvR	16	Core	hoi102_00358	hoi103_00103	hoi104_00018	hoi108_00974	hoi110_00359	hoi111_00596	hoi112_00359	hoi113_00103	hoi114_00359	hoi115_00358	hoi116_00031	hoi105_00964	hoi107_00487	hoi109_00103	hoi117_00103	hoi106_00479
356	PTS system maltose-specific EIICB component	16	Core	hoi102_00359	hoi103_00102	hoi104_00017	hoi108_00975	hoi110_00360	hoi111_00595	hoi112_00360	hoi113_00102	hoi114_00360	hoi115_00359	hoi116_00030	hoi105_00965	hoi107_00488	hoi109_00102	hoi117_00102	hoi106_00480
357	Maltose 6'-phosphate phosphatase	16	Core	hoi102_00360	hoi103_00101	hoi104_00016	hoi108_00976	hoi110_00361	hoi111_00594	hoi112_00361	hoi113_00101	hoi114_00361	hoi115_00360	hoi116_00029	hoi105_00966	hoi107_00489	hoi109_00101	hoi117_00101	hoi106_00481
358	Oligo-1,6-glucosidase	16	Core	hoi102_00361	hoi103_00100	hoi104_00015	hoi108_00977	hoi110_00362	hoi111_00593	hoi112_00362	hoi113_00100	hoi114_00362	hoi115_00361	hoi116_00028	hoi105_00967	hoi107_00490	hoi109_00100	hoi117_00100	hoi106_00482
359	putative kinase inhibitor protein	16	Core	hoi102_00363	hoi103_00098	hoi104_00013	hoi108_00979	hoi110_00364	hoi111_00591	hoi112_00364	hoi113_00098	hoi114_00364	hoi115_00363	hoi116_00026	hoi105_00969	hoi107_00492	hoi109_00098	hoi117_00098	hoi106_00484
360	hypothetical protein	16	Core	hoi102_00364	hoi103_00097	hoi104_00012	hoi108_00980	hoi110_00365	hoi111_00590	hoi112_00365	hoi113_00097	hoi114_00365	hoi115_00364	hoi116_00025	hoi105_00970	hoi107_00493	hoi109_00097	hoi117_00097	hoi106_00485
361	Dihydroorotate dehydrogenase (quinone)	16	Core	hoi102_00365	hoi103_00096	hoi104_00011	hoi108_00981	hoi110_00366	hoi111_00589	hoi112_00366	hoi113_00096	hoi114_00366	hoi115_00365	hoi116_00024	hoi105_00971	hoi107_00494	hoi109_00096	hoi117_00096	hoi106_00486
362	hypothetical protein	16	Core	hoi102_00366	hoi103_00095	hoi104_00010	hoi108_00982	hoi110_00367	hoi111_00588	hoi112_00367	hoi113_00095	hoi114_00367	hoi115_00366	hoi116_00023	hoi105_00972	hoi107_00495	hoi109_00095	hoi117_00095	hoi106_00487
363	General stress protein 69	16	Core	hoi102_00367	hoi103_00094	hoi104_00009	hoi108_00983	hoi110_00368	hoi111_00587	hoi112_00368	hoi113_00094	hoi114_00368	hoi115_00367	hoi116_00022	hoi105_00973	hoi107_00496	hoi109_00094	hoi117_00094	hoi106_00488
364	Undecaprenyl-diphosphatase	16	Core	hoi102_00368	hoi103_00093	hoi104_00008	hoi108_00984	hoi110_00369	hoi111_00586	hoi112_00369	hoi113_00093	hoi114_00369	hoi115_00368	hoi116_00021	hoi105_00974	hoi107_00497	hoi109_00093	hoi117_00093	hoi106_00489
365	L-cysteine:1D-myo-inositol 2-amino-2-deoxy-alpl	16	Core	hoi102_00369	hoi103_00092	hoi104_00007	hoi108_00985	hoi110_00370	hoi111_00585	hoi112_00370	hoi113_00092	hoi114_00370	hoi115_00369	hoi116_00020	hoi105_00975	hoi107_00498	hoi109_00092	hoi117_00092	hoi106_00490
366	hypothetical protein	16	Core	hoi102_00370	hoi103_00091	hoi104_00006	hoi108_00986	hoi110_00371	hoi111_00584	hoi112_00371	hoi113_00091	hoi114_00371	hoi115_00370	hoi116_00019	hoi105_00976	hoi107_00499	hoi109_00091	hoi117_00091	hoi106_00491
367	Methionine synthase	16	Core	hoi102_00371	hoi103_00090	hoi104_00005	hoi108_00987	hoi110_00372	hoi111_00583	hoi112_00372	hoi113_00090	hoi114_00372	hoi115_00371	hoi116_00018	hoi105_00977	hoi107_00500	hoi109_00090	hoi117_00090	hoi106_00492
368	hypothetical protein	16	Core	hoi102_00372	hoi103_00089	hoi104_00004	hoi108_00988	hoi110_00373	hoi111_00582	hoi112_00373	hoi113_00089	hoi114_00373	hoi115_00372	hoi116_00017	hoi105_00978	hoi107_00501	hoi109_00089	hoi117_00089	hoi106_00493
369	Phosphorylated carbohydrates phosphatase	16	Core	hoi102_00373	hoi103_00088	hoi104_00003	hoi108_00989	hoi110_00374	hoi111_00581	hoi112_00374	hoi113_00088	hoi114_00374	hoi115_00373	hoi116_00016	hoi105_00979	hoi107_00502	hoi109_00088	hoi117_00088	hoi106_00494
370	Phosphoribosyl-ATP pyrophosphatase	16	Core	hoi102_00374	hoi103_00087	hoi104_00002	hoi108_00990	hoi110_00375	hoi111_00580	hoi112_00375	hoi113_00087	hoi114_00375	hoi115_00374	hoi116_00015	hoi105_00980	hoi107_00503	hoi109_00087	hoi117_00087	hoi106_00495
371	ATP phosphoribosyltransferase	16	Core	hoi102_00375	hoi103_00086	hoi104_00001	hoi108_00991	hoi110_00376	hoi111_00579	hoi112_00376	hoi113_00086	hoi114_00376	hoi115_00375	hoi116_00014	hoi105_00981	hoi107_00504	hoi109_00086	hoi117_00086	hoi106_00496
372	Aspartate ammonia-lyase	16	Core	hoi102_00376	hoi103_00085	hoi104_01946	hoi108_00992	hoi110_00377	hoi111_00578	hoi112_00377	hoi113_00085	hoi114_00377	hoi115_00376	hoi116_00013	hoi105_00982	hoi107_00505	hoi109_00085	hoi117_00085	hoi106_00497
373	Anaerobic C4-dicarboxylate transporter DcuA	16	Core	hoi102_00377	hoi103_00084	hoi104_01947	hoi108_00993	hoi110_00378	hoi111_00577	hoi112_00378	hoi113_00084	hoi114_00378	hoi115_00377	hoi116_00012	hoi105_00983	hoi107_00506	hoi109_00084	hoi117_00084	hoi106_00498
374	C4-dicarboxylate transporter/malic acid transpor	16	Core	hoi102_00378	hoi103_00083	hoi104_01948	hoi108_00994	hoi110_00379	hoi111_00576	hoi112_00379	hoi113_00083	hoi114_00379	hoi115_00378	hoi116_00011	hoi105_00984	hoi107_00507	hoi109_00083	hoi117_00083	hoi106_00499
375	HTH-type transcriptional regulator CysB	16	Core	hoi102_00379	hoi103_00082	hoi104_01949	hoi108_00995	hoi110_00380	hoi111_00575	hoi112_00380	hoi113_00082	hoi114_00380	hoi115_00379	hoi116_00010	hoi105_00985	hoi107_00508	hoi109_00082	hoi117_00082	hoi106_00500
376	PD-(D/E)XK nuclease superfamily protein	16	Core	hoi102_00380	hoi103_00081	hoi104_01950	hoi108_00996	hoi110_00381	hoi111_00574	hoi112_00381	hoi113_00081	hoi114_00381	hoi115_00380	hoi116_00009	hoi105_00986	hoi107_00509	hoi109_00081	hoi117_00081	hoi106_00501
377	putative M18 family aminopeptidase 2	16	Core	hoi102_00381	hoi103_00080	hoi104_01951	hoi108_00997	hoi110_00382	hoi111_00573	hoi112_00382	hoi113_00080	hoi114_00382	hoi115_00381	hoi116_00008	hoi105_00987	hoi107_00510	hoi109_00080	hoi117_00080	hoi106_00502
378	tRNA (adenine(58)-N(1))-methyltransferase TrmI	16	Core	hoi102_00382	hoi103_00079	hoi104_01952	hoi108_00998	hoi110_00383	hoi111_00572	hoi112_00383	hoi113_00079	hoi114_00383	hoi115_00382	hoi116_00007	hoi105_00988	hoi107_00511	hoi109_00079	hoi117_00079	hoi106_00503
379	Proteasome-associated ATPase	16	Core	hoi102_00383	hoi103_00078	hoi104_01953	hoi108_00999	hoi110_00384	hoi111_00571	hoi112_00384	hoi113_00078	hoi114_00384	hoi115_00383	hoi116_00006	hoi105_00989	hoi107_00512	hoi109_00078	hoi117_00078	hoi106_00504
380	Pup deamidase/depupylase	16	Core	hoi102_00384	hoi103_00077	hoi104_01954	hoi108_01000	hoi110_00385	hoi111_00570	hoi112_00385	hoi113_00077	hoi114_00385	hoi115_00384	hoi116_00005	hoi105_00990	hoi107_00513	hoi109_00077	hoi117_00077	hoi106_00505
381	Prokaryotic ubiquitin-like protein Pup	16	Core	hoi102_00385	hoi103_00076	hoi104_01955	hoi108_01001	hoi110_00386	hoi111_00569	hoi112_00386	hoi113_00076	hoi114_00386	hoi115_00385	hoi116_00004	hoi105_00991	hoi107_00514	hoi109_00076	hoi117_00076	hoi106_00506
382	Pup--protein ligase	16	Core	hoi102_00386	hoi103_00075	hoi104_01956	hoi108_01002	hoi110_00387	hoi111_00568	hoi112_00387	hoi113_00075	hoi114_00387	hoi115_00386	hoi116_00003	hoi105_00992	hoi107_00515	hoi109_00075	hoi117_00075	hoi106_00507
383	hypothetical protein	16	Core	hoi102_00387	hoi103_00074	hoi104_01957	hoi108_01003	hoi110_00388	hoi111_00567	hoi112_00388	hoi113_00074	hoi114_00388	hoi115_00387	hoi116_00002	hoi105_00993	hoi107_00516	hoi109_00074	hoi117_00074	hoi106_00508
384	hypothetical protein	16	Core	hoi102_00388	hoi103_00073	hoi104_01958	hoi108_01004	hoi110_00389	hoi111_00566	hoi112_00389	hoi113_00073	hoi114_00389	hoi115_00388	hoi116_00001	hoi105_00994	hoi107_00517	hoi109_00073	hoi117_00073	hoi106_00509
385	twin arginine translocase protein A	12	Accessory	hoi102_00389	hoi103_00072	hoi104_01959	hoi108_01005	hoi110_00390		hoi112_00390	hoi113_00072	hoi114_00390	hoi115_00389		hoi105_00995		hoi109_00072	hoi117_00072	
386	Sec-independent protein translocase protein Tat	16	Core	hoi102_00390	hoi103_00071	hoi104_01960	hoi108_01006	hoi110_00391	hoi111_01915	hoi112_00391	hoi113_00071	hoi114_00391	hoi115_00390	hoi116_02008	hoi105_00996	hoi107_02090	hoi109_00071	hoi117_00071	hoi106_01820
387	ski2-like helicase	16	Core	hoi102_00391	hoi103_00070	hoi104_01961	hoi108_01007	hoi110_00392	hoi111_01916	hoi112_00392	hoi113_00070	hoi114_00392	hoi115_00391	hoi116_02009	hoi105_00997	hoi107_02089	hoi109_00070	hoi117_00070	hoi106_01821
388	Xaa-Pro dipeptidase	16	Core	hoi102_00392	hoi103_00069	hoi104_01962	hoi108_01008	hoi110_00393	hoi111_01917	hoi112_00393	hoi113_00069	hoi114_00393	hoi115_00392	hoi116_02010	hoi105_00998	hoi107_02088	hoi109_00069	hoi117_00069	hoi106_01822
389	putative oxidoreductase	16	Core	hoi102_00393	hoi103_00068	hoi104_01963	hoi108_01009	hoi110_00394	hoi111_01918	hoi112_00394	hoi113_00068	hoi114_00394	hoi115_00393	hoi116_02011	hoi105_00999	hoi107_02087	hoi109_00068	hoi117_00068	hoi106_01823
390	Precorrin-6Y C(5,15)-methyltransferase [decarbo	16	Core	hoi102_00394	hoi103_00067	hoi104_01964	hoi108_01010	hoi110_00395	hoi111_01919	hoi112_00395	hoi113_00067	hoi114_00395	hoi115_00394	hoi116_02012	hoi105_01000	hoi107_02086	hoi109_00067	hoi117_00067	hoi106_01824
391	Precorrin-4 C(11)-methyltransferase	16	Core	hoi102_00395	hoi103_00066	hoi104_01965	hoi108_01011	hoi110_00396	hoi111_01920	hoi112_00396	hoi113_00066	hoi114_00396	hoi115_00395	hoi116_02013	hoi105_01001	hoi107_02085	hoi109_00066	hoi117_00066	hoi106_01825
392	Precorrin-6A reductase	16	Core	hoi102_00396	hoi103_00065	hoi104_01966	hoi108_01012	hoi110_00397	hoi111_01921	hoi112_00397	hoi113_00065	hoi114_00397	hoi115_00396	hoi116_02014	hoi105_01002	hoi107_02084	hoi109_00065	hoi117_00065	hoi106_01826
393	hypothetical protein	5	Accessory												hoi105_01003	hoi107_02083	hoi109_00064	hoi117_00064	hoi106_01828
394	hypothetical protein	12	Accessory	hoi102_00397	hoi103_00064	hoi104_01967	hoi108_01013	hoi110_00398	hoi111_01922	hoi112_00398	hoi113_00064	hoi114_00398	hoi115_00397	hoi116_02015					hoi106_01827
395	hypothetical protein	11	Accessory	hoi102_00398	hoi103_00063	hoi104_01968	hoi108_01014	hoi110_00399	hoi111_01923	hoi112_00399	hoi113_00063	hoi114_00399	hoi115_00398	hoi116_02016					
396	hypothetical protein	16	Core	hoi102_00399	hoi103_00062	hoi104_01969	hoi108_01015	hoi110_00400	hoi111_01924	hoi112_00400	hoi113_00062	hoi114_00400	hoi115_00399	hoi116_02017	hoi105_01004	hoi107_02082	hoi109_00063	hoi117_00063	hoi106_01829
397	Precorrin-2 C(20)-methyltransferase	16	Core	hoi102_00400	hoi103_00061	hoi104_01970	hoi108_01016	hoi110_00401	hoi111_01925	hoi112_00401	hoi113_00061	hoi114_00401	hoi115_00400	hoi116_02018	hoi105_01005	hoi107_02081	hoi109_00062	hoi117_00062	hoi106_01830

398	Precorrin-8X methylmutase	16	Core	hoi102_00401	hoi103_00060	hoi104_01971	hoi108_01017	hoi110_00402	hoi111_01926	hoi112_00402	hoi113_00060	hoi114_00402	hoi115_00401	hoi116_02019	hoi105_01006	hoi107_02080	hoi109_00061	hoi117_00061	hoi106_01831
399	ferredoxin-nitrite reductase	16	Core	hoi102_00402	hoi103_00059	hoi104_01972	hoi108_01018	hoi110_00403	hoi111_01927	hoi112_00403	hoi113_00059	hoi114_00403	hoi115_00402	hoi116_02020	hoi105_01007	hoi107_02079	hoi109_00060	hoi117_00060	hoi106_01832
400	hypothetical protein	16	Core	hoi102_00403	hoi103_00058	hoi104_01973	hoi108_01019	hoi110_00404	hoi111_01928	hoi112_00404	hoi113_00058	hoi114_00404	hoi115_00403	hoi116_02021	hoi105_01008	hoi107_02078	hoi109_00059	hoi117_00059	hoi106_01833
401	putative inactive lipase/MT1628	16	Core	hoi102_00404	hoi103_00057	hoi104_01974	hoi108_01020	hoi110_00405	hoi111_01929	hoi112_00405	hoi113_00057	hoi114_00405	hoi115_00404	hoi116_02022	hoi105_01009	hoi107_02077	hoi109_00058	hoi117_00058	hoi106_01834
402	Aerobic cobaltochelatase subunit CobN	16	Core	hoi102_00405	hoi103_00056	hoi104_01975	hoi108_01021	hoi110_00406	hoi111_01930	hoi112_00406	hoi113_00056	hoi114_00406	hoi115_00405	hoi116_02023	hoi105_01010	hoi107_02076	hoi109_00057	hoi117_00057	hoi106_01835
403	phage T7 F exclusion suppressor FxsA	16	Core	hoi102_00406	hoi103_00055	hoi104_01976	hoi108_01022	hoi110_00407	hoi111_01931	hoi112_00407	hoi113_00055	hoi114_00407	hoi115_00406	hoi116_02024	hoi105_01011	hoi107_02075	hoi109_00056	hoi117_00056	hoi106_01836
404	Apolipoprotein N-acyltransferase	16	Core	hoi102_00407	hoi103_00054	hoi104_01977	hoi108_01023	hoi110_00408	hoi111_01932	hoi112_00408	hoi113_00054	hoi114_00408	hoi115_00407	hoi116_02025	hoi105_01012	hoi107_02074	hoi109_00055	hoi117_00055	hoi106_01837
405	Undecaprenyl-phosphate mannosyltransferase	16	Core	hoi102_00408	hoi103_00053	hoi104_01978	hoi108_01024	hoi110_00409	hoi111_01933	hoi112_00409	hoi113_00053	hoi114_00409	hoi115_00408	hoi116_02026	hoi105_01013	hoi107_02073	hoi109_00054	hoi117_00054	hoi106_01838
406	RNA polymerase-binding protein RbpA	16	Core	hoi102_00409	hoi103_00052	hoi104_01979	hoi108_01025	hoi110_00410	hoi111_01934	hoi112_00410	hoi113_00052	hoi114_00410	hoi115_00409	hoi116_02027	hoi105_01014	hoi107_02072	hoi109_00053	hoi117_00053	hoi106_01839
407	hypothetical protein	16	Core	hoi102_00410	hoi103_00051	hoi104_01980	hoi108_01026	hoi110_00411	hoi111_01935	hoi112_00411	hoi113_00051	hoi114_00411	hoi115_00410	hoi116_02028	hoi105_01015	hoi107_02071	hoi109_00052	hoi117_00052	hoi106_01840
408	YceI-like domain protein	16	Core	hoi102_00411	hoi103_00050	hoi104_01981	hoi108_01027	hoi110_00412	hoi111_01936	hoi112_00412	hoi113_00050	hoi114_00412	hoi115_00411	hoi116_02029	hoi105_01016	hoi107_02070	hoi109_00051	hoi117_00051	hoi106_01841
409	hypothetical protein	16	Core	hoi102_00412	hoi103_00049	hoi104_01982	hoi108_01028	hoi110_00413	hoi111_01937	hoi112_00413	hoi113_00049	hoi114_00413	hoi115_00412	hoi116_02030	hoi105_01017	hoi107_02069	hoi109_00050	hoi117_00050	hoi106_01842
410	hypothetical protein	16	Core	hoi102_00413	hoi103_00048	hoi104_01983	hoi108_01029	hoi110_00414	hoi111_01938	hoi112_00414	hoi113_00048	hoi114_00414	hoi115_00413	hoi116_02031	hoi105_01018	hoi107_02068	hoi109_00049	hoi117_00049	hoi106_01843
411	hypothetical protein	16	Core	hoi102_00414	hoi103_00047	hoi104_01984	hoi108_01030	hoi110_00415	hoi111_01939	hoi112_00415	hoi113_00047	hoi114_00415	hoi115_00414	hoi116_02032	hoi105_01019	hoi107_02067	hoi109_00048	hoi117_00048	hoi106_01844
412	hypothetical protein	16	Core	hoi102_00415	hoi103_00046	hoi104_01985	hoi108_01031	hoi110_00416	hoi111_01940	hoi112_00416	hoi113_00046	hoi114_00416	hoi115_00415	hoi116_02033	hoi105_01020	hoi107_02066	hoi109_00047	hoi117_00047	hoi106_01845
413	cyclopropane fatty acyl phospholipid synthase	16	Core	hoi102_00417	hoi103_00044	hoi104_01987	hoi108_01033	hoi110_00418	hoi111_01942	hoi112_00418	hoi113_00044	hoi114_00418	hoi115_00417	hoi116_02035	hoi105_01022	hoi107_02064	hoi109_00045	hoi117_00045	hoi106_01847
414	NADH dehydrogenase-like protein	16	Core	hoi102_00418	hoi103_00043	hoi104_01988	hoi108_01034	hoi110_00419	hoi111_01943	hoi112_00419	hoi113_00043	hoi114_00419	hoi115_00418	hoi116_02036	hoi105_01023	hoi107_02063	hoi109_00044	hoi117_00044	hoi106_01848
415	Formate acetyltransferase 1	16	Core	hoi102_00419	hoi103_00042	hoi104_01989	hoi108_01035	hoi110_00420	hoi111_01944	hoi112_00420	hoi113_00042	hoi114_00420	hoi115_00419	hoi116_02037	hoi105_01024	hoi107_02062	hoi109_00043	hoi117_00043	hoi106_01849
416	Formate acetyltransferase	16	Core	hoi102_00420	hoi103_00041	hoi104_01990	hoi108_01036	hoi110_00421	hoi111_01945	hoi112_00421	hoi113_00041	hoi114_00421	hoi115_00420	hoi116_02038	hoi105_01025	hoi107_02061	hoi109_00042	hoi117_00042	hoi106_01850
417	Pyruvate formate-lyase 1-activating enzyme	16	Core	hoi102_00421	hoi103_00040	hoi104_01991	hoi108_01037	hoi110_00422	hoi111_01946	hoi112_00422	hoi113_00040	hoi114_00422	hoi115_00421	hoi116_02039	hoi105_01026	hoi107_02060	hoi109_00041	hoi117_00041	hoi106_01851
418	Serine transporter	16	Core	hoi102_00422	hoi103_00039	hoi104_01992	hoi108_01038	hoi110_00423	hoi111_01947	hoi112_00423	hoi113_00039	hoi114_00423	hoi115_00422	hoi116_02040	hoi105_01027	hoi107_02059	hoi109_00040	hoi117_00040	hoi106_01852
419	Kynurenine formamidase	16	Core	hoi102_00423	hoi103_00038	hoi104_01993	hoi108_01039	hoi110_00424	hoi111_01948	hoi112_00424	hoi113_00038	hoi114_00424	hoi115_00423	hoi116_02041	hoi105_01029	hoi107_02057	hoi109_00038	hoi117_00038	hoi106_01853
420	Magnesium-protoporphyrin O-methyltransferase	16	Core	hoi102_00424	hoi103_00037	hoi104_01994	hoi108_01040	hoi110_00425	hoi111_01949	hoi112_00425	hoi113_00037	hoi114_00425	hoi115_00424	hoi116_02042	hoi105_01030	hoi107_02056	hoi109_00037	hoi117_00037	hoi106_01854
421	Putative esterase/MT1895	16	Core	hoi102_00425	hoi103_00036	hoi104_01995	hoi108_01041	hoi110_00426	hoi111_01950	hoi112_00426	hoi113_00036	hoi114_00426	hoi115_00425	hoi116_02043	hoi105_01031	hoi107_02055	hoi109_00036	hoi117_00036	hoi106_01855
422	6-phosphogluconate dehydrogenase, NADP(+)-d	16	Core	hoi102_00426	hoi103_00035	hoi104_01996	hoi108_01042	hoi110_00427	hoi111_01951	hoi112_00427	hoi113_00035	hoi114_00427	hoi115_00426	hoi116_02044	hoi105_01032	hoi107_02054	hoi109_00035	hoi117_00035	hoi106_01856
423	ATP-dependent RNA helicase RhlE	16	Core	hoi102_00427	hoi103_00034	hoi104_01997	hoi108_01043	hoi110_00428	hoi111_01952	hoi112_00428	hoi113_00034	hoi114_00428	hoi115_00427	hoi116_02045	hoi105_01033	hoi107_02053	hoi109_00034	hoi117_00034	hoi106_01857
424	Hemolysin C	16	Core	hoi102_00428	hoi103_00033	hoi104_01998	hoi108_01044	hoi110_00429	hoi111_01953	hoi112_00429	hoi113_00033	hoi114_00429	hoi115_00428	hoi116_02046	hoi105_01034	hoi107_02052	hoi109_00033	hoi117_00033	hoi106_01858
425	Hemolysin C	16	Core	hoi102_00429	hoi103_00032	hoi104_01999	hoi108_01045	hoi110_00430	hoi111_01954	hoi112_00430	hoi113_00032	hoi114_00430	hoi115_00429	hoi116_02047	hoi105_01035	hoi107_02051	hoi109_00032	hoi117_00032	hoi106_01859
426	hypothetical protein	16	Core	hoi102_00430	hoi103_00031	hoi104_02000	hoi108_01046	hoi110_00431	hoi111_01955	hoi112_00431	hoi113_00031	hoi114_00431	hoi115_00430	hoi116_02048	hoi105_01036	hoi107_02050	hoi109_00031	hoi117_00031	hoi106_01860
427	hypothetical protein	16	Core	hoi102_00431	hoi103_00030	hoi104_02001	hoi108_01047	hoi110_00432	hoi111_01956	hoi112_00432	hoi113_00030	hoi114_00432	hoi115_00431	hoi116_02049	hoi105_01037	hoi107_02049	hoi109_00030	hoi117_00030	hoi106_01861
428	hypothetical protein	16	Core	hoi102_00432	hoi103_00029	hoi104_02002	hoi108_01048	hoi110_00433	hoi111_01957	hoi112_00433	hoi113_00029	hoi114_00433	hoi115_00432	hoi116_02050	hoi105_01038	hoi107_02048	hoi109_00029	hoi117_00029	hoi106_01862
429	hypothetical protein	16	Core	hoi102_00433	hoi103_00028	hoi104_02003	hoi108_01049	hoi110_00434	hoi111_01958	hoi112_00434	hoi113_00028	hoi114_00434	hoi115_00433	hoi116_02051	hoi105_01039	hoi107_02047	hoi109_00028	hoi117_00028	hoi106_01863
430	zinc-responsive transcriptional regulator	16	Core	hoi102_00434	hoi103_00027	hoi104_02004	hoi108_01050	hoi110_00435	hoi111_01959	hoi112_00435	hoi113_00027	hoi114_00435	hoi115_00434	hoi116_02052	hoi105_01040	hoi107_02046	hoi109_00027	hoi117_00027	hoi106_01864
431	Oxoglutarate dehydrogenase inhibitor	16	Core	hoi102_00435	hoi103_00026	hoi104_02005	hoi108_01051	hoi110_00436	hoi111_01960	hoi112_00436	hoi113_00026	hoi114_00436	hoi115_00435	hoi116_02053	hoi105_01041	hoi107_02045	hoi109_00026	hoi117_00026	hoi106_01865
432	preprotein translocase subunit SecA	16	Core	hoi102_00436	hoi103_00025	hoi104_02006	hoi108_01052	hoi110_00437	hoi111_01961	hoi112_00437	hoi113_00025	hoi114_00437	hoi115_00436	hoi116_02054	hoi105_01043	hoi107_02043	hoi109_00024	hoi117_00024	hoi106_01866
433	hypothetical protein	11	Accessory	hoi102_00437	hoi103_00024	hoi104_02007	hoi108_01053	hoi110_00438	hoi111_01962	hoi112_00438	hoi113_00024	hoi114_00438	hoi115_00437	hoi116_02055					
434	Alpha/beta hydrolase family protein	16	Core	hoi102_00438	hoi103_00023	hoi104_02008	hoi108_01054	hoi110_00439	hoi111_01963	hoi112_00439	hoi113_00023	hoi114_00439	hoi115_00438	hoi116_02056	hoi105_01044	hoi107_02042	hoi109_00023	hoi117_00023	hoi106_01867
435	hypothetical protein	16	Core	hoi102_00439	hoi103_00022	hoi104_02009	hoi108_01055	hoi110_00440	hoi111_01964	hoi112_00440	hoi113_00022	hoi114_00440	hoi115_00439	hoi116_02057	hoi105_01045	hoi107_02041	hoi109_00022	hoi117_00022	hoi106_01868
436	hypothetical protein	16	Core	hoi102_00441	hoi103_00020	hoi104_02011	hoi108_01057	hoi110_00442	hoi111_01966	hoi112_00442	hoi113_00020	hoi114_00442	hoi115_00441	hoi116_02059	hoi105_01047	hoi107_02039	hoi109_00020	hoi117_00020	hoi106_01872
437	GTPase Der	16	Core	hoi102_00442	hoi103_00019	hoi104_02012	hoi108_01058	hoi110_00443	hoi111_01967	hoi112_00443	hoi113_00019	hoi114_00443	hoi115_00442	hoi116_02060	hoi105_01048	hoi107_02038	hoi109_00019	hoi117_00019	hoi106_01873
438	Cytidylate kinase	16	Core	hoi102_00443	hoi103_00018	hoi104_02013	hoi108_01059	hoi110_00444	hoi111_01968	hoi112_00444	hoi113_00018	hoi114_00444	hoi115_00443	hoi116_02061	hoi105_01049	hoi107_02037	hoi109_00018	hoi117_00018	hoi106_01874
439	Ribosomal large subunit pseudouridine synthase	16	Core	hoi102_00444	hoi103_00017	hoi104_02014	hoi108_01060	hoi110_00445	hoi111_01969	hoi112_00445	hoi113_00017	hoi114_00445	hoi115_00444	hoi116_02062	hoi105_01050	hoi107_02036	hoi109_00017	hoi117_00017	hoi106_01875
440	ATP-dependent dethiobiotin synthetase BioD	16	Core	hoi102_00445	hoi103_00016	hoi104_02015	hoi108_01061	hoi110_00446	hoi111_01970	hoi112_00446	hoi113_00016	hoi114_00446	hoi115_00445	hoi116_02063	hoi105_01051	hoi107_02035	hoi109_00016	hoi117_00016	hoi106_01876
441	Adenosylmethionine-8-amino-7-oxononanoate a	16	Core	hoi102_00446	hoi103_00015	hoi104_02016	hoi108_01062	hoi110_00447	hoi111_01971	hoi112_00447	hoi113_00015	hoi114_00447	hoi115_00446	hoi116_02064	hoi105_01052	hoi107_02034	hoi109_00015	hoi117_00015	hoi106_01877
442	Segregation and condensation protein B	16	Core	hoi102_00447	hoi103_00014	hoi104_02017	hoi108_01063	hoi110_00448	hoi111_01972	hoi112_00448	hoi113_00014	hoi114_00448	hoi115_00447	hoi116_02065	hoi105_01053	hoi107_02033	hoi109_00014	hoi117_00014	hoi106_01878
443	3-mercaptopyruvate sulfurtransferase	16	Core	hoi102_00448	hoi103_00013	hoi104_02018	hoi108_01064	hoi110_00449	hoi111_01973	hoi112_00449	hoi113_00013	hoi114_00449	hoi115_00448	hoi116_02066	hoi105_01054	hoi107_02032	hoi109_00013	hoi117_00013	hoi106_01879
444	Segregation and condensation protein A	16	Core	hoi102_00449	hoi103_00012	hoi104_02019	hoi108_01065	hoi110_00450	hoi111_01974	hoi112_00450	hoi113_00012	hoi114_00450	hoi115_00449	hoi116_02067	hoi105_01055	hoi107_02031	hoi109_00012	hoi117_00012	hoi106_01880
445	Sporulation initiation inhibitor protein Soj	16	Core	hoi102_00450	hoi103_00011	hoi104_02020	hoi108_01066	hoi110_00451	hoi111_01975	hoi112_00451	hoi113_00011	hoi114_00451	hoi115_00450	hoi116_02068	hoi105_01056	hoi107_02030	hoi109_00011	hoi117_00011	hoi106_01881
446	Tyrosine recombinase XerD	16	Core	hoi102_00451	hoi103_00010	hoi104_02021	hoi108_01067	hoi110_00452	hoi111_01976	hoi112_00452	hoi113_00010	hoi114_00452	hoi115_00451	hoi116_02069	hoi105_01057	hoi107_02029	hoi109_00010	hoi117_00010	hoi106_01882
447	ADP-ribose pyrophosphatase	16	Core	hoi102_00452	hoi103_00009	hoi104_02022	hoi108_01068	hoi110_00453	hoi111_01977	hoi112_00453	hoi113_00009	hoi114_00453	hoi115_00452	hoi116_02070	hoi105_01058	hoi107_02028	hoi109_00009	hoi117_00009	hoi106_01883
448	Copper transporter MctB precursor	16	Core	hoi102_00453	hoi103_00008	hoi104_02023	hoi108_01069	hoi110_00454	hoi111_01978	hoi112_00454	hoi113_00008	hoi114_00454	hoi115_00453	hoi116_02071	hoi105_01059	hoi107_02027	hoi109_00008	hoi117_00008	hoi106_01884
449	hypothetical protein	16	Core	hoi102_00454	hoi103_00007	hoi104_02024	hoi108_01070	hoi110_00455	hoi111_01979	hoi112_00455	hoi113_00007	hoi114_00455	hoi115_00454	hoi116_02072	hoi105_01060	hoi107_02026	hoi109_00007	hoi117_00007	hoi106_01885
450	DNA repair protein RecN	16	Core	hoi102_00455	hoi103_00006	hoi104_02025	hoi108_01071	hoi110_00456	hoi111_01980	hoi112_00456	hoi113_00006	hoi114_00456	hoi115_00455	hoi116_02073	hoi105_01061	hoi107_02025	hoi109_00006	hoi117_00006	hoi106_01886
451	Inorganic polyphosphate/ATP-NAD kinase	16	Core	hoi102_00456	hoi103_00005	hoi104_02026	hoi108_01072	hoi110_00457	hoi111_01981	hoi112_00457	hoi113_00005	hoi114_00457	hoi115_00456	hoi116_02074	hoi105_01062	hoi107_02024	hoi109_00005	hoi117_00005	hoi106_01887
452	16S/23S rRNA (cytidine-2'-O)-methyltransferase	16	Core	hoi102_00457	hoi103_00004	hoi104_02027	hoi108_01073	hoi110_00458	hoi111_01982	hoi112_00458	hoi113_00004	hoi114_00458	hoi115_00457	hoi116_02075	hoi105_01063	hoi107_02023	hoi109_00004	hoi117_00004	hoi106_01888
453	hypothetical protein	16	Core	hoi102_00458	hoi103_00003	hoi104_02028	hoi108_01074	hoi110_00459	hoi111_01983	hoi112_00459	hoi113_00003	hoi114_00459	hoi115_00458	hoi116_02076	hoi105_01064	hoi107_02022	hoi109_00003	hoi117_00003	hoi106_01889
454	putative hydrolase YutF	16	Core	hoi102_00459	hoi103_00002	hoi104_02029	hoi108_01075	hoi110_00460	hoi111_01984	hoi112_00460	hoi113_00002	hoi114_00460	hoi115_00459	hoi116_02077	hoi105_01065	hoi107_02021	hoi109_00002	hoi117_00002	hoi106_01890
455	Tetratricopeptide repeat protein	16	Core	hoi102_00460	hoi103_00001	hoi104_02030	hoi108_01076	hoi110_00461	hoi111_01985	hoi112_00461	hoi113_00001	hoi114_00461	hoi115_00460	hoi116_02078	hoi105_01066	hoi107_02020	hoi109_00001	hoi117_00001	hoi106_01891
456	UDP-N-acetylglucosamine 1-carboxyvinyltransfe	16	Core	hoi102_00461	hoi103_02120	hoi104_00376	hoi108_00411	hoi110_01879	hoi111_01677	hoi112_00743	hoi113_00885	hoi114_02120	hoi115_00461	hoi116_01817	hoi105_01710	hoi107_01756	hoi109_02222	hoi117_01947	hoi106_01291
457	Cob(I)yrinic acid a,c-diamide adenosyltransferase	16	Core	hoi102_00462	hoi103_02121	hoi104_00377	hoi108_00412	hoi110_01878	hoi111_01676	hoi112_00744	hoi113_00886	hoi114_02119	hoi115_00462	hoi116_01818	hoi105_01711	hoi107_01757	hoi109_02223	hoi117_01948	hoi106_01292
458	transcriptional regulator MalT	16	Core	hoi102_00463	hoi103_02122	hoi104_00378	hoi108_00413	hoi110_01877	hoi111_01675	hoi112_00745	hoi113_00887	hoi114_02118	hoi115_00463	hoi116_01819	hoi105_01712	hoi107_01758	hoi109_02224	hoi117_01949	hoi106_01293
459	Cobalt import ATP-binding protein CbiO	16	Core	hoi102_00464	hoi103_00461	hoi104_00379	hoi108_00414	hoi110_01876	hoi111_01674	hoi112_00746	hoi113_00888	hoi114_01704	hoi115_00464	hoi116_01820	hoi105_01713	hoi107_01759	hoi109_01801	hoi117_01950	hoi106_01294
460	Cobalt transport protein CbiQ	16	Core	hoi102_00465	hoi103_00462	hoi104_00380	hoi108_00415	hoi110_01875	hoi111_01673	hoi112_00747	hoi113_00889	hoi114_01705	hoi115_00465	hoi116_01821	hoi105_01714	hoi107_01760	hoi109_01800	hoi117_01951	hoi106_01295
461	Cobalt transport protein CbiN	16	Core	hoi102_00466	hoi103_00463	hoi104_00381	hoi108_00416	hoi110_01874	hoi111_01672	hoi112_00748	hoi113_00890	hoi114_01706	hoi115_00466	hoi116_01822	hoi105_01715	hoi107_01761	hoi109_01799	hoi117_01952	hoi106_01296
462	Cobalt transport protein CbiM	16	Core	hoi102_00467	hoi103_00464	hoi104_00382	hoi108_00417	hoi110_01873	hoi111_01671	hoi112_00749	hoi113_00891	hoi114_01707	hoi115_00467	hoi116_01823	hoi105_01716	hoi107_01762	hoi109_01798	hoi117_01953	hoi106_01297
463	O-acetylserine sulfhydrylase	16	Core	hoi102_00468	hoi103_00465	hoi104_00383	hoi108_00418	hoi110_01872	hoi111_01670	hoi112_00750	hoi113_00892	hoi114_01708	hoi115_00468	hoi116_01824	hoi105_01717	hoi107_01763	hoi109_01797	hoi117_01954	hoi106_01298
464	Serine acetyltransferase	16	Core	hoi102_00469	hoi103_00466	hoi104_00384	hoi108_00419	hoi110_01871	hoi111_01669	hoi112_00751	hoi113_00893	hoi114_01709	hoi115_00469	hoi116_01825	hoi105_01718	hoi107_01764	hoi109_01796	hoi117_01955	hoi106_01299
465	hypothetical protein	16	Core	hoi102_00470	hoi103_00467	hoi104_00385	hoi108_00420	hoi110_01870	hoi111_01668	hoi112_00752	hoi113_00894	hoi114_01710	hoi115_00470	hoi116_01826	hoi105_01719	hoi107_01765	hoi109_01795	hoi117_01956	hoi106_01300
466	Putative membrane protein YdgH	16	Core	hoi102_00475	hoi103_00472	hoi104_00390	hoi108_00425	hoi110_01865	hoi111_01663	hoi112_00757	hoi113_00899	hoi114_01715	hoi115_00475	hoi116_01831	hoi105_01724	hoi107_01770	hoi109_01790	hoi117_01961	hoi106_01305
467	Succinyl-CoA:coenzyme A transferase	16	Core	hoi102_00477	hoi103_00474	hoi104_00392	hoi108_00427	hoi110_01863	hoi111_01661	hoi112_00759	hoi113_00901	hoi114_01717	hoi115_00477	hoi116_01833	hoi105_01726	hoi107_01772	hoi109_01788	hoi117_01963	hoi106_01307
468	putative tRNA-dihydrouridine synthase	16	Core	hoi102_00478	hoi103_00475	hoi104_00393	hoi108_00428	hoi110_01862	hoi111_01660	hoi112_00760	hoi113_00902	hoi114_01718	hoi115_00478	hoi116_01834	hoi105_01727	hoi107_01773	hoi109_01787	hoi117_01964	hoi106_01308
469	hypothetical protein	16	Core	hoi102_00479	hoi103_00476	hoi104_00394	hoi108_00429	hoi110_01861	hoi111_01659	hoi112_00761	hoi113_00903	hoi114_01719	hoi115_00479	hoi116_01835	hoi105_01728	hoi107_01774	hoi109_01786	hoi117_01965	hoi106_01309
470	Phosphate import ATP-binding protein PstB 3	16	Core	hoi102_00480	hoi103_00477	hoi104_00395	hoi108_00430	hoi110_01860	hoi111_01658	hoi112_00762	hoi113_00904	hoi114_01720	hoi115_00480	hoi116_01836	hoi105_01729	hoi107_01775	hoi109_01785	hoi117_01966	hoi106_01310
471	Phosphate transport system permease protein P	16	Core	hoi102_00481	hoi103_00478	hoi104_00396	hoi108_00431	hoi110_01859	hoi111_01657	hoi112_00763	hoi113_00905	hoi114_01721	hoi115_00481	hoi116_01837	hoi105_01730	hoi107_01776	hoi109_01784	hoi117_01967	hoi106_01311
472	Phosphate transport system permease protein P	16	Core	hoi102_00482	hoi103_00479	hoi104_00397	hoi108_00432	hoi110_01858	hoi111_01656	hoi112_00764	hoi113_00906	hoi114_01722	hoi115_00482	hoi116_01838	hoi105_01731	hoi107_01777	hoi109_01783	hoi117_01968	hoi106_01312
473	Phosphate-binding protein PstS 3 precursor	16	Core	hoi102_00483	hoi103_00480	hoi104_00398	hoi108_00433	hoi110_01857	hoi111_01655	hoi112_00765	hoi113_00907	hoi114_01723	hoi115_00483	hoi116_01839	hoi105_01732	hoi107_01778	hoi109_01782	hoi117_01969	hoi106_01313
474	Mycothiol acetyltransferase	16	Core	hoi102_00484	hoi103_00481	hoi104_00399	hoi108_00434	hoi110_01856	hoi111_01654	hoi112_00766	hoi113_00908	hoi114_01724	hoi115_00484	hoi116_01840	hoi105_01733	hoi107_01779	hoi109_01781	hoi117_01970	hoi106_01314
475	hypothetical protein	16	Core	hoi102_00485	hoi103_00482	hoi104_00400	hoi108_00435	hoi110_01855	hoi111_01653	hoi112_00767	hoi113_00909	hoi114_01725	hoi115_00485	hoi116_01841	hoi105_01734	hoi107_01780	hoi109_01780	hoi117_01971	hoi106_01315
476	hypothetical protein	16	Core	hoi102_00486	hoi103_00483	hoi104_00401	hoi108_00436	hoi110_01854	hoi111_01652	hoi112_00768	hoi113_00910	hoi114_01726	hoi115_00486	hoi116_01842	hoi105_01735	hoi107_01781	hoi109_01779	hoi117_01972	hoi106_01316
477	D-alanine aminotransferase	16	Core	hoi102_00487	hoi103_00484	hoi104_00402	hoi108_00437	hoi110_01853	hoi111_01651	hoi112_00769	hoi113_00911	hoi114_01727	hoi115_00487	hoi116_01843	hoi105_01736	hoi107_01782	hoi109_01778	hoi117_01973	hoi106_01317

478	putative deferrochelatase/peroxidase YfeX	16	Core	hoi102_00488	hoi103_00485	hoi104_00403	hoi108_00438	hoi110_01852	hoi111_01650	hoi112_00770	hoi113_00912	hoi114_01728	hoi115_00488	hoi116_01844	hoi105_01737	hoi107_01783	hoi109_01777	hoi117_01974	hoi106_01318
479	putative global regulator	16	Core	hoi102_00489	hoi103_00486	hoi104_00404	hoi108_00439	hoi110_01851	hoi111_01649	hoi112_00771	hoi113_00913	hoi114_01729	hoi115_00489	hoi116_01845	hoi105_01738	hoi107_01784	hoi109_01776	hoi117_01975	hoi106_01319
480	hypothetical protein	16	Core	hoi102_00490	hoi103_00487	hoi104_00405	hoi108_00440	hoi110_01850	hoi111_01648	hoi112_00772	hoi113_00914	hoi114_01730	hoi115_00490	hoi116_01846	hoi105_01739	hoi107_01785	hoi109_01775	hoi117_01976	hoi106_01320
481	Phosphoribosylformylglycinamidine cyclo-ligase	16	Core	hoi102_00491	hoi103_00488	hoi104_00406	hoi108_00441	hoi110_01849	hoi111_01647	hoi112_00773	hoi113_00915	hoi114_01731	hoi115_00491	hoi116_01847	hoi105_01740	hoi107_01786	hoi109_01774	hoi117_01977	hoi106_01321
482	Amidophosphoribosyltransferase precursor	16	Core	hoi102_00492	hoi103_00489	hoi104_00407	hoi108_00442	hoi110_01848	hoi111_01646	hoi112_00774	hoi113_00916	hoi114_01732	hoi115_00492	hoi116_01848	hoi105_01741	hoi107_01787	hoi109_01773	hoi117_01978	hoi106_01322
483	hypothetical protein	16	Core	hoi102_00493	hoi103_00490	hoi104_00408	hoi108_00443	hoi110_01847	hoi111_01645	hoi112_00775	hoi113_00917	hoi114_01733	hoi115_00493	hoi116_01849	hoi105_01742	hoi107_01788	hoi109_01772	hoi117_01979	hoi106_01323
484	putative acyl-CoA thioester hydrolase	16	Core	hoi102_00494	hoi103_00491	hoi104_00409	hoi108_00444	hoi110_01846	hoi111_01644	hoi112_00776	hoi113_00918	hoi114_01734	hoi115_00494	hoi116_01850	hoi105_01743	hoi107_01789	hoi109_01771	hoi117_01980	hoi106_01324
485	Transcriptional regulatory protein DevR (DosR)	16	Core	hoi102_00495	hoi103_00492	hoi104_00410	hoi108_00445	hoi110_01845	hoi111_01643	hoi112_00777	hoi113_00919	hoi114_01735	hoi115_00495	hoi116_01851	hoi105_01744	hoi107_01790	hoi109_01770	hoi117_01981	hoi106_01325
486	Sensor histidine kinase LiaS	16	Core	hoi102_00496	hoi103_00493	hoi104_00411	hoi108_00446	hoi110_01844	hoi111_01642	hoi112_00778	hoi113_00920	hoi114_01736	hoi115_00496	hoi116_01852	hoi105_01745	hoi107_01791	hoi109_01769	hoi117_01982	hoi106_01326
487	Membrane protein YdfJ	16	Core	hoi102_00497	hoi103_00494	hoi104_00412	hoi108_00447	hoi110_01843	hoi111_01641	hoi112_00779	hoi113_00921	hoi114_01737	hoi115_00497	hoi116_01853	hoi105_01746	hoi107_01792	hoi109_01768	hoi117_01983	hoi106_01327
488	Phosphoribosylformylglycinamidine synthase 2	16	Core	hoi102_00498	hoi103_00495	hoi104_00413	hoi108_00448	hoi110_01842	hoi111_01640	hoi112_00780	hoi113_00922	hoi114_01738	hoi115_00498	hoi116_01854	hoi105_01747	hoi107_01793	hoi109_01767	hoi117_01984	hoi106_01328
489	Phosphoribosylformylglycinamidine synthase 1	16	Core	hoi102_00499	hoi103_00496	hoi104_00414	hoi108_00449	hoi110_01841	hoi111_01639	hoi112_00781	hoi113_00923	hoi114_01739	hoi115_00499	hoi116_01855	hoi105_01748	hoi107_01794	hoi109_01766	hoi117_01985	hoi106_01329
490	phosphoribosylformylglycinamidine synthase sul	16	Core	hoi102_00500	hoi103_00497	hoi104_00415	hoi108_00450	hoi110_01840	hoi111_01638	hoi112_00782	hoi113_00924	hoi114_01740	hoi115_00500	hoi116_01856	hoi105_01749	hoi107_01795	hoi109_01765	hoi117_01986	hoi106_01330
491	Alpha/beta hydrolase family protein	16	Core	hoi102_00501	hoi103_00498	hoi104_00416	hoi108_00451	hoi110_01839	hoi111_01637	hoi112_00783	hoi113_00925	hoi114_01741	hoi115_00501	hoi116_01857	hoi105_01750	hoi107_01796	hoi109_01764	hoi117_01987	hoi106_01331
492	hypothetical protein	16	Core	hoi102_00502	hoi103_00499	hoi104_00417	hoi108_00452	hoi110_01838	hoi111_01636	hoi112_00784	hoi113_00926	hoi114_01742	hoi115_00502	hoi116_01858	hoi105_01751	hoi107_01797	hoi109_01763	hoi117_01988	hoi106_01332
493	GMP synthase [glutamine-hydrolyzing]	16	Core	hoi102_00503	hoi103_00500	hoi104_00418	hoi108_00453	hoi110_01837	hoi111_01635	hoi112_00785	hoi113_00927	hoi114_01743	hoi115_00503	hoi116_01859	hoi105_01752	hoi107_01798	hoi109_01762	hoi117_01989	hoi106_01333
494	hypothetical protein	16	Core	hoi102_00504	hoi103_00501	hoi104_00419	hoi108_00454	hoi110_01836	hoi111_01634	hoi112_00786	hoi113_00928	hoi114_01744	hoi115_00504	hoi116_01860	hoi105_01753	hoi107_01799	hoi109_01761	hoi117_01990	hoi106_01334
495	Cupin domain protein	16	Core	hoi102_00505	hoi103_00502	hoi104_00420	hoi108_00455	hoi110_01835	hoi111_01633	hoi112_00787	hoi113_00929	hoi114_01745	hoi115_00505	hoi116_01861	hoi105_01754	hoi107_01800	hoi109_01760	hoi117_01991	hoi106_01335
496	Pantothenate synthetase	16	Core	hoi102_00506	hoi103_00503	hoi104_00421	hoi108_00456	hoi110_01834	hoi111_01632	hoi112_00788	hoi113_00930	hoi114_01746	hoi115_00506	hoi116_01862	hoi105_01755	hoi107_01801	hoi109_01759	hoi117_01992	hoi106_01336
497	hypothetical protein	16	Core	hoi102_00507	hoi103_00504	hoi104_00422	hoi108_00457	hoi110_01833	hoi111_01631	hoi112_00789	hoi113_00931	hoi114_01747	hoi115_00507	hoi116_01863	hoi105_01756	hoi107_01802	hoi109_01758	hoi117_01993	hoi106_01337
498	hypothetical protein	16	Core	hoi102_00508	hoi103_00505	hoi104_00423	hoi108_00458	hoi110_01832	hoi111_01630	hoi112_00790	hoi113_00932	hoi114_01748	hoi115_00508	hoi116_01864	hoi105_01757	hoi107_01803	hoi109_01757	hoi117_01994	hoi106_01338
499	2-amino-4-hydroxy-6-hydroxymethyldihydropter	16	Core	hoi102_00509	hoi103_00506	hoi104_00424	hoi108_00459	hoi110_01831	hoi111_01629	hoi112_00791	hoi113_00933	hoi114_01749	hoi115_00509	hoi116_01865	hoi105_01758	hoi107_01804	hoi109_01756	hoi117_01995	hoi106_01339
500	putative dihydroneopterin aldolase	16	Core	hoi102_00510	hoi103_00507	hoi104_00425	hoi108_00460	hoi110_01830	hoi111_01628	hoi112_00792	hoi113_00934	hoi114_01750	hoi115_00510	hoi116_01866	hoi105_01759	hoi107_01805	hoi109_01755	hoi117_01996	hoi106_01340
501	Dihydropteroate synthase 1	16	Core	hoi102_00511	hoi103_00508	hoi104_00426	hoi108_00461	hoi110_01829	hoi111_01627	hoi112_00793	hoi113_00935	hoi114_01751	hoi115_00511	hoi116_01867	hoi105_01760	hoi107_01806	hoi109_01754	hoi117_01997	hoi106_01341
502	GTP cyclohydrolase 1	16	Core	hoi102_00512	hoi103_00509	hoi104_00427	hoi108_00462	hoi110_01828	hoi111_01626	hoi112_00794	hoi113_00936	hoi114_01752	hoi115_00512	hoi116_01868	hoi105_01761	hoi107_01807	hoi109_01753	hoi117_01998	hoi106_01342
503	ATP-dependent zinc metalloprotease FtsH	16	Core	hoi102_00513	hoi103_00510	hoi104_00428	hoi108_00463	hoi110_01827	hoi111_01625	hoi112_00795	hoi113_00937	hoi114_01753	hoi115_00513	hoi116_01869	hoi105_01762	hoi107_01808	hoi109_01752	hoi117_01999	hoi106_01343
504	Hypoxanthine phosphoribosyltransferase	16	Core	hoi102_00514	hoi103_00511	hoi104_00429	hoi108_00464	hoi110_01826	hoi111_01624	hoi112_00796	hoi113_00938	hoi114_01754	hoi115_00514	hoi116_01870	hoi105_01763	hoi107_01809	hoi109_01751	hoi117_02000	hoi106_01344
505	tRNA(Ile)-lysidine synthase	16	Core	hoi102_00515	hoi103_00512	hoi104_00430	hoi108_00465	hoi110_01825	hoi111_01623	hoi112_00797	hoi113_00939	hoi114_01755	hoi115_00515	hoi116_01871	hoi105_01764	hoi107_01810	hoi109_01750	hoi117_02001	hoi106_01345
506	D-alanyl-D-alanine carboxypeptidase DacC precu	16	Core	hoi102_00516	hoi103_00513	hoi104_00431	hoi108_00466	hoi110_01824	hoi111_01622	hoi112_00798	hoi113_00940	hoi114_01756	hoi115_00516	hoi116_01872	hoi105_01765	hoi107_01811	hoi109_01749	hoi117_02002	hoi106_01346
507	Inorganic pyrophosphatase	16	Core	hoi102_00517	hoi103_00514	hoi104_00432	hoi108_00467	hoi110_01823	hoi111_01621	hoi112_00799	hoi113_00941	hoi114_01757	hoi115_00517	hoi116_01873	hoi105_01766	hoi107_01812	hoi109_01748	hoi117_02003	hoi106_01347
508	transcriptional regulator SlyA	16	Core	hoi102_00518	hoi103_00515	hoi104_00433	hoi108_00468	hoi110_01822	hoi111_01620	hoi112_00800	hoi113_00942	hoi114_01758	hoi115_00518	hoi116_01874	hoi105_01767	hoi107_01813	hoi109_01747	hoi117_02004	hoi106_01348
509	Tyrocidine synthase 3	16	Core	hoi102_00519	hoi103_00516	hoi104_00434	hoi108_00469	hoi110_01821	hoi111_01619	hoi112_00801	hoi113_00943	hoi114_01759	hoi115_00519	hoi116_01875	hoi105_01768	hoi107_01814	hoi109_01746	hoi117_02005	hoi106_01349
510	Polyphosphate kinase 2 (PPK2)	16	Core	hoi102_00520	hoi103_00517	hoi104_00435	hoi108_00470	hoi110_01820	hoi111_01618	hoi112_00802	hoi113_00944	hoi114_01760	hoi115_00520	hoi116_01876	hoi105_01769	hoi107_01815	hoi109_01745	hoi117_02006	hoi106_01350
511	Rossmann-like domain protein	16	Core	hoi102_00521	hoi103_00518	hoi104_00436	hoi108_00471	hoi110_01819	hoi111_01617	hoi112_00803	hoi113_00945	hoi114_01761	hoi115_00521	hoi116_01877	hoi105_01770	hoi107_01816	hoi109_01744	hoi117_02007	hoi106_01351
512	60 kDa chaperonin 1	16	Core	hoi102_00522	hoi103_00519	hoi104_00437	hoi108_00472	hoi110_01818	hoi111_01616	hoi112_00804	hoi113_00946	hoi114_01762	hoi115_00522	hoi116_01878	hoi105_01771	hoi107_01817	hoi109_01743	hoi117_02008	hoi106_01352
513	putative succinyl-diaminopimelate desuccinylase	16	Core	hoi102_00523	hoi103_00520	hoi104_00438	hoi108_00473	hoi110_01817	hoi111_01615	hoi112_00805	hoi113_00947	hoi114_01763	hoi115_00523	hoi116_01879	hoi105_01772	hoi107_01818	hoi109_01742	hoi117_02009	hoi106_01353
514	hypothetical protein	11	Accessory	hoi102_00524	hoi103_00521	hoi104_00439	hoi108_00474	hoi110_01816	hoi111_01614	hoi112_00806	hoi113_00948	hoi114_01764	hoi115_00524	hoi116_01880					
515	hypothetical protein	16	Core	hoi102_00525	hoi103_00522	hoi104_00440	hoi108_00475	hoi110_01815	hoi111_01613	hoi112_00807	hoi113_00949	hoi114_01765	hoi115_00525	hoi116_01881	hoi105_01773	hoi107_01819	hoi109_01741	hoi117_02010	hoi106_01354
516	hypothetical protein	12	Accessory	hoi102_00526	hoi103_00523	hoi104_00441	hoi108_00476	hoi110_01814	hoi111_01612	hoi112_00808	hoi113_00950	hoi114_01766	hoi115_00526	hoi116_01882					hoi106_01355
517	Na(+)/H(+) antiporter subunit A	16	Core	hoi102_00527	hoi103_00524	hoi104_00442	hoi108_00477	hoi110_01813	hoi111_01611	hoi112_00809	hoi113_00951	hoi114_01767	hoi115_00527	hoi116_01883	hoi105_01774	hoi107_01820	hoi109_01740	hoi117_02011	hoi106_01356
518	Na(+)/H(+) antiporter subunit C	16	Core	hoi102_00528	hoi103_00525	hoi104_00443	hoi108_00478	hoi110_01812	hoi111_01610	hoi112_00810	hoi113_00952	hoi114_01768	hoi115_00528	hoi116_01884	hoi105_01775	hoi107_01821	hoi109_01739	hoi117_02012	hoi106_01357
519	Na(+)/H(+) antiporter subunit D	16	Core	hoi102_00529	hoi103_00526	hoi104_00444	hoi108_00479	hoi110_01811	hoi111_01609	hoi112_00811	hoi113_00953	hoi114_01769	hoi115_00529	hoi116_01885	hoi105_01776	hoi107_01822	hoi109_01738	hoi117_02013	hoi106_01358
520	Na(+)/H(+) antiporter subunit E	16	Core	hoi102_00530	hoi103_00527	hoi104_00445	hoi108_00480	hoi110_01810	hoi111_01608	hoi112_00812	hoi113_00954	hoi114_01770	hoi115_00530	hoi116_01886	hoi105_01777	hoi107_01823	hoi109_01737	hoi117_02014	hoi106_01359
521	putative monovalent cation/H+ antiporter subun	16	Core	hoi102_00531	hoi103_00528	hoi104_00446	hoi108_00481	hoi110_01809	hoi111_01607	hoi112_00813	hoi113_00955	hoi114_01771	hoi115_00531	hoi116_01887	hoi105_01778	hoi107_01824	hoi109_01736	hoi117_02015	hoi106_01360
522	Na(+)/H(+) antiporter subunit G	16	Core	hoi102_00532	hoi103_00529	hoi104_00447	hoi108_00482	hoi110_01808	hoi111_01606	hoi112_00814	hoi113_00956	hoi114_01772	hoi115_00532	hoi116_01888	hoi105_01779	hoi107_01825	hoi109_01735	hoi117_02016	hoi106_01361
523	Carboxylate-amine ligase YbdK	16	Core	hoi102_00533	hoi103_00530	hoi104_00448	hoi108_00483	hoi110_01807	hoi111_01605	hoi112_00815	hoi113_00957	hoi114_01773	hoi115_00533	hoi116_01889	hoi105_01780	hoi107_01826	hoi109_01734	hoi117_02017	hoi106_01362
524	hypothetical protein	16	Core	hoi102_00534	hoi103_00531	hoi104_00449	hoi108_00484	hoi110_01806	hoi111_01604	hoi112_00816	hoi113_00958	hoi114_01774	hoi115_00534	hoi116_01890	hoi105_01781	hoi107_01827	hoi109_01733	hoi117_02018	hoi106_01363
525	hypothetical protein	16	Core	hoi102_00535	hoi103_00532	hoi104_00450	hoi108_00485	hoi110_01805	hoi111_01603	hoi112_00817	hoi113_00959	hoi114_01775	hoi115_00535	hoi116_01891	hoi105_01782	hoi107_01828	hoi109_01732	hoi117_02019	hoi106_01364
526	Peptide deformylase	16	Core	hoi102_00536	hoi103_00533	hoi104_00451	hoi108_00486	hoi110_01804	hoi111_01602	hoi112_00818	hoi113_00960	hoi114_01776	hoi115_00536	hoi116_01892	hoi105_01783	hoi107_01829	hoi109_01731	hoi117_02020	hoi106_01365
527	Mycothiol acetyltransferase	16	Core	hoi102_00537	hoi103_00534	hoi104_00452	hoi108_00487	hoi110_01803	hoi111_01601	hoi112_00819	hoi113_00961	hoi114_01777	hoi115_00537	hoi116_01893	hoi105_01784	hoi107_01830	hoi109_01730	hoi117_02021	hoi106_01366
528	RDD family protein	16	Core	hoi102_00538	hoi103_00535	hoi104_00453	hoi108_00488	hoi110_01802	hoi111_01600	hoi112_00820	hoi113_00962	hoi114_01778	hoi115_00538	hoi116_01894	hoi105_01785	hoi107_01831	hoi109_01729	hoi117_02022	hoi106_01367
529	Exodeoxyribonuclease III	16	Core	hoi102_00539	hoi103_00536	hoi104_00454	hoi108_00489	hoi110_01801	hoi111_01599	hoi112_00821	hoi113_00963	hoi114_01779	hoi115_00539	hoi116_01895	hoi105_01786	hoi107_01832	hoi109_01728	hoi117_02023	hoi106_01368
530	Cardiolipin synthase	16	Core	hoi102_00540	hoi103_00537	hoi104_00455	hoi108_00490	hoi110_01800	hoi111_01598	hoi112_00822	hoi113_00964	hoi114_01780	hoi115_00540	hoi116_01896	hoi105_01787	hoi107_01833	hoi109_01727	hoi117_02024	hoi106_01369
531	Na+/Pi-cotransporter	16	Core	hoi102_00541	hoi103_00538	hoi104_00456	hoi108_00491	hoi110_01799	hoi111_01597	hoi112_00823	hoi113_00965	hoi114_01781	hoi115_00541	hoi116_01897	hoi105_01788	hoi107_01834	hoi109_01726	hoi117_02025	hoi106_01370
532	ABC-2 family transporter protein	16	Core	hoi102_00542	hoi103_00539	hoi104_00457	hoi108_00492	hoi110_01798	hoi111_01596	hoi112_00824	hoi113_00966	hoi114_01782	hoi115_00542	hoi116_01898	hoi105_01789	hoi107_01835	hoi109_01725	hoi117_02026	hoi106_01371
533	putative ABC transporter ATP-binding protein Yxl	16	Core	hoi102_00543	hoi103_00540	hoi104_00458	hoi108_00493	hoi110_01797	hoi111_01595	hoi112_00825	hoi113_00967	hoi114_01783	hoi115_00543	hoi116_01899	hoi105_01790	hoi107_01836	hoi109_01724	hoi117_02027	hoi106_01372
534	putative NUDIX hydrolase	16	Core	hoi102_00544	hoi103_00541	hoi104_00459	hoi108_00494	hoi110_01796	hoi111_01594	hoi112_00826	hoi113_00968	hoi114_01784	hoi115_00544	hoi116_01900	hoi105_01791	hoi107_01837	hoi109_01723	hoi117_02028	hoi106_01373
535	hypothetical protein	16	Core	hoi102_00545	hoi103_00542	hoi104_00460	hoi108_00495	hoi110_01795	hoi111_01593	hoi112_00827	hoi113_00969	hoi114_01785	hoi115_00545	hoi116_01901	hoi105_01792	hoi107_01838	hoi109_01722	hoi117_02029	hoi106_01374
536	ABC transporter glutamine-binding protein GlnH	16	Core	hoi102_00546	hoi103_00543	hoi104_00461	hoi108_00496	hoi110_01794	hoi111_01592	hoi112_00828	hoi113_00970	hoi114_01786	hoi115_00546	hoi116_01902	hoi105_01793	hoi107_01839	hoi109_01721	hoi117_02030	hoi106_01375
537	Serine/threonine-protein kinase PknG	16	Core	hoi102_00547	hoi103_00544	hoi104_00462	hoi108_00497	hoi110_01793	hoi111_01591	hoi112_00829	hoi113_00971	hoi114_01787	hoi115_00547	hoi116_01903	hoi105_01794	hoi107_01840	hoi109_01720	hoi117_02031	hoi106_01376
538	Acetate kinase	16	Core	hoi102_00548	hoi103_00545	hoi104_00463	hoi108_00498	hoi110_01792	hoi111_01590	hoi112_00830	hoi113_00972	hoi114_01788	hoi115_00548	hoi116_01904	hoi105_01796	hoi107_01842	hoi109_01718	hoi117_02033	hoi106_01378
539	Phosphate acetyltransferase	16	Core	hoi102_00549	hoi103_00546	hoi104_00464	hoi108_00499	hoi110_01791	hoi111_01589	hoi112_00831	hoi113_00973	hoi114_01789	hoi115_00549	hoi116_01905	hoi105_01797	hoi107_01843	hoi109_01717	hoi117_02034	hoi106_01379
540	NADPH-ferredoxin reductase FprA	16	Core	hoi102_00550	hoi103_00547	hoi104_00465	hoi108_00500	hoi110_01790	hoi111_01588	hoi112_00832	hoi113_00974	hoi114_01790	hoi115_00550	hoi116_01906	hoi105_01798	hoi107_01844	hoi109_01716	hoi117_02035	hoi106_01380
541	Maltodextrin phosphorylase	16	Core	hoi102_00551	hoi103_00548	hoi104_00466	hoi108_00501	hoi110_01789	hoi111_01587	hoi112_00833	hoi113_00975	hoi114_01791	hoi115_00551	hoi116_01907	hoi105_01799	hoi107_01845	hoi109_01715	hoi117_02036	hoi106_01381
542	Periplasmic zinc-binding protein TroA precursor	16	Core	hoi102_00552	hoi103_00549	hoi104_00467	hoi108_00502	hoi110_01788	hoi111_01586	hoi112_00834	hoi113_00976	hoi114_01792	hoi115_00552	hoi116_01908	hoi105_01800	hoi107_01846	hoi109_01714	hoi117_02037	hoi106_01382
543	putative ABC transporter ATP-binding protein	16	Core	hoi102_00553	hoi103_00550	hoi104_00468	hoi108_00503	hoi110_01787	hoi111_01585	hoi112_00835	hoi113_00977	hoi114_01793	hoi115_00553	hoi116_01909	hoi105_01801	hoi107_01847	hoi109_01713	hoi117_02038	hoi106_01383
544	Manganese transport system membrane protein	16	Core	hoi102_00554	hoi103_00551	hoi104_00469	hoi108_00504	hoi110_01786	hoi111_01584	hoi112_00836	hoi113_00978	hoi114_01794	hoi115_00554	hoi116_01910	hoi105_01802	hoi107_01848	hoi109_01712	hoi117_02039	hoi106_01384
545	Manganese transport system membrane protein	16	Core	hoi102_00555	hoi103_00552	hoi104_00470	hoi108_00505	hoi110_01785	hoi111_01583	hoi112_00837	hoi113_00979	hoi114_01795	hoi115_00555	hoi116_01911	hoi105_01803	hoi107_01849	hoi109_01711	hoi117_02040	hoi106_01385
546	Xanthine phosphoribosyltransferase	16	Core	hoi102_00556	hoi103_00553	hoi104_00471	hoi108_00506	hoi110_01784	hoi111_01582	hoi112_00838	hoi113_00980	hoi114_01796	hoi115_00556	hoi116_01912	hoi105_01804	hoi107_01850	hoi109_01710	hoi117_02041	hoi106_01386
547	Phosphoribosylglycinamide formyltransferase 2	16	Core	hoi102_00557	hoi103_00554	hoi104_00472	hoi108_00507	hoi110_01783	hoi111_01581	hoi112_00839	hoi113_00981	hoi114_01797	hoi115_00557	hoi116_01913	hoi105_01805	hoi107_01851	hoi109_01709	hoi117_02042	hoi106_01387
548	Fimbrial subunit type 1 precursor	16	Core	hoi102_00558	hoi103_00555	hoi104_00473	hoi108_00508	hoi110_01782	hoi111_01580	hoi112_00840	hoi113_00982	hoi114_01798	hoi115_00558	hoi116_01914	hoi105_01806	hoi107_01852	hoi109_01708	hoi117_02043	hoi106_01388
549	Sortase family protein	16	Core	hoi102_00559	hoi103_00556	hoi104_00474	hoi108_00509	hoi110_01781	hoi111_01579	hoi112_00841	hoi113_00983	hoi114_01799	hoi115_00559	hoi116_01915	hoi105_01807	hoi107_01853	hoi109_01707	hoi117_02044	hoi106_01389
550	Fimbrial subunit type 1 precursor	16	Core	hoi102_00560	hoi103_00557	hoi104_00475	hoi108_00510	hoi110_01780	hoi111_01578	hoi112_00842	hoi113_00984	hoi114_01800	hoi115_00560	hoi116_01916	hoi105_01808	hoi107_01854	hoi109_01706	hoi117_02045	hoi106_01390
551	hypothetical protein	16	Core	hoi102_00561	hoi103_00558	hoi104_00476	hoi108_00511	hoi110_01779	hoi111_01577	hoi112_00843	hoi113_00985	hoi114_01801	hoi115_00561	hoi116_01917	hoi105_01809	hoi107_01855	hoi109_01705	hoi117_02046	hoi106_01391
552	Adenylosuccinate synthetase	16	Core	hoi102_00562	hoi103_00559	hoi104_00477	hoi108_00512	hoi110_01778	hoi111_01576	hoi112_00844	hoi113_00986	hoi114_01802	hoi115_00562	hoi116_01918	hoi105_01810	hoi107_01856	hoi109_01704	hoi117_02048	hoi106_01392
553	Deoxyribodipyrimidine photo-lyase	16	Core	hoi102_00563	hoi103_00560	hoi104_00478	hoi108_00513	hoi110_01777	hoi111_01575	hoi112_00845	hoi113_00987	hoi114_01803	hoi115_00563	hoi116_01919	hoi105_01811	hoi107_01857	hoi109_01703	hoi117_02049	hoi106_01393
554	hypothetical protein	16	Core	hoi102_00564	hoi103_00561	hoi104_00479	hoi108_00514	hoi110_01776	hoi111_01574	hoi112_00846	hoi113_00988	hoi114_01804	hoi115_00564	hoi116_01920	hoi105_01812	hoi107_01858	hoi109_01702	hoi117_02050	hoi106_01394
555	hypothetical protein	16	Core	hoi102_00565	hoi103_00562	hoi104_00480	hoi108_00515	hoi110_01775	hoi111_01573	hoi112_00847	hoi113_00989	hoi114_01805	hoi115_00565	hoi116_01921	hoi105_01813	hoi107_01859	hoi109_01701	hoi117_02051	hoi106_01395
556	putative 2-keto-3-deoxy-galactonate aldolase Ya	16	Core	hoi102_00566	hoi103_00563	hoi104_00481	hoi108_00516	hoi110_01774	hoi111_01572	hoi112_00848	hoi113_00990	hoi114_01806	hoi115_00566	hoi116_01922	hoi105_01814	hoi107_01860	hoi109_01700	hoi117_02052	hoi106_01396
557	Oligopeptide transport ATP-binding protein OppI	16	Core	hoi102_00567	hoi103_00564	hoi104_00482	hoi108_00517	hoi110_01773	hoi111_01571	hoi112_00849	hoi113_00991	hoi114_01807	hoi115_00567	hoi116_01923	hoi105_01815	hoi107_01861	hoi109_01699	hoi117_02053	hoi106_01397

558	Oligopeptide transport ATP-binding protein OppI	16	Core	hoi102_00568	hoi103_00565	hoi104_00483	hoi108_00518	hoi110_01772	hoi111_01570	hoi112_00850	hoi113_00992	hoi114_01808	hoi115_00568	hoi116_01924	hoi105_01816	hoi107_01862	hoi109_01698	hoi117_02054	hoi106_01398
559	Glutathione transport system permease protein	16	Core	hoi102_00569	hoi103_00566	hoi104_00484	hoi108_00519	hoi110_01771	hoi111_01569	hoi112_00851	hoi113_00993	hoi114_01809	hoi115_00569	hoi116_01925	hoi105_01817	hoi107_01863	hoi109_01697	hoi117_02055	hoi106_01399
560	Glutathione-binding protein GsiB precursor	16	Core	hoi102_00570	hoi103_00567	hoi104_00485	hoi108_00520	hoi110_01770	hoi111_01568	hoi112_00852	hoi113_00994	hoi114_01810	hoi115_00570	hoi116_01926	hoi105_01818	hoi107_01864	hoi109_01696	hoi117_02056	hoi106_01400
561	HTH-type transcriptional regulator LutR	16	Core	hoi102_00571	hoi103_00568	hoi104_00486	hoi108_00521	hoi110_01769	hoi111_01567	hoi112_00853	hoi113_00995	hoi114_01811	hoi115_00571	hoi116_01927	hoi105_01819	hoi107_01865	hoi109_01695	hoi117_02057	hoi106_01401
562	N-acetyl-D-glucosamine kinase	16	Core	hoi102_00572	hoi103_00569	hoi104_00487	hoi108_00522	hoi110_01768	hoi111_01566	hoi112_00854	hoi113_00996	hoi114_01812	hoi115_00572	hoi116_01928	hoi105_01820	hoi107_01866	hoi109_01694	hoi117_02058	hoi106_01402
563	Putative N-acetylmannosamine-6-phosphate 2-e	16	Core	hoi102_00573	hoi103_00570	hoi104_00488	hoi108_00523	hoi110_01767	hoi111_01565	hoi112_00855	hoi113_00997	hoi114_01813	hoi115_00573	hoi116_01929	hoi105_01821	hoi107_01867	hoi109_01693	hoi117_02059	hoi106_01403
564	N-acetylglucosamine-6-phosphate deacetylase	16	Core	hoi102_00574	hoi103_00571	hoi104_00489	hoi108_00524	hoi110_01766	hoi111_01564	hoi112_00856	hoi113_00998	hoi114_01814	hoi115_00574	hoi116_01930	hoi105_01822	hoi107_01868	hoi109_01692	hoi117_02060	hoi106_01404
565	hypothetical protein	16	Core	hoi102_00575	hoi103_00572	hoi104_00490	hoi108_00525	hoi110_01765	hoi111_01563	hoi112_00857	hoi113_00999	hoi114_01815	hoi115_00575	hoi116_01931	hoi105_01823	hoi107_01869	hoi109_01691	hoi117_02061	hoi106_01405
566	Fructose-bisphosphate aldolase	15	Accessory	hoi102_00576	hoi103_00573	hoi104_00491	hoi108_00526	hoi110_01764	hoi111_01562	hoi112_00858	hoi113_01000	hoi114_01816	hoi115_00576		hoi105_01826	hoi107_01872	hoi109_01687	hoi117_02064	hoi106_01406
567	Microcin C7 self-immunity protein MccF	16	Core	hoi102_00577	hoi103_00574	hoi104_00492	hoi108_00527	hoi110_00875	hoi111_00955	hoi112_00859	hoi113_01001	hoi114_01441	hoi115_00577	hoi116_01349	hoi105_01261	hoi107_01289	hoi109_01220	hoi117_01620	hoi106_01675
568	hypothetical protein	16	Core	hoi102_00578	hoi103_00575	hoi104_00493	hoi108_00528	hoi110_00876	hoi111_00956	hoi112_00860	hoi113_01002	hoi114_01442	hoi115_00578	hoi116_01350	hoi105_01263	hoi107_01291	hoi109_01222	hoi117_01622	hoi106_01677
569	Glycosyl hydrolase family 76	16	Core	hoi102_00579	hoi103_00576	hoi104_00494	hoi108_00529	hoi110_00877	hoi111_00957	hoi112_00861	hoi113_01003	hoi114_01443	hoi115_00579	hoi116_01351	hoi105_01272	hoi107_01299	hoi109_01231	hoi117_01631	hoi106_01678
570	tRNA (guanosine(18)-2'-O)-methyltransferase	16	Core	hoi102_00580	hoi103_00577	hoi104_00495	hoi108_00530	hoi110_00878	hoi111_00958	hoi112_00862	hoi113_01004	hoi114_01444	hoi115_00580	hoi116_01352	hoi105_01273	hoi107_01300	hoi109_01232	hoi117_01632	hoi106_01679
571	Orotate phosphoribosyltransferase	16	Core	hoi102_00581	hoi103_00578	hoi104_00496	hoi108_00531	hoi110_00879	hoi111_00959	hoi112_00863	hoi113_01005	hoi114_01445	hoi115_00581	hoi116_01353	hoi105_01274	hoi107_01301	hoi109_01233	hoi117_01633	hoi106_01680
572	hypothetical protein	16	Core	hoi102_00582	hoi103_00579	hoi104_00497	hoi108_00532	hoi110_00880	hoi111_00960	hoi112_00864	hoi113_01006	hoi114_01446	hoi115_00582	hoi116_01354	hoi105_01275	hoi107_01302	hoi109_01234	hoi117_01634	hoi106_01681
573	3-mercaptopyruvate sulfurtransferase	16	Core	hoi102_00583	hoi103_00580	hoi104_00498	hoi108_00533	hoi110_00881	hoi111_00961	hoi112_00865	hoi113_01007	hoi114_01447	hoi115_00583	hoi116_01355	hoi105_01276	hoi107_01303	hoi109_01235	hoi117_01635	hoi106_01682
574	hypothetical protein	16	Core	hoi102_00584	hoi103_00581	hoi104_00499	hoi108_00534	hoi110_00882	hoi111_00962	hoi112_00866	hoi113_01008	hoi114_01448	hoi115_00584	hoi116_01356	hoi105_01277	hoi107_01304	hoi109_01236	hoi117_01636	hoi106_01683
575	Chaperone protein ClpB	16	Core	hoi102_00585	hoi103_00582	hoi104_00500	hoi108_00535	hoi110_00883	hoi111_00963	hoi112_00867	hoi113_01009	hoi114_01449	hoi115_00585	hoi116_01357	hoi105_01278	hoi107_01305	hoi109_01237	hoi117_01637	hoi106_01684
576	Phenol hydroxylase P5 protein	16	Core	hoi102_00586	hoi103_00583	hoi104_00501	hoi108_00536	hoi110_00884	hoi111_00964	hoi112_00868	hoi113_01010	hoi114_01450	hoi115_00586	hoi116_01358	hoi105_01279	hoi107_01306	hoi109_01238	hoi117_01638	hoi106_01685
577	(R)-stereoselective amidase	16	Core	hoi102_00587	hoi103_00584	hoi104_00502	hoi108_00537	hoi110_00885	hoi111_00965	hoi112_00869	hoi113_01011	hoi114_01451	hoi115_00587	hoi116_01359	hoi105_01280	hoi107_01307	hoi109_01239	hoi117_01639	hoi106_01686
578	hypothetical protein	16	Core	hoi102_00588	hoi103_00585	hoi104_00503	hoi108_00538	hoi110_00886	hoi111_00966	hoi112_00870	hoi113_01012	hoi114_01452	hoi115_00588	hoi116_01360	hoi105_01281	hoi107_01308	hoi109_01240	hoi117_01640	hoi106_01687
579	hypothetical protein	16	Core	hoi102_00589	hoi103_00586	hoi104_00504	hoi108_00539	hoi110_00887	hoi111_00967	hoi112_00871	hoi113_01013	hoi114_01453	hoi115_00589	hoi116_01361	hoi105_01282	hoi107_01309	hoi109_01241	hoi117_01641	hoi106_01688
580	EPTC-inducible aldehyde dehydrogenase	16	Core	hoi102_00590	hoi103_00587	hoi104_00505	hoi108_00540	hoi110_00888	hoi111_00968	hoi112_00872	hoi113_01014	hoi114_01454	hoi115_00590	hoi116_01362	hoi105_01283	hoi107_01310	hoi109_01242	hoi117_01642	hoi106_01689
581	putative metallo-hydrolase	16	Core	hoi102_00591	hoi103_00588	hoi104_00506	hoi108_00541	hoi110_00889	hoi111_00969	hoi112_00873	hoi113_01015	hoi114_01455	hoi115_00591	hoi116_01363	hoi105_01284	hoi107_01311	hoi109_01243	hoi117_01643	hoi106_01690
582	S-(hydroxymethyl)mycothiol dehydrogenase	16	Core	hoi102_00592	hoi103_00589	hoi104_00507	hoi108_00542	hoi110_00890	hoi111_00970	hoi112_00874	hoi113_01016	hoi114_01456	hoi115_00592	hoi116_01364	hoi105_01285	hoi107_01312	hoi109_01244	hoi117_01644	hoi106_01691
583	Putative heat shock protein HspR	16	Core	hoi102_00593	hoi103_00590	hoi104_00508	hoi108_00543	hoi110_00891	hoi111_00971	hoi112_00875	hoi113_01017	hoi114_01457	hoi115_00593	hoi116_01365	hoi105_01286	hoi107_01313	hoi109_01245	hoi117_01645	hoi106_01692
584	Chaperone protein DnaJ	16	Core	hoi102_00594	hoi103_00591	hoi104_00509	hoi108_00544	hoi110_00892	hoi111_00972	hoi112_00876	hoi113_01018	hoi114_01458	hoi115_00594	hoi116_01366	hoi105_01287	hoi107_01314	hoi109_01246	hoi117_01646	hoi106_01693
585	heat shock protein GrpE	16	Core	hoi102_00595	hoi103_00592	hoi104_00510	hoi108_00545	hoi110_00893	hoi111_00973	hoi112_00877	hoi113_01019	hoi114_01459	hoi115_00595	hoi116_01367	hoi105_01288	hoi107_01315	hoi109_01247	hoi117_01647	hoi106_01694
586	Chaperone protein DnaK	16	Core	hoi102_00596	hoi103_00593	hoi104_00511	hoi108_00546	hoi110_00894	hoi111_00974	hoi112_00878	hoi113_01020	hoi114_01460	hoi115_00596	hoi116_01368	hoi105_01289	hoi107_01316	hoi109_01248	hoi117_01648	hoi106_01695
587	Porphobilinogen deaminase	16	Core	hoi102_00597	hoi103_00594	hoi104_00512	hoi108_00547	hoi110_00895	hoi111_00975	hoi112_00879	hoi113_01021	hoi114_01461	hoi115_00597	hoi116_01369	hoi105_01290	hoi107_01317	hoi109_01249	hoi117_01649	hoi106_01696
588	Glutamyl-tRNA reductase	16	Core	hoi102_00598	hoi103_00595	hoi104_00513	hoi108_00548	hoi110_00896	hoi111_00976	hoi112_00880	hoi113_01022	hoi114_01462	hoi115_00598	hoi116_01370	hoi105_01291	hoi107_01318	hoi109_01250	hoi117_01650	hoi106_01697
589	hypothetical protein	16	Core	hoi102_00599	hoi103_00596	hoi104_00514	hoi108_00549	hoi110_00897	hoi111_00977	hoi112_00881	hoi113_01023	hoi114_01463	hoi115_00599	hoi116_01371	hoi105_01292	hoi107_01319	hoi109_01251	hoi117_01651	hoi106_01698
590	Phosphoserine phosphatase	16	Core	hoi102_00600	hoi103_00597	hoi104_00515	hoi108_00550	hoi110_00898	hoi111_00978	hoi112_00882	hoi113_01024	hoi114_01464	hoi115_00600	hoi116_01372	hoi105_01293	hoi107_01320	hoi109_01252	hoi117_01652	hoi106_01699
591	Helix-turn-helix domain protein	16	Core	hoi102_00602	hoi103_00599	hoi104_00517	hoi108_00552	hoi110_00900	hoi111_00980	hoi112_00884	hoi113_01026	hoi114_01466	hoi115_00602	hoi116_01374	hoi105_01295	hoi107_01322	hoi109_01254	hoi117_01654	hoi106_01701
592	Pyrroline-5-carboxylate reductase	16	Core	hoi102_00603	hoi103_00600	hoi104_00518	hoi108_00553	hoi110_00901	hoi111_00981	hoi112_00885	hoi113_01027	hoi114_01467	hoi115_00603	hoi116_01375	hoi105_01296	hoi107_01323	hoi109_01255	hoi117_01655	hoi106_01702
593	hypothetical protein	16	Core	hoi102_00604	hoi103_00601	hoi104_00519	hoi108_00554	hoi110_00902	hoi111_00982	hoi112_00886	hoi113_01028	hoi114_01468	hoi115_00604	hoi116_01376	hoi105_01297	hoi107_01324	hoi109_01256	hoi117_01656	hoi106_01703
594	Exopolyphosphatase	16	Core	hoi102_00605	hoi103_00602	hoi104_00520	hoi108_00555	hoi110_00903	hoi111_00983	hoi112_00887	hoi113_01029	hoi114_01469	hoi115_00605	hoi116_01377	hoi105_01298	hoi107_01325	hoi109_01257	hoi117_01657	hoi106_01704
595	hypothetical protein	16	Core	hoi102_00606	hoi103_00603	hoi104_00521	hoi108_00556	hoi110_00904	hoi111_00984	hoi112_00888	hoi113_01030	hoi114_01470	hoi115_00606	hoi116_01378	hoi105_01299	hoi107_01326	hoi109_01258	hoi117_01658	hoi106_01705
596	Sensory transduction protein regX3	16	Core	hoi102_00607	hoi103_00604	hoi104_00522	hoi108_00557	hoi110_00905	hoi111_00985	hoi112_00889	hoi113_01031	hoi114_01471	hoi115_00607	hoi116_01379	hoi105_01300	hoi107_01327	hoi109_01259	hoi117_01659	hoi106_01706
597	Signal-transduction histidine kinase senX3	16	Core	hoi102_00608	hoi103_00605	hoi104_00523	hoi108_00558	hoi110_00906	hoi111_00986	hoi112_00890	hoi113_01032	hoi114_01472	hoi115_00608	hoi116_01380	hoi105_01301	hoi107_01328	hoi109_01260	hoi117_01660	hoi106_01707
598	2,3-bisphosphoglycerate-dependent phosphogly	16	Core	hoi102_00609	hoi103_00606	hoi104_00524	hoi108_00559	hoi110_00907	hoi111_00987	hoi112_00891	hoi113_01033	hoi114_01473	hoi115_00609	hoi116_01381	hoi105_01302	hoi107_01329	hoi109_01261	hoi117_01661	hoi106_01708
599	D-inositol 3-phosphate glycosyltransferase	16	Core	hoi102_00610	hoi103_00607	hoi104_00525	hoi108_00560	hoi110_00908	hoi111_00988	hoi112_00892	hoi113_01034	hoi114_01474	hoi115_00610	hoi116_01382	hoi105_01303	hoi107_01330	hoi109_01262	hoi117_01662	hoi106_01709
600	Long-chain-fatty-acid--CoA ligase	16	Core	hoi102_00611	hoi103_00608	hoi104_00526	hoi108_00561	hoi110_00909	hoi111_00989	hoi112_00893	hoi113_01035	hoi114_01475	hoi115_00611	hoi116_01383	hoi105_01304	hoi107_01331	hoi109_01263	hoi117_01663	hoi106_01710
601	hypothetical protein	16	Core	hoi102_00612	hoi103_00609	hoi104_00527	hoi108_00562	hoi110_00910	hoi111_00990	hoi112_00894	hoi113_01036	hoi114_01476	hoi115_00612	hoi116_01384	hoi105_01305	hoi107_01332	hoi109_01264	hoi117_01664	hoi106_01711
602	UDP-N-acetylenolpyruvoylglucosamine reductas	16	Core	hoi102_00613	hoi103_00610	hoi104_00528	hoi108_00563	hoi110_00911	hoi111_00991	hoi112_00895	hoi113_01037	hoi114_01477	hoi115_00613	hoi116_01385	hoi105_01306	hoi107_01333	hoi109_01265	hoi117_01665	hoi106_01712
603	hypothetical protein	16	Core	hoi102_00614	hoi103_00611	hoi104_00529	hoi108_00564	hoi110_00912	hoi111_00992	hoi112_00896	hoi113_01038	hoi114_01478	hoi115_00614	hoi116_01386	hoi105_01307	hoi107_01334	hoi109_01266	hoi117_01666	hoi106_01713
604	hypothetical protein	16	Core	hoi102_00615	hoi103_00612	hoi104_00530	hoi108_00565	hoi110_00913	hoi111_00993	hoi112_00897	hoi113_01039	hoi114_01479	hoi115_00615	hoi116_01387	hoi105_01308	hoi107_01335	hoi109_01267	hoi117_01667	hoi106_01714
605	hypothetical protein	16	Core	hoi102_00616	hoi103_00613	hoi104_00531	hoi108_00566	hoi110_00914	hoi111_00994	hoi112_00898	hoi113_01040	hoi114_01480	hoi115_00616	hoi116_01388	hoi105_01309	hoi107_01336	hoi109_01268	hoi117_01668	hoi106_01715
606	deoxyribose-phosphate aldolase	16	Core	hoi102_00617	hoi103_00614	hoi104_00532	hoi108_00567	hoi110_00915	hoi111_00995	hoi112_00899	hoi113_01041	hoi114_01481	hoi115_00617	hoi116_01389	hoi105_01310	hoi107_01337	hoi109_01269	hoi117_01669	hoi106_01716
607	Daunorubicin/doxorubicin resistance ATP-bindin	11	Accessory	hoi102_00618	hoi103_00615	hoi104_00533	hoi108_00568	hoi110_00916	hoi111_00996	hoi112_00900	hoi113_01042	hoi114_01482	hoi115_00618	hoi116_01390					
608	ABC-2 type transporter	11	Accessory	hoi102_00619	hoi103_00616	hoi104_00534	hoi108_00569	hoi110_00917	hoi111_00997	hoi112_00901	hoi113_01043	hoi114_01483	hoi115_00619	hoi116_01391					
609	Sensor histidine kinase DesK	11	Accessory	hoi102_00620	hoi103_00617	hoi104_00535	hoi108_00570	hoi110_00918	hoi111_00998	hoi112_00902	hoi113_01044	hoi114_01484	hoi115_00620	hoi116_01392					
610	Transcriptional regulatory protein UhpA	11	Accessory	hoi102_00621	hoi103_00618	hoi104_00536	hoi108_00571	hoi110_00919	hoi111_00999	hoi112_00903	hoi113_01045	hoi114_01485	hoi115_00621	hoi116_01393					
611	hypothetical protein	16	Core	hoi102_00622	hoi103_00619	hoi104_00537	hoi108_00572	hoi110_00920	hoi111_01000	hoi112_00904	hoi113_01046	hoi114_01486	hoi115_00622	hoi116_01394	hoi105_01311	hoi107_01338	hoi109_01270	hoi117_01670	hoi106_01717
612	hypothetical protein	16	Core	hoi102_00623	hoi103_00620	hoi104_00538	hoi108_00573	hoi110_00921	hoi111_01001	hoi112_00905	hoi113_01047	hoi114_01487	hoi115_00623	hoi116_01395	hoi105_01312	hoi107_01339	hoi109_01271	hoi117_01671	hoi106_01718
613	hypothetical protein	16	Core	hoi102_00625	hoi103_00622	hoi104_00540	hoi108_00575	hoi110_00923	hoi111_01003	hoi112_00907	hoi113_01049	hoi114_01489	hoi115_00625	hoi116_01397	hoi105_01314	hoi107_01341	hoi109_01273	hoi117_01673	hoi106_01720
614	Fumarate reductase iron-sulfur subunit	16	Core	hoi102_00626	hoi103_00623	hoi104_00541	hoi108_00576	hoi110_00924	hoi111_01004	hoi112_00908	hoi113_01050	hoi114_01490	hoi115_00626	hoi116_01398	hoi105_01315	hoi107_01342	hoi109_01274	hoi117_01674	hoi106_01721
615	Fumarate reductase flavoprotein subunit	16	Core	hoi102_00627	hoi103_00624	hoi104_00542	hoi108_00577	hoi110_00925	hoi111_01005	hoi112_00909	hoi113_01051	hoi114_01491	hoi115_00627	hoi116_01399	hoi105_01316	hoi107_01343	hoi109_01275	hoi117_01675	hoi106_01722
616	Succinate dehydrogenase/Fumarate reductase tr	16	Core	hoi102_00628	hoi103_00625	hoi104_00543	hoi108_00578	hoi110_00926	hoi111_01006	hoi112_00910	hoi113_01052	hoi114_01492	hoi115_00628	hoi116_01400	hoi105_01317	hoi107_01344	hoi109_01276	hoi117_01676	hoi106_01723
617	DNA-binding transcriptional repressor PuuR	16	Core	hoi102_00629	hoi103_00626	hoi104_00544	hoi108_00579	hoi110_00927	hoi111_01007	hoi112_00911	hoi113_01053	hoi114_01493	hoi115_00629	hoi116_01401	hoi105_01318	hoi107_01345	hoi109_01277	hoi117_01677	hoi106_01724
618	2-succinylbenzoate--CoA ligase	16	Core	hoi102_00630	hoi103_00627	hoi104_00545	hoi108_00580	hoi110_00928	hoi111_01008	hoi112_00912	hoi113_01054	hoi114_01494	hoi115_00630	hoi116_01402	hoi105_01319	hoi107_01346	hoi109_01278	hoi117_01678	hoi106_01725
619	hypothetical protein	16	Core	hoi102_00631	hoi103_00628	hoi104_00546	hoi108_00581	hoi110_00929	hoi111_01009	hoi112_00913	hoi113_01055	hoi114_01495	hoi115_00631	hoi116_01403	hoi105_01320	hoi107_01347	hoi109_01279	hoi117_01679	hoi106_01726
620	1,4-dihydroxy-2-naphthoate octaprenyltransfera	16	Core	hoi102_00632	hoi103_00629	hoi104_00547	hoi108_00582	hoi110_00930	hoi111_01010	hoi112_00914	hoi113_01056	hoi114_01496	hoi115_00632	hoi116_01404	hoi105_01321	hoi107_01348	hoi109_01280	hoi117_01680	hoi106_01727
621	hypothetical protein	16	Core	hoi102_00633	hoi103_00630	hoi104_00548	hoi108_00583	hoi110_00931	hoi111_01011	hoi112_00915	hoi113_01057	hoi114_01497	hoi115_00633	hoi116_01405	hoi105_01322	hoi107_01349	hoi109_01281	hoi117_01681	hoi106_01728
622	hypothetical protein	16	Core	hoi102_00634	hoi103_00631	hoi104_00549	hoi108_00584	hoi110_00932	hoi111_01012	hoi112_00916	hoi113_01058	hoi114_01498	hoi115_00634	hoi116_01406	hoi105_01323	hoi107_01350	hoi109_01282	hoi117_01682	hoi106_01729
623	Glucose-1-phosphate thymidylyltransferase	16	Core	hoi102_00635	hoi103_00632	hoi104_00550	hoi108_00585	hoi110_00933	hoi111_01013	hoi112_00917	hoi113_01059	hoi114_01499	hoi115_00635	hoi116_01407	hoi105_01324	hoi107_01351	hoi109_01283	hoi117_01683	hoi106_01730
624	dTDP-4-dehydrorhamnose 3,5-epimerase	16	Core	hoi102_00636	hoi103_00633	hoi104_00551	hoi108_00586	hoi110_00934	hoi111_01014	hoi112_00918	hoi113_01060	hoi114_01500	hoi115_00636	hoi116_01408	hoi105_01325	hoi107_01352	hoi109_01284	hoi117_01684	hoi106_01731
625	dTDP-glucose 4,6-dehydratase	16	Core	hoi102_00637	hoi103_00634	hoi104_00552	hoi108_00587	hoi110_00935	hoi111_01015	hoi112_00919	hoi113_01061	hoi114_01501	hoi115_00637	hoi116_01409	hoi105_01326	hoi107_01353	hoi109_01285	hoi117_01685	hoi106_01732
626	Cytochrome c biogenesis protein CcsA	16	Core	hoi102_00638	hoi103_00635	hoi104_00553	hoi108_00588	hoi110_00936	hoi111_01016	hoi112_00920	hoi113_01062	hoi114_01502	hoi115_00638	hoi116_01410	hoi105_01327	hoi107_01354	hoi109_01286	hoi117_01686	hoi106_01733
627	Cytochrome c biogenesis protein Ccs1	16	Core	hoi102_00639	hoi103_00636	hoi104_00554	hoi108_00589	hoi110_00937	hoi111_01017	hoi112_00921	hoi113_01063	hoi114_01503	hoi115_00639	hoi116_01411	hoi105_01328	hoi107_01355	hoi109_01287	hoi117_01687	hoi106_01734
628	Cytochrome C biogenesis protein transmembran	16	Core	hoi102_00640	hoi103_00637	hoi104_00555	hoi108_00590	hoi110_00938	hoi111_01018	hoi112_00922	hoi113_01064	hoi114_01504	hoi115_00640	hoi116_01412	hoi105_01329	hoi107_01356	hoi109_01288	hoi117_01688	hoi106_01735
629	Thiol-disulfide oxidoreductase ResA	16	Core	hoi102_00641	hoi103_00638	hoi104_00556	hoi108_00591	hoi110_00939	hoi111_01019	hoi112_00923	hoi113_01065	hoi114_01505	hoi115_00641	hoi116_01413	hoi105_01330	hoi107_01357	hoi109_01289	hoi117_01689	hoi106_01736
630	bifunctional RNase H/acid phosphatase	16	Core	hoi102_00642	hoi103_00639	hoi104_00557	hoi108_00592	hoi110_00940	hoi111_01020	hoi112_00924	hoi113_01066	hoi114_01506	hoi115_00642	hoi116_01414	hoi105_01331	hoi107_01358	hoi109_01290	hoi117_01690	hoi106_01737
631	Glutamate-1-semialdehyde 2,1-aminomutase	16	Core	hoi102_00643	hoi103_00640	hoi104_00558	hoi108_00593	hoi110_00941	hoi111_01021	hoi112_00925	hoi113_01067	hoi114_01507	hoi115_00643	hoi116_01415	hoi105_01332	hoi107_01359	hoi109_01291	hoi117_01691	hoi106_01738
632	Protoporphyrinogen oxidase	16	Core	hoi102_00644	hoi103_00641	hoi104_00559	hoi108_00594	hoi110_00942	hoi111_01022	hoi112_00926	hoi113_01068	hoi114_01508	hoi115_00644	hoi116_01416	hoi105_01333	hoi107_01360	hoi109_01292	hoi117_01692	hoi106_01739
633	Uroporphyrinogen decarboxylase	16	Core	hoi102_00645	hoi103_00642	hoi104_00560	hoi108_00595	hoi110_00943	hoi111_01023	hoi112_00927	hoi113_01069	hoi114_01509	hoi115_00645	hoi116_01417	hoi105_01334	hoi107_01361	hoi109_01293	hoi117_01693	hoi106_01740
634	Sulfite exporter TauE/SafE	16	Core	hoi102_00646	hoi103_00643	hoi104_00561	hoi108_00596	hoi110_00944	hoi111_01024	hoi112_00928	hoi113_01070	hoi114_01510	hoi115_00646	hoi116_01418	hoi105_01335	hoi107_01362	hoi109_01294	hoi117_01694	hoi106_01741
635	putative copper-exporting P-type ATPase V	16	Core	hoi102_00647	hoi103_00644	hoi104_00562	hoi108_00597	hoi110_00945	hoi111_01025	hoi112_00929	hoi113_01071	hoi114_01511	hoi115_00647	hoi116_01419	hoi105_01336	hoi107_01363	hoi109_01295	hoi117_01695	hoi106_01742
636	hypothetical protein	16	Core	hoi102_00648	hoi103_00645	hoi104_00563	hoi108_00598	hoi110_00946	hoi111_01026	hoi112_00930	hoi113_01072	hoi114_01512	hoi115_00648	hoi116_01420	hoi105_01337	hoi107_01364	hoi109_01296	hoi117_01696	hoi106_01743
637	hypothetical protein	16	Core	hoi102_00649	hoi103_00646	hoi104_00564	hoi108_00599	hoi110_00947	hoi111_01027	hoi112_00931	hoi113_01073	hoi114_01513	hoi115_00649	hoi116_01421	hoi105_01338	hoi107_01365	hoi109_01297	hoi117_01697	hoi106_01744

638	Delta-aminolevulinic acid dehydratase	16	Core	hoi102_00650	hoi103_00647	hoi104_00565	hoi108_00600	hoi110_00948	hoi111_01028	hoi112_00932	hoi113_01074	hoi114_01514	hoi115_00650	hoi116_01422	hoi105_01339	hoi107_01366	hoi109_01298	hoi117_01698	hoi106_01745
639	Siroheme synthase	16	Core	hoi102_00651	hoi103_00648	hoi104_00566	hoi108_00601	hoi110_00949	hoi111_01029	hoi112_00933	hoi113_01075	hoi114_01515	hoi115_00651	hoi116_01423	hoi105_01340	hoi107_01367	hoi109_01299	hoi117_01699	hoi106_01746
640	Polyprenol-phosphate-mannose-dependent alph	16	Core	hoi102_00652	hoi103_00649	hoi104_00567	hoi108_00602	hoi110_00950	hoi111_01030	hoi112_00934	hoi113_01076	hoi114_01516	hoi115_00652	hoi116_01424	hoi105_01341	hoi107_01368	hoi109_01300	hoi117_01700	hoi106_01747
641	Na+/H+ antiporter family protein	16	Core	hoi102_00653	hoi103_00650	hoi104_00568	hoi108_00603	hoi110_00951	hoi111_01031	hoi112_00935	hoi113_01077	hoi114_01517	hoi115_00653	hoi116_01425	hoi105_01342	hoi107_01369	hoi109_01301	hoi117_01701	hoi106_01748
642	pheromone autoinducer 2 transporter	16	Core	hoi102_00654	hoi103_00651	hoi104_00569	hoi108_00604	hoi110_00952	hoi111_01032	hoi112_00936	hoi113_01078	hoi114_01518	hoi115_00654	hoi116_01426	hoi105_01343	hoi107_01370	hoi109_01302	hoi117_01702	hoi106_01749
643	5'-methylthioadenosine/S-adenosylhomocystein	16	Core	hoi102_00655	hoi103_00652	hoi104_00570	hoi108_00605	hoi110_00953	hoi111_01033	hoi112_00937	hoi113_01079	hoi114_01519	hoi115_00655	hoi116_01427	hoi105_01344	hoi107_01371	hoi109_01303	hoi117_01703	hoi106_01750
644	Cyclomaltodextrinase	16	Core	hoi102_00656	hoi103_00653	hoi104_00571	hoi108_00606	hoi110_00954	hoi111_01034	hoi112_00938	hoi113_01080	hoi114_01520	hoi115_00656	hoi116_01428	hoi105_01345	hoi107_01372	hoi109_01304	hoi117_01704	hoi106_01751
645	Putative hydrolase YdeN	16	Core	hoi102_00658	hoi103_00655	hoi104_00573	hoi108_00608	hoi110_00956	hoi111_01036	hoi112_00940	hoi113_01082	hoi114_01522	hoi115_00658	hoi116_01430	hoi105_01347	hoi107_01374	hoi109_01306	hoi117_01706	hoi106_01407
646	Glutamate-pyruvate aminotransferase AlaA	16	Core	hoi102_00659	hoi103_00656	hoi104_00574	hoi108_00609	hoi110_00957	hoi111_01037	hoi112_00941	hoi113_01083	hoi114_01523	hoi115_00659	hoi116_01431	hoi105_01348	hoi107_01375	hoi109_01307	hoi117_01707	hoi106_01408
647	YibE/F-like protein	16	Core	hoi102_00660	hoi103_00657	hoi104_00575	hoi108_00610	hoi110_00958	hoi111_01038	hoi112_00942	hoi113_01084	hoi114_01524	hoi115_00660	hoi116_01432	hoi105_01349	hoi107_01376	hoi109_01308	hoi117_01708	hoi106_01409
648	UDP-glucose 6-dehydrogenase TuaD	16	Core	hoi102_00661	hoi103_00658	hoi104_00576	hoi108_00611	hoi110_00959	hoi111_01039	hoi112_00943	hoi113_01085	hoi114_01525	hoi115_00661	hoi116_01433	hoi105_01350	hoi107_01377	hoi109_01309	hoi117_01709	hoi106_01410
649	Putative osmoprotectant uptake system substrat	16	Core	hoi102_00662	hoi103_00659	hoi104_00577	hoi108_00612	hoi110_00960	hoi111_01040	hoi112_00944	hoi113_01086	hoi114_01526	hoi115_00662	hoi116_01434	hoi105_01351	hoi107_01378	hoi109_01310	hoi117_01710	hoi106_01411
650	Glycine betaine/carnitine/choline transport syste	16	Core	hoi102_00663	hoi103_00660	hoi104_00578	hoi108_00613	hoi110_00961	hoi111_01041	hoi112_00945	hoi113_01087	hoi114_01527	hoi115_00663	hoi116_01435	hoi105_01352	hoi107_01379	hoi109_01311	hoi117_01711	hoi106_01412
651	Choline transport system permease protein Opul	16	Core	hoi102_00664	hoi103_00661	hoi104_00579	hoi108_00614	hoi110_00962	hoi111_01042	hoi112_00946	hoi113_01088	hoi114_01528	hoi115_00664	hoi116_01436	hoi105_01353	hoi107_01380	hoi109_01312	hoi117_01712	hoi106_01413
652	Choline transport ATP-binding protein OpuBA	16	Core	hoi102_00665	hoi103_00662	hoi104_00580	hoi108_00615	hoi110_00963	hoi111_01043	hoi112_00947	hoi113_01089	hoi114_01529	hoi115_00665	hoi116_01437	hoi105_01354	hoi107_01381	hoi109_01313	hoi117_01713	hoi106_01414
653	Deoxycytidine triphosphate deaminase	16	Core	hoi102_00666	hoi103_00663	hoi104_00581	hoi108_00616	hoi110_00964	hoi111_01044	hoi112_00948	hoi113_01090	hoi114_01530	hoi115_00666	hoi116_01438	hoi105_01355	hoi107_01382	hoi109_01314	hoi117_01714	hoi106_01415
654	hypothetical protein	9	Accessory	hoi102_00668	hoi103_00665	hoi104_00583	hoi108_00618	hoi110_00966	hoi111_01046	hoi112_00950		hoi114_01532	hoi115_00668						
655	hypothetical protein	16	Core	hoi102_00669	hoi103_00666	hoi104_00584	hoi108_00619	hoi110_00967	hoi111_01047	hoi112_00951	hoi113_01092	hoi114_01533	hoi115_00669	hoi116_01440	hoi105_01357	hoi107_01384	hoi109_01316	hoi117_01716	hoi106_01417
656	Iron uptake protein A1 precursor	16	Core	hoi102_00670	hoi103_00667	hoi104_00585	hoi108_00620	hoi110_00968	hoi111_01048	hoi112_00952	hoi113_01093	hoi114_01534	hoi115_00670	hoi116_01441	hoi105_01358	hoi107_01385	hoi109_01317	hoi117_01717	hoi106_01418
657	Spermidine/putrescine transport system permea	16	Core	hoi102_00671	hoi103_00668	hoi104_00586	hoi108_00621	hoi110_00969	hoi111_01049	hoi112_00953	hoi113_01094	hoi114_01535	hoi115_00671	hoi116_01442	hoi105_01359	hoi107_01386	hoi109_01318	hoi117_01718	hoi106_01419
658	Sulfate/thiosulfate import ATP-binding protein C	16	Core	hoi102_00672	hoi103_00669	hoi104_00587	hoi108_00622	hoi110_00970	hoi111_01050	hoi112_00954	hoi113_01095	hoi114_01536	hoi115_00672	hoi116_01443	hoi105_01360	hoi107_01387	hoi109_01319	hoi117_01719	hoi106_01420
659	Surfactin synthase subunit 2	16	Core	hoi102_00673	hoi103_00670	hoi104_00588	hoi108_00623	hoi110_00971	hoi111_01051	hoi112_00955	hoi113_01096	hoi114_01537	hoi115_00673	hoi116_01444	hoi105_01361	hoi107_01388	hoi109_01320	hoi117_01720	hoi106_01421
660	Sodium Bile acid symporter family protein	16	Core	hoi102_00674	hoi103_00671	hoi104_00589	hoi108_00624	hoi110_00972	hoi111_01052	hoi112_00956	hoi113_01097	hoi114_01538	hoi115_00674	hoi116_01445	hoi105_01362	hoi107_01389	hoi109_01321	hoi117_01721	hoi106_01422
661	Iron(3+)-hydroxamate import ATP-binding protei	15	Accessory	hoi102_00675	hoi103_00672	hoi104_00590	hoi108_00625	hoi110_00973	hoi111_01053	hoi112_00957	hoi113_01098	hoi114_01539	hoi115_00675	hoi116_01446	hoi105_01363	hoi107_01390	hoi109_01322	hoi117_01722	
662	hypothetical protein	16	Core	hoi102_00676	hoi103_00673	hoi104_00591	hoi108_00626	hoi110_00974	hoi111_01054	hoi112_00958	hoi113_01099	hoi114_01540	hoi115_00676	hoi116_01447	hoi105_01364	hoi107_01391	hoi109_01323	hoi117_01723	hoi106_01424
663	Alcohol dehydrogenase	16	Core	hoi102_00677	hoi103_00674	hoi104_00592	hoi108_00627	hoi110_00975	hoi111_01055	hoi112_00959	hoi113_01100	hoi114_01541	hoi115_00677	hoi116_01448	hoi105_01365	hoi107_01392	hoi109_01324	hoi117_01724	hoi106_01425
664	hypothetical protein	16	Core	hoi102_00678	hoi103_00675	hoi104_00593	hoi108_00628	hoi110_00976	hoi111_01056	hoi112_00960	hoi113_01101	hoi114_01542	hoi115_00678	hoi116_01449	hoi105_01366	hoi107_01393	hoi109_01325	hoi117_01725	hoi106_01426
665	ABC-2 family transporter protein	16	Core	hoi102_00679	hoi103_00676	hoi104_00594	hoi108_00629	hoi110_00977	hoi111_01057	hoi112_00961	hoi113_01102	hoi114_01543	hoi115_00679	hoi116_01450	hoi105_01367	hoi107_01394	hoi109_01326	hoi117_01726	hoi106_01427
666	Prolyl endopeptidase precursor	16	Core	hoi102_00680	hoi103_00677	hoi104_00595	hoi108_00630	hoi110_00978	hoi111_01058	hoi112_00962	hoi113_01103	hoi114_01544	hoi115_00680	hoi116_01451	hoi105_01368	hoi107_01395	hoi109_01327	hoi117_01727	hoi106_01428
667	Aminopeptidase N	16	Core	hoi102_00681	hoi103_00678	hoi104_00596	hoi108_00631	hoi110_00979	hoi111_01059	hoi112_00963	hoi113_01104	hoi114_01545	hoi115_00681	hoi116_01452	hoi105_01369	hoi107_01396	hoi109_01328	hoi117_01728	hoi106_01429
668	hypothetical protein	16	Core	hoi102_00682	hoi103_00679	hoi104_00597	hoi108_00632	hoi110_00980	hoi111_01060	hoi112_00964	hoi113_01105	hoi114_01546	hoi115_00682	hoi116_01453	hoi105_01370	hoi107_01397	hoi109_01329	hoi117_01729	hoi106_01430
669	DoxX	16	Core	hoi102_00683	hoi103_00680	hoi104_00598	hoi108_00633	hoi110_00981	hoi111_01061	hoi112_00965	hoi113_01106	hoi114_01547	hoi115_00683	hoi116_01454	hoi105_01371	hoi107_01398	hoi109_01330	hoi117_01730	hoi106_01431
670	Pectin degradation repressor protein KdgR	16	Core	hoi102_00684	hoi103_00681	hoi104_00599	hoi108_00634	hoi110_00982	hoi111_01062	hoi112_00966	hoi113_01107	hoi114_01548	hoi115_00684	hoi116_01455	hoi105_01372	hoi107_01399	hoi109_01331	hoi117_01731	hoi106_01432
671	putative formaldehyde dehydrogenase AdhA	16	Core	hoi102_00685	hoi103_00682	hoi104_00600	hoi108_00635	hoi110_00983	hoi111_01063	hoi112_00967	hoi113_01108	hoi114_01549	hoi115_00685	hoi116_01456	hoi105_01373	hoi107_01400	hoi109_01332	hoi117_01732	hoi106_01433
672	hypothetical protein	1	Accessory	hoi102_00686															
673	hypothetical protein	16	Core	hoi102_00687	hoi103_00683	hoi104_00601	hoi108_00636	hoi110_00984	hoi111_01064	hoi112_00968	hoi113_01109	hoi114_01550	hoi115_00686	hoi116_01457	hoi105_01374	hoi107_01401	hoi109_01333	hoi117_01733	hoi106_01434
674	UDP-Gal:alpha-D-GlcNAc-diphosphoundecapren	16	Core	hoi102_00688	hoi103_00684	hoi104_00602	hoi108_00637	hoi110_00985	hoi111_01065	hoi112_00969	hoi113_01110	hoi114_01551	hoi115_00687	hoi116_01458	hoi105_01375	hoi107_01402	hoi109_01334	hoi117_01734	hoi106_01435
675	N-glycosyltransferase	16	Core	hoi102_00689	hoi103_00685	hoi104_00603	hoi108_00638	hoi110_00986	hoi111_01066	hoi112_00970	hoi113_01111	hoi114_01552	hoi115_00688	hoi116_01459	hoi105_01376	hoi107_01403	hoi109_01335	hoi117_01735	hoi106_01436
676	hypothetical protein	16	Core	hoi102_00690	hoi103_00686	hoi104_00604	hoi108_00639	hoi110_00987	hoi111_01067	hoi112_00971	hoi113_01112	hoi114_01553	hoi115_00689	hoi116_01460	hoi105_01377	hoi107_01404	hoi109_01336	hoi117_01736	hoi106_01437
677	Disulfide bond formation protein D precursor	16	Core	hoi102_00691	hoi103_00687	hoi104_00605	hoi108_00640	hoi110_00988	hoi111_01068	hoi112_00972	hoi113_01113	hoi114_01554	hoi115_00690	hoi116_01461	hoi105_01378	hoi107_01405	hoi109_01337	hoi117_01737	hoi106_01438
678	Cytochrome C biogenesis protein transmembran	16	Core	hoi102_00692	hoi103_00688	hoi104_00606	hoi108_00641	hoi110_00989	hoi111_01069	hoi112_00973	hoi113_01114	hoi114_01555	hoi115_00691	hoi116_01462	hoi105_01379	hoi107_01406	hoi109_01338	hoi117_01738	hoi106_01439
679	UDP-glucose 4-epimerase	16	Core	hoi102_00693	hoi103_00689	hoi104_00607	hoi108_00642	hoi110_00990	hoi111_01070	hoi112_00974	hoi113_01115	hoi114_01556	hoi115_00692	hoi116_01463	hoi105_01380	hoi107_01407	hoi109_01339	hoi117_01739	hoi106_01440
680	Inner membrane protein YohD	16	Core	hoi102_00694	hoi103_00690	hoi104_00608	hoi108_00643	hoi110_00991	hoi111_01071	hoi112_00975	hoi113_01116	hoi114_01557	hoi115_00693	hoi116_01464	hoi105_01381	hoi107_01408	hoi109_01340	hoi117_01740	hoi106_01441
681	Glutamyl-Q tRNA(Asp) synthetase	16	Core	hoi102_00695	hoi103_00691	hoi104_00609	hoi108_00644	hoi110_00992	hoi111_01072	hoi112_00976	hoi113_01117	hoi114_01558	hoi115_00694	hoi116_01465	hoi105_01382	hoi107_01409	hoi109_01341	hoi117_01741	hoi106_01442
682	Suppressor of fused protein (SUFU)	16	Core	hoi102_00696	hoi103_00692	hoi104_00610	hoi108_00645	hoi110_00993	hoi111_01073	hoi112_00977	hoi113_01118	hoi114_01559	hoi115_00695	hoi116_01466	hoi105_01383	hoi107_01410	hoi109_01342	hoi117_01742	hoi106_01443
683	Putative aminotransferase/MSMEI_6121	16	Core	hoi102_00697	hoi103_00693	hoi104_00611	hoi108_00646	hoi110_00994	hoi111_01074	hoi112_00978	hoi113_01119	hoi114_01560	hoi115_00696	hoi116_01467	hoi105_01384	hoi107_01411	hoi109_01343	hoi117_01743	hoi106_01444
684	Anti-sigma-K factor RskA	16	Core	hoi102_00698	hoi103_00694	hoi104_00612	hoi108_00647	hoi110_00995	hoi111_01075	hoi112_00979	hoi113_01120	hoi114_01561	hoi115_00697	hoi116_01468	hoi105_01385	hoi107_01412	hoi109_01344	hoi117_01744	hoi106_01445
685	Immunogenic protein MPT70 precursor	16	Core	hoi102_00699	hoi103_00695	hoi104_00613	hoi108_00648	hoi110_00996	hoi111_01076	hoi112_00980	hoi113_01121	hoi114_01562	hoi115_00698	hoi116_01469	hoi105_01386	hoi107_01413	hoi109_01345	hoi117_01745	hoi106_01446
686	hypothetical protein	16	Core	hoi102_00700	hoi103_00696	hoi104_00614	hoi108_00649	hoi110_00997	hoi111_01077	hoi112_00981	hoi113_01122	hoi114_01563	hoi115_00699	hoi116_01470	hoi105_01387	hoi107_01414	hoi109_01346	hoi117_01746	hoi106_01447
687	hypothetical protein	16	Core	hoi102_00701	hoi103_00697	hoi104_00615	hoi108_00650	hoi110_00998	hoi111_01078	hoi112_00982	hoi113_01123	hoi114_01564	hoi115_00700	hoi116_01471	hoi105_01388	hoi107_01415	hoi109_01347	hoi117_01747	hoi106_01448
688	putative ABC transporter ATP-binding protein Yxl	16	Core	hoi102_00702	hoi103_00698	hoi104_00616	hoi108_00651	hoi110_00999	hoi111_01079	hoi112_00983	hoi113_01124	hoi114_01565	hoi115_00701	hoi116_01472	hoi105_01389	hoi107_01416	hoi109_01348	hoi117_01748	hoi106_01449
689	hypothetical protein	16	Core	hoi102_00703	hoi103_00699	hoi104_00617	hoi108_00652	hoi110_01000	hoi111_01080	hoi112_00984	hoi113_01125	hoi114_01566	hoi115_00702	hoi116_01473	hoi105_01390	hoi107_01417	hoi109_01349	hoi117_01749	hoi106_01450
690	Putrescine oxidase	16	Core	hoi102_00705	hoi103_00701	hoi104_00619	hoi108_00654	hoi110_01002	hoi111_01082	hoi112_00986	hoi113_01127	hoi114_01568	hoi115_00704	hoi116_01475	hoi105_01392	hoi107_01419	hoi109_01351	hoi117_01751	hoi106_01452
691	hypothetical protein	16	Core	hoi102_00706	hoi103_00702	hoi104_00620	hoi108_00655	hoi110_01003	hoi111_01083	hoi112_00987	hoi113_01128	hoi114_01569	hoi115_00705	hoi116_01476	hoi105_01394	hoi107_01421	hoi109_01353	hoi117_01753	hoi106_01453
692	hypothetical protein	16	Core	hoi102_00707	hoi103_00703	hoi104_00621	hoi108_00656	hoi110_01004	hoi111_01084	hoi112_00988	hoi113_01129	hoi114_01570	hoi115_00706	hoi116_01477	hoi105_01395	hoi107_01422	hoi109_01354	hoi117_01754	hoi106_01454
693	Transcriptional regulator PadR-like family proteir	16	Core	hoi102_00708	hoi103_00704	hoi104_00622	hoi108_00657	hoi110_01005	hoi111_01085	hoi112_00989	hoi113_01130	hoi114_01571	hoi115_00707	hoi116_01478	hoi105_01396	hoi107_01423	hoi109_01355	hoi117_01755	hoi106_01455
694	hypothetical protein	15	Accessory	hoi102_00709	hoi103_00705	hoi104_00623	hoi108_00658	hoi110_01006	hoi111_01086	hoi112_00990	hoi113_01131	hoi114_01572	hoi115_00708	hoi116_01479	hoi105_01398	hoi107_01425	hoi109_01357	hoi117_01757	
695	DNA polymerase III subunit tau	16	Core	hoi102_00711	hoi103_00707	hoi104_00625	hoi108_00660	hoi110_01008	hoi111_01088	hoi112_00992	hoi113_01133	hoi114_01574	hoi115_00710	hoi116_01481	hoi105_01400	hoi107_01427	hoi109_01359	hoi117_01759	hoi106_01457
696	Adenylate cyclase 2	16	Core	hoi102_00712	hoi103_00708	hoi104_00626	hoi108_00661	hoi110_01009	hoi111_01089	hoi112_00993	hoi113_01134	hoi114_01575	hoi115_00711	hoi116_01482	hoi105_01401	hoi107_01428	hoi109_01360	hoi117_01760	hoi106_01458
697	hypothetical protein	16	Core	hoi102_00713	hoi103_00709	hoi104_00627	hoi108_00662	hoi110_01010	hoi111_01090	hoi112_00994	hoi113_01135	hoi114_01576	hoi115_00712	hoi116_01483	hoi105_01402	hoi107_01429	hoi109_01361	hoi117_01761	hoi106_01459
698	DNA topoisomerase 1	16	Core	hoi102_00714	hoi103_00710	hoi104_00628	hoi108_00663	hoi110_01011	hoi111_01091	hoi112_00995	hoi113_01136	hoi114_01577	hoi115_00713	hoi116_01484	hoi105_01403	hoi107_01430	hoi109_01362	hoi117_01762	hoi106_01460
699	putative cold shock protein A	16	Core	hoi102_00715	hoi103_00711	hoi104_00629	hoi108_00664	hoi110_01012	hoi111_01092	hoi112_00996	hoi113_01137	hoi114_01578	hoi115_00714	hoi116_01485	hoi105_01404	hoi107_01431	hoi109_01363	hoi117_01763	hoi106_01461
700	putative ATP-dependent helicase Lhr	16	Core	hoi102_00716	hoi103_00712	hoi104_00630	hoi108_00665	hoi110_01013	hoi111_01093	hoi112_00997	hoi113_01138	hoi114_01579	hoi115_00715	hoi116_01486	hoi105_01405	hoi107_01432	hoi109_01364	hoi117_01764	hoi106_01462
701	hypothetical protein	16	Core	hoi102_00717	hoi103_00713	hoi104_00631	hoi108_00666	hoi110_01014	hoi111_01094	hoi112_00998	hoi113_01139	hoi114_01580	hoi115_00716	hoi116_01487	hoi105_01406	hoi107_01433	hoi109_01365	hoi117_01765	hoi106_01463
702	hypothetical protein	16	Core	hoi102_00718	hoi103_00714	hoi104_00632	hoi108_00667	hoi110_01015	hoi111_01095	hoi112_00999	hoi113_01140	hoi114_01581	hoi115_00717	hoi116_01488	hoi105_01407	hoi107_01434	hoi109_01366	hoi117_01766	hoi106_01464
703	hypothetical protein	16	Core	hoi102_00719	hoi103_00715	hoi104_00633	hoi108_00668	hoi110_01016	hoi111_01096	hoi112_01000	hoi113_01141	hoi114_01582	hoi115_00718	hoi116_01489	hoi105_01408	hoi107_01435	hoi109_01367	hoi117_01767	hoi106_01465
704	Bacterial type II secretion system protein F doma	16	Core	hoi102_00720	hoi103_00716	hoi104_00634	hoi108_00669	hoi110_01017	hoi111_01097	hoi112_01001	hoi113_01142	hoi114_01583	hoi115_00719	hoi116_01490	hoi105_01409	hoi107_01436	hoi109_01368	hoi117_01768	hoi106_01466
705	Bacterial type II secretion system protein F doma	16	Core	hoi102_00721	hoi103_00717	hoi104_00635	hoi108_00670	hoi110_01018	hoi111_01098	hoi112_01002	hoi113_01143	hoi114_01584	hoi115_00720	hoi116_01491	hoi105_01410	hoi107_01437	hoi109_01369	hoi117_01769	hoi106_01467
706	Putative conjugal transfer protein/MT3759	16	Core	hoi102_00722	hoi103_00718	hoi104_00636	hoi108_00671	hoi110_01019	hoi111_01099	hoi112_01003	hoi113_01144	hoi114_01585	hoi115_00721	hoi116_01492	hoi105_01411	hoi107_01438	hoi109_01370	hoi117_01770	hoi106_01468
707	hypothetical protein	16	Core	hoi102_00723	hoi103_00719	hoi104_00637	hoi108_00672	hoi110_01020	hoi111_01100	hoi112_01004	hoi113_01145	hoi114_01586	hoi115_00722	hoi116_01493	hoi105_01412	hoi107_01439	hoi109_01371	hoi117_01771	hoi106_01469
708	drug efflux system protein MdtG	14	Accessory	hoi102_00724	hoi103_00761		hoi108_02140	hoi110_01494		hoi112_00531	hoi113_00673	hoi114_00721	hoi115_01022	hoi116_01023	hoi105_00804	hoi107_00639	hoi109_00728	hoi117_01147	hoi106_01221
709	HTH-type transcriptional regulator KmtR	16	Core	hoi102_00725	hoi103_00762	hoi104_00744	hoi108_02139	hoi110_01495	hoi111_01561	hoi112_00532	hoi113_00674	hoi114_00722	hoi115_01023	hoi116_01024	hoi105_00803	hoi107_00638	hoi109_00727	hoi117_01146	hoi106_01220
710	nicotinamidase/pyrazinamidase	16	Core	hoi102_00726	hoi103_00763	hoi104_00745	hoi108_02138	hoi110_01496	hoi111_01560	hoi112_00533	hoi113_00675	hoi114_00723	hoi115_01024	hoi116_01025	hoi105_00802	hoi107_00637	hoi109_00726	hoi117_01145	hoi106_01219
711	Putative cysteine protease YraA	16	Core	hoi102_00727	hoi103_00764	hoi104_00746	hoi108_02137	hoi110_01497	hoi111_01559	hoi112_00534	hoi113_00676	hoi114_00724	hoi115_01025	hoi116_01026	hoi105_00801	hoi107_00636	hoi109_00725	hoi117_01144	hoi106_01218
712	hypothetical protein	16	Core	hoi102_00728	hoi103_00765	hoi104_00747	hoi108_02136	hoi110_01498	hoi111_01558	hoi112_00535	hoi113_00677	hoi114_00725	hoi115_01026	hoi116_01027	hoi105_00800	hoi107_00635	hoi109_00724	hoi117_01143	hoi106_01217
713	Sodium:neurotransmitter symporter family prote	16	Core	hoi102_00729	hoi103_00766	hoi104_00748	hoi108_02135	hoi110_01499	hoi111_01557	hoi112_00536	hoi113_00678	hoi114_00726	hoi115_01027	hoi116_01028	hoi105_00799	hoi107_00634	hoi109_00723	hoi117_01142	hoi106_01216
714	Inner membrane protein YedI	16	Core	hoi102_00730	hoi103_00767	hoi104_00749	hoi108_02134	hoi110_01500	hoi111_01556	hoi112_00537	hoi113_00679	hoi114_00727	hoi115_01028	hoi116_01029	hoi105_00798	hoi107_00633	hoi109_00722	hoi117_01141	hoi106_01215
715	Putative HMP/thiamine permease protein YkoE	16	Core	hoi102_00731	hoi103_00768	hoi104_00750	hoi108_02133	hoi110_01501	hoi111_01555	hoi112_00538	hoi113_00680	hoi114_00728	hoi115_01029	hoi116_01030	hoi105_00797	hoi107_00632	hoi109_00721	hoi117_01140	hoi106_01214
716	Putative HMP/thiamine import ATP-binding prot	16	Core	hoi102_00732	hoi103_00769	hoi104_00751	hoi108_02132	hoi110_01502	hoi111_01554	hoi112_00539	hoi113_00681	hoi114_00729	hoi115_01030	hoi116_01031	hoi105_00796	hoi107_00631	hoi109_00720	hoi117_01139	hoi106_01213
717	Putative HMP/thiamine permease protein YkoC	16	Core	hoi102_00733	hoi103_00770	hoi104_00752	hoi108_02131	hoi110_01503	hoi111_01553	hoi112_00540	hoi113_00682	hoi114_00730	hoi115_01031	hoi116_01032	hoi105_00795	hoi107_00630	hoi109_00719	hoi117_01138	hoi106_01212

718	hypothetical protein	16	Core	hoi102_00734	hoi103_00771	hoi104_00753	hoi108_02130	hoi110_01504	hoi111_01552	hoi112_00541	hoi113_00683	hoi114_00731	hoi115_01032	hoi116_01033	hoi105_00794	hoi107_00629	hoi109_00718	hoi117_01137	hoi106_01211
719	Periplasmic murein peptide-binding protein prec	16	Core	hoi102_00735	hoi103_00772	hoi104_00754	hoi108_02129	hoi110_01505	hoi111_01551	hoi112_00542	hoi113_00684	hoi114_00732	hoi115_01033	hoi116_01034	hoi105_00793	hoi107_00628	hoi109_00717	hoi117_01136	hoi106_01210
720	Dipeptide transport system permease protein Dp	16	Core	hoi102_00736	hoi103_00773	hoi104_00755	hoi108_02128	hoi110_01506	hoi111_01550	hoi112_00543	hoi113_00685	hoi114_00733	hoi115_01034	hoi116_01035	hoi105_00792	hoi107_00627	hoi109_00716	hoi117_01135	hoi106_01209
721	Oligopeptide transport system permease protein	16	Core	hoi102_00737	hoi103_00774	hoi104_00756	hoi108_02127	hoi110_01507	hoi111_01549	hoi112_00544	hoi113_00686	hoi114_00734	hoi115_01035	hoi116_01036	hoi105_00791	hoi107_00626	hoi109_00715	hoi117_01134	hoi106_01208
722	Glutathione import ATP-binding protein GsiA	16	Core	hoi102_00738	hoi103_00775	hoi104_00757	hoi108_02126	hoi110_01508	hoi111_01548	hoi112_00545	hoi113_00687	hoi114_00735	hoi115_01036	hoi116_01037	hoi105_00790	hoi107_00625	hoi109_00714	hoi117_01133	hoi106_01207
723	Arsenate reductase	16	Core	hoi102_00739	hoi103_00776	hoi104_00758	hoi108_02125	hoi110_01509	hoi111_01547	hoi112_00546	hoi113_00688	hoi114_00736	hoi115_01037	hoi116_01038	hoi105_00789	hoi107_00624	hoi109_00713	hoi117_01132	hoi106_01206
724	hypothetical protein	16	Core	hoi102_00740	hoi103_00777	hoi104_00759	hoi108_02124	hoi110_01510	hoi111_01546	hoi112_00547	hoi113_00689	hoi114_00737	hoi115_01038	hoi116_01039	hoi105_00788	hoi107_00623	hoi109_00712	hoi117_01131	hoi106_01205
725	hypothetical protein	16	Core	hoi102_00741	hoi103_00778	hoi104_00760	hoi108_02123	hoi110_01511	hoi111_01545	hoi112_00548	hoi113_00690	hoi114_00738	hoi115_01039	hoi116_01040	hoi105_00787	hoi107_00622	hoi109_00711	hoi117_01130	hoi106_01204
726	GTP-binding protein TypA/BipA	16	Core	hoi102_00742	hoi103_00779	hoi104_00761	hoi108_02122	hoi110_01512	hoi111_01544	hoi112_00549	hoi113_00691	hoi114_00739	hoi115_01040	hoi116_01041	hoi105_00786	hoi107_00621	hoi109_00710	hoi117_01129	hoi106_01203
727	putative monoacyl phosphatidylinositol tetramar	16	Core	hoi102_00743	hoi103_00780	hoi104_00762	hoi108_02121	hoi110_01513	hoi111_01543	hoi112_00550	hoi113_00692	hoi114_00740	hoi115_01041	hoi116_01042	hoi105_00785	hoi107_00620	hoi109_00709	hoi117_01128	hoi106_01202
728	1D-myo-inositol 2-acetamido-2-deoxy-alpha-D-g	16	Core	hoi102_00744	hoi103_00781	hoi104_00763	hoi108_02120	hoi110_01514	hoi111_01542	hoi112_00551	hoi113_00693	hoi114_00741	hoi115_01042	hoi116_01043	hoi105_00784	hoi107_00619	hoi109_00708	hoi117_01127	hoi106_01201
729	hypothetical protein	16	Core	hoi102_00745	hoi103_00782	hoi104_00764	hoi108_02119	hoi110_01515	hoi111_01541	hoi112_00552	hoi113_00694	hoi114_00742	hoi115_01043	hoi116_01044	hoi105_00783	hoi107_00618	hoi109_00707	hoi117_01126	hoi106_01200
730	Ferredoxin	16	Core	hoi102_00746	hoi103_00783	hoi104_00765	hoi108_02118	hoi110_01516	hoi111_01540	hoi112_00553	hoi113_00695	hoi114_00743	hoi115_01044	hoi116_01045	hoi105_00782	hoi107_00617	hoi109_00706	hoi117_01125	hoi106_01199
731	LL-diaminopimelate aminotransferase	16	Core	hoi102_00747	hoi103_00784	hoi104_00766	hoi108_02117	hoi110_01517	hoi111_01539	hoi112_00554	hoi113_00696	hoi114_00744	hoi115_01045	hoi116_01046	hoi105_00781	hoi107_00616	hoi109_00705	hoi117_01124	hoi106_01198
732	GtrA-like protein	16	Core	hoi102_00748	hoi103_00785	hoi104_00767	hoi108_02116	hoi110_01518	hoi111_01538	hoi112_00555	hoi113_00697	hoi114_00745	hoi115_01046	hoi116_01047	hoi105_00780	hoi107_00615	hoi109_00704	hoi117_01123	hoi106_01197
733	GABA permease	16	Core	hoi102_00749	hoi103_00786	hoi104_00768	hoi108_02115	hoi110_01519	hoi111_01537	hoi112_00556	hoi113_00698	hoi114_00746	hoi115_01047	hoi116_01048	hoi105_00779	hoi107_00614	hoi109_00703	hoi117_01122	hoi106_01196
734	2,3,4,5-tetrahydropyridine-2,6-dicarboxylate N-s	16	Core	hoi102_00750	hoi103_00787	hoi104_00769	hoi108_02114	hoi110_01520	hoi111_01536	hoi112_00557	hoi113_00699	hoi114_00747	hoi115_01048	hoi116_01049	hoi105_00778	hoi107_00613	hoi109_00702	hoi117_01121	hoi106_01195
735	LOG family protein ORF6 in fasciation locus	16	Core	hoi102_00751	hoi103_00788	hoi104_00770	hoi108_02113	hoi110_01521	hoi111_01535	hoi112_00558	hoi113_00700	hoi114_00748	hoi115_01049	hoi116_01050	hoi105_00777	hoi107_00612	hoi109_00701	hoi117_01120	hoi106_01194
736	Inactive dihydropteroate synthase 2	16	Core	hoi102_00752	hoi103_00789	hoi104_00771	hoi108_02112	hoi110_01522	hoi111_01534	hoi112_00559	hoi113_00701	hoi114_00749	hoi115_01050	hoi116_01051	hoi105_00776	hoi107_00611	hoi109_00700	hoi117_01119	hoi106_01193
737	Glucosyl-3-phosphoglycerate synthase	16	Core	hoi102_00753	hoi103_00790	hoi104_00772	hoi108_02111	hoi110_01523	hoi111_01533	hoi112_00560	hoi113_00702	hoi114_00750	hoi115_01051	hoi116_01052	hoi105_00775	hoi107_00610	hoi109_00699	hoi117_01118	hoi106_01192
738	23S rRNA (guanine(748)-N(1))-methyltransferase	16	Core	hoi102_00754	hoi103_00791	hoi104_00773	hoi108_02110	hoi110_01524	hoi111_01532	hoi112_00561	hoi113_00703	hoi114_00751	hoi115_01052	hoi116_01053	hoi105_00774	hoi107_00609	hoi109_00698	hoi117_01117	hoi106_01191
739	Sucrose-6-phosphate hydrolase	16	Core	hoi102_00755	hoi103_00792	hoi104_00774	hoi108_02109	hoi110_01525	hoi111_01531	hoi112_00562	hoi113_00704	hoi114_00752	hoi115_01053	hoi116_01054	hoi105_00773	hoi107_00608	hoi109_00697	hoi117_01116	hoi106_01190
740	Alpha-acetolactate decarboxylase	16	Core	hoi102_00756	hoi103_00793	hoi104_00775	hoi108_02108	hoi110_01526	hoi111_01530	hoi112_00563	hoi113_00705	hoi114_00753	hoi115_01054	hoi116_01055	hoi105_00772	hoi107_00607	hoi109_00696	hoi117_01115	hoi106_01189
741	Capsular glucan synthase	16	Core	hoi102_00757	hoi103_00794	hoi104_00776	hoi108_02107	hoi110_01527	hoi111_01529	hoi112_00564	hoi113_00706	hoi114_00754	hoi115_01055	hoi116_01056	hoi105_00771	hoi107_00606	hoi109_00695	hoi117_01114	hoi106_01188
742	Glucose-1-phosphate adenylyltransferase	16	Core	hoi102_00758	hoi103_00795	hoi104_00777	hoi108_02106	hoi110_01528	hoi111_01528	hoi112_00565	hoi113_00707	hoi114_00755	hoi115_01056	hoi116_01057	hoi105_00770	hoi107_00605	hoi109_00694	hoi117_01113	hoi106_01187
743	Putative O-methyltransferase/MSMEI_4947	16	Core	hoi102_00759	hoi103_00796	hoi104_00778	hoi108_02105	hoi110_01529	hoi111_01527	hoi112_00566	hoi113_00708	hoi114_00756	hoi115_01057	hoi116_01058	hoi105_00769	hoi107_00604	hoi109_00693	hoi117_01112	hoi106_01186
744	ECF RNA polymerase sigma factor SigE	16	Core	hoi102_00760	hoi103_00797	hoi104_00779	hoi108_02104	hoi110_01530	hoi111_01526	hoi112_00567	hoi113_00709	hoi114_00757	hoi115_01058	hoi116_01059	hoi105_00768	hoi107_00603	hoi109_00692	hoi117_01111	hoi106_01185
745	Anti-sigma-E factor RseA	16	Core	hoi102_00761	hoi103_00798	hoi104_00780	hoi108_02103	hoi110_01531	hoi111_01525	hoi112_00568	hoi113_00710	hoi114_00758	hoi115_01059	hoi116_01060	hoi105_00767	hoi107_00602	hoi109_00691	hoi117_01110	hoi106_01184
746	sec-independent translocase	16	Core	hoi102_00762	hoi103_00799	hoi104_00781	hoi108_02102	hoi110_01532	hoi111_01524	hoi112_00569	hoi113_00711	hoi114_00759	hoi115_01060	hoi116_01061	hoi105_00766	hoi107_00601	hoi109_00690	hoi117_01109	hoi106_01183
747	Flagellum site-determining protein YlxH	16	Core	hoi102_00763	hoi103_00800	hoi104_00782	hoi108_02101	hoi110_01533	hoi111_01523	hoi112_00570	hoi113_00712	hoi114_00760	hoi115_01061	hoi116_01062	hoi105_00765	hoi107_00600	hoi109_00689	hoi117_01108	hoi106_01182
748	hypothetical protein	16	Core	hoi102_00764	hoi103_00801	hoi104_00783	hoi108_02100	hoi110_01534	hoi111_01522	hoi112_00571	hoi113_00713	hoi114_00761	hoi115_01062	hoi116_01063	hoi105_00764	hoi107_00599	hoi109_00688	hoi117_01107	hoi106_01181
749	Magnesium transporter MgtE	16	Core	hoi102_00765	hoi103_00802	hoi104_00784	hoi108_02099	hoi110_01535	hoi111_01521	hoi112_00572	hoi113_00714	hoi114_00762	hoi115_01063	hoi116_01064	hoi105_00763	hoi107_00598	hoi109_00687	hoi117_01106	hoi106_01180
750	preprotein translocase subunit SecG	16	Core	hoi102_00766	hoi103_00803	hoi104_00785	hoi108_02098	hoi110_01536	hoi111_01520	hoi112_00573	hoi113_00715	hoi114_00763	hoi115_01064	hoi116_01065	hoi105_00762	hoi107_00597	hoi109_00686	hoi117_01105	hoi106_01179
751	Magnesium transport protein CorA	16	Core	hoi102_00767	hoi103_00804	hoi104_00786	hoi108_02097	hoi110_01537	hoi111_01519	hoi112_00574	hoi113_00716	hoi114_00764	hoi115_01065	hoi116_01066	hoi105_00761	hoi107_00596	hoi109_00685	hoi117_01104	hoi106_01178
752	hypothetical protein	16	Core	hoi102_00768	hoi103_00805	hoi104_00787	hoi108_02096	hoi110_01538	hoi111_01518	hoi112_00575	hoi113_00717	hoi114_00765	hoi115_01066	hoi116_01067	hoi105_00760	hoi107_00595	hoi109_00684	hoi117_01103	hoi106_01177
753	2-oxoglutarate dehydrogenase E1/E2 componen	16	Core	hoi102_00769	hoi103_00806	hoi104_00788	hoi108_02095	hoi110_01539	hoi111_01517	hoi112_00576	hoi113_00718	hoi114_00766	hoi115_01067	hoi116_01068	hoi105_00759	hoi107_00594	hoi109_00683	hoi117_01102	hoi106_01176
754	Putative multidrug export ATP-binding/permease	16	Core	hoi102_00770	hoi103_00807	hoi104_00789	hoi108_01336	hoi110_01540	hoi111_01516	hoi112_00577	hoi113_00719	hoi114_00767	hoi115_01068	hoi116_01069	hoi105_00758	hoi107_00593	hoi109_00682	hoi117_01101	hoi106_01819
755	hypothetical protein	16	Core	hoi102_00771	hoi103_00808	hoi104_00790	hoi108_01337	hoi110_01541	hoi111_01515	hoi112_00578	hoi113_00720	hoi114_00768	hoi115_01069	hoi116_01070	hoi105_00757	hoi107_00592	hoi109_00681	hoi117_01100	hoi106_01818
756	Para-nitrobenzyl esterase	16	Core	hoi102_00772	hoi103_00809	hoi104_00791	hoi108_01338	hoi110_01542	hoi111_01514	hoi112_00579	hoi113_00721	hoi114_00769	hoi115_01070	hoi116_01071	hoi105_00756	hoi107_00591	hoi109_00680	hoi117_01099	hoi106_01817
757	hypothetical protein	16	Core	hoi102_00773	hoi103_00810	hoi104_00792	hoi108_01339	hoi110_01543	hoi111_01513	hoi112_00580	hoi113_00722	hoi114_00770	hoi115_01071	hoi116_01072	hoi105_00755	hoi107_00590	hoi109_00679	hoi117_01098	hoi106_01816
758	hypothetical protein	16	Core	hoi102_00774	hoi103_00811	hoi104_00793	hoi108_01340	hoi110_01544	hoi111_01512	hoi112_00581	hoi113_00723	hoi114_00771	hoi115_01072	hoi116_01073	hoi105_00754	hoi107_00589	hoi109_00678	hoi117_01097	hoi106_01815
759	hypothetical protein	16	Core	hoi102_00775	hoi103_00812	hoi104_00794	hoi108_01341	hoi110_01545	hoi111_01511	hoi112_00582	hoi113_00724	hoi114_00772	hoi115_01073	hoi116_01074	hoi105_00753	hoi107_00588	hoi109_00677	hoi117_01096	hoi106_01814
760	Tetracycline resistance protein, class C	16	Core	hoi102_00776	hoi103_00813	hoi104_00795	hoi108_01342	hoi110_01546	hoi111_01510	hoi112_00583	hoi113_00725	hoi114_00773	hoi115_01074	hoi116_01075	hoi105_00752	hoi107_00587	hoi109_00676	hoi117_01095	hoi106_01813
761	ATP-dependent RNA helicase DeaD	16	Core	hoi102_00777	hoi103_00814	hoi104_00796	hoi108_01343	hoi110_01547	hoi111_01509	hoi112_00584	hoi113_00726	hoi114_00774	hoi115_01075	hoi116_01076	hoi105_00751	hoi107_00586	hoi109_00675	hoi117_01094	hoi106_01812
762	hypothetical protein	16	Core	hoi102_00778	hoi103_00815	hoi104_00797	hoi108_01344	hoi110_01548	hoi111_01508	hoi112_00585	hoi113_00727	hoi114_00775	hoi115_01076	hoi116_01077	hoi105_00750	hoi107_00585	hoi109_00674	hoi117_01093	hoi106_01811
763	hypothetical protein	16	Core	hoi102_00779	hoi103_00816	hoi104_00798	hoi108_01345	hoi110_01549	hoi111_01507	hoi112_00586	hoi113_00728	hoi114_00776	hoi115_01077	hoi116_01078	hoi105_00749	hoi107_00584	hoi109_00673	hoi117_01092	hoi106_01810
764	Sodium/proline symporter	16	Core	hoi102_00780	hoi103_00817	hoi104_00799	hoi108_01346	hoi110_01550	hoi111_01506	hoi112_00587	hoi113_00729	hoi114_00777	hoi115_01078	hoi116_01079	hoi105_00748	hoi107_00583	hoi109_00672	hoi117_01091	hoi106_01809
765	ATP-dependent helicase HepA	16	Core	hoi102_00781	hoi103_00818	hoi104_00800	hoi108_01347	hoi110_01551	hoi111_01505	hoi112_00588	hoi113_00730	hoi114_00778	hoi115_01079	hoi116_01080	hoi105_00747	hoi107_00582	hoi109_00671	hoi117_01090	hoi106_01808
766	hypothetical protein	16	Core	hoi102_00782	hoi103_00819	hoi104_00801	hoi108_01348	hoi110_01552	hoi111_01504	hoi112_00589	hoi113_00731	hoi114_00779	hoi115_01080	hoi116_01081	hoi105_00746	hoi107_00581	hoi109_00670	hoi117_01089	hoi106_01807
767	putative metallophosphoesterase YhaO	16	Core	hoi102_00783	hoi103_00820	hoi104_00802	hoi108_01349	hoi110_01553	hoi111_01503	hoi112_00590	hoi113_00732	hoi114_00780	hoi115_01081	hoi116_01082	hoi105_00745	hoi107_00580	hoi109_00669	hoi117_01088	hoi106_01806
768	chromosome segregation protein	16	Core	hoi102_00784	hoi103_00821	hoi104_00803	hoi108_01350	hoi110_01554	hoi111_01502	hoi112_00591	hoi113_00733	hoi114_00781	hoi115_01082	hoi116_01083	hoi105_00744	hoi107_00579	hoi109_00668	hoi117_01087	hoi106_01805
769	transcriptional repressor MprA	16	Core	hoi102_00785	hoi103_00822	hoi104_00804	hoi108_01351	hoi110_01555	hoi111_01501	hoi112_00592	hoi113_00734	hoi114_00782	hoi115_01083	hoi116_01084	hoi105_00743	hoi107_00578	hoi109_00667	hoi117_01086	hoi106_01804
770	Lactate utilization protein C	16	Core	hoi102_00787	hoi103_00824	hoi104_00806	hoi108_01353	hoi110_01557	hoi111_01499	hoi112_00594	hoi113_00736	hoi114_00784	hoi115_01085	hoi116_01086	hoi105_00741	hoi107_00576	hoi109_00665	hoi117_01084	hoi106_01802
771	Lactate utilization protein B	16	Core	hoi102_00788	hoi103_00825	hoi104_00807	hoi108_01354	hoi110_01558	hoi111_01498	hoi112_00595	hoi113_00737	hoi114_00785	hoi115_01086	hoi116_01087	hoi105_00740	hoi107_00575	hoi109_00664	hoi117_01083	hoi106_01801
772	Lactate utilization protein A	16	Core	hoi102_00789	hoi103_00826	hoi104_00808	hoi108_01355	hoi110_01559	hoi111_01497	hoi112_00596	hoi113_00738	hoi114_00786	hoi115_01087	hoi116_01088	hoi105_00739	hoi107_00574	hoi109_00663	hoi117_01082	hoi106_01800
773	L-lactate permease	16	Core	hoi102_00790	hoi103_00827	hoi104_00809	hoi108_01356	hoi110_01560	hoi111_01496	hoi112_00597	hoi113_00739	hoi114_00787	hoi115_01088	hoi116_01089	hoi105_00738	hoi107_00573	hoi109_00662	hoi117_01081	hoi106_01799
774	Arginine--tRNA ligase	16	Core	hoi102_00791	hoi103_00828	hoi104_00810	hoi108_01357	hoi110_01561	hoi111_01495	hoi112_00598	hoi113_00740	hoi114_00788	hoi115_01089	hoi116_01090	hoi105_00737	hoi107_00572	hoi109_00661	hoi117_01080	hoi106_01798
775	Diaminopimelate decarboxylase	16	Core	hoi102_00792	hoi103_00829	hoi104_00811	hoi108_01358	hoi110_01562	hoi111_01494	hoi112_00599	hoi113_00741	hoi114_00789	hoi115_01090	hoi116_01091	hoi105_00736	hoi107_00571	hoi109_00660	hoi117_01079	hoi106_01797
776	Homoserine dehydrogenase	16	Core	hoi102_00793	hoi103_00830	hoi104_00812	hoi108_01359	hoi110_01563	hoi111_01493	hoi112_00600	hoi113_00742	hoi114_00790	hoi115_01091	hoi116_01092	hoi105_00735	hoi107_00570	hoi109_00659	hoi117_01078	hoi106_01796
777	Homoserine kinase	16	Core	hoi102_00794	hoi103_00831	hoi104_00813	hoi108_01360	hoi110_01564	hoi111_01492	hoi112_00601	hoi113_00743	hoi114_00791	hoi115_01092	hoi116_01093	hoi105_00734	hoi107_00569	hoi109_00658	hoi117_01077	hoi106_01795
778	hypothetical protein	16	Core	hoi102_00795	hoi103_00832	hoi104_00814	hoi108_01361	hoi110_01565	hoi111_01491	hoi112_00602	hoi113_00744	hoi114_00792	hoi115_01093	hoi116_01094	hoi105_00733	hoi107_00568	hoi109_00657	hoi117_01076	hoi106_01794
779	Putative 2-aminoethylphosphonate import ATP-b	16	Core	hoi102_00796	hoi103_00833	hoi104_00815	hoi108_01362	hoi110_01566	hoi111_01490	hoi112_00603	hoi113_00745	hoi114_00793	hoi115_01094	hoi116_01095	hoi105_00732	hoi107_00567	hoi109_00656	hoi117_01075	hoi106_01793
780	Molybdate-binding periplasmic protein precurso	16	Core	hoi102_00797	hoi103_00834	hoi104_00816	hoi108_01363	hoi110_01567	hoi111_01489	hoi112_00604	hoi113_00746	hoi114_00794	hoi115_01095	hoi116_01096	hoi105_00731	hoi107_00566	hoi109_00655	hoi117_01074	hoi106_01792
781	Molybdenum cofactor biosynthesis protein B	16	Core	hoi102_00798	hoi103_00835	hoi104_00817	hoi108_01364	hoi110_01568	hoi111_01488	hoi112_00605	hoi113_00747	hoi114_00795	hoi115_01096	hoi116_01097	hoi105_00730	hoi107_00565	hoi109_00654	hoi117_01073	hoi106_01791
782	molybdopterin-guanine dinucleotide biosynthesi	16	Core	hoi102_00799	hoi103_00836	hoi104_00818	hoi108_01365	hoi110_01569	hoi111_01487	hoi112_00606	hoi113_00748	hoi114_00796	hoi115_01097	hoi116_01098	hoi105_00729	hoi107_00564	hoi109_00653	hoi117_01072	hoi106_01790
783	Cyclic pyranopterin monophosphate synthase ac	16	Core	hoi102_00800	hoi103_00837	hoi104_00819	hoi108_01366	hoi110_01570	hoi111_01486	hoi112_00607	hoi113_00749	hoi114_00797	hoi115_01098	hoi116_01099	hoi105_00728	hoi107_00563	hoi109_00652	hoi117_01071	hoi106_01789
784	Molybdopterin molybdenumtransferase	16	Core	hoi102_00801	hoi103_00838	hoi104_00820	hoi108_01367	hoi110_01571	hoi111_01485	hoi112_00608	hoi113_00750	hoi114_00798	hoi115_01099	hoi116_01100	hoi105_00727	hoi107_00562	hoi109_00651	hoi117_01070	hoi106_01788
785	Molybdopterin synthase catalytic subunit	16	Core	hoi102_00802	hoi103_00839	hoi104_00821	hoi108_01368	hoi110_01572	hoi111_01484	hoi112_00609	hoi113_00751	hoi114_00799	hoi115_01100	hoi116_01101	hoi105_00726	hoi107_00561	hoi109_00650	hoi117_01069	hoi106_01787
786	hypothetical protein	16	Core	hoi102_00803	hoi103_00840	hoi104_00822	hoi108_01369	hoi110_01573	hoi111_01483	hoi112_00610	hoi113_00752	hoi114_00800	hoi115_01101	hoi116_01102	hoi105_00725	hoi107_00560	hoi109_00649	hoi117_01068	hoi106_01786
787	Cyclic pyranopterin monophosphate synthase	16	Core	hoi102_00804	hoi103_00841	hoi104_00823	hoi108_01370	hoi110_01574	hoi111_01482	hoi112_00611	hoi113_00753	hoi114_00801	hoi115_01102	hoi116_01103	hoi105_00724	hoi107_00559	hoi109_00648	hoi117_01067	hoi106_01785
788	Long-chain-fatty-acid--CoA ligase	16	Core	hoi102_00805	hoi103_00842	hoi104_00824	hoi108_01371	hoi110_01575	hoi111_01481	hoi112_00612	hoi113_00754	hoi114_00802	hoi115_01103	hoi116_01104	hoi105_00723	hoi107_00558	hoi109_00647	hoi117_01066	hoi106_01784
789	hypothetical protein	16	Core	hoi102_00806	hoi103_00843	hoi104_00825	hoi108_01372	hoi110_01576	hoi111_01480	hoi112_00613	hoi113_00755	hoi114_00803	hoi115_01104	hoi116_01105	hoi105_00722	hoi107_00557	hoi109_00646	hoi117_01065	hoi106_01783
790	Peptide chain release factor 1	16	Core	hoi102_00807	hoi103_00844	hoi104_00826	hoi108_01373	hoi110_01577	hoi111_01479	hoi112_00614	hoi113_00756	hoi114_00804	hoi115_01105	hoi116_01106	hoi105_00721	hoi107_00556	hoi109_00645	hoi117_01064	hoi106_01782
791	Release factor glutamine methyltransferase	16	Core	hoi102_00808	hoi103_00845	hoi104_00827	hoi108_01374	hoi110_01578	hoi111_01478	hoi112_00615	hoi113_00757	hoi114_00805	hoi115_01106	hoi116_01107	hoi105_00720	hoi107_00555	hoi109_00644	hoi117_01063	hoi106_01781
792	Alkaline phosphatase precursor	16	Core	hoi102_00809	hoi103_00846	hoi104_00828	hoi108_01375	hoi110_01579	hoi111_01477	hoi112_00616	hoi113_00758	hoi114_00806	hoi115_01107	hoi116_01108	hoi105_00719	hoi107_00554	hoi109_00643	hoi117_01062	hoi106_01780
793	hypothetical protein	16	Core	hoi102_00810	hoi103_00847	hoi104_00829	hoi108_01376	hoi110_01580	hoi111_01476	hoi112_00617	hoi113_00759	hoi114_00807	hoi115_01108	hoi116_01109	hoi105_00718	hoi107_00553	hoi109_00642	hoi117_01061	hoi106_01779
794	putative permease	16	Core	hoi102_00811	hoi103_00848	hoi104_00830	hoi108_01377	hoi110_01581	hoi111_01475	hoi112_00618	hoi113_00760	hoi114_00808	hoi115_01109	hoi116_01110	hoi105_00717	hoi107_00552	hoi109_00641	hoi117_01060	hoi106_01778
795	Threonylcarbamoyl-AMP synthase	16	Core	hoi102_00812	hoi103_00849	hoi104_00831	hoi108_01378	hoi110_01582	hoi111_01474	hoi112_00619	hoi113_00761	hoi114_00809	hoi115_01110	hoi116_01111	hoi105_00716	hoi107_00551	hoi109_00640	hoi117_01059	hoi106_01777
796	Decaprenyl-phosphate N-acetylglucosaminephos	16	Core	hoi102_00813	hoi103_00850	hoi104_00832	hoi108_01379	hoi110_01583	hoi111_01473	hoi112_00620	hoi113_00762	hoi114_00810	hoi115_01111	hoi116_01112	hoi105_00715	hoi107_00550	hoi109_00639	hoi117_01058	hoi106_01776
797	hypothetical protein	16	Core	hoi102_00814	hoi103_00851	hoi104_00833	hoi108_01380	hoi110_01584	hoi111_01472	hoi112_00621	hoi113_00763	hoi114_00811	hoi115_01112	hoi116_01113	hoi105_00714	hoi107_00549	hoi109_00638	hoi117_01057	hoi106_01775

798	ATP synthase subunit a	16	Core	hoi102_00815	hoi103_00852	hoi104_00834	hoi108_01381	hoi110_01585	hoi111_01471	hoi112_00622	hoi113_00764	hoi114_00812	hoi115_01113	hoi116_01114	hoi105_00712	hoi107_00548	hoi109_00636	hoi117_01056	hoi106_01773
799	ATP synthase subunit c	16	Core	hoi102_00816	hoi103_00853	hoi104_00835	hoi108_01382	hoi110_01586	hoi111_01470	hoi112_00623	hoi113_00765	hoi114_00813	hoi115_01114	hoi116_01115	hoi105_00711	hoi107_00547	hoi109_00635	hoi117_01055	hoi106_01772
800	ATP synthase subunit b	16	Core	hoi102_00817	hoi103_00854	hoi104_00836	hoi108_01383	hoi110_01587	hoi111_01469	hoi112_00624	hoi113_00766	hoi114_00814	hoi115_01115	hoi116_01116	hoi105_00710	hoi107_00546	hoi109_00634	hoi117_01054	hoi106_01771
801	ATP synthase subunit delta	16	Core	hoi102_00818	hoi103_00855	hoi104_00837	hoi108_01384	hoi110_01588	hoi111_01468	hoi112_00625	hoi113_00767	hoi114_00815	hoi115_01116	hoi116_01117	hoi105_00709	hoi107_00545	hoi109_00633	hoi117_01053	hoi106_01770
802	ATP synthase subunit alpha	16	Core	hoi102_00819	hoi103_00856	hoi104_00838	hoi108_01385	hoi110_01589	hoi111_01467	hoi112_00626	hoi113_00768	hoi114_00816	hoi115_01117	hoi116_01118	hoi105_00708	hoi107_00544	hoi109_00632	hoi117_01052	hoi106_01769
803	ATP synthase gamma chain	16	Core	hoi102_00820	hoi103_00857	hoi104_00839	hoi108_01386	hoi110_01590	hoi111_01466	hoi112_00627	hoi113_00769	hoi114_00817	hoi115_01118	hoi116_01119	hoi105_00707	hoi107_00543	hoi109_00631	hoi117_01051	hoi106_01768
804	ATP synthase subunit beta	16	Core	hoi102_00821	hoi103_00858	hoi104_00840	hoi108_01387	hoi110_01591	hoi111_01465	hoi112_00628	hoi113_00770	hoi114_00818	hoi115_01119	hoi116_01120	hoi105_00706	hoi107_00542	hoi109_00630	hoi117_01050	hoi106_01767
805	ATP synthase epsilon chain	16	Core	hoi102_00822	hoi103_00859	hoi104_00841	hoi108_01388	hoi110_01592	hoi111_01464	hoi112_00629	hoi113_00771	hoi114_00819	hoi115_01120	hoi116_01121	hoi105_00705	hoi107_00541	hoi109_00629	hoi117_01049	hoi106_01766
806	hypothetical protein	16	Core	hoi102_00823	hoi103_00860	hoi104_00842	hoi108_01389	hoi110_01593	hoi111_01463	hoi112_00630	hoi113_00772	hoi114_00820	hoi115_01121	hoi116_01122	hoi105_00704	hoi107_00540	hoi109_00628	hoi117_01048	hoi106_01765
807	hypothetical protein	16	Core	hoi102_00824	hoi103_00861	hoi104_00843	hoi108_01390	hoi110_01594	hoi111_01462	hoi112_00631	hoi113_00773	hoi114_00821	hoi115_01122	hoi116_01123	hoi105_00703	hoi107_00539	hoi109_00627	hoi117_01047	hoi106_01764
808	Glyoxalase/Bleomycin resistance protein/Dioxyg	16	Core	hoi102_00825	hoi103_00862	hoi104_00844	hoi108_01391	hoi110_01595	hoi111_01461	hoi112_00632	hoi113_00774	hoi114_00822	hoi115_01123	hoi116_01124	hoi105_00702	hoi107_00538	hoi109_00626	hoi117_01046	hoi106_01762
809	hypothetical protein	16	Core	hoi102_00826	hoi103_00863	hoi104_00845	hoi108_01392	hoi110_01596	hoi111_01460	hoi112_00633	hoi113_00775	hoi114_00823	hoi115_01124	hoi116_01125	hoi105_00701	hoi107_00537	hoi109_00625	hoi117_01045	hoi106_01761
810	Thioredoxin	16	Core	hoi102_00827	hoi103_00864	hoi104_00846	hoi108_01393	hoi110_01597	hoi111_01459	hoi112_00634	hoi113_00776	hoi114_00824	hoi115_01125	hoi116_01126	hoi105_00700	hoi107_00536	hoi109_00624	hoi117_01044	hoi106_01760
811	1,4-alpha-glucan branching enzyme GlgB	16	Core	hoi102_00828	hoi103_00865	hoi104_00847	hoi108_01394	hoi110_01598	hoi111_01458	hoi112_00635	hoi113_00777	hoi114_00825	hoi115_01126	hoi116_01127	hoi105_00699	hoi107_00535	hoi109_00623	hoi117_01043	hoi106_01759
812	Alpha-1,4-glucan:maltose-1-phosphate maltosylt	16	Core	hoi102_00829	hoi103_00866	hoi104_00848	hoi108_01395	hoi110_01599	hoi111_01457	hoi112_00636	hoi113_00778	hoi114_00826	hoi115_01127	hoi116_01128	hoi105_00698	hoi107_00534	hoi109_00622	hoi117_01042	hoi106_01758
813	putative ABC transporter ATP-binding protein Ylr	16	Core	hoi102_00830	hoi103_00867	hoi104_00849	hoi108_01396	hoi110_01600	hoi111_01456	hoi112_00637	hoi113_00779	hoi114_00827	hoi115_01128	hoi116_01129	hoi105_00697	hoi107_00533	hoi109_00621	hoi117_01041	hoi106_01757
814	NUDIX domain protein	16	Core	hoi102_00831	hoi103_00868	hoi104_00850	hoi108_01397	hoi110_01601	hoi111_01455	hoi112_00638	hoi113_00780	hoi114_00828	hoi115_01129	hoi116_01130	hoi105_00696	hoi107_00532	hoi109_00620	hoi117_01040	hoi106_01756
815	hypothetical protein	16	Core	hoi102_00832	hoi103_00869	hoi104_00851	hoi108_01398	hoi110_01602	hoi111_01454	hoi112_00639	hoi113_00781	hoi114_00829	hoi115_01130	hoi116_01131	hoi105_00695	hoi107_00531	hoi109_00619	hoi117_01039	hoi106_01755
816	Electron transfer flavoprotein subunit beta	16	Core	hoi102_00833	hoi103_00870	hoi104_00852	hoi108_01399	hoi110_01603	hoi111_01453	hoi112_00640	hoi113_00782	hoi114_00830	hoi115_01131	hoi116_01132	hoi105_00694	hoi107_00530	hoi109_00618	hoi117_01038	hoi106_01754
817	Electron transfer flavoprotein subunit alpha	16	Core	hoi102_00834	hoi103_00871	hoi104_00853	hoi108_01400	hoi110_01604	hoi111_01452	hoi112_00641	hoi113_00783	hoi114_00831	hoi115_01132	hoi116_01133	hoi105_00693	hoi107_00529	hoi109_00617	hoi117_01037	hoi106_01753
818	Cysteine desulfurase	16	Core	hoi102_00835	hoi103_00872	hoi104_00854	hoi108_01401	hoi110_01605	hoi111_01451	hoi112_00642	hoi113_00784	hoi114_00832	hoi115_01133	hoi116_01134	hoi105_00692	hoi107_00528	hoi109_00616	hoi117_01036	hoi106_02114
819	Putative metal chaperone YciC	16	Core	hoi102_00836	hoi103_00873	hoi104_00855	hoi108_01402	hoi110_01606	hoi111_01450	hoi112_00643	hoi113_00785	hoi114_00833	hoi115_01134	hoi116_01135	hoi105_00691	hoi107_00527	hoi109_00615	hoi117_01035	hoi106_02115
820	spermidine synthase	16	Core	hoi102_00837	hoi103_00874	hoi104_00856	hoi108_01403	hoi110_01607	hoi111_01449	hoi112_00644	hoi113_00786	hoi114_00834	hoi115_01135	hoi116_01136	hoi105_00690	hoi107_00526	hoi109_00614	hoi117_01034	hoi106_02116
821	Aminodeoxychorismate synthase component 1	16	Core	hoi102_00838	hoi103_00875	hoi104_00857	hoi108_01404	hoi110_01608	hoi111_01448	hoi112_00645	hoi113_00787	hoi114_00835	hoi115_01136	hoi116_01137	hoi105_00689	hoi107_00525	hoi109_00613	hoi117_01033	hoi106_02117
822	hypothetical protein	16	Core	hoi102_00839	hoi103_00876	hoi104_00858	hoi108_01405	hoi110_01609	hoi111_01447	hoi112_00646	hoi113_00788	hoi114_00836	hoi115_01137	hoi116_01138	hoi105_00688	hoi107_00524	hoi109_00612	hoi117_01032	hoi106_02118
823	tRNA-specific 2-thiouridylase MnmA	16	Core	hoi102_00840	hoi103_00877	hoi104_00859	hoi108_01406	hoi110_01610	hoi111_01446	hoi112_00647	hoi113_00789	hoi114_00837	hoi115_01138	hoi116_01139	hoi105_00687	hoi107_00523	hoi109_00611	hoi117_01031	hoi106_02119
824	hypothetical protein	16	Core	hoi102_00841	hoi103_00878	hoi104_00860	hoi108_01407	hoi110_01611	hoi111_01445	hoi112_00648	hoi113_00790	hoi114_00838	hoi115_01139	hoi116_01140	hoi105_00686	hoi107_00522	hoi109_00610	hoi117_01030	hoi106_02120
825	hypothetical protein	16	Core	hoi102_00842	hoi103_00879	hoi104_00861	hoi108_01408	hoi110_01612	hoi111_01444	hoi112_00649	hoi113_00791	hoi114_00839	hoi115_01140	hoi116_01141	hoi105_00685	hoi107_00521	hoi109_00609	hoi117_01029	hoi106_02121
826	DNA polymerase III subunit epsilon	16	Core	hoi102_00843	hoi103_00880	hoi104_00862	hoi108_01409	hoi110_01613	hoi111_01443	hoi112_00650	hoi113_00792	hoi114_00840	hoi115_01141	hoi116_01142	hoi105_00684	hoi107_00520	hoi109_00608	hoi117_01028	hoi106_02122
827	DNA ligase	16	Core	hoi102_00844	hoi103_00881	hoi104_00863	hoi108_01410	hoi110_01614	hoi111_01442	hoi112_00651	hoi113_00793	hoi114_00841	hoi115_01142	hoi116_01143	hoi105_00683	hoi107_00519	hoi109_00607	hoi117_01027	hoi106_02123
828	hypothetical protein	16	Core	hoi102_00845	hoi103_00882	hoi104_00864	hoi108_01411	hoi110_01615	hoi111_01441	hoi112_00652	hoi113_00794	hoi114_00842	hoi115_01143	hoi116_01144	hoi105_00682	hoi107_00518	hoi109_00606	hoi117_01026	hoi106_02124
829	Glutamyl-tRNA(Gln) amidotransferase subunit C	16	Core	hoi102_00846	hoi103_00883	hoi104_00865	hoi108_01412	hoi110_01616	hoi111_01440	hoi112_00653	hoi113_00795	hoi114_00843	hoi115_01144	hoi116_01145	hoi105_00681	hoi107_02117	hoi109_00605	hoi117_02155	hoi106_02113
830	Glutamyl-tRNA(Gln) amidotransferase subunit A	16	Core	hoi102_00847	hoi103_00884	hoi104_00866	hoi108_01413	hoi110_01617	hoi111_01439	hoi112_00654	hoi113_00796	hoi114_00844	hoi115_01145	hoi116_01146	hoi105_00680	hoi107_02118	hoi109_00604	hoi117_02154	hoi106_02112
831	2',5' RNA ligase family	16	Core	hoi102_00848	hoi103_00885	hoi104_00867	hoi108_01414	hoi110_01618	hoi111_01438	hoi112_00655	hoi113_00797	hoi114_00845	hoi115_01146	hoi116_01147	hoi105_00679	hoi107_02119	hoi109_00603	hoi117_02153	hoi106_02111
832	hypothetical protein	16	Core	hoi102_00849	hoi103_00886	hoi104_00868	hoi108_01415	hoi110_01619	hoi111_01437	hoi112_00656	hoi113_00798	hoi114_00846	hoi115_01147	hoi116_01148	hoi105_00678	hoi107_02120	hoi109_00602	hoi117_02152	hoi106_02110
833	Multidrug resistance protein stp	16	Core	hoi102_00850	hoi103_00887	hoi104_00869	hoi108_01416	hoi110_01620	hoi111_01436	hoi112_00657	hoi113_00799	hoi114_00847	hoi115_01148	hoi116_01149	hoi105_00677	hoi107_02121	hoi109_00601	hoi117_02151	hoi106_02109
834	6-phosphofructokinase	16	Core	hoi102_00851	hoi103_00888	hoi104_00870	hoi108_01417	hoi110_01621	hoi111_01435	hoi112_00658	hoi113_00800	hoi114_00848	hoi115_01149	hoi116_01150	hoi105_00676	hoi107_02122	hoi109_00600	hoi117_02150	hoi106_02108
835	Aspartyl/glutamyl-tRNA(Asn/Gln) amidotransfera	16	Core	hoi102_00852	hoi103_00889	hoi104_00871	hoi108_01418	hoi110_01622	hoi111_01434	hoi112_00659	hoi113_00801	hoi114_00849	hoi115_01150	hoi116_01151	hoi105_00675	hoi107_02123	hoi109_00599	hoi117_02149	hoi106_02107
836	HNH endonuclease	16	Core	hoi102_00853	hoi103_00890	hoi104_00872	hoi108_01419	hoi110_01623	hoi111_01433	hoi112_00660	hoi113_00802	hoi114_00851	hoi115_01151	hoi116_01152	hoi105_00674	hoi107_02124	hoi109_00598	hoi117_02148	hoi106_02106
837	Succinyl-diaminopimelate desuccinylase	16	Core	hoi102_00854	hoi103_00891	hoi104_00873	hoi108_01420	hoi110_01624	hoi111_01432	hoi112_00661	hoi113_00803	hoi114_00852	hoi115_01152	hoi116_01153	hoi105_00673	hoi107_02125	hoi109_00597	hoi117_02147	hoi106_02105
838	L-glyceraldehyde 3-phosphate reductase	16	Core	hoi102_00855	hoi103_00892	hoi104_00874	hoi108_01421	hoi110_01625	hoi111_01431	hoi112_00662	hoi113_00804	hoi114_00853	hoi115_01153	hoi116_01154	hoi105_00672	hoi107_02126	hoi109_00596	hoi117_02146	hoi106_02104
839	Arginine exporter protein ArgO	16	Core	hoi102_00856	hoi103_00893	hoi104_00875	hoi108_01422	hoi110_01626	hoi111_01430	hoi112_00663	hoi113_00805	hoi114_00854	hoi115_01154	hoi116_01155	hoi105_00671	hoi107_02127	hoi109_00595	hoi117_02145	hoi106_02103
840	putative HTH-type transcriptional regulator/MT2	16	Core	hoi102_00857	hoi103_00894	hoi104_00876	hoi108_01423	hoi110_01627	hoi111_01429	hoi112_00664	hoi113_00806	hoi114_00855	hoi115_01155	hoi116_01156	hoi105_00670	hoi107_02128	hoi109_00594	hoi117_02144	hoi106_02102
841	DoxX	16	Core	hoi102_00858	hoi103_00895	hoi104_00877	hoi108_01424	hoi110_01628	hoi111_01428	hoi112_00665	hoi113_00807	hoi114_00856	hoi115_01156	hoi116_01157	hoi105_00669	hoi107_02129	hoi109_00593	hoi117_02143	hoi106_02101
842	Dihydroxy-acid dehydratase	16	Core	hoi102_00859	hoi103_00896	hoi104_00878	hoi108_01425	hoi110_01629	hoi111_01427	hoi112_00666	hoi113_00808	hoi114_00857	hoi115_01157	hoi116_01158	hoi105_00668	hoi107_02130	hoi109_00592	hoi117_02142	hoi106_02100
843	Low molecular weight protein antigen 6	16	Core	hoi102_00860	hoi103_00897	hoi104_00879	hoi108_01426	hoi110_01630	hoi111_01426	hoi112_00667	hoi113_00809	hoi114_00858	hoi115_01158	hoi116_01159	hoi105_00667	hoi107_02131	hoi109_00591	hoi117_02141	hoi106_02099
844	putative MscS family protein YkuT	8	Accessory	hoi102_00861	hoi103_00898	hoi104_00880	hoi108_01427			hoi112_00668	hoi113_00810	hoi114_00859	hoi115_01159						
845	Acetolactate synthase large subunit	16	Core	hoi102_00862	hoi103_00899	hoi104_00881	hoi108_01428	hoi110_01953	hoi111_01772	hoi112_00669	hoi113_00811	hoi114_00860	hoi115_01160	hoi116_01934	hoi105_01910	hoi107_01875	hoi109_01886	hoi117_02067	hoi106_01672
846	Acetolactate synthase small subunit	16	Core	hoi102_00863	hoi103_00900	hoi104_00882	hoi108_01429	hoi110_01952	hoi111_01773	hoi112_00670	hoi113_00812	hoi114_00861	hoi115_01161	hoi116_01935	hoi105_01911	hoi107_01876	hoi109_01887	hoi117_02068	hoi106_01671
847	Ketol-acid reductoisomerase	16	Core	hoi102_00864	hoi103_00901	hoi104_00883	hoi108_01430	hoi110_01951	hoi111_01774	hoi112_00671	hoi113_00813	hoi114_00862	hoi115_01162	hoi116_01936	hoi105_01912	hoi107_01877	hoi109_01888	hoi117_02069	hoi106_01670
848	Cadmium, cobalt and zinc/H(+)-K(+) antiporter	16	Core	hoi102_00865	hoi103_00902	hoi104_00884	hoi108_01431	hoi110_01950	hoi111_01775	hoi112_00672	hoi113_00814	hoi114_00863	hoi115_01163	hoi116_01937	hoi105_01913	hoi107_01878	hoi109_01889	hoi117_02070	hoi106_01669
849	hypothetical protein	16	Core	hoi102_00866	hoi103_00903	hoi104_00885	hoi108_01432	hoi110_01949	hoi111_01776	hoi112_00673	hoi113_00815	hoi114_00864	hoi115_01164	hoi116_01938	hoi105_01914	hoi107_01879	hoi109_01890	hoi117_02071	hoi106_01668
850	hypothetical protein	16	Core	hoi102_00867	hoi103_00904	hoi104_00886	hoi108_01433	hoi110_01948	hoi111_01777	hoi112_00674	hoi113_00816	hoi114_00865	hoi115_01165	hoi116_01939	hoi105_01915	hoi107_01880	hoi109_01891	hoi117_02072	hoi106_01667
851	D-3-phosphoglycerate dehydrogenase	16	Core	hoi102_00868	hoi103_00905	hoi104_00887	hoi108_01434	hoi110_01947	hoi111_01778	hoi112_00675	hoi113_00817	hoi114_00866	hoi115_01166	hoi116_01940	hoi105_01916	hoi107_01881	hoi109_01892	hoi117_02073	hoi106_01666
852	hypothetical protein	16	Core	hoi102_00869	hoi103_00906	hoi104_00888	hoi108_01435	hoi110_01946	hoi111_01779	hoi112_00676	hoi113_00818	hoi114_00867	hoi115_01167	hoi116_01941	hoi105_01917	hoi107_01882	hoi109_01893	hoi117_02074	hoi106_01665
853	3-isopropylmalate dehydrogenase	16	Core	hoi102_00870	hoi103_00907	hoi104_00889	hoi108_01436	hoi110_01945	hoi111_01780	hoi112_00677	hoi113_00819	hoi114_00868	hoi115_01168	hoi116_01942	hoi105_01918	hoi107_01883	hoi109_01894	hoi117_02075	hoi106_01664
854	Arabinose 5-phosphate isomerase KdsD	16	Core	hoi102_00871	hoi103_00908	hoi104_00890	hoi108_01437	hoi110_01944	hoi111_01781	hoi112_00678	hoi113_00820	hoi114_00869	hoi115_01169	hoi116_01943	hoi105_01919	hoi107_01884	hoi109_01895	hoi117_02076	hoi106_01663
855	DNA polymerase III PolC-type	16	Core	hoi102_00872	hoi103_00909	hoi104_00891	hoi108_01438	hoi110_01943	hoi111_01782	hoi112_00679	hoi113_00821	hoi114_00870	hoi115_01170	hoi116_01944	hoi105_01920	hoi107_01885	hoi109_01896	hoi117_02077	hoi106_01662
856	Ureidoglycolate lyase	16	Core	hoi102_00873	hoi103_00910	hoi104_00892	hoi108_01439	hoi110_01942	hoi111_01783	hoi112_00680	hoi113_00822	hoi114_00871	hoi115_01171	hoi116_01945	hoi105_01921	hoi107_01886	hoi109_01897	hoi117_02078	hoi106_01661
857	Isochorismate synthase DhbC	16	Core	hoi102_00874	hoi103_00911	hoi104_00893	hoi108_01440	hoi110_01941	hoi111_01784	hoi112_00681	hoi113_00823	hoi114_00872	hoi115_01172	hoi116_01946	hoi105_01922	hoi107_01887	hoi109_01898	hoi117_02079	hoi106_01660
858	Glutamate--tRNA ligase	16	Core	hoi102_00875	hoi103_00912	hoi104_00894	hoi108_01441	hoi110_01940	hoi111_01785	hoi112_00682	hoi113_00824	hoi114_00873	hoi115_01173	hoi116_01947	hoi105_01923	hoi107_01888	hoi109_01899	hoi117_02080	hoi106_01659
859	Putative niacin/nicotinamide transporter NaiP	16	Core	hoi102_00878	hoi103_00915	hoi104_00897	hoi108_01444	hoi110_01937	hoi111_01788	hoi112_00685	hoi113_00827	hoi114_00876	hoi115_01176	hoi116_01950	hoi105_01926	hoi107_01891	hoi109_01902	hoi117_02083	hoi106_01656
860	Rieske [2Fe-2S] domain protein	16	Core	hoi102_00879	hoi103_00916	hoi104_00898	hoi108_01445	hoi110_01936	hoi111_01789	hoi112_00686	hoi113_00828	hoi114_00877	hoi115_01177	hoi116_01951	hoi105_01927	hoi107_01892	hoi109_01903	hoi117_02084	hoi106_01655
861	HTH-type transcriptional repressor AllR	16	Core	hoi102_00881	hoi103_00918	hoi104_00900	hoi108_01447	hoi110_01934	hoi111_01791	hoi112_00688	hoi113_00830	hoi114_00879	hoi115_01179	hoi116_01953	hoi105_01929	hoi107_01894	hoi109_01905	hoi117_02086	hoi106_01652
862	3-isopropylmalate dehydratase large subunit	16	Core	hoi102_00882	hoi103_00919	hoi104_00901	hoi108_01448	hoi110_01933	hoi111_01792	hoi112_00689	hoi113_00831	hoi114_00880	hoi115_01180	hoi116_01954	hoi105_01930	hoi107_01895	hoi109_01906	hoi117_02087	hoi106_01651
863	3-isopropylmalate dehydratase small subunit	16	Core	hoi102_00883	hoi103_00920	hoi104_00902	hoi108_01449	hoi110_01932	hoi111_01793	hoi112_00690	hoi113_00832	hoi114_00881	hoi115_01181	hoi116_01955	hoi105_01931	hoi107_01896	hoi109_01907	hoi117_02088	hoi106_01650
864	Putative mutator protein MutT4	16	Core	hoi102_00884	hoi103_00921	hoi104_00903	hoi108_01450	hoi110_01931	hoi111_01794	hoi112_00691	hoi113_00833	hoi114_00882	hoi115_01182	hoi116_01956	hoi105_01932	hoi107_01897	hoi109_01908	hoi117_02089	hoi106_01649
865	hypothetical protein	16	Core	hoi102_00885	hoi103_00922	hoi104_00904	hoi108_01451	hoi110_01930	hoi111_01795	hoi112_00692	hoi113_00834	hoi114_00883	hoi115_01183	hoi116_01957	hoi105_01933	hoi107_01898	hoi109_01909	hoi117_02090	hoi106_01648
866	Glycerol-3-phosphate dehydrogenase [NAD(P)+]	16	Core	hoi102_00886	hoi103_00923	hoi104_00905	hoi108_01452	hoi110_01929	hoi111_01796	hoi112_00693	hoi113_00835	hoi114_00884	hoi115_01184	hoi116_01958	hoi105_01934	hoi107_01899	hoi109_01910	hoi117_02091	hoi106_01647
867	D-alanine--D-alanine ligase	16	Core	hoi102_00887	hoi103_00924	hoi104_00906	hoi108_01453	hoi110_01928	hoi111_01797	hoi112_00694	hoi113_00836	hoi114_00885	hoi115_01185	hoi116_01959	hoi105_01935	hoi107_01900	hoi109_01911	hoi117_02092	hoi106_01646
868	putative ABC transporter permease protein	16	Core	hoi102_00888	hoi103_00925	hoi104_00907	hoi108_01454	hoi110_01927	hoi111_01798	hoi112_00695	hoi113_00837	hoi114_00886	hoi115_01186	hoi116_01960	hoi105_01936	hoi107_01901	hoi109_01912	hoi117_02093	hoi106_01645
869	putative siderophore transport system ATP-bindi	16	Core	hoi102_00889	hoi103_00926	hoi104_00908	hoi108_01455	hoi110_01926	hoi111_01799	hoi112_00696	hoi113_00838	hoi114_00887	hoi115_01187	hoi116_01961	hoi105_01937	hoi107_01902	hoi109_01913	hoi117_02094	hoi106_01644
870	Periplasmic binding protein	16	Core	hoi102_00890	hoi103_00927	hoi104_00909	hoi108_01456	hoi110_01925	hoi111_01800	hoi112_00697	hoi113_00839	hoi114_00888	hoi115_01188	hoi116_01962	hoi105_01938	hoi107_01903	hoi109_01914	hoi117_02095	hoi106_01643
871	hypothetical protein	16	Core	hoi102_00891	hoi103_00928	hoi104_00910	hoi108_01457	hoi110_01924	hoi111_01801	hoi112_00698	hoi113_00840	hoi114_00889	hoi115_01189	hoi116_01963	hoi105_01939	hoi107_01904	hoi109_01915	hoi117_02096	hoi106_01642
872	Thiamine-monophosphate kinase	16	Core	hoi102_00892	hoi103_00929	hoi104_00911	hoi108_01458	hoi110_01923	hoi111_01802	hoi112_00699	hoi113_00841	hoi114_00890	hoi115_01190	hoi116_01964	hoi105_01940	hoi107_01905	hoi109_01916	hoi117_02097	hoi106_01641
873	Uracil-DNA glycosylase	16	Core	hoi102_00893	hoi103_00930	hoi104_00912	hoi108_01459	hoi110_01922	hoi111_01803	hoi112_00700	hoi113_00842	hoi114_00891	hoi115_01191	hoi116_01965	hoi105_01941	hoi107_01906	hoi109_01917	hoi117_02098	hoi106_01640
874	DAK2 domain protein	16	Core	hoi102_00894	hoi103_00931	hoi104_00913	hoi108_01460	hoi110_01921	hoi111_01804	hoi112_00701	hoi113_00843	hoi114_00892	hoi115_01192	hoi116_01966	hoi105_01942	hoi107_01907	hoi109_01918	hoi117_02099	hoi106_01639
875	ATP-dependent DNA helicase RecG	16	Core	hoi102_00895	hoi103_00932	hoi104_00914	hoi108_01461	hoi110_01920	hoi111_01805	hoi112_00702	hoi113_00844	hoi114_00893	hoi115_01193	hoi116_01967	hoi105_01943	hoi107_01908	hoi109_01919	hoi117_02100	hoi106_01638
876	pyruvate carboxylase	16	Core	hoi102_00896	hoi103_00933	hoi104_00915	hoi108_01462	hoi110_01919	hoi111_01806	hoi112_00703	hoi113_00845	hoi114_00894	hoi115_01194	hoi116_01968	hoi105_01944	hoi107_01909	hoi109_01920	hoi117_02101	hoi106_01637
877	Ribosomal RNA small subunit methyltransferase	16	Core	hoi102_00897	hoi103_00934	hoi104_00916	hoi108_01463	hoi110_01918	hoi111_01807	hoi112_00704	hoi113_00846	hoi114_00895	hoi115_01195	hoi116_01969	hoi105_01945	hoi107_01910	hoi109_01921	hoi117_02102	hoi106_01636

878	Phosphopantetheine adenylyltransferase	16	Core	hoi102_00898	hoi103_00935	hoi104_00917	hoi108_01464	hoi110_01917	hoi111_01808	hoi112_00705	hoi113_00847	hoi114_00896	hoi115_01196	hoi116_01970	hoi105_01946	hoi107_01911	hoi109_01922	hoi117_02103	hoi106_01635
879	Sulfite exporter TauE/SafE	16	Core	hoi102_00899	hoi103_00936	hoi104_00918	hoi108_01465	hoi110_01916	hoi111_01809	hoi112_00706	hoi113_00848	hoi114_00897	hoi115_01197	hoi116_01971	hoi105_01947	hoi107_01912	hoi109_01923	hoi117_02104	hoi106_01634
880	L-cystine import ATP-binding protein TcyC	16	Core	hoi102_00900	hoi103_00937	hoi104_00919	hoi108_01466	hoi110_01915	hoi111_01810	hoi112_00707	hoi113_00849	hoi114_00898	hoi115_01198	hoi116_01972	hoi105_01948	hoi107_01913	hoi109_01924	hoi117_02105	hoi106_01633
881	Inner membrane amino-acid ABC transporter pe	16	Core	hoi102_00901	hoi103_00938	hoi104_00920	hoi108_01467	hoi110_01914	hoi111_01811	hoi112_00708	hoi113_00850	hoi114_00899	hoi115_01199	hoi116_01973	hoi105_01949	hoi107_01914	hoi109_01925	hoi117_02106	hoi106_01632
882	Glutamine-binding periplasmic protein precursor	16	Core	hoi102_00902	hoi103_00939	hoi104_00921	hoi108_01468	hoi110_01913	hoi111_01812	hoi112_00709	hoi113_00851	hoi114_00900	hoi115_01200	hoi116_01974	hoi105_01950	hoi107_01915	hoi109_01926	hoi117_02107	hoi106_01631
883	hypothetical protein	16	Core	hoi102_00903	hoi103_00940	hoi104_00922	hoi108_01469	hoi110_01912	hoi111_01813	hoi112_00710	hoi113_00852	hoi114_00901	hoi115_01201	hoi116_01975	hoi105_01951	hoi107_01916	hoi109_01927	hoi117_02108	hoi106_01630
884	DNA polymerase I	16	Core	hoi102_00905	hoi103_00942	hoi104_00924	hoi108_01471	hoi110_01910	hoi111_01815	hoi112_00712	hoi113_00854	hoi114_00903	hoi115_01203	hoi116_01977	hoi105_01953	hoi107_01918	hoi109_01929	hoi117_02110	hoi106_01628
885	Membrane transport protein	16	Core	hoi102_00906	hoi103_00943	hoi104_00925	hoi108_01472	hoi110_01909	hoi111_01816	hoi112_00713	hoi113_00855	hoi114_00904	hoi115_01204	hoi116_01978	hoi105_01954	hoi107_01919	hoi109_01930	hoi117_02111	hoi106_01627
886	Malonyl-[acyl-carrier protein] O-methyltransfera	16	Core	hoi102_00907	hoi103_00944	hoi104_00926	hoi108_01473	hoi110_01908	hoi111_01817	hoi112_00714	hoi113_00856	hoi114_00905	hoi115_01205	hoi116_01979	hoi105_01955	hoi107_01920	hoi109_01931	hoi117_02112	hoi106_01626
887	30S ribosomal protein S1	16	Core	hoi102_00908	hoi103_00945	hoi104_00927	hoi108_01474	hoi110_01907	hoi111_01818	hoi112_00715	hoi113_00857	hoi114_00906	hoi115_01206	hoi116_01980	hoi105_01956	hoi107_01921	hoi109_01932	hoi117_02113	hoi106_01625
888	PTS system beta-glucoside-specific EIIBCA compo	16	Core	hoi102_00909	hoi103_00946	hoi104_00928	hoi108_01475	hoi110_01906	hoi111_01819	hoi112_00716	hoi113_00858	hoi114_00907	hoi115_01207	hoi116_01981	hoi105_01957	hoi107_01922	hoi109_01933	hoi117_02114	hoi106_01624
889	L-lactate dehydrogenase	16	Core	hoi102_00910	hoi103_00947	hoi104_00929	hoi108_01476	hoi110_01905	hoi111_01820	hoi112_00717	hoi113_00859	hoi114_00908	hoi115_01208	hoi116_01982	hoi105_01958	hoi107_01923	hoi109_01934	hoi117_02115	hoi106_01623
890	Dephospho-CoA kinase	16	Core	hoi102_00911	hoi103_00948	hoi104_00930	hoi108_01477	hoi110_01904	hoi111_01821	hoi112_00718	hoi113_00860	hoi114_00909	hoi115_01209	hoi116_01983	hoi105_01959	hoi107_01924	hoi109_01935	hoi117_02116	hoi106_01622
891	UvrABC system protein B	16	Core	hoi102_00912	hoi103_00949	hoi104_00931	hoi108_01478	hoi110_01903	hoi111_01822	hoi112_00719	hoi113_00861	hoi114_00910	hoi115_01210	hoi116_01984	hoi105_01960	hoi107_01925	hoi109_01936	hoi117_02117	hoi106_01621
892	Universal stress protein/MT1672	16	Core	hoi102_00913	hoi103_00950	hoi104_00932	hoi108_01479	hoi110_01902	hoi111_01823	hoi112_00720	hoi113_00862	hoi114_00911	hoi115_01211	hoi116_01985	hoi105_01961	hoi107_01926	hoi109_01937	hoi117_02118	hoi106_01620
893	Helicase IV	16	Core	hoi102_00914	hoi103_00951	hoi104_00933	hoi108_01480	hoi110_01901	hoi111_01824	hoi112_00721	hoi113_00863	hoi114_00912	hoi115_01212	hoi116_01986	hoi105_01962	hoi107_01927	hoi109_01938	hoi117_02119	hoi106_01619
894	DoxX	16	Core	hoi102_00915	hoi103_00952	hoi104_00934	hoi108_01481	hoi110_01900	hoi111_01825	hoi112_00722	hoi113_00864	hoi114_00913	hoi115_01213	hoi116_01987	hoi105_01963	hoi107_01928	hoi109_01939	hoi117_02120	hoi106_01618
895	putative polyketide biosynthesis zinc-dependent	16	Core	hoi102_00916	hoi103_00953	hoi104_00935	hoi108_01482	hoi110_01899	hoi111_01826	hoi112_00723	hoi113_00865	hoi114_00914	hoi115_01214	hoi116_01988	hoi105_01964	hoi107_01929	hoi109_01940	hoi117_02121	hoi106_01617
896	UvrABC system protein A	16	Core	hoi102_00917	hoi103_00954	hoi104_00936	hoi108_01483	hoi110_01898	hoi111_01827	hoi112_00724	hoi113_00866	hoi114_00915	hoi115_01215	hoi116_01989	hoi105_01965	hoi107_01930	hoi109_01941	hoi117_02122	hoi106_01616
897	hypothetical protein	16	Core	hoi102_00918	hoi103_00955	hoi104_00937	hoi108_01484	hoi110_01897	hoi111_01828	hoi112_00725	hoi113_00867	hoi114_00916	hoi115_01216	hoi116_01990	hoi105_01966	hoi107_01931	hoi109_01942	hoi117_02123	hoi106_01615
898	Translation initiation factor IF-3	16	Core	hoi102_00919	hoi103_00956	hoi104_00938	hoi108_01485	hoi110_01896	hoi111_01829	hoi112_00726	hoi113_00868	hoi114_00917	hoi115_01217	hoi116_01991	hoi105_01967	hoi107_01932	hoi109_01943	hoi117_02124	hoi106_01614
899	50S ribosomal protein L35	16	Core	hoi102_00920	hoi103_00957	hoi104_00939	hoi108_01486	hoi110_01895	hoi111_01830	hoi112_00727	hoi113_00869	hoi114_00918	hoi115_01218	hoi116_01992	hoi105_01968	hoi107_01933	hoi109_01944	hoi117_02125	hoi106_01613
900	50S ribosomal protein L20	16	Core	hoi102_00921	hoi103_00958	hoi104_00940	hoi108_01487	hoi110_01894	hoi111_01831	hoi112_00728	hoi113_00870	hoi114_00919	hoi115_01219	hoi116_01993	hoi105_01969	hoi107_01934	hoi109_01945	hoi117_02126	hoi106_01612
901	TM2 domain protein	16	Core	hoi102_00922	hoi103_00959	hoi104_00941	hoi108_01488	hoi110_01893	hoi111_01832	hoi112_00729	hoi113_00871	hoi114_00920	hoi115_01220	hoi116_01994	hoi105_01970	hoi107_01935	hoi109_01946	hoi117_02127	hoi106_01611
902	23S rRNA (uridine(2479)-2'-O)-methyltransferase	16	Core	hoi102_00923	hoi103_00960	hoi104_00942	hoi108_01489	hoi110_01892	hoi111_01833	hoi112_00730	hoi113_00872	hoi114_00921	hoi115_01221	hoi116_01995	hoi105_01971	hoi107_01936	hoi109_01947	hoi117_02128	hoi106_01610
903	Phenylalanine--tRNA ligase alpha subunit	16	Core	hoi102_00924	hoi103_00961	hoi104_00943	hoi108_01490	hoi110_01891	hoi111_01834	hoi112_00731	hoi113_00873	hoi114_00922	hoi115_01222	hoi116_01996	hoi105_01972	hoi107_01937	hoi109_01948	hoi117_02129	hoi106_01609
904	Phenylalanine--tRNA ligase beta subunit	16	Core	hoi102_00925	hoi103_00962	hoi104_00944	hoi108_01491	hoi110_01890	hoi111_01835	hoi112_00732	hoi113_00874	hoi114_00923	hoi115_01223	hoi116_01997	hoi105_01973	hoi107_01938	hoi109_01949	hoi117_02130	hoi106_01608
905	N-acetyl-gamma-glutamyl-phosphate reductase	16	Core	hoi102_00926	hoi103_00963	hoi104_00945	hoi108_01492	hoi110_01889	hoi111_01836	hoi112_00733	hoi113_00875	hoi114_00924	hoi115_01224	hoi116_01998	hoi105_01974	hoi107_01939	hoi109_01950	hoi117_02131	hoi106_01607
906	Arginine biosynthesis bifunctional protein ArgJ	16	Core	hoi102_00927	hoi103_00964	hoi104_00946	hoi108_01493	hoi110_01888	hoi111_01837	hoi112_00734	hoi113_00876	hoi114_00925	hoi115_01225	hoi116_01999	hoi105_01975	hoi107_01940	hoi109_01951	hoi117_02132	hoi106_01606
907	Acetylglutamate kinase	16	Core	hoi102_00928	hoi103_00965	hoi104_00947	hoi108_01494	hoi110_01887	hoi111_01838	hoi112_00735	hoi113_00877	hoi114_00926	hoi115_01226	hoi116_02000	hoi105_01976	hoi107_01941	hoi109_01952	hoi117_02133	hoi106_01605
908	Acetylornithine aminotransferase	16	Core	hoi102_00929	hoi103_00966	hoi104_00948	hoi108_01495	hoi110_01886	hoi111_01839	hoi112_00736	hoi113_00878	hoi114_00927	hoi115_01227	hoi116_02001	hoi105_01977	hoi107_01942	hoi109_01953	hoi117_02134	hoi106_01604
909	Ornithine carbamoyltransferase	16	Core	hoi102_00930	hoi103_00967	hoi104_00949	hoi108_01496	hoi110_01885	hoi111_01840	hoi112_00737	hoi113_00879	hoi114_00928	hoi115_01228	hoi116_02002	hoi105_01978	hoi107_01943	hoi109_01954	hoi117_02135	hoi106_01603
910	Arginine repressor	16	Core	hoi102_00931	hoi103_00968	hoi104_00950	hoi108_01497	hoi110_01884	hoi111_01841	hoi112_00738	hoi113_00880	hoi114_00929	hoi115_01229	hoi116_02003	hoi105_01979	hoi107_01944	hoi109_01955	hoi117_02136	hoi106_01602
911	Argininosuccinate synthase	16	Core	hoi102_00932	hoi103_00969	hoi104_00951	hoi108_01498	hoi110_01883	hoi111_01842	hoi112_00739	hoi113_00881	hoi114_00930	hoi115_01230	hoi116_02004	hoi105_01980	hoi107_01945	hoi109_01956	hoi117_02137	hoi106_01601
912	Argininosuccinate lyase	16	Core	hoi102_00933	hoi103_00970	hoi104_00952	hoi108_01499	hoi110_01882	hoi111_01843	hoi112_00740	hoi113_00882	hoi114_00931	hoi115_01231	hoi116_02005	hoi105_01981	hoi107_01946	hoi109_01957	hoi117_02138	hoi106_01600
913	hypothetical protein	16	Core	hoi102_00934	hoi103_00971	hoi104_00953	hoi108_01500	hoi110_01881	hoi111_01844	hoi112_00741	hoi113_00883	hoi114_00932	hoi115_01232	hoi116_02006	hoi105_01982	hoi107_01947	hoi109_01958	hoi117_02139	hoi106_01599
914	Tyrosine--tRNA ligase	16	Core	hoi102_00935	hoi103_00972	hoi104_00954	hoi108_01501	hoi110_01880	hoi111_01845	hoi112_00742	hoi113_00884	hoi114_00933	hoi115_01233	hoi116_02007	hoi105_01983	hoi107_01948	hoi109_01959	hoi117_02140	hoi106_01598
915	putative transcriptional regulator WhiB3	16	Core	hoi102_00936	hoi103_01020	hoi104_00955	hoi108_00001	hoi110_00462	hoi111_00343	hoi112_01227	hoi113_01146	hoi114_00934	hoi115_01234	hoi116_00953	hoi105_00666	hoi107_01017	hoi109_01209	hoi117_00756	hoi106_00510
916	60 kDa chaperonin 1	16	Core	hoi102_00937	hoi103_01019	hoi104_00956	hoi108_00002	hoi110_00463	hoi111_00344	hoi112_01226	hoi113_01147	hoi114_00935	hoi115_01235	hoi116_00952	hoi105_00665	hoi107_01018	hoi109_01208	hoi117_00757	hoi106_00511
917	10 kDa chaperonin	16	Core	hoi102_00938	hoi103_01018	hoi104_00957	hoi108_00003	hoi110_00464	hoi111_00345	hoi112_01225	hoi113_01148	hoi114_00936	hoi115_01236	hoi116_00951	hoi105_00664	hoi107_01019	hoi109_01207	hoi117_00758	hoi106_00512
918	hypothetical protein	16	Core	hoi102_00939	hoi103_01017	hoi104_00958	hoi108_00004	hoi110_00465	hoi111_00346	hoi112_01224	hoi113_01149	hoi114_00937	hoi115_01237	hoi116_00950	hoi105_00663	hoi107_01020	hoi109_01206	hoi117_00759	hoi106_00513
919	hypothetical protein	16	Core	hoi102_00940	hoi103_01016	hoi104_00959	hoi108_00005	hoi110_00466	hoi111_00347	hoi112_01223	hoi113_01150	hoi114_00938	hoi115_01238	hoi116_00949	hoi105_00662	hoi107_01021	hoi109_01205	hoi117_00760	hoi106_00514
920	tRNA N6-adenosine threonylcarbamoyltransfera	16	Core	hoi102_00941	hoi103_01015	hoi104_00960	hoi108_00006	hoi110_00467	hoi111_00348	hoi112_01222	hoi113_01151	hoi114_00939	hoi115_01239	hoi116_00948	hoi105_00661	hoi107_01022	hoi109_01204	hoi117_00761	hoi106_00515
921	ribosomal-protein-alanine N-acetyltransferase	16	Core	hoi102_00942	hoi103_01014	hoi104_00961	hoi108_00007	hoi110_00468	hoi111_00349	hoi112_01221	hoi113_01152	hoi114_00940	hoi115_01240	hoi116_00947	hoi105_00660	hoi107_01023	hoi109_01203	hoi117_00762	hoi106_00516
922	tRNA threonylcarbamoyladenosine biosynthesis	16	Core	hoi102_00943	hoi103_01013	hoi104_00962	hoi108_00008	hoi110_00469	hoi111_00350	hoi112_01220	hoi113_01153	hoi114_00941	hoi115_01241	hoi116_00946	hoi105_00659	hoi107_01024	hoi109_01202	hoi117_00763	hoi106_00517
923	Methylated-DNA--protein-cysteine methyltransf	16	Core	hoi102_00944	hoi103_01012	hoi104_00963	hoi108_00009	hoi110_00470	hoi111_00351	hoi112_01219	hoi113_01154	hoi114_00942	hoi115_01242	hoi116_00945	hoi105_00658	hoi107_01025	hoi109_01201	hoi117_00764	hoi106_00518
924	Multidrug resistance protein stp	16	Core	hoi102_00945	hoi103_01011	hoi104_00964	hoi108_00010	hoi110_00471	hoi111_00352	hoi112_01218	hoi113_01155	hoi114_00943	hoi115_01243	hoi116_00944	hoi105_00657	hoi107_01026	hoi109_01200	hoi117_00765	hoi106_00519
925	hypothetical protein	16	Core	hoi102_00946	hoi103_01010	hoi104_00965	hoi108_00011	hoi110_00472	hoi111_00353	hoi112_01217	hoi113_01156	hoi114_00944	hoi115_01244	hoi116_00943	hoi105_00656	hoi107_01027	hoi109_01199	hoi117_00766	hoi106_00520
926	tRNA threonylcarbamoyladenosine biosynthesis	16	Core	hoi102_00947	hoi103_01009	hoi104_00966	hoi108_00012	hoi110_00473	hoi111_00354	hoi112_01216	hoi113_01157	hoi114_00945	hoi115_01245	hoi116_00942	hoi105_00655	hoi107_01028	hoi109_01198	hoi117_00767	hoi106_00521
927	Alanine racemase	16	Core	hoi102_00948	hoi103_01008	hoi104_00967	hoi108_00013	hoi110_00474	hoi111_00355	hoi112_01215	hoi113_01158	hoi114_00946	hoi115_01246	hoi116_00941	hoi105_00654	hoi107_01029	hoi109_01197	hoi117_00768	hoi106_00522
928	CAAX amino terminal protease self- immunity	16	Core	hoi102_00949	hoi103_01007	hoi104_00968	hoi108_00014	hoi110_00475	hoi111_00356	hoi112_01214	hoi113_01159	hoi114_00947	hoi115_01247	hoi116_00940	hoi105_00653	hoi107_01030	hoi109_01196	hoi117_00769	hoi106_00523
929	Glutamine--fructose-6-phosphate aminotransfer	16	Core	hoi102_00950	hoi103_01006	hoi104_00969	hoi108_00015	hoi110_00476	hoi111_00357	hoi112_01213	hoi113_01160	hoi114_00948	hoi115_01248	hoi116_00939	hoi105_00652	hoi107_01031	hoi109_01195	hoi117_00770	hoi106_00524
930	Alpha/beta hydrolase family protein	16	Core	hoi102_00951	hoi103_01005	hoi104_00970	hoi108_00016	hoi110_00477	hoi111_00358	hoi112_01212	hoi113_01161	hoi114_00949	hoi115_01249	hoi116_00938	hoi105_00651	hoi107_01032	hoi109_01194	hoi117_00771	hoi106_00525
931	hypothetical protein	9	Accessory	hoi102_00952	hoi103_01004	hoi104_00971	hoi108_00017		hoi111_00359	hoi112_01211	hoi113_01162		hoi115_01250	hoi116_00937					
932	hypothetical protein	16	Core	hoi102_00953	hoi103_01003	hoi104_00972	hoi108_00018	hoi110_00478	hoi111_00360	hoi112_01210	hoi113_01163	hoi114_00950	hoi115_01251	hoi116_00936	hoi105_00650	hoi107_01033	hoi109_01193	hoi117_00772	hoi106_00526
933	hypothetical protein	16	Core	hoi102_00954	hoi103_01002	hoi104_00973	hoi108_00019	hoi110_00479	hoi111_00361	hoi112_01209	hoi113_01164	hoi114_00951	hoi115_01252	hoi116_00935	hoi105_00649	hoi107_01034	hoi109_01192	hoi117_00773	hoi106_00527
934	Phosphoglucosamine mutase	16	Core	hoi102_00955	hoi103_01001	hoi104_00974	hoi108_00020	hoi110_00480	hoi111_00362	hoi112_01208	hoi113_01165	hoi114_00952	hoi115_01253	hoi116_00934	hoi105_00648	hoi107_01035	hoi109_01191	hoi117_00774	hoi106_00528
935	LOG family protein YvdD	16	Core	hoi102_00956	hoi103_01000	hoi104_00975	hoi108_00021	hoi110_00481	hoi111_00363	hoi112_01207	hoi113_01166	hoi114_00953	hoi115_01254	hoi116_00933	hoi105_00647	hoi107_01036	hoi109_01190	hoi117_00775	hoi106_00529
936	30S ribosomal protein S9	16	Core	hoi102_00957	hoi103_00999	hoi104_00976	hoi108_00022	hoi110_00482	hoi111_00364	hoi112_01206	hoi113_01167	hoi114_00954	hoi115_01255	hoi116_00932	hoi105_00646	hoi107_01037	hoi109_01189	hoi117_00776	hoi106_00530
937	50S ribosomal protein L13	16	Core	hoi102_00958	hoi103_00998	hoi104_00977	hoi108_00023	hoi110_00483	hoi111_00365	hoi112_01205	hoi113_01168	hoi114_00955	hoi115_01256	hoi116_00931	hoi105_00645	hoi107_01038	hoi109_01188	hoi117_00777	hoi106_00531
938	putative sugar epimerase YhfK	15	Accessory	hoi102_00959	hoi103_00997	hoi104_00978	hoi108_00024	hoi110_00484	hoi111_00366	hoi112_01204	hoi113_01169	hoi114_00956	hoi115_01257	hoi116_00930	hoi105_00644	hoi107_01039	hoi109_01187	hoi117_00778	
939	6 kDa early secretory antigenic target	16	Core	hoi102_00960	hoi103_00996	hoi104_00979	hoi108_00025	hoi110_00485	hoi111_00367	hoi112_01203	hoi113_01170	hoi114_00957	hoi115_01258	hoi116_00929	hoi105_00643	hoi107_01040	hoi109_01186	hoi117_00779	hoi106_00532
940	Proteins of 100 residues with WXG	16	Core	hoi102_00961	hoi103_00995	hoi104_00980	hoi108_00026	hoi110_00486	hoi111_00368	hoi112_01202	hoi113_01171	hoi114_00958	hoi115_01259	hoi116_00928	hoi105_00642	hoi107_01041	hoi109_01185	hoi117_00780	hoi106_00533
941	hypothetical protein	16	Core	hoi102_00962	hoi103_00994	hoi104_00981	hoi108_00027	hoi110_00487	hoi111_00369	hoi112_01201	hoi113_01172	hoi114_00959	hoi115_01260	hoi116_00927	hoi105_00641	hoi107_01042	hoi109_01184	hoi117_00781	hoi106_00534
942	ESX-1 secretion system protein EccCa1	16	Core	hoi102_00963	hoi103_00993	hoi104_00982	hoi108_00028	hoi110_00488	hoi111_00370	hoi112_01200	hoi113_01173	hoi114_00960	hoi115_01261	hoi116_00926	hoi105_00640	hoi107_01043	hoi109_01183	hoi117_00782	hoi106_00535
943	hypothetical protein	16	Core	hoi102_00964	hoi103_00992	hoi104_00983	hoi108_00029	hoi110_00489	hoi111_00371	hoi112_01199	hoi113_01174	hoi114_00961	hoi115_01262	hoi116_00925	hoi105_00639	hoi107_01044	hoi109_01182	hoi117_00783	hoi106_00536
944	Subtilisin	16	Core	hoi102_00965	hoi103_00991	hoi104_00984	hoi108_00030	hoi110_00490	hoi111_00372	hoi112_01198	hoi113_01175	hoi114_00962	hoi115_01263	hoi116_00924	hoi105_00638	hoi107_01045	hoi109_01181	hoi117_00784	hoi106_00537
945	ESX-1 secretion system protein eccB1	16	Core	hoi102_00966	hoi103_00990	hoi104_00985	hoi108_00031	hoi110_00491	hoi111_00373	hoi112_01197	hoi113_01176	hoi114_00963	hoi115_01264	hoi116_00923	hoi105_00637	hoi107_01046	hoi109_01180	hoi117_00785	hoi106_00538
946	hypothetical protein	16	Core	hoi102_00967	hoi103_00989	hoi104_00986	hoi108_00032	hoi110_00492	hoi111_00374	hoi112_01196	hoi113_01177	hoi114_00964	hoi115_01265	hoi116_00922	hoi105_00636	hoi107_01047	hoi109_01179	hoi117_00786	hoi106_00539
947	Iron-dependent repressor IdeR	16	Core	hoi102_00968	hoi103_00988	hoi104_00987	hoi108_00033	hoi110_00493	hoi111_00375	hoi112_01195	hoi113_01178	hoi114_00965	hoi115_01266	hoi116_00921	hoi105_00635	hoi107_01048	hoi109_01178	hoi117_00787	hoi106_00540
948	hypothetical protein	16	Core	hoi102_00969	hoi103_00987	hoi104_00988	hoi108_00034	hoi110_00494	hoi111_00376	hoi112_01194	hoi113_01179	hoi114_00966	hoi115_01267	hoi116_00920	hoi105_00634	hoi107_01049	hoi109_01177	hoi117_00788	hoi106_00541
949	NADPH-dependent FMN reductase	16	Core	hoi102_00970	hoi103_00986	hoi104_00989	hoi108_00035	hoi110_00495	hoi111_00377	hoi112_01193	hoi113_01180	hoi114_00967	hoi115_01268	hoi116_00919	hoi105_00633	hoi107_01050	hoi109_01176	hoi117_00789	hoi106_00542
950	hypothetical protein	16	Core	hoi102_00971	hoi103_00985	hoi104_00990	hoi108_00036	hoi110_00496	hoi111_00378	hoi112_01192	hoi113_01181	hoi114_00968	hoi115_01269	hoi116_00918	hoi105_00631	hoi107_01052	hoi109_01174	hoi117_00791	hoi106_00544
951	hypothetical protein	16	Core	hoi102_00972	hoi103_00984	hoi104_00991	hoi108_00037	hoi110_00497	hoi111_00379	hoi112_01191	hoi113_01182	hoi114_00969	hoi115_01270	hoi116_00917	hoi105_00630	hoi107_01053	hoi109_01173	hoi117_00792	hoi106_00545
952	tRNA pseudouridine synthase A	16	Core	hoi102_00973	hoi103_00983	hoi104_00992	hoi108_00038	hoi110_00498	hoi111_00380	hoi112_01190	hoi113_01183	hoi114_00970	hoi115_01271	hoi116_00916	hoi105_00629	hoi107_01054	hoi109_01172	hoi117_00793	hoi106_00546
953	hypothetical protein	16	Core	hoi102_00974	hoi103_00982	hoi104_00993	hoi108_00039	hoi110_00499	hoi111_00381	hoi112_01189	hoi113_01184	hoi114_00971	hoi115_01272	hoi116_00915	hoi105_00628	hoi107_01055	hoi109_01171	hoi117_00794	hoi106_00547
954	Iron import ATP-binding/permease protein IrtA	16	Core	hoi102_00975	hoi103_00981	hoi104_00994	hoi108_00040	hoi110_00500	hoi111_00382	hoi112_01188	hoi113_01185	hoi114_00972	hoi115_01273	hoi116_00914	hoi105_00627	hoi107_01056	hoi109_01170	hoi117_00795	hoi106_00548
955	50S ribosomal protein L17	16	Core	hoi102_00976	hoi103_00980	hoi104_00995	hoi108_00041	hoi110_00501	hoi111_00383	hoi112_01187	hoi113_01186	hoi114_00973	hoi115_01274	hoi116_00913	hoi105_00626	hoi107_01057	hoi109_01169	hoi117_00796	hoi106_00549
956	DNA-directed RNA polymerase subunit alpha	16	Core	hoi102_00977	hoi103_00979	hoi104_00996	hoi108_00042	hoi110_00502	hoi111_00384	hoi112_01186	hoi113_01187	hoi114_00974	hoi115_01275	hoi116_00912	hoi105_00625	hoi107_01058	hoi109_01168	hoi117_00797	hoi106_00550
957	30S ribosomal protein S4	16	Core	hoi102_00978	hoi103_00978	hoi104_00997	hoi108_00043	hoi110_00503	hoi111_00385	hoi112_01185	hoi113_01188	hoi114_00975	hoi115_01276	hoi116_00911	hoi105_00624	hoi107_01059	hoi109_01167	hoi117_00798	hoi106_00551

958	30S ribosomal protein S11	16	Core	hoi102_00979	hoi103_00977	hoi104_00998	hoi108_00044	hoi110_00504	hoi111_00386	hoi112_01184	hoi113_01189	hoi114_00976	hoi115_01277	hoi116_00910	hoi105_00623	hoi107_01060	hoi109_01166	hoi117_00799	hoi106_00552
959	30S ribosomal protein S13	16	Core	hoi102_00980	hoi103_00976	hoi104_00999	hoi108_00045	hoi110_00505	hoi111_00387	hoi112_01183	hoi113_01190	hoi114_00977	hoi115_01278	hoi116_00909	hoi105_00622	hoi107_01061	hoi109_01165	hoi117_00800	hoi106_00553
960	Translation initiation factor IF-1	16	Core	hoi102_00981	hoi103_00975	hoi104_01000	hoi108_00046	hoi110_00506	hoi111_00388	hoi112_01182	hoi113_01191	hoi114_00978	hoi115_01279	hoi116_00908	hoi105_00621	hoi107_01062	hoi109_01164	hoi117_00801	hoi106_00554
961	putative acetyltransferase	16	Core	hoi102_00982	hoi103_00974	hoi104_01001	hoi108_00047	hoi110_00507	hoi111_00389	hoi112_01181	hoi113_01192	hoi114_00979	hoi115_01280	hoi116_00907	hoi105_00620	hoi107_01063	hoi109_01163	hoi117_00802	hoi106_00555
962	hypothetical protein	16	Core	hoi102_00983	hoi103_00973	hoi104_01002	hoi108_00048	hoi110_00508	hoi111_00390	hoi112_01180	hoi113_01193	hoi114_00980	hoi115_01281	hoi116_00906	hoi105_00619	hoi107_01064	hoi109_01162	hoi117_00803	hoi106_00556
963	Methionine aminopeptidase 1	16	Core	hoi102_00984	hoi103_01724	hoi104_01003	hoi108_00049	hoi110_00509	hoi111_00391	hoi112_01179	hoi113_01194	hoi114_00981	hoi115_01282	hoi116_00905	hoi105_00618	hoi107_01065	hoi109_01161	hoi117_00804	hoi106_00557
964	Adenylate kinase	16	Core	hoi102_00985	hoi103_01723	hoi104_01004	hoi108_00050	hoi110_00510	hoi111_00392	hoi112_01178	hoi113_01195	hoi114_00982	hoi115_01283	hoi116_00904	hoi105_00617	hoi107_01066	hoi109_01160	hoi117_00805	hoi106_00558
965	preprotein translocase subunit SecY	16	Core	hoi102_00986	hoi103_01722	hoi104_01005	hoi108_00051	hoi110_00511	hoi111_00393	hoi112_01177	hoi113_01196	hoi114_00983	hoi115_01284	hoi116_00903	hoi105_00616	hoi107_01067	hoi109_01159	hoi117_00806	hoi106_00559
966	50S ribosomal protein L15	16	Core	hoi102_00987	hoi103_01721	hoi104_01006	hoi108_00052	hoi110_00512	hoi111_00394	hoi112_01176	hoi113_01197	hoi114_00984	hoi115_01285	hoi116_00902	hoi105_00615	hoi107_01068	hoi109_01158	hoi117_00807	hoi106_00560
967	50S ribosomal protein L30	16	Core	hoi102_00988	hoi103_01720	hoi104_01007	hoi108_00053	hoi110_00513	hoi111_00395	hoi112_01175	hoi113_01198	hoi114_00985	hoi115_01286	hoi116_00901	hoi105_00614	hoi107_01069	hoi109_01157	hoi117_00808	hoi106_00561
968	30S ribosomal protein S5	16	Core	hoi102_00989	hoi103_01719	hoi104_01008	hoi108_00054	hoi110_00514	hoi111_00396	hoi112_01174	hoi113_01199	hoi114_00986	hoi115_01287	hoi116_00900	hoi105_00613	hoi107_01070	hoi109_01156	hoi117_00809	hoi106_00562
969	50S ribosomal protein L18	16	Core	hoi102_00990	hoi103_01718	hoi104_01009	hoi108_00055	hoi110_00515	hoi111_00397	hoi112_01173	hoi113_01200	hoi114_00987	hoi115_01288	hoi116_00899	hoi105_00612	hoi107_01071	hoi109_01155	hoi117_00810	hoi106_00563
970	50S ribosomal protein L6	16	Core	hoi102_00991	hoi103_01717	hoi104_01010	hoi108_00056	hoi110_00516	hoi111_00398	hoi112_01172	hoi113_01201	hoi114_00988	hoi115_01289	hoi116_00898	hoi105_00611	hoi107_01072	hoi109_01154	hoi117_00811	hoi106_00564
971	30S ribosomal protein S8	16	Core	hoi102_00992	hoi103_01716	hoi104_01011	hoi108_00057	hoi110_00517	hoi111_00399	hoi112_01171	hoi113_01202	hoi114_00989	hoi115_01290	hoi116_00897	hoi105_00610	hoi107_01073	hoi109_01153	hoi117_00812	hoi106_00565
972	Ribose import ATP-binding protein RbsA	16	Core	hoi102_00993	hoi103_01715	hoi104_01012	hoi108_00058	hoi110_00518	hoi111_00400	hoi112_01170	hoi113_01203	hoi114_00990	hoi115_01291	hoi116_00896	hoi105_00609	hoi107_01074	hoi109_01152	hoi117_00813	hoi106_00566
973	Ribose transport system permease protein RbsC	16	Core	hoi102_00994	hoi103_01714	hoi104_01013	hoi108_00059	hoi110_00519	hoi111_00401	hoi112_01169	hoi113_01204	hoi114_00991	hoi115_01292	hoi116_00895	hoi105_00608	hoi107_01075	hoi109_01151	hoi117_00814	hoi106_00567
974	TMAO reductase system periplasmic protein Tor	16	Core	hoi102_00995	hoi103_01713	hoi104_01014	hoi108_00060	hoi110_00520	hoi111_00402	hoi112_01168	hoi113_01205	hoi114_00992	hoi115_01293	hoi116_00894	hoi105_00607	hoi107_01076	hoi109_01150	hoi117_00815	hoi106_00568
975	Doxorubicin resistance ATP-binding protein DrrA	16	Core	hoi102_00996	hoi103_01712	hoi104_01015	hoi108_00061	hoi110_00521	hoi111_00403	hoi112_01167	hoi113_01206	hoi114_00993	hoi115_01294	hoi116_00893	hoi105_00606	hoi107_01077	hoi109_01149	hoi117_00816	hoi106_00569
976	Doxorubicin resistance ABC transporter permeas	16	Core	hoi102_00997	hoi103_01711	hoi104_01016	hoi108_00062	hoi110_00522	hoi111_00404	hoi112_01166	hoi113_01207	hoi114_00994	hoi115_01295	hoi116_00892	hoi105_00605	hoi107_01078	hoi109_01148	hoi117_00817	hoi106_00570
977	ABC-2 type transporter	16	Core	hoi102_00998	hoi103_01710	hoi104_01017	hoi108_00063	hoi110_00523	hoi111_00405	hoi112_01165	hoi113_01208	hoi114_00995	hoi115_01296	hoi116_00891	hoi105_00604	hoi107_01079	hoi109_01147	hoi117_00818	hoi106_00571
978	RNA-splicing ligase RtcB	16	Core	hoi102_00999	hoi103_01709	hoi104_01018	hoi108_00064	hoi110_00524	hoi111_00406	hoi112_01164	hoi113_01209	hoi114_00996	hoi115_01297	hoi116_00890	hoi105_00603	hoi107_01080	hoi109_01146	hoi117_00819	hoi106_00572
979	6-carboxyhexanoate--CoA ligase	16	Core	hoi102_01000	hoi103_01708	hoi104_01019	hoi108_00065	hoi110_00525	hoi111_00407	hoi112_01163	hoi113_01210	hoi114_00997	hoi115_01298	hoi116_00889	hoi105_00602	hoi107_01081	hoi109_01145	hoi117_00820	hoi106_00573
980	8-amino-7-oxononanoate synthase	16	Core	hoi102_01001	hoi103_01707	hoi104_01020	hoi108_00066	hoi110_00526	hoi111_00408	hoi112_01162	hoi113_01211	hoi114_00998	hoi115_01299	hoi116_00888	hoi105_00601	hoi107_01082	hoi109_01144	hoi117_00821	hoi106_00574
981	Formate dehydrogenase H	16	Core	hoi102_01002	hoi103_01706	hoi104_01021	hoi108_00067	hoi110_00527	hoi111_00409	hoi112_01161	hoi113_01212	hoi114_00999	hoi115_01300	hoi116_00887	hoi105_00600	hoi107_01083	hoi109_01143	hoi117_00822	hoi106_00575
982	hypothetical protein	16	Core	hoi102_01003	hoi103_01705	hoi104_01022	hoi108_00068	hoi110_00528	hoi111_00410	hoi112_01160	hoi113_01213	hoi114_01000	hoi115_01301	hoi116_00886	hoi105_00599	hoi107_01084	hoi109_01142	hoi117_00823	hoi106_00576
983	formate dehydrogenase accessory protein	16	Core	hoi102_01004	hoi103_01704	hoi104_01023	hoi108_00069	hoi110_00529	hoi111_00411	hoi112_01159	hoi113_01214	hoi114_01001	hoi115_01302	hoi116_00885	hoi105_00598	hoi107_01085	hoi109_01141	hoi117_00824	hoi106_00577
984	hypothetical protein	16	Core	hoi102_01005	hoi103_01703	hoi104_01024	hoi108_00070	hoi110_00530	hoi111_00412	hoi112_01158	hoi113_01215	hoi114_01002	hoi115_01303	hoi116_00884	hoi105_00597	hoi107_01086	hoi109_01140	hoi117_00825	hoi106_00578
985	Fatty acyl-CoA reductase	16	Core	hoi102_01006	hoi103_01702	hoi104_01025	hoi108_00071	hoi110_00531	hoi111_00413	hoi112_01157	hoi113_01216	hoi114_01003	hoi115_01304	hoi116_00883	hoi105_00596	hoi107_01087	hoi109_01139	hoi117_00826	hoi106_00579
986	hypothetical protein	16	Core	hoi102_01007	hoi103_01701	hoi104_01026	hoi108_00072	hoi110_00532	hoi111_00414	hoi112_01156	hoi113_01217	hoi114_01004	hoi115_01305	hoi116_00882	hoi105_00595	hoi107_01088	hoi109_01138	hoi117_00827	hoi106_00580
987	hypothetical protein	16	Core	hoi102_01008	hoi103_01700	hoi104_01027	hoi108_00073	hoi110_00533	hoi111_00415	hoi112_01155	hoi113_01218	hoi114_01005	hoi115_01306	hoi116_00881	hoi105_00594	hoi107_01089	hoi109_01137	hoi117_00828	hoi106_00581
988	Major phosphate-irrepressible acid phosphatase	16	Core	hoi102_01009	hoi103_01699	hoi104_01028	hoi108_00074	hoi110_00534	hoi111_00416	hoi112_01154	hoi113_01219	hoi114_01006	hoi115_01307	hoi116_00880	hoi105_00593	hoi107_01090	hoi109_01136	hoi117_00829	hoi106_00582
989	Phospholipase D precursor	16	Core	hoi102_01010	hoi103_01698	hoi104_01029	hoi108_00075	hoi110_00535	hoi111_00417	hoi112_01153	hoi113_01220	hoi114_01007	hoi115_01308	hoi116_00879	hoi105_00592	hoi107_01091	hoi109_01135	hoi117_00830	hoi106_00583
990	Pullulanase	16	Core	hoi102_01011	hoi103_01697	hoi104_01030	hoi108_00076	hoi110_00536	hoi111_00418	hoi112_01152	hoi113_01221	hoi114_01008	hoi115_01309	hoi116_00878	hoi105_00591	hoi107_01092	hoi109_01134	hoi117_00831	hoi106_00584
991	hypothetical protein	16	Core	hoi102_01012	hoi103_01696	hoi104_01031	hoi108_00077	hoi110_00537	hoi111_00419	hoi112_01151	hoi113_01222	hoi114_01009	hoi115_01310	hoi116_00877	hoi105_00590	hoi107_01093	hoi109_01133	hoi117_00832	hoi106_00585
992	50S ribosomal protein L5	16	Core	hoi102_01013	hoi103_01695	hoi104_01032	hoi108_00078	hoi110_00538	hoi111_00420	hoi112_01150	hoi113_01223	hoi114_01010	hoi115_01311	hoi116_00876	hoi105_00589	hoi107_01094	hoi109_01132	hoi117_00833	hoi106_00586
993	50S ribosomal protein L24	16	Core	hoi102_01014	hoi103_01694	hoi104_01033	hoi108_00079	hoi110_00539	hoi111_00421	hoi112_01149	hoi113_01224	hoi114_01011	hoi115_01312	hoi116_00875	hoi105_00588	hoi107_01095	hoi109_01131	hoi117_00834	hoi106_00587
994	50S ribosomal protein L14	16	Core	hoi102_01015	hoi103_01693	hoi104_01034	hoi108_00080	hoi110_00540	hoi111_00422	hoi112_01148	hoi113_01225	hoi114_01012	hoi115_01313	hoi116_00874	hoi105_00587	hoi107_01096	hoi109_01130	hoi117_00835	hoi106_00588
995	Bacterial regulatory proteins, tetR family	16	Core	hoi102_01016	hoi103_01692	hoi104_01035	hoi108_00081	hoi110_00541	hoi111_00423	hoi112_01147	hoi113_01226	hoi114_01013	hoi115_01314	hoi116_00873	hoi105_00586	hoi107_01097	hoi109_01129	hoi117_00836	hoi106_00589
996	3-oxoacyl-[acyl-carrier-protein] reductase FabG	16	Core	hoi102_01017	hoi103_01691	hoi104_01036	hoi108_00082	hoi110_00542	hoi111_00424	hoi112_01146	hoi113_01227	hoi114_01014	hoi115_01315	hoi116_00872	hoi105_00585	hoi107_01098	hoi109_01128	hoi117_00837	hoi106_00590
997	Vibriobactin utilization protein ViuB	16	Core	hoi102_01018	hoi103_01690	hoi104_01037	hoi108_00083	hoi110_00543	hoi111_00425	hoi112_01145	hoi113_01228	hoi114_01015	hoi115_01316	hoi116_00871	hoi105_00584	hoi107_01099	hoi109_01127	hoi117_00838	hoi106_00591
998	Ferric enterobactin transport system permease p	11	Accessory	hoi102_01019	hoi103_01689	hoi104_01038	hoi108_00084	hoi110_00544	hoi111_00426	hoi112_01144	hoi113_01229	hoi114_01016	hoi115_01317	hoi116_00870					
999	putative siderophore transport system permease	11	Accessory	hoi102_01020	hoi103_01688	hoi104_01039	hoi108_00085	hoi110_00545	hoi111_00427	hoi112_01143	hoi113_01230	hoi114_01017	hoi115_01318	hoi116_00869					
1000	putative siderophore transport system ATP-bindi	11	Accessory	hoi102_01021	hoi103_01687	hoi104_01040	hoi108_00086	hoi110_00546	hoi111_00428	hoi112_01142	hoi113_01231	hoi114_01018	hoi115_01319	hoi116_00868					
1001	Putative ABC transporter substrate-binding lipop	11	Accessory	hoi102_01022	hoi103_01686	hoi104_01041	hoi108_00087	hoi110_00547	hoi111_00429	hoi112_01141	hoi113_01232	hoi114_01019	hoi115_01320	hoi116_00867					
1002	Purine catabolism regulatory protein-like family p	16	Core	hoi102_01023	hoi103_01685	hoi104_01042	hoi108_00088	hoi110_00548	hoi111_00430	hoi112_01140	hoi113_01233	hoi114_01020	hoi115_01321	hoi116_00866	hoi105_00583	hoi107_01100	hoi109_01126	hoi117_00839	hoi106_00592
1003	4-aminobutyrate aminotransferase GabT	16	Core	hoi102_01024	hoi103_01684	hoi104_01043	hoi108_00089	hoi110_00549	hoi111_00431	hoi112_01139	hoi113_01234	hoi114_01021	hoi115_01322	hoi116_00865	hoi105_00582	hoi107_01101	hoi109_01125	hoi117_00840	hoi106_00593
1004	tryptophan permease	16	Core	hoi102_01025	hoi103_01683	hoi104_01044	hoi108_00090	hoi110_00550	hoi111_00432	hoi112_01138	hoi113_01235	hoi114_01022	hoi115_01323	hoi116_00864	hoi105_00581	hoi107_01102	hoi109_01124	hoi117_00841	hoi106_00594
1005	hypothetical protein	16	Core	hoi102_01026	hoi103_01682	hoi104_01045	hoi108_00091	hoi110_00551	hoi111_00433	hoi112_01137	hoi113_01236	hoi114_01023	hoi115_01324	hoi116_00863	hoi105_00580	hoi107_01103	hoi109_01123	hoi117_00842	hoi106_00595
1006	30S ribosomal protein S17	16	Core	hoi102_01027	hoi103_01681	hoi104_01046	hoi108_00092	hoi110_00552	hoi111_00434	hoi112_01136	hoi113_01237	hoi114_01024	hoi115_01325	hoi116_00862	hoi105_00576	hoi107_01107	hoi109_01119	hoi117_00846	hoi106_00599
1007	50S ribosomal protein L29	16	Core	hoi102_01028	hoi103_01680	hoi104_01047	hoi108_00093	hoi110_00553	hoi111_00435	hoi112_01135	hoi113_01238	hoi114_01025	hoi115_01326	hoi116_00861	hoi105_00575	hoi107_01108	hoi109_01118	hoi117_00847	hoi106_00600
1008	50S ribosomal protein L16	16	Core	hoi102_01029	hoi103_01679	hoi104_01048	hoi108_00094	hoi110_00554	hoi111_00436	hoi112_01134	hoi113_01239	hoi114_01026	hoi115_01327	hoi116_00860	hoi105_00574	hoi107_01109	hoi109_01117	hoi117_00848	hoi106_00601
1009	30S ribosomal protein S3	16	Core	hoi102_01030	hoi103_01678	hoi104_01049	hoi108_00095	hoi110_00555	hoi111_00437	hoi112_01133	hoi113_01240	hoi114_01027	hoi115_01328	hoi116_00859	hoi105_00573	hoi107_01110	hoi109_01116	hoi117_00849	hoi106_00602
1010	50S ribosomal protein L22	16	Core	hoi102_01031	hoi103_01677	hoi104_01050	hoi108_00096	hoi110_00556	hoi111_00438	hoi112_01132	hoi113_01241	hoi114_01028	hoi115_01329	hoi116_00858	hoi105_00572	hoi107_01111	hoi109_01115	hoi117_00850	hoi106_00603
1011	30S ribosomal protein S19	16	Core	hoi102_01032	hoi103_01676	hoi104_01051	hoi108_00097	hoi110_00557	hoi111_00439	hoi112_01131	hoi113_01242	hoi114_01029	hoi115_01330	hoi116_00857	hoi105_00571	hoi107_01112	hoi109_01114	hoi117_00851	hoi106_00604
1012	50S ribosomal protein L2	16	Core	hoi102_01033	hoi103_01675	hoi104_01052	hoi108_00098	hoi110_00558	hoi111_00440	hoi112_01130	hoi113_01243	hoi114_01030	hoi115_01331	hoi116_00856	hoi105_00570	hoi107_01113	hoi109_01113	hoi117_00852	hoi106_00605
1013	50S ribosomal protein L23	16	Core	hoi102_01034	hoi103_01674	hoi104_01053	hoi108_00099	hoi110_00559	hoi111_00441	hoi112_01129	hoi113_01244	hoi114_01031	hoi115_01332	hoi116_00855	hoi105_00569	hoi107_01114	hoi109_01112	hoi117_00853	hoi106_00606
1014	50S ribosomal protein L4	16	Core	hoi102_01035	hoi103_01673	hoi104_01054	hoi108_00100	hoi110_00560	hoi111_00442	hoi112_01128	hoi113_01245	hoi114_01032	hoi115_01333	hoi116_00854	hoi105_00568	hoi107_01115	hoi109_01111	hoi117_00854	hoi106_00607
1015	50S ribosomal protein L3	16	Core	hoi102_01036	hoi103_01672	hoi104_01055	hoi108_00101	hoi110_00561	hoi111_00443	hoi112_01127	hoi113_01246	hoi114_01033	hoi115_01334	hoi116_00853	hoi105_00567	hoi107_01116	hoi109_01110	hoi117_00855	hoi106_00608
1016	30S ribosomal protein S10	16	Core	hoi102_01037	hoi103_01671	hoi104_01056	hoi108_00102	hoi110_00562	hoi111_00444	hoi112_01126	hoi113_01247	hoi114_01034	hoi115_01335	hoi116_00852	hoi105_00566	hoi107_01117	hoi109_01109	hoi117_00856	hoi106_00609
1017	Trypsin	16	Core	hoi102_01038	hoi103_01670	hoi104_01057	hoi108_00103	hoi110_00563	hoi111_00445	hoi112_01125	hoi113_01248	hoi114_01035	hoi115_01336	hoi116_00851	hoi105_00565	hoi107_01118	hoi109_01108	hoi117_00857	hoi106_00610
1018	Alkaline shock protein 23	16	Core	hoi102_01039	hoi103_01669	hoi104_01058	hoi108_00104	hoi110_00564	hoi111_00446	hoi112_01124	hoi113_01249	hoi114_01036	hoi115_01337	hoi116_00850	hoi105_00564	hoi107_01119	hoi109_01107	hoi117_00858	hoi106_00611
1019	hypothetical protein	16	Core	hoi102_01040	hoi103_01668	hoi104_01059	hoi108_00105	hoi110_00565	hoi111_00447	hoi112_01123	hoi113_01250	hoi114_01037	hoi115_01338	hoi116_00849	hoi105_00563	hoi107_01120	hoi109_01106	hoi117_00859	hoi106_00612
1020	hypothetical protein	16	Core	hoi102_01041	hoi103_01667	hoi104_01060	hoi108_00106	hoi110_00566	hoi111_00448	hoi112_01122	hoi113_01251	hoi114_01038	hoi115_01339	hoi116_00848	hoi105_00562	hoi107_01121	hoi109_01105	hoi117_00860	hoi106_00613
1021	hypothetical protein	16	Core	hoi102_01042	hoi103_01666	hoi104_01061	hoi108_00107	hoi110_00567	hoi111_00449	hoi112_01121	hoi113_01252	hoi114_01039	hoi115_01340	hoi116_00847	hoi105_00561	hoi107_01122	hoi109_01104	hoi117_00861	hoi106_00614
1022	hypothetical protein	16	Core	hoi102_01043	hoi103_01665	hoi104_01062	hoi108_00108	hoi110_00568	hoi111_00450	hoi112_01120	hoi113_01253	hoi114_01040	hoi115_01341	hoi116_00846	hoi105_00560	hoi107_01123	hoi109_01103	hoi117_00862	hoi106_00615
1023	hypothetical protein	16	Core	hoi102_01044	hoi103_01664	hoi104_01063	hoi108_00109	hoi110_00569	hoi111_00451	hoi112_01119	hoi113_01254	hoi114_01041	hoi115_01342	hoi116_00845	hoi105_00559	hoi107_01124	hoi109_01102	hoi117_00863	hoi106_00616
1024	Elongation factor Tu	16	Core	hoi102_01045	hoi103_01663	hoi104_01064	hoi108_00110	hoi110_00570	hoi111_00452	hoi112_01118	hoi113_01255	hoi114_01042	hoi115_01343	hoi116_00844	hoi105_00558	hoi107_01125	hoi109_01101	hoi117_00864	hoi106_00617
1025	Elongation factor G	16	Core	hoi102_01046	hoi103_01662	hoi104_01065	hoi108_00111	hoi110_00571	hoi111_00453	hoi112_01117	hoi113_01256	hoi114_01043	hoi115_01344	hoi116_00843	hoi105_00557	hoi107_01126	hoi109_01100	hoi117_00865	hoi106_00618
1026	30S ribosomal protein S7	16	Core	hoi102_01047	hoi103_01661	hoi104_01066	hoi108_00112	hoi110_00572	hoi111_00454	hoi112_01116	hoi113_01257	hoi114_01044	hoi115_01345	hoi116_00842	hoi105_00556	hoi107_01127	hoi109_01099	hoi117_00866	hoi106_00619
1027	30S ribosomal protein S12	16	Core	hoi102_01048	hoi103_01660	hoi104_01067	hoi108_00113	hoi110_00573	hoi111_00455	hoi112_01115	hoi113_01258	hoi114_01045	hoi115_01346	hoi116_00841	hoi105_00555	hoi107_01128	hoi109_01098	hoi117_00867	hoi106_00620
1028	hypothetical protein	16	Core	hoi102_01049	hoi103_01659	hoi104_01068	hoi108_00114	hoi110_00574	hoi111_00456	hoi112_01114	hoi113_01259	hoi114_01046	hoi115_01347	hoi116_00840	hoi105_00554	hoi107_01129	hoi109_01097	hoi117_00868	hoi106_00621
1029	DNA-directed RNA polymerase subunit beta'	16	Core	hoi102_01050	hoi103_01658	hoi104_01069	hoi108_00115	hoi110_00575	hoi111_00457	hoi112_01113	hoi113_01260	hoi114_01047	hoi115_01348	hoi116_00839	hoi105_00553	hoi107_01130	hoi109_01096	hoi117_00869	hoi106_00622
1030	DNA-directed RNA polymerase subunit beta	16	Core	hoi102_01051	hoi103_01657	hoi104_01070	hoi108_00116	hoi110_00576	hoi111_00458	hoi112_01112	hoi113_01261	hoi114_01048	hoi115_01349	hoi116_00838	hoi105_00552	hoi107_01131	hoi109_01095	hoi117_00870	hoi106_00623
1031	hypothetical protein	16	Core	hoi102_01052	hoi103_01656	hoi104_01071	hoi108_00117	hoi110_00577	hoi111_00459	hoi112_01111	hoi113_01262	hoi114_01049	hoi115_01350	hoi116_00837	hoi105_00551	hoi107_01132	hoi109_01094	hoi117_00871	hoi106_00624
1032	SNARE associated Golgi protein	16	Core	hoi102_01053	hoi103_01655	hoi104_01072	hoi108_00118	hoi110_00578	hoi111_00460	hoi112_01110	hoi113_01263	hoi114_01050	hoi115_01351	hoi116_00836	hoi105_00550	hoi107_01133	hoi109_01093	hoi117_00872	hoi106_00625
1033	2,5-diketo-D-gluconic acid reductase B	16	Core	hoi102_01054	hoi103_01654	hoi104_01073	hoi108_00119	hoi110_00579	hoi111_00461	hoi112_01109	hoi113_01264	hoi114_01051	hoi115_01352	hoi116_00835	hoi105_00549	hoi107_01134	hoi109_01092	hoi117_00873	hoi106_00626
1034	Beta-glucosidase A	11	Accessory	hoi102_01055	hoi103_01653	hoi104_01074	hoi108_00120	hoi110_00580	hoi111_00462	hoi112_01108	hoi113_01265	hoi114_01052	hoi115_01353	hoi116_00834					
1035	Alpha/beta hydrolase family protein	16	Core	hoi102_01056	hoi103_01652	hoi104_01075	hoi108_00121	hoi110_00581	hoi111_00463	hoi112_01107	hoi113_01266	hoi114_01053	hoi115_01354	hoi116_00833	hoi105_00548	hoi107_01135	hoi109_01091	hoi117_00874	hoi106_00627
1036	hypothetical protein	16	Core	hoi102_01057	hoi103_01651	hoi104_01076	hoi108_00122	hoi110_00582	hoi111_00464	hoi112_01106	hoi113_01267	hoi114_01054	hoi115_01355	hoi116_00832	hoi105_00547	hoi107_01136	hoi109_01090	hoi117_00875	hoi106_00628
1037	50S ribosomal protein L7/L12	16	Core	hoi102_01058	hoi103_01650	hoi104_01077	hoi108_00123	hoi110_00583	hoi111_00465	hoi112_01105	hoi113_01268	hoi114_01055	hoi115_01356	hoi116_00831	hoi105_00546	hoi107_01137	hoi109_01089	hoi117_00876	hoi106_00629

1038	50S ribosomal protein L10	16	Core	hoi102_01059	hoi103_01649	hoi104_01078	hoi108_00124	hoi110_00584	hoi111_00466	hoi112_01104	hoi113_01269	hoi114_01056	hoi115_01357	hoi116_00830	hoi105_00545	hoi107_01138	hoi109_01088	hoi117_00877	hoi106_00630
1039	hypothetical protein	16	Core	hoi102_01060	hoi103_01648	hoi104_01079	hoi108_00125	hoi110_00585	hoi111_00467	hoi112_01103	hoi113_01270	hoi114_01057	hoi115_01358	hoi116_00829	hoi105_00544	hoi107_01139	hoi109_01087	hoi117_00878	hoi106_00631
1040	Acetyltransferase Pat	16	Core	hoi102_01061	hoi103_01647	hoi104_01080	hoi108_00126	hoi110_00586	hoi111_00468	hoi112_01102	hoi113_01271	hoi114_01058	hoi115_01359	hoi116_00828	hoi105_00543	hoi107_01140	hoi109_01086	hoi117_00879	hoi106_00632
1041	Acetoin utilization protein AcuC	16	Core	hoi102_01062	hoi103_01646	hoi104_01081	hoi108_00127	hoi110_00587	hoi111_00469	hoi112_01101	hoi113_01272	hoi114_01059	hoi115_01360	hoi116_00827	hoi105_00542	hoi107_01141	hoi109_01085	hoi117_00880	hoi106_00633
1042	putative HTH-type transcriptional regulator YdfH	16	Core	hoi102_01063	hoi103_01645	hoi104_01082	hoi108_00128	hoi110_00588	hoi111_00470	hoi112_01100	hoi113_01273	hoi114_01060	hoi115_01361	hoi116_00826	hoi105_00541	hoi107_01142	hoi109_01084	hoi117_00881	hoi106_00634
1043	Amino-acid carrier protein AlsT	16	Core	hoi102_01064	hoi103_01644	hoi104_01083	hoi108_00129	hoi110_00589	hoi111_00471	hoi112_01099	hoi113_01274	hoi114_01061	hoi115_01362	hoi116_00825	hoi105_00540	hoi107_01143	hoi109_01083	hoi117_00882	hoi106_00635
1044	Diacetyl reductase [(S)-acetoin forming]	16	Core	hoi102_01065	hoi103_01643	hoi104_01084	hoi108_00130	hoi110_00590	hoi111_00472	hoi112_01098	hoi113_01275	hoi114_01062	hoi115_01363	hoi116_00824	hoi105_00539	hoi107_01144	hoi109_01082	hoi117_00883	hoi106_00636
1045	Acyl-CoA dehydrogenase	16	Core	hoi102_01066	hoi103_01642	hoi104_01085	hoi108_00131	hoi110_00591	hoi111_00473	hoi112_01097	hoi113_01276	hoi114_01063	hoi115_01364	hoi116_00823	hoi105_00538	hoi107_01145	hoi109_01081	hoi117_00884	hoi106_00637
1046	hypothetical protein	16	Core	hoi102_01067	hoi103_01641	hoi104_01086	hoi108_00132	hoi110_00592	hoi111_00474	hoi112_01096	hoi113_01277	hoi114_01064	hoi115_01365	hoi116_00822	hoi105_00537	hoi107_01146	hoi109_01080	hoi117_00885	hoi106_00638
1047	Putative succinate-semialdehyde dehydrogenase	16	Core	hoi102_01068	hoi103_01640	hoi104_01087	hoi108_00133	hoi110_00593	hoi111_00475	hoi112_01095	hoi113_01278	hoi114_01065	hoi115_01366	hoi116_00821	hoi105_00536	hoi107_01147	hoi109_01079	hoi117_00886	hoi106_00639
1048	Acetyl-coenzyme A synthetase	16	Core	hoi102_01069	hoi103_01639	hoi104_01088	hoi108_00134	hoi110_00594	hoi111_00476	hoi112_01094	hoi113_01279	hoi114_01066	hoi115_01367	hoi116_00820	hoi105_00535	hoi107_01148	hoi109_01078	hoi117_00887	hoi106_00640
1049	putative enoyl-CoA hydratase echA8	16	Core	hoi102_01070	hoi103_01638	hoi104_01089	hoi108_00135	hoi110_00595	hoi111_00477	hoi112_01093	hoi113_01280	hoi114_01067	hoi115_01368	hoi116_00819	hoi105_00534	hoi107_01149	hoi109_01077	hoi117_00888	hoi106_00641
1050	putative acetyl-CoA acetyltransferase	16	Core	hoi102_01071	hoi103_01637	hoi104_01090	hoi108_00136	hoi110_00596	hoi111_00478	hoi112_01092	hoi113_01281	hoi114_01068	hoi115_01369	hoi116_00818	hoi105_00533	hoi107_01150	hoi109_01076	hoi117_00889	hoi106_00642
1051	hypothetical protein	16	Core	hoi102_01072	hoi103_01636	hoi104_01091	hoi108_00137	hoi110_00597	hoi111_00479	hoi112_01091	hoi113_01282	hoi114_01069	hoi115_01370	hoi116_00817	hoi105_00532	hoi107_01151	hoi109_01075	hoi117_00890	hoi106_00643
1052	hypothetical protein	16	Core	hoi102_01073	hoi103_01635	hoi104_01092	hoi108_00138	hoi110_00598	hoi111_00480	hoi112_01090	hoi113_01283	hoi114_01070	hoi115_01371	hoi116_00816	hoi105_00531	hoi107_01152	hoi109_01074	hoi117_00891	hoi106_00644
1053	hypothetical protein	16	Core	hoi102_01074	hoi103_01634	hoi104_01093	hoi108_00139	hoi110_00599	hoi111_00481	hoi112_01089	hoi113_01284	hoi114_01071	hoi115_01372	hoi116_00815	hoi105_00530	hoi107_01153	hoi109_01073	hoi117_00892	hoi106_00645
1054	hypothetical protein	11	Accessory	hoi102_01075	hoi103_01633	hoi104_01094	hoi108_00140	hoi110_00600	hoi111_00482	hoi112_01088	hoi113_01285	hoi114_01072	hoi115_01373	hoi116_00814					
1055	hypothetical protein	16	Core	hoi102_01076	hoi103_01632	hoi104_01095	hoi108_00141	hoi110_00601	hoi111_00483	hoi112_01087	hoi113_01286	hoi114_01073	hoi115_01374	hoi116_00813	hoi105_00529	hoi107_01155	hoi109_01072	hoi117_00893	hoi106_00646
1056	50S ribosomal protein L1	16	Core	hoi102_01077	hoi103_01631	hoi104_01096	hoi108_00142	hoi110_00602	hoi111_00484	hoi112_01086	hoi113_01287	hoi114_01074	hoi115_01375	hoi116_00812	hoi105_00528	hoi107_01156	hoi109_01071	hoi117_00894	hoi106_00647
1057	50S ribosomal protein L11	16	Core	hoi102_01078	hoi103_01630	hoi104_01097	hoi108_00143	hoi110_00603	hoi111_00485	hoi112_01085	hoi113_01288	hoi114_01075	hoi115_01376	hoi116_00811	hoi105_00527	hoi107_01157	hoi109_01070	hoi117_00895	hoi106_00648
1058	hypothetical protein	16	Core	hoi102_01079	hoi103_01629	hoi104_01098	hoi108_00144	hoi110_00604	hoi111_00486	hoi112_01084	hoi113_01289	hoi114_01076	hoi115_01377	hoi116_00810	hoi105_00526	hoi107_01158	hoi109_01069	hoi117_00896	hoi106_00649
1059	preprotein translocase subunit SecE	16	Core	hoi102_01080	hoi103_01628	hoi104_01099	hoi108_00145	hoi110_00605	hoi111_00487	hoi112_01083	hoi113_01290	hoi114_01077	hoi115_01378	hoi116_00809	hoi105_00525	hoi107_01159	hoi109_01068	hoi117_00897	hoi106_00650
1060	Heptaprenyl diphosphate synthase component 2	16	Core	hoi102_01085	hoi103_01623	hoi104_01104	hoi108_00150	hoi110_00610	hoi111_00492	hoi112_01078	hoi113_01295	hoi114_01082	hoi115_01383	hoi116_00804	hoi105_00520	hoi107_01164	hoi109_01063	hoi117_00902	hoi106_00655
1061	Putative oxidoreductase/MT0587	16	Core	hoi102_01086	hoi103_01622	hoi104_01105	hoi108_00151	hoi110_00611	hoi111_00493	hoi112_01077	hoi113_01296	hoi114_01083	hoi115_01384	hoi116_00803	hoi105_00519	hoi107_01165	hoi109_01062	hoi117_00903	hoi106_00656
1062	Ubiquinone/menaquinone biosynthesis C-methy	16	Core	hoi102_01087	hoi103_01621	hoi104_01106	hoi108_00152	hoi110_00612	hoi111_00494	hoi112_01076	hoi113_01297	hoi114_01084	hoi115_01385	hoi116_00802	hoi105_00518	hoi107_01166	hoi109_01061	hoi117_00904	hoi106_00657
1063	GDP-mannose-dependent alpha-mannosyltransf	16	Core	hoi102_01088	hoi103_01620	hoi104_01107	hoi108_00153	hoi110_00613	hoi111_00495	hoi112_01075	hoi113_01298	hoi114_01085	hoi115_01386	hoi116_00801	hoi105_00517	hoi107_01167	hoi109_01060	hoi117_00905	hoi106_00658
1064	hypothetical protein	16	Core	hoi102_01089	hoi103_01619	hoi104_01108	hoi108_00154	hoi110_00614	hoi111_00496	hoi112_01074	hoi113_01299	hoi114_01086	hoi115_01387	hoi116_00800	hoi105_00516	hoi107_01168	hoi109_01059	hoi117_00906	hoi106_00659
1065	2-succinyl-5-enolpyruvyl-6-hydroxy-3-cyclohexer	16	Core	hoi102_01090	hoi103_01618	hoi104_01109	hoi108_00155	hoi110_00615	hoi111_00497	hoi112_01073	hoi113_01300	hoi114_01087	hoi115_01388	hoi116_00799	hoi105_00515	hoi107_01169	hoi109_01058	hoi117_00907	hoi106_00660
1066	L-Ala-D/L-Glu epimerase	16	Core	hoi102_01091	hoi103_01617	hoi104_01110	hoi108_00156	hoi110_00616	hoi111_00498	hoi112_01072	hoi113_01301	hoi114_01088	hoi115_01389	hoi116_00798	hoi105_00514	hoi107_01170	hoi109_01057	hoi117_00908	hoi106_00661
1067	1,4-Dihydroxy-2-naphthoyl-CoA synthase	16	Core	hoi102_01092	hoi103_01616	hoi104_01111	hoi108_00157	hoi110_00617	hoi111_00499	hoi112_01071	hoi113_01302	hoi114_01089	hoi115_01390	hoi116_00797	hoi105_00513	hoi107_01171	hoi109_01056	hoi117_00909	hoi106_00662
1068	Dihydrolipoyl dehydrogenase	16	Core	hoi102_01093	hoi103_01615	hoi104_01112	hoi108_00158	hoi110_00618	hoi111_00500	hoi112_01070	hoi113_01303	hoi114_01090	hoi115_01391	hoi116_00796	hoi105_00512	hoi107_01172	hoi109_01055	hoi117_00910	hoi106_00663
1069	Diacylglycerol acyltransferase/mycolyltransferas	16	Core	hoi102_01095	hoi103_01613	hoi104_01114	hoi108_00160	hoi110_00620	hoi111_00502	hoi112_01068	hoi113_01305	hoi114_01092	hoi115_01393	hoi116_00794	hoi105_00510	hoi107_01174	hoi109_01053	hoi117_00912	hoi106_00665
1070	VanW like protein	16	Core	hoi102_01096	hoi103_01612	hoi104_01115	hoi108_00161	hoi110_00621	hoi111_00503	hoi112_01067	hoi113_01306	hoi114_01093	hoi115_01394	hoi116_00793	hoi105_00509	hoi107_01175	hoi109_01052	hoi117_00913	hoi106_00666
1071	Universal stress protein family protein	16	Core	hoi102_01097	hoi103_01611	hoi104_01116	hoi108_00162	hoi110_00622	hoi111_00504	hoi112_01066	hoi113_01307	hoi114_01094	hoi115_01395	hoi116_00792	hoi105_00508	hoi107_01176	hoi109_01051	hoi117_00914	hoi106_00667
1072	hypothetical protein	16	Core	hoi102_01098	hoi103_01610	hoi104_01117	hoi108_00163	hoi110_00623	hoi111_00505	hoi112_01065	hoi113_01308	hoi114_01095	hoi115_01396	hoi116_00791	hoi105_00507	hoi107_01177	hoi109_01050	hoi117_00915	hoi106_00668
1073	Alpha-(1->3)-arabinofuranosyltransferase	16	Core	hoi102_01099	hoi103_01609	hoi104_01118	hoi108_00164	hoi110_00624	hoi111_00506	hoi112_01064	hoi113_01309	hoi114_01096	hoi115_01397	hoi116_00790	hoi105_00506	hoi107_01178	hoi109_01049	hoi117_00916	hoi106_00669
1074	O-acetyltransferase OatA	16	Core	hoi102_01100	hoi103_01608	hoi104_01119	hoi108_00165	hoi110_00625	hoi111_00507	hoi112_01063	hoi113_01310	hoi114_01097	hoi115_01398	hoi116_00789	hoi105_00505	hoi107_01179	hoi109_01048	hoi117_00917	hoi106_00670
1075	hypothetical protein	16	Core	hoi102_01101	hoi103_01607	hoi104_01120	hoi108_00166	hoi110_00626	hoi111_00508	hoi112_01062	hoi113_01311	hoi114_01098	hoi115_01399	hoi116_00788	hoi105_00504	hoi107_01180	hoi109_01047	hoi117_00918	hoi106_00671
1076	hypothetical protein	16	Core	hoi102_01102	hoi103_01606	hoi104_01121	hoi108_00167	hoi110_00627	hoi111_00509	hoi112_01061	hoi113_01312	hoi114_01099	hoi115_01400	hoi116_00787	hoi105_00503	hoi107_01181	hoi109_01046	hoi117_00919	hoi106_00672
1077	GDP-mannose-dependent alpha-(1-6)-phosphati	16	Core	hoi102_01103	hoi103_01605	hoi104_01122	hoi108_00168	hoi110_00628	hoi111_00510	hoi112_01060	hoi113_01313	hoi114_01100	hoi115_01401	hoi116_00786	hoi105_00502	hoi107_01182	hoi109_01045	hoi117_00920	hoi106_00673
1078	putative methyltransferase	16	Core	hoi102_01104	hoi103_01604	hoi104_01123	hoi108_00169	hoi110_00629	hoi111_00511	hoi112_01059	hoi113_01314	hoi114_01101	hoi115_01402	hoi116_00785	hoi105_00501	hoi107_01183	hoi109_01044	hoi117_00921	hoi106_00674
1079	hypothetical protein	16	Core	hoi102_01105	hoi103_01603	hoi104_01124	hoi108_00170	hoi110_00630	hoi111_00512	hoi112_01058	hoi113_01315	hoi114_01102	hoi115_01403	hoi116_00784	hoi105_00500	hoi107_01184	hoi109_01043	hoi117_00922	hoi106_00675
1080	hypothetical protein	16	Core	hoi102_01106	hoi103_01602	hoi104_01125	hoi108_00171	hoi110_00631	hoi111_00513	hoi112_01057	hoi113_01316	hoi114_01103	hoi115_01404	hoi116_00783	hoi105_00499	hoi107_01185	hoi109_01042	hoi117_00923	hoi106_00676
1081	Phosphoenolpyruvate carboxykinase [GTP]	16	Core	hoi102_01107	hoi103_01601	hoi104_01126	hoi108_00172	hoi110_00632	hoi111_00514	hoi112_01056	hoi113_01317	hoi114_01104	hoi115_01405	hoi116_00782	hoi105_00498	hoi107_01186	hoi109_01041	hoi117_00924	hoi106_00677
1082	tRNA (guanine-N(7)-)-methyltransferase	16	Core	hoi102_01108	hoi103_01600	hoi104_01127	hoi108_00173	hoi110_00633	hoi111_00515	hoi112_01055	hoi113_01318	hoi114_01105	hoi115_01406	hoi116_00781	hoi105_00497	hoi107_01187	hoi109_01040	hoi117_00925	hoi106_00678
1083	NYN domain protein	16	Core	hoi102_01109	hoi103_01599	hoi104_01128	hoi108_00174	hoi110_00634	hoi111_00516	hoi112_01054	hoi113_01319	hoi114_01106	hoi115_01407	hoi116_00780	hoi105_00496	hoi107_01188	hoi109_01039	hoi117_00926	hoi106_00679
1084	Membrane protein YdfJ	16	Core	hoi102_01110	hoi103_01598	hoi104_01129	hoi108_00175	hoi110_00635	hoi111_00517	hoi112_01053	hoi113_01320	hoi114_01107	hoi115_01408	hoi116_00779	hoi105_00495	hoi107_01189	hoi109_01038	hoi117_00927	hoi106_00680
1085	hypothetical protein	16	Core	hoi102_01111	hoi103_01597	hoi104_01130	hoi108_00176	hoi110_00636	hoi111_00518	hoi112_01052	hoi113_01321	hoi114_01108	hoi115_01409	hoi116_00778	hoi105_00494	hoi107_01190	hoi109_01037	hoi117_00928	hoi106_00681
1086	hypothetical protein	16	Core	hoi102_01112	hoi103_01596	hoi104_01131	hoi108_00177	hoi110_00637	hoi111_00519	hoi112_01051	hoi113_01322	hoi114_01109	hoi115_01410	hoi116_00777	hoi105_00493	hoi107_01191	hoi109_01036	hoi117_00929	hoi106_00682
1087	putative propionyl-CoA carboxylase beta chain 5	16	Core	hoi102_01113	hoi103_01595	hoi104_01132	hoi108_00178	hoi110_00638	hoi111_00520	hoi112_01050	hoi113_01323	hoi114_01110	hoi115_01411	hoi116_00776	hoi105_00492	hoi107_01192	hoi109_01035	hoi117_00930	hoi106_00683
1088	Phthiocerol/phenolphthiocerol synthesis polyket	16	Core	hoi102_01114	hoi103_01594	hoi104_01133	hoi108_00179	hoi110_00639	hoi111_00521	hoi112_01049	hoi113_01324	hoi114_01111	hoi115_01412	hoi116_00775	hoi105_00491	hoi107_01193	hoi109_01034	hoi117_00931	hoi106_00684
1089	Long-chain-fatty-acid--AMP ligase FadD32	16	Core	hoi102_01115	hoi103_01593	hoi104_01134	hoi108_00180	hoi110_00640	hoi111_00522	hoi112_01048	hoi113_01325	hoi114_01112	hoi115_01413	hoi116_00774	hoi105_00490	hoi107_01194	hoi109_01033	hoi117_00932	hoi106_00685
1090	Cutinase	16	Core	hoi102_01116	hoi103_01592	hoi104_01135	hoi108_00181	hoi110_00641	hoi111_00523	hoi112_01047	hoi113_01326	hoi114_01113	hoi115_01414	hoi116_00773	hoi105_00489	hoi107_01195	hoi109_01032	hoi117_00933	hoi106_00686
1091	hypothetical protein	16	Core	hoi102_01117	hoi103_01591	hoi104_01136	hoi108_00182	hoi110_00642	hoi111_00524	hoi112_01046	hoi113_01327	hoi114_01114	hoi115_01415	hoi116_00772	hoi105_00488	hoi107_01196	hoi109_01031	hoi117_00934	hoi106_00687
1092	Diacylglycerol acyltransferase/mycolyltransferas	16	Core	hoi102_01118	hoi103_01590	hoi104_01137	hoi108_00183	hoi110_00643	hoi111_00525	hoi112_01045	hoi113_01328	hoi114_01115	hoi115_01416	hoi116_00771	hoi105_00487	hoi107_01197	hoi109_01030	hoi117_00935	hoi106_00688
1093	Diacylglycerol acyltransferase/mycolyltransferas	16	Core	hoi102_01119	hoi103_01589	hoi104_01138	hoi108_00184	hoi110_00644	hoi111_00526	hoi112_01044	hoi113_01329	hoi114_01116	hoi115_01417	hoi116_00770	hoi105_00486	hoi107_01198	hoi109_01029	hoi117_00936	hoi106_00689
1094	Decaprenyl-phosphate phosphoribosyltransferas	16	Core	hoi102_01120	hoi103_01588	hoi104_01139	hoi108_00185	hoi110_00645	hoi111_00527	hoi112_01043	hoi113_01330	hoi114_01117	hoi115_01418	hoi116_00769	hoi105_00485	hoi107_01199	hoi109_01028	hoi117_00937	hoi106_00690
1095	PAP2 superfamily protein	16	Core	hoi102_01121	hoi103_01587	hoi104_01140	hoi108_00186	hoi110_00646	hoi111_00528	hoi112_01042	hoi113_01331	hoi114_01118	hoi115_01419	hoi116_00768	hoi105_00484	hoi107_01200	hoi109_01027	hoi117_00938	hoi106_00691
1096	Galactofuranosyl transferase GlfT2	16	Core	hoi102_01122	hoi103_01586	hoi104_01141	hoi108_00187	hoi110_00647	hoi111_00529	hoi112_01041	hoi113_01332	hoi114_01119	hoi115_01420	hoi116_00767	hoi105_00483	hoi107_01201	hoi109_01026	hoi117_00939	hoi106_00692
1097	hypothetical protein	16	Core	hoi102_01123	hoi103_01585	hoi104_01142	hoi108_00188	hoi110_00648	hoi111_00530	hoi112_01040	hoi113_01333	hoi114_01120	hoi115_01421	hoi116_00766	hoi105_00482	hoi107_01202	hoi109_01025	hoi117_00940	hoi106_00693
1098	Putative trans-acting enoyl reductase	16	Core	hoi102_01124	hoi103_01584	hoi104_01143	hoi108_00189	hoi110_00649	hoi111_00531	hoi112_01039	hoi113_01334	hoi114_01121	hoi115_01422	hoi116_00765	hoi105_00481	hoi107_01203	hoi109_01024	hoi117_00941	hoi106_00694
1099	Mechanosensitive channel MscK precursor	16	Core	hoi102_01125	hoi103_01583	hoi104_01144	hoi108_00190	hoi110_00650	hoi111_00532	hoi112_01038	hoi113_01335	hoi114_01122	hoi115_01423	hoi116_00764	hoi105_00480	hoi107_01204	hoi109_01023	hoi117_00942	hoi106_00695
1100	Glycerol-3-phosphate transporter	16	Core	hoi102_01126	hoi103_01582	hoi104_01145	hoi108_00191	hoi110_00651	hoi111_00533	hoi112_01037	hoi113_01336	hoi114_01123	hoi115_01424	hoi116_00763	hoi105_00479	hoi107_01205	hoi109_01022	hoi117_00943	hoi106_00696
1101	UDP-galactopyranose mutase	16	Core	hoi102_01127	hoi103_01581	hoi104_01146	hoi108_00192	hoi110_00652	hoi111_00534	hoi112_01036	hoi113_01337	hoi114_01124	hoi115_01425	hoi116_00762	hoi105_00478	hoi107_01206	hoi109_01021	hoi117_00944	hoi106_00697
1102	N-acetylmuramoyl-L-alanine amidase	16	Core	hoi102_01128	hoi103_01580	hoi104_01147	hoi108_00193	hoi110_00653	hoi111_00535	hoi112_01035	hoi113_01338	hoi114_01125	hoi115_01426	hoi116_00761	hoi105_00477	hoi107_01207	hoi109_01020	hoi117_00945	hoi106_00698
1103	Putative phosphatase	16	Core	hoi102_01129	hoi103_01579	hoi104_01148	hoi108_00194	hoi110_00654	hoi111_00536	hoi112_01034	hoi113_01339	hoi114_01126	hoi115_01427	hoi116_00760	hoi105_00476	hoi107_01208	hoi109_01019	hoi117_00946	hoi106_00699
1104	1-acyl-sn-glycerol-3-phosphate acyltransferase	16	Core	hoi102_01130	hoi103_01578	hoi104_01149	hoi108_00195	hoi110_00655	hoi111_00537	hoi112_01033	hoi113_01340	hoi114_01127	hoi115_01428	hoi116_00759	hoi105_00475	hoi107_01209	hoi109_01018	hoi117_00947	hoi106_00700
1105	Serine--tRNA ligase	16	Core	hoi102_01131	hoi103_01577	hoi104_01150	hoi108_00196	hoi110_00656	hoi111_00538	hoi112_01032	hoi113_01341	hoi114_01128	hoi115_01429	hoi116_00758	hoi105_00474	hoi107_01210	hoi109_01017	hoi117_00948	hoi106_00701
1106	HTH-type transcriptional repressor DasR	16	Core	hoi102_01132	hoi103_01576	hoi104_01151	hoi108_00197	hoi110_00657	hoi111_00539	hoi112_01031	hoi113_01342	hoi114_01129	hoi115_01430	hoi116_00757	hoi105_00473	hoi107_01211	hoi109_01016	hoi117_00949	hoi106_00702
1107	putative membrane protein/MT3943	16	Core	hoi102_01133	hoi103_01575	hoi104_01152	hoi108_00198	hoi110_00658	hoi111_00540	hoi112_01030	hoi113_01343	hoi114_01130	hoi115_01431	hoi116_00756	hoi105_00472	hoi107_01212	hoi109_01015	hoi117_00950	hoi106_00703
1108	Possibl zinc metallo-peptidase	16	Core	hoi102_01134	hoi103_01574	hoi104_01153	hoi108_00199	hoi110_00659	hoi111_00541	hoi112_01029	hoi113_01344	hoi114_01131	hoi115_01432	hoi116_00755	hoi105_00471	hoi107_01213	hoi109_01014	hoi117_00951	hoi106_00704
1109	2,3-bisphosphoglycerate-dependent phosphogly	16	Core	hoi102_01135	hoi103_01573	hoi104_01154	hoi108_00200	hoi110_00660	hoi111_00542	hoi112_01028	hoi113_01345	hoi114_01132	hoi115_01433	hoi116_00754	hoi105_00470	hoi107_01214	hoi109_01013	hoi117_00952	hoi106_00705
1110	Prephenate dehydratase	16	Core	hoi102_01136	hoi103_01572	hoi104_01155	hoi108_00201	hoi110_00661	hoi111_00543	hoi112_01027	hoi113_01346	hoi114_01133	hoi115_01434	hoi116_00753	hoi105_00469	hoi107_01215	hoi109_01012	hoi117_00953	hoi106_00706
1111	Enantioselective amidase	16	Core	hoi102_01137	hoi103_01571	hoi104_01156	hoi108_00202	hoi110_00662	hoi111_00544	hoi112_01026	hoi113_01347	hoi114_01134	hoi115_01435	hoi116_00752	hoi105_00468	hoi107_01216	hoi109_01011	hoi117_00954	hoi106_00707
1112	CAAX amino terminal protease self- immunity	16	Core	hoi102_01138	hoi103_01570	hoi104_01157	hoi108_00203	hoi110_00663	hoi111_00545	hoi112_01025	hoi113_01348	hoi114_01135	hoi115_01436	hoi116_00751	hoi105_00467	hoi107_01217	hoi109_01010	hoi117_00955	hoi106_00708
1113	Regulatory protein MsrR	16	Core	hoi102_01139	hoi103_01569	hoi104_01158	hoi108_00204	hoi110_00664	hoi111_00546	hoi112_01024	hoi113_01349	hoi114_01136	hoi115_01437	hoi116_00750	hoi105_00466	hoi107_01218	hoi109_01009	hoi117_00956	hoi106_00709
1114	hypothetical protein	16	Core	hoi102_01140	hoi103_01568	hoi104_01159	hoi108_00205	hoi110_00665	hoi111_00547	hoi112_01023	hoi113_01350	hoi114_01137	hoi115_01438	hoi116_00749	hoi105_00465	hoi107_01219	hoi109_01008	hoi117_00957	hoi106_00710
1115	hypothetical protein	16	Core	hoi102_01141	hoi103_01567	hoi104_01160	hoi108_00206	hoi110_00666	hoi111_00548	hoi112_01022	hoi113_01351	hoi114_01138	hoi115_01439	hoi116_00748	hoi105_00464	hoi107_01220	hoi109_01007	hoi117_00958	hoi106_00711
1116	putative glycerophosphoryl diester phosphodiest	16	Core	hoi102_01142	hoi103_01566	hoi104_01161	hoi108_00207	hoi110_00667	hoi111_00549	hoi112_01021	hoi113_01352	hoi114_01139	hoi115_01440	hoi116_00747	hoi105_00463	hoi107_01221	hoi109_01006	hoi117_00959	hoi106_00712
1117	L-lactate dehydrogenase 1	16	Core	hoi102_01143	hoi103_01565	hoi104_01162	hoi108_00208	hoi110_00668	hoi111_00550	hoi112_01020	hoi113_01353	hoi114_01140	hoi115_01441	hoi116_00746	hoi105_00462	hoi107_01222	hoi109_01005	hoi117_00960	hoi106_00713

1118	Pyrimidine 5'-nucleotidase YjjG	16	Core		hoi102_01144	hoi103_01564	hoi104_01163	hoi108_00209	hoi110_00669	hoi111_00551	hoi112_01019	hoi113_01354	hoi114_01141	hoi115_01442	hoi116_00745	hoi105_00461	hoi107_01223	hoi109_01004	hoi117_00961	hoi106_00714
1119	phosphatidylglycerophosphatase B	16	Core		hoi102_01145	hoi103_01563	hoi104_01164	hoi108_00210	hoi110_00670	hoi111_00552	hoi112_01018	hoi113_01355	hoi114_01142	hoi115_01443	hoi116_00744	hoi105_00460	hoi107_01224	hoi109_01003	hoi117_00962	hoi106_00715
1120	Peptide methionine sulfoxide reductase MsrA	16	Core		hoi102_01146	hoi103_01562	hoi104_01165	hoi108_00211	hoi110_00671	hoi111_00553	hoi112_01017	hoi113_01356	hoi114_01143	hoi115_01444	hoi116_00743	hoi105_00459	hoi107_01225	hoi109_01002	hoi117_00963	hoi106_00716
1121	Superoxide dismutase [Mn]	16	Core		hoi102_01147	hoi103_01561	hoi104_01166	hoi108_00212	hoi110_00672	hoi111_00554	hoi112_01016	hoi113_01357	hoi114_01144	hoi115_01445	hoi116_00742	hoi105_00458	hoi107_01226	hoi109_01001	hoi117_00964	hoi106_00717
1122	Lipase 2 precursor	16	Core		hoi102_01148	hoi103_01560	hoi104_01167	hoi108_00213	hoi110_00673	hoi111_00555	hoi112_01015	hoi113_01358	hoi114_01145	hoi115_01446	hoi116_00741	hoi105_00457	hoi107_01227	hoi109_01000	hoi117_00965	hoi106_00718
1123	Aspartokinase	16	Core		hoi102_01149	hoi103_01559	hoi104_01168	hoi108_00214	hoi110_00674	hoi111_00556	hoi112_01014	hoi113_01359	hoi114_01146	hoi115_01447	hoi116_00740	hoi105_00456	hoi107_01228	hoi109_00999	hoi117_00966	hoi106_00719
1124	hypothetical protein	16	Core		hoi102_01150	hoi103_01558	hoi104_01169	hoi108_00215	hoi110_00675	hoi111_00557	hoi112_01013	hoi113_01360	hoi114_01147	hoi115_01448	hoi116_00739	hoi105_00455	hoi107_01229	hoi109_00998	hoi117_00967	hoi106_00720
1125	2-isopropylmalate synthase	16	Core		hoi102_01151	hoi103_01557	hoi104_01170	hoi108_00216	hoi110_00676	hoi111_00558	hoi112_01012	hoi113_01361	hoi114_01148	hoi115_01449	hoi116_00738	hoi105_00454	hoi107_01230	hoi109_00997	hoi117_00968	hoi106_00721
1126	Divalent metal cation transporter MntH	16	Core		hoi102_01152	hoi103_01556	hoi104_01171	hoi108_00217	hoi110_00677	hoi111_00559	hoi112_01011	hoi113_01362	hoi114_01149	hoi115_01450	hoi116_00737	hoi105_00453	hoi107_01231	hoi109_00996	hoi117_00969	hoi106_00722
1127	DNA polymerase III subunit epsilon	16	Core		hoi102_01153	hoi103_01555	hoi104_01172	hoi108_00218	hoi110_00678	hoi111_00560	hoi112_01010	hoi113_01363	hoi114_01150	hoi115_01451	hoi116_00736	hoi105_00452	hoi107_01232	hoi109_00995	hoi117_00970	hoi106_00723
1128	UDP-N-acetylmuramoyl-L-alanyl-D-glutamate syr	16	Core		hoi102_01154	hoi103_01554	hoi104_01173	hoi108_00219	hoi110_00679	hoi111_00561	hoi112_01009	hoi113_01364	hoi114_01151	hoi115_01452	hoi116_00735	hoi105_00451	hoi107_01233	hoi109_00994	hoi117_00971	hoi106_00724
1129	cobyric acid synthase	16	Core		hoi102_01155	hoi103_01553	hoi104_01174	hoi108_00220	hoi110_00680	hoi111_00562	hoi112_01008	hoi113_01365	hoi114_01152	hoi115_01453	hoi116_00734	hoi105_00450	hoi107_01234	hoi109_00993	hoi117_00972	hoi106_00725
1130	Recombination protein RecR	16	Core		hoi102_01156	hoi103_01552	hoi104_01175	hoi108_00221	hoi110_00681	hoi111_00563	hoi112_01007	hoi113_01366	hoi114_01153	hoi115_01454	hoi116_00733	hoi105_00449	hoi107_01235	hoi109_00992	hoi117_00973	hoi106_00726
1131	Nucleoid-associated protein/MT3819	16	Core		hoi102_01157	hoi103_01551	hoi104_01176	hoi108_00222	hoi110_00682	hoi111_00564	hoi112_01006	hoi113_01367	hoi114_01154	hoi115_01455	hoi116_00732	hoi105_00448	hoi107_01236	hoi109_00991	hoi117_00974	hoi106_00727
1132	hypothetical protein	12	Accessory		hoi102_01158	hoi103_01550	hoi104_01177	hoi108_00223	hoi110_00683	hoi111_00565	hoi112_01005	hoi113_01368	hoi114_01155			hoi105_00447	hoi107_01237		hoi117_00975	
1133	hypothetical protein	12	Accessory		hoi102_01159	hoi103_01171	hoi104_01178		hoi110_02069	hoi111_02122	hoi112_02039	hoi113_01369	hoi114_02059	hoi115_01457	hoi116_01160			hoi109_02153		hoi106_01997
1134	Putative glucose-6-phosphate 1-epimerase	16	Core		hoi102_01160	hoi103_01172	hoi104_01179	hoi108_00225	hoi110_02068	hoi111_02121	hoi112_02038	hoi113_01370	hoi114_02058	hoi115_01458	hoi116_01161	hoi105_00446	hoi107_01238	hoi109_02154	hoi117_00976	hoi106_01996
1135	Aspartate-semialdehyde dehydrogenase	16	Core		hoi102_01161	hoi103_01173	hoi104_01180	hoi108_00226	hoi110_02067	hoi111_02120	hoi112_02037	hoi113_01371	hoi114_02057	hoi115_01459	hoi116_01162	hoi105_00445	hoi107_01239	hoi109_02155	hoi117_00977	hoi106_01995
1136	HTH-type transcriptional regulator GltC	16	Core		hoi102_01162	hoi103_01174	hoi104_01181	hoi108_00227	hoi110_02066	hoi111_02119	hoi112_02036	hoi113_01372	hoi114_02056	hoi115_01460	hoi116_01163	hoi105_00444	hoi107_01240	hoi109_02156	hoi117_00978	hoi106_01994
1137	Inner membrane transport protein YnfM	16	Core		hoi102_01163	hoi103_01175	hoi104_01182	hoi108_00228	hoi110_02065	hoi111_02118	hoi112_02035	hoi113_01373	hoi114_02055	hoi115_01461	hoi116_01164	hoi105_00443	hoi107_01241	hoi109_02157	hoi117_00979	hoi106_01993
1138	Copper-containing nitrite reductase precursor	16	Core		hoi102_01164	hoi103_01176	hoi104_01183	hoi108_00229	hoi110_02064	hoi111_02117	hoi112_02034	hoi113_01374	hoi114_02054	hoi115_01462	hoi116_01165	hoi105_00442	hoi107_01242	hoi109_02158	hoi117_00980	hoi106_01992
1139	hypothetical protein	16	Core		hoi102_01165	hoi103_01177	hoi104_01184	hoi108_00230	hoi110_02063	hoi111_02116	hoi112_02033	hoi113_01375	hoi114_02053	hoi115_01463	hoi116_01166	hoi105_00441	hoi107_01243	hoi109_02159	hoi117_00981	hoi106_01991
1140	Nucleoid-associated protein Lsr2	16	Core		hoi102_01166	hoi103_01178	hoi104_01185	hoi108_00231	hoi110_02062	hoi111_02115	hoi112_02032	hoi113_01376	hoi114_02052	hoi115_01464	hoi116_01167	hoi105_00440	hoi107_01244	hoi109_02160	hoi117_00982	hoi106_01990
1141	hypothetical protein	16	Core		hoi102_01167	hoi103_01179	hoi104_01186	hoi108_00232	hoi110_02061	hoi111_02114	hoi112_02031	hoi113_01377	hoi114_02051	hoi115_01465	hoi116_01168	hoi105_00439	hoi107_01245	hoi109_02161	hoi117_00983	hoi106_01989
1142	hypothetical protein	16	Core		hoi102_01168	hoi103_01180	hoi104_01187	hoi108_00233	hoi110_02060	hoi111_02113	hoi112_02030	hoi113_01378	hoi114_02050	hoi115_01466	hoi116_01169	hoi105_00438	hoi107_01246	hoi109_02162	hoi117_00984	hoi106_01988
1143	Sortase family protein	16	Core		hoi102_01169	hoi103_01181	hoi104_01188	hoi108_00234	hoi110_02059	hoi111_02112	hoi112_02029	hoi113_01379	hoi114_02049	hoi115_01467	hoi116_01170	hoi105_00437	hoi107_01247	hoi109_02163	hoi117_00985	hoi106_01987
1144	Membrane protein insertase MisCB precursor	16	Core		hoi102_01170	hoi103_01182	hoi104_01189	hoi108_00235	hoi110_02058	hoi111_02111	hoi112_02028	hoi113_01380	hoi114_02048	hoi115_01468	hoi116_01171	hoi105_00436	hoi107_01248	hoi109_02164	hoi117_00986	hoi106_01986
1145	putative HTH-type transcriptional regulator YxaF	16	Core		hoi102_01171	hoi103_01183	hoi104_01190	hoi108_00236	hoi110_02057	hoi111_02110	hoi112_02027	hoi113_01381	hoi114_02047	hoi115_01469	hoi116_01172	hoi105_00435	hoi107_01249	hoi109_02165	hoi117_00987	hoi106_01985
1146	hypothetical protein	16	Core		hoi102_01172	hoi103_01184	hoi104_01191	hoi108_00237	hoi110_02056	hoi111_02109	hoi112_02026	hoi113_01382	hoi114_02046	hoi115_01470	hoi116_01173	hoi105_00434	hoi107_01250	hoi109_02166	hoi117_00988	hoi106_01984
1147	Universal stress protein/MT2061	16	Core		hoi102_01173	hoi103_01185	hoi104_01192	hoi108_00238	hoi110_02055	hoi111_02108	hoi112_02025	hoi113_01383	hoi114_02045	hoi115_01471	hoi116_01174	hoi105_00433	hoi107_01251	hoi109_02167	hoi117_00989	hoi106_01983
1148	hypothetical protein	11	Accessory		hoi102_01174	hoi103_01186	hoi104_01194	hoi108_00239	hoi110_02054	hoi111_02045	hoi112_02024	hoi113_01384	hoi114_02044	hoi115_01472	hoi116_01175					
1149	CsbD-like protein	16	Core		hoi102_01176	hoi103_01188	hoi104_01196	hoi108_00241	hoi110_02052	hoi111_02047	hoi112_02022	hoi113_01386	hoi114_02042	hoi115_01474	hoi116_01177	hoi105_00431	hoi107_01253	hoi109_02169	hoi117_00991	hoi106_01981
1150	tRNA-specific adenosine deaminase	16	Core		hoi102_01177	hoi103_01189	hoi104_01197	hoi108_00242	hoi110_02051	hoi111_02048	hoi112_02021	hoi113_01387	hoi114_02041	hoi115_01475	hoi116_01178	hoi105_00430	hoi107_01254	hoi109_02170	hoi117_00992	hoi106_01980
1151	hypothetical protein	16	Core		hoi102_01178	hoi103_01190	hoi104_01198	hoi108_00243	hoi110_02050	hoi111_02049	hoi112_02020	hoi113_01388	hoi114_02040	hoi115_01476	hoi116_01179	hoi105_00429	hoi107_01255	hoi109_02171	hoi117_00993	hoi106_01979
1152	prephenate dehydrogenase	16	Core		hoi102_01179	hoi103_01191	hoi104_01199	hoi108_00244	hoi110_02049	hoi111_02050	hoi112_02019	hoi113_01389	hoi114_02039	hoi115_01477	hoi116_01180	hoi105_00428	hoi107_01256	hoi109_02172	hoi117_00994	hoi106_01978
1153	Sodium-dependent dicarboxylate transporter Sd	16	Core		hoi102_01181	hoi103_01193	hoi104_01201	hoi108_00246	hoi110_02047	hoi111_02052	hoi112_02017	hoi113_01391	hoi114_02037	hoi115_01479	hoi116_01182	hoi105_00426	hoi107_01258	hoi109_02174	hoi117_00996	hoi106_01976
1154	Glucose-specific phosphotransferase enzyme IIA	16	Core		hoi102_01182	hoi103_01194	hoi104_01202	hoi108_00247	hoi110_02046	hoi111_02053	hoi112_02016	hoi113_01392	hoi114_02036	hoi115_01480	hoi116_01183	hoi105_00425	hoi107_01259	hoi109_02175	hoi117_00997	hoi106_01975
1155	PTS system glucose-specific EIICBA component	16	Core		hoi102_01183	hoi103_01195	hoi104_01203	hoi108_00248	hoi110_02045	hoi111_02054	hoi112_02015	hoi113_01393	hoi114_02035	hoi115_01481	hoi116_01184	hoi105_00424	hoi107_01260	hoi109_02176	hoi117_00998	hoi106_01974
1156	Cryptic beta-glucoside bgl operon antiterminator	16	Core		hoi102_01184	hoi103_01196	hoi104_01204	hoi108_00249	hoi110_02044	hoi111_02055	hoi112_02014	hoi113_01394	hoi114_02034	hoi115_01482	hoi116_01185	hoi105_00423	hoi107_01261	hoi109_02177	hoi117_00999	hoi106_01973
1157	Glycerophosphoryl diester phosphodiesterase pr	16	Core		hoi102_01185	hoi103_01197	hoi104_01205	hoi108_00250	hoi110_02043	hoi111_02056	hoi112_02013	hoi113_01395	hoi114_02033	hoi115_01483	hoi116_01186	hoi105_00422	hoi107_01262	hoi109_02178	hoi117_01000	hoi106_01972
1158	Major Facilitator Superfamily protein	16	Core		hoi102_01186	hoi103_01198	hoi104_01206	hoi108_00251	hoi110_02042	hoi111_02057	hoi112_02012	hoi113_01396	hoi114_02032	hoi115_01484	hoi116_01187	hoi105_00421	hoi107_01263	hoi109_02179	hoi117_01001	hoi106_01971
1159	Putative phenylalanine aminotransferase	16	Core		hoi102_01189	hoi103_01201	hoi104_01209	hoi108_00254	hoi110_02039	hoi111_02060	hoi112_02009	hoi113_01399	hoi114_02029	hoi115_01487	hoi116_01190	hoi105_00418	hoi107_01266	hoi109_02182	hoi117_01004	hoi106_01968
1160	Quinone oxidoreductase 1	16	Core		hoi102_01191	hoi103_01203	hoi104_01211	hoi108_00256	hoi110_02037	hoi111_02062	hoi112_02007	hoi113_01401	hoi114_02027	hoi115_01489	hoi116_01192	hoi105_00416	hoi107_01268	hoi109_02184	hoi117_01006	hoi106_01966
1161	Aminotransferase class-V	16	Core		hoi102_01192	hoi103_01204	hoi104_01212	hoi108_00257	hoi110_02036	hoi111_02063	hoi112_02006	hoi113_01402	hoi114_02026	hoi115_01490	hoi116_01193	hoi105_00415	hoi107_01269	hoi109_02185	hoi117_01007	hoi106_01965
1162	Teichoic acid translocation permease protein Tag	16	Core		hoi102_01193	hoi103_01205	hoi104_01213	hoi108_00258	hoi110_02035	hoi111_02064	hoi112_02005	hoi113_01403	hoi114_02025	hoi115_01491	hoi116_01194	hoi105_00414	hoi107_01270	hoi109_02186	hoi117_01008	hoi106_01964
1163	Teichoic acids export ATP-binding protein TagH	16	Core		hoi102_01194	hoi103_01206	hoi104_01214	hoi108_00259	hoi110_02034	hoi111_02065	hoi112_02004	hoi113_01404	hoi114_02024	hoi115_01492	hoi116_01195	hoi105_00413	hoi107_01271	hoi109_02187	hoi117_01009	hoi106_01963
1164	Galactofuranosyl transferase GlfT1	16	Core		hoi102_01195	hoi103_01207	hoi104_01215	hoi108_00260	hoi110_02033	hoi111_02066	hoi112_02003	hoi113_01405	hoi114_02023	hoi115_01493	hoi116_01196	hoi105_00412	hoi107_01272	hoi109_02188	hoi117_01010	hoi106_01962
1165	GtrA-like protein	16	Core		hoi102_01196	hoi103_01208	hoi104_01216	hoi108_00261	hoi110_02032	hoi111_02067	hoi112_02002	hoi113_01406	hoi114_02022	hoi115_01494	hoi116_01197	hoi105_00411	hoi107_01273	hoi109_02189	hoi117_01011	hoi106_01961
1166	hypothetical protein	16	Core		hoi102_01197	hoi103_01209	hoi104_01217	hoi108_00262	hoi110_02031	hoi111_02068	hoi112_02001	hoi113_01407	hoi114_02021	hoi115_01495	hoi116_01198	hoi105_00410	hoi107_01274	hoi109_02190	hoi117_01012	hoi106_01960
1167	hypothetical protein	16	Core		hoi102_01198	hoi103_01210	hoi104_01218	hoi108_00263	hoi110_02030	hoi111_02069	hoi112_02000	hoi113_01408	hoi114_02020	hoi115_01496	hoi116_01199	hoi105_00409	hoi107_01275	hoi109_02191	hoi117_01013	hoi106_01959
1168	hypothetical protein	16	Core		hoi102_01199	hoi103_01211	hoi104_01219	hoi108_00264	hoi110_02029	hoi111_02070	hoi112_01999	hoi113_01409	hoi114_02019	hoi115_01497	hoi116_01200	hoi105_00408	hoi107_01276	hoi109_02192	hoi117_01014	hoi106_01958
1169	hypothetical protein	16	Core		hoi102_01200	hoi103_01212	hoi104_01220	hoi108_00265	hoi110_02028	hoi111_02071	hoi112_01998	hoi113_01410	hoi114_02018	hoi115_01498	hoi116_01201	hoi105_00407	hoi107_01277	hoi109_02193	hoi117_01015	hoi106_01957
1170	hypothetical protein	16	Core		hoi102_01201	hoi103_01213	hoi104_01221	hoi108_00266	hoi110_02027	hoi111_02072	hoi112_01997	hoi113_01411	hoi114_02017	hoi115_01499	hoi116_01202	hoi105_00406	hoi107_01278	hoi109_02194	hoi117_01016	hoi106_01956
1171	hypothetical protein	16	Core		hoi102_01202	hoi103_01214	hoi104_01222	hoi108_00267	hoi110_02026	hoi111_02073	hoi112_01996	hoi113_01412	hoi114_02016	hoi115_01500	hoi116_01203	hoi105_00405	hoi107_01279	hoi109_02195	hoi117_01017	hoi106_01955
1172	N-acetylglucosamine-6-phosphate deacetylase	16	Core	GI6	hoi102_01203	hoi103_01215	hoi104_01223	hoi108_00268	hoi110_02025	hoi111_02074	hoi112_01995	hoi113_01413	hoi114_02015	hoi115_01501	hoi116_01204	hoi105_00404	hoi107_01280	hoi109_02196	hoi117_01018	hoi106_01954
1173	Fructose-bisphosphate aldolase	16	Core	GI6	hoi102_01204	hoi103_01216	hoi104_01224	hoi108_00269	hoi110_02024	hoi111_02075	hoi112_01994	hoi113_01414	hoi114_02014	hoi115_01502	hoi116_01205	hoi105_00403	hoi107_01281	hoi109_02197	hoi117_01019	hoi106_01953
1174	Tagatose-6-phosphate kinase	16	Core	GI6	hoi102_01205	hoi103_01217	hoi104_01225	hoi108_00270	hoi110_02023	hoi111_02076	hoi112_01993	hoi113_01415	hoi114_02013	hoi115_01503	hoi116_01206	hoi105_00402	hoi107_01282	hoi109_02198	hoi117_01020	hoi106_01952
1175	Glutamine--fructose-6-phosphate aminotransfer	16	Core	GI6	hoi102_01206	hoi103_01218	hoi104_01226	hoi108_00271	hoi110_02022	hoi111_02077	hoi112_01992	hoi113_01416	hoi114_02012	hoi115_01504	hoi116_01207	hoi105_00401	hoi107_01283	hoi109_02199	hoi117_01021	hoi106_01951
1176	Ascorbate-specific phosphotransferase enzyme I	16	Core	GI6	hoi102_01207	hoi103_01219	hoi104_01227	hoi108_00272	hoi110_02021	hoi111_02078	hoi112_01991	hoi113_01417	hoi114_02011	hoi115_01505	hoi116_01208	hoi105_00400	hoi107_01284	hoi109_02200	hoi117_01022	hoi106_01950
1177	Ascorbate-specific phosphotransferase enzyme I	16	Core	GI6	hoi102_01208	hoi103_01220	hoi104_01228	hoi108_00273	hoi110_02020	hoi111_02079	hoi112_01990	hoi113_01418	hoi114_02010	hoi115_01506	hoi116_01209	hoi105_00399	hoi107_01285	hoi109_02201	hoi117_01023	hoi106_01949
1178	Ascorbate-specific permease IIC component Ula	16	Core	GI6	hoi102_01209	hoi103_01221	hoi104_01229	hoi108_00274	hoi110_02019	hoi111_02080	hoi112_01989	hoi113_01419	hoi114_02009	hoi115_01507	hoi116_01210	hoi105_00398	hoi107_01286	hoi109_02202	hoi117_01024	hoi106_01948
1179	hypothetical protein	16	Core	GI6	hoi102_01210	hoi103_01222	hoi104_01230	hoi108_00275	hoi110_02018	hoi111_02081	hoi112_01988	hoi113_01420	hoi114_02008	hoi115_01508	hoi116_01211	hoi105_00397	hoi107_01287	hoi109_02203	hoi117_01025	hoi106_01947
1180	Fructose-bisphosphate aldolase	16	Core		hoi102_01211	hoi103_01223	hoi104_01231	hoi108_00276	hoi110_01212	hoi111_01142	hoi112_01704	hoi113_01421	hoi114_01305	hoi115_01509	hoi116_01212	hoi105_01587	hoi107_01613	hoi109_01545	hoi117_01946	hoi106_02068
1181	Glucitol operon repressor	16	Core		hoi102_01212	hoi103_01224	hoi104_01232	hoi108_00277	hoi110_01213	hoi111_01143	hoi112_01703	hoi113_01422	hoi114_01306	hoi115_01510	hoi116_01213	hoi105_01586	hoi107_01612	hoi109_01544	hoi117_01945	hoi106_02069
1182	RNA polymerase-associated protein RapA	16	Core		hoi102_01213	hoi103_01225	hoi104_01233	hoi108_00278	hoi110_01214	hoi111_01144	hoi112_01702	hoi113_01423	hoi114_01307	hoi115_01511	hoi116_01214	hoi105_01585	hoi107_01611	hoi109_01543	hoi117_01944	hoi106_02070
1183	hypothetical protein	16	Core		hoi102_01214	hoi103_01226	hoi104_01234	hoi108_00279	hoi110_01215	hoi111_01145	hoi112_01701	hoi113_01424	hoi114_01308	hoi115_01512	hoi116_01215	hoi105_01584	hoi107_01610	hoi109_01542	hoi117_01943	hoi106_02071
1184	ATP-dependent RNA helicase SrmB	16	Core		hoi102_01215	hoi103_01227	hoi104_01235	hoi108_00280	hoi110_01216	hoi111_01146	hoi112_01700	hoi113_01425	hoi114_01309	hoi115_01513	hoi116_01216	hoi105_01583	hoi107_01609	hoi109_01541	hoi117_01942	hoi106_02072
1185	Trehalose import ATP-binding protein SugC	16	Core		hoi102_01216	hoi103_01228	hoi104_01236	hoi108_00281	hoi110_01217	hoi111_01147	hoi112_01699	hoi113_01426	hoi114_01310	hoi115_01514	hoi116_01217	hoi105_01582	hoi107_01608	hoi109_01540	hoi117_01941	hoi106_02073
1186	hypothetical protein	16	Core		hoi102_01217	hoi103_01229	hoi104_01237	hoi108_00282	hoi110_01218	hoi111_01148	hoi112_01698	hoi113_01427	hoi114_01311	hoi115_01515	hoi116_01218	hoi105_01581	hoi107_01607	hoi109_01539	hoi117_01940	hoi106_02074
1187	hypothetical protein	16	Core		hoi102_01218	hoi103_01230	hoi104_01238	hoi108_00283	hoi110_01219	hoi111_01149	hoi112_01697	hoi113_01428	hoi114_01312	hoi115_01516	hoi116_01219	hoi105_01580	hoi107_01606	hoi109_01538	hoi117_01939	hoi106_02075
1188	Daunorubicin/doxorubicin resistance ATP-bindin	11	Accessory		hoi102_01219	hoi103_01231	hoi104_01239	hoi108_00284	hoi110_01220	hoi111_01150	hoi112_01696	hoi113_01429	hoi114_01313	hoi115_01517	hoi116_01220					
1189	hypothetical protein	11	Accessory		hoi102_01220	hoi103_01232	hoi104_01240	hoi108_00285	hoi110_01221	hoi111_01151	hoi112_01695	hoi113_01430	hoi114_01314	hoi115_01518	hoi116_01221					
1190	Sensor histidine kinase DesK	11	Accessory		hoi102_01221	hoi103_01233	hoi104_01241	hoi108_00286	hoi110_01222	hoi111_01152	hoi112_01694	hoi113_01431	hoi114_01315	hoi115_01519	hoi116_01222					
1191	Transcriptional regulatory protein UhpA	11	Accessory		hoi102_01222	hoi103_01234	hoi104_01242	hoi108_00287	hoi110_01223	hoi111_01153	hoi112_01693	hoi113_01432	hoi114_01316	hoi115_01520	hoi116_01223					
1192	ATP-dependent DNA helicase UvrD1	16	Core		hoi102_01223	hoi103_01235	hoi104_01243	hoi108_00288	hoi110_01224	hoi111_01154	hoi112_01692	hoi113_01433	hoi114_01317	hoi115_01521	hoi116_01224	hoi105_01579	hoi107_01605	hoi109_01537	hoi117_01938	hoi106_02076
1193	putative decaprenylphosphoryl-beta-D-ribose ox	16	Core		hoi102_01224	hoi103_01236	hoi104_01244	hoi108_00289	hoi110_01225	hoi111_01155	hoi112_01691	hoi113_01434	hoi114_01318	hoi115_01522	hoi116_01225	hoi105_01578	hoi107_01604	hoi109_01536	hoi117_01937	hoi106_02077
1194	putative oxidoreductase	16	Core		hoi102_01225	hoi103_01237	hoi104_01245	hoi108_00290	hoi110_01226	hoi111_01156	hoi112_01690	hoi113_01435	hoi114_01319	hoi115_01523	hoi116_01226	hoi105_01577	hoi107_01603	hoi109_01535	hoi117_01936	hoi106_02078
1195	Arabinofuranosyltransferase AftA	16	Core		hoi102_01226	hoi103_01238	hoi104_01246	hoi108_00291	hoi110_01227	hoi111_01157	hoi112_01689	hoi113_01436	hoi114_01320	hoi115_01524	hoi116_01227	hoi105_01576	hoi107_01602	hoi109_01534	hoi117_01935	hoi106_01175
1196	putative arabinosyltransferase C	16	Core		hoi102_01227	hoi103_01239	hoi104_01247	hoi108_00292	hoi110_01228	hoi111_01158	hoi112_01688	hoi113_01437	hoi114_01321	hoi115_01525	hoi116_01228	hoi105_01575	hoi107_01601	hoi109_01533	hoi117_01934	hoi106_01174
1197	NADPH-dependent curcumin reductase	11	Accessory		hoi102_01228	hoi103_01240	hoi104_01248	hoi108_00293	hoi110_01229	hoi111_01159	hoi112_01687	hoi113_01438	hoi114_01322	hoi115_01526	hoi116_01229					

1198	hypothetical protein	16	Core		hoi102_01229	hoi103_01241	hoi104_01249	hoi108_00294	hoi110_01230	hoi111_01160	hoi112_01686	hoi113_01439	hoi114_01323	hoi115_01527	hoi116_01230	hoi105_01574	hoi107_01600	hoi109_01532	hoi117_01933	hoi106_01173
1199	hypothetical protein	16	Core		hoi102_01230	hoi103_01242	hoi104_01250	hoi108_00295	hoi110_01231	hoi111_01161	hoi112_01685	hoi113_01440	hoi114_01324	hoi115_01528	hoi116_01231	hoi105_01573	hoi107_01599	hoi109_01531	hoi117_01932	hoi106_01172
1200	hypothetical protein	16	Core		hoi102_01231	hoi103_01243	hoi104_01251	hoi108_00296	hoi110_01232	hoi111_01162	hoi112_01684	hoi113_01441	hoi114_01325	hoi115_01529	hoi116_01232	hoi105_01572	hoi107_01598	hoi109_01530	hoi117_01931	hoi106_01171
1201	hypothetical protein	16	Core		hoi102_01232	hoi103_01244	hoi104_01252	hoi108_00297	hoi110_01233	hoi111_01163	hoi112_01683	hoi113_01442	hoi114_01326	hoi115_01530	hoi116_01233	hoi105_01571	hoi107_01597	hoi109_01529	hoi117_01930	hoi106_01170
1202	Neutral endopeptidase	16	Core		hoi102_01233	hoi103_01245	hoi104_01253	hoi108_00298	hoi110_01234	hoi111_01164	hoi112_01682	hoi113_01443	hoi114_01327	hoi115_01531	hoi116_01234	hoi105_01570	hoi107_01596	hoi109_01528	hoi117_01929	hoi106_01169
1203	alpha/beta hydrolase fold protein	16	Core		hoi102_01234	hoi103_01246	hoi104_01254	hoi108_00299	hoi110_01235	hoi111_01165	hoi112_01681	hoi113_01444	hoi114_01328	hoi115_01532	hoi116_01235	hoi105_01569	hoi107_01595	hoi109_01527	hoi117_01928	hoi106_01168
1204	27 kDa antigen Cfp30B	16	Core		hoi102_01235	hoi103_01247	hoi104_01255	hoi108_00300	hoi110_01236	hoi111_01166	hoi112_01680	hoi113_01445	hoi114_01329	hoi115_01533	hoi116_01236	hoi105_01568	hoi107_01594	hoi109_01526	hoi117_01927	hoi106_01167
1205	ribosome-associated protein	16	Core		hoi102_01236	hoi103_01248	hoi104_01256	hoi108_00301	hoi110_01237	hoi111_01167	hoi112_01679	hoi113_01446	hoi114_01330	hoi115_01534	hoi116_01237	hoi105_01567	hoi107_01593	hoi109_01525	hoi117_01926	hoi106_01166
1206	Threonine efflux protein	16	Core		hoi102_01237	hoi103_01249	hoi104_01257	hoi108_00302	hoi110_01238	hoi111_01168	hoi112_01678	hoi113_01447	hoi114_01331	hoi115_01535	hoi116_01238	hoi105_01566	hoi107_01592	hoi109_01524	hoi117_01925	hoi106_01165
1207	NADP-dependent 3-hydroxy acid dehydrogenase	16	Core		hoi102_01238	hoi103_01250	hoi104_01258	hoi108_00303	hoi110_01239	hoi111_01169	hoi112_01677	hoi113_01448	hoi114_01332	hoi115_01536	hoi116_01239	hoi105_01565	hoi107_01591	hoi109_01523	hoi117_01924	hoi106_01164
1208	Alpha/beta hydrolase family protein	16	Core		hoi102_01239	hoi103_01251	hoi104_01259	hoi108_00304	hoi110_01240	hoi111_01170	hoi112_01676	hoi113_01449	hoi114_01333	hoi115_01537	hoi116_01240	hoi105_01564	hoi107_01590	hoi109_01522	hoi117_01923	hoi106_01163
1209	DNA-3-methyladenine glycosylase 1	16	Core		hoi102_01240	hoi103_01252	hoi104_01260	hoi108_00305	hoi110_01241	hoi111_01171	hoi112_01675	hoi113_01450	hoi114_01334	hoi115_01538	hoi116_01241	hoi105_01563	hoi107_01589	hoi109_01521	hoi117_01922	hoi106_01162
1210	Galactoside O-acetyltransferase	16	Core		hoi102_01241	hoi103_01253	hoi104_01261	hoi108_00306	hoi110_01242	hoi111_01172	hoi112_01674	hoi113_01451	hoi114_01335	hoi115_01539	hoi116_01242	hoi105_01562	hoi107_01588	hoi109_01520	hoi117_01921	hoi106_01161
1211	hypothetical protein	16	Core		hoi102_01242	hoi103_01254	hoi104_01262	hoi108_00307	hoi110_01243	hoi111_01173	hoi112_01673	hoi113_01452	hoi114_01336	hoi115_01540	hoi116_01243	hoi105_01561	hoi107_01587	hoi109_01519	hoi117_01920	hoi106_01160
1212	ATP-dependent RNA helicase HrpB	16	Core		hoi102_01243	hoi103_01255	hoi104_01263	hoi108_00308	hoi110_01244	hoi111_01174	hoi112_01672	hoi113_01453	hoi114_01337	hoi115_01541	hoi116_01244	hoi105_01560	hoi107_01586	hoi109_01518	hoi117_01919	hoi106_01159
1213	hypothetical protein	11	Accessory		hoi102_01244	hoi103_01256	hoi104_01264	hoi108_00309	hoi110_01245	hoi111_01175	hoi112_01671	hoi113_01454	hoi114_01338	hoi115_01542	hoi116_01245					
1214	Copper-sensing transcriptional repressor CsoR	16	Core		hoi102_01245	hoi103_01257	hoi104_01265	hoi108_00310	hoi110_01246	hoi111_01176	hoi112_01670	hoi113_01455	hoi114_01339	hoi115_01543	hoi116_01246	hoi105_01557	hoi107_01583	hoi109_01515	hoi117_01916	hoi106_01156
1215	Putative ammonia monooxygenase	16	Core		hoi102_01246	hoi103_01258	hoi104_01266	hoi108_00311	hoi110_01247	hoi111_01177	hoi112_01669	hoi113_01456	hoi114_01340	hoi115_01544	hoi116_01247	hoi105_01556	hoi107_01582	hoi109_01514	hoi117_01915	hoi106_01155
1216	Carbon starvation protein A	16	Core		hoi102_01247	hoi103_01259	hoi104_01267	hoi108_00312	hoi110_01248	hoi111_01178	hoi112_01668	hoi113_01457	hoi114_01341	hoi115_01545	hoi116_01248	hoi105_01555	hoi107_01581	hoi109_01513	hoi117_01914	hoi106_01154
1217	hypothetical protein	16	Core		hoi102_01248	hoi103_01260	hoi104_01268	hoi108_00313	hoi110_01249	hoi111_01179	hoi112_01667	hoi113_01458	hoi114_01342	hoi115_01546	hoi116_01249	hoi105_01554	hoi107_01580	hoi109_01512	hoi117_01913	hoi106_01153
1218	Arsenical pump-driving ATPase	16	Core		hoi102_01249	hoi103_01261	hoi104_01269	hoi108_00314	hoi110_01250	hoi111_01180	hoi112_01666	hoi113_01459	hoi114_01343	hoi115_01547	hoi116_01250	hoi105_01553	hoi107_01579	hoi109_01511	hoi117_01912	hoi106_01152
1219	NAD-dependent protein deacylase	16	Core		hoi102_01250	hoi103_01262	hoi104_01270	hoi108_00315	hoi110_01251	hoi111_01181	hoi112_01665	hoi113_01460	hoi114_01344	hoi115_01548	hoi116_01251	hoi105_01552	hoi107_01578	hoi109_01510	hoi117_01911	hoi106_01151
1220	hypothetical protein	16	Core		hoi102_01251	hoi103_01263	hoi104_01271	hoi108_00316	hoi110_01252	hoi111_01182	hoi112_01664	hoi113_01461	hoi114_01345	hoi115_01549	hoi116_01252	hoi105_01551	hoi107_01577	hoi109_01509	hoi117_01910	hoi106_01150
1221	2,3,4,5-tetrahydropyridine-2,6-dicarboxylate N-a	16	Core		hoi102_01252	hoi103_01264	hoi104_01272	hoi108_00317	hoi110_01253	hoi111_01183	hoi112_01663	hoi113_01462	hoi114_01346	hoi115_01550	hoi116_01253	hoi105_01550	hoi107_01576	hoi109_01508	hoi117_01909	hoi106_01149
1222	S-ribosylhomocysteine lyase	16	Core		hoi102_01253	hoi103_01265	hoi104_01273	hoi108_00318	hoi110_01254	hoi111_01184	hoi112_01662	hoi113_01463	hoi114_01347	hoi115_01551	hoi116_01254	hoi105_01549	hoi107_01575	hoi109_01507	hoi117_01908	hoi106_01148
1223	FAD-containing monooxygenase EthA	16	Core		hoi102_01254	hoi103_01266	hoi104_01274	hoi108_00319	hoi110_01255	hoi111_01185	hoi112_01661	hoi113_01464	hoi114_01348	hoi115_01552	hoi116_01255	hoi105_01548	hoi107_01574	hoi109_01506	hoi117_01907	hoi106_01147
1224	AMP nucleosidase	16	Core		hoi102_01255	hoi103_01267	hoi104_01275	hoi108_00320	hoi110_01256	hoi111_01186	hoi112_01660	hoi113_01465	hoi114_01349	hoi115_01553	hoi116_01256	hoi105_01547	hoi107_01573	hoi109_01505	hoi117_01906	hoi106_01146
1225	Tripeptidyl aminopeptidase precursor	16	Core		hoi102_01256	hoi103_01268	hoi104_01276	hoi108_00321	hoi110_01257	hoi111_01187	hoi112_01659	hoi113_01466	hoi114_01350	hoi115_01554	hoi116_01257	hoi105_01546	hoi107_01572	hoi109_01504	hoi117_01905	hoi106_01145
1226	hypothetical protein	16	Core		hoi102_01257	hoi103_01269	hoi104_01277	hoi108_00322	hoi110_01258	hoi111_01188	hoi112_01658	hoi113_01467	hoi114_01351	hoi115_01555	hoi116_01258	hoi105_01545	hoi107_01571	hoi109_01503	hoi117_01904	hoi106_01144
1227	hypothetical protein	12	Accessory		hoi102_01258	hoi103_01270	hoi104_01278	hoi108_00323	hoi110_01259	hoi111_01189	hoi112_01657	hoi113_01468	hoi114_01352	hoi115_01556	hoi116_01259					hoi106_01143
1228	FHA domain-containing protein FhaA	16	Core		hoi102_01260	hoi103_01272	hoi104_01280	hoi108_00325	hoi110_01261	hoi111_01191	hoi112_01655	hoi113_01470	hoi114_01354	hoi115_01558	hoi116_01261	hoi105_01535	hoi107_01562	hoi109_01493	hoi117_01894	hoi106_01141
1229	FHA domain-containing protein FhaB	16	Core		hoi102_01261	hoi103_01273	hoi104_01281	hoi108_00326	hoi110_01262	hoi111_01192	hoi112_01654	hoi113_01471	hoi114_01355	hoi115_01559	hoi116_01262	hoi105_01534	hoi107_01561	hoi109_01492	hoi117_01893	hoi106_01140
1230	PP2C-family Ser/Thr phosphatase	16	Core		hoi102_01262	hoi103_01274	hoi104_01282	hoi108_00327	hoi110_01263	hoi111_01193	hoi112_01653	hoi113_01472	hoi114_01356	hoi115_01560	hoi116_01263	hoi105_01533	hoi107_01560	hoi109_01491	hoi117_01892	hoi106_01139
1231	Lipid II flippase FtsW	16	Core		hoi102_01263	hoi103_01275	hoi104_01283	hoi108_00328	hoi110_01264	hoi111_01194	hoi112_01652	hoi113_01473	hoi114_01357	hoi115_01561	hoi116_01264	hoi105_01532	hoi107_01559	hoi109_01490	hoi117_01891	hoi106_01138
1232	Penicillin-binding protein A	16	Core		hoi102_01264	hoi103_01276	hoi104_01284	hoi108_00329	hoi110_01265	hoi111_01195	hoi112_01651	hoi113_01474	hoi114_01358	hoi115_01562	hoi116_01265	hoi105_01531	hoi107_01558	hoi109_01489	hoi117_01890	hoi106_01137
1233	Serine/threonine-protein kinase PknA	16	Core		hoi102_01265	hoi103_01277	hoi104_01285	hoi108_00330	hoi110_01266	hoi111_01196	hoi112_01650	hoi113_01475	hoi114_01359	hoi115_01563	hoi116_01266	hoi105_01530	hoi107_01557	hoi109_01488	hoi117_01889	hoi106_01136
1234	Serine/threonine-protein kinase PknB	16	Core		hoi102_01266	hoi103_01278	hoi104_01286	hoi108_00331	hoi110_01267	hoi111_01197	hoi112_01649	hoi113_01476	hoi114_01360	hoi115_01564	hoi116_01267	hoi105_01529	hoi107_01556	hoi109_01487	hoi117_01888	hoi106_01135
1235	Cell division protein CrgA	16	Core		hoi102_01267	hoi103_01279	hoi104_01287	hoi108_00332	hoi110_01268	hoi111_01198	hoi112_01648	hoi113_01477	hoi114_01361	hoi115_01565	hoi116_01268	hoi105_01528	hoi107_01555	hoi109_01486	hoi117_01887	hoi106_01134
1236	Rhomboid family protein	16	Core		hoi102_01268	hoi103_01280	hoi104_01288	hoi108_00333	hoi110_01269	hoi111_01199	hoi112_01647	hoi113_01478	hoi114_01362	hoi115_01566	hoi116_01269	hoi105_01527	hoi107_01554	hoi109_01485	hoi117_01886	hoi106_01133
1237	Peptidyl-prolyl cis-trans isomerase A	16	Core		hoi102_01269	hoi103_01281	hoi104_01289	hoi108_00334	hoi110_01270	hoi111_01200	hoi112_01646	hoi113_01479	hoi114_01363	hoi115_01567	hoi116_01270	hoi105_01526	hoi107_01553	hoi109_01484	hoi117_01885	hoi106_01132
1238	hypothetical protein	16	Core		hoi102_01270	hoi103_01282	hoi104_01290	hoi108_00335	hoi110_01271	hoi111_01201	hoi112_01645	hoi113_01480	hoi114_01364	hoi115_01568	hoi116_01271	hoi105_01525	hoi107_01552	hoi109_01483	hoi117_01884	hoi106_01131
1239	OsmC-like protein	11	Accessory		hoi102_01271	hoi103_01283	hoi104_01291	hoi108_00336	hoi110_01272	hoi111_01202	hoi112_01644	hoi113_01481	hoi114_01365	hoi115_01569	hoi116_01272					
1240	hypothetical protein	11	Accessory		hoi102_01272	hoi103_01284	hoi104_01292	hoi108_00337	hoi110_01273	hoi111_01203	hoi112_01643	hoi113_01482	hoi114_01366	hoi115_01570	hoi116_01274					
1241	4-nitrophenol 4-monooxygenase/4-nitrocatecho	11	Accessory		hoi102_01273	hoi103_01285	hoi104_01293	hoi108_00338	hoi110_01274	hoi111_01204	hoi112_01642	hoi113_01483	hoi114_01367	hoi115_01571	hoi116_01275					
1242	Bifunctional NAD(P)H-hydrate repair enzyme Nn	16	Core		hoi102_01274	hoi103_01286	hoi104_01294	hoi108_00339	hoi110_01275	hoi111_01205	hoi112_01641	hoi113_01484	hoi114_01368	hoi115_01572	hoi116_01276	hoi105_01524	hoi107_01551	hoi109_01482	hoi117_01883	hoi106_01130
1243	PRC-barrel domain protein	16	Core		hoi102_01275	hoi103_01287	hoi104_01295	hoi108_00340	hoi110_01276	hoi111_01206	hoi112_01640	hoi113_01485	hoi114_01369	hoi115_01573	hoi116_01277	hoi105_01523	hoi107_01550	hoi109_01481	hoi117_01882	hoi106_01129
1244	hypothetical protein	16	Core		hoi102_01278	hoi103_01290	hoi104_01298	hoi108_00343	hoi110_01279	hoi111_01209	hoi112_01637	hoi113_01488	hoi114_01372	hoi115_01576	hoi116_01280	hoi105_01520	hoi107_01547	hoi109_01478	hoi117_01879	hoi106_01126
1245	DNA gyrase subunit A	16	Core		hoi102_01279	hoi103_01291	hoi104_01299	hoi108_00344	hoi110_01280	hoi111_01210	hoi112_01636	hoi113_01489	hoi114_01373	hoi115_01577	hoi116_01281	hoi105_01519	hoi107_01546	hoi109_01477	hoi117_01878	hoi106_01125
1246	hypothetical protein	16	Core		hoi102_01280	hoi103_01292	hoi104_01300	hoi108_00345	hoi110_01281	hoi111_01211	hoi112_01635	hoi113_01490	hoi114_01374	hoi115_01578	hoi116_01282	hoi105_01518	hoi107_01545	hoi109_01476	hoi117_01877	hoi106_01124
1247	DNA gyrase subunit B	16	Core		hoi102_01281	hoi103_01293	hoi104_01301	hoi108_00346	hoi110_01282	hoi111_01212	hoi112_01634	hoi113_01491	hoi114_01375	hoi115_01579	hoi116_01283	hoi105_01517	hoi107_01544	hoi109_01475	hoi117_01876	hoi106_01123
1248	hypothetical protein	16	Core		hoi102_01282	hoi103_01294	hoi104_01302	hoi108_00347	hoi110_01283	hoi111_01213	hoi112_01633	hoi113_01492	hoi114_01376	hoi115_01580	hoi116_01284	hoi105_01516	hoi107_01543	hoi109_01474	hoi117_01875	hoi106_01122
1249	DNA replication and repair protein RecF	16	Core		hoi102_01283	hoi103_01295	hoi104_01303	hoi108_00348	hoi110_01284	hoi111_01214	hoi112_01632	hoi113_01493	hoi114_01377	hoi115_01581	hoi116_01285	hoi105_01515	hoi107_01542	hoi109_01473	hoi117_01874	hoi106_01121
1250	DNA polymerase III subunit beta	16	Core		hoi102_01284	hoi103_01296	hoi104_01304	hoi108_00349	hoi110_01285	hoi111_01215	hoi112_01631	hoi113_01494	hoi114_01378	hoi115_01582	hoi116_01286	hoi105_01514	hoi107_01541	hoi109_01472	hoi117_01873	hoi106_01120
1251	Chromosomal replication initiator protein DnaA	16	Core		hoi102_01285	hoi103_01297	hoi104_01305	hoi108_00350	hoi110_01286	hoi111_01216	hoi112_01630	hoi113_01495	hoi114_01379	hoi115_01583	hoi116_01287	hoi105_01513	hoi107_01540	hoi109_01471	hoi117_01872	hoi106_01119
1252	50S ribosomal protein L34	16	Core		hoi102_01286	hoi103_01298	hoi104_01306	hoi108_00351	hoi110_01287	hoi111_01217	hoi112_01629	hoi113_01496	hoi114_01380	hoi115_01584	hoi116_01288	hoi105_01512	hoi107_01539	hoi109_01470	hoi117_01871	hoi106_01118
1253	ribonuclease P	16	Core		hoi102_01287	hoi103_01299	hoi104_01307	hoi108_00352	hoi110_01288	hoi111_01218	hoi112_01628	hoi113_01497	hoi114_01381	hoi115_01585	hoi116_01289	hoi105_01511	hoi107_01538	hoi109_01469	hoi117_01870	hoi106_01117
1254	Putative membrane protein insertion efficiency f	7	Accessory		hoi102_01288	hoi103_01300			hoi110_01289		hoi112_01627	hoi113_01498	hoi114_01382		hoi116_01290					
1255	Membrane protein insertase MisCA precursor	16	Core		hoi102_01289	hoi103_01301	hoi104_01308	hoi108_00353	hoi110_01290	hoi111_01219	hoi112_01626	hoi113_01499	hoi114_01383	hoi115_01586	hoi116_01291	hoi105_01510	hoi107_01537	hoi109_01468	hoi117_01869	hoi106_01116
1256	Ribosomal RNA small subunit methyltransferase	16	Core		hoi102_01290	hoi103_01302	hoi104_01309	hoi108_00354	hoi110_01291	hoi111_01220	hoi112_01625	hoi113_01500	hoi114_01384	hoi115_01587	hoi116_01292	hoi105_01509	hoi107_01536	hoi109_01467	hoi117_01868	hoi106_01115
1257	Sporulation initiation inhibitor protein Soj	16	Core	Φ_1	hoi102_01291	hoi103_01303	hoi104_01310	hoi108_00355	hoi110_01292	hoi111_01221	hoi112_01624	hoi113_01501	hoi114_01385	hoi115_01588	hoi116_01293	hoi105_01508	hoi107_01535	hoi109_01466	hoi117_01867	hoi106_01114
1258	putative chromosome-partitioning protein ParB	16	Core	Φ_1	hoi102_01292	hoi103_01304	hoi104_01311	hoi108_00356	hoi110_01293	hoi111_01222	hoi112_01623	hoi113_01502	hoi114_01386	hoi115_01589	hoi116_01294	hoi105_01507	hoi107_01534	hoi109_01465	hoi117_01866	hoi106_01113
1259	hypothetical protein	16	Core	Φ_1	hoi102_01293	hoi103_01305	hoi104_01312	hoi108_00357	hoi110_01294	hoi111_01223	hoi112_01622	hoi113_01503	hoi114_01387	hoi115_01590	hoi116_01295	hoi105_01506	hoi107_01533	hoi109_01464	hoi117_01865	hoi106_01112
1260	N-acetylmuramoyl-L-alanine amidase LytC precu	16	Core	Φ_1	hoi102_01294	hoi103_01306	hoi104_01313	hoi108_00358	hoi110_01295	hoi111_01224	hoi112_01621	hoi113_01504	hoi114_01388	hoi115_01591	hoi116_01296	hoi105_01505	hoi107_01532	hoi109_01463	hoi117_01864	hoi106_01111
1261	Thioredoxin	16	Core	Φ_1	hoi102_01295	hoi103_01307	hoi104_01314	hoi108_00359	hoi110_01296	hoi111_01225	hoi112_01620	hoi113_01505	hoi114_01389	hoi115_01592	hoi116_01297	hoi105_01504	hoi107_01531	hoi109_01462	hoi117_01863	hoi106_01110
1262	Thioredoxin reductase	16	Core	Φ_1	hoi102_01296	hoi103_01308	hoi104_01315	hoi108_00360	hoi110_01297	hoi111_01226	hoi112_01619	hoi113_01506	hoi114_01390	hoi115_01593	hoi116_01298	hoi105_01503	hoi107_01530	hoi109_01461	hoi117_01862	hoi106_01109
1263	ECF RNA polymerase sigma factor SigM	16	Core	Φ_1	hoi102_01297	hoi103_01309	hoi104_01316	hoi108_00361	hoi110_01298	hoi111_01227	hoi112_01618	hoi113_01507	hoi114_01391	hoi115_01594	hoi116_01299	hoi105_01502	hoi107_01529	hoi109_01460	hoi117_01861	hoi106_01108
1264	putative peptidoglycan biosynthesis protein Mvi	16	Core		hoi102_01298	hoi103_01310	hoi104_01317	hoi108_00362	hoi110_01299	hoi111_01228	hoi112_01617	hoi113_01508	hoi114_01392	hoi115_01595	hoi116_01300	hoi105_01501	hoi107_01528	hoi109_01459	hoi117_01860	hoi106_01107
1265	hypothetical protein	16	Core		hoi102_01299	hoi103_01311	hoi104_01318	hoi108_00363	hoi110_01300	hoi111_01229	hoi112_01616	hoi113_01509	hoi114_01393	hoi115_01596	hoi116_01301	hoi105_01500	hoi107_01527	hoi109_01458	hoi117_01859	hoi106_01106
1266	Putative mutator protein MutT4	16	Core		hoi102_01300	hoi103_01312	hoi104_01319	hoi108_00364	hoi110_01301	hoi111_01230	hoi112_01615	hoi113_01510	hoi114_01394	hoi115_01597	hoi116_01302	hoi105_01499	hoi107_01526	hoi109_01457	hoi117_01858	hoi106_01105
1267	CCA-adding enzyme	16	Core		hoi102_01301	hoi103_01313	hoi104_01320	hoi108_00365	hoi110_01302	hoi111_01231	hoi112_01614	hoi113_01511	hoi114_01395	hoi115_01598	hoi116_01303	hoi105_01498	hoi107_01525	hoi109_01456	hoi117_01857	hoi106_01104
1268	hypothetical protein	16	Core		hoi102_01302	hoi103_01314	hoi104_01321	hoi108_00366	hoi110_01303	hoi111_01232	hoi112_01613	hoi113_01512	hoi114_01396	hoi115_01599	hoi116_01304	hoi105_01497	hoi107_01524	hoi109_01455	hoi117_01856	hoi106_01103
1269	hypothetical protein	16	Core		hoi102_01303	hoi103_01315	hoi104_01322	hoi108_00367	hoi110_01304	hoi111_01233	hoi112_01612	hoi113_01513	hoi114_01397	hoi115_01600	hoi116_01305	hoi105_01496	hoi107_01523	hoi109_01454	hoi117_01855	hoi106_01102
1270	Sodium Bile acid symporter family protein	16	Core		hoi102_01304	hoi103_01316	hoi104_01323	hoi108_00368	hoi110_01305	hoi111_01234	hoi112_01611	hoi113_01514	hoi114_01398	hoi115_01601	hoi116_01306	hoi105_01495	hoi107_01522	hoi109_01453	hoi117_01854	hoi106_01101
1271	Tryptophan synthase alpha chain	16	Core		hoi102_01305	hoi103_01317	hoi104_01324	hoi108_00369	hoi110_01306	hoi111_01235	hoi112_01610	hoi113_01515	hoi114_01399	hoi115_01602	hoi116_01307	hoi105_01494	hoi107_01521	hoi109_01452	hoi117_01853	hoi106_01100
1272	Tryptophan synthase beta chain	16	Core		hoi102_01306	hoi103_01318	hoi104_01325	hoi108_00370	hoi110_01307	hoi111_01236	hoi112_01609	hoi113_01516	hoi114_01400	hoi115_01603	hoi116_01308	hoi105_01493	hoi107_01520	hoi109_01451	hoi117_01852	hoi106_01099
1273	Tryptophan biosynthesis protein TrpCF	16	Core		hoi102_01307	hoi103_01319	hoi104_01326	hoi108_00371	hoi110_01308	hoi111_01237	hoi112_01608	hoi113_01517	hoi114_01401	hoi115_01604	hoi116_01309	hoi105_01492	hoi107_01519	hoi109_01450	hoi117_01851	hoi106_01098
1274	Anthranilate phosphoribosyltransferase	16	Core		hoi102_01308	hoi103_01320	hoi104_01327	hoi108_00372	hoi110_01309	hoi111_01238	hoi112_01607	hoi113_01518	hoi114_01402	hoi115_01605	hoi116_01310	hoi105_01491	hoi107_01518	hoi109_01449	hoi117_01850	hoi106_01097
1275	Anthranilate synthase component 2	16	Core		hoi102_01309	hoi103_01321	hoi104_01328	hoi108_00373	hoi110_01310	hoi111_01239	hoi112_01606	hoi113_01519	hoi114_01403	hoi115_01606	hoi116_01311	hoi105_01490	hoi107_01517	hoi109_01448	hoi117_01849	hoi106_01096
1276	Anthranilate synthase component 1	16	Core		hoi102_01310	hoi103_01322	hoi104_01329	hoi108_00374	hoi110_01311	hoi111_01240	hoi112_01605	hoi113_01520	hoi114_01404	hoi115_01607	hoi116_01312	hoi105_01489	hoi107_01516	hoi109_01447	hoi117_01848	hoi106_01095
1277	Zinc-transporting ATPase	16	Core		hoi102_01311	hoi103_01323	hoi104_01330	hoi108_00375	hoi110_01312	hoi111_01241	hoi112_01604	hoi113_01521	hoi114_01405	hoi115_01608	hoi116_01313	hoi105_01488	hoi107_01515	hoi109_01446	hoi117_01847	hoi106_01094

1278	HTH-type transcriptional regulator CmtR	16	Core	hoi102_01312	hoi103_01324	hoi104_01331	hoi108_00376	hoi110_01313	hoi111_01242	hoi112_01603	hoi113_01522	hoi114_01406	hoi115_01609	hoi116_01314	hoi105_01487	hoi107_01514	hoi109_01445	hoi117_01846	hoi106_01093
1279	hypothetical protein	16	Core	hoi102_01313	hoi103_01325	hoi104_01332	hoi108_00377	hoi110_01314	hoi111_01243	hoi112_01602	hoi113_01523	hoi114_01407	hoi115_01610	hoi116_01315	hoi105_01486	hoi107_01513	hoi109_01444	hoi117_01845	hoi106_01092
1280	Serine/threonine transporter SstT	16	Core	hoi102_01314	hoi103_01326	hoi104_01333	hoi108_00378	hoi110_01315	hoi111_01244	hoi112_01601	hoi113_01524	hoi114_01408	hoi115_01611	hoi116_01316	hoi105_01485	hoi107_01512	hoi109_01443	hoi117_01844	hoi106_01091
1281	Leucine--tRNA ligase	16	Core	hoi102_01315	hoi103_01327	hoi104_01334	hoi108_00379	hoi110_01316	hoi111_01245	hoi112_01600	hoi113_01525	hoi114_01409	hoi115_01612	hoi116_01317	hoi105_01484	hoi107_01511	hoi109_01442	hoi117_01843	hoi106_01090
1282	VanZ like family protein	16	Core	hoi102_01316	hoi103_01328	hoi104_01335	hoi108_00380	hoi110_01317	hoi111_01246	hoi112_01599	hoi113_01526	hoi114_01410	hoi115_01613	hoi116_01318	hoi105_01483	hoi107_01510	hoi109_01441	hoi117_01842	hoi106_01089
1283	HTH-type transcriptional regulator TtgR	16	Core	hoi102_01317	hoi103_01329	hoi104_01336	hoi108_00381	hoi110_01318	hoi111_01247	hoi112_01598	hoi113_01527	hoi114_01411	hoi115_01614	hoi116_01319	hoi105_01482	hoi107_01509	hoi109_01440	hoi117_01841	hoi106_01088
1284	PTS-dependent dihydroxyacetone kinase, phospl	16	Core	hoi102_01318	hoi103_01330	hoi104_01337	hoi108_00382	hoi110_01319	hoi111_01248	hoi112_01597	hoi113_01528	hoi114_01412	hoi115_01615	hoi116_01320	hoi105_01481	hoi107_01508	hoi109_01439	hoi117_01840	hoi106_01087
1285	PTS-dependent dihydroxyacetone kinase, ADP-bi	16	Core	hoi102_01319	hoi103_01331	hoi104_01338	hoi108_00383	hoi110_01320	hoi111_01249	hoi112_01596	hoi113_01529	hoi114_01413	hoi115_01616	hoi116_01321	hoi105_01480	hoi107_01507	hoi109_01438	hoi117_01839	hoi106_01086
1286	PTS-dependent dihydroxyacetone kinase, dihydr	16	Core	hoi102_01320	hoi103_01332	hoi104_01339	hoi108_00384	hoi110_01321	hoi111_01250	hoi112_01595	hoi113_01530	hoi114_01414	hoi115_01617	hoi116_01322	hoi105_01479	hoi107_01506	hoi109_01437	hoi117_01838	hoi106_01085
1287	Endonuclease 8 1	16	Core	hoi102_01321	hoi103_01333	hoi104_01340	hoi108_00385	hoi110_01322	hoi111_01251	hoi112_01594	hoi113_01531	hoi114_01415	hoi115_01618	hoi116_01323	hoi105_01478	hoi107_01505	hoi109_01436	hoi117_01837	hoi106_01084
1288	Putative N-acetyl-LL-diaminopimelate aminotran	16	Core	hoi102_01322	hoi103_01334	hoi104_01341	hoi108_00386	hoi110_01323	hoi111_01252	hoi112_01593	hoi113_01532	hoi114_01416	hoi115_01619	hoi116_01324	hoi105_01477	hoi107_01504	hoi109_01435	hoi117_01836	hoi106_01083
1289	hypothetical protein	16	Core	hoi102_01323	hoi103_01335	hoi104_01342	hoi108_00387	hoi110_01324	hoi111_01253	hoi112_01592	hoi113_01533	hoi114_01417	hoi115_01620	hoi116_01325	hoi105_01476	hoi107_01503	hoi109_01434	hoi117_01835	hoi106_01082
1290	hypothetical protein	16	Core	hoi102_01324	hoi103_01336	hoi104_01343	hoi108_00388	hoi110_01325	hoi111_01254	hoi112_01591	hoi113_01534	hoi114_01418	hoi115_01621	hoi116_01326	hoi105_01475	hoi107_01502	hoi109_01433	hoi117_01834	hoi106_01081
1291	putative rhodanese-related sulfurtransferase	16	Core	hoi102_01325	hoi103_01337	hoi104_01344	hoi108_00389	hoi110_01326	hoi111_01255	hoi112_01590	hoi113_01535	hoi114_01419	hoi115_01622	hoi116_01327	hoi105_01474	hoi107_01501	hoi109_01432	hoi117_01833	hoi106_01080
1292	hypothetical protein	16	Core	hoi102_01326	hoi103_01338	hoi104_01345	hoi108_00390	hoi110_01327	hoi111_01256	hoi112_01589	hoi113_01536	hoi114_01420	hoi115_01623	hoi116_01328	hoi105_01473	hoi107_01500	hoi109_01431	hoi117_01832	hoi106_01079
1293	putative oxidoreductase	16	Core	hoi102_01327	hoi103_01339	hoi104_01346	hoi108_00391	hoi110_01328	hoi111_01257	hoi112_01588	hoi113_01537	hoi114_01421	hoi115_01624	hoi116_01329	hoi105_01472	hoi107_01499	hoi109_01430	hoi117_01831	hoi106_01078
1294	Universal stress protein family protein	16	Core	hoi102_01328	hoi103_01340	hoi104_01347	hoi108_00392	hoi110_01329	hoi111_01258	hoi112_01587	hoi113_01538	hoi114_01422	hoi115_01625	hoi116_01330	hoi105_01471	hoi107_01498	hoi109_01429	hoi117_01830	hoi106_01077
1295	putative HTH-type transcriptional regulator YusO	16	Core	hoi102_01329	hoi103_01341	hoi104_01348	hoi108_00393	hoi110_01330	hoi111_01259	hoi112_01586	hoi113_01539	hoi114_01423	hoi115_01626	hoi116_01331	hoi105_01470	hoi107_01497	hoi109_01428	hoi117_01829	hoi106_01076
1296	Inositol-3-phosphate synthase	16	Core	hoi102_01330	hoi103_01342	hoi104_01349	hoi108_00394	hoi110_01331	hoi111_01260	hoi112_01585	hoi113_01540	hoi114_01424	hoi115_01627	hoi116_01332	hoi105_01469	hoi107_01496	hoi109_01427	hoi117_01828	hoi106_01075
1297	hypothetical protein	16	Core	hoi102_01331	hoi103_01343	hoi104_01350	hoi108_00395	hoi110_01332	hoi111_01261	hoi112_01584	hoi113_01541	hoi114_01425	hoi115_01628	hoi116_01333	hoi105_01468	hoi107_01495	hoi109_01426	hoi117_01827	hoi106_01074
1298	Penicillin-binding protein 1A	16	Core	hoi102_01332	hoi103_01344	hoi104_01351	hoi108_00396	hoi110_01333	hoi111_01262	hoi112_01583	hoi113_01542	hoi114_01426	hoi115_01629	hoi116_01334	hoi105_01467	hoi107_01494	hoi109_01425	hoi117_01826	hoi106_01073
1299	hypothetical protein	16	Core	hoi102_01333	hoi103_01345	hoi104_01352	hoi108_00397	hoi110_01334	hoi111_01263	hoi112_01582	hoi113_01543	hoi114_01427	hoi115_01630	hoi116_01335	hoi105_01466	hoi107_01493	hoi109_01424	hoi117_01825	hoi106_01072
1300	30S ribosomal protein S6	16	Core	hoi102_01334	hoi103_01346	hoi104_01353	hoi108_00398	hoi110_01335	hoi111_01264	hoi112_01581	hoi113_01544	hoi114_01428	hoi115_01631	hoi116_01336	hoi105_01465	hoi107_01492	hoi109_01423	hoi117_01824	hoi106_01071
1301	Single-stranded DNA-binding protein	16	Core	hoi102_01335	hoi103_01347	hoi104_01354	hoi108_00399	hoi110_01336	hoi111_01265	hoi112_01580	hoi113_01545	hoi114_01429	hoi115_01632	hoi116_01337	hoi105_01464	hoi107_01491	hoi109_01422	hoi117_01823	hoi106_01070
1302	50S ribosomal protein L9	16	Core	hoi102_01336	hoi103_01348	hoi104_01355	hoi108_00400	hoi110_01337	hoi111_01266	hoi112_01579	hoi113_01546	hoi114_01430	hoi115_01633	hoi116_01338	hoi105_01463	hoi107_01490	hoi109_01421	hoi117_01822	hoi106_01069
1303	Replicative DNA helicase	16	Core	hoi102_01337	hoi103_01349	hoi104_01356	hoi108_00401	hoi110_01338	hoi111_01267	hoi112_01578	hoi113_01547	hoi114_01431	hoi115_01634	hoi116_01339	hoi105_01462	hoi107_01489	hoi109_01420	hoi117_01821	hoi106_01068
1304	HTH-type transcriptional regulator LutR	16	Core	hoi102_01338	hoi103_01350	hoi104_01357	hoi108_00402	hoi110_01339	hoi111_01268	hoi112_01577	hoi113_01548	hoi114_01432	hoi115_01635	hoi116_01340	hoi105_01461	hoi107_01488	hoi109_01419	hoi117_01820	hoi106_01067
1305	Copper-exporting P-type ATPase A	16	Core	hoi102_01339	hoi103_01351	hoi104_01358	hoi108_00403	hoi110_01340	hoi111_01269	hoi112_01576	hoi113_01549	hoi114_01433	hoi115_01636	hoi116_01341	hoi105_01460	hoi107_01487	hoi109_01418	hoi117_01819	hoi106_01066
1306	Copper chaperone CopZ	16	Core	hoi102_01340	hoi103_01352	hoi104_01359	hoi108_00404	hoi110_01341	hoi111_01270	hoi112_01575	hoi113_01550	hoi114_01434	hoi115_01637	hoi116_01342	hoi105_01459	hoi107_01486	hoi109_01417	hoi117_01818	hoi106_01065
1307	Putative thioredoxin-2	16	Core	hoi102_01341	hoi103_01353	hoi104_01360	hoi108_00405	hoi110_01342	hoi111_01271	hoi112_01574	hoi113_01551	hoi114_01435	hoi115_01638	hoi116_01343	hoi105_01458	hoi107_01485	hoi109_01416	hoi117_01817	hoi106_01064
1308	PspA/IM30 family protein	16	Core	hoi102_01342	hoi103_01354	hoi104_01361	hoi108_00406	hoi110_01343	hoi111_01272	hoi112_01573	hoi113_01552	hoi114_01436	hoi115_01639	hoi116_01344	hoi105_01457	hoi107_01484	hoi109_01415	hoi117_01816	hoi106_01063
1309	NYN domain protein	16	Core	hoi102_01343	hoi103_01355	hoi104_01362	hoi108_00407	hoi110_01344	hoi111_01273	hoi112_01572	hoi113_01553	hoi114_01437	hoi115_01640	hoi116_01345	hoi105_01456	hoi107_01483	hoi109_01414	hoi117_01815	hoi106_01062
1310	HTH-type transcriptional repressor YtrA	16	Core	hoi102_01344	hoi103_01356	hoi104_01363	hoi108_00408	hoi110_01345	hoi111_01274	hoi112_01571	hoi113_01554	hoi114_01438	hoi115_01641	hoi116_01346	hoi105_01455	hoi107_01482	hoi109_01413	hoi117_01814	hoi106_01061
1311	ABC transporter ATP-binding protein YtrB	16	Core	hoi102_01345	hoi103_01357	hoi104_01364	hoi108_00409	hoi110_01346	hoi111_01275	hoi112_01570	hoi113_01555	hoi114_01439	hoi115_01642	hoi116_01347	hoi105_01454	hoi107_01481	hoi109_01412	hoi117_01813	hoi106_01060
1312	hypothetical protein	16	Core	hoi102_01346	hoi103_01358	hoi104_01365	hoi108_00410	hoi110_01347	hoi111_01276	hoi112_01569	hoi113_01556	hoi114_01440	hoi115_01643	hoi116_01348	hoi105_01453	hoi107_01480	hoi109_01411	hoi117_01812	hoi106_01059
1313	putative D,D-dipeptide transport system permea	16	Core	hoi102_01347	hoi103_01549	hoi104_01366	hoi108_01077	hoi110_00684	hoi111_00191	hoi112_01228	hoi113_01557	hoi114_00462	hoi115_00763	hoi116_00540	hoi105_01259	hoi107_00900	hoi109_00989	hoi117_01408	hoi106_00920
1314	Glutathione import ATP-binding protein GsiA	16	Core	hoi102_01348	hoi103_01548	hoi104_01367	hoi108_01078	hoi110_00685	hoi111_00190	hoi112_01229	hoi113_01558	hoi114_00463	hoi115_00764	hoi116_00541	hoi105_01258	hoi107_00899	hoi109_00988	hoi117_01407	hoi106_00919
1315	Phospholipase YtpA	16	Core	hoi102_01349	hoi103_01547	hoi104_01368	hoi108_01079	hoi110_00686	hoi111_00189	hoi112_01230	hoi113_01559	hoi114_00464	hoi115_00765	hoi116_00542	hoi105_01257	hoi107_00898	hoi109_00987	hoi117_01406	hoi106_00918
1316	Moderate conductance mechanosensitive chann	16	Core	hoi102_01350	hoi103_01546	hoi104_01369	hoi108_01080	hoi110_00687	hoi111_00188	hoi112_01231	hoi113_01560	hoi114_00465	hoi115_00766	hoi116_00543	hoi105_01256	hoi107_00897	hoi109_00986	hoi117_01405	hoi106_00917
1317	CAAX amino terminal protease self- immunity	16	Core	hoi102_01351	hoi103_01545	hoi104_01370	hoi108_01081	hoi110_00688	hoi111_00187	hoi112_01232	hoi113_01561	hoi114_00466	hoi115_00767	hoi116_00544	hoi105_01255	hoi107_00896	hoi109_00985	hoi117_01404	hoi106_00916
1318	Antilisterial bacteriocin subtilosin biosynthesis pr	16	Core	hoi102_01352	hoi103_01544	hoi104_01371	hoi108_01082	hoi110_00689	hoi111_00186	hoi112_01233	hoi113_01562	hoi114_00467	hoi115_00768	hoi116_00545	hoi105_01254	hoi107_00895	hoi109_00984	hoi117_01403	hoi106_00915
1319	Aminopeptidase N	16	Core	hoi102_01353	hoi103_01543	hoi104_01372	hoi108_01083	hoi110_00690	hoi111_00185	hoi112_01234	hoi113_01563	hoi114_00468	hoi115_00769	hoi116_00546	hoi105_01253	hoi107_00894	hoi109_00983	hoi117_01402	hoi106_00914
1320	DSBA-like thioredoxin domain protein	16	Core	hoi102_01354	hoi103_01542	hoi104_01373	hoi108_01084	hoi110_00691	hoi111_00184	hoi112_01235	hoi113_01564	hoi114_00469	hoi115_00770	hoi116_00547	hoi105_01252	hoi107_00893	hoi109_00982	hoi117_01401	hoi106_00913
1321	hypothetical protein	16	Core	hoi102_01355	hoi103_01541	hoi104_01374	hoi108_01085	hoi110_00692	hoi111_00183	hoi112_01236	hoi113_01565	hoi114_00470	hoi115_00771	hoi116_00548	hoi105_01251	hoi107_00892	hoi109_00981	hoi117_01400	hoi106_00912
1322	Excalibur calcium-binding domain protein	16	Core	hoi102_01356	hoi103_01540	hoi104_01375	hoi108_01086	hoi110_00693	hoi111_00182	hoi112_01237	hoi113_01566	hoi114_00471	hoi115_00772	hoi116_00549	hoi105_01250	hoi107_00891	hoi109_00980	hoi117_01399	hoi106_00911
1323	Ribose-5-phosphate isomerase B	16	Core	hoi102_01357	hoi103_01539	hoi104_01376	hoi108_01087	hoi110_00694	hoi111_00181	hoi112_01238	hoi113_01567	hoi114_00472	hoi115_00773	hoi116_00550	hoi105_01249	hoi107_00890	hoi109_00979	hoi117_01398	hoi106_00910
1324	hypothetical protein	16	Core	hoi102_01358	hoi103_01538	hoi104_01377	hoi108_01088	hoi110_00695	hoi111_00180	hoi112_01239	hoi113_01568	hoi114_00473	hoi115_00774	hoi116_00551	hoi105_01248	hoi107_00889	hoi109_00978	hoi117_01397	hoi106_00909
1325	hypothetical protein	16	Core	hoi102_01359	hoi103_01537	hoi104_01378	hoi108_01089	hoi110_00696	hoi111_00179	hoi112_01240	hoi113_01569	hoi114_00474	hoi115_00775	hoi116_00552	hoi105_01247	hoi107_00888	hoi109_00977	hoi117_01396	hoi106_00908
1326	HTH-type transcriptional activator TipA	16	Core	hoi102_01361	hoi103_01535	hoi104_01380	hoi108_01091	hoi110_00698	hoi111_00177	hoi112_01242	hoi113_01571	hoi114_00476	hoi115_00777	hoi116_00554	hoi105_01245	hoi107_00886	hoi109_00975	hoi117_01394	hoi106_00906
1327	putative monoacyl phosphatidylinositol tetramar	16	Core	hoi102_01362	hoi103_01534	hoi104_01381	hoi108_01092	hoi110_00699	hoi111_00176	hoi112_01243	hoi113_01572	hoi114_00477	hoi115_00778	hoi116_00555	hoi105_01244	hoi107_00885	hoi109_00974	hoi117_01393	hoi106_00905
1328	Glutathione import ATP-binding protein GsiA	16	Core	hoi102_01363	hoi103_01533	hoi104_01382	hoi108_01093	hoi110_00700	hoi111_00175	hoi112_01244	hoi113_01573	hoi114_00478	hoi115_00779	hoi116_00556	hoi105_01243	hoi107_00884	hoi109_00973	hoi117_01392	hoi106_00904
1329	Oligopeptide transport system permease protein	16	Core	hoi102_01364	hoi103_01532	hoi104_01383	hoi108_01094	hoi110_00701	hoi111_00174	hoi112_01245	hoi113_01574	hoi114_00479	hoi115_00780	hoi116_00557	hoi105_01242	hoi107_00883	hoi109_00972	hoi117_01391	hoi106_00903
1330	Oligopeptide transport system permease protein	16	Core	hoi102_01365	hoi103_01531	hoi104_01384	hoi108_01095	hoi110_00702	hoi111_00173	hoi112_01246	hoi113_01575	hoi114_00480	hoi115_00781	hoi116_00558	hoi105_01241	hoi107_00882	hoi109_00971	hoi117_01390	hoi106_00902
1331	Trigger factor	16	Core	hoi102_01367	hoi103_01529	hoi104_01386	hoi108_01097	hoi110_00704	hoi111_00171	hoi112_01248	hoi113_01577	hoi114_00482	hoi115_00783	hoi116_00560	hoi105_01239	hoi107_00880	hoi109_00969	hoi117_01388	hoi106_00900
1332	ATP-dependent Clp protease proteolytic subunit	16	Core	hoi102_01368	hoi103_01528	hoi104_01387	hoi108_01098	hoi110_00705	hoi111_00170	hoi112_01249	hoi113_01578	hoi114_00483	hoi115_00784	hoi116_00561	hoi105_01238	hoi107_00879	hoi109_00968	hoi117_01387	hoi106_00899
1333	ATP-dependent Clp protease proteolytic subunit	16	Core	hoi102_01369	hoi103_01527	hoi104_01388	hoi108_01099	hoi110_00706	hoi111_00169	hoi112_01250	hoi113_01579	hoi114_00484	hoi115_00785	hoi116_00562	hoi105_01237	hoi107_00878	hoi109_00967	hoi117_01386	hoi106_00898
1334	hypothetical protein	16	Core	hoi102_01370	hoi103_01526	hoi104_01389	hoi108_01100	hoi110_00707	hoi111_00168	hoi112_01251	hoi113_01580	hoi114_00485	hoi115_00786	hoi116_00563	hoi105_01236	hoi107_00877	hoi109_00966	hoi117_01385	hoi106_00897
1335	ATP-dependent Clp protease ATP-binding subuni	16	Core	hoi102_01371	hoi103_01525	hoi104_01390	hoi108_01101	hoi110_00708	hoi111_00167	hoi112_01252	hoi113_01581	hoi114_00486	hoi115_00787	hoi116_00564	hoi105_01235	hoi107_00876	hoi109_00965	hoi117_01384	hoi106_00896
1336	HTH-type transcriptional repressor NicS	16	Core	hoi102_01372	hoi103_01524	hoi104_01391	hoi108_01102	hoi110_00709	hoi111_00166	hoi112_01253	hoi113_01582	hoi114_00487	hoi115_00788	hoi116_00565	hoi105_01234	hoi107_00875	hoi109_00964	hoi117_01383	hoi106_00895
1337	Malate dehydrogenase	16	Core	hoi102_01373	hoi103_01523	hoi104_01392	hoi108_01103	hoi110_00710	hoi111_00165	hoi112_01254	hoi113_01583	hoi114_00488	hoi115_00789	hoi116_00566	hoi105_01233	hoi107_00874	hoi109_00963	hoi117_01382	hoi106_00894
1338	HTH-type transcriptional regulator CmtR	16	Core	hoi102_01374	hoi103_01522	hoi104_01393	hoi108_01104	hoi110_00711	hoi111_00164	hoi112_01255	hoi113_01584	hoi114_00489	hoi115_00790	hoi116_00567	hoi105_01232	hoi107_00873	hoi109_00962	hoi117_01381	hoi106_00893
1339	Valine--tRNA ligase	16	Core	hoi102_01375	hoi103_01521	hoi104_01394	hoi108_01105	hoi110_00712	hoi111_00163	hoi112_01256	hoi113_01585	hoi114_00490	hoi115_00791	hoi116_00568	hoi105_01231	hoi107_00872	hoi109_00961	hoi117_01380	hoi106_00892
1340	Folylpolyglutamate synthase	16	Core	hoi102_01376	hoi103_01520	hoi104_01395	hoi108_01106	hoi110_00713	hoi111_00162	hoi112_01257	hoi113_01586	hoi114_00491	hoi115_00792	hoi116_00569	hoi105_01230	hoi107_00871	hoi109_00960	hoi117_01379	hoi106_00891
1341	hypothetical protein	16	Core	hoi102_01377	hoi103_01519	hoi104_01396	hoi108_01107	hoi110_00714	hoi111_00161	hoi112_01258	hoi113_01587	hoi114_00492	hoi115_00793	hoi116_00570	hoi105_01229	hoi107_00870	hoi109_00959	hoi117_01378	hoi106_00890
1342	hypothetical protein	16	Core	hoi102_01378	hoi103_01518	hoi104_01397	hoi108_01108	hoi110_00715	hoi111_00160	hoi112_01259	hoi113_01588	hoi114_00493	hoi115_00794	hoi116_00571	hoi105_01228	hoi107_00869	hoi109_00958	hoi117_01377	hoi106_00889
1343	hypothetical protein	16	Core	hoi102_01379	hoi103_01517	hoi104_01398	hoi108_01109	hoi110_00716	hoi111_00159	hoi112_01260	hoi113_01589	hoi114_00494	hoi115_00795	hoi116_00572	hoi105_01227	hoi107_00868	hoi109_00957	hoi117_01376	hoi106_00888
1344	Nucleoside diphosphate kinase	16	Core	hoi102_01380	hoi103_01516	hoi104_01399	hoi108_01110	hoi110_00717	hoi111_00158	hoi112_01261	hoi113_01590	hoi114_00495	hoi115_00796	hoi116_00573	hoi105_01226	hoi107_00867	hoi109_00956	hoi117_01375	hoi106_00887
1345	NAD(P) transhydrogenase subunit beta	16	Core	hoi102_01381	hoi103_01515	hoi104_01400	hoi108_01111	hoi110_00718	hoi111_00157	hoi112_01262	hoi113_01591	hoi114_00496	hoi115_00797	hoi116_00574	hoi105_01225	hoi107_00866	hoi109_00955	hoi117_01374	hoi106_00886
1346	NAD(P) transhydrogenase subunit alpha	16	Core	hoi102_01382	hoi103_01514	hoi104_01401	hoi108_01112	hoi110_00719	hoi111_00156	hoi112_01263	hoi113_01592	hoi114_00497	hoi115_00798	hoi116_00575	hoi105_01224	hoi107_00865	hoi109_00954	hoi117_01373	hoi106_00885
1347	Ribonuclease E	16	Core	hoi102_01383	hoi103_01513	hoi104_01402	hoi108_01113	hoi110_00720	hoi111_00155	hoi112_01264	hoi113_01593	hoi114_00498	hoi115_00799	hoi116_00576	hoi105_01223	hoi107_00864	hoi109_00953	hoi117_01372	hoi106_00884
1348	50S ribosomal protein L21	16	Core	hoi102_01384	hoi103_01512	hoi104_01403	hoi108_01114	hoi110_00721	hoi111_00154	hoi112_01265	hoi113_01594	hoi114_00499	hoi115_00800	hoi116_00577	hoi105_01222	hoi107_00863	hoi109_00952	hoi117_01371	hoi106_00883
1349	50S ribosomal protein L27	16	Core	hoi102_01385	hoi103_01511	hoi104_01404	hoi108_01115	hoi110_00722	hoi111_00153	hoi112_01266	hoi113_01595	hoi114_00500	hoi115_00801	hoi116_00578	hoi105_01221	hoi107_00862	hoi109_00951	hoi117_01370	hoi106_00882
1350	GTPase Obg	16	Core	hoi102_01386	hoi103_01510	hoi104_01405	hoi108_01116	hoi110_00723	hoi111_00152	hoi112_01267	hoi113_01596	hoi114_00501	hoi115_00802	hoi116_00579	hoi105_01220	hoi107_00861	hoi109_00950	hoi117_01369	hoi106_00881
1351	Glutamate 5-kinase	16	Core	hoi102_01387	hoi103_01509	hoi104_01406	hoi108_01117	hoi110_00724	hoi111_00151	hoi112_01268	hoi113_01597	hoi114_00502	hoi115_00803	hoi116_00580	hoi105_01219	hoi107_00860	hoi109_00949	hoi117_01368	hoi106_00880
1352	Glycerate dehydrogenase	16	Core	hoi102_01388	hoi103_01508	hoi104_01407	hoi108_01118	hoi110_00725	hoi111_00150	hoi112_01269	hoi113_01598	hoi114_00503	hoi115_00804	hoi116_00581	hoi105_01218	hoi107_00859	hoi109_00948	hoi117_01367	hoi106_00879
1353	Gamma-glutamyl phosphate reductase	16	Core	hoi102_01389	hoi103_01507	hoi104_01408	hoi108_01119	hoi110_00726	hoi111_00149	hoi112_01270	hoi113_01599	hoi114_00504	hoi115_00805	hoi116_00582	hoi105_01217	hoi107_00858	hoi109_00947	hoi117_01366	hoi106_00878
1354	Nicotinate-nucleotide adenylyltransferase	16	Core	hoi102_01390	hoi103_01506	hoi104_01409	hoi108_01120	hoi110_00727	hoi111_00148	hoi112_01271	hoi113_01600	hoi114_00505	hoi115_00806	hoi116_00583	hoi105_01216	hoi107_00857	hoi109_00946	hoi117_01365	hoi106_00877
1355	Ribosomal silencing factor RsfS	16	Core	hoi102_01391	hoi103_01505	hoi104_01410	hoi108_01121	hoi110_00728	hoi111_00147	hoi112_01272	hoi113_01601	hoi114_00506	hoi115_00807	hoi116_00584	hoi105_01215	hoi107_00856	hoi109_00945	hoi117_01364	hoi106_00876
1356	Glucosyl-3-phosphoglycerate phosphatase	16	Core	hoi102_01392	hoi103_01504	hoi104_01411	hoi108_01122	hoi110_00729	hoi111_00146	hoi112_01273	hoi113_01602	hoi114_00507	hoi115_00808	hoi116_00585	hoi105_01214	hoi107_00855	hoi109_00944	hoi117_01363	hoi106_00875
1357	DegV domain-containing protein/cg2579	16	Core	hoi102_01393	hoi103_01503	hoi104_01412	hoi108_01123	hoi110_00730	hoi111_00145	hoi112_01274	hoi113_01603	hoi114_00508	hoi115_00809	hoi116_00586	hoi105_01213	hoi107_00854	hoi109_00943	hoi117_01362	hoi106_00874

1358	ComE operon protein 1	16	Core		hoi102_01394	hoi103_01502	hoi104_01413	hoi108_01124	hoi110_00731	hoi111_00144	hoi112_01275	hoi113_01604	hoi114_00509	hoi115_00810	hoi116_00587	hoi105_01212	hoi107_00853	hoi109_00942	hoi117_01361	hoi106_00873
1359	Competence protein	16	Core		hoi102_01395	hoi103_01501	hoi104_01414	hoi108_01125	hoi110_00732	hoi111_00143	hoi112_01276	hoi113_01605	hoi114_00510	hoi115_00811	hoi116_00588	hoi105_01211	hoi107_00852	hoi109_00941	hoi117_01360	hoi106_00872
1360	CRISPR associated protein	16	Core	GI7	hoi102_01396	hoi103_01500	hoi104_01415	hoi108_01126	hoi110_00733	hoi111_00142	hoi112_01277	hoi113_01606	hoi114_00511	hoi115_00812	hoi116_00589	hoi105_01210	hoi107_00851	hoi109_00940	hoi117_01359	hoi106_00871
1361	hypothetical protein	16	Core	GI7	hoi102_01397	hoi103_01499	hoi104_01416	hoi108_01127	hoi110_00734	hoi111_00141	hoi112_01278	hoi113_01607	hoi114_00512	hoi115_00813	hoi116_00590	hoi105_01209	hoi107_00850	hoi109_00939	hoi117_01358	hoi106_00870
1362	CRISPR-associated protein, family (Cas_GSU0054	16	Core	GI7	hoi102_01398	hoi103_01498	hoi104_01417	hoi108_01128	hoi110_00735	hoi111_00140	hoi112_01279	hoi113_01608	hoi114_00513	hoi115_00814	hoi116_00591	hoi105_01208	hoi107_00849	hoi109_00938	hoi117_01357	hoi106_00869
1363	helicase Cas3	16	Core	GI7	hoi102_01399	hoi103_01497	hoi104_01418	hoi108_01129	hoi110_00736	hoi111_00139	hoi112_01280	hoi113_01609	hoi114_00514	hoi115_00815	hoi116_00592	hoi105_01207	hoi107_00848	hoi109_00937	hoi117_01356	hoi106_00868
1364	hypothetical protein	16	Core		hoi102_01400	hoi103_01496	hoi104_01419	hoi108_01130	hoi110_00737	hoi111_00138	hoi112_01281	hoi113_01610	hoi114_00515	hoi115_00816	hoi116_00593	hoi105_01206	hoi107_00847	hoi109_00936	hoi117_01355	hoi106_00867
1365	30S ribosomal protein S20	16	Core		hoi102_01401	hoi103_01495	hoi104_01420	hoi108_01131	hoi110_00738	hoi111_00137	hoi112_01282	hoi113_01611	hoi114_00516	hoi115_00817	hoi116_00594	hoi105_01205	hoi107_00846	hoi109_00935	hoi117_01354	hoi106_00866
1366	PemK-like protein	16	Core		hoi102_01402	hoi103_01494	hoi104_01421	hoi108_01132	hoi110_00739	hoi111_00136	hoi112_01283	hoi113_01612	hoi114_00517	hoi115_00818	hoi116_00595	hoi105_01204	hoi107_00845	hoi109_00934	hoi117_01353	hoi106_00865
1367	Elongation factor 4	16	Core		hoi102_01403	hoi103_01493	hoi104_01422	hoi108_01133	hoi110_00740	hoi111_00135	hoi112_01284	hoi113_01613	hoi114_00518	hoi115_00819	hoi116_00596	hoi105_01203	hoi107_00844	hoi109_00933	hoi117_01352	hoi106_00864
1368	hypothetical protein	16	Core		hoi102_01404	hoi103_01492	hoi104_01423	hoi108_01134	hoi110_00741	hoi111_00134	hoi112_01285	hoi113_01614	hoi114_00519	hoi115_00820	hoi116_00597	hoi105_01202	hoi107_00843	hoi109_00932	hoi117_01351	hoi106_00863
1369	hypothetical protein	16	Core		hoi102_01405	hoi103_01491	hoi104_01424	hoi108_01135	hoi110_00742	hoi111_00133	hoi112_01286	hoi113_01615	hoi114_00520	hoi115_00821	hoi116_00598	hoi105_01201	hoi107_00842	hoi109_00931	hoi117_01350	hoi106_00862
1370	hypothetical protein	16	Core		hoi102_01406	hoi103_01490	hoi104_01425	hoi108_01136	hoi110_00743	hoi111_00132	hoi112_01287	hoi113_01616	hoi114_00521	hoi115_00822	hoi116_00599	hoi105_01200	hoi107_00841	hoi109_00930	hoi117_01349	hoi106_00861
1371	Uric acid transporter UacT	16	Core		hoi102_01407	hoi103_01489	hoi104_01426	hoi108_01137	hoi110_00744	hoi111_00131	hoi112_01288	hoi113_01617	hoi114_00522	hoi115_00823	hoi116_00600	hoi105_01199	hoi107_00840	hoi109_00929	hoi117_01348	hoi106_00860
1372	Biotin synthase	16	Core		hoi102_01408	hoi103_01488	hoi104_01427	hoi108_01138	hoi110_00745	hoi111_00130	hoi112_01289	hoi113_01618	hoi114_00523	hoi115_00824	hoi116_00601	hoi105_01198	hoi107_00839	hoi109_00928	hoi117_01347	hoi106_00859
1373	hypothetical protein	16	Core		hoi102_01409	hoi103_01487	hoi104_01428	hoi108_01139	hoi110_00746	hoi111_00129	hoi112_01290	hoi113_01619	hoi114_00524	hoi115_00825	hoi116_00602	hoi105_01197	hoi107_00838	hoi109_00927	hoi117_01346	hoi106_00858
1374	Ribokinase	16	Core		hoi102_01410	hoi103_01486	hoi104_01429	hoi108_01140	hoi110_00747	hoi111_00128	hoi112_01291	hoi113_01620	hoi114_00525	hoi115_00826	hoi116_00603	hoi105_01196	hoi107_00837	hoi109_00926	hoi117_01345	hoi106_00857
1375	ADP-ribosyl-[dinitrogen reductase] glycohydrolas	16	Core		hoi102_01411	hoi103_01485	hoi104_01430	hoi108_01141	hoi110_00748	hoi111_00127	hoi112_01292	hoi113_01621	hoi114_00526	hoi115_00827	hoi116_00604	hoi105_01195	hoi107_00836	hoi109_00925	hoi117_01344	hoi106_00856
1376	Glutathione import ATP-binding protein GsiA	16	Core		hoi102_01412	hoi103_01484	hoi104_01431	hoi108_01142	hoi110_00749	hoi111_00126	hoi112_01293	hoi113_01622	hoi114_00527	hoi115_00828	hoi116_00605	hoi105_01194	hoi107_00835	hoi109_00924	hoi117_01343	hoi106_00855
1377	Glutathione transport system permease protein	16	Core		hoi102_01413	hoi103_01483	hoi104_01432	hoi108_01143	hoi110_00750	hoi111_00125	hoi112_01294	hoi113_01623	hoi114_00528	hoi115_00829	hoi116_00606	hoi105_01193	hoi107_00834	hoi109_00923	hoi117_01342	hoi106_00854
1378	Dipeptide transport system permease protein Dp	16	Core		hoi102_01414	hoi103_01482	hoi104_01433	hoi108_01144	hoi110_00751	hoi111_00124	hoi112_01295	hoi113_01624	hoi114_00529	hoi115_00830	hoi116_00607	hoi105_01192	hoi107_00833	hoi109_00922	hoi117_01341	hoi106_00853
1379	Nickel-binding periplasmic protein precursor	16	Core		hoi102_01415	hoi103_01481	hoi104_01434	hoi108_01145	hoi110_00752	hoi111_00123	hoi112_01296	hoi113_01625	hoi114_00530	hoi115_00831	hoi116_00608	hoi105_01191	hoi107_00832	hoi109_00921	hoi117_01340	hoi106_00852
1380	hypothetical protein	16	Core		hoi102_01416	hoi103_01480	hoi104_01435	hoi108_01146	hoi110_00753	hoi111_00122	hoi112_01297	hoi113_01626	hoi114_00531	hoi115_00832	hoi116_00609	hoi105_01190	hoi107_00831	hoi109_00920	hoi117_01339	hoi106_00851
1381	Branched-chain amino acid transport system 2 ca	16	Core		hoi102_01417	hoi103_01479	hoi104_01436	hoi108_01147	hoi110_00754	hoi111_00121	hoi112_01298	hoi113_01627	hoi114_00532	hoi115_00833	hoi116_00610	hoi105_01189	hoi107_00830	hoi109_00919	hoi117_01338	hoi106_00850
1382	Cystathionine beta-lyase PatB	16	Core		hoi102_01418	hoi103_01478	hoi104_01437	hoi108_01148	hoi110_00755	hoi111_00120	hoi112_01299	hoi113_01628	hoi114_00533	hoi115_00834	hoi116_00611	hoi105_01188	hoi107_00829	hoi109_00918	hoi117_01337	hoi106_00849
1383	Isopentenyl-diphosphate Delta-isomerase	16	Core		hoi102_01419	hoi103_01477	hoi104_01438	hoi108_01149	hoi110_00756	hoi111_00119	hoi112_01300	hoi113_01629	hoi114_00534	hoi115_00835	hoi116_00612	hoi105_01187	hoi107_00828	hoi109_00917	hoi117_01336	hoi106_00848
1384	Maltokinase	16	Core		hoi102_01420	hoi103_01476	hoi104_01439	hoi108_01150	hoi110_00757	hoi111_00118	hoi112_01301	hoi113_01630	hoi114_00535	hoi115_00836	hoi116_00613	hoi105_01186	hoi107_00827	hoi109_00916	hoi117_01335	hoi106_00847
1385	Trehalose synthase/amylase TreS	16	Core		hoi102_01421	hoi103_01475	hoi104_01440	hoi108_01151	hoi110_00758	hoi111_00117	hoi112_01302	hoi113_01631	hoi114_00536	hoi115_00837	hoi116_00614	hoi105_01185	hoi107_00826	hoi109_00915	hoi117_01334	hoi106_00846
1386	hypothetical protein	16	Core		hoi102_01422	hoi103_01474	hoi104_01441	hoi108_01152	hoi110_00759	hoi111_00116	hoi112_01303	hoi113_01632	hoi114_00537	hoi115_00838	hoi116_00615	hoi105_01184	hoi107_00825	hoi109_00914	hoi117_01333	hoi106_00845
1387	Carboxylesterase	16	Core		hoi102_01423	hoi103_01473	hoi104_01442	hoi108_01153	hoi110_00760	hoi111_00115	hoi112_01304	hoi113_01633	hoi114_00538	hoi115_00839	hoi116_00616	hoi105_01183	hoi107_00824	hoi109_00913	hoi117_01332	hoi106_00844
1388	Peptidyl-dipeptidase dcp	16	Core		hoi102_01424	hoi103_01472	hoi104_01443	hoi108_01154	hoi110_00761	hoi111_00114	hoi112_01305	hoi113_01634	hoi114_00539	hoi115_00840	hoi116_00617	hoi105_01182	hoi107_00823	hoi109_00912	hoi117_01331	hoi106_00843
1389	hypothetical protein	16	Core		hoi102_01425	hoi103_01471	hoi104_01444	hoi108_01155	hoi110_00762	hoi111_00113	hoi112_01306	hoi113_01635	hoi114_00540	hoi115_00841	hoi116_00618	hoi105_01181	hoi107_00822	hoi109_00911	hoi117_01330	hoi106_00842
1390	4-alpha-glucanotransferase	16	Core		hoi102_01427	hoi103_01469	hoi104_01446	hoi108_01157	hoi110_00764	hoi111_00111	hoi112_01308	hoi113_01637	hoi114_00542	hoi115_00843	hoi116_00620	hoi105_01179	hoi107_00820	hoi109_00909	hoi117_01328	hoi106_00840
1391	Long-chain-fatty-acid--CoA ligase FadD15	16	Core		hoi102_01428	hoi103_01468	hoi104_01447	hoi108_01158	hoi110_00765	hoi111_00110	hoi112_01309	hoi113_01638	hoi114_00543	hoi115_00844	hoi116_00621	hoi105_01178	hoi107_00819	hoi109_00908	hoi117_01327	hoi106_00839
1392	hypothetical protein	16	Core		hoi102_01429	hoi103_01467	hoi104_01448	hoi108_01159	hoi110_00766	hoi111_00109	hoi112_01310	hoi113_01639	hoi114_00544	hoi115_00845	hoi116_00622	hoi105_01177	hoi107_00818	hoi109_00907	hoi117_01326	hoi106_00838
1393	Oxygen-independent coproporphyrinogen-III oxi	16	Core		hoi102_01430	hoi103_01466	hoi104_01449	hoi108_01160	hoi110_00767	hoi111_00108	hoi112_01311	hoi113_01640	hoi114_00545	hoi115_00846	hoi116_00623	hoi105_01176	hoi107_00817	hoi109_00906	hoi117_01325	hoi106_00837
1394	Heat-inducible transcription repressor HrcA	16	Core		hoi102_01431	hoi103_01465	hoi104_01450	hoi108_01161	hoi110_00768	hoi111_00107	hoi112_01312	hoi113_01641	hoi114_00546	hoi115_00847	hoi116_00624	hoi105_01175	hoi107_00816	hoi109_00905	hoi117_01324	hoi106_00836
1395	Chaperone protein DnaJ	16	Core		hoi102_01432	hoi103_01464	hoi104_01451	hoi108_01162	hoi110_00769	hoi111_00106	hoi112_01313	hoi113_01642	hoi114_00547	hoi115_00848	hoi116_00625	hoi105_01174	hoi107_00815	hoi109_00904	hoi117_01323	hoi106_00835
1396	Ribosomal RNA small subunit methyltransferase	16	Core		hoi102_01433	hoi103_01463	hoi104_01452	hoi108_01163	hoi110_00770	hoi111_00105	hoi112_01314	hoi113_01643	hoi114_00548	hoi115_00849	hoi116_00626	hoi105_01173	hoi107_00814	hoi109_00903	hoi117_01322	hoi106_00834
1397	PhoH-like protein	16	Core		hoi102_01434	hoi103_01462	hoi104_01453	hoi108_01164	hoi110_00771	hoi111_00104	hoi112_01315	hoi113_01644	hoi114_00549	hoi115_00850	hoi116_00627	hoi105_01172	hoi107_00813	hoi109_00902	hoi117_01321	hoi106_00833
1398	Endoribonuclease YbeY	16	Core		hoi102_01435	hoi103_01461	hoi104_01454	hoi108_01165	hoi110_00772	hoi111_00103	hoi112_01316	hoi113_01645	hoi114_00550	hoi115_00851	hoi116_00628	hoi105_01171	hoi107_00812	hoi109_00901	hoi117_01320	hoi106_00832
1399	Magnesium and cobalt efflux protein CorC	16	Core		hoi102_01436	hoi103_01460	hoi104_01455	hoi108_01166	hoi110_00773	hoi111_00102	hoi112_01317	hoi113_01646	hoi114_00551	hoi115_00852	hoi116_00629	hoi105_01170	hoi107_00811	hoi109_00900	hoi117_01319	hoi106_00831
1400	GTPase Era	16	Core		hoi102_01437	hoi103_01459	hoi104_01456	hoi108_01167	hoi110_00774	hoi111_00101	hoi112_01318	hoi113_01647	hoi114_00552	hoi115_00853	hoi116_00630	hoi105_01169	hoi107_00810	hoi109_00899	hoi117_01318	hoi106_00830
1401	DNA repair protein RecO	16	Core		hoi102_01438	hoi103_01458	hoi104_01457	hoi108_01168	hoi110_00775	hoi111_00100	hoi112_01319	hoi113_01648	hoi114_00553	hoi115_00854	hoi116_00631	hoi105_01168	hoi107_00809	hoi109_00898	hoi117_01317	hoi106_00829
1402	Trans,polycis-polyprenyl diphosphate synthase ((	16	Core		hoi102_01439	hoi103_01457	hoi104_01458	hoi108_01169	hoi110_00776	hoi111_00099	hoi112_01320	hoi113_01649	hoi114_00554	hoi115_00855	hoi116_00632	hoi105_01167	hoi107_00808	hoi109_00897	hoi117_01316	hoi106_00828
1403	VIT family protein	16	Core		hoi102_01440	hoi103_01456	hoi104_01459	hoi108_01170	hoi110_00777	hoi111_00098	hoi112_01321	hoi113_01650	hoi114_00555	hoi115_00856	hoi116_00633	hoi105_01166	hoi107_00807	hoi109_00896	hoi117_01315	hoi106_00827
1404	hypothetical protein	16	Core		hoi102_01441	hoi103_01455	hoi104_01460	hoi108_01171	hoi110_00778	hoi111_00097	hoi112_01322	hoi113_01651	hoi114_00556	hoi115_00857	hoi116_00634	hoi105_01165	hoi107_00806	hoi109_00895	hoi117_01314	hoi106_00826
1405	Zinc uptake regulation protein	16	Core		hoi102_01442	hoi103_01454	hoi104_01461	hoi108_01172	hoi110_00779	hoi111_00096	hoi112_01323	hoi113_01652	hoi114_00557	hoi115_00858	hoi116_00635	hoi105_01164	hoi107_00805	hoi109_00894	hoi117_01313	hoi106_00825
1406	Glycine--tRNA ligase	16	Core		hoi102_01443	hoi103_01453	hoi104_01462	hoi108_01173	hoi110_00780	hoi111_00095	hoi112_01324	hoi113_01653	hoi114_00558	hoi115_00859	hoi116_00636	hoi105_01163	hoi107_00804	hoi109_00893	hoi117_01312	hoi106_00824
1407	hypothetical protein	16	Core		hoi102_01444	hoi103_01452	hoi104_01463	hoi108_01174	hoi110_00781	hoi111_00094	hoi112_01325	hoi113_01654	hoi114_00559	hoi115_00860	hoi116_00637	hoi105_01162	hoi107_00803	hoi109_00892	hoi117_01311	hoi106_00823
1408	hypothetical protein	16	Core		hoi102_01445	hoi103_01451	hoi104_01464	hoi108_01175	hoi110_00782	hoi111_00093	hoi112_01326	hoi113_01655	hoi114_00560	hoi115_00861	hoi116_00638	hoi105_01161	hoi107_00802	hoi109_00891	hoi117_01310	hoi106_00822
1409	hypothetical protein	16	Core		hoi102_01446	hoi103_01450	hoi104_01465	hoi108_01176	hoi110_00783	hoi111_00092	hoi112_01327	hoi113_01656	hoi114_00561	hoi115_00862	hoi116_00639	hoi105_01160	hoi107_00801	hoi109_00890	hoi117_01309	hoi106_00821
1410	hypothetical protein	16	Core		hoi102_01447	hoi103_01449	hoi104_01466	hoi108_01177	hoi110_00784	hoi111_00091	hoi112_01328	hoi113_01657	hoi114_00562	hoi115_00863	hoi116_00640	hoi105_01159	hoi107_00800	hoi109_00889	hoi117_01308	hoi106_00820
1411	Deoxyguanosinetriphosphate triphosphohydrola	16	Core		hoi102_01448	hoi103_01448	hoi104_01467	hoi108_01178	hoi110_00785	hoi111_00090	hoi112_01329	hoi113_01658	hoi114_00563	hoi115_00864	hoi116_00641	hoi105_01158	hoi107_00799	hoi109_00888	hoi117_01307	hoi106_00819
1412	Dehydrosqualene desaturase	16	Core		hoi102_01449	hoi103_01447	hoi104_01468	hoi108_01179	hoi110_00786	hoi111_00089	hoi112_01330	hoi113_01659	hoi114_00564	hoi115_00865	hoi116_00642	hoi105_01157	hoi107_00798	hoi109_00887	hoi117_01306	hoi106_00818
1413	15-cis-phytoene synthase	16	Core		hoi102_01450	hoi103_01446	hoi104_01469	hoi108_01180	hoi110_00787	hoi111_00088	hoi112_01331	hoi113_01660	hoi114_00565	hoi115_00866	hoi116_00643	hoi105_01156	hoi107_00797	hoi109_00886	hoi117_01305	hoi106_00817
1414	hypothetical protein	16	Core		hoi102_01451	hoi103_01445	hoi104_01470	hoi108_01181	hoi110_00788	hoi111_00087	hoi112_01332	hoi113_01661	hoi114_00566	hoi115_00867	hoi116_00644	hoi105_01155	hoi107_00796	hoi109_00885	hoi117_01304	hoi106_00816
1415	Guanyl-specific ribonuclease St	16	Core		hoi102_01452	hoi103_01444	hoi104_01471	hoi108_01182	hoi110_00789	hoi111_00086	hoi112_01333	hoi113_01662	hoi114_00567	hoi115_00868	hoi116_00645	hoi105_01154	hoi107_00795	hoi109_00884	hoi117_01303	hoi106_00815
1416	DNA primase	16	Core		hoi102_01453	hoi103_01443	hoi104_01472	hoi108_01183	hoi110_00790	hoi111_00085	hoi112_01334	hoi113_01663	hoi114_00568	hoi115_00869	hoi116_00646	hoi105_01153	hoi107_00794	hoi109_00883	hoi117_01302	hoi106_00814
1417	hypothetical protein	16	Core		hoi102_01454	hoi103_01442	hoi104_01473	hoi108_01184	hoi110_00791	hoi111_00084	hoi112_01335	hoi113_01664	hoi114_00569	hoi115_00870	hoi116_00647	hoi105_01152	hoi107_00793	hoi109_00882	hoi117_01301	hoi106_00813
1418	Short-chain-fatty-acid--CoA ligase	16	Core		hoi102_01455	hoi103_01441	hoi104_01474	hoi108_01185	hoi110_00792	hoi111_00083	hoi112_01336	hoi113_01665	hoi114_00570	hoi115_00871	hoi116_00648	hoi105_01151	hoi107_00792	hoi109_00881	hoi117_01300	hoi106_00812
1419	hypothetical protein	16	Core		hoi102_01458	hoi103_01438	hoi104_01477	hoi108_01188	hoi110_00795	hoi111_00080	hoi112_01339	hoi113_01668	hoi114_00573	hoi115_00874	hoi116_00651	hoi105_01148	hoi107_00789	hoi109_00878	hoi117_01297	hoi106_00809
1420	Proton glutamate symport protein	16	Core		hoi102_01459	hoi103_01437	hoi104_01478	hoi108_01189	hoi110_00796	hoi111_00079	hoi112_01340	hoi113_01669	hoi114_00574	hoi115_00875	hoi116_00652	hoi105_01147	hoi107_00788	hoi109_00877	hoi117_01296	hoi106_00808
1421	beta-lactamase/D-alanine carboxypeptidase	16	Core		hoi102_01460	hoi103_01436	hoi104_01479	hoi108_01190	hoi110_00797	hoi111_00078	hoi112_01341	hoi113_01670	hoi114_00575	hoi115_00876	hoi116_00653	hoi105_01146	hoi107_00787	hoi109_00876	hoi117_01295	hoi106_00807
1422	hypothetical protein	16	Core		hoi102_01461	hoi103_01435	hoi104_01480	hoi108_01191	hoi110_00798	hoi111_00077	hoi112_01342	hoi113_01671	hoi114_00576	hoi115_00877	hoi116_00654	hoi105_01145	hoi107_00786	hoi109_00875	hoi117_01294	hoi106_00806
1423	Ribonucleotide monophosphatase NagD	16	Core		hoi102_01462	hoi103_01434	hoi104_01481	hoi108_01192	hoi110_00799	hoi111_00076	hoi112_01343	hoi113_01672	hoi114_00577	hoi115_00878	hoi116_00655	hoi105_01144	hoi107_00785	hoi109_00874	hoi117_01293	hoi106_00805
1424	acyl carrier protein	16	Core		hoi102_01463	hoi103_01433	hoi104_01482	hoi108_01193	hoi110_00800	hoi111_00075	hoi112_01344	hoi113_01673	hoi114_00578	hoi115_00879	hoi116_00656	hoi105_01143	hoi107_00784	hoi109_00873	hoi117_01292	hoi106_00804
1425	AB hydrolase superfamily protein YdjP	16	Core		hoi102_01464	hoi103_01432	hoi104_01483	hoi108_01194	hoi110_00801	hoi111_00074	hoi112_01345	hoi113_01674	hoi114_00579	hoi115_00880	hoi116_00657	hoi105_01142	hoi107_00783	hoi109_00872	hoi117_01291	hoi106_00803
1426	Pyruvate dehydrogenase E1 component	16	Core		hoi102_01465	hoi103_01431	hoi104_01484	hoi108_01195	hoi110_00802	hoi111_00073	hoi112_01346	hoi113_01675	hoi114_00580	hoi115_00881	hoi116_00658	hoi105_01141	hoi107_00782	hoi109_00871	hoi117_01290	hoi106_00802
1427	hypothetical protein	16	Core		hoi102_01466	hoi103_01430	hoi104_01485	hoi108_01196	hoi110_00803	hoi111_00072	hoi112_01347	hoi113_01676	hoi114_00581	hoi115_00882	hoi116_00659	hoi105_01140	hoi107_00781	hoi109_00870	hoi117_01289	hoi106_00801
1428	Lipase 2 precursor	16	Core		hoi102_01468	hoi103_01428	hoi104_01487	hoi108_01198	hoi110_00805	hoi111_00070	hoi112_01349	hoi113_01678	hoi114_00583	hoi115_00884	hoi116_00661	hoi105_01138	hoi107_00779	hoi109_00868	hoi117_01287	hoi106_00799
1429	cobalamin biosynthesis protein	16	Core		hoi102_01469	hoi103_01427	hoi104_01488	hoi108_01199	hoi110_00806	hoi111_00069	hoi112_01350	hoi113_01679	hoi114_00584	hoi115_00885	hoi116_00662	hoi105_01137	hoi107_00778	hoi109_00867	hoi117_01286	hoi106_00798
1430	SURF1 family protein	16	Core		hoi102_01470	hoi103_01426	hoi104_01489	hoi108_01200	hoi110_00807	hoi111_00068	hoi112_01351	hoi113_01680	hoi114_00585	hoi115_00886	hoi116_00663	hoi105_01136	hoi107_00777	hoi109_00866	hoi117_01285	hoi106_00797
1431	putative low molecular weight protein-tyrosine-p	16	Core		hoi102_01471	hoi103_01425	hoi104_01490	hoi108_01201	hoi110_00808	hoi111_00067	hoi112_01352	hoi113_01681	hoi114_00586	hoi115_00887	hoi116_00664	hoi105_01135	hoi107_00776	hoi109_00865	hoi117_01284	hoi106_00796
1432	5'-nucleotidase	16	Core		hoi102_01472	hoi103_01424	hoi104_01491	hoi108_01202	hoi110_00809	hoi111_00066	hoi112_01353	hoi113_01682	hoi114_00587	hoi115_00888	hoi116_00665	hoi105_01134	hoi107_00775	hoi109_00864	hoi117_01283	hoi106_00795
1433	Threonine-phosphate decarboxylase	16	Core		hoi102_01473	hoi103_01423	hoi104_01492	hoi108_01203	hoi110_00810	hoi111_00065	hoi112_01354	hoi113_01683	hoi114_00588	hoi115_00889	hoi116_00666	hoi105_01133	hoi107_00774	hoi109_00863	hoi117_01282	hoi106_00794
1434	Putative GTP cyclohydrolase 1 type 2	16	Core		hoi102_01474	hoi103_01422	hoi104_01493	hoi108_01204	hoi110_00811	hoi111_00064	hoi112_01355	hoi113_01684	hoi114_00589	hoi115_00890	hoi116_00667	hoi105_01132	hoi107_00773	hoi109_00862	hoi117_01281	hoi106_00793
1435	Putative zinc ribbon domain protein	16	Core		hoi102_01475	hoi103_01421	hoi104_01494	hoi108_01205	hoi110_00812	hoi111_00063	hoi112_01356	hoi113_01685	hoi114_00590	hoi115_00891	hoi116_00668	hoi105_01131	hoi107_00772	hoi109_00861	hoi117_01280	hoi106_00792
1436	2,3-bisphosphoglycerate-dependent phosphogly	16	Core		hoi102_01476	hoi103_01420	hoi104_01495	hoi108_01206	hoi110_00813	hoi111_00062	hoi112_01357	hoi113_01686	hoi114_00591	hoi115_00892	hoi116_00669	hoi105_01130	hoi107_00771	hoi109_00860	hoi117_01279	hoi106_00791
1437	Hydroxymethylpyrimidine/phosphomethylpyrimi	16	Core		hoi102_01477	hoi103_01419	hoi104_01496	hoi108_01207	hoi110_00814	hoi111_00061	hoi112_01358	hoi113_01687	hoi114_00592	hoi115_00893	hoi116_00670	hoi105_01129	hoi107_00770	hoi109_00859	hoi117_01278	hoi106_00790

1438	hypothetical protein	16	Core	hoi102_01478	hoi103_01418	hoi104_01497	hoi108_01208	hoi110_00815	hoi111_00060	hoi112_01359	hoi113_01688	hoi114_00593	hoi115_00894	hoi116_00671	hoi105_01128	hoi107_00769	hoi109_00858	hoi117_01277	hoi106_00789
1439	hypothetical protein	16	Core	hoi102_01479	hoi103_01417	hoi104_01498	hoi108_01209	hoi110_00816	hoi111_00059	hoi112_01360	hoi113_01689	hoi114_00594	hoi115_00895	hoi116_00672	hoi105_01127	hoi107_00768	hoi109_00857	hoi117_01276	hoi106_00788
1440	CHAD domain protein	16	Core	hoi102_01480	hoi103_01416	hoi104_01499	hoi108_01210	hoi110_00817	hoi111_00058	hoi112_01361	hoi113_01690	hoi114_00595	hoi115_00896	hoi116_00673	hoi105_01126	hoi107_00767	hoi109_00856	hoi117_01275	hoi106_00787
1441	putative glutamine synthetase 2	16	Core	hoi102_01481	hoi103_01415	hoi104_01500	hoi108_01211	hoi110_00818	hoi111_00057	hoi112_01362	hoi113_01691	hoi114_00596	hoi115_00897	hoi116_00674	hoi105_01125	hoi107_00766	hoi109_00855	hoi117_01274	hoi106_00786
1442	Glutamate-ammonia-ligase adenylyltransferase	16	Core	hoi102_01482	hoi103_01414	hoi104_01501	hoi108_01212	hoi110_00819	hoi111_00056	hoi112_01363	hoi113_01692	hoi114_00597	hoi115_00898	hoi116_00675	hoi105_01124	hoi107_00765	hoi109_00854	hoi117_01273	hoi106_00785
1443	hypothetical protein	16	Core	hoi102_01483	hoi103_01413	hoi104_01502	hoi108_01213	hoi110_00820	hoi111_00055	hoi112_01364	hoi113_01693	hoi114_00598	hoi115_00899	hoi116_00676	hoi105_01123	hoi107_00764	hoi109_00853	hoi117_01272	hoi106_00784
1444	L-galactonate transporter	16	Core	hoi102_01484	hoi103_01412	hoi104_01503	hoi108_01214	hoi110_00821	hoi111_00054	hoi112_01365	hoi113_01694	hoi114_00599	hoi115_00900	hoi116_00677	hoi105_01122	hoi107_00763	hoi109_00852	hoi117_01271	hoi106_00783
1445	Glutaminase 1	16	Core	hoi102_01485	hoi103_01411	hoi104_01504	hoi108_01215	hoi110_00822	hoi111_00053	hoi112_01366	hoi113_01695	hoi114_00600	hoi115_00901	hoi116_00678	hoi105_01121	hoi107_00762	hoi109_00851	hoi117_01270	hoi106_00782
1446	hypothetical protein	16	Core	hoi102_01486	hoi103_01410	hoi104_01505	hoi108_01216	hoi110_00823	hoi111_00052	hoi112_01367	hoi113_01696	hoi114_00601	hoi115_00902	hoi116_00679	hoi105_01120	hoi107_00761	hoi109_00850	hoi117_01269	hoi106_00781
1447	Cation/acetate symporter ActP	16	Core	hoi102_01487	hoi103_01409	hoi104_01506	hoi108_01217	hoi110_00824	hoi111_00051	hoi112_01368	hoi113_01697	hoi114_00602	hoi115_00903	hoi116_00680	hoi105_01119	hoi107_00760	hoi109_00849	hoi117_01268	hoi106_00780
1448	Inner membrane protein YbaN	16	Core	hoi102_01488	hoi103_01408	hoi104_01507	hoi108_01218	hoi110_00825	hoi111_00050	hoi112_01369	hoi113_01698	hoi114_00603	hoi115_00904	hoi116_00681	hoi105_01118	hoi107_00759	hoi109_00848	hoi117_01267	hoi106_00779
1449	Threonine synthase	16	Core	hoi102_01489	hoi103_01407	hoi104_01508	hoi108_01219	hoi110_00826	hoi111_00049	hoi112_01370	hoi113_01699	hoi114_00604	hoi115_00905	hoi116_00682	hoi105_01117	hoi107_00758	hoi109_00847	hoi117_01266	hoi106_00778
1450	dihydroneopterin triphosphate pyrophosphatase	16	Core	hoi102_01490	hoi103_01406	hoi104_01509	hoi108_01220	hoi110_00827	hoi111_00048	hoi112_01371	hoi113_01700	hoi114_00605	hoi115_00906	hoi116_00683	hoi105_01116	hoi107_00757	hoi109_00846	hoi117_01265	hoi106_00777
1451	Aerobic glycerol-3-phosphate dehydrogenase	16	Core	hoi102_01491	hoi103_01405	hoi104_01510	hoi108_01221	hoi110_00828	hoi111_00047	hoi112_01372	hoi113_01701	hoi114_00606	hoi115_00907	hoi116_00684	hoi105_01115	hoi107_00756	hoi109_00845	hoi117_01264	hoi106_00776
1452	putative glycerol uptake facilitator protein	16	Core	hoi102_01492	hoi103_01404	hoi104_01511	hoi108_01222	hoi110_00829	hoi111_00046	hoi112_01373	hoi113_01702	hoi114_00607	hoi115_00908	hoi116_00685	hoi105_01114	hoi107_00755	hoi109_00844	hoi117_01263	hoi106_00775
1453	Glycerol kinase	16	Core	hoi102_01493	hoi103_01403	hoi104_01512	hoi108_01223	hoi110_00830	hoi111_00045	hoi112_01374	hoi113_01703	hoi114_00608	hoi115_00909	hoi116_00686	hoi105_01113	hoi107_00754	hoi109_00843	hoi117_01262	hoi106_00774
1454	hypothetical protein	16	Core	hoi102_01494	hoi103_01402	hoi104_01513	hoi108_01224	hoi110_00831	hoi111_00044	hoi112_01375	hoi113_01704	hoi114_00609	hoi115_00910	hoi116_00687	hoi105_01112	hoi107_00753	hoi109_00842	hoi117_01261	hoi106_00773
1455	division inhibitor protein	16	Core	hoi102_01495	hoi103_01401	hoi104_01514	hoi108_01225	hoi110_00832	hoi111_00043	hoi112_01376	hoi113_01705	hoi114_00610	hoi115_00911	hoi116_00688	hoi105_01111	hoi107_00752	hoi109_00841	hoi117_01260	hoi106_00772
1456	Antiseptic resistance protein	16	Core	hoi102_01496	hoi103_01400	hoi104_01515	hoi108_01226	hoi110_00833	hoi111_00042	hoi112_01377	hoi113_01706	hoi114_00611	hoi115_00912	hoi116_00689	hoi105_01110	hoi107_00751	hoi109_00840	hoi117_01259	hoi106_00771
1457	Haemolysin-III related	16	Core	hoi102_01497	hoi103_01399	hoi104_01516	hoi108_01227	hoi110_00834	hoi111_00041	hoi112_01378	hoi113_01707	hoi114_00612	hoi115_00913	hoi116_00690	hoi105_01109	hoi107_00750	hoi109_00839	hoi117_01258	hoi106_00770
1458	Metal-binding protein ZinT precursor	16	Core	hoi102_01498	hoi103_01398	hoi104_01517	hoi108_01228	hoi110_00835	hoi111_00040	hoi112_01379	hoi113_01708	hoi114_00613	hoi115_00914	hoi116_00691	hoi105_01108	hoi107_00749	hoi109_00838	hoi117_01257	hoi106_00769
1459	High-affinity zinc uptake system binding-protein	16	Core	hoi102_01499	hoi103_01397	hoi104_01518	hoi108_01229	hoi110_00836	hoi111_00039	hoi112_01380	hoi113_01709	hoi114_00614	hoi115_00915	hoi116_00692	hoi105_01107	hoi107_00748	hoi109_00837	hoi117_01256	hoi106_00768
1460	High-affinity zinc uptake system ATP-binding pro	16	Core	hoi102_01500	hoi103_01396	hoi104_01519	hoi108_01230	hoi110_00837	hoi111_00038	hoi112_01381	hoi113_01710	hoi114_00615	hoi115_00916	hoi116_00693	hoi105_01106	hoi107_00747	hoi109_00836	hoi117_01255	hoi106_00767
1461	High-affinity zinc uptake system membrane prot	16	Core	hoi102_01501	hoi103_01395	hoi104_01520	hoi108_01231	hoi110_00838	hoi111_00037	hoi112_01382	hoi113_01711	hoi114_00616	hoi115_00917	hoi116_00694	hoi105_01105	hoi107_00746	hoi109_00835	hoi117_01254	hoi106_00766
1462	YodA lipocalin-like domain protein	16	Core	hoi102_01502	hoi103_01394	hoi104_01521	hoi108_01232	hoi110_00839	hoi111_00036	hoi112_01383	hoi113_01712	hoi114_00617	hoi115_00918	hoi116_00695	hoi105_01104	hoi107_00745	hoi109_00834	hoi117_01253	hoi106_00765
1463	Glutamine synthetase 1	16	Core	hoi102_01503	hoi103_01393	hoi104_01522	hoi108_01233	hoi110_00840	hoi111_00035	hoi112_01384	hoi113_01713	hoi114_00618	hoi115_00919	hoi116_00696	hoi105_01102	hoi107_00743	hoi109_00832	hoi117_01251	hoi106_00763
1464	RDD family protein	16	Core	hoi102_01504	hoi103_01392	hoi104_01523	hoi108_01234	hoi110_00841	hoi111_00034	hoi112_01385	hoi113_01714	hoi114_00619	hoi115_00920	hoi116_00697	hoi105_01101	hoi107_00742	hoi109_00831	hoi117_01250	hoi106_00762
1465	Aspartate/alanine antiporter	16	Core	hoi102_01505	hoi103_01391	hoi104_01524	hoi108_01235	hoi110_00842	hoi111_00033	hoi112_01386	hoi113_01715	hoi114_00620	hoi115_00921	hoi116_00698	hoi105_01100	hoi107_00741	hoi109_00830	hoi117_01249	hoi106_00761
1466	hypothetical protein	16	Core	hoi102_01506	hoi103_01390	hoi104_01525	hoi108_01236	hoi110_00843	hoi111_00032	hoi112_01387	hoi113_01716	hoi114_00621	hoi115_00922	hoi116_00699	hoi105_01099	hoi107_00740	hoi109_00829	hoi117_01248	hoi106_00760
1467	Lipoyl synthase	16	Core	hoi102_01507	hoi103_01389	hoi104_01526	hoi108_01237	hoi110_00844	hoi111_00031	hoi112_01388	hoi113_01717	hoi114_00622	hoi115_00923	hoi116_00700	hoi105_01098	hoi107_00739	hoi109_00828	hoi117_01247	hoi106_00759
1468	Octanoyltransferase	16	Core	hoi102_01508	hoi103_01388	hoi104_01527	hoi108_01238	hoi110_00845	hoi111_00030	hoi112_01389	hoi113_01718	hoi114_00623	hoi115_00924	hoi116_00701	hoi105_01097	hoi107_00738	hoi109_00827	hoi117_01246	hoi106_00758
1469	Glycine cleavage system H protein	16	Core	hoi102_01509	hoi103_01387	hoi104_01528	hoi108_01239	hoi110_00846	hoi111_00029	hoi112_01390	hoi113_01719	hoi114_00624	hoi115_00925	hoi116_00702	hoi105_01096	hoi107_00737	hoi109_00826	hoi117_01245	hoi106_00757
1470	Aminomethyltransferase	16	Core	hoi102_01510	hoi103_01386	hoi104_01529	hoi108_01240	hoi110_00847	hoi111_00028	hoi112_01391	hoi113_01720	hoi114_00625	hoi115_00926	hoi116_00703	hoi105_01095	hoi107_00736	hoi109_00825	hoi117_01244	hoi106_00756
1471	Glycine dehydrogenase (decarboxylating)	16	Core	hoi102_01511	hoi103_01385	hoi104_01530	hoi108_01241	hoi110_00848	hoi111_00027	hoi112_01392	hoi113_01721	hoi114_00626	hoi115_00927	hoi116_00704	hoi105_01094	hoi107_00735	hoi109_00824	hoi117_01243	hoi106_00755
1472	Dihydrolipoyllysine-residue acetyltransferase cor	16	Core	hoi102_01512	hoi103_01384	hoi104_01531	hoi108_01242	hoi110_00849	hoi111_00026	hoi112_01393	hoi113_01722	hoi114_00627	hoi115_00928	hoi116_00705	hoi105_01093	hoi107_00734	hoi109_00823	hoi117_01242	hoi106_00754
1473	hypothetical protein	16	Core	hoi102_01513	hoi103_01383	hoi104_01532	hoi108_01243	hoi110_00850	hoi111_00025	hoi112_01394	hoi113_01723	hoi114_00628	hoi115_00929	hoi116_00706	hoi105_01092	hoi107_00733	hoi109_00822	hoi117_01241	hoi106_00753
1474	Cytosol aminopeptidase	16	Core	hoi102_01514	hoi103_01382	hoi104_01533	hoi108_01244	hoi110_00851	hoi111_00024	hoi112_01395	hoi113_01724	hoi114_00629	hoi115_00930	hoi116_00707	hoi105_01091	hoi107_00732	hoi109_00821	hoi117_01240	hoi106_00752
1475	Branched-chain-amino-acid aminotransferase	16	Core	hoi102_01515	hoi103_01381	hoi104_01534	hoi108_01245	hoi110_00852	hoi111_00023	hoi112_01396	hoi113_01725	hoi114_00630	hoi115_00931	hoi116_00708	hoi105_01090	hoi107_00731	hoi109_00820	hoi117_01239	hoi106_00751
1476	hypothetical protein	16	Core	hoi102_01516	hoi103_01380	hoi104_01535	hoi108_01246	hoi110_00853	hoi111_00022	hoi112_01397	hoi113_01726	hoi114_00631	hoi115_00932	hoi116_00709	hoi105_01089	hoi107_00730	hoi109_00819	hoi117_01238	hoi106_00750
1477	Cobalamin synthase	16	Core	hoi102_01517	hoi103_01379	hoi104_01536	hoi108_01247	hoi110_00854	hoi111_00021	hoi112_01398	hoi113_01727	hoi114_00632	hoi115_00933	hoi116_00710	hoi105_01088	hoi107_00729	hoi109_00818	hoi117_01237	hoi106_00749
1478	Nicotinate-nucleotide--dimethylbenzimidazole pl	16	Core	hoi102_01518	hoi103_01378	hoi104_01537	hoi108_01248	hoi110_00855	hoi111_00020	hoi112_01399	hoi113_01728	hoi114_00633	hoi115_00934	hoi116_00711	hoi105_01087	hoi107_00728	hoi109_00817	hoi117_01236	hoi106_00748
1479	Bifunctional adenosylcobalamin biosynthesis pro	16	Core	hoi102_01519	hoi103_01377	hoi104_01538	hoi108_01249	hoi110_00856	hoi111_00019	hoi112_01400	hoi113_01729	hoi114_00634	hoi115_00935	hoi116_00712	hoi105_01086	hoi107_00727	hoi109_00816	hoi117_01235	hoi106_00747
1480	hypothetical protein	16	Core	hoi102_01520	hoi103_01376	hoi104_01539	hoi108_01250	hoi110_00857	hoi111_00018	hoi112_01401	hoi113_01730	hoi114_00635	hoi115_00936	hoi116_00713	hoi105_01085	hoi107_00726	hoi109_00815	hoi117_01234	hoi106_00746
1481	Iron-sulfur cluster insertion protein ErpA	16	Core	hoi102_01521	hoi103_01375	hoi104_01540	hoi108_01251	hoi110_00858	hoi111_00017	hoi112_01402	hoi113_01731	hoi114_00636	hoi115_00937	hoi116_00714	hoi105_01084	hoi107_00725	hoi109_00814	hoi117_01233	hoi106_00745
1482	Putative asparagine synthetase [glutamine-hydro	16	Core	hoi102_01522	hoi103_01374	hoi104_01541	hoi108_01252	hoi110_00859	hoi111_00016	hoi112_01403	hoi113_01732	hoi114_00637	hoi115_00938	hoi116_00715	hoi105_01083	hoi107_00724	hoi109_00813	hoi117_01232	hoi106_00744
1483	Cytochrome c oxidase subunit 2 precursor	16	Core	hoi102_01523	hoi103_01373	hoi104_01542	hoi108_01253	hoi110_00860	hoi111_00015	hoi112_01404	hoi113_01733	hoi114_00638	hoi115_00939	hoi116_00716	hoi105_01082	hoi107_00723	hoi109_00812	hoi117_01231	hoi106_00743
1484	Cytochrome c oxidase polypeptide 4	16	Core	hoi102_01524	hoi103_01372	hoi104_01543	hoi108_01254	hoi110_00861	hoi111_00014	hoi112_01405	hoi113_01734	hoi114_00639	hoi115_00940	hoi116_00717	hoi105_01081	hoi107_00722	hoi109_00811	hoi117_01230	hoi106_00742
1485	Cytochrome c oxidase subunit 3	16	Core	hoi102_01525	hoi103_01371	hoi104_01544	hoi108_01255	hoi110_00862	hoi111_00013	hoi112_01406	hoi113_01735	hoi114_00640	hoi115_00941	hoi116_00718	hoi105_01080	hoi107_00721	hoi109_00810	hoi117_01229	hoi106_00741
1486	Menaquinol-cytochrome c reductase cytochrom	16	Core	hoi102_01526	hoi103_01370	hoi104_01545	hoi108_01256	hoi110_00863	hoi111_00012	hoi112_01407	hoi113_01736	hoi114_00641	hoi115_00942	hoi116_00719	hoi105_01079	hoi107_00720	hoi109_00809	hoi117_01228	hoi106_00740
1487	Arsenite oxidase subunit AioB precursor	16	Core	hoi102_01527	hoi103_01369	hoi104_01546	hoi108_01257	hoi110_00864	hoi111_00011	hoi112_01408	hoi113_01737	hoi114_00642	hoi115_00943	hoi116_00720	hoi105_01078	hoi107_00719	hoi109_00808	hoi117_01227	hoi106_00739
1488	Cytochrome b6	16	Core	hoi102_01528	hoi103_01368	hoi104_01547	hoi108_01258	hoi110_00865	hoi111_00010	hoi112_01409	hoi113_01738	hoi114_00643	hoi115_00944	hoi116_00721	hoi105_01077	hoi107_00718	hoi109_00807	hoi117_01226	hoi106_00738
1489	putative endopeptidase precursor	16	Core	hoi102_01529	hoi103_01367	hoi104_01548	hoi108_01259	hoi110_00866	hoi111_00009	hoi112_01410	hoi113_01739	hoi114_00644	hoi115_00945	hoi116_00722	hoi105_01075	hoi107_00716	hoi109_00805	hoi117_01224	hoi106_00737
1490	putative endopeptidase precursor	16	Core	hoi102_01530	hoi103_01366	hoi104_01549	hoi108_01260	hoi110_00867	hoi111_00008	hoi112_01411	hoi113_01740	hoi114_00645	hoi115_00946	hoi116_00723	hoi105_01074	hoi107_00715	hoi109_00804	hoi117_01223	hoi106_00736
1491	GDP-mannose-dependent alpha-(1-6)-phosphati	16	Core	hoi102_01531	hoi103_01365	hoi104_01550	hoi108_01261	hoi110_00868	hoi111_00007	hoi112_01412	hoi113_01741	hoi114_00646	hoi115_00947	hoi116_00724	hoi105_01073	hoi107_00714	hoi109_00803	hoi117_01222	hoi106_00735
1492	Glucokinase	16	Core	hoi102_01532	hoi103_01364	hoi104_01551	hoi108_01262	hoi110_00869	hoi111_00006	hoi112_01413	hoi113_01742	hoi114_00647	hoi115_00948	hoi116_00725	hoi105_01072	hoi107_00713	hoi109_00802	hoi117_01221	hoi106_00734
1493	2-acyl-glycerophospho-ethanolamine acyltransfe	16	Core	hoi102_01533	hoi103_01363	hoi104_01552	hoi108_01263	hoi110_00870	hoi111_00005	hoi112_01414	hoi113_01743	hoi114_00648	hoi115_00949	hoi116_00726	hoi105_01071	hoi107_00712	hoi109_00801	hoi117_01220	hoi106_00733
1494	Acyltransferase family protein	16	Core	hoi102_01534	hoi103_01362	hoi104_01553	hoi108_01264	hoi110_00871	hoi111_00004	hoi112_01415	hoi113_01744	hoi114_00649	hoi115_00950	hoi116_00727	hoi105_01070	hoi107_00711	hoi109_00800	hoi117_01219	hoi106_00732
1495	3'-5' exoribonuclease/MT2234.1	16	Core	hoi102_01535	hoi103_01361	hoi104_01554	hoi108_01265	hoi110_00872	hoi111_00003	hoi112_01416	hoi113_01745	hoi114_00650	hoi115_00951	hoi116_00728	hoi105_01069	hoi107_00710	hoi109_00799	hoi117_01218	hoi106_00731
1496	Phospho-2-dehydro-3-deoxyheptonate aldolase	16	Core	hoi102_01536	hoi103_01360	hoi104_01555	hoi108_01266	hoi110_00873	hoi111_00002	hoi112_01417	hoi113_01746	hoi114_00651	hoi115_00952	hoi116_00729	hoi105_01068	hoi107_00709	hoi109_00798	hoi117_01217	hoi106_00730
1497	Serine/threonine-protein kinase PK-1	16	Core	hoi102_01537	hoi103_01359	hoi104_01556	hoi108_01267	hoi110_00874	hoi111_00001	hoi112_01418	hoi113_01747	hoi114_00652	hoi115_00953	hoi116_00730	hoi105_01067	hoi107_00708	hoi109_00797	hoi117_01216	hoi106_00729
1498	acyl-CoA thioesterase YbgC	16	Core	hoi102_01538	hoi103_01166	hoi104_01557	hoi108_01667	hoi110_01348	hoi111_00197	hoi112_01423	hoi113_02043	hoi114_01160	hoi115_01798	hoi116_00534	hoi105_02135	hoi107_00906	hoi109_01681	hoi117_00611	hoi106_00926
1499	Pyridoxamine kinase	16	Core	hoi102_01539	hoi103_01165	hoi104_01558	hoi108_01668	hoi110_01349	hoi111_00198	hoi112_01424	hoi113_02042	hoi114_01161	hoi115_01799	hoi116_00533	hoi105_02134	hoi107_00907	hoi109_01680	hoi117_00612	hoi106_00927
1500	putative ABC transporter ATP-binding protein/M	16	Core	hoi102_01540	hoi103_01164	hoi104_01559	hoi108_01669	hoi110_01350	hoi111_00199	hoi112_01425	hoi113_02041	hoi114_01162	hoi115_01800	hoi116_00532	hoi105_02133	hoi107_00908	hoi109_01679	hoi117_00613	hoi106_00928
1501	Single-stranded DNA-binding protein	16	Core	hoi102_01541	hoi103_01163	hoi104_01560	hoi108_01670	hoi110_01351	hoi111_00200	hoi112_01426	hoi113_02040	hoi114_01163	hoi115_01801	hoi116_00531	hoi105_02132	hoi107_00909	hoi109_01678	hoi117_00614	hoi106_00929
1502	Inner membrane protein YebZ	16	Core	hoi102_01542	hoi103_01162	hoi104_01561	hoi108_01671	hoi110_01352	hoi111_00201	hoi112_01427	hoi113_02039	hoi114_01164	hoi115_01802	hoi116_00530	hoi105_02131	hoi107_00910	hoi109_01677	hoi117_00615	hoi106_00930
1503	ornithine cyclodeaminase	16	Core	hoi102_01544	hoi103_01160	hoi104_01563	hoi108_01673	hoi110_01354	hoi111_00203	hoi112_01429	hoi113_02037	hoi114_01166	hoi115_01804	hoi116_00528	hoi105_02129	hoi107_00912	hoi109_01675	hoi117_00617	hoi106_00932
1504	Putative ketoacyl reductase	16	Core	hoi102_01545	hoi103_01159	hoi104_01564	hoi108_01674	hoi110_01355	hoi111_00204	hoi112_01430	hoi113_02036	hoi114_01167	hoi115_01805	hoi116_00527	hoi105_02128	hoi107_00913	hoi109_01674	hoi117_00618	hoi106_00933
1505	Oligoribonuclease	16	Core	hoi102_01546	hoi103_01158	hoi104_01565	hoi108_01675	hoi110_01356	hoi111_00205	hoi112_01431	hoi113_02035	hoi114_01168	hoi115_01806	hoi116_00526	hoi105_02127	hoi107_00914	hoi109_01673	hoi117_00619	hoi106_00934
1506	Aspartate/alanine antiporter	16	Core	hoi102_01547	hoi103_01157	hoi104_01566	hoi108_01676	hoi110_01357	hoi111_00206	hoi112_01432	hoi113_02034	hoi114_01169	hoi115_01807	hoi116_00525	hoi105_02126	hoi107_00915	hoi109_01672	hoi117_00620	hoi106_00935
1507	hypothetical protein	16	Core	hoi102_01549	hoi103_01155	hoi104_01568	hoi108_01678	hoi110_01359	hoi111_00208	hoi112_01434	hoi113_02032	hoi114_01171	hoi115_01809	hoi116_00523	hoi105_02124	hoi107_00917	hoi109_01670	hoi117_00622	hoi106_00937
1508	hypothetical protein	16	Core	hoi102_01550	hoi103_01154	hoi104_01569	hoi108_01679	hoi110_01360	hoi111_00209	hoi112_01435	hoi113_02031	hoi114_01172	hoi115_01810	hoi116_00522	hoi105_02123	hoi107_00918	hoi109_01669	hoi117_00623	hoi106_00938
1509	Alkanal monooxygenase alpha chain	16	Core	hoi102_01551	hoi103_01153	hoi104_01570	hoi108_01680	hoi110_01361	hoi111_00210	hoi112_01436	hoi113_02030	hoi114_01173	hoi115_01811	hoi116_00521	hoi105_02122	hoi107_00919	hoi109_01668	hoi117_00624	hoi106_00939
1510	Putative L,D-transpeptidase LppS precursor	16	Core	hoi102_01552	hoi103_01152	hoi104_01571	hoi108_01681	hoi110_01362	hoi111_00211	hoi112_01437	hoi113_02029	hoi114_01174	hoi115_01812	hoi116_00520	hoi105_02121	hoi107_00920	hoi109_01667	hoi117_00625	hoi106_00940
1511	hypothetical protein	16	Core	hoi102_01554	hoi103_01150	hoi104_01573	hoi108_01683	hoi110_01364	hoi111_00213	hoi112_01439	hoi113_02027	hoi114_01176	hoi115_01814	hoi116_00518	hoi105_02118	hoi107_00923	hoi109_01664	hoi117_00628	hoi106_00943
1512	hypothetical protein	16	Core	hoi102_01555	hoi103_01149	hoi104_01574	hoi108_01684	hoi110_01365	hoi111_00214	hoi112_01440	hoi113_02026	hoi114_01177	hoi115_01815	hoi116_00517	hoi105_02117	hoi107_00924	hoi109_01663	hoi117_00629	hoi106_00944
1513	hypothetical protein	16	Core	hoi102_01556	hoi103_01148	hoi104_01575	hoi108_01685	hoi110_01366	hoi111_00215	hoi112_01441	hoi113_02025	hoi114_01178	hoi115_01816	hoi116_00516	hoi105_02116	hoi107_00925	hoi109_01662	hoi117_00630	hoi106_00945
1514	Putative peroxiredoxin/MT2597	16	Core	hoi102_01557	hoi103_01147	hoi104_01576	hoi108_01686	hoi110_01367	hoi111_00216	hoi112_01442	hoi113_02024	hoi114_01179	hoi115_01817	hoi116_00515	hoi105_02115	hoi107_00926	hoi109_01661	hoi117_00631	hoi106_00946
1515	Bacterial regulatory proteins, tetR family	16	Core	hoi102_01558	hoi103_01146	hoi104_01577	hoi108_01687	hoi110_01368	hoi111_00217	hoi112_01443	hoi113_02023	hoi114_01180	hoi115_01818	hoi116_00514	hoi105_02114	hoi107_00927	hoi109_01660	hoi117_00632	hoi106_00947
1516	hypothetical protein	16	Core	hoi102_01559	hoi103_01145	hoi104_01578	hoi108_01688	hoi110_01369	hoi111_00218	hoi112_01444	hoi113_02022	hoi114_01181	hoi115_01819	hoi116_00513	hoi105_02113	hoi107_00928	hoi109_01659	hoi117_00633	hoi106_00948
1517	Holo-[acyl-carrier-protein] synthase	16	Core	hoi102_01560	hoi103_01144	hoi104_01579	hoi108_01689	hoi110_01370	hoi111_00219	hoi112_01445	hoi113_02021	hoi114_01182	hoi115_01820	hoi116_00512	hoi105_01709	hoi107_00929	hoi109_01658	hoi117_00634	hoi106_00949

1518	3-oxoacyl-[acyl-carrier-protein] synthase 2	16	Core	hoi102_01561	hoi103_01143	hoi104_01580	hoi108_01690	hoi110_01371	hoi111_00220	hoi112_01446	hoi113_02020	hoi114_01183	hoi115_01821	hoi116_00511	hoi105_01708	hoi107_00930	hoi109_01657	hoi117_00635	hoi106_00950
1519	FtsX-like permease family protein	16	Core	hoi102_01562	hoi103_01142	hoi104_01581	hoi108_01691	hoi110_01372	hoi111_00221	hoi112_01447	hoi113_02019	hoi114_01184	hoi115_01822	hoi116_00510	hoi105_01707	hoi107_00931	hoi109_01656	hoi117_00636	hoi106_00951
1520	Macrolide export ATP-binding/permease protein	16	Core	hoi102_01563	hoi103_01141	hoi104_01582	hoi108_01692	hoi110_01373	hoi111_00222	hoi112_01448	hoi113_02018	hoi114_01185	hoi115_01823	hoi116_00509	hoi105_01706	hoi107_00932	hoi109_01655	hoi117_00637	hoi106_00952
1521	hypothetical protein	16	Core	hoi102_01564	hoi103_01140	hoi104_01583	hoi108_01693	hoi110_01374	hoi111_00223	hoi112_01449	hoi113_02017	hoi114_01186	hoi115_01824	hoi116_00508	hoi105_01705	hoi107_00933	hoi109_01654	hoi117_00638	hoi106_00953
1522	Sensor histidine kinase ComP	11	Accessory	hoi102_01565	hoi103_01139	hoi104_01584	hoi108_01694	hoi110_01375	hoi111_00224	hoi112_01450	hoi113_02016	hoi114_01187	hoi115_01825	hoi116_00507					
1523	Nitrogen assimilation regulatory protein	16	Core	hoi102_01566	hoi103_01138	hoi104_01585	hoi108_01695	hoi110_01376	hoi111_00225	hoi112_01451	hoi113_02015	hoi114_01188	hoi115_01826	hoi116_00506	hoi105_01704	hoi107_00934	hoi109_01653	hoi117_00639	hoi106_00954
1524	Nitrate/nitrite response regulator protein NarL	11	Accessory	hoi102_01567	hoi103_01137	hoi104_01586	hoi108_01696	hoi110_01377	hoi111_00226	hoi112_01452	hoi113_02014	hoi114_01189	hoi115_01827	hoi116_00505					
1525	Succinate-semialdehyde dehydrogenase [NADP(·	16	Core	hoi102_01568	hoi103_01136	hoi104_01587	hoi108_01697	hoi110_01378	hoi111_00227	hoi112_01453	hoi113_02013	hoi114_01190	hoi115_01828	hoi116_00504	hoi105_01703	hoi107_00935	hoi109_01652	hoi117_00640	hoi106_00955
1526	hypothetical protein	11	Accessory	hoi102_01569	hoi103_01135	hoi104_01588	hoi108_01698	hoi110_01379	hoi111_00228	hoi112_01454	hoi113_02012	hoi114_01191	hoi115_01829	hoi116_00503					
1527	hypothetical protein	11	Accessory	hoi102_01570	hoi103_01134	hoi104_01589	hoi108_01699	hoi110_01380	hoi111_00229	hoi112_01455	hoi113_02011	hoi114_01192	hoi115_01830	hoi116_00502					
1528	hypothetical protein	16	Core	hoi102_01573	hoi103_01131	hoi104_01592	hoi108_01702	hoi110_01383	hoi111_00232	hoi112_01458	hoi113_02008	hoi114_01195	hoi115_01833	hoi116_00499	hoi105_01701	hoi107_00937	hoi109_01650	hoi117_00642	hoi106_00957
1529	hypothetical protein	16	Core	hoi102_01574	hoi103_01130	hoi104_01593	hoi108_01703	hoi110_01384	hoi111_00233	hoi112_01459	hoi113_02007	hoi114_01196	hoi115_01834	hoi116_00498	hoi105_01700	hoi107_00938	hoi109_01649	hoi117_00643	hoi106_00958
1530	Non-canonical purine NTP pyrophosphatase	16	Core	hoi102_01575	hoi103_01129	hoi104_01594	hoi108_01704	hoi110_01385	hoi111_00234	hoi112_01460	hoi113_02006	hoi114_01197	hoi115_01835	hoi116_00497	hoi105_01699	hoi107_00939	hoi109_01648	hoi117_00644	hoi106_00959
1531	Ribonuclease PH	16	Core	hoi102_01576	hoi103_01128	hoi104_01595	hoi108_01705	hoi110_01386	hoi111_00235	hoi112_01461	hoi113_02005	hoi114_01198	hoi115_01836	hoi116_00496	hoi105_01698	hoi107_00940	hoi109_01647	hoi117_00645	hoi106_00960
1532	Ribonuclease BN	16	Core	hoi102_01577	hoi103_01127	hoi104_01596	hoi108_01706	hoi110_01387	hoi111_00236	hoi112_01462	hoi113_02004	hoi114_01199	hoi115_01837	hoi116_00495	hoi105_01697	hoi107_00941	hoi109_01646	hoi117_00646	hoi106_00961
1533	Glutamate racemase	16	Core	hoi102_01578	hoi103_01126	hoi104_01597	hoi108_01707	hoi110_01388	hoi111_00237	hoi112_01463	hoi113_02003	hoi114_01200	hoi115_01838	hoi116_00494	hoi105_01696	hoi107_00942	hoi109_01645	hoi117_00647	hoi106_00962
1534	hypothetical protein	16	Core	hoi102_01579	hoi103_01125	hoi104_01598	hoi108_01708	hoi110_01389	hoi111_00238	hoi112_01464	hoi113_02002	hoi114_01201	hoi115_01839	hoi116_00493	hoi105_01695	hoi107_00943	hoi109_01644	hoi117_00648	hoi106_00963
1535	Rhomboid family protein	16	Core	hoi102_01580	hoi103_01124	hoi104_01599	hoi108_01709	hoi110_01390	hoi111_00239	hoi112_01465	hoi113_02001	hoi114_01202	hoi115_01840	hoi116_00492	hoi105_01694	hoi107_00944	hoi109_01643	hoi117_00649	hoi106_00964
1536	Peptidase family S58	16	Core	hoi102_01581	hoi103_01123	hoi104_01600	hoi108_01710	hoi110_01391	hoi111_00240	hoi112_01466	hoi113_02000	hoi114_01203	hoi115_01841	hoi116_00491	hoi105_01693	hoi107_00945	hoi109_01642	hoi117_00650	hoi106_00965
1537	hypothetical protein	16	Core	hoi102_01582	hoi103_01122	hoi104_01601	hoi108_01711	hoi110_01392	hoi111_00241	hoi112_01467	hoi113_01999	hoi114_01204	hoi115_01842	hoi116_00490	hoi105_01692	hoi107_00946	hoi109_01641	hoi117_00651	hoi106_00966
1538	ATP-dependent Clp protease adaptor protein Clp	16	Core	hoi102_01583	hoi103_01121	hoi104_01602	hoi108_01712	hoi110_01393	hoi111_00242	hoi112_01468	hoi113_01998	hoi114_01205	hoi115_01843	hoi116_00489	hoi105_01691	hoi107_00947	hoi109_01640	hoi117_00652	hoi106_00967
1539	Nicotinate phosphoribosyltransferase pncB1	16	Core	hoi102_01584	hoi103_01120	hoi104_01603	hoi108_01713	hoi110_01394	hoi111_00243	hoi112_01469	hoi113_01997	hoi114_01206	hoi115_01844	hoi116_00488	hoi105_01690	hoi107_00948	hoi109_01639	hoi117_00653	hoi106_00968
1540	hypothetical protein	16	Core	hoi102_01585	hoi103_01119	hoi104_01604	hoi108_01714	hoi110_01395	hoi111_00244	hoi112_01470	hoi113_01996	hoi114_01207	hoi115_01845	hoi116_00487	hoi105_01689	hoi107_00949	hoi109_01638	hoi117_00654	hoi106_00969
1541	Long-chain-fatty-acid--CoA ligase	16	Core	hoi102_01586	hoi103_01118	hoi104_01605	hoi108_01715	hoi110_01396	hoi111_00245	hoi112_01471	hoi113_01995	hoi114_01208	hoi115_01846	hoi116_00486	hoi105_01688	hoi107_00950	hoi109_01637	hoi117_00655	hoi106_00970
1542	hypothetical protein	16	Core	hoi102_01587	hoi103_01117	hoi104_01606	hoi108_01716	hoi110_01397	hoi111_00246	hoi112_01472	hoi113_01994	hoi114_01209	hoi115_01847	hoi116_00485	hoi105_01687	hoi107_00951	hoi109_01636	hoi117_00656	hoi106_00971
1543	hypothetical protein	16	Core	hoi102_01588	hoi103_01116	hoi104_01607	hoi108_01717	hoi110_01398	hoi111_00247	hoi112_01473	hoi113_01993	hoi114_01210	hoi115_01848	hoi116_00484	hoi105_01686	hoi107_00952	hoi109_01635	hoi117_00657	hoi106_00972
1544	peptidyl-tRNA hydrolase	16	Core	hoi102_01589	hoi103_01115	hoi104_01608	hoi108_01718	hoi110_01399	hoi111_00248	hoi112_01474	hoi113_01992	hoi114_01211	hoi115_01849	hoi116_00483	hoi105_01685	hoi107_00953	hoi109_01634	hoi117_00658	hoi106_00973
1545	Phosphoserine phosphatase	16	Core	hoi102_01590	hoi103_01114	hoi104_01609	hoi108_01719	hoi110_01400	hoi111_00249	hoi112_01475	hoi113_01991	hoi114_01212	hoi115_01850	hoi116_00482	hoi105_01684	hoi107_00954	hoi109_01633	hoi117_00659	hoi106_00974
1546	Cytochrome c oxidase subunit 1	16	Core	hoi102_01591	hoi103_01113	hoi104_01610	hoi108_01720	hoi110_01401	hoi111_00250	hoi112_01476	hoi113_01990	hoi114_01213	hoi115_01851	hoi116_00481	hoi105_01683	hoi107_00955	hoi109_01632	hoi117_00660	hoi106_00975
1547	Ribonucleoside-diphosphate reductase subunit b	16	Core	hoi102_01592	hoi103_01112	hoi104_01611	hoi108_01721	hoi110_01402	hoi111_00251	hoi112_01477	hoi113_01989	hoi114_01214	hoi115_01852	hoi116_00480	hoi105_01682	hoi107_00956	hoi109_01631	hoi117_00661	hoi106_00976
1548	Ferritin	16	Core	hoi102_01593	hoi103_01111	hoi104_01612	hoi108_01722	hoi110_01403	hoi111_00252	hoi112_01478	hoi113_01988	hoi114_01215	hoi115_01853	hoi116_00479	hoi105_01681	hoi107_00957	hoi109_01630	hoi117_00662	hoi106_00977
1549	Thermoresistant gluconokinase	16	Core	hoi102_01594	hoi103_01110	hoi104_01613	hoi108_01723	hoi110_01404	hoi111_00253	hoi112_01479	hoi113_01987	hoi114_01216	hoi115_01854	hoi116_00478	hoi105_01680	hoi107_00958	hoi109_01629	hoi117_00663	hoi106_00978
1550	Inner membrane permease YgbN	16	Core	hoi102_01595	hoi103_01109	hoi104_01614	hoi108_01724	hoi110_01405	hoi111_00254	hoi112_01480	hoi113_01986	hoi114_01217	hoi115_01855	hoi116_00477	hoi105_01679	hoi107_00959	hoi109_01628	hoi117_00664	hoi106_00979
1551	hypothetical protein	16	Core	hoi102_01596	hoi103_01108	hoi104_01615	hoi108_01725	hoi110_01406	hoi111_00255	hoi112_01481	hoi113_01985	hoi114_01218	hoi115_01856	hoi116_00476	hoi105_01678	hoi107_00960	hoi109_01627	hoi117_00665	hoi106_00980
1552	hypothetical protein	16	Core	hoi102_01597	hoi103_01107	hoi104_01616	hoi108_01726	hoi110_01407	hoi111_00256	hoi112_01482	hoi113_01984	hoi114_01219	hoi115_01857	hoi116_00475	hoi105_01672	hoi107_00966	hoi109_01621	hoi117_00671	hoi106_00981
1553	Hydroperoxy fatty acid reductase gpx1	16	Core	hoi102_01598	hoi103_01106	hoi104_01617	hoi108_01727	hoi110_01408	hoi111_00257	hoi112_01483	hoi113_01983	hoi114_01220	hoi115_01858	hoi116_00474	hoi105_01671	hoi107_00967	hoi109_01620	hoi117_00672	hoi106_00982
1554	hypothetical protein	16	Core	hoi102_01599	hoi103_01105	hoi104_01618	hoi108_01728	hoi110_01409	hoi111_00258	hoi112_01484	hoi113_01982	hoi114_01221	hoi115_01859	hoi116_00473	hoi105_01670	hoi107_00968	hoi109_01619	hoi117_00673	hoi106_00983
1555	5,6-dimethylbenzimidazole synthase	16	Core	hoi102_01600	hoi103_01104	hoi104_01619	hoi108_01729	hoi110_01410	hoi111_00259	hoi112_01485	hoi113_01981	hoi114_01222	hoi115_01860	hoi116_00472	hoi105_01669	hoi107_00969	hoi109_01618	hoi117_00674	hoi106_00984
1556	Protease 2	16	Core	hoi102_01601	hoi103_01103	hoi104_01620	hoi108_01730	hoi110_01411	hoi111_00260	hoi112_01486	hoi113_01980	hoi114_01223	hoi115_01861	hoi116_00471	hoi105_01668	hoi107_00970	hoi109_01617	hoi117_00675	hoi106_00985
1557	hypothetical protein	16	Core	hoi102_01602	hoi103_01102	hoi104_01621	hoi108_01731	hoi110_01412	hoi111_00261	hoi112_01487	hoi113_01979	hoi114_01224	hoi115_01862	hoi116_00470	hoi105_01667	hoi107_00971	hoi109_01616	hoi117_00676	hoi106_00986
1558	Hemin import ATP-binding protein HmuV	16	Core	hoi102_01603	hoi103_01101	hoi104_01622	hoi108_01732	hoi110_01413	hoi111_00262	hoi112_01488	hoi113_01978	hoi114_01225	hoi115_01863	hoi116_00469	hoi105_01666	hoi107_00972	hoi109_01615	hoi117_00677	hoi106_00987
1559	Hemin transport system permease protein HmuU	16	Core	hoi102_01604	hoi103_01100	hoi104_01623	hoi108_01733	hoi110_01414	hoi111_00263	hoi112_01489	hoi113_01977	hoi114_01226	hoi115_01864	hoi116_00468	hoi105_01665	hoi107_00973	hoi109_01614	hoi117_00678	hoi106_00988
1560	corrinoid ABC transporter substrate-binding prot	16	Core	hoi102_01605	hoi103_01099	hoi104_01624	hoi108_01734	hoi110_01415	hoi111_00264	hoi112_01490	hoi113_01976	hoi114_01227	hoi115_01865	hoi116_00467	hoi105_01664	hoi107_00974	hoi109_01613	hoi117_00679	hoi106_00989
1561	Phosphoribosylaminoimidazole-succinocarboxan	16	Core	hoi102_01606	hoi103_01098	hoi104_01625	hoi108_01735	hoi110_01416	hoi111_00265	hoi112_01491	hoi113_01975	hoi114_01228	hoi115_01866	hoi116_00466	hoi105_01663	hoi107_00975	hoi109_01612	hoi117_00680	hoi106_00990
1562	Adenylosuccinate lyase	16	Core	hoi102_01607	hoi103_01097	hoi104_01626	hoi108_01736	hoi110_01417	hoi111_00266	hoi112_01492	hoi113_01974	hoi114_01229	hoi115_01867	hoi116_00465	hoi105_01662	hoi107_00976	hoi109_01611	hoi117_00681	hoi106_00991
1563	Ktr system potassium uptake protein A	16	Core	hoi102_01608	hoi103_01096	hoi104_01627	hoi108_01737	hoi110_01418	hoi111_00267	hoi112_01493	hoi113_01973	hoi114_01230	hoi115_01868	hoi116_00464	hoi105_01661	hoi107_00977	hoi109_01610	hoi117_00682	hoi106_00992
1564	Ktr system potassium uptake protein B	16	Core	hoi102_01609	hoi103_01095	hoi104_01628	hoi108_01738	hoi110_01419	hoi111_00268	hoi112_01494	hoi113_01972	hoi114_01231	hoi115_01869	hoi116_00463	hoi105_01660	hoi107_00978	hoi109_01609	hoi117_00683	hoi106_00993
1565	Putative acetyltransferase	16	Core	hoi102_01610	hoi103_01094	hoi104_01629	hoi108_01739	hoi110_01420	hoi111_00269	hoi112_01495	hoi113_01971	hoi114_01232	hoi115_01870	hoi116_00462	hoi105_01659	hoi107_00979	hoi109_01608	hoi117_00684	hoi106_00994
1566	Phosphoribosylamine--glycine ligase	16	Core	hoi102_01611	hoi103_01093	hoi104_01630	hoi108_01740	hoi110_01421	hoi111_00270	hoi112_01496	hoi113_01970	hoi114_01233	hoi115_01871	hoi116_00461	hoi105_01658	hoi107_00980	hoi109_01607	hoi117_00685	hoi106_00995
1567	HIT-like protein	16	Core	hoi102_01612	hoi103_01092	hoi104_01631	hoi108_01741	hoi110_01422	hoi111_00271	hoi112_01497	hoi113_01969	hoi114_01234	hoi115_01872	hoi116_00460	hoi105_01657	hoi107_00981	hoi109_01606	hoi117_00686	hoi106_00996
1568	3-ketosteroid-9-alpha-hydroxylase reductase sub	16	Core	hoi102_01613	hoi103_01091	hoi104_01632	hoi108_01742	hoi110_01423	hoi111_00272	hoi112_01498	hoi113_01968	hoi114_01235	hoi115_01873	hoi116_00459	hoi105_01656	hoi107_00982	hoi109_01605	hoi117_00687	hoi106_00997
1569	putative sensor histidine kinase TcrY	16	Core	hoi102_01614	hoi103_01090	hoi104_01633	hoi108_01743	hoi110_01424	hoi111_00273	hoi112_01499	hoi113_01967	hoi114_01236	hoi115_01874	hoi116_00458	hoi105_01655	hoi107_00983	hoi109_01604	hoi117_00688	hoi106_00998
1570	putative transcriptional regulatory protein TcrX	16	Core	hoi102_01615	hoi103_01089	hoi104_01634	hoi108_01744	hoi110_01425	hoi111_00274	hoi112_01500	hoi113_01966	hoi114_01237	hoi115_01875	hoi116_00457	hoi105_01654	hoi107_00984	hoi109_01603	hoi117_00689	hoi106_00999
1571	Pyruvate dehydrogenase [ubiquinone]	16	Core	hoi102_01616	hoi103_01088	hoi104_01635	hoi108_01745	hoi110_01426	hoi111_00275	hoi112_01501	hoi113_01965	hoi114_01238	hoi115_01876	hoi116_00456	hoi105_01653	hoi107_00985	hoi109_01602	hoi117_00690	hoi106_01000
1572	Vacuole effluxer Atg22 like protein	16	Core	hoi102_01617	hoi103_01087	hoi104_01636	hoi108_01746	hoi110_01427	hoi111_00276	hoi112_01502	hoi113_01964	hoi114_01239	hoi115_01877	hoi116_00455	hoi105_01652	hoi107_00986	hoi109_01601	hoi117_00691	hoi106_01001
1573	Meso-diaminopimelate D-dehydrogenase	16	Core	hoi102_01618	hoi103_01086	hoi104_01637	hoi108_01747	hoi110_01428	hoi111_00277	hoi112_01503	hoi113_01963	hoi114_01240	hoi115_01878	hoi116_00454	hoi105_01651	hoi107_00987	hoi109_01600	hoi117_00692	hoi106_01002
1574	hypothetical protein	10	Accessory	hoi102_01619	hoi103_01085	hoi104_01638	hoi108_01748	hoi110_01429	hoi111_00278	hoi112_01504	hoi113_01962		hoi115_01879	hoi116_00453					
1575	Multidrug resistance ABC transporter ATP-bindin	11	Accessory	hoi102_01620	hoi103_01084	hoi104_01639	hoi108_01749	hoi110_01430	hoi111_00279	hoi112_01505	hoi113_01961	hoi114_01241	hoi115_01880	hoi116_00452					
1576	putative formate transporter 1	16	Core	hoi102_01621	hoi103_01083	hoi104_01640	hoi108_01750	hoi110_01431	hoi111_00280	hoi112_01506	hoi113_01960	hoi114_01242	hoi115_01881	hoi116_00451	hoi105_01650	hoi107_00988	hoi109_01599	hoi117_00693	hoi106_01003
1577	hypothetical protein	16	Core	hoi102_01623	hoi103_01081	hoi104_01642	hoi108_01752	hoi110_01433	hoi111_00282	hoi112_01508	hoi113_01958	hoi114_01244	hoi115_01883	hoi116_00449	hoi105_01648	hoi107_00990	hoi109_01597	hoi117_00695	hoi106_01005
1578	hypothetical protein	16	Core	hoi102_01624	hoi103_01080	hoi104_01643	hoi108_01753	hoi110_01434	hoi111_00283	hoi112_01509	hoi113_01957	hoi114_01245	hoi115_01884	hoi116_00448	hoi105_01647	hoi107_00991	hoi109_01596	hoi117_00696	hoi106_01006
1579	Trehalose-phosphate synthase	16	Core	hoi102_01625	hoi103_01079	hoi104_01644	hoi108_01754	hoi110_01435	hoi111_00284	hoi112_01510	hoi113_01956	hoi114_01246	hoi115_01885	hoi116_00447	hoi105_01646	hoi107_00992	hoi109_01595	hoi117_00697	hoi106_01007
1580	hypothetical protein	16	Core	hoi102_01626	hoi103_01078	hoi104_01645	hoi108_01755	hoi110_01436	hoi111_00285	hoi112_01511	hoi113_01955	hoi114_01247	hoi115_01886	hoi116_00446	hoi105_01645	hoi107_00993	hoi109_01594	hoi117_00698	hoi106_01008
1581	Trehalose-6-phosphate phosphatase	16	Core	hoi102_01627	hoi103_01077	hoi104_01646	hoi108_01756	hoi110_01437	hoi111_00286	hoi112_01512	hoi113_01954	hoi114_01248	hoi115_01887	hoi116_00445	hoi105_01644	hoi107_00994	hoi109_01593	hoi117_00699	hoi106_01009
1582	Catabolite control protein A	16	Core	hoi102_01628	hoi103_01076	hoi104_01647	hoi108_01757	hoi110_01438	hoi111_00287	hoi112_01513	hoi113_01953	hoi114_01249	hoi115_01888	hoi116_00444	hoi105_01643	hoi107_00995	hoi109_01592	hoi117_00700	hoi106_01010
1583	Manganese ABC transporter substrate-binding li	16	Core	hoi102_01629	hoi103_01075	hoi104_01648	hoi108_01758	hoi110_01439	hoi111_00288	hoi112_01514	hoi113_01952	hoi114_01250	hoi115_01889	hoi116_00443	hoi105_01642	hoi107_00996	hoi109_01591	hoi117_00701	hoi106_01011
1584	putative ABC transporter ATP-binding protein	16	Core	hoi102_01630	hoi103_01074	hoi104_01649	hoi108_01759	hoi110_01440	hoi111_00289	hoi112_01515	hoi113_01951	hoi114_01251	hoi115_01890	hoi116_00442	hoi105_01641	hoi107_00997	hoi109_01590	hoi117_00702	hoi106_01012
1585	High-affinity zinc uptake system membrane prot	16	Core	hoi102_01631	hoi103_01073	hoi104_01650	hoi108_01760	hoi110_01441	hoi111_00290	hoi112_01516	hoi113_01950	hoi114_01252	hoi115_01891	hoi116_00441	hoi105_01640	hoi107_00998	hoi109_01589	hoi117_00703	hoi106_01013
1586	Inner membrane metabolite transport protein Ył	16	Core	hoi102_01632	hoi103_01072	hoi104_01651	hoi108_01761	hoi110_01442	hoi111_00291	hoi112_01517	hoi113_01949	hoi114_01253	hoi115_01892	hoi116_00440	hoi105_01639	hoi107_00999	hoi109_01588	hoi117_00704	hoi106_01014
1587	putative multidrug resistance ABC transporter AT	16	Core	hoi102_01633	hoi103_01071	hoi104_01652	hoi108_01762	hoi110_01443	hoi111_00292	hoi112_01518	hoi113_01948	hoi114_01254	hoi115_01893	hoi116_00439	hoi105_01638	hoi107_01000	hoi109_01587	hoi117_00705	hoi106_01015
1588	putative ABC transporter ATP-binding protein	16	Core	hoi102_01634	hoi103_01070	hoi104_01653	hoi108_01763	hoi110_01444	hoi111_00293	hoi112_01519	hoi113_01947	hoi114_01255	hoi115_01894	hoi116_00438	hoi105_01637	hoi107_01001	hoi109_01586	hoi117_00706	hoi106_01016
1589	Putative TrmH family tRNA/rRNA methyltransfer	16	Core	hoi102_01635	hoi103_01069	hoi104_01654	hoi108_01764	hoi110_01445	hoi111_00294	hoi112_01520	hoi113_01946	hoi114_01256	hoi115_01895	hoi116_00437	hoi105_01636	hoi107_01002	hoi109_01585	hoi117_00707	hoi106_01017
1590	Cysteine--tRNA ligase	16	Core	hoi102_01636	hoi103_01068	hoi104_01655	hoi108_01765	hoi110_01446	hoi111_00295	hoi112_01521	hoi113_01945	hoi114_01257	hoi115_01896	hoi116_00436	hoi105_01635	hoi107_01003	hoi109_01584	hoi117_00708	hoi106_01018
1591	2-C-methyl-D-erythritol 2,4-cyclodiphosphate sy	16	Core	hoi102_01637	hoi103_01067	hoi104_01656	hoi108_01766	hoi110_01447	hoi111_00296	hoi112_01522	hoi113_01944	hoi114_01258	hoi115_01897	hoi116_00435	hoi105_01634	hoi107_01004	hoi109_01583	hoi117_00709	hoi106_01019
1592	2-C-methyl-D-erythritol 4-phosphate cytidylyltra	16	Core	hoi102_01638	hoi103_01066	hoi104_01657	hoi108_01767	hoi110_01448	hoi111_00297	hoi112_01523	hoi113_01943	hoi114_01259	hoi115_01898	hoi116_00434	hoi105_01633	hoi107_01005	hoi109_01582	hoi117_00710	hoi106_01020
1593	RNA polymerase-binding transcription factor Car	16	Core	hoi102_01639	hoi103_01065	hoi104_01658	hoi108_01768	hoi110_01449	hoi111_00298	hoi112_01524	hoi113_01942	hoi114_01260	hoi115_01899	hoi116_00433	hoi105_01632	hoi107_01006	hoi109_01581	hoi117_00711	hoi106_01021
1594	hypothetical protein	16	Core	hoi102_01640	hoi103_01064	hoi104_01659	hoi108_01769	hoi110_01450	hoi111_00299	hoi112_01525	hoi113_01941	hoi114_01261	hoi115_01900	hoi116_00432	hoi105_01631	hoi107_01007	hoi109_01580	hoi117_00712	hoi106_01022
1595	hypothetical protein	16	Core	hoi102_01641	hoi103_01063	hoi104_01660	hoi108_01770	hoi110_01451	hoi111_00300	hoi112_01526	hoi113_01940	hoi114_01262	hoi115_01901	hoi116_00431	hoi105_01630	hoi107_01008	hoi109_01579	hoi117_00713	hoi106_01023
1596	hypothetical protein	16	Core	hoi102_01642	hoi103_01062	hoi104_01661	hoi108_01771	hoi110_01452	hoi111_00301	hoi112_01527	hoi113_01939	hoi114_01263	hoi115_01902	hoi116_00430	hoi105_01629	hoi107_01009	hoi109_01578	hoi117_00714	hoi106_01024
1597	Carbonic anhydrase	16	Core	hoi102_01643	hoi103_01061	hoi104_01662	hoi108_01772	hoi110_01453	hoi111_00302	hoi112_01528	hoi113_01938	hoi114_01264	hoi115_01903	hoi116_00429	hoi105_01628	hoi107_01010	hoi109_01577	hoi117_00715	hoi106_01025

1598	A/G-specific adenine glycosylase	16	Core		hoi102_01644	hoi103_01060	hoi104_01663	hoi108_01773	hoi110_01454	hoi111_00303	hoi112_01529	hoi113_01937	hoi114_01265	hoi115_01904	hoi116_00428	hoi105_01627	hoi107_01011	hoi109_01576	hoi117_00716	hoi106_01026
1599	Hemin transport system permease protein HmuU	15	Accessory		hoi102_01645	hoi103_01059	hoi104_01664	hoi108_01774	hoi110_01455	hoi111_00304	hoi112_01530	hoi113_01936	hoi114_01266	hoi115_01905	hoi116_00427	hoi105_01626	hoi107_01012	hoi109_01575	hoi117_00717	
1600	Hemin import ATP-binding protein HmuV	15	Accessory		hoi102_01646	hoi103_01058	hoi104_01665	hoi108_01775	hoi110_01456	hoi111_00305	hoi112_01531	hoi113_01935	hoi114_01267	hoi115_01906	hoi116_00426	hoi105_01625	hoi107_01013	hoi109_01574	hoi117_00718	
1601	Htaa	15	Accessory		hoi102_01647	hoi103_01057	hoi104_01666	hoi108_01776	hoi110_01457	hoi111_00306	hoi112_01532	hoi113_01934	hoi114_01268	hoi115_01907	hoi116_00425	hoi105_01624	hoi107_01014	hoi109_01573	hoi117_00719	
1602	hypothetical protein	15	Accessory		hoi102_01648	hoi103_01056	hoi104_01667	hoi108_01777	hoi110_01458	hoi111_00307	hoi112_01533	hoi113_01933	hoi114_01269	hoi115_01908	hoi116_00424	hoi105_01623	hoi107_01015	hoi109_01572	hoi117_00720	
1603	Htaa	15	Accessory		hoi102_01649	hoi103_01055	hoi104_01668	hoi108_01778	hoi110_01459	hoi111_00308	hoi112_01534	hoi113_01932	hoi114_01270	hoi115_01909	hoi116_00423	hoi105_01622	hoi107_02091	hoi109_01571	hoi117_00721	
1604	Hemin-binding periplasmic protein HmuT precurs	15	Accessory		hoi102_01650	hoi103_01054	hoi104_01669	hoi108_01779	hoi110_01460	hoi111_00309	hoi112_01535	hoi113_01931	hoi114_01271	hoi115_01910	hoi116_00422	hoi105_01621	hoi107_02092	hoi109_01570	hoi117_00722	
1605	Htaa	15	Accessory		hoi102_01651	hoi103_01053	hoi104_01670	hoi108_01780	hoi110_01461	hoi111_00310	hoi112_01536	hoi113_01930	hoi114_01272	hoi115_01911	hoi116_00421	hoi105_01620	hoi107_02093	hoi109_01569	hoi117_00723	
1606	hypothetical protein	16	Core		hoi102_01652	hoi103_01052	hoi104_01671	hoi108_01781	hoi110_01462	hoi111_00311	hoi112_01537	hoi113_01929	hoi114_01273	hoi115_01912	hoi116_00420	hoi105_01619	hoi107_02094	hoi109_01568	hoi117_00724	hoi106_01027
1607	ATP-dependent Clp protease ATP-binding subuni	16	Core		hoi102_01653	hoi103_01051	hoi104_01672	hoi108_01782	hoi110_01463	hoi111_00312	hoi112_01538	hoi113_01928	hoi114_01274	hoi115_01913	hoi116_00419	hoi105_01618	hoi107_02095	hoi109_01567	hoi117_00725	hoi106_01028
1608	Heme oxygenase	16	Core		hoi102_01654	hoi103_01050	hoi104_01673	hoi108_01783	hoi110_01464	hoi111_00313	hoi112_01539	hoi113_01927	hoi114_01275	hoi115_01914	hoi116_00418	hoi105_01617	hoi107_02096	hoi109_01566	hoi117_00726	hoi106_01029
1609	Inosine-5'-monophosphate dehydrogenase	16	Core		hoi102_01655	hoi103_01049	hoi104_01674	hoi108_01784	hoi110_01465	hoi111_00314	hoi112_01540	hoi113_01926	hoi114_01276	hoi115_01915	hoi116_00417	hoi105_01616	hoi107_02097	hoi109_01565	hoi117_00727	hoi106_01030
1610	putative phosphomannomutase	16	Core		hoi102_01656	hoi103_01048	hoi104_01675	hoi108_01785	hoi110_01466	hoi111_00315	hoi112_01541	hoi113_01925	hoi114_01277	hoi115_01916	hoi116_00416	hoi105_01615	hoi107_02098	hoi109_01564	hoi117_00728	hoi106_01031
1611	Deoxyribose-phosphate aldolase	16	Core		hoi102_01657	hoi103_01047	hoi104_01676	hoi108_01786	hoi110_01467	hoi111_00316	hoi112_01542	hoi113_01924	hoi114_01278	hoi115_01917	hoi116_00415	hoi105_01614	hoi107_02099	hoi109_01563	hoi117_00729	hoi106_01032
1612	Pyrimidine-nucleoside phosphorylase	16	Core		hoi102_01658	hoi103_01046	hoi104_01677	hoi108_01787	hoi110_01468	hoi111_00317	hoi112_01543	hoi113_01923	hoi114_01279	hoi115_01918	hoi116_00414	hoi105_01613	hoi107_02100	hoi109_01562	hoi117_00730	hoi106_01033
1613	Cytidine deaminase	16	Core		hoi102_01659	hoi103_01045	hoi104_01678	hoi108_01788	hoi110_01469	hoi111_00318	hoi112_01544	hoi113_01922	hoi114_01280	hoi115_01919	hoi116_00413	hoi105_01612	hoi107_02101	hoi109_01561	hoi117_00731	hoi106_01034
1614	Nucleoside permease NupX	16	Core		hoi102_01660	hoi103_01044	hoi104_01679	hoi108_01789	hoi110_01470	hoi111_00319	hoi112_01545	hoi113_01921	hoi114_01281	hoi115_01920	hoi116_00412	hoi105_01611	hoi107_02102	hoi109_01560	hoi117_00732	hoi106_01035
1615	Deoxyribonucleoside regulator	16	Core		hoi102_01661	hoi103_01043	hoi104_01680	hoi108_01790	hoi110_01471	hoi111_00320	hoi112_01546	hoi113_01920	hoi114_01282	hoi115_01921	hoi116_00411	hoi105_01610	hoi107_02103	hoi109_01559	hoi117_00733	hoi106_01036
1616	Hydrogenase expression/formation protein HypD	16	Core		hoi102_01662	hoi103_01042	hoi104_01681	hoi108_01791	hoi110_01472	hoi111_00321	hoi112_01547	hoi113_01919	hoi114_01283	hoi115_01922	hoi116_00410	hoi105_01609	hoi107_02104	hoi109_01558	hoi117_00734	hoi106_01037
1617	Hydrogenase isoenzymes formation protein Hyp	16	Core		hoi102_01663	hoi103_01041	hoi104_01682	hoi108_01792	hoi110_01473	hoi111_00322	hoi112_01548	hoi113_01918	hoi114_01284	hoi115_01923	hoi116_00409	hoi105_01608	hoi107_02105	hoi109_01557	hoi117_00735	hoi106_01038
1618	Hydrogenase isoenzymes formation protein Hyp	16	Core		hoi102_01664	hoi103_01040	hoi104_01683	hoi108_01793	hoi110_01474	hoi111_00323	hoi112_01549	hoi113_01917	hoi114_01285	hoi115_01924	hoi116_00408	hoi105_01607	hoi107_02106	hoi109_01556	hoi117_00736	hoi106_01039
1619	NAD-reducing hydrogenase HoxS subunit beta	16	Core		hoi102_01665	hoi103_01039	hoi104_01684	hoi108_01794	hoi110_01475	hoi111_00324	hoi112_01550	hoi113_01916	hoi114_01286	hoi115_01925	hoi116_00407	hoi105_01606	hoi107_02107	hoi109_01555	hoi117_00737	hoi106_01040
1620	hydrogenase nickel incorporation protein	16	Core		hoi102_01666	hoi103_01038	hoi104_01685	hoi108_01795	hoi110_01476	hoi111_00325	hoi112_01551	hoi113_01915	hoi114_01287	hoi115_01926	hoi116_00406	hoi105_01605	hoi107_02108	hoi109_01554	hoi117_00738	hoi106_01041
1621	Hydrogenase 2 maturation protease	16	Core		hoi102_01667	hoi103_01037	hoi104_01686	hoi108_01796	hoi110_01477	hoi111_00326	hoi112_01552	hoi113_01914	hoi114_01288	hoi115_01927	hoi116_00405	hoi105_01604	hoi107_02109	hoi109_01553	hoi117_00739	hoi106_01042
1622	putative Ni/Fe-hydrogenase B-type cytochrome s	16	Core		hoi102_01668	hoi103_01036	hoi104_01687	hoi108_01797	hoi110_01478	hoi111_00327	hoi112_01553	hoi113_01913	hoi114_01289	hoi115_01928	hoi116_00404	hoi105_01603	hoi107_02110	hoi109_01552	hoi117_00740	hoi106_01043
1623	Hydrogenase-1 large chain	16	Core		hoi102_01669	hoi103_01035	hoi104_01688	hoi108_01798	hoi110_01479	hoi111_00328	hoi112_01554	hoi113_01912	hoi114_01290	hoi115_01929	hoi116_00403	hoi105_01602	hoi107_02111	hoi109_01551	hoi117_00741	hoi106_01044
1624	Uptake hydrogenase small subunit precursor	16	Core		hoi102_01670	hoi103_01034	hoi104_01689	hoi108_01799	hoi110_01480	hoi111_00329	hoi112_01555	hoi113_01911	hoi114_01291	hoi115_01930	hoi116_00402	hoi105_01601	hoi107_02112	hoi109_01550	hoi117_00742	hoi106_01045
1625	Hydrogenase isoenzymes nickel incorporation pr	16	Core		hoi102_01671	hoi103_01033	hoi104_01690	hoi108_01800	hoi110_01481	hoi111_00330	hoi112_01556	hoi113_01910	hoi114_01292	hoi115_01931	hoi116_00401	hoi105_01600	hoi107_02113	hoi109_01549	hoi117_00743	hoi106_01046
1626	hydrogenase nickel incorporation protein	16	Core		hoi102_01672	hoi103_01032	hoi104_01691	hoi108_01801	hoi110_01482	hoi111_00331	hoi112_01557	hoi113_01909	hoi114_01293	hoi115_01932	hoi116_00400	hoi105_01599	hoi107_02114	hoi109_01548	hoi117_00744	hoi106_01047
1627	Carbamoyltransferase HypF	16	Core		hoi102_01673	hoi103_01031	hoi104_01692	hoi108_01802	hoi110_01483	hoi111_00332	hoi112_01558	hoi113_01908	hoi114_01294	hoi115_01933	hoi116_00399	hoi105_01598	hoi107_02115	hoi109_01547	hoi117_00745	hoi106_01048
1628	Hydrogenase expression/formation protein HypE	16	Core		hoi102_01674	hoi103_01030	hoi104_01693	hoi108_01803	hoi110_01484	hoi111_00333	hoi112_01559	hoi113_01907	hoi114_01295	hoi115_01934	hoi116_00398	hoi105_01597	hoi107_02116	hoi109_01546	hoi117_00746	hoi106_01049
1629	Endo-1,4-beta-xylanase Z precursor	16	Core		hoi102_01675	hoi103_01029	hoi104_01694	hoi108_01804	hoi110_01485	hoi111_00334	hoi112_01560	hoi113_01906	hoi114_01296	hoi115_01935	hoi116_00397	hoi105_01596	hoi107_02149	hoi109_01218	hoi117_00747	hoi106_01050
1630	Lysylphosphatidylglycerol biosynthesis bifunctior	16	Core		hoi102_01676	hoi103_01028	hoi104_01695	hoi108_01805	hoi110_01486	hoi111_00335	hoi112_01561	hoi113_01905	hoi114_01297	hoi115_01936	hoi116_00396	hoi105_01595	hoi107_02150	hoi109_01217	hoi117_00748	hoi106_01051
1631	Lysine--tRNA ligase, heat inducible	16	Core		hoi102_01677	hoi103_01027	hoi104_01696	hoi108_01806	hoi110_01487	hoi111_00336	hoi112_01562	hoi113_01904	hoi114_01298	hoi115_01937	hoi116_00395	hoi105_01594	hoi107_02151	hoi109_01216	hoi117_00749	hoi106_01052
1632	Phospholipase YtpA	16	Core		hoi102_01678	hoi103_01026	hoi104_01697	hoi108_01807	hoi110_01488	hoi111_00337	hoi112_01563	hoi113_01903	hoi114_01299	hoi115_01938	hoi116_00394	hoi105_01593	hoi107_02152	hoi109_01215	hoi117_00750	hoi106_01053
1633	putative oxidoreductase/MSMEI_1564	16	Core		hoi102_01679	hoi103_01025	hoi104_01698	hoi108_01808	hoi110_01489	hoi111_00338	hoi112_01564	hoi113_01902	hoi114_01300	hoi115_01939	hoi116_00393	hoi105_01592	hoi107_02153	hoi109_01214	hoi117_00751	hoi106_01054
1634	Inosine-5'-monophosphate dehydrogenase	16	Core		hoi102_01680	hoi103_01024	hoi104_01699	hoi108_01809	hoi110_01490	hoi111_00339	hoi112_01565	hoi113_01901	hoi114_01301	hoi115_01940	hoi116_00392	hoi105_01591	hoi107_02154	hoi109_01213	hoi117_00752	hoi106_01055
1635	hypothetical protein	16	Core		hoi102_01681	hoi103_01023	hoi104_01700	hoi108_01810	hoi110_01491	hoi111_00340	hoi112_01566	hoi113_01900	hoi114_01302	hoi115_01941	hoi116_00391	hoi105_01590	hoi107_02155	hoi109_01212	hoi117_00753	hoi106_01056
1636	hypothetical protein	16	Core		hoi102_01682	hoi103_01022	hoi104_01701	hoi108_01811	hoi110_01492	hoi111_00341	hoi112_01567	hoi113_01899	hoi114_01303	hoi115_01942	hoi116_00390	hoi105_01589	hoi107_02156	hoi109_01211	hoi117_00754	hoi106_01057
1637	ECF RNA polymerase sigma factor SigD	16	Core		hoi102_01683	hoi103_01021	hoi104_01702	hoi108_01812	hoi110_01493	hoi111_00342	hoi112_01568	hoi113_01898	hoi114_01304	hoi115_01943	hoi116_00389	hoi105_01588	hoi107_02157	hoi109_01210	hoi117_00755	hoi106_01058
1638	Bax inhibitor 1 like protein	16	Core		hoi102_01684	hoi103_01725	hoi104_01834	hoi108_01813	hoi110_01763	hoi111_01277	hoi112_01705	hoi113_00462	hoi114_01892	hoi115_02169	hoi116_01816	hoi105_00140	hoi107_00140	hoi109_00451	hoi117_00450	hoi106_00139
1639	Guanosine-5'-triphosphate,3'-diphosphate pyrop	16	Core		hoi102_01686	hoi103_01727	hoi104_01832	hoi108_01815	hoi110_01761	hoi111_01279	hoi112_01707	hoi113_00464	hoi114_01890	hoi115_02171	hoi116_01814	hoi105_00138	hoi107_00138	hoi109_00453	hoi117_00452	hoi106_00137
1640	hypothetical protein	16	Core		hoi102_01687	hoi103_01728	hoi104_01831	hoi108_01816	hoi110_01760	hoi111_01280	hoi112_01708	hoi113_00465	hoi114_01889	hoi115_02172	hoi116_01813	hoi105_00137	hoi107_00137	hoi109_00454	hoi117_00453	hoi106_00136
1641	Septum formation initiator	16	Core		hoi102_01688	hoi103_01729	hoi104_01830	hoi108_01817	hoi110_01759	hoi111_01281	hoi112_01709	hoi113_00466	hoi114_01888	hoi115_02173	hoi116_01812	hoi105_00136	hoi107_00136	hoi109_00455	hoi117_00454	hoi106_00135
1642	Enolase	16	Core		hoi102_01689	hoi103_01730	hoi104_01829	hoi108_01818	hoi110_01758	hoi111_01282	hoi112_01710	hoi113_00467	hoi114_01887	hoi115_02174	hoi116_01811	hoi105_00135	hoi107_00135	hoi109_00456	hoi117_00455	hoi106_00134
1643	hypothetical protein	16	Core		hoi102_01690	hoi103_01731	hoi104_01828	hoi108_01819	hoi110_01757	hoi111_01283	hoi112_01711	hoi113_00468	hoi114_01886	hoi115_02175	hoi116_01810	hoi105_00134	hoi107_00134	hoi109_00457	hoi117_00456	hoi106_00133
1644	Nucleoside triphosphate pyrophosphohydrolase	16	Core		hoi102_01691	hoi103_01732	hoi104_01827	hoi108_01820	hoi110_01756	hoi111_01284	hoi112_01712	hoi113_00469	hoi114_01885	hoi115_02176	hoi116_01809	hoi105_00133	hoi107_00133	hoi109_00458	hoi117_00457	hoi106_00132
1645	Arginine/ornithine antiporter	16	Core		hoi102_01692	hoi103_01733	hoi104_01826	hoi108_01821	hoi110_01755	hoi111_01285	hoi112_01713	hoi113_00470	hoi114_01884	hoi115_02177	hoi116_01808	hoi105_00132	hoi107_00132	hoi109_00459	hoi117_00458	hoi106_00131
1646	Transcription-repair-coupling factor	16	Core		hoi102_01693	hoi103_01734	hoi104_01825	hoi108_01822	hoi110_01754	hoi111_01286	hoi112_01714	hoi113_00471	hoi114_01883	hoi115_02178	hoi116_01807	hoi105_00131	hoi107_00131	hoi109_00460	hoi117_00459	hoi106_00130
1647	HTH-type transcriptional repressor KstR2	16	Core		hoi102_01694	hoi103_01735	hoi104_01824	hoi108_01823	hoi110_01753	hoi111_01287	hoi112_01715	hoi113_00472	hoi114_01882	hoi115_02179	hoi116_01806	hoi105_00130	hoi107_00130	hoi109_00461	hoi117_00460	hoi106_00129
1648	Lipase 2 precursor	16	Core		hoi102_01696	hoi103_01737	hoi104_01822	hoi108_01825	hoi110_01751	hoi111_01289	hoi112_01717	hoi113_00474	hoi114_01880	hoi115_02181	hoi116_01804	hoi105_00128	hoi107_00128	hoi109_00463	hoi117_00462	hoi106_00127
1649	Pyrimidine-specific ribonucleoside hydrolase Rih	15	Accessory		hoi102_01697	hoi103_01738	hoi104_01821	hoi108_01826	hoi110_01750	hoi111_01290	hoi112_01718	hoi113_00475	hoi114_01879		hoi116_01803	hoi105_00127	hoi107_00127	hoi109_00464	hoi117_00463	hoi106_00126
1650	Methionine-binding lipoprotein MetQ precursor	16	Core		hoi102_01698	hoi103_01739	hoi104_01820	hoi108_01827	hoi110_01749	hoi111_01291	hoi112_01719	hoi113_00476	hoi114_01878	hoi115_02061	hoi116_01802	hoi105_00126	hoi107_00126	hoi109_00465	hoi117_00464	hoi106_00125
1651	2-oxoglutarate-dependent ethylene/succinate-fc	16	Core		hoi102_01699	hoi103_01740	hoi104_01819	hoi108_01828	hoi110_01748	hoi111_01292	hoi112_01720	hoi113_00477	hoi114_01877	hoi115_02060	hoi116_01801	hoi105_00125	hoi107_00125	hoi109_00466	hoi117_00465	hoi106_00124
1652	DNA polymerase III PolC-type	15	Accessory		hoi102_01700	hoi103_01741	hoi104_01818	hoi108_01829	hoi110_01747	hoi111_01293	hoi112_01721	hoi113_00478	hoi114_01876		hoi116_01800	hoi105_00124	hoi107_00124	hoi109_00467	hoi117_00466	hoi106_00123
1653	DNA polymerase III PolC-type	2	Accessory		hoi102_01701									hoi115_02059						
1654	Major Facilitator Superfamily protein	16	Core		hoi102_01702	hoi103_01742	hoi104_01817	hoi108_01830	hoi110_01746	hoi111_01294	hoi112_01722	hoi113_00479	hoi114_01875	hoi115_02058	hoi116_01799	hoi105_00123	hoi107_00123	hoi109_00468	hoi117_00467	hoi106_00122
1655	Bifunctional protein GlmU	16	Core		hoi102_01703	hoi103_01743	hoi104_01816	hoi108_01831	hoi110_01745	hoi111_01295	hoi112_01723	hoi113_00480	hoi114_01874	hoi115_02057	hoi116_01798	hoi105_00122	hoi107_00122	hoi109_00469	hoi117_00468	hoi106_00121
1656	Ribose-phosphate pyrophosphokinase	16	Core		hoi102_01704	hoi103_01744	hoi104_01815	hoi108_01832	hoi110_01744	hoi111_01296	hoi112_01724	hoi113_00481	hoi114_01873	hoi115_02056	hoi116_01797	hoi105_00121	hoi107_00121	hoi109_00470	hoi117_00469	hoi106_00120
1657	Modification methylase HpaII	11	Accessory		hoi102_01705	hoi103_01745	hoi104_01814	hoi108_01833	hoi110_01743	hoi111_01297	hoi112_01725	hoi113_00482	hoi114_01872	hoi115_02055	hoi116_01796					
1658	hypothetical protein	11	Accessory	GI8	hoi102_01706	hoi103_01746	hoi104_01813	hoi108_01834	hoi110_01742	hoi111_01298	hoi112_01726	hoi113_00483	hoi114_01871	hoi115_02054	hoi116_01795					
1659	Z1 domain protein	11	Accessory	GI8	hoi102_01707	hoi103_01747	hoi104_01812	hoi108_01835	hoi110_01741	hoi111_01299	hoi112_01727	hoi113_00484	hoi114_01870	hoi115_02053	hoi116_01794					
1660	hypothetical protein	11	Accessory	GI8	hoi102_01708	hoi103_01748	hoi104_01811	hoi108_01836	hoi110_01739	hoi111_01301	hoi112_01729	hoi113_00486	hoi114_01869	hoi115_02052	hoi116_01793					
1661	hypothetical protein	11	Accessory	GI8	hoi102_01709	hoi103_01749	hoi104_01810	hoi108_01837	hoi110_01738	hoi111_01302	hoi112_01730	hoi113_00487	hoi114_01868	hoi115_02051	hoi116_01792					
1662	hypothetical protein	11	Accessory	GI8	hoi102_01710	hoi103_01750	hoi104_01809	hoi108_01838	hoi110_01737	hoi111_01303	hoi112_01731	hoi113_00488	hoi114_01867	hoi115_02050	hoi116_01791					
1663	3-phenylpropionate/cinnamic acid dioxygenase f	16	Core		hoi102_01711	hoi103_01751	hoi104_01808	hoi108_01839	hoi110_01736	hoi111_01304	hoi112_01732	hoi113_00489	hoi114_01866	hoi115_02049	hoi116_01790	hoi105_00104	hoi107_00103	hoi109_00487	hoi117_00486	hoi106_00103
1664	Ureidoglycolate lyase	16	Core		hoi102_01712	hoi103_01752	hoi104_01807	hoi108_01840	hoi110_01735	hoi111_01305	hoi112_01733	hoi113_00490	hoi114_01865	hoi115_02048	hoi116_01789	hoi105_00103	hoi107_00102	hoi109_00488	hoi117_00487	hoi106_00102
1665	General stress protein CTC	16	Core		hoi102_01713	hoi103_01753	hoi104_01806	hoi108_01841	hoi110_01734	hoi111_01306	hoi112_01734	hoi113_00491	hoi114_01864	hoi115_02047	hoi116_01788	hoi105_00102	hoi107_00101	hoi109_00489	hoi117_00488	hoi106_00101
1666	putative D,D-dipeptide-binding periplasmic prote	16	Core		hoi102_01714	hoi103_01754	hoi104_01805	hoi108_01842	hoi110_01733	hoi111_01307	hoi112_01735	hoi113_00492	hoi114_01863	hoi115_02046	hoi116_01787	hoi105_00101	hoi107_00100	hoi109_00490	hoi117_00489	hoi106_00100
1667	L-fuculose phosphate aldolase	16	Core		hoi102_01715	hoi103_01755	hoi104_01804	hoi108_01843	hoi110_01732	hoi111_01308	hoi112_01736	hoi113_00493	hoi114_01862	hoi115_02045	hoi116_01786	hoi105_00100	hoi107_00099	hoi109_00491	hoi117_00490	hoi106_00099
1668	L-fucose isomerase	16	Core		hoi102_01716	hoi103_01756	hoi104_01803	hoi108_01844	hoi110_01731	hoi111_01309	hoi112_01737	hoi113_00494	hoi114_01861	hoi115_02044	hoi116_01785	hoi105_00099	hoi107_00098	hoi109_00492	hoi117_00491	hoi106_00098
1669	Rhamnulokinase	16	Core		hoi102_01717	hoi103_01757	hoi104_01802	hoi108_01845	hoi110_01730	hoi111_01310	hoi112_01738	hoi113_00495	hoi114_01860	hoi115_02043	hoi116_01784	hoi105_00098	hoi107_00097	hoi109_00493	hoi117_00492	hoi106_00097
1670	L-fucose-proton symporter	16	Core		hoi102_01718	hoi103_01758	hoi104_01801	hoi108_01846	hoi110_01729	hoi111_01311	hoi112_01739	hoi113_00496	hoi114_01859	hoi115_02042	hoi116_01783	hoi105_00097	hoi107_00096	hoi109_00494	hoi117_00493	hoi106_00096
1671	L-fucose mutarotase	16	Core		hoi102_01719	hoi103_01759	hoi104_01800	hoi108_01847	hoi110_01728	hoi111_01312	hoi112_01740	hoi113_00497	hoi114_01858	hoi115_02041	hoi116_01782	hoi105_00096	hoi107_00095	hoi109_00495	hoi117_00494	hoi106_00095
1672	Lactose operon repressor	16	Core		hoi102_01720	hoi103_01760	hoi104_01799	hoi108_01848	hoi110_01727	hoi111_01313	hoi112_01741	hoi113_00498	hoi114_01857	hoi115_02040	hoi116_01781	hoi105_00095	hoi107_00094	hoi109_00496	hoi117_00495	hoi106_00094
1673	Peptidyl-tRNA hydrolase	16	Core		hoi102_01721	hoi103_01761	hoi104_01798	hoi108_01849	hoi110_01726	hoi111_01314	hoi112_01742	hoi113_00499	hoi114_01856	hoi115_02039	hoi116_01780	hoi105_00094	hoi107_00093	hoi109_00497	hoi117_00496	hoi106_00093
1674	FtsX-like permease family protein	16	Core		hoi102_01722	hoi103_01762	hoi104_01797	hoi108_01850	hoi110_01725	hoi111_01315	hoi112_01743	hoi113_00500	hoi114_01855	hoi115_02038	hoi116_01779	hoi105_00093	hoi107_00092	hoi109_00498	hoi117_00497	hoi106_00092
1675	ABC transporter ATP-binding protein YtrE	16	Core		hoi102_01723	hoi103_01763	hoi104_01796	hoi108_01851	hoi110_01724	hoi111_01316	hoi112_01744	hoi113_00501	hoi114_01854	hoi115_02037	hoi116_01778	hoi105_00092	hoi107_00091	hoi109_00499	hoi117_00498	hoi106_00091
1676	Sensor histidine kinase LiaS	16	Core		hoi102_01724	hoi103_01764	hoi104_01795	hoi108_01852	hoi110_01723	hoi111_01317	hoi112_01745	hoi113_00502	hoi114_01853	hoi115_02036	hoi116_01777	hoi105_00091	hoi107_00090	hoi109_00500	hoi117_00499	hoi106_00090
1677	Oxygen regulatory protein NreC	16	Core		hoi102_01725	hoi103_01765	hoi104_01794	hoi108_01853	hoi110_01722	hoi111_01318	hoi112_01746	hoi113_00503	hoi114_01852	hoi115_02035	hoi116_01776	hoi105_00090	hoi107_00089	hoi109_00501	hoi117_00500	hoi106_00089

1678	putative thiol peroxidase	16	Core	hoi102_01726	hoi103_01766	hoi104_01793	hoi108_01854	hoi110_01721	hoi111_01319	hoi112_01747	hoi113_00504	hoi114_01851	hoi115_02034	hoi116_01775	hoi105_00089	hoi107_00088	hoi109_00502	hoi117_00501	hoi106_00088
1679	Sodium/glutamate symporter	16	Core	hoi102_01727	hoi103_01767	hoi104_01792	hoi108_01855	hoi110_01720	hoi111_01320	hoi112_01748	hoi113_00505	hoi114_01850	hoi115_02033	hoi116_01774	hoi105_00088	hoi107_00087	hoi109_00503	hoi117_00502	hoi106_00087
1680	Glyceraldehyde-3-phosphate dehydrogenase	16	Core	hoi102_01728	hoi103_01768	hoi104_01791	hoi108_01856	hoi110_01719	hoi111_01321	hoi112_01749	hoi113_00506	hoi114_01849	hoi115_02032	hoi116_01773	hoi105_00087	hoi107_00086	hoi109_00504	hoi117_00503	hoi106_00086
1681	hypothetical protein	16	Core	hoi102_01729	hoi103_01769	hoi104_01790	hoi108_01857	hoi110_01718	hoi111_01322	hoi112_01750	hoi113_00507	hoi114_01848	hoi115_02031	hoi116_01772	hoi105_00086	hoi107_00085	hoi109_00505	hoi117_00504	hoi106_00085
1682	Nitronate monooxygenase	16	Core	hoi102_01730	hoi103_01770	hoi104_01789	hoi108_01858	hoi110_01717	hoi111_01323	hoi112_01751	hoi113_00508	hoi114_01847	hoi115_02030	hoi116_01771	hoi105_00085	hoi107_00084	hoi109_00506	hoi117_00505	hoi106_00084
1683	Peptidyl-tRNA hydrolase	16	Core	hoi102_01731	hoi103_01771	hoi104_01788	hoi108_01859	hoi110_01716	hoi111_01324	hoi112_01752	hoi113_00509	hoi114_01846	hoi115_02029	hoi116_01770	hoi105_00084	hoi107_00083	hoi109_00507	hoi117_00506	hoi106_00083
1684	Anaerobic C4-dicarboxylate transporter DcuB	16	Core	hoi102_01732	hoi103_01772	hoi104_01787	hoi108_01860	hoi110_01715	hoi111_01325	hoi112_01753	hoi113_00510	hoi114_01845	hoi115_02028	hoi116_01769	hoi105_00083	hoi107_00082	hoi109_00508	hoi117_00507	hoi106_00082
1685	hypothetical protein	11	Accessory	hoi102_01733	hoi103_01773	hoi104_01786	hoi108_01861	hoi110_01714	hoi111_01326	hoi112_01754	hoi113_00511	hoi114_01844	hoi115_02027	hoi116_01768					
1686	hypothetical protein	11	Accessory	hoi102_01734	hoi103_01774	hoi104_01785	hoi108_01862	hoi110_01713	hoi111_01327	hoi112_01755	hoi113_00512	hoi114_01843	hoi115_02026	hoi116_01767					
1687	Excalibur calcium-binding domain protein	11	Accessory	hoi102_01735	hoi103_01775	hoi104_01784	hoi108_01863	hoi110_01712	hoi111_01328	hoi112_01756	hoi113_00513	hoi114_01842	hoi115_02025	hoi116_01766					
1688	NAD-dependent protein deacetylase	16	Core	hoi102_01736	hoi103_01776	hoi104_01783	hoi108_01864	hoi110_01711	hoi111_01329	hoi112_01757	hoi113_00514	hoi114_01841	hoi115_02024	hoi116_01765	hoi105_00082	hoi107_00081	hoi109_00509	hoi117_00508	hoi106_00081
1689	Peptide chain release factor 3	16	Core	hoi102_01737	hoi103_01777	hoi104_01782	hoi108_01865	hoi110_01710	hoi111_01330	hoi112_01758	hoi113_00515	hoi114_01840	hoi115_02023	hoi116_01764	hoi105_00081	hoi107_00080	hoi109_00510	hoi117_00509	hoi106_00080
1690	hypothetical protein	15	Accessory	hoi102_01738	hoi103_01778	hoi104_01781	hoi108_01866	hoi110_01709	hoi111_01331	hoi112_01759	hoi113_00516	hoi114_01839	hoi115_02022	hoi116_01763	hoi105_00080	hoi107_00079	hoi109_00511	hoi117_00510	
1691	hypothetical protein	16	Core	hoi102_01739	hoi103_01779	hoi104_01780	hoi108_01867	hoi110_01708	hoi111_01332	hoi112_01760	hoi113_00517	hoi114_01838	hoi115_02021	hoi116_01762	hoi105_00079	hoi107_00078	hoi109_00512	hoi117_00511	hoi106_00077
1692	Nicotinamide mononucleotide transporter	16	Core	hoi102_01740	hoi103_01780	hoi104_01779	hoi108_01868	hoi110_01707	hoi111_01333	hoi112_01761	hoi113_00518	hoi114_01837	hoi115_02020	hoi116_01761	hoi105_00078	hoi107_00077	hoi109_00513	hoi117_00512	hoi106_00076
1693	putative enoyl-CoA hydratase echA8	16	Core	hoi102_01741	hoi103_01781	hoi104_01778	hoi108_01869	hoi110_01706	hoi111_01334	hoi112_01762	hoi113_00519	hoi114_01836	hoi115_02019	hoi116_01760	hoi105_00077	hoi107_00076	hoi109_00514	hoi117_00513	hoi106_00075
1694	Polyphosphate kinase 2 (PPK2)	16	Core	hoi102_01742	hoi103_01782	hoi104_01777	hoi108_01870	hoi110_01705	hoi111_01335	hoi112_01763	hoi113_00520	hoi114_01835	hoi115_02018	hoi116_01759	hoi105_00076	hoi107_00075	hoi109_00515	hoi117_00514	hoi106_00074
1695	Putative monooxygenase YcnE	16	Core	hoi102_01743	hoi103_01783	hoi104_01776	hoi108_01871	hoi110_01704	hoi111_01336	hoi112_01764	hoi113_00521	hoi114_01834	hoi115_02017	hoi116_01758	hoi105_00075	hoi107_00074	hoi109_00516	hoi117_00515	hoi106_00073
1696	putative ABC transporter ATP-binding protein/M	16	Core	hoi102_01744	hoi103_01784	hoi104_01775	hoi108_01872	hoi110_01703	hoi111_01337	hoi112_01765	hoi113_00522	hoi114_01833	hoi115_02016	hoi116_01757	hoi105_00074	hoi107_00073	hoi109_00517	hoi117_00516	hoi106_00072
1697	4-diphosphocytidyl-2-C-methyl-D-erythritol kinas	16	Core	hoi102_01745	hoi103_01785	hoi104_01774	hoi108_01873	hoi110_01702	hoi111_01338	hoi112_01766	hoi113_00523	hoi114_01832	hoi115_02015	hoi116_01756	hoi105_00073	hoi107_00072	hoi109_00518	hoi117_00517	hoi106_00071
1698	Ribosomal RNA small subunit methyltransferase	16	Core	hoi102_01746	hoi103_01786	hoi104_01773	hoi108_01874	hoi110_01701	hoi111_01339	hoi112_01767	hoi113_00524	hoi114_01831	hoi115_02014	hoi116_01755	hoi105_00072	hoi107_00071	hoi109_00519	hoi117_00518	hoi106_00070
1699	Resuscitation-promoting factor Rpf2 precursor	16	Core	hoi102_01747	hoi103_01787	hoi104_01772	hoi108_01875	hoi110_01700	hoi111_01340	hoi112_01768	hoi113_00525	hoi114_01830	hoi115_02013	hoi116_01754	hoi105_00071	hoi107_00070	hoi109_00520	hoi117_00519	hoi106_00069
1700	Flavin mononucleotide phosphatase YbjI	16	Core	hoi102_01748	hoi103_01788	hoi104_01771	hoi108_01876	hoi110_01699	hoi111_01341	hoi112_01769	hoi113_00526	hoi114_01829	hoi115_02012	hoi116_01753	hoi105_00070	hoi107_00069	hoi109_00521	hoi117_00520	hoi106_00068
1701	putative deoxyribonuclease YcfH	16	Core	hoi102_01749	hoi103_01789	hoi104_01770	hoi108_01877	hoi110_01698	hoi111_01342	hoi112_01770	hoi113_00527	hoi114_01828	hoi115_02011	hoi116_01752	hoi105_00069	hoi107_00068	hoi109_00522	hoi117_00521	hoi106_00067
1702	Methionine--tRNA ligase	16	Core	hoi102_01750	hoi103_01790	hoi104_01769	hoi108_01878	hoi110_01697	hoi111_01343	hoi112_01771	hoi113_00528	hoi114_01827	hoi115_02010	hoi116_01751	hoi105_00068	hoi107_00067	hoi109_00523	hoi117_00522	hoi106_00066
1703	Proline iminopeptidase	16	Core	hoi102_01751	hoi103_01791	hoi104_01768	hoi108_01879	hoi110_01696	hoi111_01344	hoi112_01772	hoi113_00529	hoi114_01826	hoi115_02009	hoi116_01750	hoi105_00067	hoi107_00066	hoi109_00524	hoi117_00523	hoi106_00065
1704	Glycine betaine transporter BetP	16	Core	hoi102_01752	hoi103_01792	hoi104_01767	hoi108_01880	hoi110_01695	hoi111_01345	hoi112_01773	hoi113_00530	hoi114_01825	hoi115_02008	hoi116_01749	hoi105_00066	hoi107_00065	hoi109_00525	hoi117_00524	hoi106_00064
1705	Ribosomal RNA small subunit methyltransferase	16	Core	hoi102_01753	hoi103_01793	hoi104_01766	hoi108_01881	hoi110_01694	hoi111_01346	hoi112_01774	hoi113_00531	hoi114_01824	hoi115_02007	hoi116_01748	hoi105_00065	hoi107_00064	hoi109_00526	hoi117_00525	hoi106_00063
1706	putative dolichyl-phosphate-mannose--protein n	16	Core	hoi102_01754	hoi103_01794	hoi104_01765	hoi108_01882	hoi110_01693	hoi111_01347	hoi112_01775	hoi113_00532	hoi114_01823	hoi115_02006	hoi116_01747	hoi105_00064	hoi107_00063	hoi109_00527	hoi117_00526	hoi106_00062
1707	hypothetical protein	16	Core	hoi102_01755	hoi103_01795	hoi104_01764	hoi108_01883	hoi110_01692	hoi111_01348	hoi112_01776	hoi113_00533	hoi114_01822	hoi115_02005	hoi116_01746	hoi105_00063	hoi107_00062	hoi109_00528	hoi117_00527	hoi106_00061
1708	Putative oxidoreductase MhqP	16	Core	hoi102_01756	hoi103_01796	hoi104_01763	hoi108_01884	hoi110_01691	hoi111_01349	hoi112_01777	hoi113_00534	hoi114_01821	hoi115_02004	hoi116_01745	hoi105_00062	hoi107_00061	hoi109_00529	hoi117_00528	hoi106_00060
1709	Fructosamine kinase	16	Core	hoi102_01757	hoi103_01797	hoi104_01762	hoi108_01885	hoi110_01690	hoi111_01350	hoi112_01778	hoi113_00535	hoi114_01820	hoi115_02003	hoi116_01744	hoi105_00061	hoi107_00060	hoi109_00530	hoi117_00529	hoi106_00059
1710	hypothetical protein	16	Core	hoi102_01758	hoi103_01798	hoi104_01761	hoi108_01886	hoi110_01689	hoi111_01351	hoi112_01779	hoi113_00536	hoi114_01819	hoi115_02002	hoi116_01743	hoi105_00060	hoi107_00059	hoi109_00531	hoi117_00530	hoi106_00058
1711	hypothetical protein	16	Core	hoi102_01759	hoi103_01799	hoi104_01760	hoi108_01887	hoi110_01688	hoi111_01352	hoi112_01780	hoi113_00537	hoi114_01818	hoi115_02001	hoi116_01742	hoi105_00059	hoi107_00058	hoi109_00532	hoi117_00531	hoi106_00057
1712	Na+/Pi-cotransporter	16	Core	hoi102_01760	hoi103_01800	hoi104_01759	hoi108_01888	hoi110_01687	hoi111_01353	hoi112_01781	hoi113_00538	hoi114_01817	hoi115_02000	hoi116_01741	hoi105_00058	hoi107_00057	hoi109_00533	hoi117_00532	hoi106_00056
1713	hypothetical protein	16	Core	hoi102_01761	hoi103_01801	hoi104_01758	hoi108_01889	hoi110_01686	hoi111_01354	hoi112_01782	hoi113_00539	hoi114_01952	hoi115_01999	hoi116_01740	hoi105_00057	hoi107_00056	hoi109_00534	hoi117_00533	hoi106_00055
1714	hypothetical protein	16	Core	hoi102_01763	hoi103_01803	hoi104_01756	hoi108_01891	hoi110_01684	hoi111_01356	hoi112_01784	hoi113_00541	hoi114_01954	hoi115_01997	hoi116_01738	hoi105_00054	hoi107_00053	hoi109_00537	hoi117_00536	hoi106_00052
1715	Putative ribosomal N-acetyltransferase YdaF	16	Core	hoi102_01764	hoi103_01804	hoi104_01755	hoi108_01892	hoi110_01683	hoi111_01357	hoi112_01785	hoi113_00542	hoi114_01955	hoi115_01996	hoi116_01737	hoi105_00053	hoi107_00052	hoi109_00538	hoi117_00537	hoi106_00051
1716	Molybdopterin molybdenumtransferase	16	Core	hoi102_01765	hoi103_01805	hoi104_01754	hoi108_01893	hoi110_01682	hoi111_01358	hoi112_01786	hoi113_00543	hoi114_01956	hoi115_01995	hoi116_01736	hoi105_00052	hoi107_00051	hoi109_00539	hoi117_00538	hoi106_00050
1717	UTP--glucose-1-phosphate uridylyltransferase	16	Core	hoi102_01766	hoi103_01806	hoi104_01753	hoi108_01894	hoi110_01681	hoi111_01359	hoi112_01787	hoi113_00544	hoi114_01957	hoi115_01994	hoi116_01735	hoi105_00051	hoi107_00050	hoi109_00540	hoi117_00539	hoi106_00049
1718	5-formyltetrahydrofolate cyclo-ligase family prot	16	Core	hoi102_01767	hoi103_01807	hoi104_01752	hoi108_01895	hoi110_01680	hoi111_01360	hoi112_01788	hoi113_00545	hoi114_01958	hoi115_01993	hoi116_01734	hoi105_00050	hoi107_00049	hoi109_00541	hoi117_00540	hoi106_00048
1719	SAF domain protein	16	Core	hoi102_01768	hoi103_01808	hoi104_01751	hoi108_01896	hoi110_01679	hoi111_01361	hoi112_01789	hoi113_00546	hoi114_01959	hoi115_01992	hoi116_01733	hoi105_00049	hoi107_00048	hoi109_00542	hoi117_00541	hoi106_00047
1720	Large-conductance mechanosensitive channel	16	Core	hoi102_01769	hoi103_01809	hoi104_01750	hoi108_01897	hoi110_01678	hoi111_01362	hoi112_01790	hoi113_00547	hoi114_01960	hoi115_01991	hoi116_01732	hoi105_00048	hoi107_00047	hoi109_00543	hoi117_00542	hoi106_00046
1721	hypothetical protein	16	Core	hoi102_01770	hoi103_01810	hoi104_01749	hoi108_01898	hoi110_01677	hoi111_01363	hoi112_01791	hoi113_00548	hoi114_01961	hoi115_01990	hoi116_01731	hoi105_00047	hoi107_00046	hoi109_00544	hoi117_00543	hoi106_00045
1722	Molybdenum cofactor biosynthesis protein B	16	Core	hoi102_01771	hoi103_01811	hoi104_01748	hoi108_01899	hoi110_01676	hoi111_01364	hoi112_01792	hoi113_00549	hoi114_01962	hoi115_01989	hoi116_01730	hoi105_00046	hoi107_00045	hoi109_00545	hoi117_00544	hoi106_00044
1723	Periplasmic pH-dependent serine endoprotease	16	Core	hoi102_01772	hoi103_01812	hoi104_01747	hoi108_01900	hoi110_01675	hoi111_01365	hoi112_01793	hoi113_00550	hoi114_01963	hoi115_01988	hoi116_01729	hoi105_00045	hoi107_00044	hoi109_00546	hoi117_00545	hoi106_00043
1724	Signal transduction histidine-protein kinase/phos	16	Core	hoi102_01773	hoi103_01813	hoi104_01746	hoi108_01901	hoi110_01674	hoi111_01366	hoi112_01794	hoi113_00551	hoi114_01964	hoi115_01987	hoi116_01728	hoi105_00044	hoi107_00043	hoi109_00547	hoi117_00546	hoi106_00042
1725	Response regulator MprA	16	Core	hoi102_01774	hoi103_01814	hoi104_01745	hoi108_01902	hoi110_01673	hoi111_01367	hoi112_01795	hoi113_00552	hoi114_01965	hoi115_01986	hoi116_01727	hoi105_00043	hoi107_00042	hoi109_00548	hoi117_00547	hoi106_00041
1726	50S ribosomal protein L32	16	Core	hoi102_01775	hoi103_01815	hoi104_01744	hoi108_01903	hoi110_01672	hoi111_01368	hoi112_01796	hoi113_00553	hoi114_01966	hoi115_01985	hoi116_01726	hoi105_00042	hoi107_00041	hoi109_00549	hoi117_00548	hoi106_00040
1727	50S ribosomal protein L31 type B	16	Core	hoi102_01776	hoi103_01816	hoi104_01743	hoi108_01904	hoi110_01671	hoi111_01369	hoi112_01797	hoi113_00554	hoi114_01967	hoi115_01984	hoi116_01725	hoi105_00041	hoi107_00040	hoi109_00550	hoi117_00549	hoi106_00039
1728	50S ribosomal protein L28	16	Core	hoi102_01777	hoi103_01817	hoi104_01742	hoi108_01905	hoi110_01670	hoi111_01370	hoi112_01798	hoi113_00555	hoi114_01968	hoi115_01983	hoi116_01724	hoi105_00040	hoi107_00039	hoi109_00551	hoi117_00550	hoi106_00038
1729	50S ribosomal protein L33 2	16	Core	hoi102_01778	hoi103_01818	hoi104_01741	hoi108_01906	hoi110_01669	hoi111_01371	hoi112_01799	hoi113_00556	hoi114_01969	hoi115_01982	hoi116_01723	hoi105_00039	hoi107_00038	hoi109_00552	hoi117_00551	hoi106_00037
1730	30S ribosomal protein S14	16	Core	hoi102_01779	hoi103_01819	hoi104_01740	hoi108_01907	hoi110_01668	hoi111_01372	hoi112_01800	hoi113_00557	hoi114_01970	hoi115_01981	hoi116_01722	hoi105_00038	hoi107_00037	hoi109_00553	hoi117_00552	hoi106_00036
1731	30S ribosomal protein S18 1	16	Core	hoi102_01780	hoi103_01820	hoi104_01739	hoi108_01908	hoi110_01667	hoi111_01373	hoi112_01801	hoi113_00558	hoi114_01971	hoi115_01980	hoi116_01721	hoi105_00037	hoi107_00036	hoi109_00554	hoi117_00553	hoi106_00035
1732	Putative nucleotidyltransferase substrate binding	16	Core	hoi102_01781	hoi103_01821	hoi104_01738	hoi108_01909	hoi110_01666	hoi111_01374	hoi112_01802	hoi113_00559	hoi114_01972	hoi115_01979	hoi116_01720	hoi105_00036	hoi107_00035	hoi109_00555	hoi117_00554	hoi106_00034
1733	HTH-type transcriptional regulator AcrR	16	Core	hoi102_01782	hoi103_01822	hoi104_01737	hoi108_01910	hoi110_01665	hoi111_01375	hoi112_01803	hoi113_00560	hoi114_01973	hoi115_01978	hoi116_01719	hoi105_00035	hoi107_00034	hoi109_00556	hoi117_00555	hoi106_00033
1734	Citrate lyase subunit beta-like protein	16	Core	hoi102_01783	hoi103_01823	hoi104_01736	hoi108_01911	hoi110_01664	hoi111_01376	hoi112_01804	hoi113_00561	hoi114_01974	hoi115_01977	hoi116_01718	hoi105_00034	hoi107_00033	hoi109_00557	hoi117_00556	hoi106_00032
1735	Bifunctional purine biosynthesis protein PurH	16	Core	hoi102_01784	hoi103_01824	hoi104_01735	hoi108_01912	hoi110_01663	hoi111_01377	hoi112_01805	hoi113_00562	hoi114_01975	hoi115_01976	hoi116_01717	hoi105_00033	hoi107_00032	hoi109_00558	hoi117_00557	hoi106_00031
1736	Phosphoribosylglycinamide formyltransferase	16	Core	hoi102_01785	hoi103_01825	hoi104_01734	hoi108_01913	hoi110_01662	hoi111_01378	hoi112_01806	hoi113_00563	hoi114_01976	hoi115_01975	hoi116_01716	hoi105_00032	hoi107_00031	hoi109_00559	hoi117_00558	hoi106_00030
1737	hypothetical protein	16	Core	hoi102_01786	hoi103_01826	hoi104_01733	hoi108_01914	hoi110_01661	hoi111_01379	hoi112_01807	hoi113_00564	hoi114_01977	hoi115_01974	hoi116_01715	hoi105_00031	hoi107_00030	hoi109_00560	hoi117_00559	hoi106_00029
1738	Murein hydrolase activator NlpD precursor	16	Core	hoi102_01787	hoi103_01827	hoi104_01732	hoi108_01915	hoi110_01660	hoi111_01380	hoi112_01808	hoi113_00565	hoi114_01978	hoi115_01973	hoi116_01714	hoi105_00030	hoi107_00029	hoi109_00561	hoi117_00560	hoi106_00028
1739	ATP-dependent DNA helicase UvrD1	16	Core	hoi102_01788	hoi103_01828	hoi104_01731	hoi108_01916	hoi110_01659	hoi111_01381	hoi112_01809	hoi113_00566	hoi114_01979	hoi115_01972	hoi116_01713	hoi105_00029	hoi107_00028	hoi109_00562	hoi117_00561	hoi106_00027
1740	Intracellular chorismate mutase	16	Core	hoi102_01789	hoi103_01829	hoi104_01730	hoi108_01917	hoi110_01658	hoi111_01382	hoi112_01810	hoi113_00567	hoi114_01980	hoi115_01971	hoi116_01712	hoi105_00028	hoi107_00027	hoi109_00563	hoi117_00562	hoi106_00026
1741	Succinate-semialdehyde dehydrogenase [NADP(	16	Core	hoi102_01790	hoi103_01830	hoi104_01729	hoi108_01918	hoi110_01657	hoi111_01383	hoi112_01811	hoi113_00568	hoi114_01981	hoi115_01970	hoi116_01711	hoi105_00027	hoi107_00026	hoi109_00564	hoi117_00563	hoi106_00025
1742	PTS system mannose-specific EIIBCA component	16	Core	hoi102_01791	hoi103_01831	hoi104_01728	hoi108_01919	hoi110_01656	hoi111_01384	hoi112_01812	hoi113_00569	hoi114_01982	hoi115_01969	hoi116_01710	hoi105_00026	hoi107_00025	hoi109_00565	hoi117_00564	hoi106_00024
1743	hypothetical protein	16	Core	hoi102_01792	hoi103_01832	hoi104_01727	hoi108_01920	hoi110_01655	hoi111_01385	hoi112_01813	hoi113_00570	hoi114_01983	hoi115_01968	hoi116_01709	hoi105_00025	hoi107_00024	hoi109_00566	hoi117_00565	hoi106_00023
1744	Glucose-6-phosphate isomerase	16	Core	hoi102_01793	hoi103_01833	hoi104_01726	hoi108_01921	hoi110_01654	hoi111_01386	hoi112_01814	hoi113_00571	hoi114_01984	hoi115_01967	hoi116_01708	hoi105_00024	hoi107_00023	hoi109_00567	hoi117_00566	hoi106_00022
1745	putative transporter YycB	16	Core	hoi102_01794	hoi103_01834	hoi104_01725	hoi108_01922	hoi110_01653	hoi111_01387	hoi112_01815	hoi113_00572	hoi114_01985	hoi115_01966	hoi116_01707	hoi105_00023	hoi107_00022	hoi109_00568	hoi117_00567	hoi106_00021
1746	Endonuclease 8 1	16	Core	hoi102_01795	hoi103_01835	hoi104_01724	hoi108_01923	hoi110_01652	hoi111_01388	hoi112_01816	hoi113_00573	hoi114_01986	hoi115_01965	hoi116_01706	hoi105_00022	hoi107_00021	hoi109_00569	hoi117_00568	hoi106_00020
1747	putative ATP-dependent helicase Lhr	16	Core	hoi102_01796	hoi103_01836	hoi104_01723	hoi108_01924	hoi110_01651	hoi111_01389	hoi112_01817	hoi113_00574	hoi114_01987	hoi115_01964	hoi116_01705	hoi105_00021	hoi107_00020	hoi109_00570	hoi117_00569	hoi106_00019
1748	3'-phosphoadenosine 5'-phosphate phosphatase	16	Core	hoi102_01797	hoi103_01837	hoi104_01722	hoi108_01925	hoi110_01650	hoi111_01390	hoi112_01818	hoi113_00575	hoi114_01988	hoi115_01963	hoi116_01704	hoi105_00020	hoi107_00019	hoi109_00571	hoi117_00570	hoi106_00018
1749	Thymidylate synthase	16	Core	hoi102_01798	hoi103_01838	hoi104_01721	hoi108_01926	hoi110_01649	hoi111_01391	hoi112_01819	hoi113_00576	hoi114_01989	hoi115_01962	hoi116_01703	hoi105_00019	hoi107_00018	hoi109_00572	hoi117_00571	hoi106_00017
1750	Dihydrofolate reductase	16	Core	hoi102_01799	hoi103_01839	hoi104_01720	hoi108_01927	hoi110_01648	hoi111_01392	hoi112_01820	hoi113_00577	hoi114_01990	hoi115_01961	hoi116_01702	hoi105_00018	hoi107_00017	hoi109_00573	hoi117_00572	hoi106_00016
1751	Mycoredoxin 1	16	Core	hoi102_01800	hoi103_01840	hoi104_01719	hoi108_01928	hoi110_01647	hoi111_01393	hoi112_01821	hoi113_00578	hoi114_01991	hoi115_01960	hoi116_01701	hoi105_00017	hoi107_00016	hoi109_00574	hoi117_00573	hoi106_00015
1752	precorrin 6A synthase	16	Core	hoi102_01801	hoi103_01841	hoi104_01718	hoi108_01929	hoi110_01646	hoi111_01394	hoi112_01822	hoi113_00579	hoi114_01992	hoi115_01959	hoi116_01700	hoi105_00016	hoi107_00015	hoi109_00575	hoi117_00574	hoi106_00014
1753	hypothetical protein	16	Core	hoi102_01802	hoi103_01842	hoi104_01717	hoi108_01930	hoi110_01645	hoi111_01395	hoi112_01823	hoi113_00580	hoi114_01993	hoi115_01958	hoi116_01699	hoi105_00015	hoi107_00014	hoi109_00576	hoi117_00575	hoi106_00013
1754	hypothetical protein	16	Core	hoi102_01803	hoi103_01843	hoi104_01716	hoi108_01931	hoi110_01644	hoi111_01396	hoi112_01824	hoi113_00581	hoi114_01994	hoi115_01957	hoi116_01698	hoi105_00014	hoi107_00013	hoi109_00577	hoi117_00576	hoi106_00012
1755	glutathione-regulated potassium-efflux system p	16	Core	hoi102_01805	hoi103_01845	hoi104_01714	hoi108_01933	hoi110_01642	hoi111_01398	hoi112_01826	hoi113_00583	hoi114_01996	hoi115_01955	hoi116_01696	hoi105_00012	hoi107_00011	hoi109_00579	hoi117_00578	hoi106_00008
1756	hypothetical protein	16	Core	hoi102_01806	hoi103_01846	hoi104_01713	hoi108_01934	hoi110_01641	hoi111_01399	hoi112_01827	hoi113_00584	hoi114_01997	hoi115_01954	hoi116_01695	hoi105_00011	hoi107_00010	hoi109_00580	hoi117_00579	hoi106_00007
1757	Cation/acetate symporter ActP	16	Core	hoi102_01807	hoi103_01847	hoi104_01712	hoi108_01935	hoi110_01640	hoi111_01400	hoi112_01828	hoi113_00585	hoi114_01998	hoi115_01953	hoi116_01694	hoi105_00010	hoi107_00009	hoi109_00581	hoi117_00580	hoi106_00006

1758	hypothetical protein	16	Core	hoi102_01808	hoi103_01848	hoi104_01711	hoi108_01936	hoi110_01639	hoi111_01401	hoi112_01829	hoi113_00586	hoi114_01999	hoi115_01952	hoi116_01693	hoi105_00009	hoi107_00008	hoi109_00582	hoi117_00581	hoi106_00005
1759	Calcium-transporting ATPase 1	16	Core	hoi102_01809	hoi103_01849	hoi104_01710	hoi108_01937	hoi110_01638	hoi111_01402	hoi112_01830	hoi113_00587	hoi114_02000	hoi115_01951	hoi116_01692	hoi105_00008	hoi107_00007	hoi109_00583	hoi117_00582	hoi106_00004
1760	putative oxidoreductase/MSMEI_2347	16	Core	hoi102_01810	hoi103_01850	hoi104_01709	hoi108_01938	hoi110_01637	hoi111_01403	hoi112_01831	hoi113_00588	hoi114_02001	hoi115_01950	hoi116_01691	hoi105_00007	hoi107_00006	hoi109_00584	hoi117_00583	hoi106_00003
1761	putative ABC transporter ATP-binding protein Yb	16	Core	hoi102_01811	hoi103_01851	hoi104_01708	hoi108_01939	hoi110_01636	hoi111_01404	hoi112_01832	hoi113_00589	hoi114_02002	hoi115_01949	hoi116_01690	hoi105_00006	hoi107_00005	hoi109_00585	hoi117_00584	hoi106_00002
1762	ABC-2 family transporter protein	16	Core	hoi102_01812	hoi103_01852	hoi104_01707	hoi108_01940	hoi110_01635	hoi111_01405	hoi112_01833	hoi113_00590	hoi114_02003	hoi115_01948	hoi116_01689	hoi105_00005	hoi107_00004	hoi109_00586	hoi117_00585	hoi106_00001
1763	Acetyl-coenzyme A carboxylase carboxyl transfe	16	Core	hoi102_01813	hoi103_01853	hoi104_01706	hoi108_01941	hoi110_01634	hoi111_01406	hoi112_01834	hoi113_00591	hoi114_02004	hoi115_01947	hoi116_01688	hoi105_00004	hoi107_00003	hoi109_00587	hoi117_00586	hoi106_02079
1764	FK506-binding protein	16	Core	hoi102_01814	hoi103_01854	hoi104_01705	hoi108_01942	hoi110_01633	hoi111_01407	hoi112_01835	hoi113_00592	hoi114_02005	hoi115_01946	hoi116_01687	hoi105_00003	hoi107_00002	hoi109_00588	hoi117_00587	hoi106_02080
1765	Citrate synthase 1	16	Core	hoi102_01815	hoi103_01855	hoi104_01704	hoi108_01943	hoi110_01632	hoi111_01408	hoi112_01836	hoi113_00593	hoi114_02006	hoi115_01945	hoi116_01686	hoi105_00002	hoi107_00001	hoi109_00589	hoi117_00588	hoi106_02081
1766	Phosphoserine aminotransferase	16	Core	hoi102_01816	hoi103_01856	hoi104_01703	hoi108_01944	hoi110_01631	hoi111_01409	hoi112_01837	hoi113_00594	hoi114_02007	hoi115_01944	hoi116_01685	hoi105_00001	hoi107_02132	hoi109_00590	hoi117_00589	hoi106_02082
1767	hypothetical protein	2	Accessory	hoi102_01817					hoi111_01678										
1768	putative hydrolase YxeP	16	Core	hoi102_01818	hoi103_01857	hoi104_01855	hoi108_01945	hoi110_01152	hoi111_01679	hoi112_01928	hoi113_01838	hoi114_01587	hoi115_01734	hoi116_01625	hoi105_01908	hoi107_01696	hoi109_01883	hoi117_01560	hoi106_01570
1769	NAD(P)H dehydrogenase (quinone)	16	Core	hoi102_01819	hoi103_01858	hoi104_01856	hoi108_01946	hoi110_01151	hoi111_01680	hoi112_01927	hoi113_01837	hoi114_01588	hoi115_01733	hoi116_01624	hoi105_01907	hoi107_01695	hoi109_01882	hoi117_01559	hoi106_01571
1770	Helix-turn-helix domain protein	16	Core	hoi102_01820	hoi103_01859	hoi104_01857	hoi108_01947	hoi110_01150	hoi111_01681	hoi112_01926	hoi113_01836	hoi114_01589	hoi115_01732	hoi116_01623	hoi105_01906	hoi107_01694	hoi109_01881	hoi117_01558	hoi106_01572
1771	2-methylcitrate dehydratase	16	Core	hoi102_01821	hoi103_01860	hoi104_01858	hoi108_01948	hoi110_01149	hoi111_01682	hoi112_01925	hoi113_01835	hoi114_01590	hoi115_01731	hoi116_01622	hoi105_01905	hoi107_01693	hoi109_01880	hoi117_01557	hoi106_01573
1772	Carboxyvinyl-carboxyphosphonate phosphorylm	16	Core	hoi102_01822	hoi103_01861	hoi104_01859	hoi108_01949	hoi110_01148	hoi111_01683	hoi112_01924	hoi113_01834	hoi114_01591	hoi115_01730	hoi116_01621	hoi105_01904	hoi107_01692	hoi109_01879	hoi117_01556	hoi106_01574
1773	2-methylcitrate synthase 2	16	Core	hoi102_01823	hoi103_01862	hoi104_01860	hoi108_01950	hoi110_01147	hoi111_01684	hoi112_01923	hoi113_01833	hoi114_01592	hoi115_01729	hoi116_01620	hoi105_01903	hoi107_01691	hoi109_01878	hoi117_01555	hoi106_01575
1774	2-oxoglutarate carboxylase small subunit	16	Core	hoi102_01824	hoi103_01863	hoi104_01861	hoi108_01951	hoi110_01146	hoi111_01685	hoi112_01922	hoi113_01832	hoi114_01593	hoi115_01728	hoi116_01619	hoi105_01902	hoi107_01690	hoi109_01877	hoi117_01554	hoi106_01576
1775	Biotin carboxylase	16	Core	hoi102_01825	hoi103_01864	hoi104_01862	hoi108_01952	hoi110_01145	hoi111_01686	hoi112_01921	hoi113_01831	hoi114_01594	hoi115_01727	hoi116_01618	hoi105_01901	hoi107_01689	hoi109_01876	hoi117_01553	hoi106_01577
1776	Putative thiosulfate sulfurtransferase SseA	16	Core	hoi102_01826	hoi103_01865	hoi104_01863	hoi108_01953	hoi110_01144	hoi111_01687	hoi112_01920	hoi113_01830	hoi114_01595	hoi115_01726	hoi116_01617	hoi105_01900	hoi107_01688	hoi109_01875	hoi117_01552	hoi106_01578
1777	hypothetical protein	16	Core	hoi102_01827	hoi103_01866	hoi104_01864	hoi108_01954	hoi110_01143	hoi111_01688	hoi112_01919	hoi113_01829	hoi114_01596	hoi115_01725	hoi116_01616	hoi105_01899	hoi107_01687	hoi109_01874	hoi117_01551	hoi106_01579
1778	hypothetical protein	16	Core	hoi102_01828	hoi103_01867	hoi104_01865	hoi108_01955	hoi110_01142	hoi111_01689	hoi112_01918	hoi113_01828	hoi114_01597	hoi115_01724	hoi116_01615	hoi105_01898	hoi107_01686	hoi109_01873	hoi117_01550	hoi106_01580
1779	hypothetical protein	16	Core	hoi102_01829	hoi103_01868	hoi104_01866	hoi108_01956	hoi110_01141	hoi111_01690	hoi112_01917	hoi113_01827	hoi114_01598	hoi115_01723	hoi116_01614	hoi105_01897	hoi107_01685	hoi109_01872	hoi117_01549	hoi106_01581
1780	Maf-like protein YceF	16	Core	hoi102_01830	hoi103_01869	hoi104_01867	hoi108_01957	hoi110_01140	hoi111_01691	hoi112_01916	hoi113_01826	hoi114_01599	hoi115_01722	hoi116_01613	hoi105_01896	hoi107_01684	hoi109_01871	hoi117_01548	hoi106_01582
1781	OPT oligopeptide transporter protein	16	Core	hoi102_01831	hoi103_01870	hoi104_01868	hoi108_01958	hoi110_01139	hoi111_01692	hoi112_01915	hoi113_01825	hoi114_01600	hoi115_01721	hoi116_01612	hoi105_01895	hoi107_01683	hoi109_01870	hoi117_01547	hoi106_01583
1782	hypothetical protein	16	Core	hoi102_01832	hoi103_01871	hoi104_01869	hoi108_01959	hoi110_01138	hoi111_01693	hoi112_01914	hoi113_01824	hoi114_01601	hoi115_01720	hoi116_01611	hoi105_01894	hoi107_01682	hoi109_01869	hoi117_01546	hoi106_01584
1783	putative propionyl-CoA carboxylase beta chain 5	16	Core	hoi102_01833	hoi103_01872	hoi104_01870	hoi108_01960	hoi110_01137	hoi111_01694	hoi112_01913	hoi113_01823	hoi114_01602	hoi115_01719	hoi116_01610	hoi105_01893	hoi107_01681	hoi109_01868	hoi117_01545	hoi106_01585
1784	putative propionyl-CoA carboxylase beta chain 5	16	Core	hoi102_01834	hoi103_01873	hoi104_01871	hoi108_01961	hoi110_01136	hoi111_01695	hoi112_01912	hoi113_01822	hoi114_01603	hoi115_01718	hoi116_01609	hoi105_01892	hoi107_01680	hoi109_01867	hoi117_01544	hoi106_01586
1785	Bifunctional ligase/repressor BirA	16	Core	hoi102_01835	hoi103_01874	hoi104_01872	hoi108_01962	hoi110_01135	hoi111_01696	hoi112_01911	hoi113_01821	hoi114_01604	hoi115_01717	hoi116_01608	hoi105_01891	hoi107_01679	hoi109_01866	hoi117_01543	hoi106_01587
1786	hypothetical protein	16	Core	hoi102_01836	hoi103_01875	hoi104_01873	hoi108_01963	hoi110_01134	hoi111_01697	hoi112_01910	hoi113_01820	hoi114_01605	hoi115_01716	hoi116_01607	hoi105_01890	hoi107_01678	hoi109_01865	hoi117_01542	hoi106_01588
1787	N5-carboxyaminoimidazole ribonucleotide synth	16	Core	hoi102_01837	hoi103_01876	hoi104_01874	hoi108_01964	hoi110_01133	hoi111_01698	hoi112_01909	hoi113_01819	hoi114_01606	hoi115_01715	hoi116_01606	hoi105_01889	hoi107_01677	hoi109_01864	hoi117_01541	hoi106_01589
1788	N5-carboxyaminoimidazole ribonucleotide muta	16	Core	hoi102_01838	hoi103_01877	hoi104_01875	hoi108_01965	hoi110_01132	hoi111_01699	hoi112_01908	hoi113_01818	hoi114_01607	hoi115_01714	hoi116_01605	hoi105_01888	hoi107_01676	hoi109_01863	hoi117_01540	hoi106_01590
1789	hypothetical protein	16	Core	hoi102_01839	hoi103_01878	hoi104_01876	hoi108_01966	hoi110_01131	hoi111_01700	hoi112_01907	hoi113_01817	hoi114_01608	hoi115_01713	hoi116_01604	hoi105_01887	hoi107_01675	hoi109_01862	hoi117_01539	hoi106_01591
1790	hypothetical protein	16	Core	hoi102_01840	hoi103_01879	hoi104_01877	hoi108_01967	hoi110_01130	hoi111_01701	hoi112_01906	hoi113_01816	hoi114_01609	hoi115_01712	hoi116_01603	hoi105_01886	hoi107_01674	hoi109_01861	hoi117_01538	hoi106_01592
1791	Trehalose transport system permease protein Su	16	Core	hoi102_01841	hoi103_01880	hoi104_01878	hoi108_01968	hoi110_01129	hoi111_01702	hoi112_01905	hoi113_01815	hoi114_01610	hoi115_01711	hoi116_01602	hoi105_01885	hoi107_01673	hoi109_01860	hoi117_01537	hoi106_01593
1792	Trehalose transport system permease protein Su	16	Core	hoi102_01842	hoi103_01881	hoi104_01879	hoi108_01969	hoi110_01128	hoi111_01703	hoi112_01904	hoi113_01814	hoi114_01611	hoi115_01710	hoi116_01601	hoi105_01884	hoi107_01672	hoi109_01859	hoi117_01536	hoi106_01594
1793	putative ABC transporter-binding protein precur	16	Core	hoi102_01843	hoi103_01882	hoi104_01880	hoi108_01970	hoi110_01127	hoi111_01704	hoi112_01903	hoi113_01813	hoi114_01612	hoi115_01709	hoi116_01600	hoi105_01883	hoi107_01671	hoi109_01858	hoi117_01535	hoi106_01595
1794	sn-glycerol-3-phosphate import ATP-binding prot	16	Core	hoi102_01844	hoi103_01883	hoi104_01881	hoi108_01971	hoi110_01126	hoi111_01705	hoi112_01902	hoi113_01812	hoi114_01613	hoi115_01708	hoi116_01599	hoi105_01882	hoi107_01670	hoi109_01857	hoi117_01534	hoi106_01596
1795	hypothetical protein	15	Accessory	hoi102_01845	hoi103_01884	hoi104_01882	hoi108_01972	hoi110_01125	hoi111_01706	hoi112_01901	hoi113_01811	hoi114_01614	hoi115_01707	hoi116_01598	hoi105_01881	hoi107_01669	hoi109_01856	hoi117_01533	
1796	Transcriptional regulator LytR	16	Core	hoi102_01846	hoi103_01885	hoi104_01883	hoi108_01973	hoi110_01124	hoi111_01707	hoi112_01900	hoi113_01810	hoi114_01615	hoi115_01706	hoi116_01597	hoi105_01880	hoi107_01668	hoi109_01855	hoi117_01532	hoi106_02128
1797	dTDP-4-dehydrorhamnose reductase	16	Core	hoi102_01847	hoi103_01886	hoi104_01884	hoi108_01974	hoi110_01123	hoi111_01708	hoi112_01899	hoi113_01809	hoi114_01616	hoi115_01705	hoi116_01596	hoi105_01879	hoi107_01667	hoi109_01854	hoi117_01531	hoi106_01945
1798	N-acetylglucosaminyl-diphospho-decaprenol L-rh	16	Core	hoi102_01848	hoi103_01887	hoi104_01885	hoi108_01975	hoi110_01122	hoi111_01709	hoi112_01898	hoi113_01808	hoi114_01617	hoi115_01704	hoi116_01595	hoi105_01878	hoi107_01666	hoi109_01853	hoi117_01530	hoi106_01944
1799	D-glycero-alpha-D-manno-heptose 1-phosphate	16	Core	hoi102_01849	hoi103_01888	hoi104_01886	hoi108_01976	hoi110_01121	hoi111_01710	hoi112_01897	hoi113_01807	hoi114_01618	hoi115_01703	hoi116_01594	hoi105_01877	hoi107_01665	hoi109_01852	hoi117_01529	hoi106_01943
1800	Transcriptional regulator WhiB2	16	Core	hoi102_01850	hoi103_01889	hoi104_01887	hoi108_01977	hoi110_01120	hoi111_01711	hoi112_01896	hoi113_01806	hoi114_01619	hoi115_01702	hoi116_01593	hoi105_01876	hoi107_01664	hoi109_01851	hoi117_01528	hoi106_01942
1801	hypothetical protein	16	Core	hoi102_01851	hoi103_01890	hoi104_01888	hoi108_01978	hoi110_01119	hoi111_01712	hoi112_01895	hoi113_01805	hoi114_01620	hoi115_01701	hoi116_01592	hoi105_01875	hoi107_01663	hoi109_01850	hoi117_01527	hoi106_01941
1802	hypothetical protein	16	Core	hoi102_01852	hoi103_01891	hoi104_01889	hoi108_01979	hoi110_01118	hoi111_01713	hoi112_01894	hoi113_01804	hoi114_01621	hoi115_01700	hoi116_01591	hoi105_01874	hoi107_01662	hoi109_01849	hoi117_01526	hoi106_01940
1803	Phosphomannomutase/phosphoglucomutase	16	Core	hoi102_01853	hoi103_01892	hoi104_01890	hoi108_01980	hoi110_01117	hoi111_01714	hoi112_01893	hoi113_01803	hoi114_01622	hoi115_01699	hoi116_01590	hoi105_01873	hoi107_01661	hoi109_01848	hoi117_01525	hoi106_01939
1804	hypothetical protein	16	Core	hoi102_01854	hoi103_01893	hoi104_01891	hoi108_01981	hoi110_01116	hoi111_01715	hoi112_01892	hoi113_01802	hoi114_01623	hoi115_01698	hoi116_01589	hoi105_01872	hoi107_01660	hoi109_01847	hoi117_01524	hoi106_01938
1805	Mannose-6-phosphate isomerase	16	Core	hoi102_01855	hoi103_01894	hoi104_01892	hoi108_01982	hoi110_01115	hoi111_01716	hoi112_01891	hoi113_01801	hoi114_01624	hoi115_01697	hoi116_01588	hoi105_01871	hoi107_01659	hoi109_01846	hoi117_01523	hoi106_01937
1806	hypothetical protein	16	Core	hoi102_01856	hoi103_01895	hoi104_01893	hoi108_01983	hoi110_01114	hoi111_01717	hoi112_01890	hoi113_01800	hoi114_01625	hoi115_01696	hoi116_01587	hoi105_01870	hoi107_01658	hoi109_01845	hoi117_01522	hoi106_01936
1807	hypothetical protein	16	Core	hoi102_01857	hoi103_01896	hoi104_01894	hoi108_01984	hoi110_01113	hoi111_01718	hoi112_01889	hoi113_01799	hoi114_01626	hoi115_01695	hoi116_01586	hoi105_01869	hoi107_01657	hoi109_01844	hoi117_01521	hoi106_01935
1808	Thymidylate kinase	16	Core	hoi102_01858	hoi103_01897	hoi104_01895	hoi108_01985	hoi110_01112	hoi111_01719	hoi112_01888	hoi113_01798	hoi114_01627	hoi115_01694	hoi116_01585	hoi105_01868	hoi107_01656	hoi109_01843	hoi117_01520	hoi106_01934
1809	DNA-binding response regulator MtrA	16	Core	hoi102_01859	hoi103_01898	hoi104_01896	hoi108_01986	hoi110_01111	hoi111_01720	hoi112_01887	hoi113_01797	hoi114_01628	hoi115_01693	hoi116_01584	hoi105_01867	hoi107_01655	hoi109_01842	hoi117_01519	hoi106_01933
1810	Sensor histidine kinase MtrB	16	Core	hoi102_01860	hoi103_01899	hoi104_01897	hoi108_01987	hoi110_01110	hoi111_01721	hoi112_01886	hoi113_01796	hoi114_01629	hoi115_01692	hoi116_01583	hoi105_01866	hoi107_01654	hoi109_01841	hoi117_01518	hoi106_01932
1811	Lipoprotein LpqB precursor	16	Core	hoi102_01861	hoi103_01900	hoi104_01898	hoi108_01988	hoi110_01109	hoi111_01722	hoi112_01885	hoi113_01795	hoi114_01630	hoi115_01691	hoi116_01582	hoi105_01865	hoi107_01653	hoi109_01840	hoi117_01517	hoi106_01931
1812	DNA utilization protein GntX	16	Core	hoi102_01862	hoi103_01901	hoi104_01899	hoi108_01989	hoi110_01108	hoi111_01723	hoi112_01884	hoi113_01794	hoi114_01631	hoi115_01690	hoi116_01581	hoi105_01864	hoi107_01652	hoi109_01839	hoi117_01516	hoi106_01930
1813	Light-repressed protein A	16	Core	hoi102_01863	hoi103_01902	hoi104_01900	hoi108_01990	hoi110_01107	hoi111_01724	hoi112_01883	hoi113_01793	hoi114_01632	hoi115_01689	hoi116_01580	hoi105_01863	hoi107_01651	hoi109_01838	hoi117_01515	hoi106_01929
1814	preprotein translocase subunit SecA	16	Core	hoi102_01864	hoi103_01903	hoi104_01901	hoi108_01991	hoi110_01106	hoi111_01725	hoi112_01882	hoi113_01792	hoi114_01633	hoi115_01688	hoi116_01579	hoi105_01862	hoi107_01650	hoi109_01837	hoi117_01514	hoi106_01928
1815	hypothetical protein	16	Core	hoi102_01865	hoi103_01904	hoi104_01902	hoi108_01992	hoi110_01105	hoi111_01726	hoi112_01881	hoi113_01791	hoi114_01634	hoi115_01687	hoi116_01578	hoi105_01861	hoi107_01649	hoi109_01836	hoi117_01513	hoi106_01927
1816	dUMP phosphatase	16	Core	hoi102_01866	hoi103_01905	hoi104_01903	hoi108_01993	hoi110_01104	hoi111_01727	hoi112_01880	hoi113_01790	hoi114_01635	hoi115_01686	hoi116_01577	hoi105_01860	hoi107_01648	hoi109_01835	hoi117_01512	hoi106_01926
1817	hypothetical protein	16	Core	hoi102_01867	hoi103_01906	hoi104_01904	hoi108_01994	hoi110_01103	hoi111_01728	hoi112_01879	hoi113_01789	hoi114_01636	hoi115_01685	hoi116_01576	hoi105_01859	hoi107_01647	hoi109_01834	hoi117_01511	hoi106_01925
1818	Stearoyl-CoA 9-desaturase electron transfer part	16	Core	hoi102_01868	hoi103_01907	hoi104_01905	hoi108_01995	hoi110_01102	hoi111_01729	hoi112_01878	hoi113_01788	hoi114_01637	hoi115_01684	hoi116_01575	hoi105_01858	hoi107_01646	hoi109_01833	hoi117_01510	hoi106_01924
1819	Stearoyl-CoA 9-desaturase	16	Core	hoi102_01869	hoi103_01908	hoi104_01906	hoi108_01996	hoi110_01101	hoi111_01730	hoi112_01877	hoi113_01787	hoi114_01638	hoi115_01683	hoi116_01574	hoi105_01857	hoi107_01645	hoi109_01832	hoi117_01509	hoi106_01923
1820	Voltage-gated ClC-type chloride channel ClcB	16	Core	hoi102_01870	hoi103_01909	hoi104_01907	hoi108_01997	hoi110_01100	hoi111_01731	hoi112_01876	hoi113_01786	hoi114_01639	hoi115_01682	hoi116_01573	hoi105_01856	hoi107_01644	hoi109_01831	hoi117_01508	hoi106_01922
1821	Putative ribosome biogenesis GTPase RsgA	16	Core	hoi102_01871	hoi103_01910	hoi104_01908	hoi108_01998	hoi110_01099	hoi111_01732	hoi112_01875	hoi113_01785	hoi114_01640	hoi115_01681	hoi116_01572	hoi105_01855	hoi107_01643	hoi109_01830	hoi117_01507	hoi106_01921
1822	3-phosphoshikimate 1-carboxyvinyltransferase	16	Core	hoi102_01872	hoi103_01911	hoi104_01909	hoi108_01999	hoi110_01098	hoi111_01733	hoi112_01874	hoi113_01784	hoi114_01641	hoi115_01680	hoi116_01571	hoi105_01854	hoi107_01642	hoi109_01829	hoi117_01506	hoi106_01920
1823	Putative SOS response-associated peptidase Yed	16	Core	hoi102_01873	hoi103_01912	hoi104_01910	hoi108_02000	hoi110_01097	hoi111_01734	hoi112_01873	hoi113_01783	hoi114_01642	hoi115_01679	hoi116_01570	hoi105_01853	hoi107_01641	hoi109_01828	hoi117_01505	hoi106_01919
1824	Cys-tRNA(Pro)/Cys-tRNA(Cys) deacylase YbaK	16	Core	hoi102_01874	hoi103_01913	hoi104_01911	hoi108_02001	hoi110_01096	hoi111_01735	hoi112_01872	hoi113_01782	hoi114_01643	hoi115_01678	hoi116_01569	hoi105_01852	hoi107_01640	hoi109_01827	hoi117_01504	hoi106_01918
1825	Iron-dependent repressor IdeR	16	Core	hoi102_01875	hoi103_01914	hoi104_01912	hoi108_02002	hoi110_01095	hoi111_01736	hoi112_01871	hoi113_01781	hoi114_01644	hoi115_01677	hoi116_01568	hoi105_01851	hoi107_01639	hoi109_01826	hoi117_01503	hoi106_01917
1826	ECF RNA polymerase sigma factor SigH	16	Core	hoi102_01876	hoi103_01915	hoi104_01913	hoi108_02003	hoi110_01094	hoi111_01737	hoi112_01870	hoi113_01780	hoi114_01645	hoi115_01676	hoi116_01567	hoi105_01850	hoi107_01638	hoi109_01825	hoi117_01502	hoi106_01916
1827	Anti-sigma factor RshA	16	Core	hoi102_01877	hoi103_01916	hoi104_01914	hoi108_02004	hoi110_01093	hoi111_01738	hoi112_01869	hoi113_01779	hoi114_01646	hoi115_01675	hoi116_01566	hoi105_01849	hoi107_01637	hoi109_01824	hoi117_01501	hoi106_01915
1828	Transcriptional regulator WhiB1	16	Core	hoi102_01878	hoi103_01917	hoi104_01915	hoi108_02005	hoi110_01092	hoi111_01739	hoi112_01868	hoi113_01778	hoi114_01647	hoi115_01674	hoi116_01565	hoi105_01848	hoi107_01636	hoi109_01823	hoi117_01500	hoi106_01914
1829	Diacylglycerol kinase	16	Core	hoi102_01879	hoi103_01918	hoi104_01916	hoi108_02006	hoi110_01091	hoi111_01740	hoi112_01867	hoi113_01777	hoi114_01648	hoi115_01673	hoi116_01564	hoi105_01847	hoi107_01635	hoi109_01822	hoi117_01499	hoi106_01913
1830	hypothetical protein	16	Core	hoi102_01880	hoi103_01919	hoi104_01917	hoi108_02007	hoi110_01090	hoi111_01741	hoi112_01866	hoi113_01776	hoi114_01649	hoi115_01672	hoi116_01563	hoi105_01846	hoi107_01634	hoi109_01821	hoi117_01498	hoi106_01912
1831	hypothetical protein	16	Core	hoi102_01881	hoi103_01920	hoi104_01918	hoi108_02008	hoi110_01089	hoi111_01742	hoi112_01865	hoi113_01775	hoi114_01650	hoi115_01671	hoi116_01562	hoi105_01845	hoi107_01633	hoi109_01820	hoi117_01497	hoi106_01911
1832	DEAD-box ATP-dependent RNA helicase CshA	16	Core	hoi102_01882	hoi103_01921	hoi104_01919	hoi108_02009	hoi110_01088	hoi111_01743	hoi112_01864	hoi113_01774	hoi114_01651	hoi115_01670	hoi116_01561	hoi105_01844	hoi107_01632	hoi109_01819	hoi117_01496	hoi106_01910
1833	hypothetical protein	16	Core	hoi102_01883	hoi103_01922	hoi104_01920	hoi108_02010	hoi110_01087	hoi111_01744	hoi112_01863	hoi113_01773	hoi114_01652	hoi115_01669	hoi116_01560	hoi105_01843	hoi107_01631	hoi109_01818	hoi117_01495	hoi106_01909
1834	hypothetical protein	16	Core	hoi102_01884	hoi103_01923	hoi104_01921	hoi108_02011	hoi110_01086	hoi111_01745	hoi112_01862	hoi113_01772	hoi114_01653	hoi115_01668	hoi116_01559	hoi105_01842	hoi107_01630	hoi109_01817	hoi117_01494	hoi106_01908
1835	putative adenylyltransferase/sulfurtransferase M	16	Core	hoi102_01885	hoi103_01924	hoi104_01922	hoi108_02012	hoi110_01085	hoi111_01746	hoi112_01861	hoi113_01771	hoi114_01654	hoi115_01667	hoi116_01558	hoi105_01841	hoi107_01629	hoi109_01816	hoi117_01493	hoi106_01907
1836	hypothetical protein	16	Core	hoi102_01886	hoi103_01925	hoi104_01923	hoi108_02013	hoi110_01084	hoi111_01747	hoi112_01860	hoi113_01770	hoi114_01655	hoi115_01666	hoi116_01557	hoi105_01840	hoi107_01628	hoi109_01815	hoi117_01492	hoi106_01906
1837	ATP-dependent DNA helicase PcrA	16	Core	hoi102_01887	hoi103_01926	hoi104_01924	hoi108_02014	hoi110_01083	hoi111_01748	hoi112_01859	hoi113_01769	hoi114_01656	hoi115_01665	hoi116_01556	hoi105_01839	hoi107_01627	hoi109_01814	hoi117_01491	hoi106_01905

1838	ATP-dependent DNA helicase PcrA	16	Core			hoi102_01888	hoi103_01927	hoi104_01925	hoi108_02015	hoi110_01082	hoi111_01749	hoi112_01858	hoi113_01768	hoi114_01657	hoi115_01664	hoi116_01555	hoi105_01838	hoi107_01626	hoi109_01813	hoi117_01490	hoi106_01904
1839	Voltage-gated potassium channel Kch	16	Core			hoi102_01889	hoi103_01928	hoi104_01926	hoi108_02016	hoi110_01081	hoi111_01750	hoi112_01857	hoi113_01767	hoi114_01658	hoi115_01663	hoi116_01554	hoi105_01837	hoi107_01625	hoi109_01812	hoi117_01489	hoi106_01903
1840	NADH pyrophosphatase	16	Core			hoi102_01890	hoi103_01929	hoi104_01927	hoi108_02017	hoi110_01080	hoi111_01751	hoi112_01856	hoi113_01766	hoi114_01659	hoi115_01662	hoi116_01553	hoi105_01836	hoi107_01624	hoi109_01811	hoi117_01488	hoi106_01902
1841	ATP-dependent DNA helicase UvrD2	16	Core			hoi102_01891	hoi103_01930	hoi104_01928	hoi108_02018	hoi110_01079	hoi111_01752	hoi112_01855	hoi113_01765	hoi114_01660	hoi115_01661	hoi116_01552	hoi105_01835	hoi107_01623	hoi109_01810	hoi117_01487	hoi106_01901
1842	hypothetical protein	16	Core			hoi102_01892	hoi103_01931	hoi104_01929	hoi108_02019	hoi110_01078	hoi111_01753	hoi112_01854	hoi113_01764	hoi114_01661	hoi115_01660	hoi116_01551	hoi105_01834	hoi107_01622	hoi109_01809	hoi117_01486	hoi106_01900
1843	SprT-like family protein	16	Core			hoi102_01893	hoi103_01932	hoi104_01930	hoi108_02020	hoi110_01077	hoi111_01754	hoi112_01853	hoi113_01763	hoi114_01662	hoi115_01659	hoi116_01550	hoi105_01833	hoi107_01621	hoi109_01808	hoi117_01485	hoi106_01899
1844	hypothetical protein	16	Core			hoi102_01894	hoi103_01933	hoi104_01931	hoi108_02021	hoi110_01076	hoi111_01755	hoi112_01852	hoi113_01762	hoi114_01663	hoi115_01658	hoi116_01549	hoi105_01832	hoi107_01620	hoi109_01807	hoi117_01484	hoi106_01898
1845	Lon protease 1	16	Core			hoi102_01895	hoi103_01934	hoi104_01932	hoi108_02022	hoi110_01075	hoi111_01756	hoi112_01851	hoi113_01761	hoi114_01664	hoi115_01657	hoi116_01548	hoi105_01831	hoi107_01619	hoi109_01806	hoi117_01483	hoi106_01897
1846	hypothetical protein	16	Core			hoi102_01896	hoi103_01935	hoi104_01933	hoi108_02023	hoi110_01074	hoi111_01757	hoi112_01850	hoi113_01760	hoi114_01665	hoi115_01656	hoi116_01547	hoi105_01830	hoi107_01618	hoi109_01805	hoi117_01482	hoi106_01896
1847	hypothetical protein	16	Core			hoi102_01897	hoi103_01936	hoi104_01934	hoi108_02024	hoi110_01073	hoi111_01758	hoi112_01849	hoi113_01759	hoi114_01666	hoi115_01655	hoi116_01546	hoi105_01829	hoi107_01617	hoi109_01804	hoi117_01481	hoi106_01895
1848	hypothetical protein	16	Core			hoi102_01898	hoi103_01937	hoi104_01935	hoi108_02025	hoi110_01072	hoi111_01759	hoi112_01848	hoi113_01758	hoi114_01667	hoi115_01654	hoi116_01545	hoi105_01828	hoi107_01616	hoi109_01803	hoi117_01480	hoi106_01894
1849	CAAX amino terminal protease self- immunity	16	Core			hoi102_01901	hoi103_01940	hoi104_01938	hoi108_02028	hoi110_01069	hoi111_01762	hoi112_01845	hoi113_01755	hoi114_01670	hoi115_01651	hoi116_01542	hoi105_02023	hoi107_01981	hoi109_01992	hoi117_01447	hoi106_02005
1850	Iron import ATP-binding/permease protein IrtB	16	Core			hoi102_01902	hoi103_01941	hoi104_01939	hoi108_02029	hoi110_01068	hoi111_01763	hoi112_01844	hoi113_01754	hoi114_01671	hoi115_01650	hoi116_01541	hoi105_02022	hoi107_01982	hoi109_01993	hoi117_01446	hoi106_02004
1851	Iron import ATP-binding/permease protein IrtA	16	Core			hoi102_01903	hoi103_01942	hoi104_01940	hoi108_02030	hoi110_01067	hoi111_01764	hoi112_01843	hoi113_01753	hoi114_01672	hoi115_01649	hoi116_01540	hoi105_02021	hoi107_01983	hoi109_01994	hoi117_01445	hoi106_02003
1852	Putative HMP/thiamine import ATP-binding prot	16	Core			hoi102_01904	hoi103_01943	hoi104_01941	hoi108_02031	hoi110_01066	hoi111_01765	hoi112_01842	hoi113_01752	hoi114_01673	hoi115_01648	hoi116_01539	hoi105_02020	hoi107_01984	hoi109_01995	hoi117_01444	hoi106_02002
1853	Energy-coupling factor transporter transmembra	16	Core			hoi102_01905	hoi103_01944	hoi104_01942	hoi108_02032	hoi110_01065	hoi111_01766	hoi112_01841	hoi113_01751	hoi114_01674	hoi115_01647	hoi116_01538	hoi105_02019	hoi107_01985	hoi109_01996	hoi117_01443	hoi106_02001
1854	hypothetical protein	16	Core			hoi102_01906	hoi103_01945	hoi104_01943	hoi108_02033	hoi110_01064	hoi111_01767	hoi112_01840	hoi113_01750	hoi114_01675	hoi115_01646	hoi116_01537	hoi105_02018	hoi107_01986	hoi109_01997	hoi117_01442	hoi106_02000
1855	hypothetical protein	16	Core			hoi102_01907	hoi103_01946	hoi104_01944	hoi108_02034	hoi110_01063	hoi111_01768	hoi112_01839	hoi113_01749	hoi114_01676	hoi115_01645	hoi116_01536	hoi105_02017	hoi107_01987	hoi109_01998	hoi117_01441	hoi106_01999
1856	NADH dehydrogenase	16	Core			hoi102_01908	hoi103_01947	hoi104_01945	hoi108_02035	hoi110_01062	hoi111_01769	hoi112_01838	hoi113_01748	hoi114_01677	hoi115_01644	hoi116_01535	hoi105_02016	hoi107_01988	hoi109_01999	hoi117_01440	hoi106_01998
1857	Phosphoserine phosphatase	16	Core			hoi102_01909	hoi103_00720	hoi104_00638	hoi108_00673	hoi110_01021	hoi111_01101	hoi112_02040	hoi113_02048	hoi114_02100	hoi115_00723	hoi116_01494	hoi105_01413	hoi107_01440	hoi109_01372	hoi117_01772	hoi106_01470
1858	hypothetical protein	16	Core			hoi102_01910	hoi103_00721	hoi104_00639	hoi108_00674	hoi110_01022	hoi111_01102	hoi112_02041	hoi113_02049	hoi114_02099	hoi115_00724	hoi116_01495	hoi105_01414	hoi107_01441	hoi109_01373	hoi117_01773	hoi106_01471
1859	hypothetical protein	16	Core			hoi102_01911	hoi103_00722	hoi104_00640	hoi108_00675	hoi110_01023	hoi111_01103	hoi112_02042	hoi113_02050	hoi114_02098	hoi115_00725	hoi116_01496	hoi105_01415	hoi107_01442	hoi109_01374	hoi117_01774	hoi106_01472
1860	Soluble epoxide hydrolase	16	Core			hoi102_01912	hoi103_00723	hoi104_00641	hoi108_00676	hoi110_01024	hoi111_01104	hoi112_02043	hoi113_02051	hoi114_02097	hoi115_00726	hoi116_01497	hoi105_01416	hoi107_01443	hoi109_01375	hoi117_01775	hoi106_01473
1861	Serine protease	16	Core			hoi102_01913	hoi103_00724	hoi104_00642	hoi108_00677	hoi110_01025	hoi111_01105	hoi112_02044	hoi113_02052	hoi114_02096	hoi115_00727	hoi116_01498	hoi105_01417	hoi107_01444	hoi109_01376	hoi117_01776	hoi106_01474
1862	putative NUDIX hydrolase	16	Core			hoi102_01914	hoi103_00725	hoi104_00643	hoi108_00678	hoi110_01026	hoi111_01106	hoi112_02045	hoi113_02053	hoi114_02095	hoi115_00728	hoi116_01499	hoi105_01418	hoi107_01445	hoi109_01377	hoi117_01777	hoi106_01475
1863	Thiol:disulfide interchange protein CycY precursc	16	Core			hoi102_01915	hoi103_00726	hoi104_00644	hoi108_00679	hoi110_01027	hoi111_01107	hoi112_02046	hoi113_02054	hoi114_02094	hoi115_00729	hoi116_01500	hoi105_01419	hoi107_01446	hoi109_01378	hoi117_01778	hoi106_01476
1864	Ultraviolet N-glycosylase/AP lyase	16	Core			hoi102_01916	hoi103_00727	hoi104_00645	hoi108_00680	hoi110_01028	hoi111_01108	hoi112_02047	hoi113_02055	hoi114_02093	hoi115_00730	hoi116_01501	hoi105_01420	hoi107_01447	hoi109_01379	hoi117_01779	hoi106_01477
1865	hypothetical protein	16	Core			hoi102_01917	hoi103_00728	hoi104_00646	hoi108_00681	hoi110_01029	hoi111_01109	hoi112_02048	hoi113_02056	hoi114_02092	hoi115_00731	hoi116_01502	hoi105_01421	hoi107_01448	hoi109_01380	hoi117_01780	hoi106_01478
1866	cAMP receptor protein	16	Core			hoi102_01918	hoi103_00729	hoi104_00647	hoi108_00682	hoi110_01030	hoi111_01110	hoi112_02049	hoi113_02057	hoi114_02091	hoi115_00732	hoi116_01503	hoi105_01422	hoi107_01449	hoi109_01381	hoi117_01781	hoi106_01479
1867	putative polyketide biosynthesis zinc-dependent	16	Core			hoi102_01919	hoi103_00730	hoi104_00648	hoi108_00683	hoi110_01031	hoi111_01111	hoi112_02050	hoi113_02058	hoi114_02090	hoi115_00733	hoi116_01504	hoi105_01423	hoi107_01450	hoi109_01382	hoi117_01782	hoi106_01480
1868	Putative reactive intermediate deaminase TdcF	16	Core			hoi102_01920	hoi103_00731	hoi104_00649	hoi108_00684	hoi110_01032	hoi111_01112	hoi112_02051	hoi113_02059	hoi114_02089	hoi115_00734	hoi116_01505	hoi105_01424	hoi107_01451	hoi109_01383	hoi117_01783	hoi106_01481
1869	hypothetical protein	16	Core			hoi102_01921	hoi103_00732	hoi104_00650	hoi108_00685	hoi110_01033	hoi111_01113	hoi112_02052	hoi113_02060	hoi114_02088	hoi115_00735	hoi116_01506	hoi105_01425	hoi107_01452	hoi109_01384	hoi117_01784	hoi106_01482
1870	Transcriptional regulator WhiB4	16	Core			hoi102_01922	hoi103_00733	hoi104_00651	hoi108_00686	hoi110_01034	hoi111_01114	hoi112_02053	hoi113_02061	hoi114_02087	hoi115_00736	hoi116_01507	hoi105_01426	hoi107_01453	hoi109_01385	hoi117_01785	hoi106_01483
1871	Penicillin-binding protein 1A	16	Core			hoi102_01923	hoi103_00734	hoi104_00652	hoi108_00687	hoi110_01035	hoi111_01115	hoi112_02054	hoi113_02062	hoi114_02086	hoi115_00737	hoi116_01508	hoi105_01427	hoi107_01454	hoi109_01386	hoi117_01786	hoi106_01484
1872	Yqey-like protein	16	Core			hoi102_01924	hoi103_00735	hoi104_00653	hoi108_00688	hoi110_01036	hoi111_01116	hoi112_02055	hoi113_02063	hoi114_02085	hoi115_00738	hoi116_01509	hoi105_01428	hoi107_01455	hoi109_01387	hoi117_01787	hoi106_01485
1873	phosphodiesterase YaeI	16	Core			hoi102_01925	hoi103_00736	hoi104_00654	hoi108_00689	hoi110_01037	hoi111_01117	hoi112_02056	hoi113_02064	hoi114_02084	hoi115_00739	hoi116_01510	hoi105_01429	hoi107_01456	hoi109_01388	hoi117_01788	hoi106_01486
1874	N-substituted formamide deformylase precursor	16	Core			hoi102_01927	hoi103_00738	hoi104_00656	hoi108_00691	hoi110_01039	hoi111_01119	hoi112_02058	hoi113_02066	hoi114_02082	hoi115_00741	hoi116_01512	hoi105_01431	hoi107_01458	hoi109_01390	hoi117_01790	hoi106_01488
1875	Proline/betaine transporter	12	Accessory			hoi102_01928	hoi103_00739	hoi104_00657	hoi108_00692	hoi110_01040	hoi111_01120	hoi112_02059	hoi113_02067	hoi114_02081	hoi115_00742	hoi116_01513					hoi106_01489
1876	pH-gated potassium channel KcsA	16	Core			hoi102_01929	hoi103_00740	hoi104_00658	hoi108_00693	hoi110_01041	hoi111_01121	hoi112_02060	hoi113_02068	hoi114_02080	hoi115_00743	hoi116_01514	hoi105_01432	hoi107_01459	hoi109_01391	hoi117_01791	hoi106_01490
1877	hypothetical protein	16	Core			hoi102_01930	hoi103_00741	hoi104_00659	hoi108_00694	hoi110_01042	hoi111_01122	hoi112_02061	hoi113_02069	hoi114_02079	hoi115_00744	hoi116_01515	hoi105_01433	hoi107_01460	hoi109_01392	hoi117_01792	hoi106_01491
1878	Na(+)/H(+) antiporter subunit A	16	Core			hoi102_01931	hoi103_00742	hoi104_00660	hoi108_00695	hoi110_01043	hoi111_01123	hoi112_02062	hoi113_02070	hoi114_02078	hoi115_00745	hoi116_01516	hoi105_01434	hoi107_01461	hoi109_01393	hoi117_01793	hoi106_01492
1879	Na(+)/H(+) antiporter subunit C1	16	Core			hoi102_01932	hoi103_00743	hoi104_00661	hoi108_00696	hoi110_01044	hoi111_01124	hoi112_02063	hoi113_02071	hoi114_02077	hoi115_00746	hoi116_01517	hoi105_01435	hoi107_01462	hoi109_01394	hoi117_01794	hoi106_01493
1880	Na(+)/H(+) antiporter subunit D	16	Core			hoi102_01933	hoi103_00744	hoi104_00662	hoi108_00697	hoi110_01045	hoi111_01125	hoi112_02064	hoi113_02072	hoi114_02076	hoi115_00747	hoi116_01518	hoi105_01436	hoi107_01463	hoi109_01395	hoi117_01795	hoi106_01494
1881	putative monovalent cation/H+ antiporter subun	16	Core			hoi102_01934	hoi103_00745	hoi104_00663	hoi108_00698	hoi110_01046	hoi111_01126	hoi112_02065	hoi113_02073	hoi114_02075	hoi115_00748	hoi116_01519	hoi105_01437	hoi107_01464	hoi109_01396	hoi117_01796	hoi106_01495
1882	putative monovalent cation/H+ antiporter subun	16	Core			hoi102_01935	hoi103_00746	hoi104_00664	hoi108_00699	hoi110_01047	hoi111_01127	hoi112_02066	hoi113_02074	hoi114_02074	hoi115_00749	hoi116_01520	hoi105_01438	hoi107_01465	hoi109_01397	hoi117_01797	hoi106_01496
1883	putative monovalent cation/H+ antiporter subun	16	Core			hoi102_01936	hoi103_00747	hoi104_00665	hoi108_00700	hoi110_01048	hoi111_01128	hoi112_02067	hoi113_02075	hoi114_02073	hoi115_00750	hoi116_01521	hoi105_01439	hoi107_01466	hoi109_01398	hoi117_01798	hoi106_01497
1884	hypothetical protein	16	Core			hoi102_01937	hoi103_00748	hoi104_00666	hoi108_00701	hoi110_01049	hoi111_01129	hoi112_02068	hoi113_02076	hoi114_02072	hoi115_00751	hoi116_01522	hoi105_01440	hoi107_01467	hoi109_01399	hoi117_01799	hoi106_01498
1885	Citrate transporter	16	Core			hoi102_01938	hoi103_00749	hoi104_00667	hoi108_00702	hoi110_01050	hoi111_01130	hoi112_02069	hoi113_02077	hoi114_02071	hoi115_00752	hoi116_01523	hoi105_01441	hoi107_01468	hoi109_01400	hoi117_01800	hoi106_01499
1886	hypothetical protein	5	Accessory	GI9		hoi102_01939		hoi104_00668	hoi108_00703						hoi115_00753				hoi109_01401		
1887	hypothetical protein	5	Accessory	GI9		hoi102_01940		hoi104_00669	hoi108_00704						hoi115_00754				hoi109_01402		
1888	hypothetical protein	5	Accessory	GI9		hoi102_01941		hoi104_00670	hoi108_00705						hoi115_00755				hoi109_01403		
1889	hypothetical protein	5	Accessory	GI9		hoi102_01942		hoi104_00671	hoi108_00706						hoi115_00756				hoi109_01404		
1890	hypothetical protein	5	Accessory	GI9	Φ_2	hoi102_01943		hoi104_00672	hoi108_00707						hoi115_00757				hoi109_01405		
1891	hypothetical protein	5	Accessory	GI9	Φ_2	hoi102_01944		hoi104_00673	hoi108_00708						hoi115_00758				hoi109_01406		
1892	hypothetical protein	5	Accessory	GI9	Φ_2	hoi102_01945		hoi104_00674	hoi108_00709						hoi115_00759				hoi109_01407		
1893	hypothetical protein	5	Accessory	GI9	Φ_2	hoi102_01946		hoi104_00675	hoi108_00710						hoi115_00760				hoi109_01408		
1894	hypothetical protein	5	Accessory	GI9	Φ_2	hoi102_01947		hoi104_00676	hoi108_00711						hoi115_00761				hoi109_01409		
1895	N-acetylmuramoyl-L-alanine amidase	5	Accessory	GI9	Φ_2	hoi102_01948		hoi104_00677	hoi108_00712						hoi115_00762				hoi109_01410		
1896	hypothetical protein	3	Accessory	GI9	Φ_2	hoi102_01949		hoi104_00678	hoi108_00713												
1897	hypothetical protein	5	Accessory	GI9	Φ_2	hoi102_01950		hoi104_00679	hoi108_00714						hoi115_02062				hoi109_02089		
1898	Phage tail repeat like protein	5	Accessory	GI9	Φ_2	hoi102_01951		hoi104_00680	hoi108_00715						hoi115_02063				hoi109_02090		
1899	hypothetical protein	5	Accessory	GI9	Φ_2	hoi102_01952		hoi104_00681	hoi108_00716						hoi115_02064				hoi109_02091		
1900	hypothetical protein	5	Accessory	GI9	Φ_2	hoi102_01953		hoi104_00682	hoi108_00717						hoi115_02065				hoi109_02092		
1901	hypothetical protein	5	Accessory	GI9	Φ_2	hoi102_01954		hoi104_00683	hoi108_00718						hoi115_02066				hoi109_02093		
1902	hypothetical protein	5	Accessory	GI9	Φ_2	hoi102_01955		hoi104_00684	hoi108_00719						hoi115_02067				hoi109_02094		
1903	Chromosome partition protein Smc	5	Accessory	GI9	Φ_2	hoi102_01956		hoi104_00685	hoi108_00720						hoi115_02068				hoi109_02095		
1904	hypothetical protein	5	Accessory	GI9	Φ_2	hoi102_01957		hoi104_00686	hoi108_00721						hoi115_02069				hoi109_02096		
1905	hypothetical protein	5	Accessory	GI9	Φ_2	hoi102_01958		hoi104_00687	hoi108_00722						hoi115_02070				hoi109_02097		
1906	hypothetical protein	5	Accessory	GI9	Φ_2	hoi102_01959		hoi104_00688	hoi108_00723						hoi115_02071				hoi109_02098		
1907	hypothetical protein	5	Accessory	GI9	Φ_2	hoi102_01960		hoi104_00689	hoi108_00724						hoi115_02072				hoi109_02099		
1908	hypothetical protein	5	Accessory	GI9	Φ_2	hoi102_01961		hoi104_00690	hoi108_00725						hoi115_02073				hoi109_02100		
1909	hypothetical protein	5	Accessory	GI9	Φ_2	hoi102_01962		hoi104_00691	hoi108_00726						hoi115_02074				hoi109_02101		
1910	Phage protein Gp19/Gp15/Gp42	5	Accessory	GI9	Φ_2	hoi102_01963		hoi104_00692	hoi108_00727						hoi115_02075				hoi109_02102		
1911	hypothetical protein	5	Accessory	GI9	Φ_2	hoi102_01964		hoi104_00693	hoi108_00728						hoi115_02076				hoi109_02103		
1912	hypothetical protein	5	Accessory	GI9	Φ_2	hoi102_01965		hoi104_00694	hoi108_00729						hoi115_02077				hoi109_02104		
1913	hypothetical protein	5	Accessory	GI9	Φ_2	hoi102_01966		hoi104_00695	hoi108_00730						hoi115_02078				hoi109_02105		
1914	ATP-dependent Clp protease proteolytic subunit	5	Accessory	GI9	Φ_2	hoi102_01967		hoi104_00696	hoi108_00731						hoi115_02079				hoi109_02106		
1915	hypothetical protein	5	Accessory	GI9	Φ_2	hoi102_01968		hoi104_00697	hoi108_00732						hoi115_02080				hoi109_02107		
1916	Phage Terminase	5	Accessory	GI9	Φ_2	hoi102_01969		hoi104_00698	hoi108_00733						hoi115_02081				hoi109_02108		
1917	hypothetical protein	5	Accessory	GI9	Φ_2	hoi102_01970		hoi104_00699	hoi108_00734						hoi115_02082				hoi109_02109		

1918	hypothetical protein	5	Accessory	GI9	Φ_2	hoi102_01971		hoi104_00700	hoi108_00735						hoi115_02083				hoi109_02110		
1919	hypothetical protein	5	Accessory	GI9	Φ_2	hoi102_01972		hoi104_00701	hoi108_00736						hoi115_02084				hoi109_02111		
1920	hypothetical protein	5	Accessory	GI9	Φ_2	hoi102_01973		hoi104_00702	hoi108_00737						hoi115_02085				hoi109_02112		
1921	hypothetical protein	5	Accessory	GI9	Φ_2	hoi102_01974		hoi104_00703	hoi108_00738						hoi115_02086				hoi109_02113		
1922	Secreted effector protein pipB2	5	Accessory	GI9	Φ_2	hoi102_01975		hoi104_00704	hoi108_00739						hoi115_02087				hoi109_02114		
1923	hypothetical protein	5	Accessory	GI9	Φ_2	hoi102_01976		hoi104_00705	hoi108_00740						hoi115_02088				hoi109_02115		
1924	Endodeoxyribonuclease RusA	5	Accessory	GI9	Φ_2	hoi102_01977		hoi104_00706	hoi108_00741						hoi115_02089				hoi109_02116		
1925	Deoxyuridine 5'-triphosphate nucleotidohydrola:	5	Accessory	GI9	Φ_2	hoi102_01978		hoi104_00707	hoi108_00742						hoi115_02090				hoi109_02117		
1926	hypothetical protein	5	Accessory	GI9	Φ_2	hoi102_01979		hoi104_00708	hoi108_00743						hoi115_02091				hoi109_02118		
1927	phosphoadenosine phosphosulfate reductase	5	Accessory	GI9	Φ_2	hoi102_01980		hoi104_00709	hoi108_00744						hoi115_02092				hoi109_02119		
1928	D12 class N6 adenine-specific DNA methyltransfe	5	Accessory	GI9		hoi102_01981		hoi104_00710	hoi108_00745						hoi115_02093				hoi109_02120		
1929	hypothetical protein	5	Accessory	GI9		hoi102_01982		hoi104_00711	hoi108_00746						hoi115_02094				hoi109_02121		
1930	hypothetical protein	5	Accessory	GI9		hoi102_01983		hoi104_00712	hoi108_00747						hoi115_02095				hoi109_02122		
1931	hypothetical protein	5	Accessory	GI9		hoi102_01984		hoi104_00713	hoi108_00748						hoi115_02096				hoi109_02123		
1932	hypothetical protein	5	Accessory	GI9		hoi102_01985		hoi104_00714	hoi108_00749						hoi115_02097				hoi109_02124		
1933	CRISPR system Cascade subunit CasE	5	Accessory	GI9		hoi102_01986		hoi104_00715	hoi108_00750						hoi115_02098				hoi109_02125		
1934	Single-stranded DNA-binding protein	5	Accessory	GI9		hoi102_01987		hoi104_00716	hoi108_00751						hoi115_02099				hoi109_02126		
1935	hypothetical protein	4	Accessory	GI9		hoi102_01988		hoi104_00717	hoi108_00752						hoi115_02100						
1936	hypothetical protein	5	Accessory	GI9		hoi102_01989		hoi104_00718	hoi108_00753						hoi115_02101				hoi109_02127		
1937	ERF superfamily protein	5	Accessory	GI9		hoi102_01990		hoi104_00719	hoi108_00754						hoi115_02102				hoi109_02128		
1938	YqaJ-like viral recombinase domain protein	5	Accessory	GI9		hoi102_01991		hoi104_00720	hoi108_00755						hoi115_02103				hoi109_02129		
1939	hypothetical protein	6	Accessory	GI9		hoi102_01992		hoi104_00721	hoi108_00756						hoi115_02104				hoi109_02130		hoi106_00414
1940	hypothetical protein	5	Accessory	GI9		hoi102_01993		hoi104_00722	hoi108_00757						hoi115_02105				hoi109_02131		
1941	hypothetical protein	5	Accessory	GI9		hoi102_01994		hoi104_00723	hoi108_00758						hoi115_02106				hoi109_02132		
1942	Helix-turn-helix domain protein	5	Accessory	GI9		hoi102_01995		hoi104_00724	hoi108_00759						hoi115_02107				hoi109_02133		
1943	hypothetical protein	5	Accessory	GI9		hoi102_01996		hoi104_00725	hoi108_00760						hoi115_02108				hoi109_02134		
1944	hypothetical protein	5	Accessory	GI9		hoi102_01997		hoi104_00726	hoi108_00761						hoi115_02109				hoi109_02135		
1945	hypothetical protein	5	Accessory	GI9		hoi102_01998		hoi104_00727	hoi108_00762						hoi115_02110				hoi109_02136		
1946	hypothetical protein	5	Accessory	GI9		hoi102_01999		hoi104_00728	hoi108_00763						hoi115_02111				hoi109_02137		
1947	hypothetical protein	5	Accessory	GI9		hoi102_02000		hoi104_00729	hoi108_00764						hoi115_02112				hoi109_02138		
1948	hypothetical protein	5	Accessory	GI9		hoi102_02001		hoi104_00730	hoi108_00765						hoi115_02113				hoi109_02139		
1949	Transposase from transposon Tn916	5	Accessory	GI9		hoi102_02002		hoi104_00731	hoi108_00766						hoi115_02114				hoi109_02140		
1950	Citrate transporter	5	Accessory	GI9		hoi102_02003		hoi104_00732	hoi108_00767						hoi115_02141				hoi109_02141		
1951	HTH-type transcriptional regulator QacR	16	Core			hoi102_02004	hoi103_00750	hoi104_00733	hoi108_00768	hoi110_01051	hoi111_01131	hoi112_02070	hoi113_02078	hoi114_02070	hoi115_02142	hoi116_01524	hoi105_01442	hoi107_01469	hoi109_02142	hoi117_01801	hoi106_01500
1952	3-hydroxyisobutyrate dehydrogenase	16	Core			hoi102_02005	hoi103_00751	hoi104_00734	hoi108_00769	hoi110_01052	hoi111_01132	hoi112_02071	hoi113_02079	hoi114_02069	hoi115_02143	hoi116_01525	hoi105_01443	hoi107_01470	hoi109_02143	hoi117_01802	hoi106_01501
1953	Methylmalonate-semialdehyde dehydrogenase [	16	Core			hoi102_02006	hoi103_00752	hoi104_00735	hoi108_00770	hoi110_01053	hoi111_01133	hoi112_02072	hoi113_02080	hoi114_02068	hoi115_02144	hoi116_01526	hoi105_01444	hoi107_01471	hoi109_02144	hoi117_01803	hoi106_01502
1954	Catalase	16	Core			hoi102_02007	hoi103_00753	hoi104_00736	hoi108_00771	hoi110_01054	hoi111_01134	hoi112_02073	hoi113_02081	hoi114_02067	hoi115_02145	hoi116_01527	hoi105_01445	hoi107_01472	hoi109_02145	hoi117_01804	hoi106_01503
1955	ECF RNA polymerase sigma factor SigC	16	Core			hoi102_02008	hoi103_00754	hoi104_00737	hoi108_00772	hoi110_01055	hoi111_01135	hoi112_02074	hoi113_02082	hoi114_02066	hoi115_02146	hoi116_01528	hoi105_01446	hoi107_01473	hoi109_02146	hoi117_01805	hoi106_01504
1956	Queuine tRNA-ribosyltransferase	16	Core			hoi102_02009	hoi103_00755	hoi104_00738	hoi108_00773	hoi110_01056	hoi111_01136	hoi112_02075	hoi113_02083	hoi114_02065	hoi115_02147	hoi116_01529	hoi105_01447	hoi107_01474	hoi109_02147	hoi117_01806	hoi106_01505
1957	Membrane protein YdfJ	16	Core			hoi102_02010	hoi103_00756	hoi104_00739	hoi108_00774	hoi110_01057	hoi111_01137	hoi112_02076	hoi113_02084	hoi114_02064	hoi115_02148	hoi116_01530	hoi105_01448	hoi107_01475	hoi109_02148	hoi117_01807	hoi106_01506
1958	Ribosomal large subunit pseudouridine synthase	16	Core			hoi102_02011	hoi103_00757	hoi104_00740	hoi108_00775	hoi110_01058	hoi111_01138	hoi112_02077	hoi113_02085	hoi114_02063	hoi115_02149	hoi116_01531	hoi105_01449	hoi107_01476	hoi109_02149	hoi117_01808	hoi106_01507
1959	putative ABC transporter ATP-binding protein/M	16	Core			hoi102_02012	hoi103_00758	hoi104_00741	hoi108_00776	hoi110_01059	hoi111_01139	hoi112_02078	hoi113_02086	hoi114_02062	hoi115_02150	hoi116_01532	hoi105_01450	hoi107_01477	hoi109_02150	hoi117_01809	hoi106_01508
1960	ABC transporter permease YtrF precursor	16	Core			hoi102_02013	hoi103_00759	hoi104_00742	hoi108_00777	hoi110_01060	hoi111_01140	hoi112_02079	hoi113_02087	hoi114_02061	hoi115_02151	hoi116_01533	hoi105_01451	hoi107_01478	hoi109_02151	hoi117_01810	hoi106_01509
1961	hypothetical protein	16	Core			hoi102_02014	hoi103_00760	hoi104_00743	hoi108_00778	hoi110_01061	hoi111_01141	hoi112_02080	hoi113_02088	hoi114_02060	hoi115_02152	hoi116_01534	hoi105_01452	hoi107_01479	hoi109_02152	hoi117_01811	hoi106_01510
1962	hypothetical protein	9	Accessory			hoi102_02015	hoi103_01948			hoi110_02118	hoi111_01914	hoi112_00462	hoi113_02115			hoi116_00954	hoi105_00873				hoi106_01290
1963	DNA-binding protein	16	Core			hoi102_02016	hoi103_01949	hoi104_02098	hoi108_01268	hoi110_02117	hoi111_01913	hoi112_00463	hoi113_02116	hoi114_00653	hoi115_00954	hoi116_00955	hoi105_00872	hoi107_00707	hoi109_00796	hoi117_01215	hoi106_01289
1964	hypothetical protein	16	Core			hoi102_02017	hoi103_01950	hoi104_02097	hoi108_01269	hoi110_02116	hoi111_01912	hoi112_00464	hoi113_02117	hoi114_00654	hoi115_00955	hoi116_00956	hoi105_00871	hoi107_00706	hoi109_00795	hoi117_01214	hoi106_01288
1965	(2E,6E)-farnesyl diphosphate synthase	16	Core			hoi102_02018	hoi103_01951	hoi104_02096	hoi108_01270	hoi110_02115	hoi111_01911	hoi112_00465	hoi113_02118	hoi114_00655	hoi115_00956	hoi116_00957	hoi105_00870	hoi107_00705	hoi109_00794	hoi117_01213	hoi106_01287
1966	5,10-methylenetetrahydrofolate reductase	16	Core			hoi102_02019	hoi103_01952	hoi104_02095	hoi108_01271	hoi110_02114	hoi111_01910	hoi112_00466	hoi113_02119	hoi114_00656	hoi115_00957	hoi116_00958	hoi105_00869	hoi107_00704	hoi109_00793	hoi117_01212	hoi106_01286
1967	Acetyltransferase (GNAT) family protein	16	Core			hoi102_02020	hoi103_01953	hoi104_02094	hoi108_01272	hoi110_02113	hoi111_01909	hoi112_00467	hoi113_02120	hoi114_00657	hoi115_00958	hoi116_00959	hoi105_00868	hoi107_00703	hoi109_00792	hoi117_01211	hoi106_01285
1968	hypothetical protein	16	Core			hoi102_02021	hoi103_01954	hoi104_02093	hoi108_01273	hoi110_02112	hoi111_01908	hoi112_00468	hoi113_02121	hoi114_00658	hoi115_00959	hoi116_00960	hoi105_00867	hoi107_00702	hoi109_00791	hoi117_01210	hoi106_01284
1969	hypothetical protein	16	Core			hoi102_02022	hoi103_01955	hoi104_02092	hoi108_01274	hoi110_02017	hoi111_01907	hoi112_00469	hoi113_00611	hoi114_00659	hoi115_00960	hoi116_00961	hoi105_00866	hoi107_00701	hoi109_00790	hoi117_01209	hoi106_01283
1970	cell division protein MraZ	16	Core			hoi102_02023	hoi103_01956	hoi104_02091	hoi108_01275	hoi110_02016	hoi111_01906	hoi112_00470	hoi113_00612	hoi114_00660	hoi115_00961	hoi116_00962	hoi105_00865	hoi107_00700	hoi109_00789	hoi117_01208	hoi106_01282
1971	Ribosomal RNA small subunit methyltransferase	16	Core			hoi102_02024	hoi103_01957	hoi104_02090	hoi108_01276	hoi110_02015	hoi111_01905	hoi112_00471	hoi113_00613	hoi114_00661	hoi115_00962	hoi116_00963	hoi105_00864	hoi107_00699	hoi109_00788	hoi117_01207	hoi106_01281
1972	hypothetical protein	16	Core			hoi102_02025	hoi103_01958	hoi104_02089	hoi108_01277	hoi110_02014	hoi111_01904	hoi112_00472	hoi113_00614	hoi114_00662	hoi115_00963	hoi116_00964	hoi105_00863	hoi107_00698	hoi109_00787	hoi117_01206	hoi106_01280
1973	Penicillin-binding protein PbpB	16	Core			hoi102_02026	hoi103_01959	hoi104_02088	hoi108_01278	hoi110_02013	hoi111_01903	hoi112_00473	hoi113_00615	hoi114_00663	hoi115_00964	hoi116_00965	hoi105_00862	hoi107_00697	hoi109_00786	hoi117_01205	hoi106_01279
1974	UDP-N-acetylmuramoyl-L-alanyl-D-glutamate--2,	16	Core			hoi102_02027	hoi103_01960	hoi104_02087	hoi108_01279	hoi110_02012	hoi111_01902	hoi112_00474	hoi113_00616	hoi114_00664	hoi115_00965	hoi116_00966	hoi105_00861	hoi107_00696	hoi109_00785	hoi117_01204	hoi106_01278
1975	UDP-N-acetylmuramoyl-tripeptide--D-alanyl-D-al	16	Core			hoi102_02028	hoi103_01961	hoi104_02086	hoi108_01280	hoi110_02011	hoi111_01901	hoi112_00475	hoi113_00617	hoi114_00665	hoi115_00966	hoi116_00967	hoi105_00860	hoi107_00695	hoi109_00784	hoi117_01203	hoi106_01277
1976	Phospho-N-acetylmuramoyl-pentapeptide-transf	16	Core			hoi102_02029	hoi103_01962	hoi104_02085	hoi108_01281	hoi110_02010	hoi111_01900	hoi112_00476	hoi113_00618	hoi114_00666	hoi115_00967	hoi116_00968	hoi105_00859	hoi107_00694	hoi109_00783	hoi117_01202	hoi106_01276
1977	UDP-N-acetylmuramoylalanine--D-glutamate liga	16	Core			hoi102_02030	hoi103_01963	hoi104_02084	hoi108_01282	hoi110_02009	hoi111_01899	hoi112_00477	hoi113_00619	hoi114_00667	hoi115_00968	hoi116_00969	hoi105_00858	hoi107_00693	hoi109_00782	hoi117_01201	hoi106_01275
1978	Lipid II flippase FtsW	16	Core			hoi102_02031	hoi103_01964	hoi104_02083	hoi108_01283	hoi110_02008	hoi111_01898	hoi112_00478	hoi113_00620	hoi114_00668	hoi115_00969	hoi116_00970	hoi105_00857	hoi107_00692	hoi109_00781	hoi117_01200	hoi106_01274
1979	UDP-N-acetylglucosamine--N-acetylmuramyl-(pe	16	Core			hoi102_02032	hoi103_01965	hoi104_02082	hoi108_01284	hoi110_02007	hoi111_01897	hoi112_00479	hoi113_00621	hoi114_00669	hoi115_00970	hoi116_00971	hoi105_00856	hoi107_00691	hoi109_00780	hoi117_01199	hoi106_01273
1980	UDP-N-acetylmuramate--L-alanine ligase	16	Core			hoi102_02033	hoi103_01966	hoi104_02081	hoi108_01285	hoi110_02006	hoi111_01896	hoi112_00480	hoi113_00622	hoi114_00670	hoi115_00971	hoi116_00972	hoi105_00855	hoi107_00690	hoi109_00779	hoi117_01198	hoi106_01272
1981	Cell division protein FtsQ	16	Core			hoi102_02034	hoi103_01967	hoi104_02080	hoi108_01286	hoi110_02005	hoi111_01895	hoi112_00481	hoi113_00623	hoi114_00671	hoi115_00972	hoi116_00973	hoi105_00854	hoi107_00689	hoi109_00778	hoi117_01197	hoi106_01271
1982	Cell division protein FtsZ	16	Core			hoi102_02035	hoi103_01968	hoi104_02079	hoi108_01287	hoi110_02004	hoi111_01894	hoi112_00482	hoi113_00624	hoi114_00672	hoi115_00973	hoi116_00974	hoi105_00853	hoi107_00688	hoi109_00777	hoi117_01196	hoi106_01270
1983	Laccase domain protein YfiH	16	Core			hoi102_02036	hoi103_01969	hoi104_02078	hoi108_01288	hoi110_02003	hoi111_01893	hoi112_00483	hoi113_00625	hoi114_00673	hoi115_00974	hoi116_00975	hoi105_00852	hoi107_00687	hoi109_00776	hoi117_01195	hoi106_01269
1984	hypothetical protein	16	Core			hoi102_02037	hoi103_01970	hoi104_02077	hoi108_01289	hoi110_02002	hoi111_01892	hoi112_00484	hoi113_00626	hoi114_00674	hoi115_00975	hoi116_00976	hoi105_00851	hoi107_00686	hoi109_00775	hoi117_01194	hoi106_01268
1985	Cell division protein SepF	16	Core			hoi102_02038	hoi103_01971	hoi104_02076	hoi108_01290	hoi110_02001	hoi111_01891	hoi112_00485	hoi113_00627	hoi114_00675	hoi115_00976	hoi116_00977	hoi105_00850	hoi107_00685	hoi109_00774	hoi117_01193	hoi106_01267
1986	YGGT family protein	16	Core			hoi102_02039	hoi103_01972	hoi104_02075	hoi108_01291	hoi110_02000	hoi111_01890	hoi112_00486	hoi113_00628	hoi114_00676	hoi115_00977	hoi116_00978	hoi105_00849	hoi107_00684	hoi109_00773	hoi117_01192	hoi106_01266
1987	Cell wall synthesis protein Wag31	16	Core			hoi102_02040	hoi103_01973	hoi104_02074	hoi108_01292	hoi110_01999	hoi111_01889	hoi112_00487	hoi113_00629	hoi114_00677	hoi115_00978	hoi116_00979	hoi105_00848	hoi107_00683	hoi109_00772	hoi117_01191	hoi106_01265
1988	Ferrous iron permease EfeU	16	Core			hoi102_02041	hoi103_01974	hoi104_02073	hoi108_01293	hoi110_01998	hoi111_01888	hoi112_00488	hoi113_00630	hoi114_00678	hoi115_00979	hoi116_00980	hoi105_00847	hoi107_00682	hoi109_00771	hoi117_01190	hoi106_01264
1989	hypothetical protein	16	Core			hoi102_02042	hoi103_01975	hoi104_02072	hoi108_01294	hoi110_01997	hoi111_01887	hoi112_00489	hoi113_00631	hoi114_00679	hoi115_00980	hoi116_00981	hoi105_00846	hoi107_00681	hoi109_00770	hoi117_01189	hoi106_01263
1990	Isoleucine--tRNA ligase	16	Core			hoi102_02043	hoi103_01976	hoi104_02071	hoi108_01295	hoi110_01996	hoi111_01886	hoi112_00490	hoi113_00632	hoi114_00680	hoi115_00981	hoi116_00982	hoi105_00845	hoi107_00680	hoi109_00769	hoi117_01188	hoi106_01262
1991	hypothetical protein	16	Core			hoi102_02044	hoi103_01977	hoi104_02070	hoi108_01296	hoi110_01995	hoi111_01885	hoi112_00491	hoi113_00633	hoi114_00681	hoi115_00982	hoi116_00983	hoi105_00844	hoi107_00679	hoi109_00768	hoi117_01187	hoi106_01261
1992	DNA polymerase IV	16	Core			hoi102_02045	hoi103_01978	hoi104_02069	hoi108_01297	hoi110_01994	hoi111_01884	hoi112_00492	hoi113_00634	hoi114_00682	hoi115_00983	hoi116_00984	hoi105_00843	hoi107_00678	hoi109_00767	hoi117_01186	hoi106_01260
1993	L-asparaginase	16	Core			hoi102_02046	hoi103_01979	hoi104_02068	hoi108_01298	hoi110_01993	hoi111_01883	hoi112_00493	hoi113_00635	hoi114_00683	hoi115_00984	hoi116_00985	hoi105_00842	hoi107_00677	hoi109_00766	hoi117_01185	hoi106_01259
1994	hypothetical protein	16	Core			hoi102_02047	hoi103_01980	hoi104_02067	hoi108_01299	hoi110_01992	hoi111_01882	hoi112_00494	hoi113_00636	hoi114_00684	hoi115_00985	hoi116_00986	hoi105_00841	hoi107_00676	hoi109_00765	hoi117_01184	hoi106_01258
1995	putative ABC transporter ATP-binding protein Yh	16	Core			hoi102_02048	hoi103_01981	hoi104_02066	hoi108_01300	hoi110_01991	hoi111_01881	hoi112_00495	hoi113_00637	hoi114_00685	hoi115_00986	hoi116_00987	hoi105_00840	hoi107_00675	hoi109_00764	hoi117_01183	hoi106_01257
1996	hypothetical protein	16	Core			hoi102_02049	hoi103_01982	hoi104_02065	hoi108_01301	hoi110_01990	hoi111_01880	hoi112_00496	hoi113_00638	hoi114_00686	hoi115_00987	hoi116_00988	hoi105_00839	hoi107_00674	hoi109_00763	hoi117_01182	hoi106_01256
1997	Lipoprotein signal peptidase	16	Core			hoi102_02050	hoi103_01983	hoi104_02064	hoi108_01302	hoi110_01989	hoi111_01879	hoi112_00497	hoi113_00639	hoi114_00687	hoi115_00988	hoi116_00989	hoi105_00838	hoi107_00673	hoi109_00762	hoi117_01181	hoi106_01255

1998	Ribosomal large subunit pseudouridine synthase	16	Core		hoi102_02051	hoi103_01984	hoi104_02063	hoi108_01303	hoi110_01988	hoi111_01878	hoi112_00498	hoi113_00640	hoi114_00688	hoi115_00989	hoi116_00990	hoi105_00837	hoi107_00672	hoi109_00761	hoi117_01180	hoi106_01254
1999	hypothetical protein	16	Core		hoi102_02052	hoi103_01985	hoi104_02062	hoi108_01304	hoi110_01987	hoi111_01877	hoi112_00499	hoi113_00641	hoi114_00689	hoi115_00990	hoi116_00991	hoi105_00836	hoi107_00671	hoi109_00760	hoi117_01179	hoi106_01253
2000	EamA-like transporter family protein	16	Core		hoi102_02053	hoi103_01986	hoi104_02061	hoi108_01305	hoi110_01986	hoi111_01876	hoi112_00500	hoi113_00642	hoi114_00690	hoi115_00991	hoi116_00992	hoi105_00835	hoi107_00670	hoi109_00759	hoi117_01178	hoi106_01252
2001	DNA polymerase III subunit alpha	16	Core		hoi102_02054	hoi103_01987	hoi104_02060	hoi108_01306	hoi110_01985	hoi111_01875	hoi112_00501	hoi113_00643	hoi114_00691	hoi115_00992	hoi116_00993	hoi105_00834	hoi107_00669	hoi109_00758	hoi117_01177	hoi106_01251
2002	L-threonine dehydratase biosynthetic IlvA	16	Core		hoi102_02055	hoi103_01988	hoi104_02059	hoi108_01307	hoi110_01984	hoi111_01874	hoi112_00502	hoi113_00644	hoi114_00692	hoi115_00993	hoi116_00994	hoi105_00833	hoi107_00668	hoi109_00757	hoi117_01176	hoi106_01250
2003	Malto-oligosyltrehalose trehalohydrolase	16	Core		hoi102_02056	hoi103_01989	hoi104_02058	hoi108_01308	hoi110_01983	hoi111_01873	hoi112_00503	hoi113_00645	hoi114_00693	hoi115_00994	hoi116_00995	hoi105_00832	hoi107_00667	hoi109_00756	hoi117_01175	hoi106_01249
2004	IMPACT family member YigZ	16	Core		hoi102_02057	hoi103_01990	hoi104_02057	hoi108_01309	hoi110_01982	hoi111_01872	hoi112_00504	hoi113_00646	hoi114_00694	hoi115_00995	hoi116_00996	hoi105_00831	hoi107_00666	hoi109_00755	hoi117_01174	hoi106_01248
2005	hypothetical protein	16	Core		hoi102_02058	hoi103_01991	hoi104_02056	hoi108_01310	hoi110_01981	hoi111_01871	hoi112_00505	hoi113_00647	hoi114_00695	hoi115_00996	hoi116_00997	hoi105_00830	hoi107_00665	hoi109_00754	hoi117_01173	hoi106_01247
2006	Heat shock protein 15	16	Core		hoi102_02059	hoi103_01992	hoi104_02055	hoi108_01311	hoi110_01980	hoi111_01870	hoi112_00506	hoi113_00648	hoi114_00696	hoi115_00997	hoi116_00998	hoi105_00829	hoi107_00664	hoi109_00753	hoi117_01172	hoi106_01246
2007	hypothetical protein	16	Core		hoi102_02060	hoi103_01993	hoi104_02054	hoi108_01312	hoi110_01979	hoi111_01869	hoi112_00507	hoi113_00649	hoi114_00697	hoi115_00998	hoi116_00999	hoi105_00828	hoi107_00663	hoi109_00752	hoi117_01171	hoi106_01245
2008	GTP pyrophosphokinase YjbM	16	Core		hoi102_02061	hoi103_01994	hoi104_02053	hoi108_01313	hoi110_01978	hoi111_01868	hoi112_00508	hoi113_00650	hoi114_00698	hoi115_00999	hoi116_01000	hoi105_00827	hoi107_00662	hoi109_00751	hoi117_01170	hoi106_01244
2009	Maltooligosyl trehalose synthase	16	Core		hoi102_02062	hoi103_01995	hoi104_02052	hoi108_01314	hoi110_01977	hoi111_01867	hoi112_00509	hoi113_00651	hoi114_00699	hoi115_01000	hoi116_01001	hoi105_00826	hoi107_00661	hoi109_00750	hoi117_01169	hoi106_01243
2010	hypothetical protein	16	Core		hoi102_02063	hoi103_01996	hoi104_02051	hoi108_01315	hoi110_01976	hoi111_01866	hoi112_00510	hoi113_00652	hoi114_00700	hoi115_01001	hoi116_01002	hoi105_00825	hoi107_00660	hoi109_00749	hoi117_01168	hoi106_01242
2011	NAD-dependent DNA ligase LigA	16	Core		hoi102_02064	hoi103_01997	hoi104_02050	hoi108_01316	hoi110_01975	hoi111_01865	hoi112_00511	hoi113_00653	hoi114_00701	hoi115_01002	hoi116_01003	hoi105_00824	hoi107_00659	hoi109_00748	hoi117_01167	hoi106_01241
2012	Glycogen debranching enzyme	16	Core		hoi102_02065	hoi103_01998	hoi104_02049	hoi108_01317	hoi110_01974	hoi111_01864	hoi112_00512	hoi113_00654	hoi114_00702	hoi115_01003	hoi116_01004	hoi105_00823	hoi107_00658	hoi109_00747	hoi117_01166	hoi106_01240
2013	Tetracycline repressor protein class E	16	Core		hoi102_02066	hoi103_01999	hoi104_02048	hoi108_01318	hoi110_01973	hoi111_01863	hoi112_00513	hoi113_00655	hoi114_00703	hoi115_01004	hoi116_01005	hoi105_00822	hoi107_00657	hoi109_00746	hoi117_01165	hoi106_01239
2014	hypothetical protein	16	Core		hoi102_02067	hoi103_02000	hoi104_02047	hoi108_01319	hoi110_01972	hoi111_01862	hoi112_00514	hoi113_00656	hoi114_00704	hoi115_01005	hoi116_01006	hoi105_00821	hoi107_00656	hoi109_00745	hoi117_01164	hoi106_01238
2015	hypothetical protein	16	Core		hoi102_02068	hoi103_02001	hoi104_02046	hoi108_01320	hoi110_01971	hoi111_01861	hoi112_00515	hoi113_00657	hoi114_00705	hoi115_01006	hoi116_01007	hoi105_00820	hoi107_00655	hoi109_00744	hoi117_01163	hoi106_01237
2016	Histidinol dehydrogenase	16	Core		hoi102_02069	hoi103_02002	hoi104_02045	hoi108_01321	hoi110_01970	hoi111_01860	hoi112_00516	hoi113_00658	hoi114_00706	hoi115_01007	hoi116_01008	hoi105_00819	hoi107_00654	hoi109_00743	hoi117_01162	hoi106_01236
2017	Histidinol-phosphate aminotransferase	16	Core		hoi102_02070	hoi103_02003	hoi104_02044	hoi108_01322	hoi110_01969	hoi111_01859	hoi112_00517	hoi113_00659	hoi114_00707	hoi115_01008	hoi116_01009	hoi105_00818	hoi107_00653	hoi109_00742	hoi117_01161	hoi106_01235
2018	Imidazoleglycerol-phosphate dehydratase	16	Core		hoi102_02071	hoi103_02004	hoi104_02043	hoi108_01323	hoi110_01968	hoi111_01858	hoi112_00518	hoi113_00660	hoi114_00708	hoi115_01009	hoi116_01010	hoi105_00817	hoi107_00652	hoi109_00741	hoi117_01160	hoi106_01234
2019	hypothetical protein	16	Core		hoi102_02072	hoi103_02005	hoi104_02042	hoi108_01324	hoi110_01967	hoi111_01857	hoi112_00519	hoi113_00661	hoi114_00709	hoi115_01010	hoi116_01011	hoi105_00816	hoi107_00651	hoi109_00740	hoi117_01159	hoi106_01233
2020	Major Facilitator Superfamily protein	16	Core		hoi102_02073	hoi103_02006	hoi104_02041	hoi108_01325	hoi110_01966	hoi111_01856	hoi112_00520	hoi113_00662	hoi114_00710	hoi115_01011	hoi116_01012	hoi105_00815	hoi107_00650	hoi109_00739	hoi117_01158	hoi106_01232
2021	Imidazole glycerol phosphate synthase subunit H	16	Core		hoi102_02074	hoi103_02007	hoi104_02040	hoi108_01326	hoi110_01965	hoi111_01855	hoi112_00521	hoi113_00663	hoi114_00711	hoi115_01012	hoi116_01013	hoi105_00814	hoi107_00649	hoi109_00738	hoi117_01157	hoi106_01231
2022	1-(5-phosphoribosyl)-5-[(5-phosphoribosylaminc	16	Core		hoi102_02075	hoi103_02008	hoi104_02039	hoi108_01327	hoi110_01964	hoi111_01854	hoi112_00522	hoi113_00664	hoi114_00712	hoi115_01013	hoi116_01014	hoi105_00813	hoi107_00648	hoi109_00737	hoi117_01156	hoi106_01230
2023	Inositol-1-monophosphatase ImpA	16	Core		hoi102_02076	hoi103_02009	hoi104_02038	hoi108_01328	hoi110_01963	hoi111_01853	hoi112_00523	hoi113_00665	hoi114_00713	hoi115_01014	hoi116_01015	hoi105_00812	hoi107_00647	hoi109_00736	hoi117_01155	hoi106_01229
2024	Imidazole glycerol phosphate synthase subunit H	16	Core		hoi102_02077	hoi103_02010	hoi104_02037	hoi108_01329	hoi110_01962	hoi111_01852	hoi112_00524	hoi113_00666	hoi114_00714	hoi115_01015	hoi116_01016	hoi105_00811	hoi107_00646	hoi109_00735	hoi117_01154	hoi106_01228
2025	phosphoribosyl-AMP cyclohydrolase	16	Core		hoi102_02078	hoi103_02011	hoi104_02036	hoi108_01330	hoi110_01961	hoi111_01851	hoi112_00525	hoi113_00667	hoi114_00715	hoi115_01016	hoi116_01017	hoi105_00810	hoi107_00645	hoi109_00734	hoi117_01153	hoi106_01227
2026	Tryptophan-associated transmembrane protein (	16	Core		hoi102_02079	hoi103_02012	hoi104_02035	hoi108_01331	hoi110_01960	hoi111_01850	hoi112_00526	hoi113_00668	hoi114_00716	hoi115_01017	hoi116_01018	hoi105_00809	hoi107_00644	hoi109_00733	hoi117_01152	hoi106_01226
2027	Indole-3-glycerol phosphate synthase	16	Core		hoi102_02080	hoi103_02013	hoi104_02034	hoi108_01332	hoi110_01959	hoi111_01849	hoi112_00527	hoi113_00669	hoi114_00717	hoi115_01018	hoi116_01019	hoi105_00808	hoi107_00643	hoi109_00732	hoi117_01151	hoi106_01225
2028	Prolipoprotein diacylglyceryl transferase	16	Core		hoi102_02081	hoi103_02014	hoi104_02033	hoi108_01333	hoi110_01958	hoi111_01848	hoi112_00528	hoi113_00670	hoi114_00718	hoi115_01019	hoi116_01020	hoi105_00807	hoi107_00642	hoi109_00731	hoi117_01150	hoi106_01224
2029	Pyruvate kinase	16	Core		hoi102_02082	hoi103_02015	hoi104_02032	hoi108_01334	hoi110_01957	hoi111_01847	hoi112_00529	hoi113_00671	hoi114_00719	hoi115_01020	hoi116_01021	hoi105_00806	hoi107_00641	hoi109_00730	hoi117_01149	hoi106_01223
2030	putative hydrolase YxeP	16	Core		hoi102_02083	hoi103_02016	hoi104_02031	hoi108_01335	hoi110_01956	hoi111_01846	hoi112_00530	hoi113_00672	hoi114_00720	hoi115_01021	hoi116_01022	hoi105_00805	hoi107_00640	hoi109_00729	hoi117_01148	hoi106_01222
2031	putative phosphomannomutase	16	Core		hoi102_02084	hoi103_02017	hoi104_01854	hoi108_02036	hoi110_01153	hoi111_01986	hoi112_01929	hoi113_01839	hoi114_01893	hoi115_01735	hoi116_01626	hoi105_02055	hoi107_01697	hoi109_02031	hoi117_01561	hoi106_01569
2032	anaerobic benzoate catabolism transcriptional re	16	Core		hoi102_02085	hoi103_02018	hoi104_01853	hoi108_02037	hoi110_01154	hoi111_01987	hoi112_01930	hoi113_01840	hoi114_01894	hoi115_01736	hoi116_01627	hoi105_02056	hoi107_01698	hoi109_02032	hoi117_01562	hoi106_01568
2033	Uracil phosphoribosyltransferase	16	Core		hoi102_02086	hoi103_02019	hoi104_01852	hoi108_02038	hoi110_01155	hoi111_01988	hoi112_01931	hoi113_01841	hoi114_01895	hoi115_01737	hoi116_01628	hoi105_02057	hoi107_01699	hoi109_02033	hoi117_01563	hoi106_01567
2034	hypothetical protein	16	Core		hoi102_02087	hoi103_02020	hoi104_01851	hoi108_02039	hoi110_01156	hoi111_01989	hoi112_01932	hoi113_01842	hoi114_01896	hoi115_01738	hoi116_01629	hoi105_02058	hoi107_01700	hoi109_02034	hoi117_01564	hoi106_01566
2035	putative endopeptidase precursor	16	Core		hoi102_02088	hoi103_02021	hoi104_01850	hoi108_02040	hoi110_01157	hoi111_01990	hoi112_01933	hoi113_01843	hoi114_01897	hoi115_01739	hoi116_01630	hoi105_02059	hoi107_01701	hoi109_02035	hoi117_01565	hoi106_01565
2036	hypothetical protein	16	Core		hoi102_02089	hoi103_02022	hoi104_01849	hoi108_02041	hoi110_01158	hoi111_01991	hoi112_01934	hoi113_01844	hoi114_01898	hoi115_01740	hoi116_01631	hoi105_02060	hoi107_01702	hoi109_02036	hoi117_01566	hoi106_01564
2037	Aminodeoxyfutalosine deaminase	16	Core		hoi102_02090	hoi103_02023	hoi104_01848	hoi108_02042	hoi110_01159	hoi111_01992	hoi112_01935	hoi113_01845	hoi114_01899	hoi115_01741	hoi116_01632	hoi105_02061	hoi107_01704	hoi109_02037	hoi117_01567	hoi106_01563
2038	D-alanyl-D-alanine carboxypeptidase DacB precu	16	Core		hoi102_02091	hoi103_02024	hoi104_01847	hoi108_02043	hoi110_01160	hoi111_01993	hoi112_01936	hoi113_01846	hoi114_01900	hoi115_01742	hoi116_01633	hoi105_02062	hoi107_01705	hoi109_02038	hoi117_01568	hoi106_01562
2039	RDD family protein	16	Core		hoi102_02092	hoi103_02025	hoi104_01846	hoi108_02044	hoi110_01161	hoi111_01994	hoi112_01937	hoi113_01847	hoi114_01901	hoi115_01743	hoi116_01634	hoi105_02063	hoi107_01706	hoi109_02039	hoi117_01569	hoi106_01561
2040	Inner membrane protein YhjD	16	Core		hoi102_02093	hoi103_02026	hoi104_01845	hoi108_02045	hoi110_01162	hoi111_01995	hoi112_01938	hoi113_01848	hoi114_01902	hoi115_01744	hoi116_01635	hoi105_02064	hoi107_01707	hoi109_02040	hoi117_01570	hoi106_01560
2041	Inner membrane protein YhjD	16	Core		hoi102_02094	hoi103_02027	hoi104_01844	hoi108_02046	hoi110_01163	hoi111_01996	hoi112_01939	hoi113_01849	hoi114_01903	hoi115_01745	hoi116_01636	hoi105_02065	hoi107_01708	hoi109_02041	hoi117_01571	hoi106_01559
2042	Tryptophan--tRNA ligase	16	Core		hoi102_02095	hoi103_02028	hoi104_01843	hoi108_02047	hoi110_01164	hoi111_01997	hoi112_01940	hoi113_01850	hoi114_01904	hoi115_01746	hoi116_01637	hoi105_02066	hoi107_01709	hoi109_02042	hoi117_01572	hoi106_01558
2043	Exodeoxyribonuclease	16	Core		hoi102_02096	hoi103_02029	hoi104_01842	hoi108_02048	hoi110_01165	hoi111_01998	hoi112_01941	hoi113_01851	hoi114_01905	hoi115_01747	hoi116_01638	hoi105_02067	hoi107_01710	hoi109_02043	hoi117_01573	hoi106_01557
2044	D-amino acid dehydrogenase small subunit	16	Core		hoi102_02097	hoi103_02030	hoi104_01841	hoi108_02049	hoi110_01166	hoi111_01999	hoi112_01942	hoi113_01852	hoi114_01906	hoi115_01748	hoi116_01639	hoi105_02068	hoi107_01711	hoi109_02044	hoi117_01574	hoi106_01556
2045	Inner membrane transport protein YdhP	16	Core		hoi102_02098	hoi103_02031	hoi104_01840	hoi108_02050	hoi110_01167	hoi111_02000	hoi112_01943	hoi113_01853	hoi114_01907	hoi115_01749	hoi116_01640	hoi105_02069	hoi107_01712	hoi109_02045	hoi117_01575	hoi106_01555
2046	Isocitrate dehydrogenase [NADP]	16	Core		hoi102_02099	hoi103_02032	hoi104_01839	hoi108_02051	hoi110_01168	hoi111_02001	hoi112_01944	hoi113_01854	hoi114_01908	hoi115_01750	hoi116_01641	hoi105_02070	hoi107_01713	hoi109_02046	hoi117_01576	hoi106_01554
2047	hypothetical protein	16	Core		hoi102_02100	hoi103_02033	hoi104_01838	hoi108_02052	hoi110_01169	hoi111_02002	hoi112_01945	hoi113_01855	hoi114_01909	hoi115_01751	hoi116_01642	hoi105_02071	hoi107_01714	hoi109_02047	hoi117_01577	hoi106_01553
2048	hypothetical protein	16	Core		hoi102_02101	hoi103_02034	hoi104_01837	hoi108_02053	hoi110_01170	hoi111_02003	hoi112_01946	hoi113_01856	hoi114_01910	hoi115_01752	hoi116_01643	hoi105_02072	hoi107_01715	hoi109_02048	hoi117_01578	hoi106_01552
2049	Alpha/beta hydrolase family protein	11	Accessory		hoi102_02102	hoi103_02035	hoi104_01836	hoi108_02054	hoi110_01171	hoi111_02004	hoi112_01947	hoi113_01857	hoi114_01911	hoi115_01753	hoi116_01644					
2050	Phosphomethylpyrimidine synthase	16	Core		hoi102_02103	hoi103_02036	hoi104_01835	hoi108_02055	hoi110_01172	hoi111_02005	hoi112_01948	hoi113_01858	hoi114_01912	hoi115_01754	hoi116_01645	hoi105_02073	hoi107_01716	hoi109_02049	hoi117_01579	hoi106_01551
2051	O-succinylhomoserine sulfhydrylase	16	Core		hoi102_02104	hoi103_02037	hoi104_02099	hoi108_02056	hoi110_01173	hoi111_02006	hoi112_01949	hoi113_01859	hoi114_01913	hoi115_01755	hoi116_01646	hoi105_02074	hoi107_01717	hoi109_02050	hoi117_01580	hoi106_01550
2052	Homoserine O-acetyltransferase	16	Core		hoi102_02105	hoi103_02038	hoi104_02100	hoi108_02057	hoi110_01174	hoi111_02007	hoi112_01950	hoi113_01860	hoi114_01914	hoi115_01756	hoi116_01647	hoi105_02075	hoi107_01718	hoi109_02051	hoi117_01581	hoi106_01549
2053	hypothetical protein	16	Core		hoi102_02106	hoi103_02039	hoi104_02101	hoi108_02058	hoi110_01175	hoi111_02008	hoi112_01951	hoi113_01861	hoi114_01915	hoi115_01757	hoi116_01648	hoi105_02076	hoi107_01719	hoi109_02052	hoi117_01582	hoi106_01548
2054	Bifunctional protein FolD protein	16	Core		hoi102_02107	hoi103_02040	hoi104_02102	hoi108_02059	hoi110_01176	hoi111_02009	hoi112_01952	hoi113_01862	hoi114_01916	hoi115_01758	hoi116_01649	hoi105_02077	hoi107_01720	hoi109_02053	hoi117_01583	hoi106_01547
2055	tRNA (cytidine(34)-2'-O)-methyltransferase	16	Core		hoi102_02108	hoi103_02041	hoi104_02103	hoi108_02060	hoi110_01177	hoi111_02010	hoi112_01953	hoi113_01863	hoi114_01917	hoi115_01759	hoi116_01650	hoi105_02078	hoi107_01721	hoi109_02054	hoi117_01584	hoi106_01546
2056	Bacterial membrane flanked domain protein	16	Core		hoi102_02109	hoi103_02042	hoi104_02104	hoi108_02061	hoi110_01178	hoi111_02011	hoi112_01954	hoi113_01864	hoi114_01918	hoi115_01760	hoi116_01651	hoi105_02079	hoi107_01722	hoi109_02055	hoi117_01585	hoi106_01545
2057	Bacterial membrane flanked domain protein	16	Core		hoi102_02110	hoi103_02043	hoi104_02105	hoi108_02062	hoi110_01179	hoi111_02012	hoi112_01955	hoi113_01865	hoi114_01919	hoi115_01761	hoi116_01652	hoi105_02080	hoi107_01723	hoi109_02056	hoi117_01586	hoi106_01544
2058	Error-prone DNA polymerase	16	Core		hoi102_02111	hoi103_02044	hoi104_02106	hoi108_02063	hoi110_01180	hoi111_02013	hoi112_01956	hoi113_01866	hoi114_01920	hoi115_01762	hoi116_01653	hoi105_02081	hoi107_01724	hoi109_02057	hoi117_01587	hoi106_01543
2059	D-methionine-binding lipoprotein MetQ precursc	16	Core		hoi102_02112	hoi103_02045	hoi104_02107	hoi108_02064	hoi110_01181	hoi111_02014	hoi112_01957	hoi113_01867	hoi114_01921	hoi115_01763	hoi116_01654	hoi105_02082	hoi107_01725	hoi109_02058	hoi117_01588	hoi106_01542
2060	Methionine import ATP-binding protein MetN 2	16	Core		hoi102_02113	hoi103_02046	hoi104_02108	hoi108_02065	hoi110_01182	hoi111_02015	hoi112_01958	hoi113_01868	hoi114_01922	hoi115_01764	hoi116_01655	hoi105_02083	hoi107_01726	hoi109_02059	hoi117_01589	hoi106_01541
2061	Methionine import system permease protein Me	16	Core		hoi102_02114	hoi103_02047	hoi104_02109	hoi108_02066	hoi110_01183	hoi111_02016	hoi112_01959	hoi113_01869	hoi114_01923	hoi115_01765	hoi116_01656	hoi105_02084	hoi107_01727	hoi109_02060	hoi117_01590	hoi106_01540
2062	CRISPR-associated endonuclease Cas9	16	Core	GI10	hoi102_02115	hoi103_02048	hoi104_02110	hoi108_02067	hoi110_01184	hoi111_02017	hoi112_01960	hoi113_01870	hoi114_01924	hoi115_01766	hoi116_01657	hoi105_02085	hoi107_01728	hoi109_02061	hoi117_01591	hoi106_01539
2063	CRISPR-associated endonuclease Cas9	1	Accessory	GI10																hoi106_01538
2064	CRISPR-associated endonuclease Cas1	16	Core	GI10	hoi102_02116	hoi103_02049	hoi104_02111	hoi108_02068	hoi110_01185	hoi111_02018	hoi112_01961	hoi113_01871	hoi114_01925	hoi115_01767	hoi116_01658	hoi105_02086	hoi107_01729	hoi109_02062	hoi117_01592	hoi106_01537
2065	CRISPR-associated endoribonuclease Cas2	16	Core	GI10	hoi102_02117	hoi103_02050	hoi104_02112	hoi108_02069	hoi110_01186	hoi111_02019	hoi112_01962	hoi113_01872	hoi114_01926	hoi115_01768	hoi116_01659	hoi105_02087	hoi107_01730	hoi109_02063	hoi117_01593	hoi106_01536
2066	putative FAD-linked oxidoreductase	16	Core		hoi102_02118	hoi103_02051	hoi104_02113	hoi108_02070	hoi110_01187	hoi111_02020	hoi112_01963	hoi113_01873	hoi114_01927	hoi115_01769	hoi116_01660	hoi105_02088	hoi107_01731	hoi109_02064	hoi117_01594	hoi106_01535
2067	Sucrase/ferredoxin-like protein	16	Core		hoi102_02119	hoi103_02052	hoi104_02114	hoi108_02071	hoi110_01188	hoi111_02021	hoi112_01964	hoi113_01874	hoi114_01928	hoi115_01770	hoi116_01661	hoi105_02089	hoi107_01732	hoi109_02065	hoi117_01595	hoi106_01534
2068	hypothetical protein	16	Core		hoi102_02120	hoi103_02053	hoi104_02115	hoi108_02072	hoi110_01189	hoi111_02022	hoi112_01965	hoi113_01875	hoi114_01929	hoi115_01771	hoi116_01662	hoi105_02090	hoi107_01733	hoi109_02066	hoi117_01596	hoi106_01533
2069	DNA polymerase IV	16	Core		hoi102_02121	hoi103_02054	hoi104_02116	hoi108_02073	hoi110_01190	hoi111_02023	hoi112_01966	hoi113_01876	hoi114_01930	hoi115_01772	hoi116_01663	hoi105_02091	hoi107_01734	hoi109_02067	hoi117_01597	hoi106_01532
2070	hypothetical protein	16	Core		hoi102_02122	hoi103_02055	hoi104_02117	hoi108_02074	hoi110_01191	hoi111_02024	hoi112_01967	hoi113_01877	hoi114_01931	hoi115_01773	hoi116_01664	hoi105_02092	hoi107_01735	hoi109_02068	hoi117_01598	hoi106_01531
2071	hypothetical protein	16	Core		hoi102_02123	hoi103_02056	hoi104_02118	hoi108_02075	hoi110_01192	hoi111_02025	hoi112_01968	hoi113_01878	hoi114_01932	hoi115_01774	hoi116_01665	hoi105_02093	hoi107_01736	hoi109_02069	hoi117_01599	hoi106_01530
2072	Ribonucleoside-diphosphate reductase subunit a	16	Core		hoi102_02124	hoi103_02057	hoi104_02119	hoi108_02076	hoi110_01193	hoi111_02026	hoi112_01969	hoi113_01879	hoi114_01933	hoi115_01775	hoi116_01666	hoi105_02094	hoi107_01737	hoi109_02070	hoi117_01600	hoi106_01529
2073	ribonucleotide reductase stimulatory protein	16	Core		hoi102_02125	hoi103_02058	hoi104_02120	hoi108_02077	hoi110_01194	hoi111_02027	hoi112_01970	hoi113_01880	hoi114_01934	hoi115_01776	hoi116_01667	hoi105_02095	hoi107_01738	hoi109_02071	hoi117_01601	hoi106_01528
2074	Glutaredoxin-like protein NrdH	16	Core		hoi102_02126	hoi103_02059	hoi104_02121	hoi108_02078	hoi110_01195	hoi111_02028	hoi112_01971	hoi113_01881	hoi114_01935	hoi115_01777	hoi116_01668	hoi105_02096	hoi107_01739	hoi109_02072	hoi117_01602	hoi106_01527
2075	50S ribosomal protein L36 2	16	Core		hoi102_02127	hoi103_02060	hoi104_02122	hoi108_02079	hoi110_01196	hoi111_02029	hoi112_01972	hoi113_01882	hoi114_01936	hoi115_01778	hoi116_01669	hoi105_02097	hoi107_01740	hoi109_02073	hoi117_01603	hoi106_01526
2076	NH(3)-dependent NAD(+) synthetase	16	Core		hoi102_02128	hoi103_02061	hoi104_02123	hoi108_02080	hoi110_01197	hoi111_02030	hoi112_01973	hoi113_01883	hoi114_01937	hoi115_01779	hoi116_01670	hoi105_02098	hoi107_01741	hoi109_02074	hoi117_01604	hoi106_01525
2077	Pyridoxamine 5'-phosphate oxidase	16	Core		hoi102_02129	hoi103_02062	hoi104_02124	hoi108_02081	hoi110_01198	hoi111_02031	hoi112_01974	hoi113_01884	hoi114_01938	hoi115_01780	hoi116_01671	hoi105_02099	hoi107_01742	hoi109_02075	hoi117_01605	hoi106_01524

2078	hypothetical protein	16	Core			hoi102_02130	hoi103_02063	hoi104_02125	hoi108_02082	hoi110_01199	hoi111_02032	hoi112_01975	hoi113_01885	hoi114_01939	hoi115_01781	hoi116_01672	hoi105_02100	hoi107_01743	hoi109_02076	hoi117_01606	hoi106_01523
2079	hypothetical protein	16	Core			hoi102_02131	hoi103_02064	hoi104_02126	hoi108_02083	hoi110_01200	hoi111_02033	hoi112_01976	hoi113_01886	hoi114_01940	hoi115_01782	hoi116_01673	hoi105_02101	hoi107_01744	hoi109_02077	hoi117_01607	hoi106_01522
2080	Bacterial regulatory proteins, tetR family	16	Core			hoi102_02132	hoi103_02065	hoi104_02127	hoi108_02084	hoi110_01201	hoi111_02034	hoi112_01977	hoi113_01887	hoi114_01941	hoi115_01783	hoi116_01674	hoi105_02102	hoi107_01745	hoi109_02078	hoi117_01608	hoi106_01521
2081	hypothetical protein	16	Core			hoi102_02133	hoi103_02066	hoi104_02128	hoi108_02085	hoi110_01202	hoi111_02035	hoi112_01978	hoi113_01888	hoi114_01942	hoi115_01784	hoi116_01675	hoi105_02103	hoi107_01746	hoi109_02079	hoi117_01609	hoi106_01520
2082	Host cell surface-exposed lipoprotein	16	Core			hoi102_02134	hoi103_02067	hoi104_02129	hoi108_02086	hoi110_01203	hoi111_02036	hoi112_01979	hoi113_01889	hoi114_01943	hoi115_01785	hoi116_01676	hoi105_02104	hoi107_01747	hoi109_02080	hoi117_01610	hoi106_01519
2083	hypothetical protein	16	Core			hoi102_02136	hoi103_02069	hoi104_02131	hoi108_02088	hoi110_01205	hoi111_02038	hoi112_01981	hoi113_01891	hoi114_01945	hoi115_01787	hoi116_01678	hoi105_02106	hoi107_01749	hoi109_02082	hoi117_01612	hoi106_01517
2084	DoxX	16	Core			hoi102_02137	hoi103_02070	hoi104_02132	hoi108_02089	hoi110_01206	hoi111_02039	hoi112_01982	hoi113_01892	hoi114_01946	hoi115_01788	hoi116_01679	hoi105_02107	hoi107_01750	hoi109_02083	hoi117_01613	hoi106_01516
2085	Phosphoglucomutase	16	Core			hoi102_02138	hoi103_02071	hoi104_02133	hoi108_02090	hoi110_01207	hoi111_02040	hoi112_01983	hoi113_01893	hoi114_01947	hoi115_01789	hoi116_01680	hoi105_02108	hoi107_01751	hoi109_02084	hoi117_01614	hoi106_01515
2086	Amino-acid carrier protein AlsT	16	Core			hoi102_02139	hoi103_02072	hoi104_02134	hoi108_02091	hoi110_01208	hoi111_02041	hoi112_01984	hoi113_01894	hoi114_01948	hoi115_01790	hoi116_01681	hoi105_02109	hoi107_01752	hoi109_02085	hoi117_01615	hoi106_01514
2087	camphor resistance protein CrcB	16	Core			hoi102_02140	hoi103_02073	hoi104_02135	hoi108_02092	hoi110_01209	hoi111_02042	hoi112_01985	hoi113_01895	hoi114_01949	hoi115_01791	hoi116_01682	hoi105_02110	hoi107_01753	hoi109_02086	hoi117_01616	hoi106_01513
2088	camphor resistance protein CrcB	16	Core			hoi102_02141	hoi103_02074	hoi104_02136	hoi108_02093	hoi110_01210	hoi111_02043	hoi112_01986	hoi113_01896	hoi114_01950	hoi115_01792	hoi116_01683	hoi105_02111	hoi107_01754	hoi109_02087	hoi117_01617	hoi106_01512
2089	hypothetical protein	16	Core			hoi102_02142	hoi103_02075	hoi104_02137	hoi108_02094	hoi110_01211	hoi111_02044	hoi112_01987	hoi113_01897	hoi114_01951	hoi115_01793	hoi116_01684	hoi105_02112	hoi107_01755	hoi109_02088	hoi117_01618	hoi106_01511
2090	hypothetical protein	16	Core			hoi102_02143	hoi103_02076	hoi104_02138	hoi108_02166	hoi110_02095	hoi111_02082	hoi112_02081	hoi113_02089	hoi114_01678	hoi115_02115	hoi116_02079	hoi105_02015	hoi107_01989	hoi109_02000	hoi117_01439	hoi106_02067
2091	Methylmalonyl-CoA carboxyltransferase 5S subu	16	Core			hoi102_02144	hoi103_02077	hoi104_02139	hoi108_02165	hoi110_02094	hoi111_02083	hoi112_02082	hoi113_02090	hoi114_01679	hoi115_02116	hoi116_02080	hoi105_02014	hoi107_01990	hoi109_02001	hoi117_01438	hoi106_02066
2092	Methylmalonyl-CoA carboxyltransferase 12S sub	16	Core			hoi102_02145	hoi103_02078	hoi104_02140	hoi108_02164	hoi110_02093	hoi111_02084	hoi112_02083	hoi113_02091	hoi114_01680	hoi115_02117	hoi116_02081	hoi105_02013	hoi107_01991	hoi109_02002	hoi117_01437	hoi106_02065
2093	Methylmalonyl-CoA carboxyltransferase 12S sub	16	Core			hoi102_02146	hoi103_02079	hoi104_02141	hoi108_02163	hoi110_02092	hoi111_02085	hoi112_02084	hoi113_02092	hoi114_01681	hoi115_02118	hoi116_02082	hoi105_02012	hoi107_01992	hoi109_02003	hoi117_01436	hoi106_02064
2094	Methylmalonyl-CoA carboxyltransferase 1.3S sub	16	Core			hoi102_02147	hoi103_02080	hoi104_02142	hoi108_02162	hoi110_02091	hoi111_02086	hoi112_02085	hoi113_02093	hoi114_01682	hoi115_02119	hoi116_02083	hoi105_02011	hoi107_01993	hoi109_02004	hoi117_01435	hoi106_02063
2095	Histidinol-phosphatase	16	Core			hoi102_02148	hoi103_02081	hoi104_02143	hoi108_02161	hoi110_02090	hoi111_02087	hoi112_02086	hoi113_02094	hoi114_01683	hoi115_02120	hoi116_02084	hoi105_02010	hoi107_01994	hoi109_02005	hoi117_01434	hoi106_02062
2096	Inositol-1-monophosphatase	16	Core			hoi102_02149	hoi103_02082	hoi104_02144	hoi108_02160	hoi110_02089	hoi111_02088	hoi112_02087	hoi113_02095	hoi114_01684	hoi115_02121	hoi116_02085	hoi105_02009	hoi107_01995	hoi109_02006	hoi117_01433	hoi106_02061
2097	Peptide chain release factor 2	16	Core			hoi102_02150	hoi103_02083	hoi104_02145	hoi108_02159	hoi110_02088	hoi111_02089	hoi112_02088	hoi113_02096	hoi114_01685	hoi115_02122	hoi116_02086	hoi105_02008	hoi107_01996	hoi109_02007	hoi117_01432	hoi106_02060
2098	Cell division ATP-binding protein FtsE	16	Core			hoi102_02151	hoi103_02084	hoi104_02146	hoi108_02158	hoi110_02087	hoi111_02090	hoi112_02089	hoi113_02097	hoi114_01686	hoi115_02123	hoi116_02087	hoi105_02007	hoi107_01997	hoi109_02008	hoi117_01431	hoi106_02059
2099	Cell division protein FtsX	16	Core			hoi102_02152	hoi103_02085	hoi104_02147	hoi108_02157	hoi110_02086	hoi111_02091	hoi112_02090	hoi113_02098	hoi114_01687	hoi115_02124	hoi116_02088	hoi105_02006	hoi107_01998	hoi109_02009	hoi117_01430	hoi106_02058
2100	putative ABC transporter solute-binding protein `	16	Core			hoi102_02153	hoi103_02086	hoi104_02148	hoi108_02156	hoi110_02085	hoi111_02092	hoi112_02091	hoi113_02099	hoi114_01688	hoi115_02125	hoi116_02089	hoi105_02005	hoi107_01999	hoi109_02010	hoi117_01429	hoi106_02057
2101	Iron-uptake system permease protein FeuB	16	Core			hoi102_02154	hoi103_02087	hoi104_02149	hoi108_02155	hoi110_02084	hoi111_02093	hoi112_02092	hoi113_02100	hoi114_01689	hoi115_02126	hoi116_02090	hoi105_02004	hoi107_02000	hoi109_02011	hoi117_01428	hoi106_02056
2102	Iron-uptake system permease protein FeuC	16	Core			hoi102_02155	hoi103_02088	hoi104_02150	hoi108_02154	hoi110_02083	hoi111_02094	hoi112_02093	hoi113_02101	hoi114_01690	hoi115_02127	hoi116_02091	hoi105_02003	hoi107_02001	hoi109_02012	hoi117_01427	hoi106_02055
2103	putative siderophore transport system ATP-bindi	16	Core			hoi102_02156	hoi103_02089	hoi104_02151	hoi108_02153	hoi110_02082	hoi111_02095	hoi112_02094	hoi113_02102	hoi114_01691	hoi115_02128	hoi116_02092	hoi105_02002	hoi107_02002	hoi109_02013	hoi117_01426	hoi106_02054
2104	SsrA-binding protein	16	Core			hoi102_02157	hoi103_02090	hoi104_02152	hoi108_02152	hoi110_02081	hoi111_02096	hoi112_02095	hoi113_02103	hoi114_01692	hoi115_02129	hoi116_02093	hoi105_02001	hoi107_02003	hoi109_02014	hoi117_01425	hoi106_02053
2105	site-specific tyrosine recombinase XerD	5	Accessory	GI11	Φ_3												hoi105_01999	hoi107_02005	hoi109_02016	hoi117_01423	hoi106_02051
2106	hypothetical protein	1	Accessory	GI11	Φ_3																hoi106_02050
2107	hypothetical protein	4	Accessory	GI11	Φ_3												hoi105_01998	hoi107_02006	hoi109_02017	hoi117_01422	
2108	Helix-turn-helix domain protein	5	Accessory	GI11	Φ_3												hoi105_01997	hoi107_02007	hoi109_02018	hoi117_01421	hoi106_02049
2109	hypothetical protein	5	Accessory	GI11	Φ_3												hoi105_01996	hoi107_02008	hoi109_02019	hoi117_01420	hoi106_02048
2110	hypothetical protein	5	Accessory	GI11	Φ_3												hoi105_01995	hoi107_02009	hoi109_02020	hoi117_01419	hoi106_02047
2111	hypothetical protein	1	Accessory	GI11	Φ_3																hoi106_02046
2112	hypothetical protein	5	Accessory	GI11	Φ_3												hoi105_01994	hoi107_02010	hoi109_02021	hoi117_01418	hoi106_02045
2113	hypothetical protein	16	Core			hoi102_02159	hoi103_02092	hoi104_02154	hoi108_02150	hoi110_02079	hoi111_02098	hoi112_02097	hoi113_02105	hoi114_01694	hoi115_02131	hoi116_02095	hoi105_01993	hoi107_02011	hoi109_02022	hoi117_01417	hoi106_02044
2114	hypothetical protein	16	Core			hoi102_02161	hoi103_02094	hoi104_02156	hoi108_02148	hoi110_02077	hoi111_02100	hoi112_02099	hoi113_02107	hoi114_01696	hoi115_02133	hoi116_02097	hoi105_01992	hoi107_02012	hoi109_02023	hoi117_01416	hoi106_02043
2115	hypothetical protein	16	Core			hoi102_02162	hoi103_02095	hoi104_02157	hoi108_02147	hoi110_02076	hoi111_02101	hoi112_02100	hoi113_02108	hoi114_01697	hoi115_02134	hoi116_02098	hoi105_01991	hoi107_02013	hoi109_02024	hoi117_01415	hoi106_02042
2116	Glucose 1-dehydrogenase 2	16	Core			hoi102_02163	hoi103_02096	hoi104_02158	hoi108_02146	hoi110_02075	hoi111_02102	hoi112_02101	hoi113_02109	hoi114_01698	hoi115_02135	hoi116_02099	hoi105_01990	hoi107_02014	hoi109_02025	hoi117_01414	hoi106_02041
2117	hypothetical protein	16	Core			hoi102_02164	hoi103_02097	hoi104_02159	hoi108_02145	hoi110_02074	hoi111_02103	hoi112_02102	hoi113_02110	hoi114_01699	hoi115_02136	hoi116_02100	hoi105_01989	hoi107_02015	hoi109_02026	hoi117_01413	hoi106_02040
2118	putative sialic acid transporter	16	Core			hoi102_02165	hoi103_02098	hoi104_02160	hoi108_02144	hoi110_02073	hoi111_02104	hoi112_02103	hoi113_02111	hoi114_01700	hoi115_02137	hoi116_02101	hoi105_01988	hoi107_02016	hoi109_02027	hoi117_01412	hoi106_02039
2119	Purine nucleoside phosphorylase DeoD-type	16	Core			hoi102_02166	hoi103_02099	hoi104_02161	hoi108_02143	hoi110_02072	hoi111_02105	hoi112_02104	hoi113_02112	hoi114_01701	hoi115_02138	hoi116_02102	hoi105_01987	hoi107_02017	hoi109_02028	hoi117_01411	hoi106_02038
2120	p-aminobenzoyl-glutamate transport protein	16	Core			hoi102_02167	hoi103_02100	hoi104_02162	hoi108_02142	hoi110_02071	hoi111_02106	hoi112_02105	hoi113_02113	hoi114_01702	hoi115_02139	hoi116_02103	hoi105_01986	hoi107_02018	hoi109_02029	hoi117_01410	hoi106_02037
2121	short chain dehydrogenase	16	Core			hoi102_02168	hoi103_02101	hoi104_02163	hoi108_02141	hoi110_02070	hoi111_02107	hoi112_02106	hoi113_02114	hoi114_01703	hoi115_02140	hoi116_02104	hoi105_01985	hoi107_02019	hoi109_02030	hoi117_01409	hoi106_02036
2122	hypothetical protein	11	Accessory			hoi102_02160	hoi103_02093	hoi104_02155	hoi108_02149	hoi110_02078	hoi111_02099	hoi112_02098	hoi113_02106	hoi114_01695	hoi115_02132	hoi116_02096					
2123	hypothetical protein	16	Core			hoi102_02169	hoi103_02102	hoi104_02164	hoi108_02182	hoi110_02096	hoi111_01410	hoi112_02107	hoi113_00595	hoi114_02116	hoi115_02153	hoi116_02105	hoi105_02141	hoi107_02133	hoi109_02219	hoi117_00590	hoi106_02083
2124	hypothetical protein	16	Core			hoi102_02170	hoi103_02103	hoi104_02165	hoi108_02181	hoi110_02097	hoi111_01411	hoi112_02108	hoi113_00596	hoi114_02115	hoi115_02154	hoi116_02106	hoi105_02142	hoi107_02134	hoi109_02218	hoi117_00591	hoi106_02084
2125	Putative TrmH family tRNA/rRNA methyltransfer	16	Core			hoi102_02171	hoi103_02104	hoi104_02166	hoi108_02180	hoi110_02098	hoi111_01412	hoi112_02109	hoi113_00597	hoi114_02114	hoi115_02155	hoi116_02107	hoi105_02143	hoi107_02135	hoi109_02217	hoi117_00592	hoi106_02085
2126	Threonine efflux protein	16	Core			hoi102_02172	hoi103_02105	hoi104_02167	hoi108_02179	hoi110_02099	hoi111_01413	hoi112_02110	hoi113_00598	hoi114_02113	hoi115_02156	hoi116_02108	hoi105_02144	hoi107_02136	hoi109_02216	hoi117_00593	hoi106_02086
2127	Guanine/hypoxanthine permease PbuG	16	Core			hoi102_02173	hoi103_02106	hoi104_02168	hoi108_02178	hoi110_02100	hoi111_01414	hoi112_02111	hoi113_00599	hoi114_02112	hoi115_02157	hoi116_02109	hoi105_02145	hoi107_02137	hoi109_02215	hoi117_00594	hoi106_02087
2128	hypothetical protein	16	Core			hoi102_02174	hoi103_02107	hoi104_02169	hoi108_02177	hoi110_02101	hoi111_01415	hoi112_02112	hoi113_00600	hoi114_02111	hoi115_02158	hoi116_02110	hoi105_02146	hoi107_02138	hoi109_02214	hoi117_00595	hoi106_02088
2129	hypothetical protein	16	Core			hoi102_02175	hoi103_02108	hoi104_02170	hoi108_02176	hoi110_02102	hoi111_01416	hoi112_02113	hoi113_00601	hoi114_02110	hoi115_02159	hoi116_02111	hoi105_02147	hoi107_02139	hoi109_02213	hoi117_00596	hoi106_02089
2130	Glutamine cyclotransferase	16	Core			hoi102_02176	hoi103_02109	hoi104_02171	hoi108_02175	hoi110_02103	hoi111_01417	hoi112_02114	hoi113_00602	hoi114_02109	hoi115_02160	hoi116_02112	hoi105_02148	hoi107_02140	hoi109_02212	hoi117_00597	hoi106_02090
2131	hypothetical protein	16	Core			hoi102_02177	hoi103_02110	hoi104_02172	hoi108_02174	hoi110_02104	hoi111_01418	hoi112_02115	hoi113_00603	hoi114_02108	hoi115_02161	hoi116_02113	hoi105_02149	hoi107_02141	hoi109_02211	hoi117_00598	hoi106_02091
2132	Major cold shock protein CspA	16	Core			hoi102_02178	hoi103_02111	hoi104_02173	hoi108_02173	hoi110_02105	hoi111_01419	hoi112_02116	hoi113_00604	hoi114_02107	hoi115_02162	hoi116_02114	hoi105_02150	hoi107_02142	hoi109_02210	hoi117_00599	hoi106_02092
2133	Resuscitation-promoting factor Rpf2 precursor	16	Core			hoi102_02179	hoi103_02112	hoi104_02174	hoi108_02172	hoi110_02106	hoi111_01420	hoi112_02117	hoi113_00605	hoi114_02106	hoi115_02163	hoi116_02115	hoi105_02151	hoi107_02143	hoi109_02209	hoi117_00600	hoi106_02093
2134	hypothetical protein	16	Core			hoi102_02180	hoi103_02113	hoi104_02175	hoi108_02171	hoi110_02107	hoi111_01421	hoi112_02118	hoi113_00606	hoi114_02105	hoi115_02164	hoi116_02116	hoi105_02152	hoi107_02144	hoi109_02208	hoi117_00601	hoi106_02094
2135	hypothetical protein	16	Core			hoi102_02181	hoi103_02114	hoi104_02176	hoi108_02170	hoi110_02108	hoi111_01422	hoi112_02119	hoi113_00607	hoi114_02104	hoi115_02165	hoi116_02117	hoi105_02153	hoi107_02145	hoi109_02207	hoi117_00602	hoi106_02095
2136	hypothetical protein	16	Core			hoi102_02182	hoi103_02115	hoi104_02177	hoi108_02169	hoi110_02109	hoi111_01423	hoi112_02120	hoi113_00608	hoi114_02103	hoi115_02166	hoi116_02118	hoi105_02154	hoi107_02146	hoi109_02206	hoi117_00603	hoi106_02096
2137	putative N-succinyldiaminopimelate aminotransf	16	Core			hoi102_02183	hoi103_02116	hoi104_02178	hoi108_02168	hoi110_02110	hoi111_01424	hoi112_02121	hoi113_00609	hoi114_02102	hoi115_02167	hoi116_02119	hoi105_02155	hoi107_02147	hoi109_02205	hoi117_00604	hoi106_02097
2138	Polyketide cyclase / dehydrase and lipid transpor	16	Core			hoi102_02184	hoi103_02117	hoi104_02179	hoi108_02167	hoi110_02111	hoi111_01425	hoi112_02122	hoi113_00610	hoi114_02101	hoi115_02168	hoi116_02120	hoi105_02156	hoi107_02148	hoi109_02204	hoi117_00605	hoi106_02098
2139	hypothetical protein	13	Accessory			hoi102_02186	hoi103_02119	hoi104_02181	hoi108_02183	hoi110_02120	hoi111_02124		hoi113_02123		hoi115_02183		hoi105_02158	hoi107_02159	hoi109_02221	hoi117_02157	hoi106_02126
2140	hypothetical protein	1	Accessory			hoi102_02187															
2141	Periplasmic dipeptide transport protein precurso	16	Core			hoi102_02188	hoi103_01170	hoi104_02185	hoi108_01663	hoi110_02121	hoi111_00193	hoi112_01419	hoi113_02047	hoi114_01156	hoi115_01794	hoi116_00538	hoi105_02139	hoi107_00902	hoi109_01685	hoi117_00607	hoi106_00922
2142	hypothetical protein	16	Core			hoi102_02189	hoi103_01169	hoi104_02184	hoi108_01664	hoi110_02122	hoi111_00194	hoi112_01420	hoi113_02046	hoi114_01157	hoi115_01795	hoi116_00537	hoi105_02138	hoi107_00903	hoi109_01684	hoi117_00608	hoi106_00923
2143	Group 2 truncated hemoglobin GlbO	16	Core			hoi102_02190	hoi103_01168	hoi104_02183	hoi108_01665	hoi110_02123	hoi111_00195	hoi112_01421	hoi113_02045	hoi114_01158	hoi115_01796	hoi116_00536	hoi105_02137	hoi107_00904	hoi109_01683	hoi117_00609	hoi106_00924
2144	hypothetical protein	16	Core			hoi102_02191	hoi103_01167	hoi104_02182	hoi108_01666	hoi110_02124	hoi111_00196	hoi112_01422	hoi113_02044	hoi114_01159	hoi115_01797	hoi116_00535	hoi105_02136	hoi107_00905	hoi109_01682	hoi117_00610	hoi106_00925
2145	hypothetical protein	5	Accessory														hoi105_00056	hoi107_00055	hoi109_00535	hoi117_00534	hoi106_00054
2146	type I restriction enzyme EcoKI subunit R	5	Accessory														hoi105_00105	hoi107_00105	hoi109_00486	hoi117_00485	hoi106_00104
2147	CTP pyrophosphohydrolase	5	Accessory														hoi105_00106	hoi107_00106	hoi109_00485	hoi117_00484	hoi106_00105
2148	2,3-dihydroxyphenylpropionate/2,3-dihydroxicin	5	Accessory														hoi105_00107	hoi107_00107	hoi109_00484	hoi117_00483	hoi106_00106
2149	2-keto-4-pentenoate hydratase	5	Accessory														hoi105_00108	hoi107_00108	hoi109_00483	hoi117_00482	hoi106_00107
2150	Acetaldehyde dehydrogenase	5	Accessory														hoi105_00109	hoi107_00109	hoi109_00482	hoi117_00481	hoi106_00108
2151	4-hydroxy-2-oxovalerate aldolase	5	Accessory														hoi105_00110	hoi107_00110	hoi109_00481	hoi117_00480	hoi106_00109
2152	Acetate operon repressor	5	Accessory	GI12													hoi105_00111	hoi107_00111	hoi109_00480	hoi117_00479	hoi106_00110
2153	3-phenylpropionate/cinnamic acid dioxygenase s	5	Accessory	GI12													hoi105_00112	hoi107_00112	hoi109_00479	hoi117_00478	hoi106_00111
2154	3-phenylpropionate/cinnamic acid dioxygenase f	5	Accessory	GI12													hoi105_00113	hoi107_00113	hoi109_00478	hoi117_00477	hoi106_00112
2155	Benzene 1,2-dioxygenase system ferredoxin--NA	5	Accessory	GI12													hoi105_00114	hoi107_00114	hoi109_00477	hoi117_00476	hoi106_00113
2156	Biphenyl dioxygenase subunit beta	5	Accessory	GI12													hoi105_00115	hoi107_00115	hoi109_00476	hoi117_00475	hoi106_00114
2157	Cis-2,3-dihydrobiphenyl-2,3-diol dehydrogenase	5	Accessory	GI12													hoi105_00116	hoi107_00116	hoi109_00475	hoi117_00474	hoi106_00115

2158	Long-chain-fatty-acid--CoA ligase FadD13	5	Accessory	GI12				hoi105_00117	hoi107_00117	hoi109_00474	hoi117_00473	hoi106_00116
2159	hypothetical protein	5	Accessory	GI12				hoi105_00118	hoi107_00118	hoi109_00473	hoi117_00472	hoi106_00117
2160	hypothetical protein	5	Accessory	GI12				hoi105_00119	hoi107_00119	hoi109_00472	hoi117_00471	hoi106_00118
2161	2-hydroxymuconate semialdehyde hydrolase	5	Accessory					hoi105_00120	hoi107_00120	hoi109_00471	hoi117_00470	hoi106_00119
2162	Gamma-aminobutyraldehyde dehydrogenase	5	Accessory					hoi105_00577	hoi107_01106	hoi109_01120	hoi117_00845	hoi106_00598
2163	Putrescine oxidase	5	Accessory					hoi105_00578	hoi107_01105	hoi109_01121	hoi117_00844	hoi106_00597
2164	Universal stress protein family protein	5	Accessory					hoi105_00579	hoi107_01104	hoi109_01122	hoi117_00843	hoi106_00596
2165	hypothetical protein	3	Accessory					hoi105_00713		hoi109_00637		hoi106_01774
2166	hypothetical protein	1	Accessory					hoi105_00874				
2167	Kynurenine formamidase	4	Accessory					hoi105_01028	hoi107_02058	hoi109_00039	hoi117_00039	
2168	hypothetical protein	4	Accessory					hoi105_01076	hoi107_00717	hoi109_00806	hoi117_01225	
2169	hypothetical protein	5	Accessory					hoi105_01103	hoi107_00744	hoi109_00833	hoi117_01252	hoi106_00764
2170	hypothetical protein	4	Accessory					hoi105_01260	hoi107_01288	hoi109_01219	hoi117_01619	
2171	hypothetical protein	5	Accessory					hoi105_01262	hoi107_01290	hoi109_01221	hoi117_01621	hoi106_01676
2172	hypothetical protein	3	Accessory					hoi105_01264		hoi109_01223	hoi117_01623	
2173	hypothetical protein	4	Accessory					hoi105_01265	hoi107_01292	hoi109_01224	hoi117_01624	
2174	hypothetical protein	4	Accessory					hoi105_01266	hoi107_01293	hoi109_01225	hoi117_01625	
2175	hypothetical protein	4	Accessory					hoi105_01267	hoi107_01294	hoi109_01226	hoi117_01626	
2176	hypothetical protein	4	Accessory					hoi105_01268	hoi107_01295	hoi109_01227	hoi117_01627	
2177	hypothetical protein	4	Accessory					hoi105_01269	hoi107_01296	hoi109_01228	hoi117_01628	
2178	hypothetical protein	4	Accessory					hoi105_01270	hoi107_01297	hoi109_01229	hoi117_01629	
2179	hypothetical protein	4	Accessory					hoi105_01271	hoi107_01298	hoi109_01230	hoi117_01630	
2180	succinate dehydrogenase/fumarate reductase iro	4	Accessory					hoi105_01346	hoi107_01373	hoi109_01305	hoi117_01705	
2181	Putrescine oxidase	4	Accessory					hoi105_01393	hoi107_01420	hoi109_01352	hoi117_01752	
2182	hypothetical protein	4	Accessory					hoi105_01397	hoi107_01424	hoi109_01356	hoi117_01756	
2183	hypothetical protein	3	Accessory					hoi105_01536		hoi109_01494	hoi117_01895	
2184	CRISPR-associated nuclease/helicase Cas3	4	Accessory	GI13				hoi105_01538	hoi107_01564	hoi109_01496	hoi117_01897	
2185	CRISPR-associated protein CasA/Cse1	4	Accessory	GI13				hoi105_01539	hoi107_01565	hoi109_01497	hoi117_01898	
2186	CRISPR-associated protein Cse2 (CRISPR_cse2)	4	Accessory	GI13				hoi105_01540	hoi107_01566	hoi109_01498	hoi117_01899	
2187	CRISPR system Cascade subunit CasC	4	Accessory	GI13				hoi105_01541	hoi107_01567	hoi109_01499	hoi117_01900	
2188	CRISPR system Cascade subunit CasD	4	Accessory	GI13				hoi105_01542	hoi107_01568	hoi109_01500	hoi117_01901	
2189	CRISPR-associated endoribonuclease Cse3	4	Accessory	GI13				hoi105_01543	hoi107_01569	hoi109_01501	hoi117_01902	
2190	CRISPR-associated endonuclease Cas1	4	Accessory	GI13				hoi105_01544	hoi107_01570	hoi109_01502	hoi117_01903	
2191	hypothetical protein	5	Accessory					hoi105_01558	hoi107_01584	hoi109_01516	hoi117_01917	hoi106_01157
2192	Ubiquinone/menaquinone biosynthesis C-methy	5	Accessory					hoi105_01559	hoi107_01585	hoi109_01517	hoi117_01918	hoi106_01158
2193	3-phenylpropionate/cinnamic acid dioxygenase s	4	Accessory					hoi105_01673	hoi107_00965	hoi109_01622	hoi117_00670	
2194	Biphenyl dioxygenase subunit beta	4	Accessory					hoi105_01674	hoi107_00964	hoi109_01623	hoi117_00669	
2195	hypothetical protein	4	Accessory					hoi105_01675	hoi107_00963	hoi109_01624	hoi117_00668	
2196	hypothetical protein	4	Accessory					hoi105_01676	hoi107_00962	hoi109_01625	hoi117_00667	
2197	Inner membrane metabolite transport protein Yh	4	Accessory					hoi105_01677	hoi107_00961	hoi109_01626	hoi117_00666	
2198	hypothetical protein	5	Accessory					hoi105_01795	hoi107_01841	hoi109_01719	hoi117_02032	hoi106_01377
2199	Toxin Doc	4	Accessory					hoi105_01824	hoi107_01870	hoi109_01689	hoi117_02062	
2200	hypothetical protein	4	Accessory					hoi105_01825	hoi107_01871	hoi109_01688	hoi117_02063	
2201	hypothetical protein	8	Accessory		hoi110_01954	hoi111_01771	hoi116_01933	hoi105_01909	hoi107_01874	hoi109_01885	hoi117_02066	hoi106_01673
2202	hypothetical protein	4	Accessory					hoi105_01984	hoi107_01949	hoi109_01960		hoi106_01597
2203	Excalibur calcium-binding domain protein	5	Accessory					hoi105_02119	hoi107_00922	hoi109_01665	hoi117_00627	hoi106_00942
2204	Glutathione transport system permease protein (	4	Accessory			hoi111_00192	hoi116_00539	hoi105_02140	hoi107_00901			
2205	hypothetical protein	5	Accessory					hoi105_02054	hoi107_01950	hoi109_01961	hoi117_01478	hoi106_02035
2206	hypothetical protein	5	Accessory					hoi105_02053	hoi107_01951	hoi109_01962	hoi117_01477	hoi106_02034
2207	hypothetical protein	5	Accessory	GI14				hoi105_02052	hoi107_01952	hoi109_01963	hoi117_01476	hoi106_02033
2208	Cutinase	5	Accessory	GI14				hoi105_02051	hoi107_01953	hoi109_01964	hoi117_01475	hoi106_02032
2209	hypothetical protein	5	Accessory	GI14				hoi105_02050	hoi107_01954	hoi109_01965	hoi117_01474	hoi106_02031
2210	TrbC/VIRB2 family protein	5	Accessory	GI14				hoi105_02049	hoi107_01955	hoi109_01966	hoi117_01473	hoi106_02030
2211	hypothetical protein	5	Accessory	GI14				hoi105_02048	hoi107_01956	hoi109_01967	hoi117_01472	hoi106_02029
2212	hypothetical protein	5	Accessory	GI14				hoi105_02047	hoi107_01957	hoi109_01968	hoi117_01471	hoi106_02028
2213	hypothetical protein	5	Accessory	GI14				hoi105_02046	hoi107_01958	hoi109_01969	hoi117_01470	hoi106_02027
2214	hypothetical protein	5	Accessory	GI14				hoi105_02045	hoi107_01959	hoi109_01970	hoi117_01469	hoi106_02026
2215	hypothetical protein	5	Accessory	GI14				hoi105_02044	hoi107_01960	hoi109_01971	hoi117_01468	hoi106_02025
2216	hypothetical protein	5	Accessory	GI14				hoi105_02043	hoi107_01961	hoi109_01972	hoi117_01467	hoi106_02024
2217	AAA-like domain protein	5	Accessory	GI14				hoi105_02042	hoi107_01962	hoi109_01973	hoi117_01466	hoi106_02023
2218	Murein DD-endopeptidase MepM	5	Accessory	GI14				hoi105_02041	hoi107_01963	hoi109_01974	hoi117_01465	hoi106_02022
2219	hypothetical protein	5	Accessory	GI14				hoi105_02040	hoi107_01964	hoi109_01975	hoi117_01464	hoi106_02021
2220	putative permease	5	Accessory	GI14				hoi105_02039	hoi107_01965	hoi109_01976	hoi117_01463	hoi106_02020
2221	hypothetical protein	5	Accessory	GI14				hoi105_02038	hoi107_01966	hoi109_01977	hoi117_01462	hoi106_02019
2222	hypothetical protein	5	Accessory	GI14				hoi105_02037	hoi107_01967	hoi109_01978	hoi117_01461	hoi106_02018
2223	TraM recognition site of TraD and TraG	5	Accessory	GI14				hoi105_02036	hoi107_01968	hoi109_01979	hoi117_01460	hoi106_02017
2224	hypothetical protein	5	Accessory	GI14				hoi105_02035	hoi107_01969	hoi109_01980	hoi117_01459	hoi106_02016
2225	hypothetical protein	5	Accessory	GI14				hoi105_02034	hoi107_01970	hoi109_01981	hoi117_01458	hoi106_02015
2226	ATP-dependent RecD-like DNA helicase	5	Accessory	GI14				hoi105_02033	hoi107_01971	hoi109_01982	hoi117_01457	hoi106_02014
2227	hypothetical protein	3	Accessory	GI14				hoi105_02032		hoi109_01983	hoi117_01456	
2228	hypothetical protein	1	Accessory	GI14					hoi107_01972			
2229	hypothetical protein	5	Accessory	GI14				hoi105_02031	hoi107_01973	hoi109_01984	hoi117_01455	hoi106_02013
2230	hypothetical protein	5	Accessory	GI14				hoi105_02030	hoi107_01974	hoi109_01985	hoi117_01454	hoi106_02012
2231	hypothetical protein	5	Accessory	GI14				hoi105_02029	hoi107_01975	hoi109_01986	hoi117_01453	hoi106_02011
2232	hypothetical protein	5	Accessory	GI14				hoi105_02028	hoi107_01976	hoi109_01987	hoi117_01452	hoi106_02010
2233	hypothetical protein	5	Accessory	GI14				hoi105_02027	hoi107_01977	hoi109_01988	hoi117_01451	hoi106_02009
2234	Sporulation initiation inhibitor protein Soj	5	Accessory	GI14				hoi105_02026	hoi107_01978	hoi109_01989	hoi117_01450	hoi106_02008
2235	hypothetical protein	5	Accessory	GI14				hoi105_02025	hoi107_01979	hoi109_01990	hoi117_01449	hoi106_02007
2236	hypothetical protein	1	Accessory									hoi106_00010
2237	tRNA(fMet)-specific endonuclease VapC	1	Accessory									hoi106_00011

2238	hypothetical protein	1	Accessory															hoi106_00078
2239	hypothetical protein	1	Accessory															hoi106_00079
2240	Adenylate and Guanylate cyclase catalytic domai	1	Accessory	GI15														hoi106_00178
2241	Adenylate and Guanylate cyclase catalytic domai	1	Accessory	GI15														hoi106_00179
2242	hypothetical protein	1	Accessory	GI15														hoi106_00180
2243	hypothetical protein	1	Accessory	GI15														hoi106_00181
2244	hypothetical protein	1	Accessory															hoi106_00254
2245	Bifunctional protein PutA	1	Accessory															hoi106_00411
2246	hypothetical protein	1	Accessory															hoi106_00413
2247	hypothetical protein	3	Accessory													hoi109_01686	hoi117_00606	hoi106_00921
2248	hypothetical protein	1	Accessory															hoi106_01423
2249	hypothetical protein	1	Accessory															hoi106_01654
2250	hypothetical protein	7	Accessory				hoi110_01955	hoi111_01770					hoi116_01932		hoi107_01873	hoi109_01884	hoi117_02065	hoi106_01674
2251	hypothetical protein	1	Accessory															hoi106_01752
2252	hypothetical protein	1	Accessory															hoi106_01763
2253	hypothetical protein	1	Accessory															hoi106_01870
2254	hypothetical protein	1	Accessory															hoi106_01871
2255	hypothetical protein	2	Accessory												hoi107_01614			hoi106_01892
2256	hypothetical protein	1	Accessory															hoi106_01946
2257	hypothetical protein	1	Accessory															hoi106_02125
2258	hypothetical protein	2	Accessory													hoi109_00331	hoi117_00330	
2259	hypothetical protein	1	Accessory														hoi117_02047	
2260	hypothetical protein	1	Accessory													hoi109_01690		
2261	type I restriction enzyme EcoKI subunit R	1	Accessory												hoi107_00104			
2262	hypothetical protein	1	Accessory												hoi107_01016			
2263	hypothetical protein	1	Accessory												hoi107_01154			
2264	hypothetical protein	1	Accessory												hoi107_01703			
2265	hypothetical protein	1	Accessory										hoi116_01273					
2266	Fructose-bisphosphate aldolase	1	Accessory										hoi116_02122					
2267	High-affinity choline transport protein	6	Accessory		hoi104_00299		hoi110_00077	hoi111_00878	hoi112_00077	hoi113_00385		hoi115_00077						
2268	hypothetical protein	1	Accessory								hoi114_00198							
2269	hypothetical protein	1	Accessory								hoi114_00850							
2270	hypothetical protein	4	Accessory				hoi110_01740	hoi111_01300	hoi112_01728	hoi113_00485								
2271	DNA polymerase III subunit tau	1	Accessory			hoi108_00224												
2272	hypothetical protein	1	Accessory		hoi104_01193													
