# Supplementary material for: Phylogenomic Characterization of a Novel Corynebacterium Species Associated with Fatal Diphtheritic Stomatitis in Endangered Yellow-Eyed Penguins
Source: mSystems. 2021 Jun 8;6(3):e00320-21. doi: 10.1128/mSystems.00320-21 (PMC8269222; doi:10.1128/mSystems.00320-21)
Supplement: TABLE S3 [file msystems.00320-21-st003.pdf]

Isolate	Lineage	infected	dead
C. ulcerans NZRM 818	+ve control	30	30
C. petrophilum NZRM 2522	-ve control	30	1
PBS	-ve control	30	2*
3B	1	30	1
6A	1	30	1
C. pseudotuberculosis NZRM 3004	+ve control	10	10
19B	1	10	0
48B	1	10	0
49B	1	10	0
50A	1	10	0
52A	1	10	0
71B	1	10	3*
7B	2	10	0
11A	2	10	1*
12B	2	10	0
20A	2	10	0
74A	2	10	0

*Organisms resembling the insect pathogen *Bacillus thuringensis* were isolated from 2 of the 3 dead larvae YEP 71b caterpillars, the dead YEP 11a caterpillar and 1 of the dead PBS caterpillars.
