# Supplementary material for: Phylogenomic Characterization of a Novel Corynebacterium Species Associated with Fatal Diphtheritic Stomatitis in Endangered Yellow-Eyed Penguins
Source: mSystems. 2021 Jun 8;6(3):e00320-21. doi: 10.1128/mSystems.00320-21 (PMC8269222; doi:10.1128/mSystems.00320-21)
Supplement: TABLE S4 [file msystems.00320-21-st004.pdf]

Gene	Function	Lineage	Amplicon	Oligo	Tm	GC mol%	seq
hoi102_00398	Hypothetical protein	1	274 bp	PL1_3F	58.84	55	CGGGCAAGAAGAAACTGAGG
				PL1_3R	58.91	55	GAACCCTGATTCGCCAACTC
hoi102_02102	α/β hydrolase family protein	1	219 bp	PL1_4F	59.07	55	GTATGGCTCTTTCGCTGCTC
				PL1_4R	59.02	50	TAGCCAACGATGTCCACCTT
hoi105_01995	Hypothetical protein	2	486 bp	PL2_5F	58.83	55	TGAGGAAGAGGTACGGTTCG
				PL2_5R	59.11	55	GTGCGGGACTGGTAGTTAGT
hoi105_02053	Hypothetical protein	2	425 bp	PL2_6F	59.07	50	TGGGGTTGACTGGAAAGGTT
				PL2_6R	59.06	55	CGCGGTATCCACATCAAAGG
